# Semi‐Reduction of Allenes to Access Deuterated Allylic Isotopomers, Isotopologs and Enantioisotopomers

**DOI:** 10.1002/anie.202525850

**Published:** 2026-02-12

**Authors:** Lihan Qi, Raviraj Ananda Thorat, Brad D. Maxwell, Jeffery A. Gladding, Aniel J. Rivera Arzola, Shashank P. Sancheti, Reilly E. Sonstrom, Xulin Tang, Isaac J. Anderson, Brooks H. Pate, Joseph R. Clark

**Affiliations:** ^1^ Department of Chemistry University of Tennessee Knoxville Tennessee USA; ^2^ Process Chemistry Vertex Pharmaceuticals Incorporated Boston Massachusetts USA; ^3^ BrightSpec Inc. Charlottesville Virginia USA; ^4^ Department of Chemistry Marquette University Milwaukee Wisconsin USA; ^5^ Department of Chemistry University of Virginia Charlottesville Virginia USA

**Keywords:** allenes, copper catalysis, deuteration, molecular rotational resonance spectroscopy, semi‐reduction

## Abstract

Selectively deuterated compounds represent high value synthetic targets with applications across many scientific disciplines. Despite their importance, reactions that enable access to products precisely deuterated at an allylic position with complete control over the degree of deuteration are extremely rare. In fact, the high enantiopurity synthesis of enantioisotopomers owing their chirality solely to hydrogen isotopes at an allylic position has remained elusive to date. Herein, we report a modular Cu‐catalyzed semi‐reductive deuteration of allenes to access a broad scope of small molecules, drug analogs, and natural product analogs precisely deuterated at allylic positions. The semi‐reduction strategy has been applied to access a range of precisely labeled *d1*‐, *d2*‐, *d3*‐, *d5*‐, and *d7*‐isotopologs. In this work, we disclose the first high enantiopurity synthesis of allylic‐*d1* enantioisotopomers, along with a highly accurate and precise analysis for enantiomeric excess (EE) determination and assignment of absolute configuration (AC) using molecular rotational resonance (MRR) spectroscopy.

## Graphical abstract

**Modular and Precise Deuteration**: The synthesis of molecules precisely deuterated at allylic, vinylic or both positions remains a challenging task. This work discloses a modular semi‐reductive deuteration that enables the synthesis of various isotopologs including precisely labeled *d1*‐, *d2*‐, *d3*‐, *d5*‐, and *d7*‐products. The method was extended to achieve chirality transfer to make *d1*‐allylic enantioisotopomers with high enantiopurity, which were analyzed using chiral‐tag MRR spectroscopy for measuring enantiomeric excess and assigning absolute configuration.

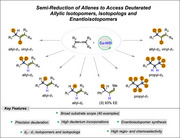

## 1 Introduction

**FIGURE 1:**
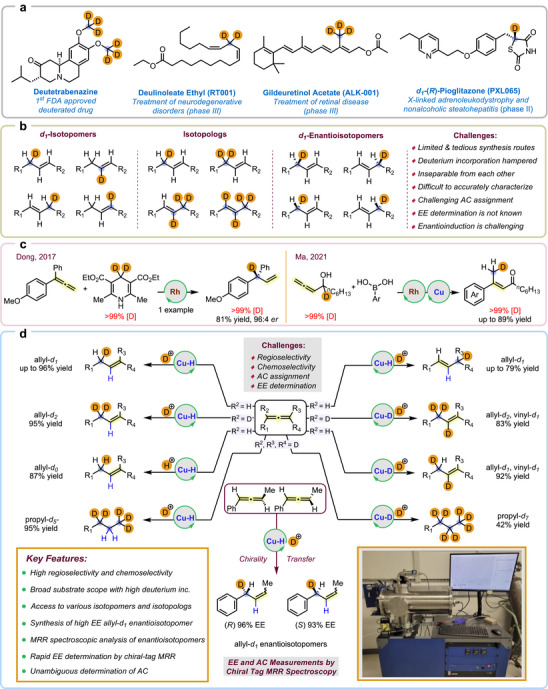
Deuterated drugs and catalytic synthesis of allyl‐*d*_*n*_ compounds.

Selectively deuterated molecules impact a broad scope of scientific disciplines, especially biological, and chemical sciences [1]. By enabling the precise measurement of kinetic isotope effects, they provide critical insights into the fundamental steps governing chemical transformations [2, 3, 4, 5, 6, 7, 8, 9, 10, 11, 12]. Apart from these applications, deuterated molecules are used in various spectroscopic techniques as internal standards, solvents, etc. The precise positioning of deuterium may also enhance drug safety and may affect pharmacokinetic properties, making it a key tool in drug development [13, 14]. As a result, there have been four Food and Drug Administration (FDA) approvals for deuterated drugs in recent years and many more are in clinical trials (Figure 1a) [15]. Of those in clinical trials, at least four current drug candidates contain deuterium at a stereogenic center.

An important yet understudied class in the category of chiral and deuterated compounds is enantioisotopomers, wherein the chiral center bears a hydrogen and a deuterium. These compounds have driven transformative breakthroughs in unraveling complex biological processes and biochemical reaction mechanisms, polymer chemistry, spectroscopy, and in understanding chirality [16, 17, 18, 19, 20, 21, 22, 23, 24, 25, 26, 27]. In the context of our research programs, we reported a Cu‐catalyzed approach that enabled the first enantioselective synthesis of certain enantioisotopomers, and new spectroscopic techniques for determining EE and assigning AC in these molecules [27].

An important class of isotopically labeled molecules are those precisely deuterated at an allylic position. The low oxidation potential of many allylic C─H bonds makes them prone to metabolic oxidation [28]. Owing to the stronger C─D bonds, these allylic sites present as important deuteration targets in drug development. For example, both deulinoleate ethyl and gildeuretinol acetate are under clinical investigation [29, 30], and each one contains a selectively deuterated allylic position (Figure 1a). In a tamoxifen metabolic study, formation of a carcinogenic metabolite was avoided in the analog deuterated at the allylic position [31, 32]. As evident, the intended application dictates the required degree, position(s) and stereochemical arrangement(s) of deuterium(s) in these molecules. Therefore, methods that enable precision deuteration, where regio‐ and stereoselective installation of deuterium along with complete control over the degree of deuterium incorporation are of high scientific importance and industrial relevance.

While compounds precisely deuterated at an allylic position may appear to be comprised of only a relatively basic set of labeling patterns, the different isotopic variants for this class of deuterated molecules actually consist of many permutations of isotopomers, isotopologs, and enantioisotopomers, with some representative variations shown in Figure 1b. Deuterated isotopomers refer to compounds that contain the same number of deuterium isotopes but differ in their positions. Deuterated isotopologs refer to compounds that differ only in isotopic composition (e.g., number of deuterium isotope substitutions) [15]. For isotopomers that only differ in terms of 3D arrangement, they are further classified as enantioisotopomers. In this case, the enantiomers that arise are due to the presence of a stereogenic carbon center that is chiral by virtue of deuterium substitution. Considering that chirality in deuterated enantioisotopomers is induced via the smallest atomistic change possible (i.e., going from hydrogen to deuterium), it is not surprising that inevitable synthetic challenges associated with their synthesis lead to formation of mis‐, under‐ and overdeuterated products. The complexity of isotopic product mixtures from unselective reactions is related to their inseparability using traditional chromatography techniques and complications in their analysis and characterization, especially for enantioisotopomers.

Although hydrogen isotope exchange reactions (HIE) represent powerful techniques to incorporate deuterium, especially in late‐stage settings [33, 34, 35, 36], or even at benzylic positions and allylic positions [37, 38, 39], access to small molecules selectively deuterated at only allylic positions typically require multi‐step synthetic sequences [3, 4, 9, 40, 41]. Notable methods for direct installation of deuterium into allylic positions include an Ene reaction of *N*‐sulfinylsulfonamides in the presence of D_2_O [42], a carbene catalyzed C–H deuteration of enals [43], and a Cu‐catalyzed deuteride transfer to allyl chlorides using 90 bar of D_2_ gas [44]. Despite the challenges associated with making molecules deuterated at an allylic position, significant advances in reductive functionalizations of allenes have enabled the synthesis of deuterated *d_n_*‐isotopomers and *d_1_*_‐3_‐isotopologs. In this regard, the synthesis of a 3°C(sp^3^)–D stereogenic center at an allylic position has also been reported, but only for individual cases (Figure 1c) [45, 46, 47]. Other significant works have been reported by the Ma group where access to terminal allyl‐*d1* compounds is possible from deuterated allene precursors (Figure 1c) [48, 49]. To the best of our knowledge, there are no reports of a concise and high enantiopurity synthesis of an allyl‐*d1* enantioisotopomer [33].

Building on our previous efforts towards achieving precision deuteration in organic molecules [50, 51, 52], and inspired by the success of [Cu–H] catalyzed allene hydrofunctionalizations reported by the Buchwald group [53, 54, 55, 56], we envisioned the development of a copper‐catalyzed semi‐reductive deuteration of deuterated and non‐deuterated allenes. If successful, this strategy could be used to access various isotopomers, isotopologs, and even enantioisotopomers that are deuterated selectively at allylic positions (Figure 1d). However, the strategy is associated with inevitable challenges. For example, nonselective deuteration at any of the three carbon positions within the allene functionality could lead to the generation of isotopomers whereas under‐/over‐deuteration would result in the generation of *d_1_‐d_n_* isotopologs. Moreover, these isotopomers and isotopologs would be inseparable and difficult to analyze, especially considering the complexity associated with the large number of possible isotopic products that can be formed. From a stereochemical standpoint, the assignment of AC and determination of EE in the case of each allylic‐*d1* enantioisotopomer remains unprecedented. In collaboration with the Pate Lab and BrightSpec Inc., we have previously disclosed the first chiral‐tag molecular rotational resonance (MRR) spectroscopy‐based approach that enables determination of EE and assignment of AC for enantioisotopomers containing a benzylic stereogenic center that is chiral by virtue of deuterium substitution [27]. Given this precedent, we hypothesized that a chiral‐tag MRR approach could be developed to analyze an allyl‐*d1* enantioisotopomer for assignment of AC and determination of EE [27, 57, 58].

**FIGURE 2:**
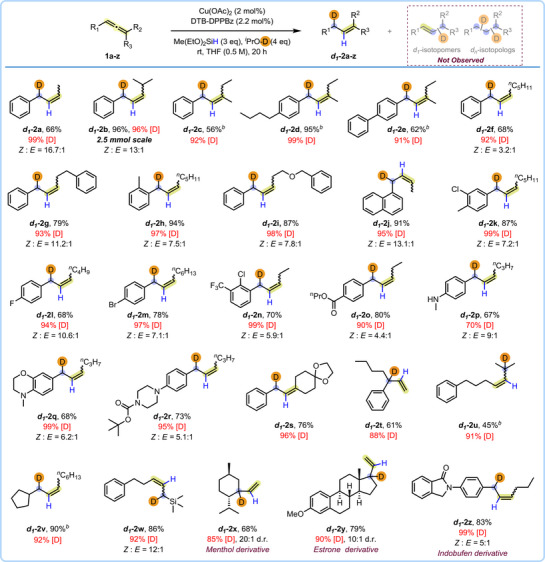
Semi‐reduction of allenes to access *d1*‐isotopomers. [D]‐incorporations are reported based on the ^1^H NMR, ^2^H NMR integrations and in some cases quantitative ^13^C NMR. *^a^* All yields are of isolated product after purification. *^b^* The *Z*/*E* ratio could not be determined. *^n^* Pr = n‐propyl.

Our investigation began by screening various reaction parameters, including catalyst loading, ligand, source of deuterium, etc. on a model substrate **1b** to achieve a highly selective allyl deuterated product (see Supporting Information for full optimization studies). After systematic screening and modifications based on empirical observations, we discovered that using the DTB‐DPPBz ligand (see **L1**, Figure 4 for chemical structure) and Cu(OAc)_2_ catalyst in the presence of diethoxymethylsilane (DEMS, 3 equiv) and *^i^* PrOD (4 equiv), with THF as the solvent, facilitated high regioselectivity, high yield, and near quantitative levels of deuterium incorporation at the allylic position (see Supporting Information for details). This permitted us to turn our attention to making a broad scope of allylic‐*d1* isotopomers (Figure 2). The alkyl components in the aryl, alkyl‐disubstituted internal allenes were evaluated first and revealed that both di‐ and tri‐substituted allenes underwent selective semi‐reductive deuteration (**2a‐2i**, 56%‐96% yield). Notably, this included a gram‐scale synthesis of allyl‐*d1* product **2b**. A naphthalene‐substituted allene was also selectively deuterated leading to allyl‐*d1* product **2j** in 91% yield. Halogen‐substituted arenes such as ‐F, ‐Cl, ‐Br, and ‐CF_3_ substituted arenes all performed well (**2k‐2n**, 68%‐87% yield). Importantly, no deutero‐dehalogenated products were observed. High levels of chemoselectivity were also observed in the isolation of ester‐containing **2o** in 80% yield as no carbonyl reduction occurred. Due to the prevalence of nitrogen and *N*‐heterocycles in FDA approved small molecule drugs [59], we explored various allene substrates that contain nitrogen or a nitrogen heterocycle. An aniline substituted allene performed well (**2p**, 67% yield), albeit with moderate levels of deuterium incorporation likely due to the presence of a mildly acidic proton. Both a morpholine containing allene substrate and Boc‐piperazine containing allene substrate led to good yields of the deuterated products (**2q‐2r**, 68%‐73% yield).

We also examined allenes with different substitution patterns. An aryl, cyclic di‐alkyl allene was effectively converted to the corresponding allyl‐*d1* product (*d1*‐**2s**, 76% yield). A 1,1‐aryl alkyl substituted allene and tri‐alkyl substituted allene were also tested. To our delight, the corresponding 3° deuterated isotopomers—a high‐value isotopomer class (*d1*‐**2t**‐**2u**), were obtained in 45%–61% yield and 88%–91% [D] incorporation. Likewise, product *d1*‐**2v** was obtained from a di‐alkyl substituted allene (90% yield, 92% [D]) in which the deuterium adds to the side containing more highly substituted alkyl functionality with more electron density. An allene substituted with a trimethyl silane was also tested and led to the formation of *d1*‐**2w** in 86% yield. No undesired trimethylsilane (TMS)‐deprotected products were observed. The semi‐reductive deuteration strategy was also evaluated on various natural products and drug‐based *d0*‐allene derivatives. In this regard, allenes derived from menthol and estrone were subjected to the standard conditions and afforded *d1*‐**2x** and *d1*‐**2y** in 68% and 79% yields and 85% and 90% [D] incorporation, respectively. An allene derived from Indobufen also performed well, and afforded the desired product *d1*‐**2z** in good yield (83%) and with 99% [D] incorporation.

**FIGURE 3:**
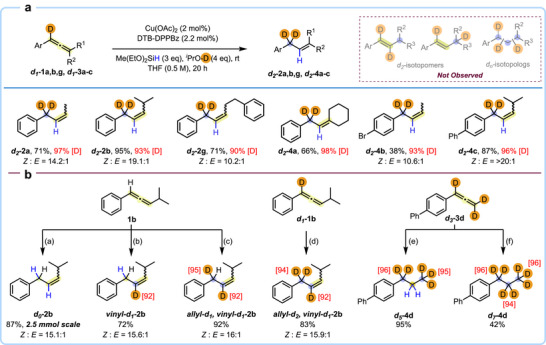
Synthesis of Allyl‐*d_n_* compounds and isotopologs. Reaction conditions: [a] Cu(OAc)_2_ (2 mol%), DTB‐DPPBz (2.2 mol%), Me(EtO)_2_SiH (3 equiv), *^i^* PrOH (4 equiv), THF, rt, 20 h; [b] Cu(OAc)_2_ (2 mol%), DTB‐DPPBz (2.2 mol%), Me(MeO)_2_SiD (3 equiv), *^i^* PrOH (4 equiv), THF, rt, 20 h; [c] Cu(OAc)_2_ (2 mol%), DTB‐DPPBz (2.2 mol%), Me(MeO)_2_SiD (3 equiv), *^i^* PrOD (4 equiv), THF, rt, 20 h; [d] Cu(OAc)_2_ (2 mol%), DTB‐DPPBz (2.2 mol%), Me(MeO)_2_SiD (3 equiv), *^i^* PrOD (4 equiv), THF, rt, 20 h.; [e] Cu(OAc)_2_ (10 mol%), DTB‐DPPBz (11 mol%), Me(MeO)_2_SiH (5 equiv), *^i^* PrOD (6 equiv), THF, 40 °C, 40 h.; [f] Cu(OAc)_2_ (10 mol%), DTB‐DPPBz (11 mol%), Me(MeO)_2_SiD (5 equiv), *^i^* PrOD (6 equiv), THF, 40 °C, 40 h.

After exploring the synthesis of allyl‐*d1* isotopomers, we shifted our attention to expanding the scope and utility of this reaction by expanding access to other *d_n_*‐isotopologs and ‐isotopomers (Figure 3). Given the pharmacological significance of per‐deuterating an allylic position in drug molecules (e.g., deulinoleate ethyl and gildeuretinol acetate are both in clinical trials), we focused our efforts to synthesizing allyl‐*d2* compounds with high levels of selectivity. The successful implementation of this strategy could have major implications for the synthesis of deuterated drug candidates. Using allene‐*d1* substrates as starting materials, a total of six different allyl‐*d2* products, all with excellent levels of D incorporation (at least 90%), were isolated (Figure 3a). Furthermore, the synthesis of various other isotopologs and isotopomers was undertaken. Starting from a non‐deuterated allene (**1b**), we could expand the scope of the reaction to access three other isotopologs—*d0*, vinyl‐*d1*, and an allyl‐*d1*, vinyl‐*d1* in high yields and selectivity (*d0*‐**2b**, vinyl‐*d1*‐2**b**, allyl‐*d1*, vinyl‐*d1*‐**2b**; 72%–92% yield). Similarly, starting from an allene‐*d1* substrate (**1b**), an allyl‐*d2*, vinyl‐*d1*‐**2b** product was synthesized in 83% yield with excellent [D] incorporations. Considering the advancing impact and significance of highly deuterated and perdeuterated alkyl groups, we further expanded the scope of this strategy to enable reductive poly‐deuteration of allene‐*d3* **3d** to afford reductive deuteration products *d5*‐**4d** and *d7*‐**4d**, each bearing a precise and multi‐deuterated *n*‐propyl chain with excellent deuterium incorporations at each deuterated alkyl carbon. Taken together, this study demonstrates that the synthesis of various *d_n_*‐isotopomers and ‐isotopologs can be achieved through our modular protocol.

**FIGURE 4:**
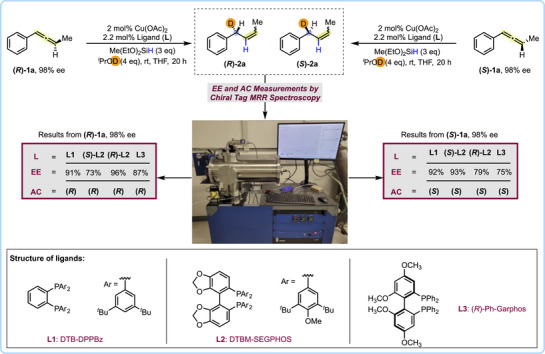
Chirality transfer by CuH‐catalyzed semi‐reductive deuteration of enantioenriched allenes. Absolute configuration and enantiomeric excess of allene substrates were determined from each dominant *Z*‐isomer product. The *Z*/*E* ratio is at least 9.7:1 in all cases. See Supporting Information for the *Z*/*E* ratio for each entry. Upon completion of reaction time, the products were subjected to MRR analysis through direct injections of reaction mixtures.

Next, we moved our attention to synthesize enantioisotopomers with high enantioenrichment by using the semi‐reduction strategy. A promising approach to achieve this utilizes the concept of “memory of chirality”, wherein a chiral substrate transforms to a chiral intermediate, which can further be transformed into a chiral product [60]. We hypothesized that a memory of chirality approach could work if the chiral intermediates formed in the case of allenes do not racemize. Accordingly, both the enantioenriched **(*R)*‐1a** and **(*S)*‐1a** were prepared and independently subjected to the [Cu–H] catalyzed semi‐reductive deuteration protocol. An achiral ligand (DTB‐DPPBz) and three chiral ligands (*S*)‐DTBM‐SEGPHOS, (*R*)‐DTBM‐SEGPHOS and (*R*)‐Ph‐Garphos were each evaluated for their efficacy in promoting a highly stereoselective synthesis of each allyl‐*d1* enantioisotopomer (Figure 4). After analysis by MRR spectroscopy (see below for analysis details), we found that **(*R*)‐2a** is formed from the **(*R*)‐1a** allene and **(*S*)‐2a** is formed from the **(*S*)‐1a** allene. The reaction is highly stereoselective using the DTB‐DPPBz ligand (**L1)** (91% EE and 92% EE for **(*R*)‐2a** and **(*S*)‐2a**, respectively). We observed a matched/mismatched scenario for each DTBM‐SEGPHOS ligand (**L2)**. The matched cases led to the highest EE's for each allyl‐*d1* enantioisotopomer synthesis (96% EE for **(*R*)‐2a** using (*R*)‐DTBM‐SEGPHOS, 93% EE for **(*S*)‐2a** using (*S*)‐DTBM‐SEGPHOS). The (*R*)‐Ph‐Garphos ligand (**L3**) resulted in slightly reduced EE's compared to the matched cases using DTBM‐SEGPHOS (87% EE for **(*R*)‐2a**, 75% EE for **(*S*)‐2a**).

Chiral tag MRR spectroscopy was used to assign the AC and measure the EE of the reaction products in Figure 4 [27, 57, 58]. In this work, a sample of the undeuterated reaction product **2a** was available. The broadband rotational spectrum of complexes formed with 1,1,1‐trifluoropropan‐2‐ol (TFIP) was analyzed by comparing the rotational constants of the two highest intensity spectra to the rotational constants from the equilibrium geometry of the lowest energy isomers identified in a theoretical isomer search and quantum chemistry geometry optimization. The two experimental spectra match the two lowest energy isomers in the theoretical study, as detailed in the Supporting Information.

**FIGURE 5:**
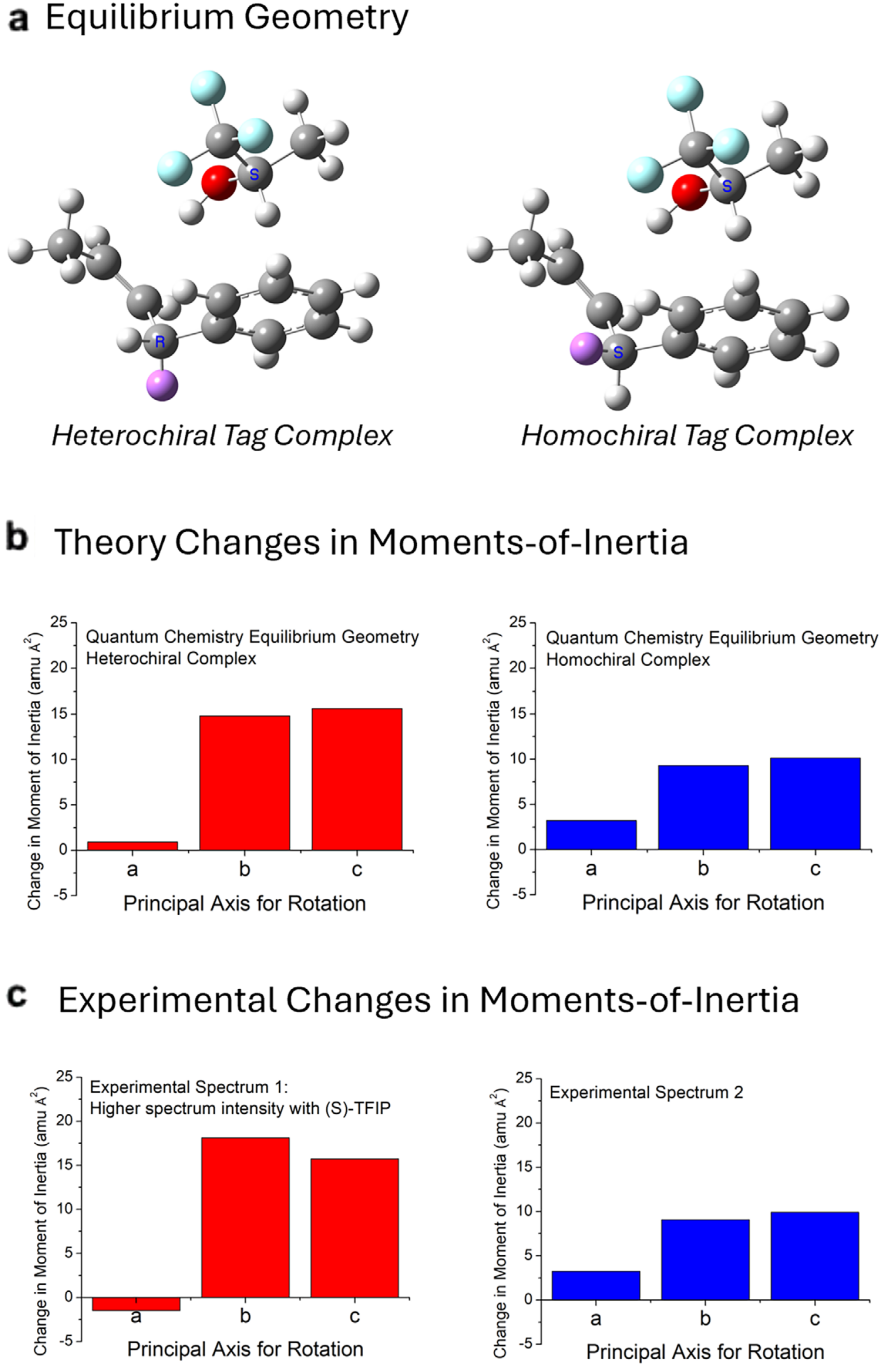
The assignment of the absolute configuration of enantioisotopomer **2a** using chiral tag MRR Spectroscopy. (a) The equilibrium geometries of the heterochiral and homochiral complexes for Isomer 2 of the tag complex between **2a** and TFIP are shown. The magenta shaded hydrogen indicates the site of the deuterium substitution. Rotational spectroscopy measures the moments‐of‐inertia about the three principal axes (by convention, these axes are labeled a, b, and c) via determination of the rotational constants. The changes in the moments‐of‐inertia upon deuteration are used to match the structures in (a) to the experimental spectra as discussed in ref. 58. (b) The changes in the principal moments‐of‐inertia upon deuterium incorporation at either of the prochiral allylic positions are shown using the equilibrium geometry obtained by quantum chemistry (see Supporting Information for computational chemistry details). The *x*‐axis labels identify the principal rotation axis. (c) The experimental changes in the principal moments‐of‐inertia are shown. The assignment of the rotational spectrum to either the homochiral or heterochiral complex is based on agreement with the theory results in (b). For all reaction products using reagent (*R*)‐**1a**, the heterochiral spectrum has higher intensity when (*S*)‐TFIP is used as the tag, therefore, the AC of the reaction products are (*R*)‐**2a**.

The AC of the enantioisotopomer, **(*R*)‐2a** or **(*S*)‐2a**, is assigned by identifying the homochiral and heterochiral complexes of **2a** with TFIP—where a homochiral complex has the same Cahn‐Ingold‐Prelog designation for the chiral center in the analyte and tag [58]. This analysis is illustrated in Figure 5. The AC of each analyte listed in Figure 4 is assigned based on relative intensities of the chiral tag complex spectra when (*S*)‐TFIP (EE = 99.6) is used as the tag. For example, in the analysis of all reaction products produced using **(*R*)‐1a**, the MRR spectrum of the heterochiral complex had higher intensity indicating enantioisotopomer **(*R*)‐2a** is produced in excess.

**FIGURE 6:**
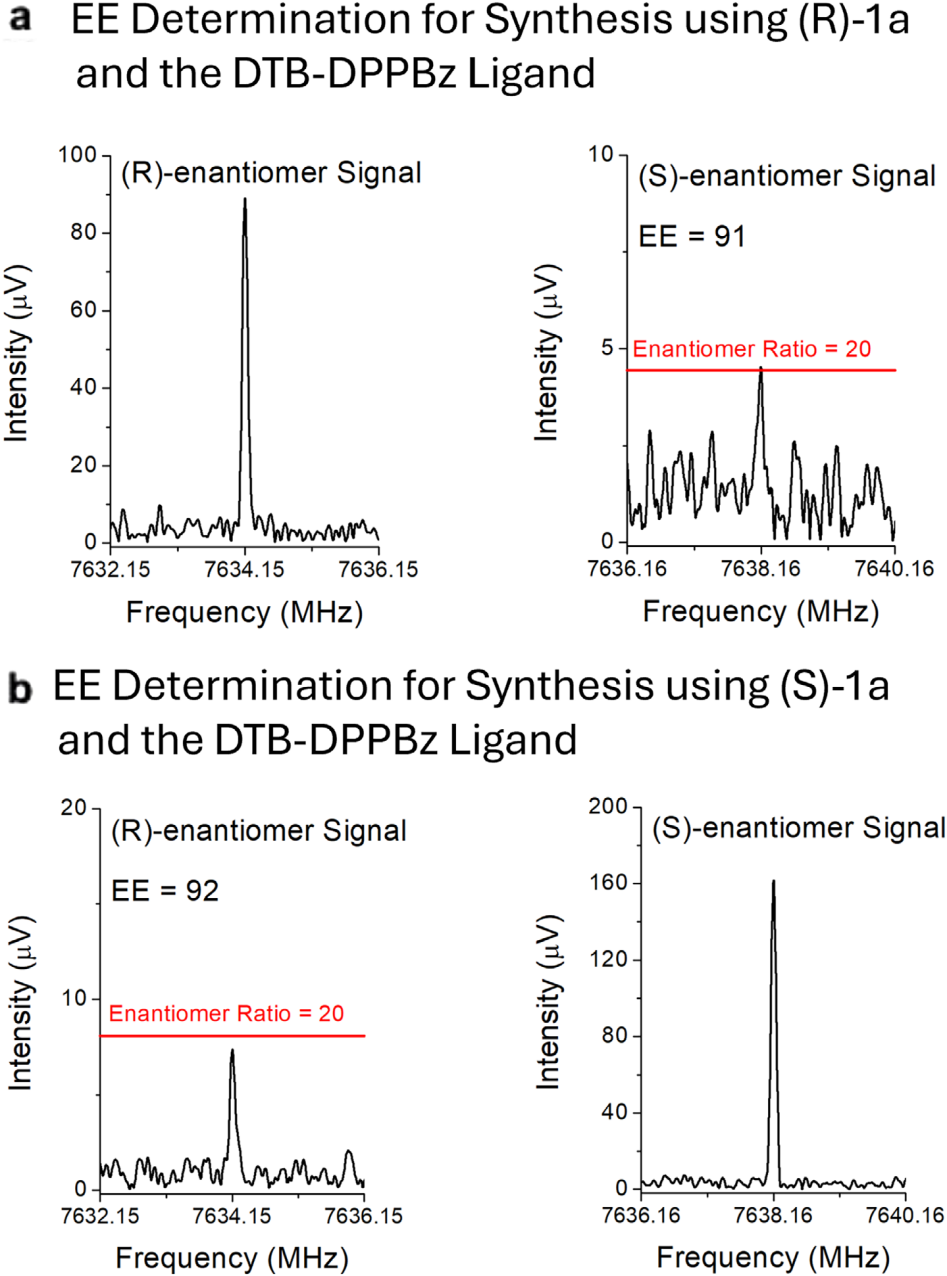
Measurement of the EE using Chiral‐Tag MRR spectroscopy. The IsoMRR measurements of the enantiomeric excess for two reaction products are shown. (a) For the reaction product produced from **(** ***R*** **)**‐**1a** using the achiral DTB‐DPPBz ligand, the heterochiral MRR transition is higher intensity with an approximate ratio of 20:1 relative to the homochiral intensity. **(** ***R*** **)**‐**2a** is produced with EE = 91. (b) For the reaction product using **(** ***S*** **)**‐**1a**, the **(** ***S*** **)**‐**2a** enantiomer is produced with EE = 92.

Measurement of the EE used the BrightSpec IsoMRR instrument, which offers lower sample consumption and shorter measurement times compared to broadband MRR spectrometers [50]. The EE is determined from the ratio of the transition intensity of a pair of homochiral and heterochiral rotational transitions. The instrument response is calibrated in an initial measurement that uses a racemic sample of the TFIP tag. In addition, the experimental intensity ratio—which is approximately the enantiomer ratio—is corrected for the EE of the enantioenriched TFIP tag sample. The results of EE measurements for reaction products produced from **(*R*)‐1a** and **(*S*)‐1a** using the achiral DTB‐DPPBz ligand are shown in Figure 6. Details for the full set of EE measurements can be found in the Supporting Information.

In conclusion, we have described a highly regio‐, chemo‐ and stereoselective semi‐reduction strategy that enables the synthesis of allylic and/or vinyl deuterated compounds. The method allows complete control over the position and degree of deuteration with high precision. The method was applied across a broad substrate scope, including natural products and drugs. Furthermore, various deuterated isotopologs and isotopomers were synthesized with high selectivity and deuterium incorporations. The first high enantiopurity synthesis and unambiguous characterization of an allyl‐*d1* enantioisotopomer was realized using a powerful chiral‐tag MRR analytical spectroscopy technique for EE measurements and determination of AC. Importantly, only 1–2 milligrams of analyte and 15‐min measurement time is required to obtain high resolution spectra for EE determination.

## Conflicts of Interest

The authors declare no conflicts of interest.

## Supporting information




**Supporting File 1**: All data are in the Supporting Information. NMR data is provided in image and peak list format; original NMR spectra files are available from the corresponding author (J.R.C.) upon request. Rotational spectroscopic data and related details can be obtained from the corresponding author (B.H.P.) upon request

## Data Availability

The data that support the findings of this study are available in the supplementary material of this article.
