# Supplementary material for: Semi‐Reduction of Allenes to Access Deuterated Allylic Isotopomers, Isotopologs and Enantioisotopomers
Source: Angew Chem Int Ed Engl. 2026 Feb 12;65(13):e25850. doi: 10.1002/anie.202525850 (PMC13007577; doi:10.1002/anie.202525850)
Supplement: Supplementary file 1 — Supporting File 1: All data are in the Supporting Information. NMR data is provided in image and peak list format; original NMR spectra files are available from the corresponding author (J.R.C.) upon request. Rotational spectroscopic data and related details can be obtained from the corresponding author (B.H.P.) upon request [file ANIE-65-e25850-s001.pdf]

Supplementary Information

Title: Semi-Reduction of Allenes to Access Deuterated Allylic Isotopomers, Isotopologs and Enantioisotopomers

Authors: Lihan Qi,[a] Raviraj Ananda Thorat,[a] Brad D. Maxwell,[b] Jeffery A. Gladding,[b] Aniel J. Rivera Arzola,[a] Shashank P. Sancheti,[a] Reilly E. Sonstrom,[c] Xulin Tang,[a] Isaac J. Anderson,[d] Brooks H. Pate*,[e] Joseph R. Clark*[a]

[a]Department of Chemistry, University of Tennessee, Knoxville, Tennessee 37996, United States

[b]Department of Process Chemistry, Vertex Pharmaceuticals inc., Boston, Massachusetts 02210, United States

[c]BrightSpec Inc., Charlottesville, Virginia 22903, United States

[b]Department of Chemistry, Marquette University, Milwaukee, Wisconsin 53233, United States

[e]Department of Chemistry, University of Virginia, Charlottesville, Virginia 22904, United States

Send correspondence to: Joseph R. Clark jclar198@utk.edu; Brooks H. Pate bp2k@virginia.edu

Table of Contents

I. Synthesis and Characterization of Organic Molecules

A. General information 2
B. Optimization Studies 3
C. Semi-Reductive Deuteration Reaction Scope 6
D. Synthesis of Allyl-d_n Compounds and Isotopologs 23
E. Chirality Transfer by CuH-Catalyzed Semi-Reductive Deuteration of Enantioenriched Allenes 32
F. Synthesis of Allene Starting Materials 34

II. Supporting Information for the Chiral Analysis of (2Z)-2-buten-1-yl-benzene-*d1* by Chiral Tag Molecular Rotational Resonance (MRR) Spectroscopy 62
A. Assigning the Absolute Configuration 64
B. Measurement of the Enantiomeric Excess 76
C. E/Z Isomer Ratio for the Monomer 82
III. References 110
IV. Spectra 114

I. Synthesis and Characterization of Organic Molecules General Information

A. General Information:

All reactions were performed in oven-dried glassware fitted with a rubber septa under a positive pressure of nitrogen, unless otherwise noted. Standard transfer hydrodeuteration reactions were performed in a glass vial sealed with an open top cap fitted with a TFE septum (Chemglass, CG-4908-01/CG-4909-03). Reactions carried out at temperatures above room temperature (25 ˚C) were conducted in a preheated silicon oil bath on top of a Heidolph heating/stir plate. Reactions were monitored by LC-MS using an Agilent 1260 infinity or thin-layer chromatography (TLC) using Silicycle silica gel 60Å F254 pre-coated plates (0.25 mm) and visualized with UV, PMA, and/or $KMnO_4$ stains. Flash chromatography was performed using SiliCycle SiliaFlash® P60, 40-60 µm (230-400 mesh), Biotage® Isolera™ One Flash Chromatography System using prepacked Biotage® Sfär HC/ Sfär HC Duo cartridges (20-200 g), and Orienda® Brix 2802 Flash Chromatography System using SiO_2 cartridges (20-120g). Reverse-phase column chromatography was performed with the aid of Biotage® Isolera™ One Flash Chromatography System using prepacked Biotage® Sfär C18 D cartridges (120 g) and/or manual flash C18 column packed with 50 g Silicycle C18 (Carbon 17% 60 Å, R33230B) with methanol.

Materials: The following chemicals were obtained from commercial vendors and were used as received: $Cu(OAc)_2$ (99.999% from Alfa Aesar and Sigma-Aldrich); 1,2-Bis[bis[3,5-di(t-butyl)phenyl]phosphino]benzene (Wako chemicals), diethoxy(methyl)silane (TCI, Ambeed, Thermo Fisher); 2-propanol-OD (Sigma-Aldrich); ethanol-OD (Acros Organic); 2-propanol (Sigma-Aldrich); *tert*-butanol-OD (Sigma-Aldrich); ammonium fluoride (Acros Organic, Sigma-Aldrich). (*R*)-(+)-3-Butyn-2-ol (99% *ee*), (*S*)-(−)-3-Butyn-2-ol (99% *ee*), boronic acid and boronic acids pinacol esters obtained from commercial vendors through Vertex Pharmaceuticals inc. were used as received. Anhydrous tetrahydrofuran (BHT stabilized) and 1,4-dioxane were obtained from Sigma-Aldrich in a Sure/Seal™ bottle. Anhydrous tetrahydrofuran for transfer hydrodeuteration was obtained from an MBRAUN solvent purification system (MB-SPS). Toluene was redistilled from sodium metal and purged with nitrogen stream. HPLC grade solvents (ethyl acetate, dichloromethane, methanol, hexane, heptane, and acetonitrile) obtained from Sigma-Aldrich were used for purifying the product of transfer hydrodeuteration reactions. Petroleum ether (Certified ACS, 36 – 60 ˚C) was obtained from Fisher scientific. Diethyl ether anhydrous (Certified ACS, BHT stabilized) were obtained from Fisher scientific and Sigma-Aldrich. DMMS-D (dimethoxy(methyl)silane-*d*) was prepared by the literature report method.

Instrumentation: ^{1}H NMR spectra were recorded on a Bruker 400, 500 MHz or Varian 300, 400, 600 MHz spectrometer and are reported in ppm using solvent as an internal standard (residual $CHCl_3$ at 7.26 ppm). Data reported as: s = singlet, d = doublet, t = triplet, q = quartet, p = pentet, sext = sextet, hep = heptet, m = multiplet, br = broad; coupling constant(s) in Hz; integration. ^{13}C NMR spectra were recorded on a Bruker or Varian 101, 126 MHz spectrometer and are reported in ppm using solvent as an internal standard ($CDCl_3$ at 77.16 ppm). ^{19}F NMR spectra were recorded on a Varian 376 MHz spectrometer. ^{2}H NMR spectra were recorded on a Varian 61 MHz spectrometer. Labeled solvent impurities were calculated out when reporting isolated yields. Thermo Scientific Q Exactive Plus Quadrupole Orbitrap hybrid mass spectrometer at University of Tennessee Knoxville was used to obtain the HRMS data for all the samples. All the samples were prepared in methanol or chloroform.

Determination of deuterium incorporation and *Z*/*E* ratio: To calculate deuterium incorporation, the peak that appears in the ^{2}H NMR spectra was correlated to the calculated deuterium incorporation at the allylic/benzylic peak in the ^{1}H NMR spectra. The *Z*/*E* ratios were determined by integration of stereoisomeric protons in the benzylic and/or allylic region of each spectrum for each isolated compound. *J*-coupling measurements combined with the aid of published NMRs for available d_0-isotopologs were used to establish each *Z*/*E* ratio. References are given throughout the SI. Further confirmation that the *Z*-isomer is the dominant product formed was obtained in the analysis of **2a** by MRR. Once the *Z*-isomer was confirmed by MRR, then the *J*-coupling in ^{1}H NMR for the *Z*-isomer could be confirmed for **2a**.

B. Optimization Studies

We initiated reaction discovery using aryl, alkyl-disubstituted internal allene **1b** (Table S1).

Table S1. Reaction Optimization

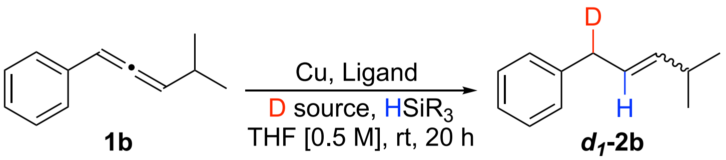

Entry	Cu source	Ligand (Cu:L = 1:1.1)	Silane (eq)	D source (eq)	Yield (%)
1	1 mol% $Cu(OAc)_2$	DTB-DPPBz	DEMS (3)	^{i}PrOD (4)	77
2	2 mol% $Cu(OAc)_2$	DTB-DPPBz	DEMS (2)	^{i}PrOD (3)	69
3	2 mol% $Cu(OAc)_2$	DTB-DPPBz	DEMS (3)	^{i}PrOD (4)	86
4	2 mol% $Cu(OAc)_2$	DTB-DPPBz	DEMS (3)	EtOD (4)	41
5	2 mol% $Cu(OAc)_2$	DTB-DPPBz	DEMS (3)	^{t}BuOD (4)	25
6	2 mol% $Cu(OAc)_2$	(*S*)-DTBM-SEGPHOS	DEMS (3)	^{i}PrOD (4)	82
7	2 mol% $Cu(OAc)_2$	Josiphos	DEMS (3)	^{i}PrOD (4)	unidentified mixture
8	2 mol% $Cu(OAc)_2$	(+)-DIOP	DEMS (3)	^{i}PrOD (4)	trace
9	2 mol% $Cu(OAc)_2$	Walphos	DEMS (3)	^{i}PrOD (4)	95% RSM

rt = room temperature. DEMS = diethoxymethylsilane. RSM = recovered starting material. $Cu(OAc)_2$ was used as stock solution in THF. Yields were determined after purification by flash chromatography.

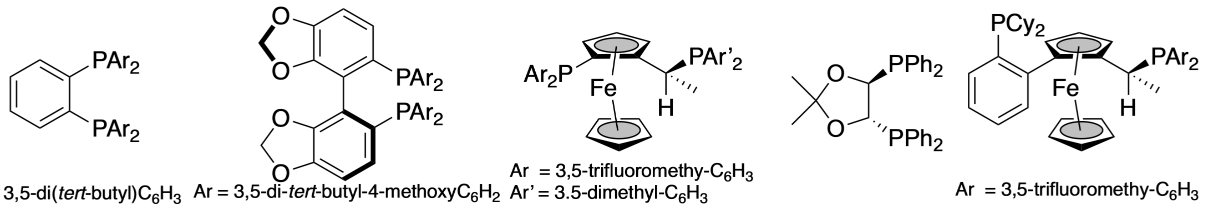

Ar = 3,5-di(*tert*-butyl)C_6H_3 — **L1**: DTB-DPPBz
Ar = 3,5-di-*tert*-butyl-4-methoxyC_6H_2 — **L2**: (*S*)-DTBM-SEGPHOS
Ar = 3,5-trifluoromethy-C_6H_3; Ar' = 3.5-dimethyl-C_6H_3 — **L3**: Josiphos
L4: (+)-DIOP
Ar = 3,5-trifluoromethy-C_6H_3 — **L5**: Walphos

Entry 1, Table S1. In a N_2 filled glovebox, DTB-DPPBz (1.97 mg, 0.00220 mmol, 0.0110 eq.), $Cu(OAc)_2$ (20 *µL* of a 0.100 M solution in THF, 0.00200 mmol, 0.0100 eq.), and THF (0.18 mL) were added to a 2-dram vial equipped with a Teflon® PTE coated stir bar followed by dropwise addition of DEMS (96 µ*L*, 0.600 mmol, 3.00 eq.). A color change from green/blue to yellow was observed while stirring for 5-10 minutes. In a separate 1-dram vial was added (4-methylpenta-1,2-dien-1-yl)benzene **1b** (31.7 mg, 0.200 mmol, 1.00 eq.), THF (0.20 mL), and 2-propanol-*d* (61 µ*L*, 0.800 mmol, 4.00 eq.). The solution in the 1-dram vial was added dropwise over 20 seconds to the 2-dram vial. The total volume of THF was calculated based on having a final reaction concentration of 0.5 M based on the allene substrate. The 2-dram vial was capped, taken out of the glovebox, and stirred for 20 h at room temperature, at which point the reaction mixture was transferred to a 100 mL round bottom flask using 20 mL of diethyl ether. After removing the solvent by rotary evaporation, the product was isolated by flash column chromatography with 250 mL 100%

Hexane as the eluent to give the pure product as colorless oil (**2b**, 24.9 mg, 0.154 mmol, 94% deuterium incorporation, 77% isolated yield).

Entry 2, Table S1. In a N_2 filled glovebox, DTB-DPPBz (3.94 mg, 0.00440 mmol, 0.0220 eq.), $Cu(OAc)_2$ (40 µ*L* of a 0.100 M solution in THF, 0.00400 mmol, 0.0200 eq.), and THF (0.16 mL) were added to a 2-dram vial equipped with a Teflon® PTE coated stir bar followed by dropwise addition of DEMS (64 µ*L*, 0.400 mmol, 2.00 eq.). A color change from green/blue to yellow was observed while stirring for 5-10 minutes. In a separate 1-dram vial was added (4-methylpenta-1,2-dien-1-yl)benzene **1b** (31.7 mg, 0.200 mmol, 1.00 eq.), THF (0.20 mL), and 2-propanol-*d* (46 µ*L*, 0.600 mmol, 3.00 eq.). The solution in the 1-dram vial was added dropwise over 20 seconds to the 2-dram vial. The total volume of THF was calculated based on having a final reaction concentration of 0.5 M based on the allene substrate. The 2-dram vial was capped, taken out of the glovebox, and stirred for 20 h at room temperature, at which point the reaction mixture was transferred to a 100 mL round bottom flask using 20 mL of diethyl ether. After removing the solvent by rotary evaporation, the product was isolated by flash column chromatography with 250 mL 100% Hexane as the eluent to give the pure product as colorless oil (**2b**, 22.4 mg, 0.138 mmol, 93% deuterium incorporation, 69% isolated yield).

Entry 3, Table S1. In a N_2 filled glovebox, DTB-DPPBz (3.94 mg, 0.00440 mmol, 0.0220 eq.), $Cu(OAc)_2$ (40 µ*L* of a 0.100 M solution in THF, 0.00400 mmol, 0.0200 eq.), and THF (0.16 mL) were added to a 2-dram vial equipped with a Teflon® PTE coated stir bar followed by dropwise addition of DEMS (96 µ*L*, 0.600 mmol, 3.00 eq.). A color change from green/blue to yellow was observed while stirring for 5-10 minutes. In a separate 1-dram vial was added (4-methylpenta-1,2-dien-1-yl)benzene **1b** (31.7 mg, 0.200 mmol, 1.00 eq.), THF (0.20 mL), and 2-propanol-*d* (61 µ*L*, 0.800 mmol, 4.00 eq.). The solution in the 1-dram vial was added dropwise over 20 seconds to the 2-dram vial. The total volume of THF was calculated based on having a final reaction concentration of 0.5 M based on the allene substrate. The 2-dram vial was capped, taken out of the glovebox, and stirred for 20 h at room temperature, at which point the reaction mixture was transferred to a 100 mL round bottom flask using 20 mL of diethyl ether. After removing the solvent by rotary evaporation, the product was isolated by flash column chromatography with 250 mL 100% Hexane as the eluent to give the pure product as colorless oil (**2b**, 27.6 mg, 0.171 mmol, 93% deuterium incorporation, 86% isolated yield).

Entry 4, Table S1. In a N_2 filled glovebox, DTB-DPPBz (3.94 mg, 0.00440 mmol, 0.0220 eq.), $Cu(OAc)_2$ (40 µL of a 0.100 M solution in THF, 0.00400 mmol, 0.0200 eq.), and THF (0.16 mL) were added to a 2-dram vial equipped with a Teflon® PTE coated stir bar followed by dropwise addition of DEMS (96 µL, 0.600 mmol, 3.00 eq.). A color change from green/blue to yellow was observed while stirring for 5-10 minutes. In a separate 1-dram vial was added (4-methylpenta-1,2-dien-1-yl)benzene **1b** (31.7 mg, 0.200 mmol, 1.00 eq.), THF (0.20 mL), and ethanol-*d* (47 µL, 0.800 mmol, 4.00 eq.). The solution in the 1-dram vial was added dropwise over 20 seconds to the 2-dram vial. The total volume of THF was calculated based on having a final reaction concentration of 0.5 M based on the allene substrate. The 2-dram vial was capped, taken out of the glovebox, and stirred for 20 h at room temperature, at which point the reaction mixture was transferred to a 100 mL round bottom flask using 20 mL of diethyl ether. After removing the solvent by rotary evaporation, the product was isolated by flash column chromatography with 250 mL 100% Hexane as the eluent to give the pure product as colorless oil (**2b**, 13.3 mg, 0.082 mmol, 99% deuterium incorporation, 41% isolated yield).

Entry 5, Table S1. In a N_2 filled glovebox, DTB-DPPBz (7.88 mg, 0.00880 mmol, 0.0220 eq.), $Cu(OAc)_2$ (80 µL of a 0.100 M solution in THF, 0.00800 mmol, 0.0200 eq.), and THF (0.32 mL) were added to a 2-dram vial equipped with a Teflon® PTE coated stir bar followed by dropwise addition of DEMS (192 µL, 1.200 mmol, 3.00 eq.). A color change from green/blue to yellow was observed while stirring for 5-10 minutes. In a separate 1-dram vial was added (4-methylpenta-1,2-dien-1-yl)benzene **1b** (63.3 mg, 0.400 mmol, 1.00 eq.), THF (0.40 mL), and *tert*-butanol-*d* (153 µL, 1.600 mmol, 4.00 eq.). The solution in the 1-dram vial was added dropwise over 20 seconds to the 2-dram vial. The total volume of THF was calculated based on having a final reaction concentration of 0.5 M based on the allene substrate. The 2-dram vial was capped, taken out of the glovebox, and stirred for 20 h at room temperature, at which point the reaction mixture was transferred to a 100 mL round bottom flask using 20 mL of diethyl ether. After removing the

solvent by rotary evaporation, the product was isolated by flash column chromatography with 450 mL 100% Hexane as the eluent to give the pure product as colorless oil (**2b**, 16.0 mg, 0.100 mmol, 92% deuterium incorporation, 25% isolated yield).

Entry 6, Table S1. In a N_2 filled glovebox, (*S*)-DTBM-SEGPHOS (10.4 mg, 0.00880 mmol, 0.0220 eq.), $Cu(OAc)_2$ (80 µL of a 0.100 M solution in THF, 0.00800 mmol, 0.0200 eq.), and THF (0.32 mL) were added to a 2-dram vial equipped with a Teflon® PTE coated stir bar followed by dropwise addition of DEMS (192 µL, 1.200 mmol, 3.00 eq.). A color change from green/blue to yellow was observed while stirring for 5-10 minutes. In a separate 1-dram vial was added (4-methylpenta-1,2-dien-1-yl)benzene **1b** (63.3 mg, 0.400 mmol, 1.00 eq.), THF (0.40 mL), and 2-propanol-*d* (122 µL, 1.600 mmol, 4.00 eq.). The solution in the 1-dram vial was added dropwise over 20 seconds to the 2-dram vial. The total volume of THF was calculated based on having a final reaction concentration of 0.5 M based on the allene substrate. The 2-dram vial was capped, taken out of the glovebox, and stirred for 20 h at room temperature, at which point the reaction mixture was transferred to a 100 mL round bottom flask using 20 mL diethyl ether. After removing the solvent by rotary evaporation, the product was isolated by flash column chromatography with 450 mL 100% Hexane as the eluent to give the pure product as colorless oil (**2b**, 52.8 mg, 0.328 mmol, 99% deuterium incorporation, 82% isolated yield).

Entry 7, Table S1. In a N_2 filled glovebox, Josiphos (4.00 mg, 0.00440 mmol, 0.0220 eq.), $Cu(OAc)_2$ (40 µL of a 0.100 M solution in THF, 0.00400 mmol, 0.0200 eq.), and THF (0.16 mL) were added to a 2-dram vial equipped with a Teflon® PTE coated stir bar followed by dropwise addition of DEMS (96 µL, 0.600 mmol, 3.00 eq.). A color change from green/blue to yellow was observed while stirring for 5-10 minutes. In a separate 1-dram vial was added (4-methylpenta-1,2-dien-1-yl)benzene **1b** (31.7 mg, 0.200 mmol, 1.00 eq.), THF (0.20 mL), and 2-propanol-*d* (61 µL, 0.800 mmol, 4.00 eq.). The solution in the 1-dram vial was added dropwise over 20 seconds to the 2-dram vial. The total volume of THF was calculated based on having a final reaction concentration of 0.5 M based on the allene substrate. The 2-dram vial was capped, taken out of the glovebox, and stirred for 20 h at room temperature, at which point the reaction mixture was transferred to a 100 mL round bottom flask using 20 mL diethyl ether. After removing the solvent by rotary evaporation, the crude material was analyzed by ^{1}H NMR as unidentified mixture.

Entry 8, Table S1. In a N_2 filled glovebox, (+)-DIOP (3.00 mg, 0.00440 mmol, 0.0220 eq.), $Cu(OAc)_2$ (40 µL of a 0.100 M solution in THF, 0.00400 mmol, 0.0200 eq.), and THF (0.16 mL) were added to a 2-dram vial equipped with a Teflon® PTE coated stir bar followed by dropwise addition of DEMS (96 µL, 0.600 mmol, 3.00 eq.). A color change from green/blue to yellow was observed while stirring for 5-10 minutes. In a separate 1-dram vial was added (4-methylpenta-1,2-dien-1-yl)benzene **1b** (31.7 mg, 0.200 mmol, 1.00 eq.), THF (0.20 mL), and 2-propanol-*d* (61 µL, 0.800 mmol, 4.00 eq.). The solution in the 1-dram vial was added dropwise over 20 seconds to the 2-dram vial. The total volume of THF was calculated based on having a final reaction concentration of 0.5 M based on the allene substrate. The 2-dram vial was capped, taken out of the glovebox, and stirred for 20 h at room temperature, at which point the reaction mixture was transferred to a 100 mL round bottom flask using 20 mL diethyl ether. After removing the solvent by rotary evaporation, the crude material was analyzed by ^{1}H NMR as recovery of starting material and trace amount of product.

Entry 9, Table S1. In a N_2 filled glovebox, Walphos (4.10 mg, 0.00440 mmol, 0.0220 eq.), $Cu(OAc)_2$ (40 µL of a 0.100 M solution in THF, 0.00400 mmol, 0.0200 eq.), and THF (0.16 mL) were added to a 2-dram vial equipped with a Teflon® PTE coated stir bar followed by dropwise addition of DEMS (96 µL, 0.600 mmol, 3.00 eq.). A color change from green/blue to yellow was observed while stirring for 5-10 minutes. In a separate 1-dram vial was added (4-methylpenta-1,2-dien-1-yl)benzene **1b** (31.7 mg, 0.200 mmol, 1.00 eq.), THF (0.20 mL), and 2-propanol-*d* (61 µL, 0.800 mmol, 4.00 eq.). The solution in the 1-dram vial was added dropwise over 20 seconds to the 2-dram vial. The total volume of THF was calculated based on having a final reaction concentration of 0.5 M based on the allene substrate. The 2-dram vial was capped, taken out of the glovebox, and stirred for 20 h at room temperature, at which point the reaction mixture was transferred to a 100 mL round bottom flask using 20 mL diethyl ether. After removing the solvent by rotary evaporation, the crude material was analyzed by ^{1}H NMR as recovery starting material (95% RSM).

C. Semi-Reductive Deuteration Reaction Scope

Figure S1. Semi-Reduction of Allenes to Access *d1*-Isotopomers.

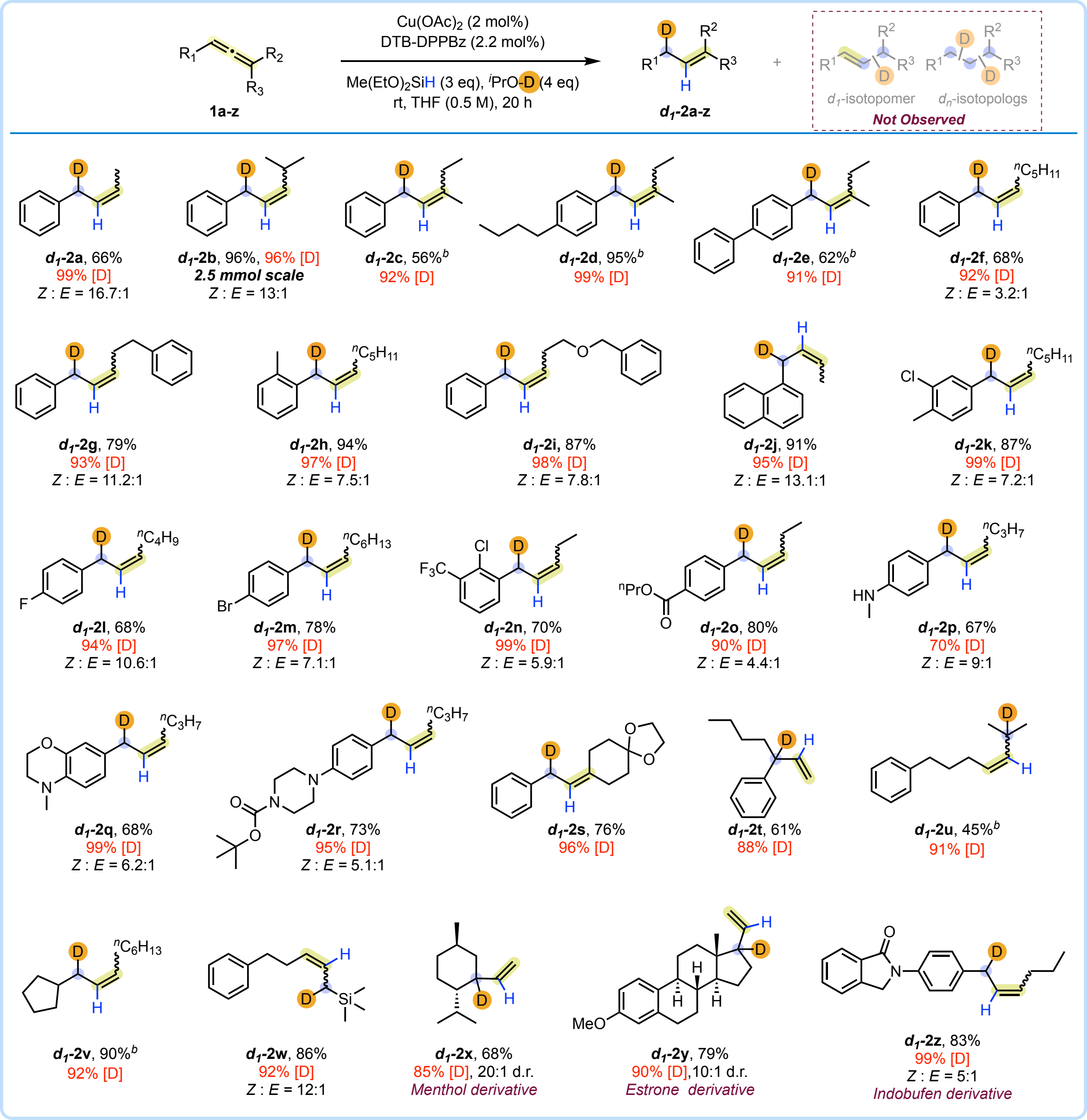

General Procedure for transfer hydrodeuteration reaction in Figure S1

0.2 mmol scale reaction: In a N_2 filled glovebox, ligand (0.0220 eq.), $Cu(OAc)_2$ (20 µL of a 0.100 M solution in THF), and THF (0.16 mL) were added to a 2-dram vial equipped with a Teflon® PTE coated stir bar followed by dropwise addition of diethoxy(methyl)silane (96 µL, 0.600 mmol, 3.00 eq.). A color change from green/blue to yellow was observed while stirring for 5-10 minutes. In a separate 1-dram vial was added the

allene substrate (0.200 mmol, 1.00 eq.), THF (0.20 mL), and 2-propanol-d_1 (61 μL, 0.800 mmol, 4.00 eq.). The solution in the 1-dram vial was added dropwise over 20 seconds to the 2-dram vial. The total volume of THF was calculated based on having a final reaction concentration of 0.5 M based on the allene substrate. The 2-dram vial was capped, taken out of the glovebox, and stirred for the respective time at the appropriate temperature, at which point the reaction mixture was transferred to a 100 mL round bottom flask with 20 mL diethyl ether, after removing the solvent by rotary evaporation, the product was isolated by flash column chromatography. Many of the reported products were isolated as inseparable mixtures of stereoisomers. Unless otherwise noted, only the major stereoisomer is reported in the peak list data below.

(But-2-en-1-yl-1-*d*)benzene [*d*1-2a]

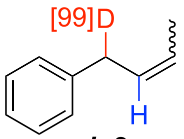

***d*1-2a**

According to the general procedure using 0.5 mmol substrate. DTB-DPPBz (9.85 mg, 0.011 mmol), $Cu(OAc)_2$ (50 μL of a 0.100 M solution in THF), diethoxy(methyl)silane (240 μL, 1.500 mmol, 3.00 eq), and THF (0.45 mL) were combined in a 1-dram vial followed by addition of a solution of buta-1,2-dien-1-ylbenzene (65.1 mg, 0.500 mmol, 1.00 eq.), THF (0.5 mL), and 2-propanol-*d* (153 μL, 2.000 mmol, 4.00 eq.). The 2-dram vial was capped with a pressure relief cap, and the reaction stirred for 20 h at room temperature. Upon completion, the crude product was dry loaded onto silica gel and isolated by flash column chromatography (250 mL of 100% Hexane) to give the pure product as a colorless oil (***d*1-2a**, 44.0 mg, 0.330 mmol, 99% deuterium incorporation, 66% isolated yield, Z/E = 16.7/1).

^{1}H NMR (400 MHz, $CDCl_3$) δ 7.36 – 7.29 (m, 2H), 7.26 – 7.19 (m, 3H), 5.72 – 5.56 (m, 2H), 3.48 – 3.41 (m, 1.01H, Z isomer), 3.39 – 3.32 (m, 0.06H, E isomer) 1.83 – 1.71 (m, 3H).

^{2}H NMR (61 MHz, $CHCl_3$) δ 3.41 (s, 1D).

^{13}C NMR (101 MHz, $CDCl_3$) δ 141.3, 129.1, 128.53, 128.46, 125.9, 125.0, 33.3 (d_0-isotopolog), 32.9 (t, J = 19.0 Hz), 13.0.
Spectroscopic data were consistent with literature reported values.[1]

(4-Methylpent-2-en-1-yl-1-*d*)benzene [*d*1-2b]

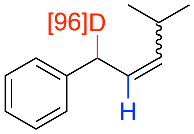

***d*1-2b**

According to the general procedure using 2.5 mmol substrate. DTB-DPPBz (49.2 mg, 0.0550 mmol), $Cu(OAc)_2$ (9.08 mg, 0.0500 mmol), diethoxy(methyl)silane (1.20 mL, 7.500 mmol, 3.00 eq), and THF (1.00 mL) were combined in a 4-dram vial followed by addition of a solution of buta-1,2-dien-1-ylbenzene (395.6 mg, 2.500 mmol, 1.00 eq.), THF (4.0 mL), and 2-propanol-*d* (766 μL, 10.00 mmol, 4.00 eq.). The 4-dram vial was capped with a pressure relief cap, and the reaction stirred for 20 h at room temperature. Upon completion, the crude product was dry loaded onto silica gel and isolated by flash column chromatography (700 mL of 100% Petroleum ether) to give the pure product as a colorless oil (***d*1-2b**, 386.6 mg, 2.40 mmol, 96% deuterium incorporation, 96% isolated yield, Z/E = 13/1).

^{1}H NMR (400 MHz, $CDCl_3$) δ 7.33 – 7.27 (m, 2H), 7.23 – 7.18 (m, 3H), 5.54 – 5.32 (m, 2H), 3.45 – 3.38 (m, 1.04H, Z isomer), 3.36 – 3.30 (m, 0.08H, E isomer), 2.83 – 2.70 (m, 1H), 1.03 (d, J = 6.7 Hz, 6H).

^{2}H NMR (61 MHz, $CHCl_3$) δ 3.42 (s, 1D).

^{13}C NMR (101 MHz, $CDCl_3$) δ 141.4, 138.6, 128.54, 128.46, 126.0, 125.6, 33.7 (d_0-isotopolog), 33.4 (t, J = 19.2 Hz), 26.6, 23.3.

HRMS: (ESI$^+$) m/z [M+H]$^+$ calculated for $C_{12}H_{16}D^+$ 162.1388; found 162.1386

Spectroscopic data were consistent with literature reported d_0-Z-isotopolog.[2]

(3-Methylpent-2-en-1-yl-1-*d*)benzene [*d₁*-2c]

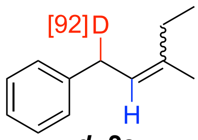

***d₁*-2c**

According to the general procedure, DTB-DPPBz (3.94 mg, 0.00440 mmol), $Cu(OAc)_2$ (20 µL of a 0.200 M solution in THF), diethoxy(methyl)silane (96 µL, 0.600 mmol, 3.00 eq), and THF (0.18 mL) were combined in a 1-dram vial followed by addition of a solution of (3-methylpenta-1,2-dien-1-yl)benzene (31.6 mg, 0.200 mmol, 1.00 eq.), THF (0.20 mL), and 2-propanol-*d* (61 µL, 0.800 mmol, 4.00 eq.). The 2-dram vial was capped with a pressure relief cap, and the reaction stirred for 20 h at room temperature. Upon completion, the crude product was dry loaded onto silica gel and isolated by flash column chromatography (250 mL of 100% Hexane) to give the pure product as a colorless oil (***d₁*-2c**, 18.1 mg, 0.112 mmol, 92% deuterium incorporation, 56% isolated yield, B/A = 2/1). Isomers A and B could not be definitively assigned as either *E* or *Z*.

^{1}H NMR (400 MHz, $CDCl_3$) δ 7.34 – 7.28 (m, 2H), 7.25 – 7.16 (m, 3H), 5.43 – 5.22 (m, 1H), 3.41 – 3.32 (m, 1.08H), 2.22 – 2.13 (m, 0.72H, isomer A), 2.12 – 2.02 (m, 1.49H, isomer B), 1.81 – 1.70 (m, 3H), 1.05 (t, J = 7.4 Hz, 3H).

^{2}H NMR (77 MHz, $CDCl_3$) δ 3.35 (s, 1D).

^{13}C NMR (101 MHz, $CDCl_3$) δ 142.0, 138.3, 138.2, 128.5, 125.83, 125.81, 122.8, 121.7, 33.9 (t, J = 19.0 Hz), 32.5, 24.9, 23.1, 16.2, 12.98, 12.89. Contains a mixture of inseparable *Z* and *E* isomers.

HRMS: (ESI$^+$) m/z [M-H]$^+$ calculated for $C_{12}H_{14}D^+$ 160.1231; found 160.1232

Spectroscopic data were consistent with literature reported *Z*/*E* mixture of the d_0-isotopolog, however authors were also not able to assign which peaks belonged to *E* or *Z* isomer definitively.[3]

1-Butyl-4-(3-methylpent-2-en-1-yl-1-*d*)benzene [*d₁*-2d]

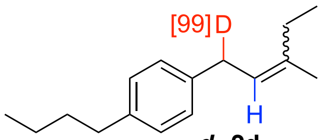

***d*₁-2d**

According to the general procedure, DTB-DPPBz (3.94 mg, 0.00440 mmol), $Cu(OAc)_2$ (20 µL of a 0.200 M solution in THF), diethoxy(methyl)silane (96 µL, 0.600 mmol, 3.00 eq), and THF (0.18 mL) were combined in a 1-dram vial followed by addition of a solution of 1-butyl-4-(3-methylpenta-1,2-dien-1-yl)benzene (42.9 mg, 0.200 mmol, 1.00 eq.), THF (0.20 mL), and 2-propanol-*d* (61 µL, 0.800 mmol, 4.00 eq.). The 2-dram vial was capped with a pressure relief cap, and the reaction stirred for 20 h at room temperature. Upon completion, the crude product was dry loaded onto silica gel and isolated by flash column chromatography (250 mL of 100% Hexane) to give the pure product as a colorless oil (***d_1*-2d**, 41.3 mg, 0.190 mmol, 99% deuterium incorporation, 95% isolated yield, B/A = 3.2/1). Isomers A and B could not be definitively assigned as either *E* or *Z*.

^{1}H NMR (600 MHz, $CDCl_3$) δ 7.21 – 6.96 (m, 4H), 5.41 – 5.22 (m, 1H), 3.37 – 3.28 (m, 1H), 2.59 (t, *J* = 7.8 Hz, 2H), 2.16 (q, *J* = 7.6 Hz, 0.54H, A isomer), 2.06 (q, *J* = 7.5 Hz, 1.73H, B isomer), 1.79 – 1.70 (m, 3H), 1.64 – 1.56 (m, 2H), 1.43 – 1.33 (m, 2H), 1.04 (t, *J* = 7.5 Hz, 3H), 0.94 (t, *J* = 7.3 Hz, 3H).

^{2}H NMR (77 MHz, $CDCl_3$) δ 3.33 (s, 1D).

^{13}C NMR (101 MHz, $CDCl_3$) δ 140.4, 139.1, 138.0, 128.53, 128.51, 128.3, 123.1, 122.0, 35.4, 33.9, 33.6 (t, *J* = 19.1 Hz), 32.5, 25.0, 23.1, 22.6, 16.2, 14.1, 13.0, 12.9. Contains a mixture of inseparable *Z* and *E* isomers.

HRMS: (ESI^+) *m/z* $[M-H]^+$ calculated for $C_{16}H_{22}D^+$ 216.1857; found 216.1858

4-(3-Methylpent-2-en-1-yl-1-*d*)-1,1'-biphenyl [*d_1*-2e]

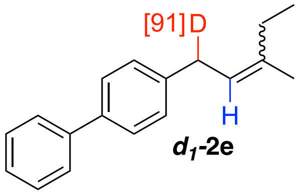

***d*₁-2e**

According to the general procedure, DTB-DPPBz (3.94 mg, 0.00440 mmol), $Cu(OAc)_2$ (20 µL of a 0.200 M solution in THF), diethoxy(methyl)silane (96 µL, 0.600 mmol, 3.00 eq), and THF (0.18 mL) were combined in a 1-dram vial followed by addition of a solution of 4-(3-methylpenta-1,2-dien-1-yl)-1,1'-biphenyl (46.8 mg, 0.200 mmol, 1.00 eq.), THF (0.20 mL), and 2-propanol-*d* (61 µL, 0.800 mmol, 4.00 eq.). The 2-dram vial was capped with a pressure relief cap, and the reaction stirred for 20 h at room temperature. Upon completion, the crude product was dry loaded onto silica gel and isolated by flash column chromatography (250 mL of 100% Hexane) to give the pure product as a colorless oil (***d_1*-2e**, 29.8 mg, 0.125 mmol, 91% deuterium incorporation, 62% isolated yield, B/A = 2.3/1). Isomers A and B could not be definitively assigned as either *E* or *Z*.

^{1}H NMR (500 MHz, $CDCl_3$) δ 7.62 (dd, *J* = 8.4, 1.4 Hz, 2H), 7.59 – 7.52 (m, 2H), 7.49 – 7.43 (m, 2H), 7.39 – 7.32 (m, 1H), 7.32 – 7.28 (m, 2H), 5.44 – 5.39 (m, 0.7 H), 5.37 (d, *J* = 7.2 Hz, 0.3H), 3.46 – 3.40 (m, 1.09H), 2.22 (q, *J* = 7.6 Hz, 0.6H, A isomer), 2.11 (q, *J* = 7.5 Hz, 1.35H, B isomer), 1.80 (t, *J* = 1.4 Hz, 0.8 H), 1.79 (d, *J* = 1.7 Hz, 1.9 H), 1.09 (td, *J* = 7.6, 2.7 Hz, 3H).

^{2}H NMR (77 MHz, $CDCl_3$) δ 3.44 (s, 1D).

^{13}C NMR (126 MHz, CDCl$_3$) δ 141.3, 141.17, 141.14, 141.10, 138.89, 138.87, 128.87, 128.84, 122.7, 121.6, 121.6, 34.0 (d_0-isotopolog), 33.8 (d_0-isotopolog), 33.6 (t, J = 19.4 Hz), 33.4 (t, J = 19.4 Hz), 25.01, 23.09, 16.27, 12.98, 12.90. Contains a mixture of inseparable *Z* and *E* isomers.

HRMS: (ESI$^+$) *m/z* [M-H]$^+$ calculated for $C_{18}H_{18}D^+$ 236.1544; found 236.1541

(Oct-2-en-1-yl-1-*d*)benzene [*d*₁-2f]

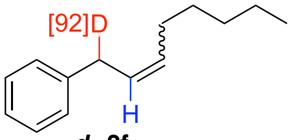

***d*$_1$-2f**

According to the general procedure, DTB-DPPBz (3.94 mg, 0.00440 mmol), $Cu(OAc)_2$ (20 µL of a 0.200 M solution in THF), diethoxy(methyl)silane (96 µL, 0.600 mmol, 3.00 eq), and THF (0.18 mL) were combined in a 1-dram vial followed by addition of a solution of octa-1,2-dien-1-ylbenzene (37.3 mg, 0.200 mmol, 1.00 eq.), THF (0.20 mL), and 2-propanol-*d* (61 µL, 0.800 mmol, 4.00 eq.). The 2-dram vial was capped with a pressure relief cap, and the reaction stirred for 20 h at room temperature. Upon completion, the crude product was dry loaded onto silica gel and isolated by flash column chromatography (250 mL of 100% Hexane) to give the pure product as a colorless oil (***d*$_1$-2f**, 25.6 mg, 0.124 mmol, 92% deuterium incorporation, 68% isolated yield, *Z/E* = 3.2/1).

^{1}H NMR (400 MHz, CDCl$_3$) δ 7.35 – 7.27 (m, 2H), 7.25 – 7.17 (m, 3H), 5.68 – 5.43 (m, 2H), 3.45 – 3.38 (m, 0.84H, *Z* isomer), 3.37 – 3.31 (m, 0.26H, *E* isomer), 2.17 (q, J = 6.9 Hz, 1.48H, *Z* isomer), 2.03 (q, J = 6.0 Hz, 0.48H, *E* isomer), 1.47 – 1.28 (m, 6H), 0.92 (t, J = 5.2 Hz, 3H).

^{2}H NMR (61 MHz, CHCl$_3$) δ 3.46 (s, 1D).

^{13}C NMR (101 MHz, CDCl$_3$) δ 141.4, 132.3, 131.2, 128.5, 128.0, 125.9, 33.6 (d_0-isotopolog), 33.3 (t, J = 19.2 Hz), 31.7, 29.5, 27.4, 22.7, 14.2.

HRMS: (ESI$^+$) *m/z* [M-H]$^+$ calculated for $C_{14}H_{18}D^+$ 188.1544; found 188.1542

Spectroscopic data were consistent with literature reported d_0-isotopologs for the *E* and *Z* isomers.[4]

(Pent-2-ene-1,5-diyl-1-*d*)dibenzene [*d*₁-2g]

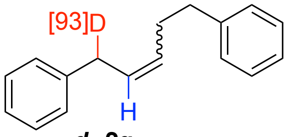

***d*$_1$-2g**

According to the general procedure, DTB-DPPBz (3.94 mg, 0.00440 mmol), $Cu(OAc)_2$ (20 µL of a 0.200 M solution in THF), diethoxy(methyl)silane (96 µL, 0.600 mmol, 3.00 eq), and THF (0.18 mL) were combined in a 1-dram vial followed by addition of a solution of penta-1,2-diene-1,5-diyldibenzene (44.0 mg, 0.200 mmol, 1.00 eq.), THF (0.20 mL), and 2-propanol-*d* (61 µL, 0.800 mmol, 4.00 eq.). The 2-dram vial was capped with a pressure relief cap, and the reaction stirred for 20 h at room temperature. Upon completion, the crude product was dry loaded onto silica gel and isolated by flash column chromatography (250 mL of

100% Hexane) to give the pure product as a colorless oil (***d₁*-2g**, 31.7 mg, 0.142 mmol, 93% deuterium incorporation, 79% isolated yield, *Z/E* = 11.2/1).

^{1}H NMR (500 MHz, $CDCl_3$) δ 7.33 – 7.27 (m, 4H), 7.25 – 7.18 (m, 4H), 7.17 – 7.10 (m, 2H), 5.66 – 5.53 (m, 2H), 3.38 – 3.32 (m, 1.07H), 2.75 (t, *J* = 7.7 Hz, 2H), 2.56 – 2.47 (m, 1.90H, *Z* isomer), 2.42 – 2.36 (m, 0.17H, *E* isomer).

^{2}H NMR (77 MHz, $CHCl_3$) δ 3.38 (0.5D), 3.35 (0.5D).

^{13}C NMR (126 MHz, $CDCl_3$) δ 142.1, 141.1, 129.9, 129.0, 128.7, 128.5, 128.48, 128.47, 126.0, 125.98, 36.1, 33.6 (d_0-isotopolog), 33.3 (t, *J* = 19.6 Hz), 29.4.

HRMS: (ESI$^+$) *m/z* [M-H]$^+$ calculated for $C_{17}H_{16}D^+$ 222.1388; found 222.1386

Spectroscopic data were consistent with literature reported d_0-*Z*-isotopolog.[5]

1-Methyl-2-(oct-2-en-1-yl-1-*d*)benzene [*d₁*-2h]

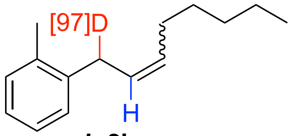

***d₁*-2h**

According to the general procedure, DTB-DPPBz (3.94 mg, 0.00440 mmol), $Cu(OAc)_2$ (20 µL of a 0.200 M solution in THF), diethoxy(methyl)silane (96 µL, 0.600 mmol, 3.00 eq), and THF (0.18 mL) were combined in a 1-dram vial followed by addition of a solution of octa-1,2-dien-1-ylbenzene (37.3 mg, 0.200 mmol, 1.00 eq.), THF (0.20 mL), and 2-propanol-*d* (61 µL, 0.800 mmol, 4.00 eq.). The 2-dram vial was capped with a pressure relief cap, and the reaction stirred for 20 h at room temperature. Upon completion, the crude product was dry loaded onto silica gel and isolated by flash column chromatography (250 mL of 100% Hexane) to give the pure product as a colorless oil (***d₁*-2h**, 38.4 mg, 0.188 mmol, 97% deuterium incorporation, 94% isolated yield, *Z/E* = 7.5/1).

^{1}H NMR (400 MHz, $CDCl_3$) δ 7.19 – 7.08 (m, 4H), 5.58 – 5.42 (m, 2H), 3.39 – 3.27 (m, 1.03H), 2.31 (s, 2.4H), 2.30 (s, 0.3H), 2.16 (dt, *J* = 8.2, 6.5 Hz, 1.65H, *Z* isomer), 2.01 (q, *J* = 7.0 Hz, 0.22H, *E* isomer), 1.42 – 1.23 (m, 6H), 0.91 (t, *J* = 6.9 Hz, 3H).

^{2}H NMR (61 MHz, $CDCl_3$) δ 3.34 (s, 1D).

^{13}C NMR (101 MHz, $CDCl_3$) δ 139.5, 136.3, 132.2, 131.2, 130.2, 128.7, 127.4, 126.1, 31.7, 31.4 (d_0-isotopolog), 31.1 (t, *J* = 18.6 Hz), 29.5, 27.5, 22.7, 19.6, 14.2.

HRMS: (ESI$^+$) *m/z* [M+H]$^+$ calculated for $C_{15}H_{22}D^+$ 204.1857; found 204.1856

(5-(Benzyloxy)pent-2-en-1-yl-1-*d*)benzene [*d₁*-2i]

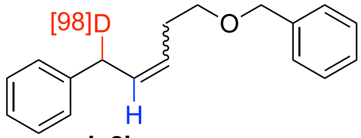

***d₁*-2i**

According to the general procedure, DTB-DPPBz (3.94 mg, 0.00440 mmol), $Cu(OAc)_2$ (20 µL of a 0.200 M solution in THF), diethoxy(methyl)silane (96 µL, 0.600 mmol, 3.00 eq), and THF (0.18 mL) were combined in a 1-dram vial followed by addition of a solution of (5-(benzyloxy)penta-1,2-dien-1-yl)benzene (50.0 mg, 0.200 mmol, 1.00 eq.), THF (0.20 mL), and 2-propanol-*d* (61 µL, 0.800 mmol, 4.00 eq.). The 2-dram vial was capped with a pressure relief cap, and the reaction stirred for 20 h at room temperature. Upon completion, the crude product was dry loaded onto silica gel and isolated by flash column chromatography (250 mL of 100% Hexane) to give the pure product as a colorless oil (***d1*-2i**, 44.2 mg, 0.174 mmol, 98% deuterium incorporation, 87% isolated yield, *Z/E* = 7.8/1).

^{1}H NMR (500 MHz, $CDCl_3$) δ 7.31 – 7.26 (m, 4H), 7.24 – 7.16 (m, 3H), 7.14 – 7.08 (m, 3H), 5.64 – 5.56 (m, 1H), 5.53 – 5.43 (m, 1H), 4.47 (s, 1.7H), 4.45 (s, 0.2H), 3.47 (t, *J* = 6.9 Hz, 1.84H), 3.45 – 3.43 (m, 0.17H), 3.37 – 3.31 (m, 1.02H, *Z* isomer), 3.29 – 3.24 (m, 0.13H, *E* isomer), 2.47 – 2.39 (m, 1.8H), 2.33 – 2.26 (m, 0.23H).

^{2}H NMR (77 MHz, $CHCl_3$) δ 3.43 (s, 1D).

^{13}C NMR (126 MHz, CDCl3) δ 141.0, 138.7, 130.3, 130.2, 128.6, 128.5, 127.8, 127.7, 126.8, 126.0, 73.1, 70.0, 33.8 (d_0-isotopolog), 33.4 (t, *J* = 19.4 Hz), 28.2.

HRMS: (ESI$^+$) *m/z* $[M+H]^+$ calculated for $C_{18}H_{20}DO^+$ 254.1650; found 254.1648

Spectroscopic data were consistent with literature reported d_0-isotopolog as *E/Z* mixture.[6]

1-(But-2-en-1-yl-1-*d*)naphthalene [*d₁*-2j]

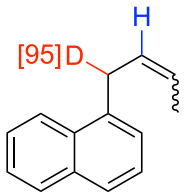

***d*$_1$-2j**

According to the general procedure, DTB-DPPBz (3.94 mg, 0.00440 mmol), $Cu(OAc)_2$ (20 µL of a 0.200 M solution in THF), diethoxy(methyl)silane (96 µL, 0.600 mmol, 3.00 eq), and THF (0.18 mL) were combined in a 1-dram vial followed by addition of a solution of 1-(buta-1,2-dien-1-yl)naphthalene (36.1 mg, 0.200 mmol, 1.00 eq.), THF (0.20 mL), and 2-propanol-*d* (61µL, 0.800 mmol, 4.00 eq.). The 2-dram vial was capped with a pressure relief cap, and the reaction stirred for 20 h at room temperature. Upon completion, the crude product was dry loaded onto silica gel and isolated by flash column chromatography (200 mL of 100% Petroleum Ether) to give the pure product as a pale yellow oil (***d*$_1$-2j**, 25.6 mg, 0.182 mmol, 95% deuterium incorporation, 91% isolated yield, *Z/E* = 13.1/1).

^{1}H NMR (400 MHz, $CDCl_3$) δ 8.05 (d, *J* = 7.5 Hz, 1H), 7.88 (d, *J* = 6.7 Hz, 1H), 7.75 (d, *J* = 8.0 Hz, 1H), 7.59 – 7.32 (m, 4H), 5.75 – 5.60 (m, 2H), 3.90 – 3.81 (m, 1.05H, *Z* isomer), 3.80 – 3.75 (m, 0.08H, *E* isomer)., 1.87 – 1.78 (m, 3H).

^{2}H NMR (61 MHz, $CHCl_3$) δ 3.85 (s, 1D).

^{13}C NMR (101 MHz, CDCl$_3$) δ 137.3, 134.0, 132.2, 128.9, 128.8, 126.8, 125.9, 125.81, 125.78, 125.6, 125.2, 124.1, 30.8 (d$_0$-isotopolog), 30.5 (t, J = 19.7 Hz), 13.2.

HRMS: (ESI$^+$) *m/z* [M-H]$^+$ calculated for $C_{14}H_{12}D^+$ 182.1075; found 182.1073

Spectroscopic data were consistent with literature reported *d*$_0$-isotopolog for the *Z* isomer.[7]

2-Chloro-1-methyl-4-(oct-2-en-1-yl-1-*d*)benzene [*d₁*-2k]

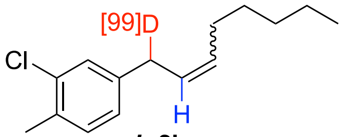

***d*$_1$-2k**

According to the general procedure, DTB-DPPBz (3.94 mg, 0.00440 mmol), Cu(OAc)$_2$ (20 µL of a 0.200 M solution in THF), diethoxy(methyl)silane (96 µL, 0.600 mmol, 3.00 eq), and THF (0.18 mL) were combined in a 1-dram vial followed by addition of a solution of 2-chloro-1-methyl-4-(octa-1,2-dien-1-yl)benzene (46.9 mg, 0.200 mmol, 1.00 eq.), THF (0.20 mL), and 2-propanol-*d* (61 µL, 0.800 mmol, 4.00 eq.). The 2-dram vial was capped with a pressure relief cap, and the reaction stirred for 20 h at room temperature. Upon completion, the crude product was dry loaded onto silica gel and isolated by flash column chromatography (150 mL of 100% Petroleum Ether) to give the pure product as a colorless oil (***d*$_1$-2k**, 41.3 mg, 0.174 mmol, 99% deuterium incorporation, 87% isolated yield, *Z/E* = 7.2/1).

^{1}H NMR (400 MHz, CDCl$_3$) δ 7.17 (s, 1H), 7.13 (d, J = 7.7 Hz, 1H), 6.98 (d, J = 7.8 Hz, 1H), 5.59 – 5.45 (m, 2H), 3.38 – 3.29 (m, 1.01H, *Z* isomer), 3.28 – 3.23 (m, 0.14H, *E* isomer), 2.34 (s, 3H), 2.13 (q, J = 6.8 Hz, 2H), 1.47 – 1.23 (m, 6H), 0.91 (t, J = 6.8 Hz, 3H).

^{2}H NMR (61 MHz, CHCl$_3$) δ 3.37 (s, 1D).

^{13}C NMR (101 MHz, CDCl$_3$) δ 140.6, 134.3, 133.4, 131.7, 131.0, 129.0, 127.4, 126.7, 32.8 (d$_0$-isotopolog), 32.5 (t, J = 19.4 Hz), 31.7, 29.5, 27.4, 22.7, 19.7, 14.2.

HRMS: (ESI$^+$) *m/z* [M]$^+$ calculated for $C_{15}H_{20}DCl^+$ 237.1389; found 237.1391

1-fluoro-4-(hept-2-en-1-yl-1-*d*)benzene [*d₁*-2l]

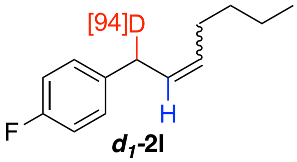

***d*$_1$-2l**

According to the general procedure, DTB-DPPBz (3.94 mg, 0.00440 mmol), Cu(OAc)$_2$ (20 µL of a 0.200 M solution in THF), diethoxy(methyl)silane (96 µL, 0.600 mmol, 3.00 eq), and THF (0.18 mL) were combined in a 1-dram vial followed by addition of a solution of 1-fluoro-4-(hepta-1,2-dien-1-yl)benzene (38.1 mg, 0.200 mmol, 1.00 eq.), THF (0.20 mL), and 2-propanol-*d* (61 µL, 0.800 mmol, 4.00 eq.). The 2-dram vial was capped with a pressure relief cap, and the reaction stirred for 20 h at room temperature. Upon completion, the crude product was dry loaded onto silica gel and isolated by flash column chromatography (250 mL of

100% Hexane) to give the pure product as a pale yellow oil (***d₁*-2l**, 26.3 mg, 0.1360 mmol, 94% deuterium incorporation, 68% isolated yield, *Z/E* = 10.6/1).

^{1}H NMR (400 MHz, $CDCl_3$) δ 7.14 (td, *J* = 5.4, 1.9 Hz, 1H), 7.01 – 6.92 (m, 2H), 5.58 – 5.45 (m, 2H), 3.41 – 3.30 (m, 1.06H, *Z* isomer), 3.30 – 3.25 (m, 0.1H, *E* isomer), 2.20 – 2.09 (m, 2.01H), 2.06 – 1.99 (m, 0.27H), 1.42 – 1.28 (m, 4H), 0.92 (t, *J* = 7.1 Hz, 3H)

^{2}H NMR (61 MHz, $CHCl_3$) δ 3.38 (s, 1D).

^{13}C NMR (101 MHz, $CDCl_3$) δ 161.4 (d, *J* = 243.0 Hz), 136.9, 130.6 (d, *J* = 154.9 Hz), 128.8 (d, *J* = 184.3 Hz), 115.3, 115.1, 32.5 (t, *J* = 19.2 Hz), 32.0, 27.1, 22.5, 14.1.

^{19}F NMR (376 MHz, $CDCl_3$) δ -117.86 – 117.95 (m).

HRMS: (ESI^{+}) *m/z* [M+H]$^{+}$ calculated for $C_{13}H_{17}DF^{+}$ 194.1450; found 194.1450

Spectroscopic data were consistent with literature reported d_0-*E*-isotopolog.[8]

1-bromo-4-(non-2-en-1-yl-1-*d*)benzene [*d₁*-2m]

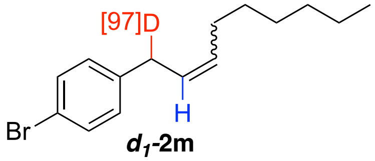

***d₁*-2m**

According to the general procedure, DTB-DPPBz (3.94 mg, 0.00440 mmol), $Cu(OAc)_2$ (20 µL of a 0.200 M solution in THF), diethoxy(methyl)silane (96 µL, 0.600 mmol, 3.00 eq), and THF (0.18 mL) were combined in a 1-dram vial followed by addition of a solution of 1-bromo-4-(nona-1,2-dien-1-yl)benzene (55.8 mg, 0.200 mmol, 1.00 eq.), THF (0.20 mL), and 2-propanol-*d* (61 µL, 0.800 mmol, 4.00 eq.). The 2-dram vial was capped with a pressure relief cap, and the reaction stirred for 20 h at room temperature. Upon completion, the crude product was dry loaded onto silica gel and isolated by flash column chromatography (250 mL of 100% Hexane) to give the pure product as a pale brown oil (***d₁*-2m**, 44.0 mg, 0.156 mmol, 97% deuterium incorporation, *Z/E* = 7.1/1).

^{1}H NMR (500 MHz, $CDCl_3$) δ 7.40 (d, *J* = 8.4 Hz, 2H), 7.07 (d, *J* = 8.3 Hz, 2H), 5.59 – 5.45 (m, 2H), 3.37 – 3.29 (m, 1.07H, *Z* isomer), 3.29 – 3.24 (m, 0.15H, *E* isomer), 2.18 – 2.08 (m, 2H), 1.44 – 1.35 (m, 2H), 1.35 – 1.28 (m, 6H), 0.90 (t, 3H).

^{2}H NMR (77 MHz, $CDCl_3$) δ 3.33 (s, 1D).

^{13}C NMR (126 MHz, $CDCl_3$) δ 140.2, 131.6, 131.4, 130.1, 127.2, 119.6, 32.9 (d_0-isotopolog), 32.6 (t, *J* = 19.7 Hz), 31.8, 29.6, 29.0, 27.3, 22.7, 14.1.

HRMS: (ESI^{+}) *m/z* [M-H]$^{+}$ calculated for $C_{15}H_{19}DBr^{+}$ 280.0806; found 280.0802

Note: The purification method for this product was not efficient to remove all the trace 1-octyne impurities from the allene synthesis step. This impurity was accounted for in calculating the yield.

2-Chloro-1-(pent-2-en-1-yl-1-*d*)-3-(trifluoromethyl)benzene [*d*$_1$-2n]

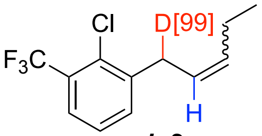

***d*$_1$-2n**

According to the general procedure, DTB-DPPBz (3.94 mg, 0.00440 mmol), $Cu(OAc)_2$ (20 µL of a 0.200 M solution in THF), diethoxy(methyl)silane (96 µL, 0.600 mmol, 3.00 eq), and THF (0.18 mL) were combined in a 1-dram vial followed by addition of a solution of 1-(buta-1,2-dien-1-yl)-2-chloro-3-(trifluoromethyl)benzene (49.3 mg, 0.200 mmol, 1.00 eq.), THF (0.20 mL), and 2-propanol-*d* (61 µL, 0.800 mmol, 4.00 eq.). The 2-dram vial was capped with a pressure relief cap, and the reaction stirred for 20 h at room temperature. Upon completion, the crude product was dry loaded onto silica gel and isolated by flash column chromatography (100 mL of 100% Petroleum Ether) to give the pure product as a colorless oil (***d*$_1$-2n**, 38.8 mg, 0.140 mmol, 99% deuterium incorporation, 70% isolated yield, *Z/E* = 5.9/1).

^{1}H NMR (400 MHz, $CDCl_3$) δ 7.56 (dd, *J* = 7.7, 1.8 Hz, 1H), 7.44 (d, *J* = 7.7 Hz, 1H), 7.28 (dd, *J* = 14.2, 6.4 Hz, 1H), 5.66 – 5.52 (m, 1H), 5.47 (t, *J* = 9.1 Hz, 1H), 3.59 – 3.51 (m, 1.01H, *Z* isomer), 3.51 – 3.46 (m, 0.17H, *E* isomer), 2.22 – 2.00 (m, 2H), 1.02 (t, *J* = 7.6 Hz, 3H).

^{2}H NMR (61 MHz, $CHCl_3$) δ 3.53 (s, 1D).

^{13}C NMR (101 MHz, $CDCl_3$) δ 141.3, 135.2, 134.3, 133.6, 133.3, 126.4, 125.4 (q, *J* = 5.5 Hz), 123.1 (q, *J* = 273.2 Hz), 31.2 (d_0-isotopolog), 30.9 (t, *J* = 20.1 Hz), 25.6, 20.7, 14.1, 13.6. Contains a mixture of inseparable *E* and *Z* isomers.

^{19}F NMR (376 MHz, $CDCl_3$) δ -62.46

HRMS: (ESI$^+$) *m/z* $[M+H]^+$ calculated for $C_{12}H_{12}DClF_3^+$ 250.0715; found 250.0715

Propyl 4-(but-2-en-1-yl-1-*d*)benzoate [*d*$_1$-2o]

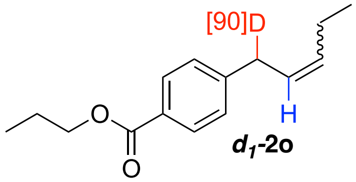

***d*$_1$-2o**

According to the general procedure, DTB-DPPBz (3.94 mg, 0.00440 mmol), $Cu(OAc)_2$ (20 µL of a 0.200 M solution in THF), diethoxy(methyl)silane (96 µL, 0.600 mmol, 3.00 eq), and THF (0.18 mL) were combined in a 1-dram vial followed by addition of a solution of propyl 4-(but-2-en-1-yl-1-*d*)benzoate (43.3 mg, 0.200 mmol, 1.00 eq.), THF (0.20 mL), and 2-propanol-*d* (61 µL, 0.800 mmol, 4.00 eq.). The 2-dram vial was capped with a pressure relief cap, and the reaction stirred for 20 h at room temperature. Upon completion, the crude product was dry loaded onto silica gel and isolated by flash column chromatography (150 mL of 5% diethyl ether in Petroleum Ether) to give the pure product as a colorless oil (***d*$_1$-2o,** 35.2 mg, 0.160 mmol, 90% deuterium incorporation, 80% isolated yield, *Z/E* = 4.4/1).

^{1}H NMR (400 MHz, $CDCl_3$) δ 7.96 (d, *J* = 8.3 Hz, 2H), 7.25 (d, *J* = 8.3 Hz, 2H), 5.63 – 5.45 (m, 2H), 4.26 (t, *J* = 6.7 Hz, 2H), 3.47 – 3.39 (m, 0.9H, *Z* isomer), 3.39 – 3.33 (m, 0.2H, *E* isomer), 2.22 – 1.99 (m, 2H), 2.23

– 2.10 (m, 1.67H, *Z* isomer), 2.09 – 2.00 (m, 0.38H, *E* isomer), 1.02 (t, *J* = 7.3 Hz, 3H), 1.00 (t, *J* = 7.6 Hz, 3H)

^{2}H NMR (61 MHz, $CHCl_3$) δ 3.43 (s, 1D).

^{13}C NMR (101 MHz, $CDCl_3$) δ 166.9, 146.7, 134.5, 133.5, 129.9, 128.4, 126.5, 66.5, 33.6 (d_0-isotopolog), 33.2 (t, *J* = 19.7 Hz), 22.3, 20.8, 14.4, 10.7.

HRMS: (ESI$^+$) *m/z* [M+H]$^+$ calculated for $C_{15}H_{20}DO_2^+$ 234.1599, found 234.1595

4-(Hex-2-en-1-yl-1-*d*)-*N*-methylaniline [*d₁*-2p]

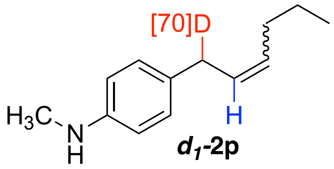

***d₁*-2p**

According to the general procedure, DTB-DPPBz (9.85 mg, 0.0110 mmol), $Cu(OAc)_2$ (100 µL of a 0.100 M solution in THF), diethoxy(methyl)silane (240 µL, 1.500 mmol, 2.00 eq), and THF (0.4 mL) were combined in a 1-dram vial followed by addition of a solution of 4-(hexa-1,2-dien-1-yl)-*N*-methylaniline (93.6 mg, 0.500 mmol, 1.00 eq.), THF (0.50 mL), and 2-propanol-*d* (153 µL, 2.00 mmol, 4.00 eq.). The 2-dram vial was capped with a pressure relief cap, and the reaction stirred for 20 h at room temperature. Upon completion, the reaction was quenched with sat. NH_4F solution in methanol. The result solution was further stirred at room temperature for 1h, then the mixture was transferred to a 100 mL round bottom flask with methanol, after removing the solvent by rotary evaporation, the crude product was dry loaded onto silica gel and isolated by Biotage® Isolera™ One Flash Chromatography System using prepacked Biotage® Sfär HC Duo cartridges 25g (12 CV 0→100% ethyl acetate in hexane) to give the pure product as a yellow oil (***d₁*-2p,** 64.0 mg, 0.335 mmol, 70% deuterium incorporation, 67% isolated yield, *Z/E* = 9/1).

^{1}H NMR (400 MHz, $CDCl_3$) δ 7.03 (d, *J* = 8.4, 2H), 6.57 (d, *J* = 8.5 Hz, 2H), 5.61 – 5.43 (m, 2H), 3.58 (br s, 1H), 3.34 – 3.25 (m, 1.30 H, *Z* isomer), 3.25 – 3.20 (m, 0.14H, *E* isomer), 2.83 (s, 3H), 2.14 (q, *J* = 7.2 Hz, 1.71H, *Z* isomer), 2.00 (q, *J* = 7.0 Hz, 0.19H, *E* isomer), 1.43 (hept, *J* = 7.3 Hz, 2H), 0.95 (t, *J* = 7.4 Hz, 3H).

^{2}H NMR (61 MHz, $CHCl_3$) δ 3.30 (s, 1D).

^{13}C NMR (126 MHz, $CDCl_3$) δ 130.0, 129.1, 129.0, 128.2, 125.3, 112.7, 32.6, 32.2 (t, *J* = 19.6 Hz), 29.3, 22.9, 13.9.

HRMS: (ESI$^+$) *m/z* [M+H]$^+$ calculated for $C_{13}H_{19}DN^+$ 191.1653; found 191.1655

7-(Hex-2-en-1-yl-1-*d*)-4-methyl-3,4-dihydro-2*H*-benzo[*b*][1,4]oxazine [*d₁*-2q]

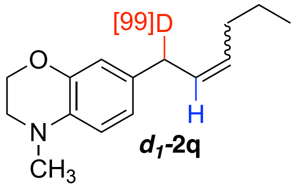

***d₁*-2q**

According to the general procedure, DTB-DPPBz (9.85 mg, 0.0110 mmol), $Cu(OAc)_2$ (100 µL of a 0.100 M solution in THF), diethoxy(methyl)silane (240 µL, 1.500 mmol, 2.00 eq), and THF (0.17 mL) were combined in a 1-dram vial followed by addition of a solution of 7-(hexa-1,2-dien-1-yl)-4-methyl-3,4-dihydro-2*H*-benzo[*b*][1,4]oxazine solution in THF (114.6 mg in 0.23 mL THF, 0.500 mmol, 1.00 eq.), THF (0.50 mL), and 2-propanol-*d* (153 µL, 2.00 mmol, 4.00 eq.). The 2-dram vial was capped with a pressure relief cap, and the reaction stirred for 20 h at room temperature. Upon completion, the reaction was quenched with sat. NH_4F solution in methanol. The result solution was further stirred at room temperature for 1h, then the mixture was transferred to a 100 mL round bottom flask with methanol, after removing the solvent by rotary evaporation, the crude product was dry loaded onto silica gel and isolated by Biotage® Isolera™ One Flash Chromatography System using prepacked Biotage® Sfär HC Duo cartridges 25g (12 CV 0→100% ethyl acetate in hexane) to give the pure product as a pale yellow oil (***d₁*-2q,** 79.5 mg, 0.340 mmol, 99% deuterium incorporation, 68% isolated yield, *Z/E* = 6.2/1).

^{1}H NMR (400 MHz, $CDCl_3$) δ 6.70 – 6.59 (m, 3H), 5.58 – 5.42 (m, 2H), 4.34 – 4.25 (m, 2H), 3.30 – 3.17 (m, 3H), 2.85 (s, 3H), 2.19 – 2.06 (m, 1.87H, *Z* isomer), 2.03 – 1.96 (m, 0.3H, *E* isomer), 1.49 – 1.34 (m, 2H), 0.94 (t, *J* = 7.4 Hz, 3H).

^{2}H NMR (61 MHz, $CHCl_3$) δ 3.26 (s, 1D).

^{13}C NMR (101 MHz, $CDCl_3$) δ 144.5, 134.8, 131.6, 130.4, 128.8, 121.1, 116.0, 113.0, 65.2, 49.5, 39.2, 32.7 (d_0-isotopolog), 32.4 (t, *J* = 19.7 Hz), 29.4, 23.1, 14.1.

HRMS: (ESI^+) *m/z* $[M+H]^+$ calculated for $C_{15}H_{21}DNO^+$ 233.1759; found 233.1758

tert-butyl 4-(4-(hex-2-en-1-yl-1-_d_)phenyl)piperazine-1-carboxylate [d_1-2r]

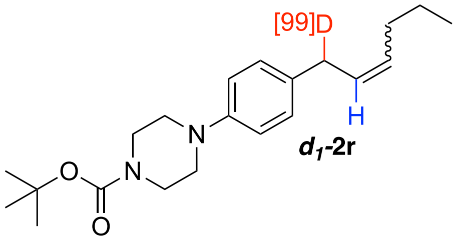

***d₁*-2r**

According to the general procedure, DTB-DPPBz (9.85 mg, 0.0110 mmol), $Cu(OAc)_2$ (100 µL of a 0.100 M solution in THF), diethoxy(methyl)silane (240 µL, 1.500 mmol, 2.00 eq), and THF (0.17 mL) were combined in a 1-dram vial followed by addition of a solution of *tert*-butyl 4-(4-(hexa-1,2-dien-1-yl)phenyl)piperazine-1-carboxylate (171.2 mg, 0.500 mmol, 1.00 eq.), THF (0.50 mL), and 2-propanol-*d* (153 µL, 2.00 mmol, 4.00 eq.). The 2-dram vial was capped with a pressure relief cap, and the reaction stirred for 20 h at room temperature. Upon completion, the reaction was quenched with sat. NH_4F solution in methanol. The result solution was further stirred at room temperature for 1h, then the mixture was transferred to a 100 mL round bottom flask with methanol, after removing the solvent by rotary evaporation, the crude product was dry loaded onto silica gel and isolated by Biotage® Isolera™ One Flash Chromatography System using prepacked Biotage® Sfär HC Duo cartridges 25g (12 CV 0→100% ethyl acetate in petroleum ether) to give the pure product as a pale yellow oil (***d₁*-2q,** 126.0 mg, 0.365 mmol, 95% deuterium incorporation, 73% isolated yield, *Z/E* = 5.1/1).

^{1}H NMR (400 MHz, $CDCl_3$) δ 7.15 – 7.04 (m, 2H), 6.91 – 6.82 (m, 2H), 5.65 – 5.37 (m, 2H), 3.57 (t, *J* = 5.2 Hz, 4H), 3.32 (t, *J* = 7.5 Hz, 4H), 3.35 – 3.28 (m, 1.05H), 2.17 – 2.08 (m, 1.7H, *Z* isomer), 2.04 – 1.95 (m, 0.3H, *E* isomer)
, 1.48 (s, 9H), 1.46 – 1.37 (m, 2H), 0.94 (t, *J* = 7.4 Hz, 3H).

^{2}H NMR (61 MHz, $CHCl_3$) δ 3.32 (s, 1D).

^{13}C NMR (101 MHz, $CDCl_3$) δ 154.9, 149.7, 133.3, 130.6, 129.1, 128.6, 117.1, 80.0, 50.0, 43.7, 32.71 (d_0-isotopolog), 32.38 (t, J = 19.8 Hz), 29.43, 28.58, 23.0, 14.0.

HRMS: (ESI$^+$) m/z [M+H]$^+$ calculated for $C_{21}H_{32}DN_2O_2^+$ 346.2599; found 346.2590

8-(2-phenylethylidene-2-*d*)-1,4-dioxaspiro[4.5]decane [*d₁*-2s]

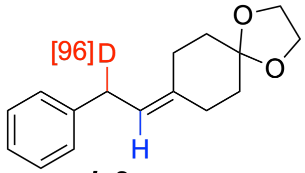

***d*$_1$-2s**

According to the general procedure using 0.2 mmol substrate, DTB-DPPBz (3.94 mg, 0.00440 mmol), $Cu(OAc)_2$ (20 µL of a 0.200 M solution in THF), diethoxy(methyl)silane (96 µL, 0.600 mmol, 3.00 eq), and THF (0.18 mL) were combined in a 1-dram vial followed by addition of a solution of 8-(2-phenylvinylidene)-1,4-dioxaspiro[4.5]decane (48.5 mg, 0.200 mmol, 1.00 eq.), THF (0.20 mL), and 2-propanol-*d* (61 µL, 0.800 mmol, 4.00 eq.). The 2-dram vial was capped with a pressure relief cap, and the reaction stirred for 20 h at room temperature. Upon completion, the crude product was dry loaded onto silica gel and isolated by flash column chromatography (150 mL of 5% diethyl ether in Petroleum Ether) to give the pure product as a colorless oil (***d*$_1$-2s,** 37.3 mg, 0.152 mmol, 96% deuterium incorporation, 76% isolated yield).

^{1}H NMR (500 MHz, $CDCl_3$) δ 7.31 – 7.26 (m, 2H), 7.21 – 7.15 (m, 3H), 5.35 (d, J = 7.5 Hz, 1H), 3.98 (s, 4H), 3.40 – 3.33 (m, 1.04H), 2.40 (t, J = 6.5 Hz, 2H), 2.29 (d, J = 6.7 Hz, 2H), 1.72 (p, J = 3.8 Hz, 4H).

^{2}H NMR (77 MHz, $CDCl_3$) δ 3.33 (s, 1D).

^{13}C NMR (126 MHz, $CDCl_3$) δ 141.6, 137.9, 128.54, 128.45, 125.9, 121.5, 109.2, 64.5, 36.4, 35.7, 33.7, 33.5 (t, J = 19.3 Hz), 25.3.

HRMS: (ESI$^+$) m/z [M+H]$^+$ calculated for $C_{16}H_{20}DO_2^+$ 246.1599; found 246.1595

(hept-1-en-3-yl-3-*d*)benzene [*d₁*-2t]

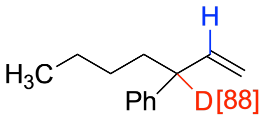

***d*$_1$-2t**

According to the general procedure using 0.2 mmol substrate, DTB-DPPBz (3.94 mg, 0.00440 mmol), $Cu(OAc)_2$ (20 µL of a 0.200 M solution in THF), diethoxy(methyl)silane (96 µL, 0.600 mmol, 3.00 eq), and THF (0.18 mL) were combined in a 1-dram vial followed by addition of a solution of hepta-1,2-dien-3-ylbenzene (34.5 mg, 0.200 mmol, 1.00 eq.), THF (0.20 mL), and 2-propanol-*d* (61 µL, 0.800 mmol, 4.00 eq.). The 2-dram vial was capped with a pressure relief cap, and the reaction stirred for 20 h at room temperature. Upon completion, the crude product was dry loaded onto silica gel and isolated by flash column chromatography (150 mL of 5% diethyl ether in Petroleum Ether) to give the pure product as a colorless oil (***d*$_1$-2t,** 21.4 mg, 0.122 mmol, 88% deuterium incorporation, 61% isolated yield).

^{1}H NMR (400 MHz, $CDCl_3$) δ 7.35 – 7.27 (m, 2H), 7.24 – 7.18 (m, 3H), 5.97 (dd, J = 17.0, 10.4 Hz, 1H), 5.08 – 5.00 (m, 2H), 3.29 – 3.21 (m, 0.12 H), 1.78 – 1.68 (m, 2H), 1.35 – 1.27 (m, 4H), 0.89 (t, J = 7.1 Hz, 3H).

^{2}H NMR (61 MHz, $CHCl_3$) δ 3.25 (s, 1D).

^{13}C NMR (101 MHz, $CDCl_3$) δ 144.8, 142.7, 128.5, 127.7, 126.2, 114.0, 50.1 (d_0-isotopolog), 49.6 (t, J = 19.2 Hz), 35.2, 29.9, 22.8, 14.2.

HRMS: (ESI$^+$) m/z [M+H]$^+$ calculated for $C_{13}H_{18}D^+$ 176.1544; found 176.1544

(6-methylhept-4-en-1-yl-6-*d*)benzene [*d₁*-2u]

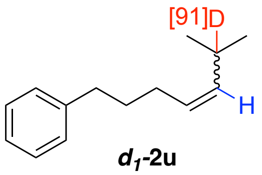

***d₁*-2u**

According to the general procedure, DTB-DPPBz (3.94 mg, 0.00440 mmol), $Cu(OAc)_2$ (20 µL of a 0.200 M solution in THF), diethoxy(methyl)silane (96 µL, 0.600 mmol, 3.00 eq), and THF (0.18 mL) were combined in a 1-dram vial followed by addition of a solution of (6-methylhepta-4,5-dien-1-yl)benzene (37.3 mg, 0.200 mmol, 1.00 eq.), THF (0.20 mL), and 2-propanol-*d* (61 µL, 0.800 mmol, 4.00 eq.). The 2-dram vial was capped with a pressure relief cap, and the reaction stirred for 20 h at room temperature. Upon completion, the crude product was dry loaded onto silica gel and isolated by flash column chromatography (150 mL of 5% diethyl ether in Hexane) to give the pure product as a colorless oil (***d₁*-2u,** 16.8 mg, 0.090 mmol, 91% deuterium incorporation, 45% isolated yield, mixture of Z and E isomers).

^{1}H NMR (400 MHz, $CDCl_3$) δ 7.33 – 7.26 (m, 2H), 7.23 – 7.16 (m, 3H), 5.51 – 5.30 (m, 2H), 2.67 – 2.56 (t, J = 7.8 Hz, 2H), 2.27 – 2.24 (m, 0.09H), 2.08 – 2.00 (m, 2H), 1.76 – 1.64 (m, 2H), 1.00 – 0.95 (m, 6H).

^{2}H NMR (61 MHz, $CDCl_3$) δ 2.26 (s, 1D).

^{13}C NMR (101 MHz, $CDCl_3$) δ 142.8, 138.2, 128.6, 128.4, 126.8, 125.7, 35.5, 32.2, 31.5, 31.20, 30.76 (t, J = 19.0 Hz), 22.8.

HRMS: (ESI$^+$) m/z [M]$^+$ calculated for $C_{14}H_{19}D^+$ 189.1622; found 189.1624

Note: The exact E/Z ratio cannot be determined by diastereotopic protons due to the low single intensity.

(non-2-en-1-yl-1-*d*)cyclopentane [*d₁*-2v]

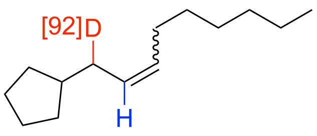

***d₁*-2v**

According to the general procedure, DTB-DPPBz (3.94 mg, 0.00440 mmol), $Cu(OAc)_2$ (20 µL of a 0.200 M solution in THF), diethoxy(methyl)silane (96 µL, 0.600 mmol, 3.00 eq), and THF (0.18 mL) were combined in a 1-dram vial followed by addition of a solution of nona-1,2-dien-1-ylcyclopentane (38.5 mg, 0.200 mmol, 1.00 eq.), THF (0.20 mL), and 2-propanol-*d* (61 µL, 0.800 mmol, 4.00 eq.). The 2-dram vial was capped

with a pressure relief cap, and the reaction stirred for 20 h at room temperature. Upon completion, the crude product was dry loaded onto silica gel and isolated by flash column chromatography (150 mL of 5% diethyl ether in Petroleum Ether) to give the pure product as a colorless oil (***d₁*-2v,** 36.0 mg, 0.180 mmol, 92% deuterium incorporation based on quantitative^{13}C NMR, 90% isolated yield, mixture of Z and E isomers).

^{1}H NMR (400 MHz, $CDCl_3$) δ 5.42 – 5.22 (m, 2H), 2.06 – 1.89 (m, 3H), 1.78 – 1.66 (m, 2H), 1.64 – 1.55 (m, 2H), 1.53 – 1.44 (m, 2H), 1.35 – 1.19 (m, 9H), 1.17 – 1.07 (m, 2H), 0.88 (t, 3H).

^{2}H NMR (77 MHz, $CDCl_3$) δ 2.02 (s, 1D).

^{13}C NMR (101 MHz, $CDCl_3$) δ 130.2, 129.2, 40.4, 33.1 (t, J = 18.7 Hz), 32.4, 31.9, 29.9, 29.2, 27.5, 25.3, 22.8, 14.3.

HRMS: (ESI$^+$) m/z $[M-H]^+$ calculated for $C_{14}H_{24}D^+$ 194.2014; found 194.2013

Note: The exact E/Z ratio cannot be determined by Diastereotopic protons due to the low single intensity.

Spectroscopic data were consistent with literature report for the d_0-isotopolog.[9], [10]

(*Z*)-trimethyl(5-phenylpent-2-en-1-yl-1-*d*)silane [*d₁*-2w]

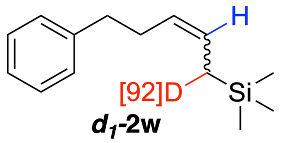

***d₁*-2w**

According to the general procedure, DTB-DPPBz (3.94 mg, 0.00440 mmol), $Cu(OAc)_2$ (20 µL of a 0.200 M solution in THF), diethoxy(methyl)silane (96 µL, 0.600 mmol, 3.00 eq), and THF (0.18 mL) were combined in a 1-dram vial followed by addition of a solution of trimethyl(5-phenylpenta-1,2-dien-1-yl)silane (43.2 mg, 0.200 mmol, 1.00 eq.), THF (0.20 mL), and 2-propanol-*d* (61 µL, 0.800 mmol, 4.00 eq.). The 2-dram vial was capped with a pressure relief cap, and the reaction stirred for 8 h at room temperature. Upon completion, the crude product was dry loaded onto silica gel and isolated by flash column chromatography (150 mL of 100% Petroleum Ether) to give the pure product as a colorless oil (***d₁*-2w,** 37.8 mg, 0.1720 mmol, 92% deuterium incorporation, 86% isolated yield, *Z/E* = 12:1).

^{1}H NMR (500 MHz, $CDCl_3$) δ 7.33 – 7.26 (m, 3H), 7.25 – 7.16 (m, 2H), 5.48 – 5.38 (m, 1H), 5.37 – 5.26 (m, 1H), 2.67 (t, J = 8.0 Hz, 2H), 2.33 (q, J = 8.2 Hz, 2H), 1.53 – 1.42 (m, 1.08 H), 0.02 (s, 8.3H, *Z* isomer), -0.02 (s, 0.6H, *E* isomer).

^{2}H NMR (77 MHz, $CDCl_3$) δ 1.46 (s, 1D).

^{13}C NMR (126 MHz, $CDCl_3$) δ 142.5, 128.6, 128.4, 126.7, 126.2, 125.9, 36.2, 29.2, 18.7 (d_0-isotopolog), 18.33 (t, J = 18.6 Hz), -1.6.

HRMS: (ESI$^+$) m/z $[M-TMS]^+$ calculated for $C_{11}H_{12}D^+$ 146.1075; found 146.1074

Spectroscopic data were consistent with the literature report for the d_0-*Z*-isotopolog.[11]

(1*S*,4*R*)-1-isopropyl-4-methyl-2-vinylcyclohexane-2-*d* [*d₁*-2x]

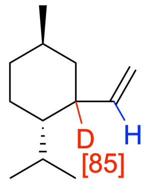

20:1 d.r.

***d₁*-2x**

According to the general procedure, DTB-DPPBz (9.85 mg, 0.0110 mmol), $Cu(OAc)_2$ (100 µL of a 0.100 M solution in THF), diethoxy(methyl)silane (153 µL, 1.50 mmol, 3.00 eq), and THF (0.40 mL) were combined in a 1-dram vial followed by addition of a solution of (1*S*,4*R*)-1-isopropyl-4-methyl-2-vinylidenecyclohexane (82.1 mg, 0.500 mmol, 1.00 eq.), THF (0.50 mL), and 2-propanol-*d* (240 µL, 2.00 mmol, 4.00 eq.). The 2-dram vial was capped with a pressure relief cap, and the reaction stirred for 8 h at room temperature. Upon completion, the crude product was dry loaded onto silica gel and isolated by flash column chromatography (250 mL of 100% Petroleum Ether) to give the pure product as a colorless oil (***d_1*-2x,** 56.7 mg, 0.338 mmol, 85% deuterium incorporation, 68% isolated yield).

^{1}H NMR (500 MHz, $CDCl_3$, major diastereomer) δ 6.07 – 5.98 (m, 1H), 5.09 – 4.99 (m, 2H), 2.60 – 2.55 (m, 0.15H), 2.03 – 1.62 (m, 5H), 1.60 – 1.55 (m, 1H), 1.34 – 1.29 (m, 1H), 1.20 – 1.14 (m, 2H), 0.87 (d, *J* = 4.3 Hz, 3H), 0.86 (d, *J* = 4.1 Hz, 3H), 0.83 (d, *J* = 6.4 Hz, 3H).

^{2}H NMR (77 MHz, $CDCl_3$) δ 2.57 (s, 1D).

^{13}C NMR (126 MHz, $CDCl_3$) δ 139.76, 114.91, 47.21, 42.92, 41.6 (d_0-isotopolog), 41.11 (t, *J* = 19.4 Hz), 36.01, 30.35, 26.97, 25.71, 23.02, 21.29, 20.77. Due to the present of diastereomers, extra signals can be found near the major signals.
HRMS: (ESI^+) *m/z* $[M-H]^+$ calculated for $C_{12}H_{20}D^+$ 166.1701; found 166.1698

(8*S*,9*S*,13*R*,14*S*)-3-methoxy-13-methyl-17-vinyl-7,8,9,11,12,13,14,15,16,17-decahydro-6*H*-cyclopenta[*a*]phenanthrene-17-*d* [*d_1*-2y]

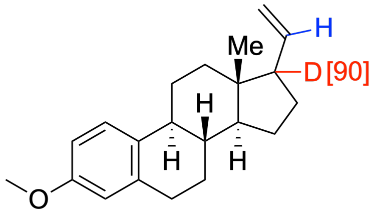

10:1 d.r.

***d₁*-2y**

According to the general procedure, DTB-DPPBz (1.97 mg, 0.00200 mmol), $Cu(OAc)_2$ (20 µL of a 0.100 M solution in THF), diethoxy(methyl)silane (80 µL, 0.500 mmol, 2.50 eq), and THF (0.180 mL) were combined in a 1-dram vial followed by addition of a solution of (8*S*,9*S*,13*S*,14*S*)-3-methoxy-13-methyl-17-vinylidene-7,8,9,11,12,13,14,15,16,17-decahydro-6*H*-cyclopenta[*a*]phenanthrene (58.8 mg, 0.200 mmol, 1.00 eq.), THF (0.20 mL), and 2-propanol-*d* (18 µL, 0.300 mmol, 1.50 eq.). The 2-dram vial was capped with a pressure relief cap, and the reaction stirred for 15 minutes at room temperature. Upon completion, the crude product was dry loaded onto silica gel and isolated by flash column chromatography (350 mL of 50% DCM in Petroleum Ether) to give the pure product as a white solid (***d_1*-2y,** 47.5 mg, 0.159 mmol, 90% deuterium incorporation, 79% isolated yield).

^{1}H NMR (500 MHz, $CDCl_3$) δ 7.22 (d, *J* = 8.7 Hz, 1H), 6.71 (dd, *J* = 8.6, 3.0 Hz, 1H), 6.66 – 6.61 (m, 1H), 5.90 – 5.73 (m, 1H), 5.06 – 4.81 (m, 2H), 3.78 (s, 3H), 2.98 – 2.77 (m, 2H), 2.25 – 2.17 (m, 1H), 2.25 – 2.17

(m, 1H), 2.11 – 2.05 (m, 0.1H), 1.97 – 1.71 (m, 4H), 1.66 – 1.56 (m, 1H), 1.53 – 1.46 (m, 1H), 1.45 – 1.35 (m, 2H), 1.33 – 1.19 (m, 3H), 0.64 (s, 3H).

^{2}H NMR (77 MHz, $CDCl_3$) δ 2.05 (s, 1D).

^{13}C NMR (126 MHz, $CDCl_3$) δ 157.56, 139.92, 138.21, 133.13, 126.45, 114.77, 113.95, 111.56, 55.35, 54.84, 44.24, 43.95, 39.08, 37.64, 30.08, 28.28 (t, J = 15.7 Hz), 28.05, 27.38, 26.48, 24.67, 13.02. Contains a mixture of inseparable diastereomers.

HRMS: (ESI$^+$) m/z [M+H]$^+$ calculated for $C_{21}H_{28}DO^+$ 298.2276; found 298.2270

2-(4-(hex-2-en-1-yl-1-*d*)phenyl)isoindolin-1-one [*d₁*-2z]

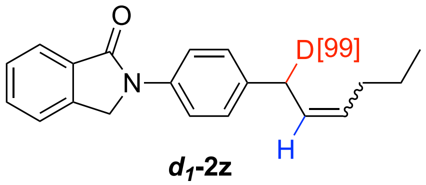

***d*$_1$-2z**

According to the general procedure, DTB-DPPBz (9.85 mg, 0.0110 mmol), $Cu(OAc)_2$ (100 µL of a 0.100 M solution in THF), diethoxy(methyl)silane (240 µL, 1.500 mmol, 2.00 eq), and THF (0.9 mL) were combined in a 1-dram vial followed by addition of a solution of 2-(4-(hexa-1,2-dien-1-yl)phenyl)isoindolin-1-one (171.2 mg, 0.500 mmol, 1.00 eq.), 1,4-dioxane (1.00 mL), and 2-propanol-*d* (153 µL, 2.00 mmol, 4.00 eq.). The 2-dram vial was capped with a pressure relief cap, and the reaction stirred for 20 h at room temperature. Upon completion, the reaction was quenched with sat. NH_4F solution in methanol. The result solution was further stirred at room temperature for 1h, then the mixture was transferred to a 100 mL round bottom flask with methanol, after removing the solvent by rotary evaporation, the crude product was dry loaded onto silica gel and isolated by Biotage® Isolera™ One Flash Chromatography System using prepacked Biotage® Sfär HC Duo cartridges 25g (12 CV 0 →100% ethyl acetate in hexane) to give the pure product as a pale yellow solid (***d*$_1$-2z,** 122.0 mg, 0.415 mmol, 99% deuterium incorporation, 83% isolated yield, *Z/E* = 5/1).

^{1}H NMR (400 MHz, $CDCl_3$) δ 7.95 – 7.90 (m, 1H), 7.80 – 7.74 (m, 2H), 7.62 – 7.55 (m, 1H), 7.47 – 7.54 (m, 2H), 7.27 – 7.23 (m, 2H), 5.61 – 5.49 (m, 2H), 4.84 (s, 2H), 3.42 – 3.30 (m, 1.01H), 2.19 – 2.10 (m, 1.61H, *Z* isomer), 2.05 – 1.96 (m, 0.32 H, *E* isomer), 1.44 (sext, J = 7.2 Hz, 2H), 0.95 (t, J = 7.4 Hz, 2.36H), 0.91 (t, J = 7.3 Hz, 0.49H).

^{2}H NMR (61 MHz, $CDCl_3$) δ 3.39 (s, 1D).

^{13}C NMR (101 MHz, $CDCl_3$) δ 167.5, 140.3, 137.7, 137.5, 133.5, 132.1, 131.1, 129.1, 128.5, 128.1, 124.3, 122.7, 119.9, 51.0, 33.1 (d_0-isotopolog), 32.73 (t, J = 19.5 Hz), 29.5, 23.0, 14.0.

HRMS: (ESI$^+$) m/z [M+H]$^+$ calculated for $C_{20}H_{21}DNO^+$ 293.1759; found 293.1752

D. Synthesis of Allyl-d_n Compounds and Isotopologs.

Figure S2. Reaction Scope for Isotopomer Syntheses

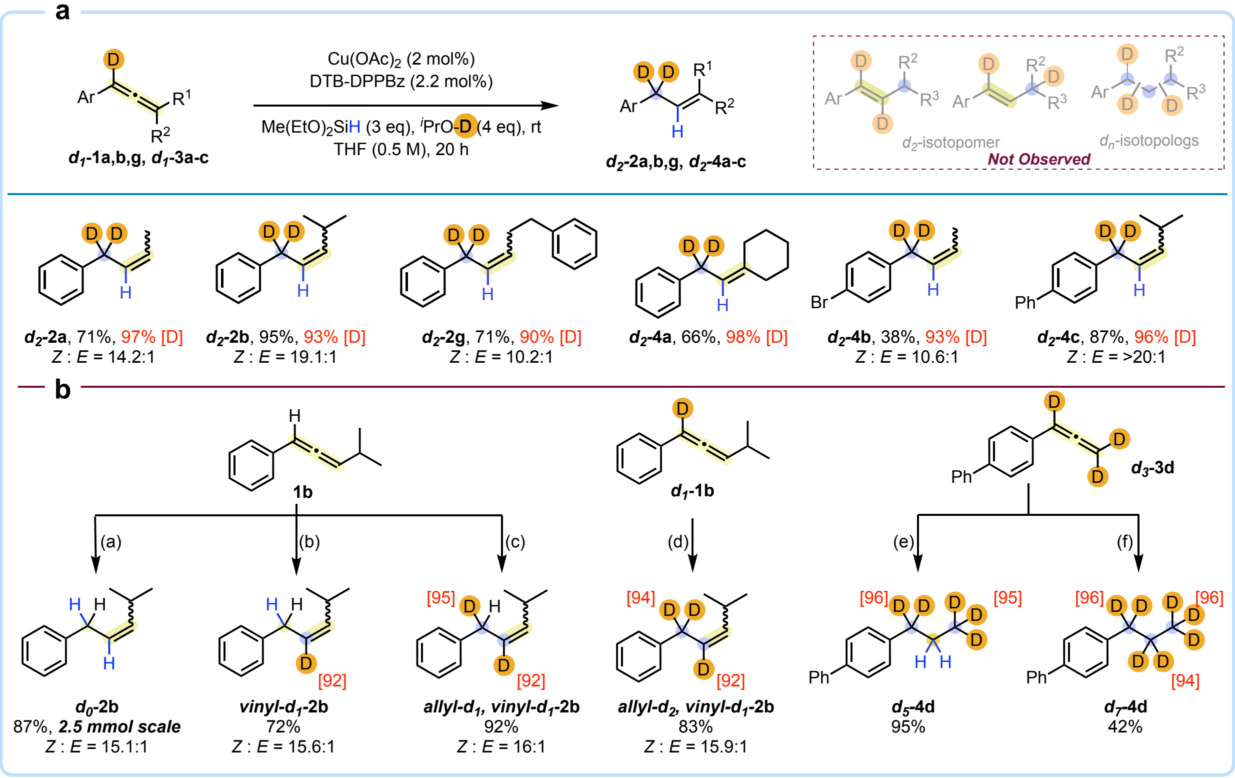

(but-2-en-1-yl-1,1-d_2)benzene [d_2-2a]

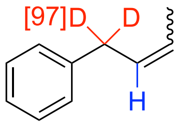

***d_2*-2a**

According to the general procedure, DTB-DPPBz (9.85 mg, 0.110 mmol), $Cu(OAc)_2$ (100 µL of a 0.100 M solution in THF), diethoxy(methyl)silane (240 µL, 1.50 mmol, 3.00 eq), and THF (0.400 mL) were combined in a 1-dram vial followed by addition of a solution of (buta-1,2-dien-1-yl-1-d)benzene (65.6 mg, 0.500 mmol, 1.00 eq.), THF (0.500 mL), and 2-propanol-d (153 µL, 2.00 mmol, 4.00 eq.). The 2-dram vial was capped with a pressure relief cap, and the reaction stirred for 20 h at room temperature. Upon completion, the crude product was dry loaded onto silica gel and isolated by flash column chromatograph (250 mL of 100% Hexane) to give the pure product as colorless oil (***d_2*-2a**, 48.0 mg, 0.355 mmol, 97% deuterium incorporation, 71% isolated yield, *Z/E* = 14.2/1).

<u>^{1}H NMR (500 MHz, $CDCl_3$)</u> δ 7.33 – 7.28 (m, 2H), 7.24 – 7.19 (m, 3H), 5.68 – 5.56 (m, 2H), 3.42 (s, 0.06H), 1.77 – 1.74 (m, 2.84H, *Z* isomer), 1.72 – 1.70 (m, 0.2H, *E* isomer).

^{2}H NMR (77 MHz, CDCl$_3$) δ 3.48 (d, *J* = 7.5 Hz, 2D).

^{13}C NMR (126 MHz, CDCl$_3$) δ 141.3, 129.1, 128.55, 128.48, 126.0, 125.0, 33.1 (d$_0$-isotopolog), 32.6 (p, *J* = 19.3 Hz), 13.0.

HRMS: (ESI$^+$) *m/z* [M-H]$^+$ calculated for $C_{10}H_9D_2^+$ 133.0981; found 133.0980

Spectroscopic data were consistent with literature reported values. [1]

(4-methylpent-2-en-1-yl-1,1-*d*$_2$)benzene [*d*$_2$-2b]

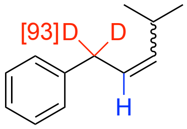

***d*$_2$-2b**

According to the general procedure, DTB-DPPBz (19.7 mg, 0.022 mmol), Cu(OAc)$_2$ (200 µL of a 0.100 M solution in THF), diethoxy(methyl)silane (480 µL, 3.00 mmol, 3.00 eq), and THF (0.800 mL) were combined in a 1-dram vial followed by addition of a solution of (4-methylpenta-1,2-dien-1-yl-1-*d*)benzene (159.2 mg, 1.00 mmol, 1.00 eq.), THF (1.00 mL), and 2-propanol-d (306 µL, 4.00 mmol, 4.00 eq.). The 2-dram vial was capped with a pressure relief cap, and the reaction stirred for 20 h at room temperature. Upon completion, the crude product was dry loaded onto silica gel and isolated by flash column chromatograph (500 mL of 100% Petroleum ether) to give the pure product as colorless oil (***d*$_2$-2b**, 154.8 mg, 0.95 mmol, 93% deuterium incorporation, 95% isolated yield, *Z/E* = 19.1/1).

^{1}H NMR (500 MHz, CDCl$_3$) δ 7.34 – 7.27 (m, 2H), 7.23 – 7.18 (m, 3H), 5.54 – 5.33 (m, 2H), 3.43 – 3.39 (m, 0.14H), 2.82 – 2.70 (m, 1H), 1.03 (d, *J* = 6.7 Hz, 6.29H, *Z* isomer), 0.90 (d, *J* = 6.7 Hz, 0.33H, *E* isomer).

^{2}H NMR (77 MHz, CDCl$_3$) δ 3.40 (s, 2D).

^{13}C NMR (126 MHz, CDCl$_3$) δ 141.3, 138.6, 128.6, 128.5, 126.0, 125.6, 33.2 (p, *J* = 20.4 Hz), 26.7, 23.3.

HRMS: (ESI$^+$) *m/z* [M-D]$^+$ calculated for $C_{12}H_{14}D^+$ 160.1231; found 160.1230

Spectroscopic data were consistent with literature reported *d*$_0$-Z-isotopolog.[12]

(pent-2-ene-1,5-diyl-1,1-*d*$_2$)dibenzene [*d*$_2$-2g]

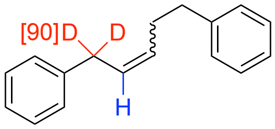

***d*$_2$-2g**

According to the general procedure, DTB-DPPBz (3.94 mg, 0.0044 mmol), $Cu(OAc)_2$ (40 μL of a 0.100 M solution in THF), diethoxy(methyl)silane (96 μL, 0.6 mmol, 3.00 eq), and THF (0.128 mL) were combined in a 1-dram vial followed by addition of a solution of (penta-1,2-diene-1,5-diyl-1-*d*)dibenzene (44.2 mg, 0.200 mmol, 1.00 eq.), THF (0.200 mL), and 2-propanol-d (61 μL, 0.8 mmol, 4 eq.). The 2-dram vial was capped with a pressure relief cap, and the reaction stirred for 20 h at room temperature. Upon completion, the crude product was dry loaded onto silica gel and isolated by flash column chromatograph (250 mL of 100% Hexane) to give the pure product as colorless oil (***d*₂-2g**, 32.0 mg, 0.142 mmol, 90% deuterium incorporation, 71% isolated yield, *Z/E* = 10.2/1).

1H NMR (500 MHz, $CDCl_3$) δ 7.32 – 7.24 (m, 4H), 7.24 – 7.17 (m, 4H), 7.15 – 7.09 (m, 2H), 5.70 – 5.51 (m, 2H), 3.35 – 3.30 (m, 0.19H), 2.80 – 2.67 (m, 2H), 2.54 – 2.44 (m, 1.74H, *Z* isomer), 2.39 – 2.34 (m, 0.17H, *E* isomer).

^{2}H NMR (77 MHz, $CDCl_3$) δ 3.28 (s, 2D).

^{13}C NMR (126 MHz, $CDCl_3$) δ 142.0, 141.0, 129.9, 128.9, 128.7, 128.53, 128.47, 126.00, 125.98, 36.1, 33.6 (d_0-isotopolog), 33.1 (p, J = 19.6 Hz), 29.4.

HRMS: (ESI$^+$) *m/z* $[M+H]^+$ calculated for $C_{17}H_{17}D_2^+$ 225.1607; found 225.1603

Spectroscopic data were consistent with literature report for the d_0-*Z*-isotopolog.[5]

(2-cyclohexylideneethyl-1,1-*d*₂)benzene [*d*₂-4a]

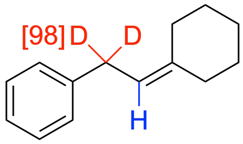

***d*₂-4a**

According to the general procedure, DTB-DPPBz (3.94 mg, 0.0044 mmol), $Cu(OAc)_2$ (40 μL of a 0.100 M solution in THF), diethoxy(methyl)silane (96 μL, 0.6 mmol, 3.00 eq), and THF (0.160 mL) were combined in a 1-dram vial followed by addition of a solution of (2-cyclohexylidenevinyl-1-d)benzene (37.0 mg, 0.200 mmol, 1.00 eq.), THF (0.200 mL), and 2-propanol-d (61 μL, 0.8 mmol, 4 eq.). The 2-dram vial was capped with a pressure relief cap, and the reaction stirred for 20 h at room temperature. Upon completion, the crude product was dry loaded onto silica gel and isolated by flash column chromatograph (250 mL of 100% Hexane) to give the pure product as colorless oil (***d*₂-4a**, 25.0 mg, 0.132 mmol, 98% deuterium incorporation, 66% isolated yield).

^{1}H NMR (500 MHz, $CDCl_3$) δ 7.33 – 7.28 (m, 2H), 7.25 – 7.16 (m, 3H), 5.29 (s, 1H), 3.39 – 3.34 (m, 0.05H), 2.31 – 2.25 (m, 2H), 2.18 – 2.12 (m, 2H), 1.65 – 1.54 (m, 6H).

^{2}H NMR (77 MHz, $CDCl_3$) δ 3.40 (s, 2D).

^{13}C NMR (126 MHz, CDCl$_3$) δ 142.1, 140.8, 128.49, 128.47, 125.8, 119.8, 37.3, 33.4 (d_0-isotopolog), 32.9 (p, J = 19.5 Hz), 28.9, 28.8, 28.0, 27.1.

HRMS: (ESI$^+$) m/z [M+H]$^+$ calculated for $C_{14}H_{17}D_2^+$ 189.1607; found 189.1604

1-bromo-4-(but-2-en-1-yl-1,1-*d*$_2$)benzene [*d*$_2$-4b]

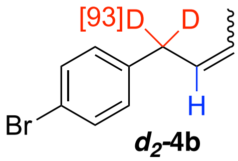

***d*$_2$-4b**

According to the general procedure, DTB-DPPBz (3.94 mg, 0.0044 mmol), Cu(OAc)$_2$ (40 μL of a 0.100 M solution in THF), diethoxy(methyl)silane (96 μL, 0.6 mmol, 3.00 eq), and THF (0.160 mL) were combined in a 1-dram vial followed by addition of a solution of 1-bromo-4-(buta-1,2-dien-1-yl-1-*d*)benzene (42.0 mg, 0.200 mmol, 1.00 eq.), THF (0.200 mL), and 2-propanol-d (61 μL, 0.800 mmol, 4 eq.). The 2-dram vial was capped with a pressure relief cap, and the reaction stirred for 20 h at room temperature. Upon completion, the crude product was dry loaded onto silica gel and isolated by flash column chromatograph (350 mL of 100% Hexane) to give the pure product as colorless oil (***d*$_2$-4b**, 16.2 mg, 0.076 mmol, 93% deuterium incorporation, 38% isolated yield, *Z/E* = 10.6/1).

^{1}H NMR (500 MHz, CDCl$_3$) δ 7.42 (d, J = 8.4 Hz, 2H), 7.09 (d, J = 8.4 Hz, 2H), 5.69 – 5.59 (m, 1H), 5.59 – 5.52 (m, 1H), 3.40 – 3.33 (m, 0.13H), 1.73 (dd, J = 6.7, 1.7 Hz, 2.67H, *Z* isomer), 1.71 (d, J = 5.0 Hz, 0.25H, *E* isomer).

^{2}H NMR (77 MHz, CDCl$_3$) δ 3.36 (s, 2D).

^{13}C NMR (126 MHz, CDCl$_3$) δ 140.2, 131.6, 130.2, 128.4, 125.6, 119.7, 32.2 (p, J = 20.2 Hz), 13.0.

HRMS: (ESI$^+$) m/z [M+H]$^+$ calculated for $C_{10}H_{10}D_2Br^+$ 213.0242; found 213.0239

Spectroscopic data were consistent with literature reported d_0-isotopolog as *E/Z* mixture.[13]

4-(4-methylpent-2-en-1-yl-1,1-*d*$_2$)-1,1'-biphenyl [*d*$_2$-4c]

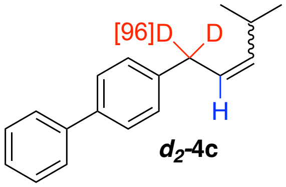

***d*$_2$-4c**

According to the general procedure, DTB-DPPBz (9.85 mg, 0.110 mmol), Cu(OAc)$_2$ (100 μL of a 0.100 M solution in THF), diethoxy(methyl)silane (240 μL, 1.50 mmol, 3.00 eq), and THF (0.400 mL) were combined

in a 1-dram vial followed by addition of a solution of 4-(4-methylpenta-1,2-dien-1-yl-1-*d*)-1,1'-biphenyl (117.6 mg, 0.500 mmol, 1.00 eq.), THF (0.500 mL), and 2-propanol-d (153 μL, 2.00 mmol, 4 eq.). The 2-dram vial was capped with a pressure relief cap, and the reaction stirred for 20 h at room temperature. Upon completion, the crude product was dry loaded onto silica gel and isolated by flash column chromatograph (200 mL of 100% Hexane → 400 mL of 1% diethyl ether in Hexane) to give the pure product as colorless oil (***d₂*-4c**, 103.8 mg, 0.435 mmol, 96% deuterium incorporation, 87% isolated yield, *Z/E* > 20/1).

^{1}H NMR (500 MHz, $CDCl_3$) δ 7.57 (d, *J* = 7.0 Hz, 2H), 7.51 (d, *J* = 8.2 Hz, 2H), 7.40 (t, *J* = 8.1 Hz, 2H), 7.31 (t, *J* = 7.4 Hz, 1H), 7.25 (d, *J* = 8.4 Hz, 2H), 5.55 – 5.51 (m, 0.13H, minor isomer), 5.44 (d, *J* = 10.8 Hz, 1H, major isomer), 5.36 (t, *J* = 9.6 Hz, 1H), 3.44 – 3.39 (m, 0.08H), 2.84 – 2.66 (m, 1H), 1.02 (d, *J* = 6.7 Hz, 5.82H, *Z* isomer), 0.89 (dd, *J* = 6.6, 2.0 Hz, 0.19H, *E* isomer).

^{2}H NMR (77 MHz, $CDCl_3$) δ 3.36 (s, 2D).

^{13}C NMR (126 MHz, $CDCl_3$) δ 141.3, 140.4, 139.5, 139.0, 138.8, 128.9, 128.9, 127.3, 127.2, 125.5, 33.2 (d_0-isotopolog), 32.7 (p, *J* = 19.3 Hz), 26.7, 23.4.

HRMS: (ESI$^+$) *m/z* [M-D]$^+$ calculated for $C_{18}H_{18}D^+$ 236.1544; found 236.1536

Spectroscopic data were consistent with literature reported d_0-isotopolog as *E/Z* mixture.[14]

(4-methylpent-2-en-1-yl)benzene [*d₀*-2b]

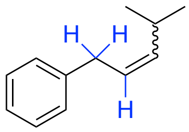

***d₀*-2b**

According to the general procedure, DTB-DPPBz (49.2 mg, 0.0550 mmol), $Cu(OAc)_2$ (500 μL of a 0.100 M solution in THF), diethoxy(methyl)silane (1.20 mL, 7.50 mmol, 3.00 eq), and THF (2.00 mL) were combined in a 20 mL vial followed by addition of a solution of (4-methylpenta-1,2-dien-1-yl)benzene (396 mg, 2.500 mmol, 1.00 eq.), THF (5.00 mL), and 2-propanol-d (766 μL, 10.00 mmol, 4 eq.). The 20 mL vial was capped with a pressure relief cap, and the reaction stirred for 20 h at room temperature. Upon completion, the crude product was dry loaded onto silica gel and isolated by flash column chromatograph (800 mL of 100% Pentane) to give the pure product as colorless oil (***d₀*-2b**, 350.0 mg, 2.17 mmol, 87% isolated yield, *Z/E* = 15.1/1).

^{1}H NMR (400 MHz, $CDCl_3$) δ 7.35 – 7.26 (m, 2H), 7.24 – 7.15 (m, 3H), 5.54 – 5.39 (m, 1H), 5.38 – 5.27 (m, 1H), 3.42 (d, *J* = 7.2 Hz, 1.82H, *Z* isomer), 3.33 (d, *J* = 5.0 Hz, 0.12H, *E* isomer), 2.83 – 2.67 (m, 1H), 1.02 (d, *J* = 6.7 Hz, 6H).

Spectroscopic data were consistent with literature reported values.[2]

(4-methylpent-2-en-1-yl-2-*d*)benzene [*d₁*-2b]

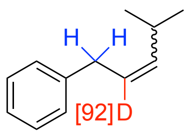

***d*₁-2b**

According to the general procedure, DTB-DPPBz (3.94 mg, 0.0440 mmol), $Cu(OAc)_2$ (40 µL of a 0.100 M solution in THF), DMMS-D (64.3 mg, 0.600 mmol, 3.00 eq), and THF (0.160 mL) were combined in a 2-dram vial followed by addition of a solution of (4-methylpenta-1,2-dien-1-yl)benzene (31.6 mg, 0.200 mmol, 1.00 eq.), THF (0.200 mL), and 2-propanol (61 µL, 0.800 mmol, 4 eq.). The 2-dram vial was capped with a pressure relief cap, and the reaction stirred for 40 h at room temperature. Upon completion, the crude product was dry loaded onto silica gel and isolated by flash column chromatograph (200 mL of 100% Petroleum ether) to give the pure product as colorless oil (***d_1*-2b**, 26.3 mg, 0.143 mmol, 12% DCM residue, 92% deuterium incorporation at vinyl position, 72% isolated yield, *Z/E* = 15.6/1).

<u>1H NMR (500 MHz, $CDCl_3$)</u> δ 7.33 – 7.27 (m, 2H), 7.25 – 7.18 (m, 3H), 5.53 – 5.49 (m, 0.08H), 5.47 – 5.40 (m, 0.04H), 5.40 – 5.32 (m, 0.95H), 3.42 (s, 2H, *Z* isomer), 3.34 (s, 0.13H, *E* isomer), 2.81 – 2.71 (m, 1H), 1.03 (d, *J* = 6.6 Hz, 6H).

<u>^{2}H NMR (77 MHz, $CDCl_3$)</u> δ 5.48 (s, 1D).

<u>^{13}C NMR (126 MHz, $CDCl_3$)</u> δ 141.4, 138.5, 128.5, 128.5, 126.0, 125.4 (t, *J* = 23.6 Hz), 33.7, 26.6, 23.3.

<u>HRMS:</u> (ESI^+) *m/z* $[M-H]^+$ calculated for $C_{12}H_{14}D$ 160.1231; found 160.1229

Spectroscopic data were consistent with literature reported d_0-*Z*-isotopolog.[2]

(4-methylpent-2-en-1-yl-1,2-*d_2*)benzene [*allyl-d_1*, vinyl-*d_1*-2b]

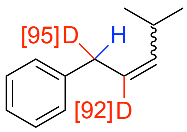

***allyl-d_1*, vinyl-*d_1*-2b**

According to the general procedure, DTB-DPPBz (9.85 mg, 0.110 mmol), $Cu(OAc)_2$ (100 µL of a 0.100 M solution in THF), DMMS-D (160.8 mg, 1.50 mmol, 3.00 eq), and THF (0.400 mL) were combined in a 1-dram vial followed by addition of a solution of (4-methylpenta-1,2-dien-1-yl)benzene (79.1 mg, 0.500 mmol, 1.00 eq.), THF (0.500 mL), and 2-propanol-d (153 µL, 2.00 mmol, 4 eq.). The 2-dram vial was capped with a pressure relief cap, and the reaction stirred for 40 h at room temperature. Upon completion, the crude product was dry loaded onto silica gel and isolated by flash column chromatograph (500 mL of 100% Petroleum ether) to give the pure product as colorless oil (***d_2*-2b**, 74.7 mg, 0.460 mmol, 95% deuterium

incorporation at allylic position, 92% deuterium incorporation at vinyl position, 92% isolated yield, *Z/E* = 16/1).

<u>^{1}H NMR (500 MHz, $CDCl_3$)</u> δ 7.34 – 7.27 (m, 2H), 7.24 – 7.16 (m, 3H), 5.53 – 5.48 (m, 0.08H), 5.46 – 5.40 (m, 0.04H), 5.35 (d, *J* = 1.7 Hz, 0.86H), 3.46 – 3.37 (m, 0.96H, *Z* isomer), 3.33 – 3.30 (m, 0.06H, *E* isomer), 2.82 – 2.69 (m, 1H), 1.02 (d, *J* = 6.7 Hz, 6H).

<u>^{2}H NMR (77 MHz, $CDCl_3$)</u> δ 5.44 (s, 1D), 3.40 (s, 1D).

<u>^{13}C NMR (126 MHz, $CDCl_3$)</u> δ 141.4, 138.5, 128.55, 128.47, 126.0, 125.3 (t, *J* = 23.8 Hz), 33.7 (d_0-isotopolog), 33.3 (t, *J* = 19.8 Hz), 26.6, 23.3.

<u>HRMS:</u> (ESI$^+$) *m/z* [M-H]$^+$ calculated for $C_{12}H_{13}D_2^+$161.1294; found 161.1292

Spectroscopic data were consistent with literature reported d_0-*Z*-isotopolog.[2]

(4-methylpent-2-en-1-yl-1,1,2-d_3)benzene [*allyl-d_2*, vinyl-d_1-2b]

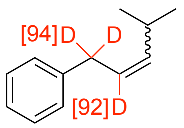

***allyl-d_2*, vinyl-d_1-2b**

According to the general procedure, DTB-DPPBz (3.94 mg, 0.0440 mmol), $Cu(OAc)_2$ (40 µL of a 0.100 M solution in THF), DMMS-D (64.3 mg, 0.600 mmol, 3.00 eq), and THF (0.160 mL) were combined in a 2-dram vial followed by addition of a solution of (4-methylpenta-1,2-dien-1-yl-1-*d*)benzene (31.8 mg, 0.200 mmol, 1.00 eq.), THF (0.200 mL), and 2-propanol-d (61 µL, 0.800 mmol, 4 eq.). The 2-dram vial was capped with a pressure relief cap, and the reaction stirred for 40 h at room temperature. Upon completion, the crude product was dry loaded onto silica gel and isolated by flash column chromatograph (200 mL of 100% Petroleum ether) to give the pure product as colorless oil (***d_3*-2b**, 32.7 mg, 0.165mmol, 17% DCM residue, 94% deuterium incorporation at allylic position, 92% deuterium incorporation at vinyl position, 83% isolated yield, *Z/E* = 15.9/1).

<u>^{1}H NMR (500 MHz, $CDCl_3$)</u> δ 7.32 – 7.28 (m, 2H), 7.23 – 7.18 (m, 3H), 5.53 – 5.49 (m, 0.08H), 5.44 (d, *J* = 11.6 Hz, 0.04H), 5.36 (d, *J* = 9.5 Hz, 0.94H), 3.43 – 3.38 (m, 0.12H), 2.82 – 2.69 (m, 1H), 1.03 (d, *J* = 6.6 Hz, 5.71H, *Z* isomer), 0.95 (d, *J* = 6.6, 0.36H, *E* isomer).

<u>^{2}H NMR (77 MHz, $CDCl_3$)</u> δ 5.48 (s, 1D), 3.40 (s, 2D).

<u>^{13}C NMR (126 MHz, $CDCl_3$)</u> δ 141.3, 138.5, 128.55, 128.47, 126.0, 125.1 (t, *J* = 24.1 Hz), 33.2 (p, *J* = 19.4 Hz), 26.6, 23.3.

<u>HRMS:</u> (ESI$^+$) *m/z* [M-H]$^+$ calculated for $C_{12}H_{12}D_3^+$ 162.1357; found 162.1354

Spectroscopic data were consistent with literature reported d_0-Z-isotopolog.[2]

4-(propyl-1,1,3,3,3-*d*5)-1,1'-biphenyl [*d*₅-4d]

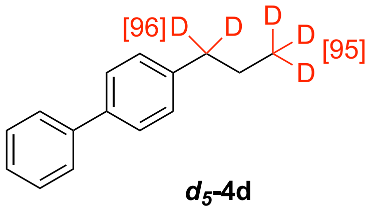

***d*₅-4d**

According to the general procedure, DTB-DPPBz (19.7 mg, 0.220 mmol), $Cu(OAc)_2$ (200 µL of a 0.100 M solution in THF), DMMS (123.5 µL, 1.00 mmol, 6.00 eq), and THF (0.300 mL) were combined in a 2-dram vial followed by addition of a solution of 4-(propa-1,2-dien-1-yl-*d*3)-1,1'-biphenyl (39.0 mg, 0.200 mmol, 1.00 eq.), THF (0.400 mL), and 2-propanol-d (91 µL, 1.20 mmol, 4 eq.). The 2-dram vial was capped with a pressure relief cap, and the reaction stirred for 40 h at 40 ˚C. Upon completion, the crude product was dry loaded onto silica gel and isolated by flash column chromatograph (250 mL of 100% Petroleum ether) to give the pure product as colorless oil (***d*₅-4d**, 34.1 mg, 0.1893 mmol, 96% deuterium incorporation at benzylic position, 95% deuterium incorporation at terminal position, 95% isolated yield).

^{1}H NMR (500 MHz, $CDCl_3$) δ 7.60 (d, *J* = 7.2 Hz, 2H), 7.53 (d, *J* = 8.2 Hz, 2H), 7.44 (t, *J* = 7.5 Hz, 2H), 7.33 (t, *J* = 7.4 Hz, 1H), 7.26 (d, *J* = 8.2 Hz, 2H), 2.65 – 2.59 (m, 0.09H), 1.66 (s, 2H), 0.98 – 0.93 (m, 0.16H).

^{2}H NMR (77 MHz, $CHCl_3$) δ 2.56 (s, 2D), 0.89 (s, 3D).

^{13}C NMR (126 MHz, $CDCl_3$) δ 142.0, 141.4, 138.7, 129.0, 128.8, 127.14, 127.11, 127.09, 37.1 (p, *J* = 19.4 Hz), 24.3, 13.08 (hept, *J* = 19.3 Hz).

HRMS: (ESI$^+$) *m/z* $[M+H]^+$ calculated for $C_{15}H_{12}D_5^+$ 202.1639; found 202.1644

Spectroscopic data were consistent with literature reported d_1-isotopolog and d_2-isotopolog.[15], [16]

4-(propyl-*d*₇)-1,1'-biphenyl [*d*₇-4d]

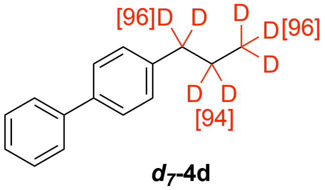

***d*₇-4d**

According to the general procedure, DTB-DPPBz (19.7 mg, 0.220 mmol), $Cu(OAc)_2$ (200 µL of a 0.100 M solution in THF), DMMS-D (107.2 mg, 1.00 mmol, 6.00 eq), and THF (0.300 mL) were combined in a 2-dram vial followed by addition of a solution of 4-(propa-1,2-dien-1-yl-*d*3)-1,1'-biphenyl (39.0 mg, 0.200 mmol, 1.00 eq.), THF (0.400 mL), and 2-propanol-d (91 µL, 1.20 mmol, 4 eq.). The 2 dram vial was capped with a pressure relief cap, and the reaction stirred for 40 h at 40 ˚C. Upon completion, the crude product was dry loaded onto silica gel and isolated by flash column chromatograph (250 mL of 100% Petroleum ether)

to give the pure product as colorless oil (***d_7*-4d**, 17.1 mg, 0.0841mmol, 96% deuterium incorporation at benzylic position, 94% deuterium incorporation at homobenzylic position, 96% deuterium incorporation at terminal position, 42% isolated yield).

^{1}H NMR (500 MHz, $CDCl_3$) δ 7.60 (d, *J* = 7.8 Hz, 2H), 7.53 (d, *J* = 8.2 Hz, 2H), 7.44 (t, *J* = 7.6 Hz, 2H), 7.34 (t, *J* = 7.9 Hz, 1H), 7.27 (d, *J* = 8.1 Hz, 2H), 2.61 (s, 0.08H), 1.65 (s, 0.12H), 0.94 (s, 0.12H).

^{2}H NMR (77 MHz, $CHCl_3$) δ 2.61 (s, 2D), 1.64 (s, 2D), 0.94 (s, 3D).

^{13}C NMR (126 MHz, $CDCl_3$) δ 142.0, 141.4, 138.7, 129.0, 128.8, 127.14, 127.11, 127.08, 36.8 (p, *J* = 19.2 Hz), 23.5 (p, *J* = 24.8 Hz), 12.9 (hept, *J* = 18.6 Hz).

LRMS: (ESI^+) *m/z* $[M]^+$ calculated for $C_{15}H_9D_7^+$ 203.17; found 203.18

Note: The accurate HRMS data for this compound could not be obtained due to an ionization issue with Waters Synapt G2-Si QTOF. The NMR spectroscopic data were consistent with literature reported values of d_1-4-(propyl)-1,1'-biphenyl and 4-(propyl-2,3-d_2)-1,1'-biphenyl isotopomer. [15], [16]

E. Chirality Transfer by CuH-Catalyzed Semi-Reductive Deuteration of Enantioenriched Allenes.

General Procedure for Asymmetric CuH-Catalyzed Transfer Hydrodeuteration of Allenes

Preparation of *d_0*-but-2-en-1-ylbenzene standard for MRR study

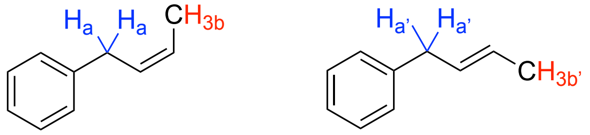

(*Z*)-***d_0*-2a** (*E*)-***d_0*-2a**

Z/E ~ 12:1

2 mmol scale reaction for MRR analysis: In a N_2 filled glovebox, DTB-DPPBz (39.39 mg, 0.0440 mmol, 0.0220 eq.), $Cu(OAc)_2$ (400 *μL* of a 0.100 M solution in THF, 0.0400 mmol, 0.0200 eq.), and THF (1.6 mL) were added to a 20 mL vial equipped with a Teflon® PTE coated stir bar followed by dropwise addition of DEMS (961 μ*L*, 6.00 mmol, 3.00 eq.). A color change from green/blue to yellow was observed while stirring for 5-10 minutes. In a separate 2-dram vial was added buta-1,2-dien-1-ylbenzene **1a** (317.5 mg, 2.000 mmol, 1.00 eq., 82 wt% in hexane), THF (2.0 mL), and 2-propanol-*d* (612 μ*L*, 8.00 mmol, 4.00 eq.). The solution in the 2-dram vial was added dropwise over 20 seconds to the 20 mL vial. The total volume of THF was calculated based on having a final reaction concentration of 0.5 M based on the allene substrate. The 2-dram vial was capped, taken out of the glovebox, and stirred for 20 h at room temperature, at which point the reaction mixture was transferred to a 100 mL round bottom flask using 20 mL of diethyl ether. After removing the solvent by rotary evaporation, the crude product was dry loaded onto silica gel and isolated by Biotage® Isolera™ One Flash Chromatography System using prepacked Biotage® Sfär HC Duo cartridges 25g (12 CV 100% hexane), target fractions were combined in a 500 mL round bottom flask using diethyl ether. The final product was obtained in solvent mixture (hexane and diethyl ether). Due to the volatility of this compound, an aliquot of the concentrated sample was dissolved in ca. 1.2 mL $CDCl_3$ for NMR study. According to the literature reported data, the *Z* and *E* isomers can be differentiated by corresponding *J* couplings, reported *J* = 4.7 Hz for H_a and *J* = 4.5 Hz for H_b in *Z* isomer, reported *J* = 6.1 Hz for $H_{a'}$ and *J* = 6.0 Hz for $H_{b'}$ in *E* isomer.[1]

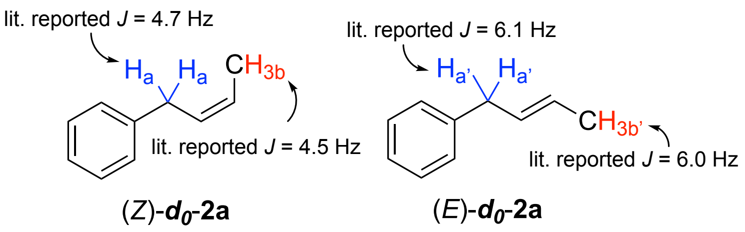

Ref: *Angew. Chem. Int. Ed.* **2011**, *50*, 9602 –9606

The corresponding *J* couplings obtained from the isolated material were *J* = 4.8 Hz for H_a and *J* = 4.8 Hz for H_b, which the major isomer was assigned as *Z* isomer. The reported *J* = 6.1 Hz for $H_{a'}$ and *J* = 6.1 for $H_{b'}$, which the minor isomer was assigned as *E* isomer. The ratio of *Z/E* was defined by the integration of the allylic/benzylic position, ratio = 2.01/0.17 = 11.8 This d_0-compound was used as the reference for MRR to obtain the broadband spectrum.

Figure S3. Z/E ratio determination for d_0 -but-2-en-1-ylbenzene

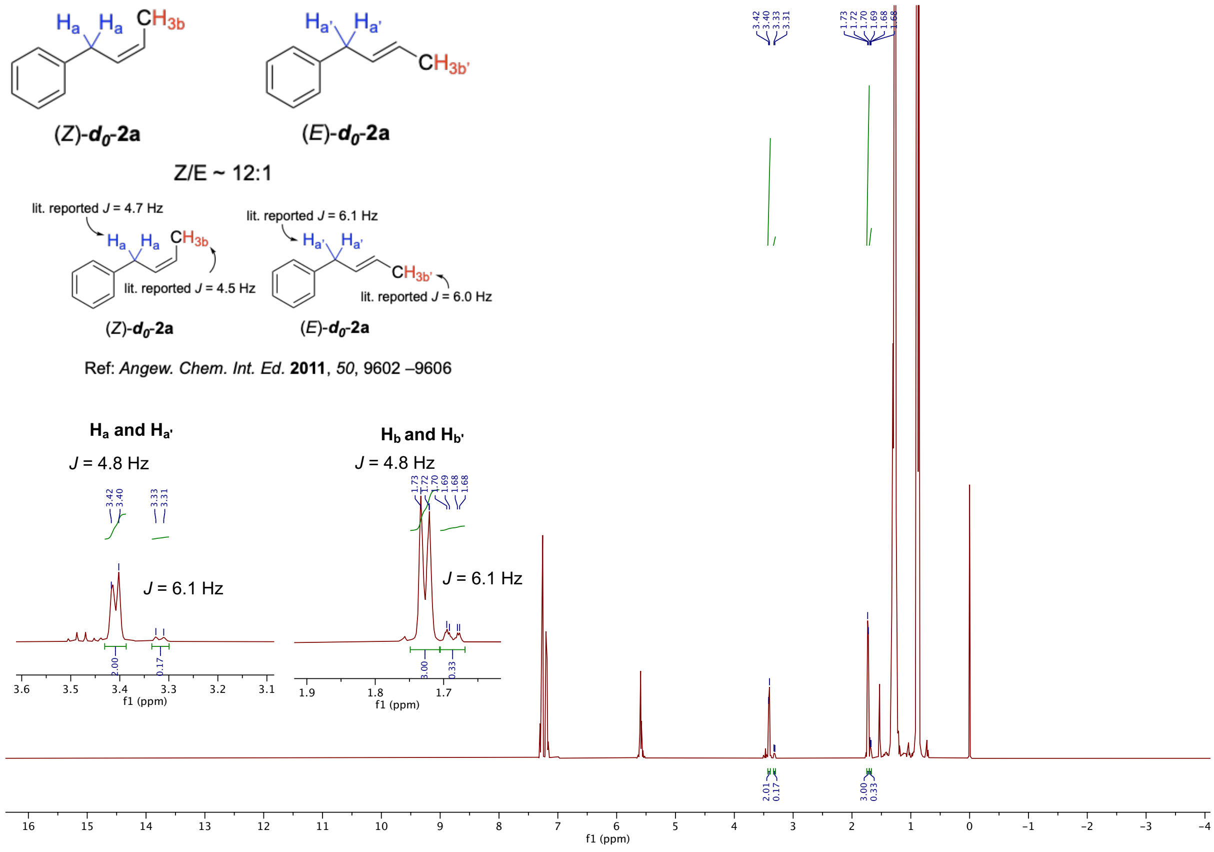

Figure S4. Chirality transfer by Cu-H catalyzed Semi-reductive deuteration of enantioenriched allenes

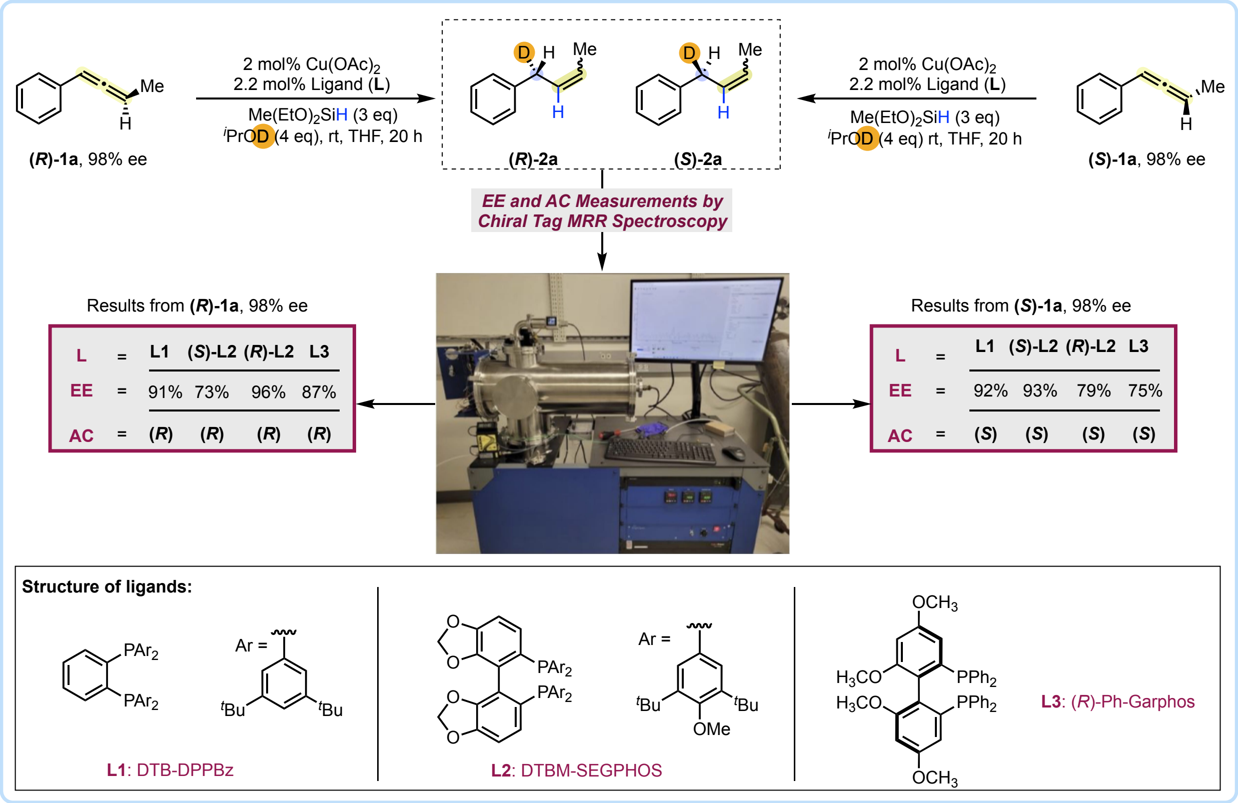

1 mmol scale reaction for MRR analysis: In a N_2 filled glovebox, ligand (0.0220 eq.), $Cu(OAc)_2$ (200 μL of a 0.100 M solution in THF), and THF (0.80 mL) were added to a 4-dram vial equipped with a Teflon® PTE coated stir bar followed by dropwise addition of diethoxy(methyl)silane (481 μL, 3.000 mmol, 3.00 eq.). A color change from green/blue to yellow was observed while stirring for 5-10 minutes. In a separate 2-dram vial was added the (*R*)-**s1a** or (*S*)-**s1a** (130.2 mg, 1.000 mmol, 1.00 eq.), THF (1.0 mL), and 2-propanol-*d* (306 μL, 4.000 mmol, 4.00 eq.). The solution in the 2-dram vial was added dropwise over 20 seconds to the 2-dram vial. The total volume of THF was calculated based on having a final reaction concentration of 0.5 M based on the allene substrate. The 4-dram vial was capped, taken out of the glovebox, and stirred for the respective time at the appropriate temperature, at which point the reaction mixture was concentrated to ca. 0.2 mL volume, ee was analyzed by MRR. An aliquot of sample was dissolved into $CDCl_3$ (ca. 0.8 mL) and analyzed by ^1H NMR for the deuterium incorporation.

Entry 1. According to the general procedure, DTB-DPPBz (19.7 mg, 0.022 mmol), $Cu(OAc)_2$ (200 μL of a 0.100 M solution in THF), diethoxy(methyl)silane (481 μL, 3.00 mmol, 3.00 eq), and THF (0.8 mL) were combined in a 1-dram vial followed by addition of a solution of (*R*)-buta-1,2-dien-1-ylbenzene (203.4 mg, 64% wt in Hexane, 1.00 mmol, 1.00 eq.), THF (1.00 mL), and 2-propanol-*d* (306 μL, 4.00 mmol, 4.00 eq.). The 2-dram vial was capped with a pressure relief cap, and the reaction stirred for 20 h at room temperature. Upon completion, the ^1H NMR indicated 91% deuterium incorporation, *Z/E* = 12.1/1. Product analyzed by the Broadband instrument as 91% ee, see MRR SI for characterization details.

Entry 2. According to the general procedure, (S)-DTBM-SEGPHOS (26.0 mg, 0.022 mmol), $Cu(OAc)_2$ (200 μL of a 0.100 M solution in THF), diethoxy(methyl)silane (481 μL, 3.00 mmol, 3.00 eq), and THF (0.8 mL) were combined in a 1-dram vial followed by addition of a solution of (*R*)-buta-1,2-dien-1-ylbenzene (203.4 mg, 64% wt in Hexane, 1.00 mmol, 1.00 eq.), THF (1.00 mL), and 2-propanol-*d* (306 μL, 4.00 mmol, 4.00 eq.). The 2-dram vial was capped with a pressure relief cap, and the reaction stirred for 20 h at room

temperature. Upon completion, the ^{1}H NMR indicated 92% deuterium incorporation, *Z/E* = 13.5/1. Product analyzed by the Broadband instrument as 73% ee, see MRR SI for characterization details.

Entry 3. According to the general procedure, (*R*)-DTBM-SEGPHOS (26.0 mg, 0.022 mmol), $Cu(OAc)_2$ (200 µL of a 0.100 M solution in THF), diethoxy(methyl)silane (481 µL, 3.00 mmol, 3.00 eq), and THF (0.8 mL) were combined in a 1-dram vial followed by addition of a solution of (*R*)-buta-1,2-dien-1-ylbenzene (203.4 mg, 64% wt in Hexane, 1.00 mmol, 1.00 eq.), THF (1.00 mL), and 2-propanol-*d* (306 µL, 4.00 mmol, 4.00 eq.). The 2-dram vial was capped with a pressure relief cap, and the reaction stirred for 20 h at room temperature. Upon completion, the ^{1}H NMR indicated 99% deuterium incorporation, *Z/E* = 9.7/1. Product analyzed by the Broadband instrument as 96% ee, see MRR SI for characterization details.

Entry 4. According to the general procedure, (*R*)-Ph-Garphos (25.4 mg, 0.022 mmol), $Cu(OAc)_2$ (200 µL of a 0.100 M solution in THF), diethoxy(methyl)silane (481 µL, 3.00 mmol, 3.00 eq), and THF (0.8 mL) were combined in a 1-dram vial followed by addition of a solution of (*R*)-buta-1,2-dien-1-ylbenzene (203.4 mg, 64% wt in Hexane, 1.00 mmol, 1.00 eq.), THF (1.00 mL), and 2-propanol-*d* (306 µL, 4.00 mmol, 4.00 eq.). The 2-dram vial was capped with a pressure relief cap, and the reaction stirred for 20 h at room temperature. Upon completion, the ^{1}H NMR indicated 93% deuterium incorporation, *Z/E* = 10.7/1. Product analyzed by the Broadband instrument as 87% ee, see MRR SI for characterization details.

Entry 5. According to the general procedure, DTB-DPPBz (19.7 mg, 0.022 mmol), $Cu(OAc)_2$ (200 µL of a 0.100 M solution in THF), diethoxy(methyl)silane (481 µL, 3.00 mmol, 3.00 eq), and THF (0.8 mL) were combined in a 1-dram vial followed by addition of a solution of (*S*)-buta-1,2-dien-1-ylbenzene (130.2 mg, 1.00 mmol, 1.00 eq.), THF (1.00 mL), and 2-propanol-*d* (306 µL, 4.00 mmol, 4.00 eq.). The 2-dram vial was capped with a pressure relief cap, and the reaction stirred for 20 h at room temperature. Upon completion, the ^{1}H NMR indicated 98% deuterium incorporation, *Z/E* = 12.7/1. Product analyzed by the Broadband instrument as 92% ee, see MRR SI for characterization details.

Entry 6. According to the general procedure, (*S*)-DTBM-SEGPHOS (26.0 mg, 0.022 mmol), $Cu(OAc)_2$ (200 µL of a 0.100 M solution in THF), diethoxy(methyl)silane (481 µL, 3.00 mmol, 3.00 eq), and THF (0.8 mL) were combined in a 1-dram vial followed by addition of a solution of (*S*)-buta-1,2-dien-1-ylbenzene (130.2 mg, 1.000 mmol, 1.00 eq.), THF (1.00 mL), and 2-propanol-*d* (306 µL, 4.00 mmol, 4.00 eq.). The 2-dram vial was capped with a pressure relief cap, and the reaction stirred for 20 h at room temperature. Upon completion, the ^{1}H NMR indicated 98% deuterium incorporation, *Z/E* = 10.2/1. Product analyzed by the Broadband instrument as 93% ee, see MRR SI for characterization details.

Entry 7. According to the general procedure, (*R*)-DTBM-SEGPHOS (26.0 mg, 0.022 mmol), $Cu(OAc)_2$ (200 µL of a 0.100 M solution in THF), diethoxy(methyl)silane (481 µL, 3.00 mmol, 3.00 eq), and THF (0.8 mL) were combined in a 1-dram vial followed by addition of a solution of (*S*)-buta-1,2-dien-1-ylbenzene (130.2 mg, 1.00 mmol, 1.00 eq.), THF (1.00 mL), and 2-propanol-*d* (306 µL, 4.00 mmol, 4.00 eq.). The 2-dram vial was capped with a pressure relief cap, and the reaction stirred for 20 h at room temperature. Upon completion, the ^{1}H NMR indicated 99% deuterium incorporation, *Z/E* > 20/1. Product analyzed by the Broadband instrument as 79% ee, see MRR SI for characterization details.

Entry 8. According to the general procedure, (*R*)-Ph-Garphos (25.4 mg, 0.022 mmol), $Cu(OAc)_2$ (200 µL of a 0.100 M solution in THF), diethoxy(methyl)silane (481 µL, 3.00 mmol, 3.00 eq), and THF (0.8 mL) were combined in a 1-dram vial followed by addition of a solution of (*S*)-buta-1,2-dien-1-ylbenzene (130.2 mg, 1.00 mmol, 1.00 eq.), THF (1.00 mL), and 2-propanol-*d* (306 µL, 4.00 mmol, 4.00 eq.). The 2-dram vial was capped with a pressure relief cap, and the reaction stirred for 20 h at room temperature. Upon completion, the ^{1}H NMR indicated 97% deuterium incorporation, *Z/E* = 14.7/1. Product analyzed by the Broadband instrument as 75% ee, see MRR SI for characterization details.

F. Synthesis of Allene Starting Materials

Buta-1,2-dien-1-ylbenzene [1a]:

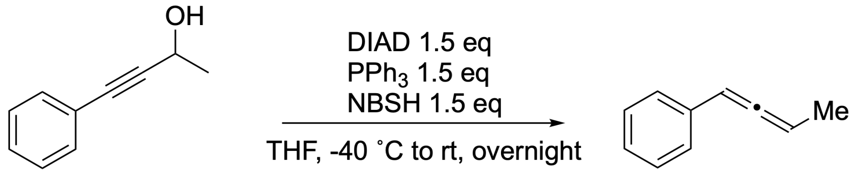

To an oven dried 200 mL round bottom flask equipped with a Teflon coated stir bar, was charged PPh_3 (5.38 g, 20.5 mmol, 1.50 eq) and anhydrous THF (54 mL). DIAD (4.03 mL, 20.5 mmol, 1.50 eq) was slowly added to the solution of PPh_3 at -40 ˚C. After 10 minutes, a solution of 4-phenylbut-3-yn-2-ol (2.00g, 13.7 mmol, 1.00 eq) in anhydrous THF (20 mL) was added to the yellow mixture over 10 minutes *via* a syringe. After 30 minutes, a solution of 2-nitrobenzenesulfonohydrazide (4.46 g, 20.5 mmol, 1.5 eq) in anhydrous THF (50 mL) was added to the reaction mixture *via* a dropping funnel over 30 minutes. The result orange solution was warmed to room temperature over 2 hours and then allowed to stand for overnight. Upon completion (judged by TLC), volatiles were carefully removed by the aid of rotary evaporation to afford crude material which was dry loaded onto an empty 25g Sfär cartridge, isolated by Biotage® Isolera™ One Flash Chromatography System with 5 CV of 100% hexane gradient with 100 g Sfär HC cartridge to obtain product as clear colorless liquid (520 mg, 29% isolated yield).

1H NMR (500 MHz, $CDCl_3$) δ 7.34 – 7.27 (m, 4H), 7.22 – 7.15 (m, 1H), 6.13 – 6.07 (m, 1H), 5.54 (p, *J* = 7.1 Hz, 1H), 1.80 (dd, *J* = 7.2, 3.2 Hz, 3H).
^{13}C NMR (126 MHz, $CDCl_3$) δ 206.2, 135.2, 128.7, 126.8, 94.1, 89.7, 14.2. one carbon signal missing is due to overlap in the aromatic range.

Spectroscopic data were consistent with literature reported values. [17]

Note: The reaction reproducibility is highly related to the water content of NBSH, it is necessary to recrystallize and vacuum-dry NBSH before using. This allene should be under nitrogen in -10 ˚C freezer due to the instability, it is also necessary to repurify the allene by passing through a 2-inch silica pad with petroleum ether before use for transfer hydrodeuteration reaction. This allene can be also isolated by vacuum distillation in larger scale, bp 60 – 62 ˚C, 1 mmHg.

(4-Methylpenta-1,2-dien-1-yl)benzene [1b]:

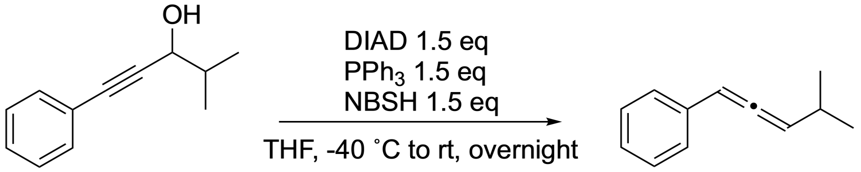

To an oven dried 100 mL round bottom flask equipped with a Teflon coated stir bar, was charged PPh_3 (790 mg, 3.0 mmol, 1.50 eq) and anhydrous THF (8 mL). DIAD (0.59 mL, 3.0 mmol, 1.50 eq) was slowly added to the solution of PPh_3 at -40 ˚C. After 10 minutes, a solution of 4-methyl-1-phenylpent-1-yn-3-ol (350 mg, 2.0 mmol, 1.00 eq) in anhydrous THF (3.7 mL) was added to the yellow mixture over 10 minutes *via* a syringe. 30 minutes later, a solution of 2-nitrobenzenesulfonohydrazide (654 mg, 3.0 mmol, 1.5 eq) in anhydrous THF (8 mL) was added to the reaction mixture *via* a dropping funnel over 30 minutes. The result orange solution was warmed to room temperature over 2 hours and then allowed to stand for overnight. Upon completion (judged by TLC), volatiles were removed by the aid of rotary evaporation to afford crude material, which was dry loaded onto an empty 25g Sfär cartridge, isolated by Biotage® Isolera™ One Flash Chromatography System with 5 CV of 100% hexane gradient with 100 g Sfär HC cartridge to obtain product as clear colorless liquid (171 mg, 54% isolated yield).

^{1}H NMR (400 MHz, CDCl$_3$) δ 7.30 (d, *J* = 4.4 Hz, 4H), 7.22 – 7.14 (m, 1H), 6.18 (dd, *J* = 6.4, 3.0 Hz, 1H), 5.60 (q, *J* = 6.1 Hz, 1H), 2.52 – 2.37 (m, 1H), 1.10 (dd, *J* = 6.8, 3.1 Hz, 6H).

^{13}C NMR (101 MHz, CDCl$_3$) δ 203.8, 135.4, 128.7, 126.8, 126.6, 102.6, 95.8, 28.6, 22.8, 22.7.

Spectroscopic data were consistent with literature reported values. [18]

Note: The reaction reproducibility highly depends on the water content and purity of NBSH, it is necessary to recrystallize and vacuum-dried NBSH before the reaction. This allene should be stored under nitrogen in -10 ˚C freezer due to the instability, it is also necessary to repurify the allene by passing through a 2-inch silica pad with petroleum ether before use for transfer hydrodeuteration reaction.

(*R*)-buta-1,2-dien-1-ylbenzene[(*R*)-1a]:

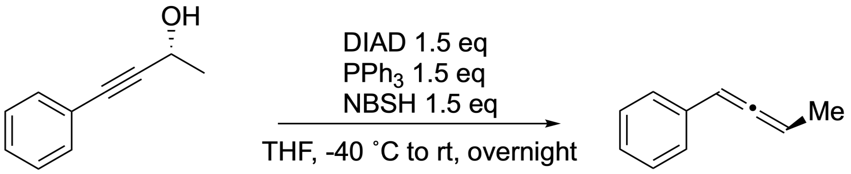

To an oven dried 200 mL round bottom flask equipped with a Teflon coated stir bar, was charged PPh$_3$ (5.38 g, 20.5 mmol, 1.50 eq) and anhydrous THF (54 mL). DIAD (4.03 mL, 20.5 mmol, 1.50 eq) was slowly added to the solution of PPh$_3$ at -40 ˚C. After 10 minutes, a solution of (*R*)-4-phenylbut-3-yn-2-ol (>98% ee, 2.00g, 13.7 mmol, 1.00 eq) in anhydrous THF (20 mL) was added to the yellow mixture over 10 minutes *via* a syringe. 30 minutes later, a solution of 2-nitrobenzenesulfonohydrazide (4.46 g, 20.5 mmol, 1.5 eq) in anhydrous THF (45 mL) was added to the reaction mixture *via* a dropping funnel over 30 minutes. The result orange solution was warmed to room temperature over 2 hours and then allowed to stand for overnight. Upon completion (judged by TLC), volatiles were removed by the aid of rotary evaporation to afford crude material, which was dry loaded onto an empty 25g Sfär cartridge, isolated by Biotage® Isolera^{TM} One Flash Chromatography System with 5 CV of 100% hexane gradient with 100 g Sfär HC cartridge to obtain product as clear colorless liquid (707 mg, 64 wt% in Hexane, 25% isolated yield, 98% ee).

^{1}H NMR (400 MHz, CDCl$_3$) δ 7.33 – 7.27 (m, 4H), 7.23 – 7.15 (m, 1H), 6.15 – 6.04 (m, 1H), 5.54 (p, *J* = 7.3 Hz, 1H), 1.79 (dd, *J* = 7.1, 3.2 Hz, 3H).

Spectroscopic data was consistent with literature reported values. [17]

HPLC: Sampled as 5 mg in 2 mL Heptane, Chiralcel® OJ-3 (3 µm, 4.6 mm X 250 mm). Molbile phase: 100% Hexane 1mL/min. Detection wavelength: 210 nm. ee: 98%

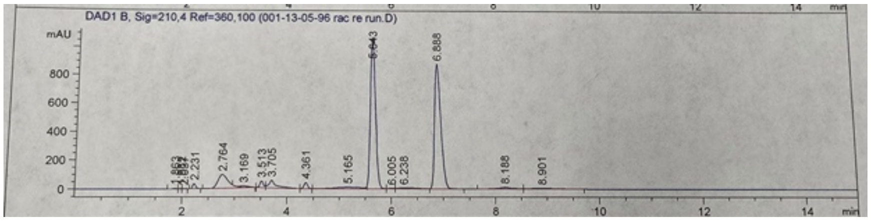

Peak	Ret. Time	Area%
1#	5.643	49.7183%
2#	6.888	50.2816%
Total		100.000%

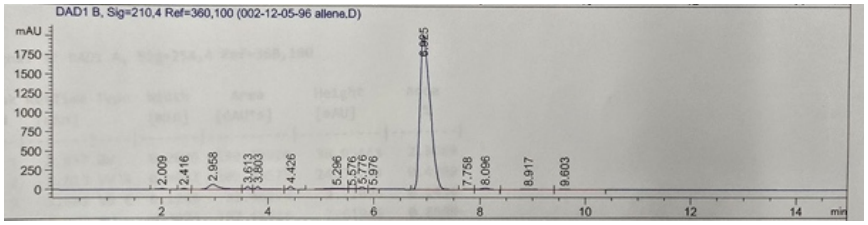

Peak	Ret. Time	Area%
1#	5.776	0.631683%
2#	6.925	99.3359%
Total		100.000%

<u>Note:</u> This allene was stored under nitrogen in -10 ˚C freezer due to the instability, it is also necessary to repurify the allene by passing through a 2-inch silica pad with petroleum ether before using for asymmetric transfer hydrodeuteration reaction.

(*S*)-buta-1,2-dien-1-ylbenzene[(*S*)-1a]:

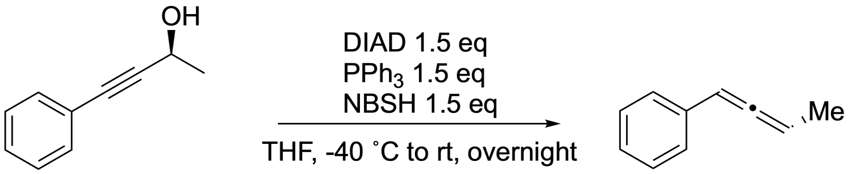

To an oven dried 200 mL round bottom flask equipped with a Teflon coated stir bar, was charged PPh_3 (5.38 g, 20.5 mmol, 1.50 eq) and anhydrous THF (54 mL). DIAD (4.03 mL, 20.5 mmol, 1.50 eq) was slowly added to the solution of PPh_3 at –40 ˚C. After 10 minutes, a solution of (*S*)-4-phenylbut-3-yn-2-ol (>98% ee, 2.00 g, 13.7 mmol, 1.00 eq) in anhydrous THF (20 mL) was added to the yellow mixture over 10 minutes *via* a syringe. 30 minutes later, a solution of 2-nitrobenzenesulfonohydrazide (NBSH, 4.46 g, 20.5 mmol, 1.5 eq) in anhydrous THF (45 mL) was added to the reaction mixture *via* a dropping funnel over 30 minutes. The result orange solution was warmed to room temperature over 2 hours and then allowed to stand for overnight. Upon completion (judged by TLC), volatiles were removed by the aid of rotary evaporation to afford crude material, which was dry loaded onto an empty 25g Sfär cartridge, isolated by Biotage® Isolera™ One Flash Chromatography System with 5 CV of 100% hexane gradient with 100 g Sfär HC cartridge to obtain product as clear colorless liquid (800 mg, 45% isolated yield, 98% ee).

<u>^{1}H NMR (400 MHz, $CDCl_3$)</u> δ 7.35 – 7.27 (m, 4H), 7.24 – 7.16 (m, 1H), 6.16 – 6.07 (m, 1H), 5.55 (p, *J* = 7.0 Hz, 1H), 1.81 (dd, *J* = 7.1, 3.2 Hz, 3H).

Spectroscopic data was consistent with literature reported values. [17]

HPLC: Sampled as 5 mg in 2 mL Heptane, Chiralcel® OJ-3 (3 µm, 4.6 mm X 250 mm). Mobile phase: 100% Hexane 1mL/min. Detection wavelength: 210 nm. ee: 98%

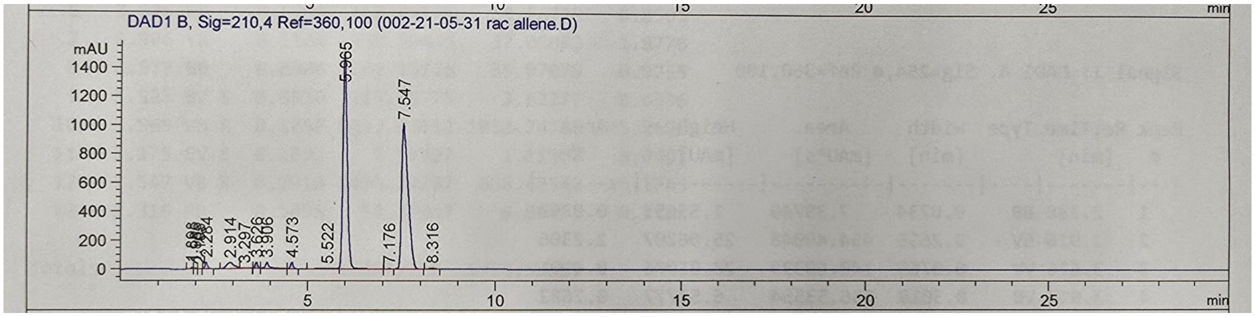

Peak	Ret. Time	Area%
1#	5.965	49.9810%
2#	7.547	50.0190%
Total		100.000%

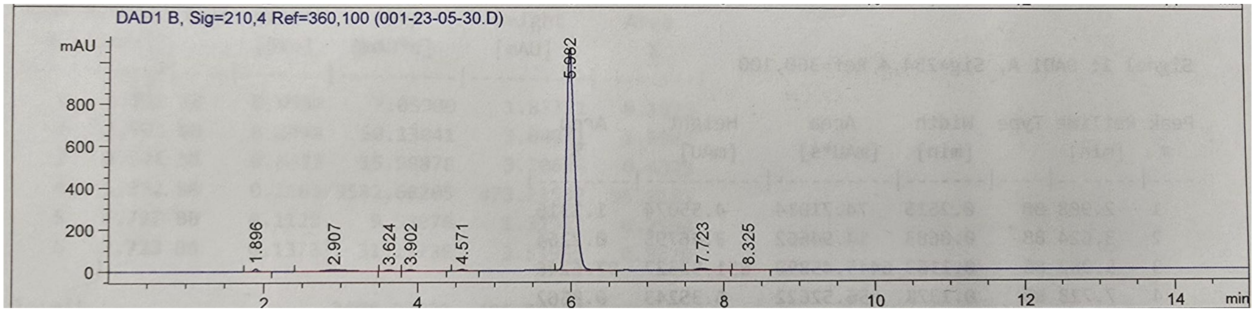

Peak	Ret. Time	Area%
1#	5.982	99.1069%
2#	7.723	0.893136%
Total		100.000%

<u>Note:</u> This allene should be stored under nitrogen in -10 ˚C freezer due to the instability, it is also necessary to repurify the allene by passing through a 2-inch silica pad with petroleum ether before using for asymmetric transfer hydrodeuteration reaction.

(3-methylpenta-1,2-dien-1-yl)benzene [1c]:

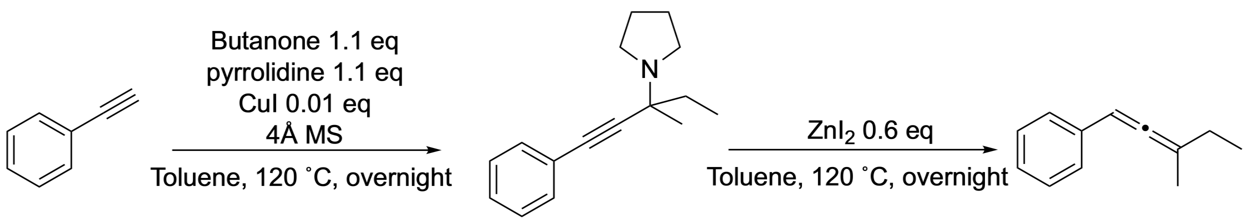

To an oven-dried 50 mL Schlenk tube, was charged phenylacetylene (1.1 mL, 10 mmol, 1.0 eq), butanone (1.0 mL, 11 mmol, 1.1 eq), pyrrolidine (0.9 mL, 11 mol, 1.1 eq), toluene (10 mL), freshly activated 4Å MS (3g), and CuI (29 mg, 0.01 mmol, 0.01). The Schlenk tube was sealed with a rubber septum, then the mixture was heated to 120˚C by oil bath. After 12 h, the mixture was filtered through a silica gel plug followed by acetone (50 mL), the solvent was removed by the aid of rotary evaporation. The residue was transferred to an oven-dried 50 mL Schlenk flask containing ZnI_2 (1.92g, 0.6 mmol, 0.6 eq) and equipped with a Teflon coated stir bar, toluene (10 mL) was added *via* a syringe. The mixture was heated to 120˚C for 12 h, the result slurry was filtered through a Celite pad. After remove solvents from the filtrate, the crude material was dry loaded to silica gel, which was further purified by flash column chromatography with 1000 mL 100% hexane as eluent to obtain product as colorless oil (351 mg, 22% isolated yield).

<u>^{1}H NMR (400 MHz, $CDCl_3$)</u> δ 7.33 – 7.26 (m, 4H), 7.21 – 7.11 (m, 1H), 6.14 – 6.04 (m, 1H), 2.17 – 2.04 (m, 2H), 1.82 (d, *J* = 2.2 Hz, 3H), 1.06 (t, *J* = 7.4 Hz, 3H).

<u>^{13}C NMR (101 MHz, $CDCl_3$)</u> δ 202.5, 136.3, 128.6, 126.6, 126.5, 105.7, 94.6, 27.3, 18.9, 12.4.

Spectroscopic data were consistent with literature reported values. [19]

butyl-4-(3-methylpenta-1,2-dien-1-yl)benzene [1d]:

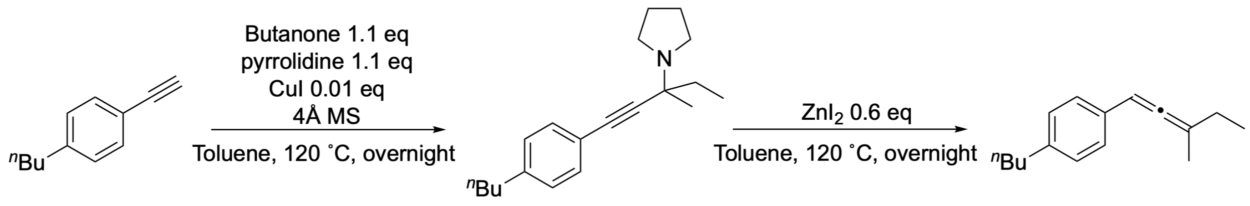

To an oven-dried 50 mL Schlenk tube, was charged 1-butyl-4-ethynylbenzene (1g, 6.3 mmol, 1.0 eq), butanone (500 mg, 6.95 mmol, 1.1 eq), pyrrolidine (494 mg, 6.95 mol, 1.1 eq), toluene (6.5 mL), freshly activated 4Å MS (1.5 g), and CuBr (14 mg, 0.01 mmol, 0.01). The Schlenk tube was sealed with a rubber septum, then the mixture was heated to 120˚C by oil bath. After 12 h, the mixture was filtered through a silica gel plug followed by acetone (25 mL), the solvent was removed by the aid of rotary evaporation. The residue was transferred to an oven-dried 50 mL Schlenk flask containing ZnI_2 (1.92g, 0.6 mmol, 0.6 eq) and equipped with a Teflon coated stir bar, toluene (6.5 mL) was added *via* a syringe. The mixture was heated to 120˚C for 12 h, the result slurry was filtered through a Celite pad. After removing solvents from the filtrate, the crude material was dry loaded to silica gel, which was further purified by flash column chromatography with 1000 mL 100% hexane as eluent to obtain product as colorless oil (528 mg, 39% isolated yield).

<u>^{1}H NMR (400 MHz, $CDCl_3$)</u> δ 7.18 (d, *J* = 7.5 Hz, 2H), 7.11 (d, *J* = 8.0 Hz, 2H), 6.07 (q, *J* = 3.2 Hz, 1H), 2.58 (t, *J* = 7.7 Hz, 2H), 2.15 – 2.00 (m, 2H), 1.81 (d, *J* = 2.3 Hz, 3H), 1.65 – 1.51 (m, 2H), 1.35 (sext, *J* = 7.5 Hz, 2H), 1.06 (t, *J* = 7.4 Hz, 3H), 0.92 (t, *J* = 7.4 Hz, 3H).

<u>^{13}C NMR (101 MHz, $CDCl_3$)</u> δ 202.1, 141.3, 133.5, 128.7, 126.5, 105.5, 94.4, 35.5, 33.9, 27.4, 22.5, 19.0, 14.1, 12.5.

<u>HRMS:</u> (ESI^+) *m/z* $[M+H]^+$ calculated for $C_{16}H_{23}^+$ 215.1794; found 215.1795

4-(3-methylpenta-1,2-dien-1-yl)-1,1'-biphenyl [1e]:

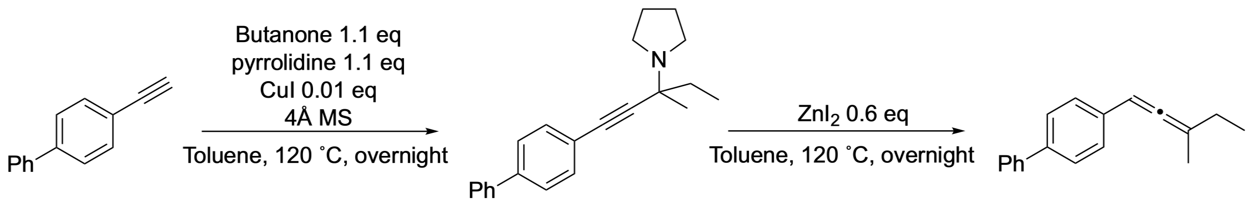

To an oven-dried 50 mL Schlenk tube, was charged 1-butyl-4-ethynylbenzene (1g, 5.61 mmol, 1.0 eq), butanone (0.60 mL, 8.25 mmol, 1.47 eq), pyrrolidine (0.6 mL, 8.25 mol, 1.47 eq), toluene (6.5 mL), freshly activated 4Å MS (1.5 g), and CuBr (22 mg, 0.01 mmol, 0.02 eq). The Schlenk tube was sealed with a rubber septum, then the mixture was heated to 120˚C by oil bath. After 12 h, the mixture was filtered through a silica gel plug followed by acetone (25 mL), the solvent was removed by the aid of rotary evaporation. The residue was transferred to an oven-dried 50 mL Schlenk flask containing ZnI_2 (1.07 g, 3.37 mmol, 0.6 eq) and equipped with a Teflon coated stir bar, toluene (6.5 mL) was added *via* a syringe. The mixture was heated to 120˚C for 12 h, the result slurry was filtered through a Celite pad. After remove solvents from the filtrate, the crude material was dry loaded to silica gel, which was further purified by flash column chromatography with 1000 mL 100% hexane as eluent to obtain product as white solid (484 mg, 37% isolated yield).

^{1}H NMR (400 MHz, CDCl$_3$) δ 7.61 – 7.57 (m, 2H), 7.53 (d, J = 8.2 Hz, 2H), 7.43 (t, J = 7.8 Hz, 2H), 7.37 – 7.30 (m, 3H), 6.16 – 6.11 (m, 1H), 2.17 – 2.06 (m, 2H), 1.84 (d, J = 2.8 Hz, 3H), 1.08 (t, J = 7.4 Hz, 3H).

^{13}C NMR (126 MHz, CDCl$_3$) δ 202.8, 141.2, 139.4, 135.4, 128.9, 127.4, 127.2, 127.1, 127.0, 105.8, 94.3, 27.4, 18.9, 12.5.
HRMS: (ESI) m/z: $[M+H]^+$ calculated for $C_{18}H_{19}^+$ 235.1481; found 235.1478.

octa-1,2-dien-1-ylbenzene [1f]:

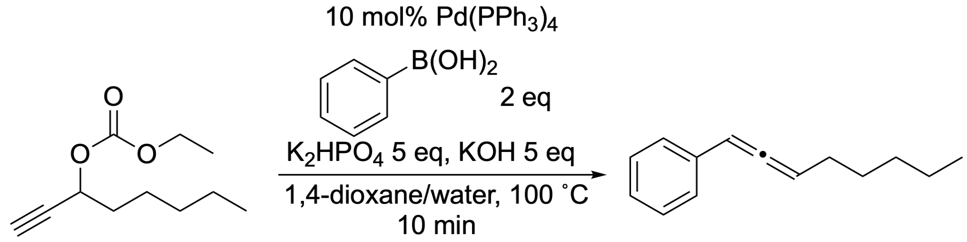

To a 50 mL round-bottom flask equipped with a Teflon coated stir bar, was charged K_2HPO_4 (4.35 g, 25 mmol, 5.0 eq), DI water (16 mL), KOH (1.40g, 25 mmol, 5.0 eq), ethyl oct-1-yn-3-yl carbonate (991 mg, 5.0 mmol, 1.0 eq), phenylboronic acid (1.22 g, 10 mmol, 2.0 eq), and dioxane (32 mL). The mixture was rapidly heated to 100 ˚C, and then $Pd(PPh_3)_4$ (578 mg, 0.5 mmol, 10 mol%) was added in one portion. The flask was sealed with a rubber sealed, and then the mixture was kept at 100 ˚C for 10 min. At which time, the reaction mixture was vacuum-filtered through a silica pad which was washed with 50 mL diethyl ether. The solvents were removed by the aid of rotary evaporation from the combined filtrate to afford the crude material, which was dry loaded onto silica gel, the product was isolated by flash column chromatography with 500 mL 100% petroleum ether as eluent to obtain product as colorless oil (146 mg, 16% isolated yield)

^{1}H NMR (400 MHz, CDCl$_3$) δ 7.37 – 7.27 (m, 4H), 7.23 – 7.13 (m, 1H), 6.18 – 6.08 (m, 1H), 5.57 (q, J = 6.7 Hz, 1H), 2.18 – 2.08 (m, 2H), 1.55 – 1.44 (m, 2H), 1.38 – 1.31 (m, 4H), 0.90 (t, J = 7.1 Hz, 3H).

Spectroscopic data was consistent with literature reported values. [20]

penta-1,2-diene-1,5-diyldibenzene [1g]:

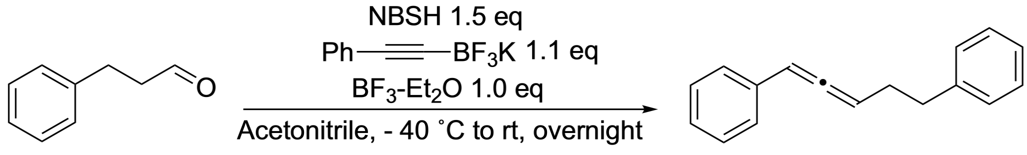

To a flame dried round bottom flask equipped with a Teflon coated stir bar, 2-Nitrobenzenesulfonylhydrazide (2.43 g, 11.18 mmol, 1.5 eq) and acetonitrile (29.81mL, 0.25 M) were added. After the hydrazide was completely dissolved, the aldehyde (1.0 g, 7.45 mmol, 1.0 eq) was added and stirred at room temperature for one hour. After one hour, trifluoro(phenylethynyl)-borane, potassium salt (1.10g, 8.20 mmol, 1.1 eq) was added in one portion, and the reaction mixture was cooled to -40ºC. After 5 minutes of stirring, boron trifluoride etherate (1.06 g, 7.45 mmol, 1.0 eq) was added dropwise at -40ºC under N_2. It was then stirred at -40ºC for one hour, then at room temperature overnight. Upon completion, the reaction mixture was transferred to a separatory funnel with 2 x 20mL of diethyl ether, followed by a wash with a 0.5 M solution of NaOH 2x 30mL. The organic layer was then dried with $MgSO_4$, and the solvent was removed under

reduced pressure. The resulting crude material was purified by flash column chromatography with 1000 mL 100% hexane as eluent to obtain product as colorless oil (714 mg, 44% isolated yield)

^{1}H NMR (500 MHz, $CDCl_3$) δ 7.32 – 7.25 (m, 4H), 7.24 – 7.21 (m, 3H), 7.19 – 7.15 (m, 3H), 6.16 – 6.09 (m, 1H), 5.60 (q, J = 6.6 Hz, 1H), 2.90 – 2.73 (m, 2H), 2.56 – 2.38 (m, 2H).

Spectroscopic data was consistent with literature reported values. [18]

1-methyl-2-(octa-1,2-dien-1-yl)benzene [1h]:

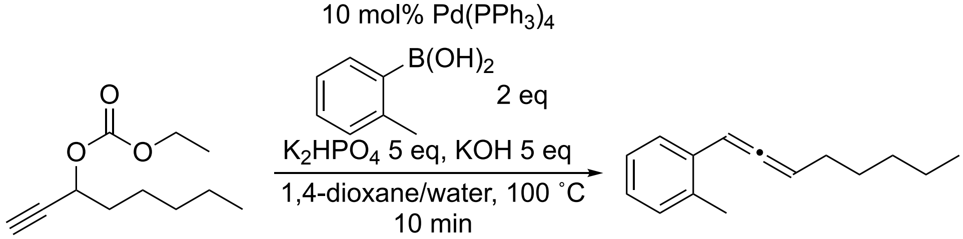

To a 50 mL round-bottom flask equipped with a Teflon coated stir bar, was charged K_2HPO_4 (4.35 g, 25 mmol, 5.0 eq), DI water (16 mL), KOH (1.40g, 25 mmol, 5.0 eq), ethyl oct-1-yn-3-yl carbonate (991 mg, 5.0 mmol, 1.0 eq), *o*-tolylboronic acid (1.36 g, 10 mmol, 2.0 eq), and dioxane (32 mL). The mixture was rapidly heated to 100 ˚C, and then $Pd(PPh_3)_4$ (578 mg, 0.5 mmol, 10 mol%) was added in one portion. The flask was sealed with a rubber sealed, and then the mixture was kept at 100˚C for 10 min. At which time, the reaction mixture was vacuum-filtered through a silica pad which was washed with 50 mL diethyl ether. The solvents were removed by the aid of rotary evaporation from the combined filtrate to afford the crude material, which was dry loaded onto silica gel, the product was isolated by flash column chromatography with 500 mL 100% petroleum ether as eluent to obtain product as colorless oil (478 mg, 48% isolated yield).

^{1}H NMR (400 MHz, $CDCl_3$) δ 7.42 – 7.37 (m, 1H), 7.19 – 7.06 (m, 3H), 6.35 – 6.29 (m, 1H), 5.54 (q, J = 6.7 Hz, 1H), 2.37 (s, 3H), 2.20 – 2.09 (m, 2H), 1.56 – 1.45 (m, 2H), 1.41 – 1.31 (m, 4H), 0.92 (t, J = 7.1 Hz, 3H).

^{13}C NMR (101 MHz, $CDCl_3$) δ 206.0, 134.9, 133.4, 130.6, 127.2, 126.6, 126.2, 94.3, 91.9, 31.6, 29.0, 28.9, 22.6, 20.0, 14.2.

Spectroscopic data was consistent with literature reported values. [21]

5-(benzyloxy)penta-1,2-dien-1-yl)benzene [1i]:

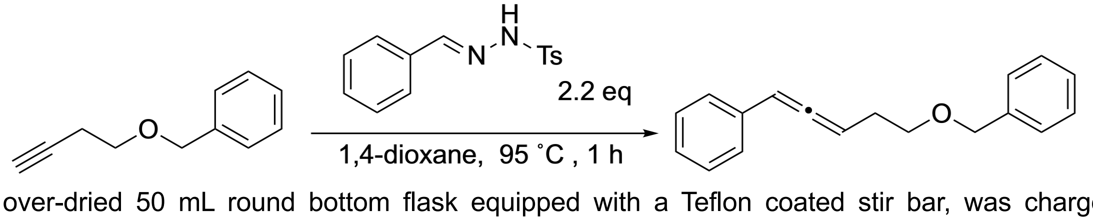

To an over-dried 50 mL round bottom flask equipped with a Teflon coated stir bar, was charged *N'*-benzylidene-4-methylbenzenesulfonohydrazide (3.020 g, 11.00 mmol, 2.2 eq), Lithium *tert*-butoxide (1.400g, 17.50 mmol, 2.2 eq), CuI (190.5 mg, 1.00 mmol, 0.2 eq) and anhydrous 1,4-dioxane (5 mL). The mixture was stirred for 10 min, then a 5 mL 1,4-dioxane solution of ((but-3-yn-1-yloxy)methyl)benzene (801.1 mg, 5 mmol, 1.0 eq) was added to the mixture *via* a syringe. The flask was then sealed and heated at 95 ˚C for 1 hour. Upon completion, the reaction mixture was filtered through a silica pad with acetone.

The solvent was removed by the aid of rotary evaporation from the filtrate to afford the crude material, which was first purified by a flash column chromatography with 10% acetone in hexane, then the combined fractions were then purified by C18 (50 g) reverse phase flash column chromatography with 4/2/1 acetonitrile/methanol/water to afford the pure product as colorless oil (183 mg, 15% isolated yield).

1H NMR (400 MHz, $CDCl_3$) δ 7.37 – 7.32 (m, 4H), 7.32 – 7.26 (m, 5H), 7.22 – 7.14 (m, 1H), 6.20 – 6.11 (m, 1H), 5.63 (q, *J* = 6.7 Hz, 1H), 4.54 (s, 2H), 3.64 (t, *J* = 6.3 Hz, 2H), 2.51 – 2.41 (m, 2H).
^{13}C NMR (101 MHz, $CDCl_3$) δ 205.7, 138.4, 134.7, 128.6, 128.4, 127.7, 127.6, 126.75, 126.70, 94.9, 91.8, 73.0, 69.6, 29.3.

HRMS: (ESI$^+$) *m/z* $[M+H]^+$ calculated for $C_{18}H_{19}O^+$ 251.1430; found 251.1431

1-(buta-1,2-dien-1-yl)naphthalene [1j]:

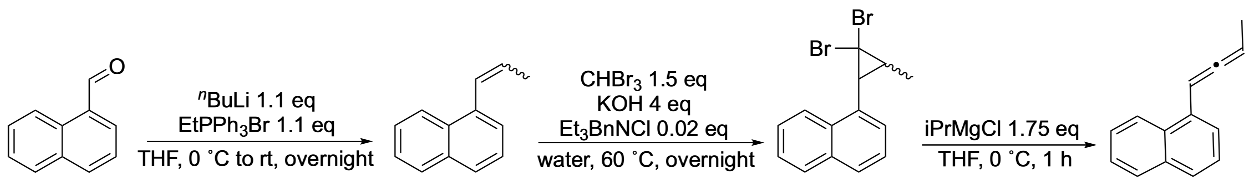

Step 1: To a flame-dried 250 mL round bottom flask quipped with a Teflon coated stir bar, ethyltriphenylphosphonium bromide was dispersed in THF (110 mL), the mixture was cooled to 0˚C by aid of ice-bath. After 10 min, ^nBuLi (10 mL, 2.3 M in Hexane, 22 mmol, 1.1 eq) was slowly added dropwise to the mixture, the result mixture kept at 0˚C for 15 min. 1-naphthaldehyde solution in THF (1 mL/1 mmol) was added to the mixture *via* a syringe, the result mixture was kept at 0˚C for 1 hour before warmed to rt. After stirring overnight, the reaction was quenched with sat. NH_4Cl (15 mL), the organic phase was separated with a separatory funnel, aqueous phase was extracted with diethyl ether (50 mL X 2), combined organic phase was further washed with brine and dried over with anhydrous $MgSO_4$. The solvents were removed by the aid of rotary evaporation from the filtrate to afford the crude material, which was dry loaded onto silica gel, the product was isolated by flash column chromatography with 800 mL 100% hexane 1 L 5% ethyl acetate in hexane as eluent to obtain product as yellow oil (1.9 g, 58% isolated yield, mixture of diastereomers, dr ~ 1.7:1).

^{1}H NMR (400 MHz, $CDCl_3$) δ 8.1 (d, *J* = 8.2 Hz, 0.6H), 8.0 – 8.0 (m, 1H), 7.9 – 7.8 (m, 1.7H), 7.8 (t, *J* = 9.3 Hz, 1.7H), 7.6 – 7.4 (m, 5.8H), 7.4 (dt, *J* = 7.1, 1.3 Hz, 1.48H), 7.1 (d, *J* = 15.5 Hz, 0.68H), 6.9 (d, *J* = 11.5 Hz, 1H), 6.3 – 6.2 (m, 0.6H), 6.1 – 6.0 (m, 1H), 2.0 (dt, *J* = 6.6, 1.5 Hz, 1.8H), 1.8 (dt, *J* = 7.1, 1.7 Hz, 3H).

Step 2: To a 20 mL reaction vial equipped with a Teflon coated stir bar, was charged KOH pellet (1.68g, 30 mmol, 4.0 eq) and DI water (1.2 mL). The vial was capped with a pressure relief cap, the KOH solution was stirred until it cooled down to room temperature. To the cooled KOH solution, was charged 1-(prop-1-en-1-yl)naphthalene (1.26 g, 7.5 mmol, 1.0 eq), *N*-benzyl-*N*,*N*-diethylethanaminium bromide (34 mg, 0.15 mmol, 0.02 eq), and freshly redistilled bromoform (1 mL, 11.25 mmol, 1.5 eq). The mixture was heated to 60˚C and aggressively stirred overnight. Upon completion (judged by TLC), the reaction mixture was diluted with DI water (30 mL) and transferred to a separatory funnel, aqueous phase was extracted with diethyl ether (30 mL X 3), combined organic phase was further washed with brine and dried over with anhydrous $MgSO_4$. The solvents were removed by the aid of rotary evaporation from the filtrate to afford the crude material, which was dry loaded onto silica gel, the product was isolated by flash column chromatography with 1000 mL 100% hexane as eluent to obtain product as yellow oil (721 mg, 28% isolated yield, mixture of diastereomers, dr ~ 2:1).

^{1}H NMR (400 MHz, CDCl$_3$) δ 8.2 (d, *J* = 8.3 Hz, 0.5H), 8.2 (d, *J* = 8.5 Hz, 1H), 7.9 – 7.8 (m, 3H), 7.7 – 7.5 (m, 3H), 7.5 – 7.4 (m, 2H), 7.3 – 7.2 (m, 0.5H), 3.2 (d, *J* = 11.1 Hz, 0.5H), 2.8 (d, *J* = 8.3 Hz, 1H), 2.4 – 2.3 (m, 0.5H), 2.2 – 2.1 (m, 1H), 1.6 (d, *J* = 6.3 Hz, 3H), 1.4 (d, *J* = 6.6 Hz, 1.5H).

Note: The product was directly used for the next step without full characterization due to instability, the purification process of this compound should be in dark to avoid the decomposition under light.

Step 3: To an oven-dried 20 mL reaction vial equipped with a Teflon coated stir bar, was charged 1-(2,2-dibromo-3-methylcyclopropyl)naphthalene (503 mg, 1.48 mmol, 1.0 eq) and anhydrous THF (2.9 mL). The result solution was cooled to 0˚C by aid of ice bath. After 10 min, isopropylmagnesium chloride 2M in THF (1.29 mL, 2.59 mmol, 1.75 eq) was added dropwise *via* syringe to the vial. The reaction vial was kept in ice bath for 1 hour. Upon completion, the reaction was quenched by sat. NH_4Cl (10 mL), then the mixture was transferred to a separatory funnel, aqueous phase was extracted with diethyl ether (30 mL X 3), combined organic phase was further washed with brine and dried over with anhydrous $MgSO_4$. The solvents were removed by the aid of rotary evaporation from the filtrate to afford the crude material, which was dry loaded onto silica gel, the product was isolated by flash column chromatography with 1000 mL 100% hexane as eluent to obtain product as bright yellow oil (211 mg, 79% isolated yield).

^{1}H NMR (400 MHz, CDCl$_3$) δ 8.24 (d, *J* = 8.0 Hz, 1H), 7.86 (d, *J* = 7.6 Hz, 1H), 7.74 (d, *J* = 8.2 Hz, 1H), 7.57 (d, *J* = 7.4 Hz, 1H), 7.50 (t, *J* = 7.0 Hz, 2H), 7.45 (t, *J* = 7.7 Hz, 1H), 6.86 – 6.74 (m, 1H), 5.59 (p, *J* = 7.0 Hz, 1H), 1.91 – 1.81 (m, 3H).

^{13}C NMR (101 MHz, CDCl$_3$) δ 207.6, 134.1, 131.4, 131.0, 128.8, 127.4, 126.1, 125.8, 125.7, 125.4, 123.7, 90.7, 88.7, 14.3.

Spectroscopic data was consistent with literature reported values.[22]

Note: This allene should be stored under nitrogen in -10 ˚C freezer due to the instability, it is also necessary to repurify the allene by passing through a 2-inch silica pad with petroleum ether before using for transfer hydrodeuteration reaction.

2-chloro-1-methyl-4-(octa-1,2-dien-1-yl)benzene [1k]:

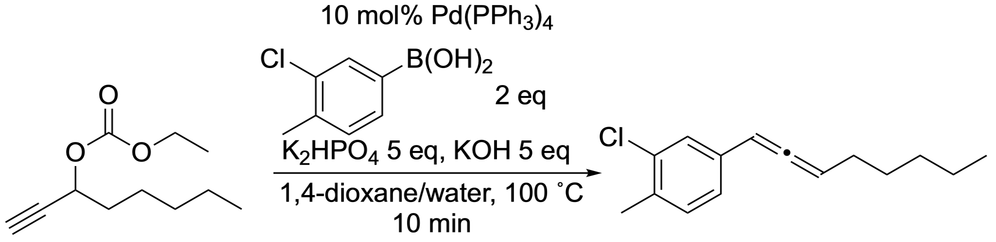

To a 50 mL round-bottom flask equipped with a Teflon coated stir bar, was charged K_2HPO_4 (4.35 g, 25 mmol, 5.0 eq), DI water (16 mL), KOH (1.40g, 25 mmol, 5.0 eq), ethyl oct-1-yn-3-yl carbonate (991 mg, 5.0 mmol, 1.0 eq), (3-chloro-4-methylphenyl)boronic acid (1.7 g, 10 mmol, 2.0 eq), and dioxane (32 mL). The mixture was rapidly heated to 100˚C, and then $Pd(PPh_3)_4$ (578 mg, 0.5 mmol, 10 mol%) was added in one portion. The flask was sealed with a rubber sealed, and then the mixture was kept at 100˚C for 10 min. At which time, the reaction mixture was vacuum-filtered through a silica pad which was washed with 50 mL diethyl ether. The solvents were removed by the aid of rotary evaporation from the combined filtrate to afford the crude material, which was dry loaded onto silica gel, the product was isolated by flash column chromatography with 500 mL 100% petroleum ether as eluent to obtain product as colorless oil (381 mg, 33% isolated yield)

^{1}H NMR (400 MHz, CDCl$_3$) δ 7.28 (s, 1H), 7.15 (d, *J* = 7.9 Hz, 1H), 7.07 (d, *J* = 7.8 Hz, 1H), 6.07 – 6.02 (m, 1H), 5.59 (q, *J* = 6.6 Hz, 1H), 2.36 (s, 3H), 2.18 – 2.09 (m, 2H), 1.54 – 1.44 (m, 2H), 1.40 – 1.29 (m, 4H), 0.91 (t, *J* = 3.9 Hz, 3H).

^{13}C NMR (101 MHz, CDCl$_3$) δ 205.2, 134.7, 134.7, 134.2, 131.1, 127.0, 124.9, 95.7, 93.6, 31.5, 28.9, 28.8, 22.6, 19.9, 14.2.

HRMS: (ESI$^+$) *m/z* [M+H]$^+$ calculated for $C_{15}H_{20}Cl^+$ 235.1248; found 235.1249

Note: This allene should be stored under nitrogen in -10 ˚C freezer due to the instability, it is also necessary to repurify the allene by passing through a 2-inch silica pad with petroleum ether before using for transfer hydrodeuteration reaction.

1-fluoro-4-(hepta-1,2-dien-1-yl)benzene [1l]:

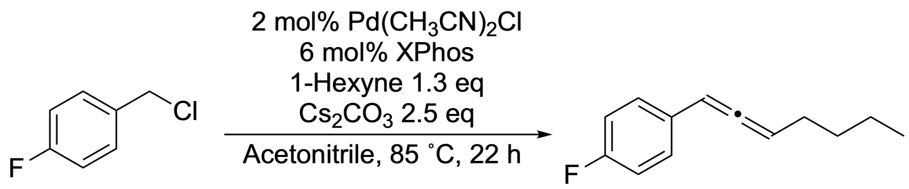

To an oven-dried schlenk flask equipped with a Teflon coated stir bar, was charged $Pd(CH_3CN)_2Cl_2$ (10.4 mg, 0.040 mmol, 0.02 eq), XPhos (55.8 mg, 0.120 mmol, 0.06 eq), cesium carbonate (1.63 g, 5 mmol, 2.5 eq), and acetonitrile (0.3 mL). The mixture was degassed by three vacuum-nitrogen cycles. Then a 10 mL solution of 1-(chloromethyl)-4-fluorobenzene (289.1 mg, 2.00 mmol, 1.0 eq) and 1-hexyne (213.6 mg, 2.6 mmol, 1.3 eq) in acetonitrile was added to the flask *via* syringe. The result slurry was stirred at 85 ˚C for 22 hr. Upon completion, the reaction mixture was filtered through a Celite pad with 10 mL DCM, the pad was washed with DCM (30 mL X 3), solvents were removed from the combined filtrate to afford the crude product, which was further purified by flash column chromatography with 300 mL100% 40-60 petroleum ether as eluent to obtain the product as a pale yellow oil (92 mg, 24% isolated yield)

^{1}H NMR (400 MHz, CDCl$_3$) δ 7.25 – 7.20 (m, 2H), 7.04 – 6.91 (m, 2H), 6.13 – 6.04 (m, 1H), 5.56 (q, *J* = 6.3 Hz, 1H), 2.17 – 2.08 (m, 2H), 1.49 – 1.35 (m, 4H), 0.91 (t, *J* = 7.2 Hz, 3H).

Spectroscopic data were consistent with literature reported values. [23]

Note: This allene was stored under nitrogen in -10 ˚C freezer due to the instability, it was repurified by passing through a 2-inch silica pad with petroleum ether before using for transfer hydrodeuteration reaction.

1-bromo-4-(nona-1,2-dien-1-yl)benzene [1m]:

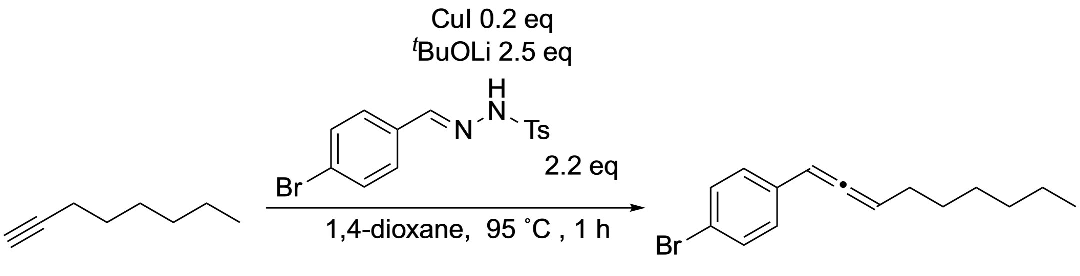

To an over-dried 50 mL round bottom flask equipped with a Teflon coated stir bar, was charged N'-(4-bromobenzylidene)-4-methylbenzenesulfonohydrazide (3.1100 g, 8.800 mmol, 2.2 eq), Lithium *tert*-butoxide (1.1200g, 14.00 mmol, 3.5 eq), CuI (152.4 mg, 0.8000 mmol, 0.2 eq) and anhydrous 1,4-dioxane (45 mL). The mixture was stirred for 10 min, then a 5 mL 1,4-dioxane solution of 1-octyne (440.8 mg, 4 mmol, 1.0 eq) was added to the mixture *via* a syringe. The flask was then sealed and heated at 95 ˚C for overnight. Upon completion, the reaction mixture was filtered through a silica pad with ethyl acetate. The solvent was removed by the aid of rotary evaporation from the filtrate to afford the crude material, which was first purified by a flash column chromatography with 100% hexane to afford the pure product as yellow oil. (600 mg, 53% isolated yield).

1H NMR (500 MHz, $CDCl_3$) δ 7.40 (d, *J* = 8.5 Hz, 2H), 7.15 (d, *J* = 8.4 Hz, 2H), 6.09 – 6.03 (m, 1H), 5.56 (q, *J* = 6.7 Hz, 1H), 2.16 – 2.09 (m, 2H), 1.50 – 1.43 (m, 2H), 1.39 – 1.33 (m, 2H), 1.33 – 1.24 (m, 1H), 0.88 (t, *J* = 6.9 Hz, 3H).

^{13}C NMR (126 MHz, $CDCl_3$) δ 205.4, 134.4, 131.7, 128.2, 120.3, 95.7, 93.9, 31.8, 29.2, 29.0, 28.8, 22.8, 14.2.

Spectroscopic data were consistent with literature reported values. [24]

Note: The purification method for this allene was not efficient to remove all the residue trace amount of 1-octyne across multiple batches. Due to the stability of this allene, the other efforts for purification were not performed in this study. The material was still used for the transfer hydrodeuteration reactions. Results indicated the impurity has no negative impact on the reaction.

2-chloro-1-(penta-1,2-dien-1-yl)-3-(trifluoromethyl)benzene [1n]:

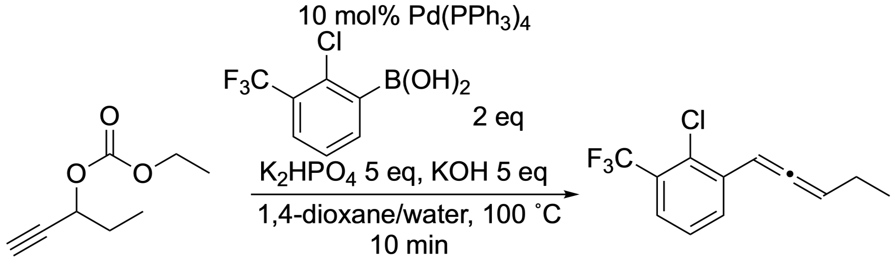

To a 50 mL round-bottom flask equipped with a Teflon coated stir bar, was charged K_2HPO_4 (4.35 g, 25 mmol, 5.0 eq), DI water (16 mL), KOH (1.40g, 25 mmol, 5.0 eq), ethyl pent-1-yn-3-yl carbonate (781 mg, 5.0 mmol, 1.0 eq), (2-chloro-3-(trifluoromethyl)phenyl)boronic acid (2.24 g, 10 mmol, 2.0 eq), and dioxane (32 mL). The mixture was rapidly heated to 100˚C, and then $Pd(PPh_3)_4$ (578 mg, 0.5 mmol, 10 mol%) was added in one portion. The flask was sealed with a rubber sealed, and then the mixture was kept at 100˚C for 10 min. At which time, the reaction mixture was vacuum-filtered through a silica pad which was washed with 50 mL diethyl ether. The solvents were removed by the aid of rotary evaporation from the combined filtrate to afford the crude material, which was dry loaded onto silica gel, the product was isolated by flash column chromatography with 800 mL 100% petroleum ether as eluent to obtain product as colorless oil (673 mg, 55% isolated yield, 94% purity).

^{1}H NMR (400 MHz, $CDCl_3$) δ 7.67 (d, *J* = 7.1 Hz, 1H), 7.54 (d, *J* = 7.9 Hz, 1H), 7.28 (t, *J* = 8.2 Hz, 1H), 6.78 – 6.63 (m, 1H), 5.73 (q, *J* = 6.4 Hz, 1H), 2.26 – 2.11 (m, 2H), 1.11 (t, *J* = 7.4 Hz, 3H).

^{13}C NMR (101 MHz, $CDCl_3$) δ 206.5, 135.6, 131.477, 131.467, 126.4, 125.8 (q, *J* = 5.5 Hz), 123.1 (q, *J* = 272.7), 97.6, 91.3, 21.8, 13.5.

^{19}F NMR (376 MHz, $CDCl_3$) δ -62.55.

HRMS: (ESI$^+$) *m/z* $[M+H]^+$ calculated for $C_{12}H_{11}ClF_3^+$ 247.0496; found 247.0497

propyl 4-(buta-1,2-dien-1-yl)benzoate [1o]:

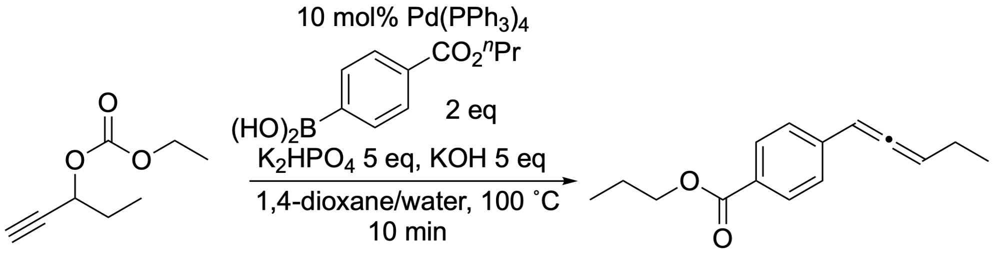

To a 50 mL round-bottom flask equipped with a Teflon coated stir bar, was charged K_2HPO_4 (4.35 g, 25 mmol, 5.48 eq), DI water (16 mL), KOH (1.40g, 25 mmol, 5.0 eq), but-3-yn-2-yl ethyl carbonate (711 mg, 4.55 mmol, 1.0 eq), (4-(propoxycarbonyl)phenyl)boronic acid (2.08 g, 10 mmol, 2.2 eq), and dioxane (32 mL). The mixture was rapidly heated to 100˚C, and then $Pd(PPh_3)_4$ (578 mg, 0.5 mmol, 11 mol %) was added in one portion. The flask was sealed with a rubber sealed, and then the mixture was kept at 100˚C for 10 min. At which time, the reaction mixture was vacuum-filtered through a silica pad which was washed with 50 mL diethyl ether. The solvents were removed by the aid of rotary evaporation from the combined filtrate to afford the crude material, which was dry loaded onto silica gel, the product was isolated by flash column chromatography with 200 mL 100% petroleum ether 1 L 10% diethyl ether in petroleum as gradient eluent to obtain product as yellow oil (174 mg, 16% isolated yield).

^{1}H NMR (400 MHz, $CDCl_3$) δ 7.97 (d, *J* = 7.8 Hz, 2H), 7.34 (d, *J* = 7.8 Hz, 2H), 6.23 – 6.15 (m, 1H), 5.69 (q, *J* = 6.3 Hz, 1H), 4.27 (t, *J* = 6.6 Hz, 2H), 2.22 – 2.11 (m, 2H), 1.79 (h, *J* = 6.9 Hz, 2H), 1.09 (t, *J* = 7.5 Hz, 3H), 1.03 (t, *J* = 7.5 Hz, 3H).

^{13}C NMR (101 MHz, $CDCl_3$) δ 206.1, 166.7, 140.3, 130.0, 128.7, 126.4, 97.3, 95.0, 66.6, 22.3, 21.9, 13.5, 10.7.

Note: The product was directly used for the next step without full characterization due to instability. The purification method for this allene was not efficient to remove the residue trace amount of deboronlation impurity across multiple batches. Despite this, the impurity has no negative impact on the transfer hydrodeuteration. This allene was stored under nitrogen in -10 ˚C freezer due to the instability, it was repurified by passing through a 2-inch silica pad with ether/petroleum ether before using for transfer hydrodeuteration reaction.

4-(Hexa-1,2-dien-1-yl)-*N*-methylaniline [1p]:

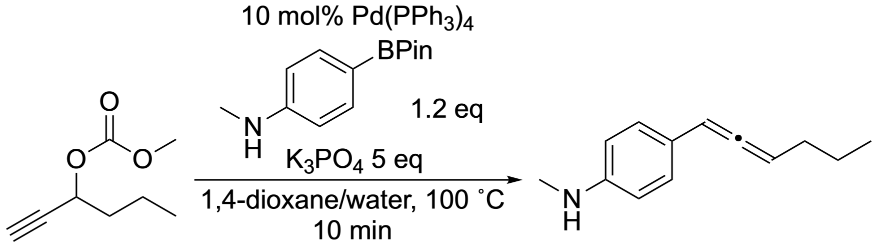

To a 50 mL round-bottom flask equipped with a Teflon coated stir bar, was charged K_3PO_4 (2.1 g, 10 mmol, 5.0 eq), DI water (6.6 mL), hex-1-yn-3-yl methyl carbonate (381 mg, 80% wt in diethyl ether, 2.0 mmol, 1.0 eq), *N*-methyl-4-(4,4,5,5-tetramethyl-1,3,2-dioxaborolan-2-yl)aniline (513 mg, 2.2 mmol, 1.1 eq), and

dioxane (13.2 mL). The mixture was rapidly heated to 100˚C, and then $Pd(PPh_3)_4$ (231 mg, 0.2 mmol, 10 mol%) was added in one portion. The flask was sealed with a rubber sealed, and then the mixture was kept at 100˚C for 10 min. At which time, the reaction mixture was vacuum-filtered through a silica pad which was washed with 25 mL MeOH. The solvents were removed by the aid of rotary evaporation from the combined filtrate to afford the crude material, which was dry loaded onto an empty 10g Sfär cartridge, isolated by Biotage® Isolera™ One Flash Chromatography System with 12 CV of 0 to 100% EA in Hexane gradient with 25 g Sfär HC cartridge to obtain product as brown oil (195 mg, 52% isolated yield).

^{1}H NMR (400 MHz, $CDCl_3$) δ 7.14 (d, *J* = 8.5 Hz, 2H), 6.60 (d, *J* = 8.6 Hz, 2H), 6.13 – 5.99 (m, 1H), 5.52 (q, *J* = 6.6 Hz, 1H), 2.84 (s, 3H), 2.14 – 2.04 (m, 2H), 1.50 (h, *J* = 7.4 Hz, 2H), 0.96 (t, *J* = 7.4 Hz, 3H). NH signal is missing due to the background H/D exchange.

^{13}C NMR (101 MHz, $CDCl_3$) δ 204.2, 148.4, 127.7, 123.9, 112.8, 94.8, 94.4, 31.4, 31.0, 22.6, 13.9.

HRMS: (ESI$^+$) *m/z* $[M+H]^+$ calculated for $C_{13}H_{18}N^+$ 188.1434; found 188.1435

7-(hexa-1,2-dien-1-yl)-4-methyl-3,4-dihydro-2*H*-benzo[*b*][1,4]oxazine [1q]:

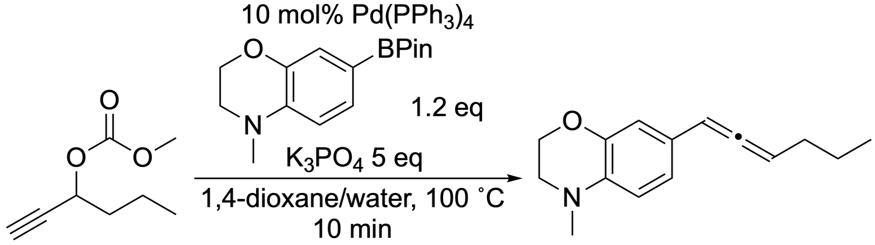

To a 50 mL round-bottom flask equipped with a Teflon coated stir bar, was charged K_3PO_4 (1.6 g, 7.5 mmol, 5.0 eq), DI water (5 mL), hex-1-yn-3-yl methyl carbonate (293 mg, 80% wt in diethyl ether, 2.0 mmol, 1.0 eq), 4-methyl-7-(4,4,5,5-tetramethyl-1,3,2-dioxaborolan-2-yl)-3,4-dihydro-2*H*-benzo[*b*][1,4]oxazine (495 mg, 1.8 mmol, 1.2 eq), and dioxane (10 mL). The mixture was rapidly heated to 100˚C, and then $Pd(PPh_3)_4$ (173 mg, 0.15 mmol, 10 mol%) was added in one portion. The flask was sealed with a rubber sealed, and then the mixture was kept at 100˚C for 10 min. At which time, the reaction mixture was vacuum-filtered through a silica pad which was washed with 25 mL MeOH. The solvents were removed by the aid of rotary evaporation from the combined filtrate to afford the crude material, which was dry loaded onto an empty 10g Sfär cartridge, isolated by Biotage® Isolera™ One Flash Chromatography System with 12 CV of 0 to 35% EA in Hexane gradient with 50 g Sfär HC cartridge to obtain product as pale yellow oil (278 mg, 81% isolated yield).

^{1}H NMR (400 MHz, $CDCl_3$) δ 6.78 – 6.74 (m, 2H), 6.60 (d, *J* = 8.0 Hz, 1H), 6.04 – 5.97 (m, 1H), 5.51 (q, *J* = 6.6 Hz, 1H), 4.29 (t, *J* = 4.4 Hz, 2H), 3.24 (t, *J* = 4.2 Hz, 2H), 2.9 (s, 3H), 2.11 – 2.04 (m, 2H), 1.49 (sext, *J* = 7.3 Hz, 2H), 0.95 (t, *J* = 7.4 Hz, 3H).

^{13}C NMR (101 MHz, $CDCl_3$) δ 204.5, 144.6, 135.7, 125.3, 120.1, 113.9, 112.7, 94.9, 94.3, 65.1, 49.4, 39.0, 31.3, 22.6, 13.9.

Note: The product was directly used for the next step without full characterization due to instability. Upon isolation, this allene should be directly used for reactions without storge. This allene was stored at -30 ˚C with 100 ppm BHT as inhibitor in THF stock solution, rapid decompositions were observed in multiple batches.

***tert*-butyl 4-(4-(hexa-1,2-dien-1-yl)phenyl)piperazine-1-carboxylate [1r]:**

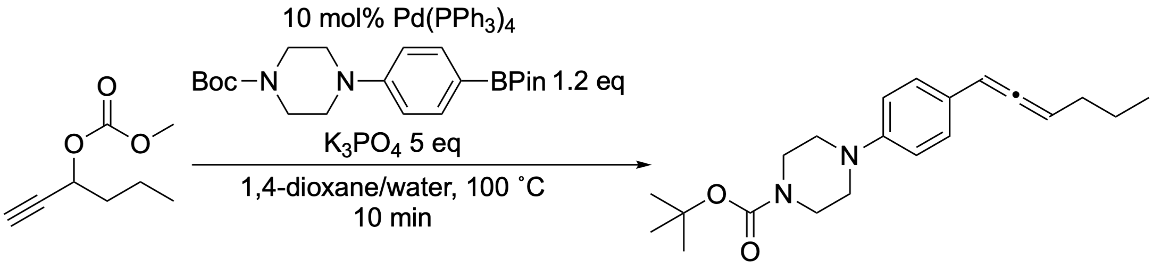

To a 50 mL round-bottom flask equipped with a Teflon coated stir bar, was charged K_3PO_4 (5.3 g, 25 mmol, 5.0 eq), DI water (16 mL), hex-1-yn-3-yl methyl carbonate (976 mg, 80% wt in diethyl ether, 5.0 mmol, 1.0 eq), *tert*-butyl 4-(4-(4,4,5,5-tetramethyl-1,3,2-dioxaborolan-2-yl)phenyl)piperazine-1-carboxylate (2.33 g, 6 mmol, 1.2 eq), and dioxane (32 mL). The mixture was rapidly heated to 100˚C, and then $Pd(PPh_3)_4$ (578 mg, 0.5 mmol, 10 mol%) was added in one portion. The flask was sealed with a rubber septa, and then the mixture was kept at 100˚C for 10 min. At which time, the reaction mixture was vacuum-filtered through a silica pad which was washed with 50 mL MeOH. The solvents were removed by the aid of rotary evaporation from the combined filtrate to afford the crude material, which was dry loaded onto an empty 10g Sfär cartridge, isolated by Biotage® Isolera™ One Flash Chromatography System with 12 CV of 0 to 20% EA in Hexane gradient with 100 g Sfär HC cartridge to obtain product as brown oil (844 mg, 49% isolated yield).

<u>^{1}H NMR (400 MHz, $CDCl_3$)</u> δ 7.20 (d, J = 8.7 Hz, 2H), 6.87 (d, J = 8.3 Hz, 2H), 6.11 – 6.02 (m, 1H), 5.53 (q, J = 6.6 Hz, 1H), 3.58 (t, J = 4.9 Hz, 2H), 3.11 (t, J = 5.2 Hz, 2H), 2.13 – 2.05 (m, 2H), 1.55 – 1.49 (m, 2H), 1.48 (s, 9H), 0.96 (t, J = 7.4 Hz, 3H).

<u>^{13}C NMR (101 MHz, $CDCl_3$)</u> δ 204.8, 154.8, 127.5, 117.1, 95.0, 94.1, 80.1, 49.7, 43.6, 31.2, 28.6, 22.6, 13.9. Two carbon signals overlapped in the aromatic range.

<u>Note:</u> The product was directly used for the next step without full characterization due to instability. This allene was stored under nitrogen in -10 ˚C freezer due to the instability, it was repurified by passing through a 2-inch silica pad with ether/petroleum ether before using for transfer hydrodeuteration reaction.

8-(2-phenylvinylidene)-1,4-dioxaspiro[4.5]decane [1s]:

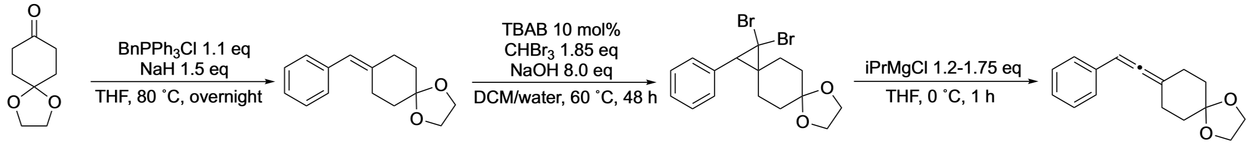

Step 1: To an oven dried 3-neck round bottom flask equipped with a Teflon stir bar, reflux condenser, and nitrogen adaptor, $BnPPh_3Cl$ (1.82 g, 4.67 mmol) and NaH (60 wt% in mineral oil, 255 mg, 6.38 mmol, 1.5 eq) were added under nitrogen stream, then 1 volume anhydrous THF was added to the flask *via* a syringe in one portion. The flask was sealed with rubber septum and connected to the condenser. The reaction mixture was kept at 70 ˚C for 5 hours under nitrogen. Upon completion of deprotonation, the orange slurry was cooled to room temperature, a solution of 1,4-dioxaspiro[4.5]decan-8-one (663.7 mg, 4.25 mmol, 1.0 eq) in THF (1 mL) was added to the flask *via* a syringe. The orange color was faded in 10-30 minutes at room temperature then the result slurry was kept at 70 ˚C overnight under nitrogen. Upon completion (judged by TLC), the reaction mixture was quenched by 2 mL D_2O, then poured into a separatory funnel with 100 mL DI water. The aqueous layer was extracted with 100 mL X 3 diethyl ether. Combined organic layers were washed with brine, dry over anhydrous $MgSO_4$. Solvents were removed from the filtrate with the aid of rotary evaporation to afford the crude product which was further purified by flask column chromatography (700 mL 2% EA in Hexane) to afford the 8-benzylidene-1,4-dioxaspiro[4.5]decane as colorless (427 mg, 43% isolated yields.) The spectra data consistent with literature reported data.

Step 2: To a dried 3-necck round bottom flask equipped with a Teflon stir bar, reflux condenser, and nitrogen adaptor, TBAB (58 mg, 0.18 mmol, 0.1 eq), NaOH (583 mg, 3.65 mmol, 4.0 eq), and DI water (0.6 mL) was combined. The solution was allowed to cool in a water bath, then a solution of 8-benzylidene-1,4-dioxaspiro[4.5]decane (420 mg, 1.82 mmol, 1.0 eq) in 1 volume DCM was added to the flask *via* a syringe in one portion. The flask was sealed with rubber septum and connected to the condenser. The flask was kept in a 60 ˚C oil bath, then a solution of bromoform (0.32 mL, 3.65 mmol, 2.0 eq) in 1 volume DCM was added to the flask *via* a syringe over 30 mins. Upon completion, the reaction was kept at 60 ˚C overnight under nitrogen. Depending on the TLC, if there is still d-alkene residue, additional 0.2 eq of bromoform and 4 eq of NaOH will be added to the flask and the reaction will be kept at 60 ˚C for another 24 hours. Upon fully consumption of d-alkene, the reaction mixture was diluted with 75 mL DI water and extracted with 75 mL X 3 DCM. Combined organic layers were washed with 200 mL DI water, 200 mL brine, and dry over anhydrous $MgSO_4$. Solvents were removed from the filtrate with the aid of rotary evaporation to afford the crude product which was further purified by flask column chromatography (1→10% EA in Hexane) to afford the 1,1-dibromo-2-phenyl-7,10-dioxadispiro[2.2.46.23]dodecane as yellow oil (0.3 g, 41% isolated yield). Due to the stability in ambient condition, the isolated materials were directly used for the next steps after drying with high vac.

Step 3: To a flame dried round bottom flask equipped with a Teflon stir bar, and a rufbber septum, a solution of 1,1-dibromo-2-phenyl-7,10-dioxadispiro[2.2.46.23]dodecane (300 mg, 0.746 mmol, 1.0 eq) in anhydrous THF (1M) was added to the flask *via* a syringe under nitrogen. The solution was cooled to 0 ˚C, then isopropylmagnesium chloride solution (0.65 mL, 1.31 mmol, 1.75 eq) was added dropwise to the flask. The result slurry was kept at room temperature for 1 hr. Upon completion, the reaction was quenched with 50 mL 2M HCl, then extracted with 75 mL X 3 diethyl ether or petroleum ether. Combined organic layers were washed with 100 mL DI water, 100 mL brine, and dry over anhydrous $MgSO_4$. Solvents were removed from the filtrate with the aid of rotary evaporation to afford the crude product which was further purified by flask column chromatography (1→5% EA in Hexane) to afford 8-(2-phenylvinylidene)-1,4-dioxaspiro[4.5]decane as white solid (133 mg, 73% isolated yield).

^{1}H NMR (500 MHz, $CDCl_3$) δ 7.25 – 7.20 (m, 4H), 7.15 – 7.06 (m, 1H), 6.04 (p, *J* = 2.2 Hz, 1H), 3.92 (s, 4H), 2.41 – 2.27 (m, 4H), 1.77 (t, *J* = 6.5 Hz, 4H).

^{13}C NMR (126 MHz, $CDCl_3$) δ 200.0, 135.8, 128.7, 126.74, 126.67, 108.4, 104.2, 93.1, 64.5, 35.8, 28.5.

Spectroscopic data were consistent with literature reported values. [25]

hepta-1,2-dien-3-ylbenzene [1t]:

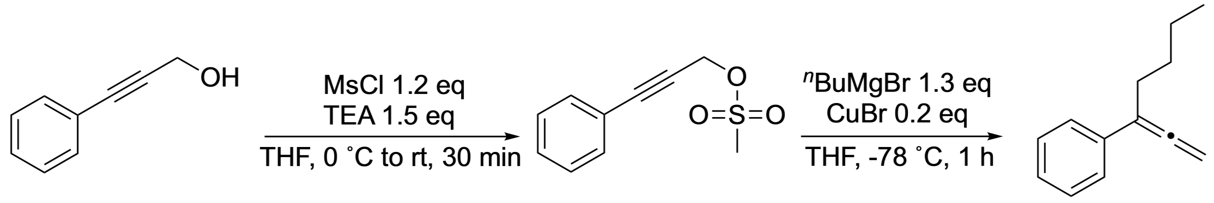

Step 1: To a 100 mL round bottom flask equipped with a Teflon coated stir bar, charged with 3-phenylprop-2-yn-1-ol (1.32 g, 10 mmol, 1.0 eq), THF (40 mL), and triethylamine (2.1 mL, 15 mmol, 1.5 eq). The mixture was cooled to 0˚C by ice bath, then methanesulfonyl chloride (0.93 mL, 12 mmol, 1.2 eq) was added dropwise to the mixture. After 30 min, the reaction was quenched by DI water (20 mL), the mixture was transferred to a separatory funnel, aqueous phase was extracted with diethyl ether (30 mL X 3), combined organic phase was further washed with brine and dried over with anhydrous $MgSO_4$ to obtain the crude product, which was used in the next step without purification.

Step 2: To a flame-dried 100 mL round bottom flask equipped with a Teflon coated stir bar, charged with CuBr (287 mg, 2 mmol, 0.2 eq, freshly prepared), THF (15 mL), and 3-phenylprop-2-yn-1-yl methanesulfonate (2.1 g, 10 mmol, 1 eq). The result slurry was cooled to -78˚C by a dry ice acetone bath, then freshly prepared ^{n}BuMgBr 1M in THF (13 mL, 13 mmol, 1.3 eq) was added slowly to the slurry. The result mixture was further stirred at -78˚C for 1 h. Upon completion (judged by TLC), the reaction was quenched with sat. NH_4Cl (15 mL), then the mixture was transferred to a separatory funnel with 50 mL diethyl ether, the aqueous layer was extracted with diethyl ether (25 mL X 2), then the combined organic layers were washed with brine, and dried over with anhydrous $MgSO_4$. After removing solvents, the crude material was wet loaded to silica gel, which was further purified by flash column chromatography with 500 mL 100% hexane as eluent to obtain product as pale yellow oil (848 mg, 49% isolated yield).

^{1}H NMR (400 MHz, $CDCl_3$) δ 7.46 – 7.41 (m, 2H), 7.38 – 7.30 (m, 2H), 7.21 (m, 1H), 5.08 (t, *J* = 3.3 Hz, 2H), 2.49 – 2.39 (m, 2H), 1.66 – 1.51 (m, 2H), 1.50 – 1.37 (m, 2H), 0.96 (t, *J* = 7.3 Hz, 3H).

^{13}C NMR (101 MHz, $CDCl_3$) δ 208.8, 136.7, 128.5, 126.7, 126.1, 105.1, 78.2, 30.2, 29.3, 22.7, 14.1.

Spectroscopic data were consistent with literature reported values. [26]

(6-methylhepta-4,5-dien-1-yl)benzene [1u]:

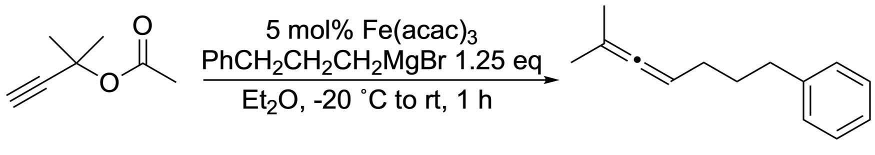

To a flame dried 250 mL round bottom flask equipped with a Teflon coated stir bar, was added $Fe(acac)_3$ (279.8 mg, 0.7925 mmol, 0.05 eq) and 25 mL anhydrous diethyl ether. Then 2-methylbut-3-yn-2-yl acetate (2.0 g, 15.85 mmol, 1.0 eq) in 80 mL diethyl ether was added to the flask at –20 ˚C. After 5 minutes, the freshly prepared (3-phenylpropyl)magnesium bromide (0.4 M in diethyl ether 50 mL, 19.82 mmol, 1.25 eq) was added to the flask via a syringe. The reaction was kept at –20 ˚C for 1 hour. Upon completion, the reaction was quenched with 25 mL saturated NH_4Cl and 25 mL DI water, the aqueous layer was extracted with 3 X 50 mL diethyl ether, combined diethyl ether layers were washed with brine, dry over $MgSO_4$. The solvents were removed from the filtrate to afford the crude material which was further purified by flash column chromatography with 1000 mL 100% petroleum ether as eluent afford the pure product as colorless liquid (1.09 g, 37% isolated yield).

^{1}H NMR (400 MHz, $CDCl_3$) δ 7.33 – 7.27 (m, 2H), 7.23 – 7.16 (m, 3H), 5.03 – 4.94 (m, 1H), 2.66 (t, *J* = 7.8 Hz, 2H), 2.04 – 1.96 (m, 2H), 1.77 – 1.72 (m, 2H), 1.72 – 1.69 (m, 6H).

Spectroscopic data were consistent with literature reported values. [27]

nona-1,2-dien-1-ylcyclopentane [1v]:

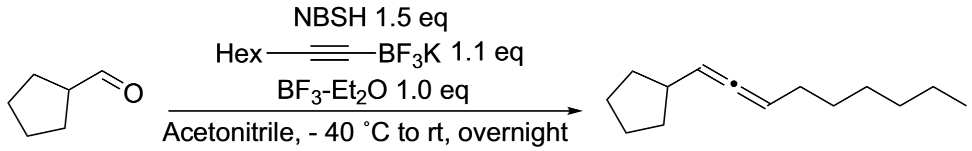

To a flame dried round bottom flask equipped with a Teflon coated stir bar, 2-Nitrobenzenesulfonylhydrazide (977.4 g, 4.50 mmol, 1.5 eq) and acetonitrile (4.5 mL) were added. After the hydrazide was completely

dissolved, the cyclopentanecarbaldehyde (204.9 mg, 3.00 mmol, 1.0 eq) was added and stirred at room temperature for one hour. After one hour, trifluoro(oct-1-yn-1-yl)-borane, potassium salt (972.5 mg, 4.50 mmol, 1.5 eq) was added in one portion, and the reaction mixture was cooled to –40 ˚C. After 5 minutes of stirring, boron trifluoride etherate (638.7 mg, 4.50 mmol, 1.0 eq) was added dropwise at –20 ˚C under N_2. It was then stirred at –20 ˚C for one hour, then at room temperature overnight. Upon completion, the reaction mixture was transferred to a separatory funnel with 2 x 20mL of diethyl ether, followed by a wash with a 0.5 M solution of NaOH 2x 30mL. The organic layer was then dried with $MgSO_4$, and the solvent was removed under reduced pressure. The resulting crude material was purified by flash column chromatography with 500 mL 100% hexane as eluent to obtain product as colorless oil (324 mg, 56% isolated yield)

^{1}H NMR (400 MHz, $CDCl_3$) δ 5.15 – 5.05 (m, 2H), 2.50 – 2.36 (m, 1H), 2.02 – 1.92 (m, 2H), 1.82 – 1.70 (m, 2H), 1.65 – 1.60 (m, 2H), 1.54 – 1.50 (m, 1H), 1.43 – 1.23 (m, 11H), 0.88 (t, J = 6.8 Hz, 3H).
^{13}C NMR (101 MHz, $CDCl_3$) δ 202.7, 96.1, 91.9, 39.5, 33.0, 32.9, 31.9, 29.4, 29.2, 29.0, 25.03, 25.01, 22.8, 14.3.

HRMS: (ESI$^+$) m/z [M-H]$^+$ calculated for $C_{14}H_{23}$ 191.1800; found 191.1794

trimethyl(5-phenylpenta-1,2-dien-1-yl)silane [1w]:

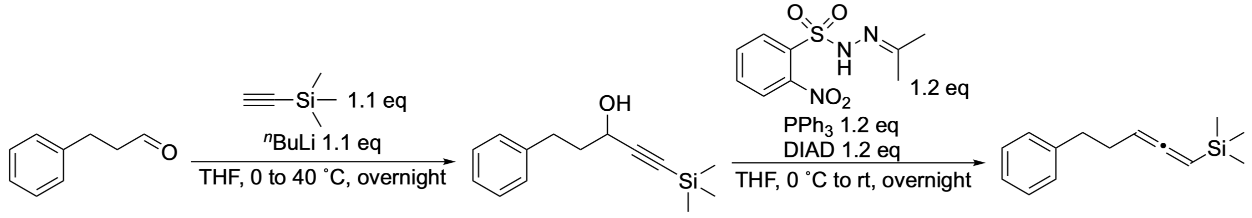

Step 1: To an oven-dried 200 mL round bottom flask equipped with a Teflon coated stir bar, was charged Ethynyltrimethylsilane (1.41 mL, 10 mmol, 1.1 eq), and anhydrous THF (6.5 mL). The resulting solution was cooled to 0 ˚C, ^{n}BuLi (2.5 M in Hexane, 4.0 mL, 10 mmol, 1.1 eq) was added dropwise to the flask *via* syringe. The resulting solution was kept in the ice bath for 1 hour. Upon completion, a 6 mL solution of 3-phenylpropanal in THF (1.22 g, 9.09 mmol, 1.0 eq) was added in one portion to the flask. The reaction mixture was kept in 40 ˚C overnight. Upon completion, the reaction was quenched with 25 mL of DI water and 25 mL of sat. NH_4Cl. Aqueous layer was extracted with ethyl acetate (50 mL X 3), combined organic layers were washed with brine, and dry over Na_2SO_4. Solvents were removed from the filtrate to afford the crude material, which was further purified by flash column chromatography with 1L 10% ethyl acetate in hexane as eluent to obtain the product as a colorless oil (1.44 g, 68% isolated yield)

^{1}H NMR (500 MHz, $CDCl_3$) δ 7.32 – 7.26 (m, 2H), 7.24 – 7.17 (m, 3H), 4.36 (q, J = 6.3 Hz, 1H), 2.80 (t, J = 7.9 Hz, 2H), 2.10 – 1.96 (m, 2H), 1.79 (d, J = 5.5 Hz, 1H), 0.19 (s, 9H).

Spectroscopic data were consistent with literature reported values. [28]

Step 2: To an over-dried 200 mL round bottom flask equipped with a Teflon coated stir bar, was charged 5-phenyl-1-(trimethylsilyl)pent-1-yn-3-ol (1.2 g, 5.16 mmol, 1.0 eq), 2-nitro-*N'*-(propan-2-ylidene)benzenesulfonohydrazide (1.25 g, 6.25 mmol, 1.21 eq), PPh_3 (1.64 g, 6.25 mmol, 1.21 eq), and anhydrous THF (50 mL). Diisopropyl azodicarboxylate (1.22 mL, 6.20 mmol, 1.2 eq) was added dropwise to the solution at 0˚C. The flask was allowed to stir at room temperature for 2 h. Upon completion, a solution of trifluoroethanol and water (1:1 V/V, 60 mL) was added to the flask in one portion, the result mixture was further stirred at room temperature for 2 h. Upon completion, the reaction mixture was transferred to a separatory funnel, extracted with diethyl ether (50 mL X 3), combined ether layers washed with brine, dry over Na_2SO_4. Solvents were removed from the filtrate to afford the crude material which was further purified

by flash column chromatography with 1L 100% hexane as eluent to obtain the product as a colorless oil (0.528 g, 47% isolated yield)

^{1}H NMR (400 MHz, $CDCl_3$) δ 7.34 – 7.26 (m, 2H), 7.23 – 7.16 (m, 3H), 4.98 – 4.89 (m, 1H), 4.88 – 4.78 (m, 1H), 2.71 (t, J = 7.9 Hz, 2H), 2.36 – 2.23 (m, 2H), 0.09 (s, 9H).

^{13}C NMR (101 MHz, $CDCl_3$) δ 210.0, 142.1, 128.6, 128.4, 126.0, 83.2, 83.0, 36.2, 29.9, -0.8.

Spectroscopic data were consistent with literature reported values. [29]

(1*S*,4*R*)-1-isopropyl-4-methyl-2-vinylidenecyclohexane [1x]:

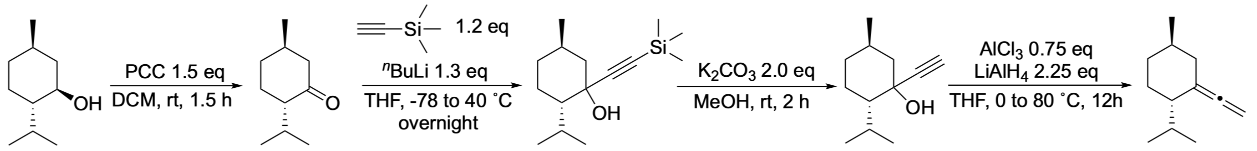

Step 1: To the flask equipped a Teflon stir bar, L-menthol (10.00 g, 63.99 mmol, 1.0 eq) was dissolved in DCM (100 mL). Then PCC (20.69 g, 95.99 mmol, 1.5 eq) was added to the solution in 3 portions. Upon completion, the reaction was left on rt for 1.5 hrs. Then the reaction mixture was filtered through a 3" silica pad, then silica pad was washed with 250 mL diethyl ether, crude material was concentrated to around 20 mL, then filter through another silica pad with 100 mL ether, then solvents were removed by rotary evaporation afford the product as pale orange oil (quantitative yield).

Step 2: To a dried 3-neck flask equipped a Teflon stir bar, THF (20 mL) was added to ^{n}BuLi (2.5 M in hexanes, 20.3 mL, 50.57 mmol, 1.3 eq) at -78˚C. Then the solution was kept at -78˚C for 10 mins. TMS-acetylene (6.47 mL, 46.68 mmol, 1.2 eq) was added slowly to the solution. The result solution kept at -78˚C for 20 mins. Then a solution of (2*S*,5*R*)-2-isopropyl-5-methylcyclohexan-1-one (88 wt% in diethyl ether, 6.82 g, 38.90 mmol, 1.0 eq) in 20 mL THF was added dropwise to the flask. The reaction mixture was kept at rt for 5 h, then 40˚C overnight under nitrogen. The mixture quenched with 50 mL sat. NH_4Cl, then extracted with EA 50 mL X3. Combined EA layers were washed with brine, then dry over $MgSO_4$. The solvents were removed from the filtrates to afford the crude material which was further purified with flask column chromatography (silica gel, 1L 1% EA in Hex to afford the diastereomer 1, then 1L 2% EA in Hex to afford the diastereomer 2). Both diastereomers were confirmed as the target product, both fractions combined by MeOH for the next step.

Step 3: To the flask containing the starting material with a Teflon stir bar, methanol (200 mL) was added to the flask. Then potassium carbonate (10.75 g, 77.80 mmol, 2.00 eq) was added to the solution in one portion. The flask was sealed with a rubber septa, the result slurry was stirred at rt for 2 hrs. Upon completion, the reaction mixture was filtered through a celite pad with methanol (ca. 100 mL), solvents were removed from the filtrate to afford the crude product, which was further purified with flask column chromatography (silica gel, 2L 2% EA in Hexane) to afford the product as pale-yellow oil (mixture of diastereomers, 6.21 g, 89% isolated yield over 2-steps).

Step 4: To a dried Schlenk flask equipped a Teflon stir bar, $AlCl_3$ (3.33 g, 24.96 mmol, 0.75 eq) was added to anhydrous THF (33 mL) at 0 ˚C. The solution allows to stir at rt for 30 min. Then a slurry of LAH (2.84 g, 74.98 mmol, 2.25 eq) in THF (10 mL) was added to the $AlCl_3$ solution in one portion at 0˚C. The result mixture kept at 0˚C for 15 min. (2*S*,5*R*)-1-ethynyl-2-isopropyl-5-methylcyclohexan-1-ol (6.00 g, 32.28 mmol, 1.00 eq) in anhydrous THF (20 mL) was added to the flask in one portion. The result mixture was kept at 80˚C overnight. Upon completion, the reaction was quenched with 5 mL DI

water, 5 mL 15% NaOH, and 5 mL DI water. Then the mixture was filtered, precipitates washed with DCM. Combined filtrates concentrate under vacuum to afford crude material, which was further purified with flash column chromatography on silica gel with 1L 100% PE to afford the product as colorless oil (1.52 g, 28% isolated yield).

^{1}H NMR (500 MHz, $CDCl_3$) δ 4.62 (dd, *J* = 3.8, 3.8 Hz, 2H), 2.27 (ddd, *J* = 13.0, 4.0, 2.1 Hz, 1H), 1.86 – 1.75 (m, 3H), 1.68 – 1.57 (m, 2H), 1.56 – 1.48 (m, 1H), 1.20 – 1.10 (m, 1H), 1.04 – 0.96 (m, 1H), 0.94 (d, *J* = 6.9 Hz, 3H), 0.92 (d, *J* = 6.6 Hz, 3H), 0.86 (d, *J* = 6.9 Hz, 3H).

Spectroscopic data were consistent with literature reported values. [30]

(8*S*,9*S*,13*S*,14*S*)-3-methoxy-13-methyl-17-vinylidene-7,8,9,11,12,13,14,15,16,17-decahydro-6*H*-cyclopenta[*a*]phenanthrene [1y]

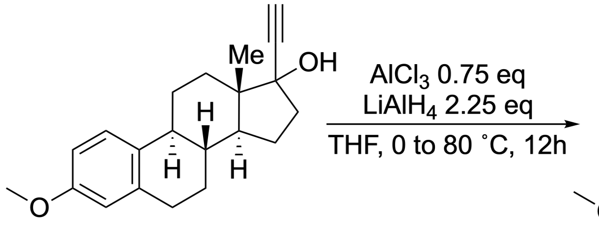

To a dried Schlenk flask equipped a Teflon stir bar, $AlCl_3$ (257.7 mg, 1.93 mmol, 0.75 eq) was added to anhydrous THF (3 mL) at 0 ˚C. The solution allows to stir at rt for 30 min. Then a slurry of LAH (220 mg, 5.80 mmol, 2.25 eq) in THF (6 mL) was added to the $AlCl_3$ solution in one portion at 0˚C. The result mixture kept at 0˚C for 15 min. (8*R*,9*S*,13*S*,14*S*)-17-ethynyl-3-methoxy-13-methyl-7,8,9,11,12,13,14,15,16,17-decahydro-6*H*-cyclopenta[*a*]phenanthren-17-ol (800 mg, 2.58 mmol, 1.00 eq) in anhydrous THF (20 mL) was added to the flask in one portion. The result mixture was kept at 80˚C overnight. Upon completion, the reaction was quenched with 0.3 mL DI water, 0.3 mL 15% NaOH, and 0.9 mL DI water. Then the mixture was filtered, precipitates washed with DCM. Combined filtrates concentrate under vacuum to afford crude material, which was further purified with flask column chromatography on silica gel with 800mL 50% DCM in PE to afford the product as white solid (370 mg, 49% isolated yield).

^{1}H NMR (500 MHz, DMSO-d_6) δ 7.17 (d, *J* = 8.5 Hz, 1H), 6.68 (dd, *J* = 8.5, 2.9 Hz, 1H), 6.61 (d, *J* = 3.1 Hz, 1H), 4.83 – 4.71 (m, 2H), 3.69 (s, 3H), 3.28 (s, 2H), 2.84 – 2.77 (m, 2H), 2.60 – 2.53 (m, 1H), 2.42 – 2.30 (m, 2H), 2.24 – 2.13 (m, 1H), 1.90 – 1.73 (m, 3H), 1.49 – 1.29 (m, 6H), 0.87 (s, 3H).

Spectroscopic data were consistent with literature reported values.[30]

2-(4-(Hexa-1,2-dien-1-yl)phenyl)isoindolin-1-one [1z]:

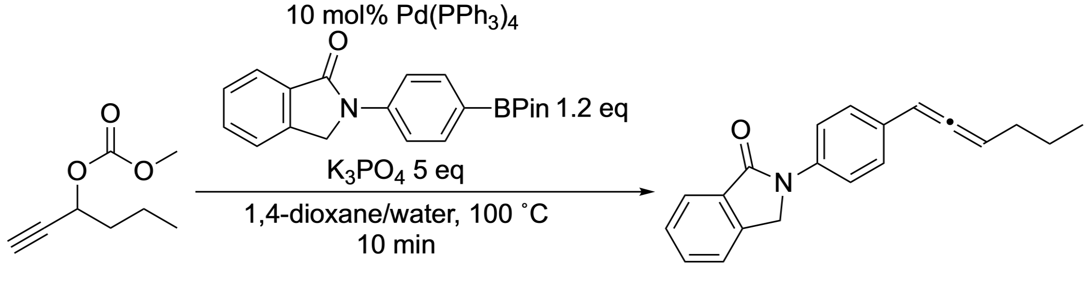

To a 200 mL round-bottom flask equipped with a Teflon coated stir bar, was charged K_3PO_4 (1.7 g, 8 mmol, 5.0 eq), DI water (5.5 mL), hex-1-yn-3-yl methyl carbonate (313 mg, 80% wt in diethyl ether, 1.6 mmol, 1.0 eq), 2-(4-(4,4,5,5-Tetramethyl-1,3,2-dioxaborolan-2-yl)phenyl)isoindolin-1-one (1.1g, 3.2 mmol, 2.0 eq), and dioxane (5.5 mL). The mixture was rapidly heated to 100˚C, and then $Pd(PPh_3)_4$ (185 mg, 0.16 mmol, 10 mol%) was added in one portion. The flask was sealed with a rubber septa, and then the mixture was kept at 100˚C for 10 min. At which time, the reaction mixture was vacuum-filtered through a silica pad which was washed with 50 mL MeOH. The solvents were removed by the aid of rotary evaporation from the combined filtrate to afford the crude material, which was dry loaded onto an empty 10g Sfär cartridge, isolated by Biotage® Isolera™ One Flash Chromatography System with 12 CV of 0-20% EA in Hexane gradient with 100 g Sfär HC cartridge to obtain product as white solid (298 mg, 63% isolated yield).

<u>^{1}H NMR (400 MHz, $CDCl_3$)</u> δ 7.96 – 7.90 (m, 1H), 7.85 – 7.79 (m, 2H), 7.62 – 7.57 (m, 1H), 7.54 – 7.48 (m, 2H), 7.35 (d, *J* = 8.7 Hz, 2H), 6.16 – 6.11 (m, 1H), 5.59 (q, *J* = 6.7 Hz, 1H), 4.85 (s, 2H), 2.20 – 2.05 (m, 2H), 1.52 (h, *J* = 7.3 Hz, 2H), 0.98 (t, *J* = 7.4 Hz, 3H).

<u>^{13}C NMR (101 MHz, $CDCl_3$)</u> δ 205.4, 167.5, 140.2, 138.2, 133.5, 132.2, 131.5, 128.5, 127.4, 124.3, 122.7, 119.7, 95.2, 94.1, 50.9, 31.0, 22.5, 13.9.

<u>Note:</u> The product was directly used for the next step without full characterization due to instability. This allene was stored under nitrogen in -10 ˚C freezer due to the instability, it was repurified by passing through a 2-inch silica pad with ether/petroleum ether before using for transfer hydrodeuteration reaction.

d_1- allenes were prepared by the indicated synthetic route using substituted d-benzaldehydes:

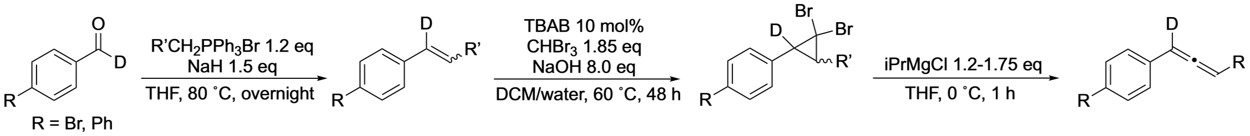

General procedure of Wittig reaction (10 - 30 mmol scale): To an oven dried 3-neck round bottom flask equipped with a Teflon stir bar, reflux condenser, and nitrogen adaptor, Wittig salt and NaH (60 wt% in mineral oil) were added under nitrogen stream, then 1 volume anhydrous THF was added to the flask *via* a syringe in one portion. The flask was sealed with rubber septum and connected to the condenser. The reaction mixture was kept at 70 ˚C for 5 hours under nitrogen. Upon completion of deprotonation, the orange slurry was cooled to room temperature, a solution of substituted benzaldehyde-d in minimal amount of THF was added to the flask *via* a syringe. The orange color was faded in 10-30 minutes at room temperature then the result slurry was kept at 70 ˚C overnight under nitrogen. Upon completion (judged by TLC), the reaction mixture was quenched by 2 mL D_2O, then poured into a separatory funnel with 100 mL DI water. The aqueous layer was extracted with 100 mL X 3 diethyl ether. Combined organic layers were washed with brine, dry over anhydrous $MgSO_4$. Solvents were removed from the filtrate with the aid of rotary evaporation to afford the crude product which was further purified by flask column chromatography (800 mL → 1L 100% Hexane) to afford the d-alkenes as Z/E mixture in good isolated yields.

General procedure of d-gembromocyclopropane reaction (1.5 – 4.0 g scale): To a dried 3-neck round bottom flask equipped with a Teflon stir bar, reflux condenser, and nitrogen adaptor, TBAB, NaOH, and DI water was combined. The solution was allowed to cool in a water bath, then a solution of d-alkene in 1 volume DCM was added to the flask *via* a syringe in one portion. The flask was sealed with rubber septum and connected to the condenser. The flask was kept in a 60 ˚C oil bath, then a solution of bromoform (redistilled and stabilized with ethanol) in 1 volume DCM was added to the flask *via* a syringe over 30 mins. Upon completion, the reaction was kept at 60 ˚C overnight under nitrogen. Depending on the TLC, if there is still d-alkene residue, additional 0.2 eq of bromoform and 4 eq of NaOH will be added to the flask and the reaction will be kept at 60 ˚C for another 24 hours. Upon fully consumption of d-alkene, the reaction mixture was diluted with 75 mL DI water and extracted with 75 mL X 3 DCM. Combined organic layers were washed with 200 mL DI water, 200 mL brine, and dry over anhydrous $MgSO_4$. Solvents were removed from

the filtrate with the aid of rotary evaporation to afford the crude product which was further purified by flask column chromatography (800 mL → 1L 100% Hexane) to afford the d-genbromocyclopropanes as mixture of diastereomers in good isolated yields. Due to their stability in ambient condition, the isolated materials were directly used for the next steps after drying with high vac.

General procedure of Skattebøl rearrangement (1 – 6 g scale): To a flame dried round bottom flask equipped with a Teflon stir bar, and a rubber septum, a solution of d-genbromocyclopropanes in anhydrous THF (1M) was added to the flask *via* a syringe under nitrogen. The solution was cooled to 0 ˚C, then isopropylmagnesium chloride solution was added dropwise to the flask. The result slurry was kept at room temperature for 1 hr. Upon completion, the reaction was quenched with 50 mL 2M HCl, then extracted with 75 mL X 3 diethyl ether or petroleum ether. Combined organic layers were washed with 100 mL DI water, 100 mL brine, and dry over anhydrous $MgSO_4$. Solvents were removed from the filtrate with the aid of rotary evaporation to afford the crude product which was further purified by flask column chromatography (500 mL → 1L 100% Hexane) to afford d-allenes in good isolated yield. All isolated d-allenes were stored under nitrogen atmosphere in a -10 ˚C before using.

benzaldehyde-*d*

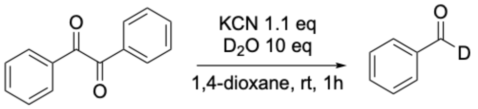

To a flamed dried 300 mL round bottom flask equipped with a Teflon stir bar, benzil (40.0 g, 190.27 mmol, 1.00 eq), freshly recrystallized with 200 proof ethanol, was added under nitrogen stream. The flask was sealed with a rubber septum, then anhydrous 1,4-dioxane (95 mL) was added *via* a syringe in one portion. Upon the solid fully dissolved, D_2O (1.90 mol, 34.3 mL, 10.00 eq) was added to the solution slowly. The result fine slurry was stirred at room temperature for 10 mins. To the result slurry, potassium cyanide (13.6 g, 209.29 mmol, 1.10 eq) was added in 4 portion of ca. 3 g over 10 mins under nitrogen stream. The result mixture was allowed to stir at room temperature for 1h. Upon completion, the mixture was poured into a separatory funnel with 200 mL DI water, extracted with 100 mL X 3 diethyl ether. Combined ether layers were washed with 200 mL DI water, 100 mL X 2 brine, and dry over anhydrous Na_2SO_4. Solvents were removed from the filtrate to afford the crude material, which was further purified by vacuum distillation. 0.1 mmHg 51-53 ˚C bp, to give the pure product as colorless oil, 10g, 49% isolated yield (99.3% purity, 0.6 wt% 1,4-dioxane residue, 99% deuterium incorporation).

^{1}H NMR (500 MHz, $CDCl_3$) δ 10.01 (s, 0.01H), 7.92 – 7.84 (m, 2H), 7.67 – 7.58 (m, 1H), 7.58 – 7.49 (m, 2H).

^{13}C NMR (126 MHz, $CDCl_3$) δ 192.4 (t, J = 26.4 Hz), 136.5 (t, J = 3.6 Hz), 134.6, 129.8, 129.1.

Spectroscopic data were consistent with literature reported values. [31]

Note: Potassium cyanide is extremely toxic, please follow SOP while working with this procedure (https://ehs.yale.edu/sites/default/files/files/potassium-sodium-cyanide-sop.pdf). This compound is sensitive to air and light. It should be stored under nitrogen at -10 ˚C. To avoid the formation of side product (2-hydroxy-2-phenylacetonitrile-*d*), 1.05 eq of potassium cyanide was tested and purified by flash column chromatograph. The additional vacuum distillation was necessary to achieve the target purity. Around 5% isolate yield dropped was observed in that case. Trace amount of this side product has no negative effects on the Wittig steps.

4-bromobenzaldehyde-*d*

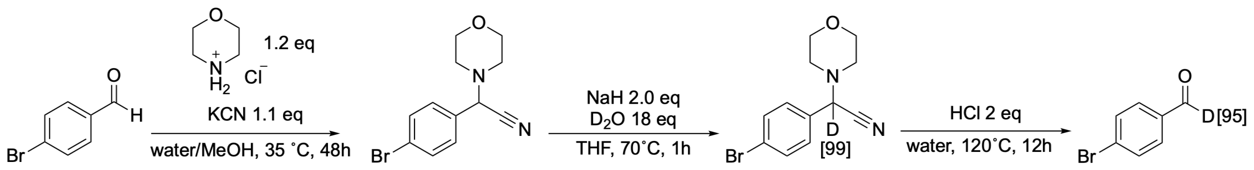

2-(4-bromophenyl)-2-morpholinoacetonitrile: To a flame dried 200 mL round bottom flask equipped with a Teflon stir bar, morpholine HCl (8.02 g, 64.8 mmol, 1.2 eq) was dissolved in DI water (15 mL). To the result solution, potassium cyanide (3.87 g, 59.5 mmol, 1.1 eq) was added in one portion. The mixture was kept at rt for 10 min. A solution of 4-bromobenzaldehyde (10 g, 54.0 mmol, 1.0 eq) in methanol (50 mL) was transferred to the flask *via* a pipette. The result mixture was kept at 35˚C for 48 h. upon completion, the reaction mixture was diluted with 100 mL DI water, then transferred to a separatory funnel with 100 mL diethyl ether. The aqueous layer was extracted twice with 100 mL diethyl ether. The combined ether layers were washed with brine (200 mL X 2), and dry over anhydrous Na_2SO_4. Solvents from the filtrate were removed by rotary evaporation to afford the crude material. The crude material was recrystallized by 200 proof ethanol, then the collected crystals were triturated with 2.5 V petroleum ether, then the product was isolated by vacuum filtration to afford the pure product as white flakes, quantitative yield.

Note: Morpholine HCl was freshly prepared by slowly dropwise conc. HCl (28 mL) to morpholine (ca. 20 mL) at 0˚C over 30 mins. Upon completion, water was removed by rotary evaporation, solvent swap with methanol, followed by azeotrope distillation with toluene, and dried over night with high vac. Morpholine HCl is hydroscopic, the material should be stored in a vacuum desiccator or under nitrogen atmosphere. HCN gas will be formed when mixing morpholine HCl and potassium cyanide, this reaction needs to set up in a well-ventilated fume hood. Potassium cyanide is extremely toxic, please follow SOP while working with this procedure (https://ehs.yale.edu/sites/default/files/files/potassium-sodium-cyanide-sop.pdf).

^{1}H NMR (500 MHz, $CDCl_3$) δ 7.55 (d, *J* = 8.5 Hz, 2H), 7.43 (d, *J* = 8.4 Hz, 2H), 4.76 (s, 1H), 3.78 – 3.66 (m, 4H), 2.57 (t, *J* = 4.7 Hz, 4H).

^{13}C NMR (126 MHz, $CDCl_3$) δ 132.2, 131.8, 129.8, 123.5, 114.9, 66.8, 62.1, 50.1.

2-(4-bromophenyl)-2-morpholinoacetonitrile-*d*: To a dried flask equipped with a Teflon stir bar, 2-(4-bromophenyl)-2-morpholinoacetonitrile (11.25 g, 40 mmol, 1.0 eq) was dissolved in anhydrous THF (40 mL). Sodium hydride (3.20 g, 80 mmol, 2.0 eq) was added to the solution in one portion, the flask was connected to a nitrogen ballon then reflux at 70˚C for 1 h. Upon completion, the result yellow solution was cooled to 0˚C by ice bath, then D_2O (13 mL, 0.72 mol, 18 eq) was added slowly to the flask. The result biphasic mixture was stirred at 0 ˚C for 30 min. Then thionyl chloride (ca. 3 mL) was added carefully to the flask to acidify the solution. The participates were collected by vacuum filtration, then the filter cake was washed with cold DI water, the cake was left on vacuum for 30 min, then dried overnight with high vac afford the product as pale white solid, directly used for the next step without purification, crude NMR indicated 99% D incorporation.

^{1}H NMR (500 MHz, $CDCl_3$) δ 7.58 (d, *J* = 8.2 Hz, 2H), 7.52 (d, *J* = 8.4 Hz, 2H), 3.90 – 3.74 (m, 4H), 2.72 (s, 4H).

4-bromobenzaldehyde-*d*: To a flask quipped a Teflon stir bar, the crude 2-(4-bromophenyl)-2-morpholinoacetonitrile-d (11.29 g, 40 mmol, 1.0 eq) was added to HCl solution (2M 200 mL, 0.2 mol, 5.0 eq). The mixture was reflux at 120 ˚C for overnight under nitrogen atmosphere. Upon completion, the reaction mixture was cooled to rt, then extracted with 200 mL X 3 diethyl ether, the combined ether layers were washed with brine, dry over anhydrous Na_2SO_4. Solvents from the filtrate were removed by rotary evaporation to afford the pure product with mineral oil from the previous step as impurity, 7.12 g, 95% isolated yield, 95% D incorporation.

^{1}H NMR (500 MHz, $CDCl_3$) δ 9.98 (s, 1H, 0.05H), 7.75 (d, *J* = 8.4 Hz, 2H), 7.69 (d, *J* = 8.4 Hz, 2H).

^{13}C NMR (126 MHz, $CDCl_3$) δ 190.9 (t, *J* = 27.13 Hz), 135.18 (t, *J* = 3.6 Hz), 132.6, 131.1, 129.9.

Spectroscopic data were consistent with literature reported values. [32]

Note: HCN gas will be formed during the hydrolysis step, this reaction needs to set up in a well-ventilated fume hood. To prevent any background H/D exchange, the workup for this reaction needs to prevent any aggressive pH changing. Extended reaction time will reduce the deuterium incorporation. The product was stored under nitrogen at -10 ˚C in an amber-glass vial before use.

[1,1'-biphenyl]-4-carbaldehyde-*d*

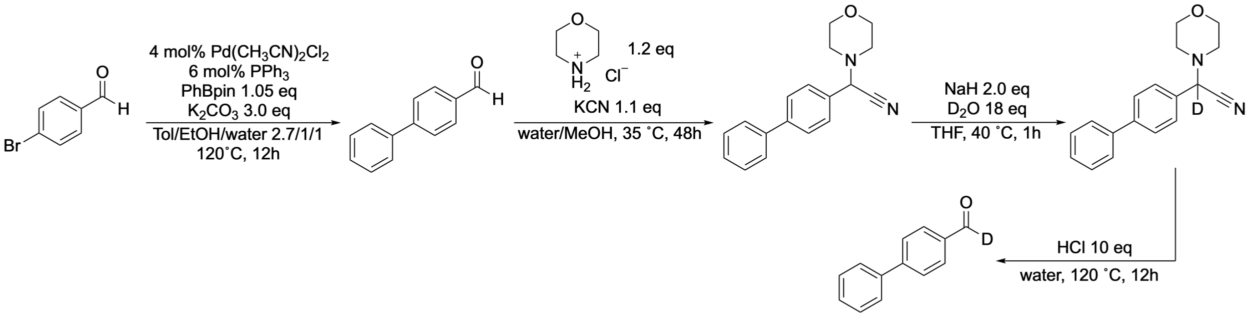

[1,1'-biphenyl]-4-carbaldehyde: To a 250 mL 3-neck round bottom flask equipped with a Teflon stir bar, 200 proof EtOH (36 mL), DI water (36 mL), and toluene (96 mL) were mixed and degassed with nitrogen stream for 10 min. Upon completion, 4-bromobenzaldehyde (5.00 g, 27.02 mmol, 1.0 eq), Phenylboronic acid pinacol ester (5.79 g, 28.38 mmol, 1.05 eq), K_2CO_3 (11.20 g, 81.07 mmol, 3.0 eq), triphenylphosphine (425.3 mg, 1.62 mmol, 0.06 eq), and Bis(acetonitrile)palladium dichloride (280.43 mg, 1.08 mmol, 0.04 eq) were added to the flask. The result bi-phase mixture was further degassed with nitrogen steam for 10 min. The flask was sealed with a rubber septa, connect to nitrogen line and condenser, the mixture was allowed to reflux at 120 ˚C for 12h. Upon completion, the reaction mixture was cooled to room temp, then the mixture was filtered through a celite pad, the flask and the pad were further washed with ethyl acetate (100 mL), combined filtrate were concentrated under vacuum. The residue was dispensed in 150 mL water and 150 mL ethyl acetate and transferred to a separatory funnel. The aqueous layer extracted with ethyl acetate (150 mL X 3), combined ethyl acetate layers were washed with 10% NH_4Cl solution, and dry over Na_2SO_4. The solvent was removed from the filtrate to afford the crude material, which was further purified with 800 mL 10% ethyl acetate in hexane to give the pure compound as white crystal, 4.10 g, 83% isolated yield.

^{1}H NMR (500 MHz, $CDCl_3$) δ 10.1 (s, 1H), 8.0 (d, *J* = 8.4 Hz, 2H), 7.8 (d, *J* = 8.2 Hz, 2H), 7.6 (d, *J* = 7.2 Hz, 2H), 7.5 (t, *J* = 7.4 Hz, 2H), 7.4 (t, *J* = 7.4 Hz, 1H).

Spectroscopic data were consistent with literature reported values. [32]

2-([1,1'-biphenyl]-4-yl)-2-morpholinoacetonitrile: To a flame dried 200 mL round bottom flask equipped with a Teflon stir bar, morpholine HCl (3.26 g, 26.34 mmol, 1.2 eq) was dissolved in DI water (6 mL). To the result solution, potassium cyanide (1.57 g, 24.2 mmol, 1.1 eq) was added in one portion. The mixture was kept at rt for 10 min. A solution of [1,1'-biphenyl]-4-carbaldehyde (4.00 g, 21.95 mmol, 1.0 eq) in methanol (16 mL) was transferred to the flask *via* a pipette. The result mixture was kept at 40˚C for 48 h. upon completion, the reaction mixture was diluted with 100 mL DI water, then transferred to a separatory funnel with 100 mL diethyl ether. The aqueous layer was extracted twice with 100 mL diethyl ether. The combined ether layers were washed with brine (200 mL X 2), and dry over anhydrous Na_2SO_4. Solvents from the

filtrate were removed by rotary evaporation to afford the pure compound as white flakes. No yield was taken, directly used for the next step after drying (high vac, overnight).

^{1}H NMR (500 MHz, $CDCl_3$) δ 7.7 – 7.6 (m, 6H), 7.5 (t, J = 7.6 Hz, 2H), 7.4 (t, J = 7.3 Hz, 1H), 4.9 (s, 1H), 3.8 – 3.7 (m, 4H), 2.7 – 2.6 (m, 4H).

^{13}C NMR (126 MHz, $CDCl_3$) δ 142.3, 140.3, 131.6, 129.1, 128.6, 127.9, 127.7, 127.3, 115.3, 66.9, 62.4, 50.2.

2-([1,1'-biphenyl]-4-yl)-2-morpholinoacetonitrile-*d*: To the flask containing the crude 2-([1,1'-biphenyl]-4-yl)-2-morpholinoacetonitrile and a Teflon stir bar, anhydrous THF (20 mL) was added under nitrogen. NaH (60 wt% in mineral oil, 1.76 g, 43.90 mmol, 2.0 eq) was added to the flask in one portion. The flask was sealed with a rubber septa and connected to a nitrogen line. the result slurry was stirred at 40˚C for 1 h. Upon completion, the result yellow solution was cooled to 0˚C by ice bath, then D_2O (7.13 mL, 0.395 mol, 18 eq) was added slowly to the flask. The result biphasic mixture was stirred at 0 ˚C for 30 min. Then thionyl chloride (ca. 3 mL) was added carefully to the flask to acidify the solution. The mixture was transferred to a separatory funnel with 100 mL diethyl ether and 125 mL ice-cold DI water. Aqueous layer was extracted twice with diethyl ether, combined ether layers were washed with 100 mL DI water and 200 mL brine, dry over anhydrous Na_2SO_4. Solvent was removed from the filtrate to afford the pure compound as pale-yellow flakes, directly used for the next step after drying (high vac, overnight).

^{1}H NMR (500 MHz, $CDCl_3$) δ 7.66 – 7.57 (m, 6H), 7.50 – 7.44 (m, 2H), 7.38 (t, J = 7.3 Hz, 1H), 4.86 (s, 0.01H), 3.81 – 3.69 (m, 4H), 2.74 – 2.52 (m, 4H).

[1,1'-biphenyl]-4-carbaldehyde-*d*: To the flask containing the crude 2-([1,1'-biphenyl]-4-yl)-2-morpholinoacetonitrile-*d*, 2M HCl (100 mL, 0.2 mol, 10 eq) was added in one portion. The result mixture was reflux at 120 ˚C for 12 hr. Upon completion, the reaction mixture was cooled to rt, then extracted with 100 mL X 3 diethyl ether, the combined ether layers were washed with brine, dry over anhydrous Na_2SO_4. Solvents from the filtrate were removed by rotary evaporation to afford the crude product, which was further purified with flask column chromatography (Silica gel, 500 mL 100% Hexane→800 mL 10% ethyl acetate in Hexane) to afford the pure product as pale yellow solid 3.02 g, 75% isolated yield over 3-steps.

^{1}H NMR (500 MHz, $CDCl_3$) δ 10.07 (s, 0.05H), 7.96 (d, J = 8.4 Hz, 2H), 7.76 (d, J = 8.4 Hz, 2H), 7.64 (d, J = 7.2 Hz, 2H), 7.49 (t, J = 7.5 Hz, 2H), 7.42 (t, J = 7.3 Hz, 1H).

^{13}C NMR (126 MHz, $CDCl_3$) δ 191.7 (t, J = 26.7 Hz), 147.4, 139.9, 135.31 (t, J = 3.5 Hz), 130.4, 129.2, 128.6, 127.9, 127.5.

Spectroscopic data were consistent with literature reported values. [33]

Note: HCN gas will be formed during the hydrolysis step, this reaction needs to set up in a well-ventilated fume hood. To prevent any background H/D exchange, the workup for this reaction needs to prevent any aggressive pH changing. Extended reaction time will reduce the deuterium incorporation. The product was stored under nitrogen at -10 ˚C in an amber-glass vial before use.

(buta-1,2-dien-1-yl-1-*d*)benzene [*d₁*-1a]:

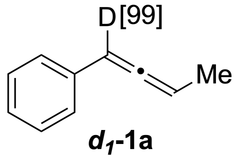

***d*$_1$-1a**

6.5 mmol scale, 520 mg, 76% isolated yield. Pale yellow oil.

^{1}H NMR (500 MHz, $CDCl_3$) δ 7.34 – 7.27 (m, 4H), 7.23 – 7.15 (m, 1H), 6.13 – 6.06 (m, 0.01H), 5.55 (q, *J* = 7.1 Hz, 1H), 1.82 – 1.77 (m, 3H).

^{13}C NMR (126 MHz, $CDCl_3$) δ 206.2, 135.2, 128.7, 126.79, 126.78, 93.9 (t, *J* = 24.47 Hz), 89.8, 14.2.

(4-methylpenta-1,2-dien-1-yl-1-*d*)benzene [*d₁*-1b]:

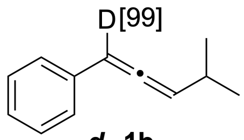

***d₁*-1b** 18.81 mmol scale, 2.61 g, 87% isolated yield. Colorless oil.

^{1}H NMR (500 MHz, DMSO-d_6) δ 7.12 – 7.03 (m, 4H), 7.00 – 6.93 (m, 1H), 6.09 (dd, *J* = 6.3, 3.3 Hz, 0.01H), 5.49 (d, *J* = 5.6 Hz, 1H), 2.19 (hept, *J* = 6.6 Hz, 1H), 0.86 – 0.79 (m, 6H). Two methyl peaks as doublet overlapped in the DMSO-d_6 spectra, thus the data was reported as multiplet.

^{13}C NMR (126 MHz, DMSO-d_6) δ 203.4, 135.1, 129.2, 127.2, 126.6, 102.7, 95.7 (t, *J* = 25.29 Hz), 28.2, 22.9, 22.8.

(penta-1,2-diene-1,5-diyl-1-*d*)dibenzene [*d₁*-1c]:

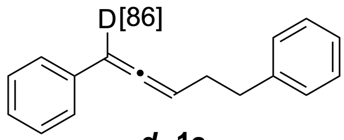

***d₁*-1c** 7.4 mmol scale, 1.52 g, 93% isolated yield. Pale yellow oil.

^{1}H NMR (500 MHz, $CDCl_3$) δ 7.36 – 7.26 (m, 4H), 7.25 – 7.16 (m, 6H), 6.16 – 6.12 (m, 0.14H), 5.65 – 5.57 (m, 1H), 2.90 – 2.76 (m, 2H), 2.57 – 2.41 (m, 2H).

^{13}C NMR (126 MHz, $CDCl_3$) δ 205.4, 141.7, 134.9, 128.73, 128.66, 128.5, 126.8, 126.78, 126.76, 95.1 (d_0-isotopolog), 94.8 (t, *J* = 24.8 Hz), 94.6, 35.6, 30.7.

(2-cyclohexylidenevinyl-1-*d*)benzene [*d₁*-3a]:

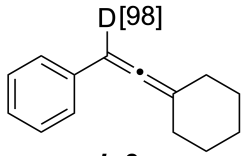

***d₁*-3a** 4 mmol scale, 400 mg, 54% isolated yield. Colorless oil.

^{1}H NMR (500 MHz, $CDCl_3$) δ 7.3 – 7.3 (m, 4H), 7.2 – 7.1 (m, 1H), 6.47 – 6.44 (m, 0.02H), 2.3 – 2.2 (m, 2H), 2.2 – 2.2 (m, 2H), 1.8 – 1.5 (m, 6H).

^{13}C NMR (126 MHz, $CDCl_3$) δ 199.8, 136.3, 128.6, 126.6, 126.4, 106.7, 92.3 (t, *J* = 24.8 Hz), 31.5, 27.9, 26.3.

1-bromo-4-(buta-1,2-dien-1-yl-1-*d*)benzene [*d₁*-3b]:

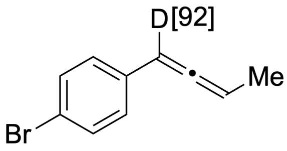

***d*₁-3b**

2.38 mmol scale, 416 mg, 83% isolated yield. Pale yellow oil, solidified at -10 ˚C.

<u>^{1}H NMR (500 MHz, $CDCl_3$)</u> δ 7.42 (d, *J* = 8.4 Hz, 2H), 7.17 (d, *J* = 8.5 Hz, 2H), 6.08 – 6.03 (m, 0.08H), 5.56 (q, *J* = 7.1 Hz, 1H), 1.81 (d, *J* = 7.2 Hz, 3H).

<u>^{13}C NMR (126 MHz, $CDCl_3$)</u> δ 206.3, 134.2, 131.7, 128.3, 120.4, 93.1 (t, *J* = 24.8 Hz), 90.3, 14.1.

4-(4-methylpenta-1,2-dien-1-yl-1-*d*)-1,1'-biphenyl [*d₁*-3c]:

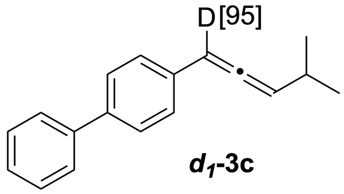

2.53 mmol scale, 479 mg, 88% isolated yield. Colorless oil, solidified at -10 ˚C.

<u>^{1}H NMR (500 MHz, $CDCl_3$)</u> δ 7.63 – 7.58 (m, 2H), 7.57 – 7.53 (m, 2H), 7.43 (t, *J* = 7.9 Hz, 2H), 7.38 (d, *J* = 8.2 Hz, 2H), 7.34 (t, *J* = 7.4 Hz, 1H), 6.23 (dd, *J* = 6.3, 3.1 Hz, 0.05H), 5.65 (d, *J* = 5.8 Hz, 1H), 2.48 (hept, *J* = 6.7 Hz, 1H), 1.13 (dd, *J* = 6.7, 4.0 Hz, 6H).

<u>^{13}C NMR (126 MHz, $CDCl_3$)</u> δ 204.1, 141.1, 139.6, 134.4, 128.9, 127.5, 127.3, 127.1, 127.0, 102.8, 95.3 (t, *J* = 25.1 Hz), 28.6, 22.8, 22.7.

(methyl-*d*₃)triphenylphosphonium bromide

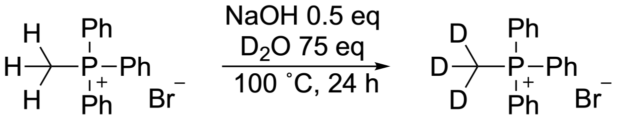

To a flame dried Schlenk flask was charged methyltriphenylphosphonium bromide (5.00 g, 14 mmol, 1 eq) and sodium hydroxide (280 mg, 7 mmol, 0.5 eq) under nitrogen stream, the flask was sealed with a rubber septa. D_2O (19 mL, 1.05 mol, 75 eq) was added to the flask *via* a syringe. The reaction mixture was refluxed under nitrogen at 100 ˚C for 24 h. Upon completion, the reaction mixture was cooled to room temperature, 75 mL DCM was added to the flask, then the bi-phase mixture was transferred to a separatory funnel, the D_2O layer was extracted twice with DCM (50 mL, 25 mL). The combined DCM layers were dried with anhydrous Na_2SO_4, then the solvents were removed from the filtrate by rotary evaporation to afford the crude product as a colorless oil. The crude material was further dried with high vac for 18 h to afford the pure product as white solid (3.64 g, 10.1 mmol, 96% D inc.) Spectroscopic data were consistent with literature reported values. [34]

4-(propa-1,2-dien-1-yl-*d*₃)-1,1'-biphenyl [*d₃*-3d]

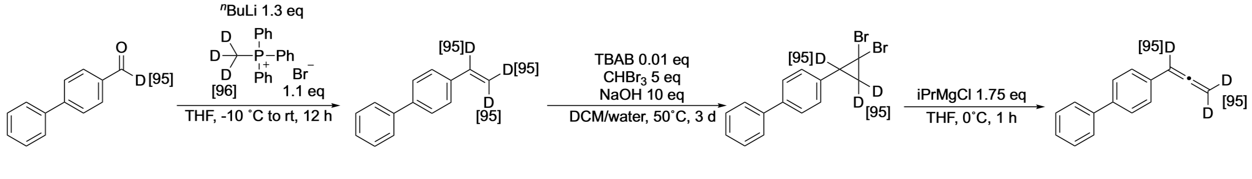

Step 1: To a 100 mL flame dried Schlenk flask equipped with a Teflon stir bar, (methyl-*d*$_3$)triphenylphosphonium bromide (3.24 g, 9.01 mmol, 1.1 eq) was added under nitrogen stream, the material was dried by high vac for 2 h. Upon drying, THF (20 mL) was added to the flask *via* a syringe, the result solution was cooled to 0 ˚C by an ice bath. ^{n}BuLi (4.3 mL, 2.5 M in Hexane, 10.64 mmol, 1.3 eq) was added to the solution *via* a syringe. The result mixture was kept at rt for 30 mins. Upon completion, a saturated solution of [1,1'-biphenyl]-4-carbaldehyde-*d* (1.5 g, 8.19 mmol, 1.0 eq) was added dropwise to the flask *via* a syringe. The mixture was kept at rt for 12 hr. Upon completion (judged by TLC), the reaction was quenched by 20 mL D_2O, then the reaction mixture was transferred to a separatory funnel with 20 mL diethyl ether. The aqueous layer was extracted with diethyl ether (20 mL X 2). Combined ether layers were washed with 10 % NH_4Cl, dry over Na_2SO_4. The solvents were removed from the filtrate to afford crude material, which was further purified by flash column chromatography with 1.5 L 100% hexane to afford the pure product as white solid (300 mg, 1.638 mmol, 20% isolated yield).

^{1}H NMR (500 MHz, $CDCl_3$) δ 7.63 – 7.59 (m, 2H), 7.59 – 7.56 (m, 2H), 7.51 – 7.47 (m, 2H), 7.44 (t, *J* = 7.7 Hz, 2H), 7.35 (t, *J* = 7.5 Hz, 1H), 6.77 – 6.73 (m, 0.05H), 5.79 – 5.77 (m, 0.05H), 5.27 – 5.25 (m, 0.05H).

Step 2: To a vial containing 4-(vinyl-*d*$_3$)-1,1'-biphenyl (300 mg, 1.638 mmol, 1.0 eq) and a Teflon stir bar, was added NaOH (655 mg, 16.37 mmol, 10 eq), TBAB (5.3 mg, 0.016 mmol, 0.01 eq), DI water (0.7 mL), and DCM (1 mL). To the result bi-phase mixture, a solution of bromoform (0.716 mL, 8.18 mmol, 5 eq) in DCM (0.7 mL) was added dropwise to the vial *via* a syringe. The vial was sealed with a pressure relief cap, then the reaction mixture was stirred at 50 ˚C for 3 d. Upon completion (judged by TLC), the reaction mixture was quenched with 20 mL DI water, then the mixture was transferred to a separatory funnel with 20 mL DCM. The aqueous layer was extracted with DCM (20 mL X 2). Combined DCM layers were washed with Sat. NH_4Cl (60 mL), dry over Na_2SO_4. Solvents were removed from the filtrate to afford the crude material, which was further purified by flash column chromatography with 200 mL 100% Hexane → 400 mL 10% DCM in Hexane to afford the pure product as white solid (294 mg, 0.829 mmol, 51% isolated yield). The purified material was directly used to the next step due to the stability.

^{1}H NMR (500 MHz, $CDCl_3$) δ 7.60 (t, *J* = 7.6 Hz, 4H), 7.44 (t, *J* = 7.7 Hz, 2H), 7.38 – 7.34 (m, 1H), 7.33 (d, *J* = 8.4 Hz, 2H), 3.00 – 2.97 (m, 0.05H), 2.17 – 2.14 (m, 0.05H), 2.05 – 2.03 (m, 0.05H).

Step 3: To the vial containing 4-(2,2-dibromocyclopropyl-1,3,3-*d*3)-1,1'-biphenyl (289 mg, 0.814 mmol, 1.0 eq) with a Teflon stir bar, THF (1 mL) was added to the vial. The vial was capped with a pressure relief cap, then the vial was kept at -10 ˚C bath for 5 mins. A solution of isopropylmagnesium chloride (2 M in THF, 0.71 mL, 1.43 mmol, 1.75 eq) was added dropwise to the vial *via* a syringe. The result mixture was kept at 0 ˚C for 1 h. Upon completion (judged by TLC), the reaction mixture was quenched with 20 mL 0.5 M HCl, then the mixture was transferred to a separatory funnel with 20 mL diethyl ether, the aqueous layer was extracted with diethyl ether (20 mL X 2). Combined ether layers were washed with brine and dry over anhydrous Na_2SO_4. Solvents were removed from the filtrate to afford the crude product, which was further purified by flash column chromatography with 600 mL 100% Hexane to afford the pure product as white solid (140 mg, 0.718 mmol, 88% isolated yield).

<u>^{1}H NMR (500 MHz, $CDCl_3$)</u> δ 7.62 – 7.58 (m, 2H), 7.57 – 7.54 (m, 2H), 7.44 (t, *J* = 7.6 Hz, 2H), 7.38 (d, *J* = 8.4 Hz, 2H), 7.34 (t, *J* = 7.4 Hz, 1H), 6.23 – 6.21 (m, 0.05H), 5.19 – 5.16 (m, 0.10H).

<u>^{13}C NMR (126 MHz, $CDCl_3$)</u> δ 210.3, 141.0, 140.0, 133.1, 128.9, 127.5, 127.4, 127.2, 127.1., 93.7 (t, *J* = 25.4 Hz), 78.8 (p, *J* = 26.7 Hz).

II. Supporting Information for the Chiral Analysis of (2Z)-2-buten-1-yl-benzene-*d1* by Chiral Tag Molecular Rotational Resonance (MRR) Spectroscopy

This section of the Supporting Information provides the details of the molecular rotational resonance (MRR) spectroscopy chiral analysis. Chiral analysis is performed using the chiral tag method where a small, chiral tag molecule forms a 1:1 complex with the analyte in the pulsed jet expansion used to introduce samples into MRR spectrometers. This derivatization by non-covalent interactions converts the enantiomers of the isotopically chiral analyte into spectroscopically distinguishable diastereomeric complexes.

Sample for Analysis

IUPAC: (2Z)-2-buten-1-yl-benzene

1-phenyl-2-butene

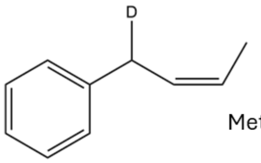

Methyl allyl-d1

Note that in this SI section, the analyte is often called methyl allyl-d1.

MRR Methods

Computational Methods

Isomers of (2Z)-2-buten-1-yl-benzene complexing with 1,1,1-trifluoropropan-2-ol (TFIP) (the chiral tag used in this work) were generated using CREST[23] 2.12 NCI mode. Single point energy calculations were performed using Gaussian16[24] at B3LYP GD3BJ / def2-TZVP and the isomers within 600 cm^{-1} were submitted for optimization at the same level of theory. A summary of the calculated isomers is presented below.

Broadband Measurements

Broadband rotational spectra in the 2-8 GHz frequency range were measured using the chirped-pulse Fourier transform microwave spectrometer at the University of Virginia.[25] The

measurement of the spectrum using the undeuterated reaction product ((2Z)-2-buten-1-yl-benzene) used 50 mg of sample in each of three reservoir nozzles. The sample was heated to 50°C. Complexes with an enantioenriched sample of (*S*)- 1,1,1-trifluoropropan-2-ol (TFIP) were measured using a neon gas mixture prepared in a small external sample cylinder (volume approximately 350 mL). This preparation used 10 μL of the (*S*)-TFIP (SynQuest, EE = 99.6) chiral tag with a total pressure of 120 psi of neon. A regulated pressure of 20-30 psi was flowed over the heated sample reservoirs and introduced into the spectrometer through pulsed solenoid valves.

Four samples of enantioenriched reaction product were also measured. In this case, 60 mg of the sample was loaded into the reservoir nozzles. The NMR spectra provided with these samples indicated a large amount of sample impurities. These impurities had lower vapor pressure and dominated the headspace early in the measurement. The spectrum of the target molecule, (2Z)-2-buten-1-yl-benzene, didn't appear until after approximately 20,000 FID acquisitions. Final data collection began once the target species appeared at its peak signal level. From there, about 100,000 FID acquisitions were possible before the sample was exhausted (about a 1-hour final measurement time). After spectrum acquisition, the reservoir nozzles were heated to 150°C and connected to a dry scroll pump vacuum line overnight. With this protocol, no carryover was detected between samples.

Targeted Measurements

Multiple transitions were identified from the broadband measurements as potential candidates for the enantiomer analysis in a targeted spectrometer. The measurements were performed on BrightSpec's cavity-enhanced Fourier transform microwave (FTMW) spectrometer. A description of the instrument and methodology has been described elsewhere.[26],[27]

For each measurement, the instrument measured the monomer, homochiral complex, and heterochiral complex in sequence. The instrument cycled between these three transitions in multiple iterations to ensure signal stability through the measurement. To generate the final spectra used for the analysis, the raw time domain data from each iteration was co-added before Fourier transformation. The phase of each measurement was corrected by calculating the phase of the excitation pulse and adjusting the phase of the molecular data by the same amount. In both the racemic and enantiopure measurements the monomer was regularly measured to confirm that the analyte was still present. Two pairs of transitions were identified to increase confidence that any detection of weaker enantiomer cannot be attributed to a spectral blend.

2 µL of sample was injected into the PTV to perform the enantiomer analysis. Two different measurement temperatures were used depending on if the samples were prepared with the (*R*)- or (*S*)-allene. For samples prepared with the (*R*)-allene, The PTV was held at 30°C while the transfer line and nozzle were held at 70°C. The samples prepared with the (*S*)-allene had a volatile impurity present and those measurements were performed at a higher temperature to increase the sensitivity of the analyte (PTV/Transfer/Nozzle: 40/80/80°C). For the measurement with racemic chiral tag, (*R,S*)-TFIP, eight iterations of 1,000 FID Average segments were acquired for the homochiral and heterochiral species. For the enantiopure measurements, performed with the (*S*)-TFIP (SynQuest, tag EE = 99.6%), iterations were set up to acquire 400 averages for the dominant enantiomer and 5000 averages for the weaker enantiomer. Multiple iterations were performed and coadded together to increase the sensitivity of the measurement.

Results

A. Assigning the Absolute Configuration

The spectroscopic analysis required to assign the absolute configuration of the isotopically chiral reaction product was performed using broadband MRR spectroscopy.[39],[40] The first step in this process was the identification of the geometries for the complexes between (2Z)-2-buten-1-yl-benzene and the chiral tag, TFIP. These complexes are identified in the spectrum using the undeuterated reaction product.

A quantum chemistry search identified four low-energy isomers of the chiral tag complex. The results are shown in Table 2S and Fig. 4S.

Table 2S. Summary of spectroscopic constants for calculated methyl allyl / TFIP isomers. Complex geometries were optimized at B3LYP GD3BJ / def2-TZVP level of theory in Gaussian16.

Isomer	ΔE (cm^{-1})	A (MHz)	B (MHz)	C (MHz)	μ_a (D)	μ_b (D)	μ_c (D)
1	0.0*	495.370	384.821	280.707	2.76	-0.57	-0.20
2	73.1	501.960	416.384	300.289	2.36	-0.57	-0.12
3	391.3	535.577	369.479	276.209	2.52	1.45	0.11
4	473.9	590.131	299.300	236.138	2.54	-0.07	-0.45

*-880.794611744 hartree

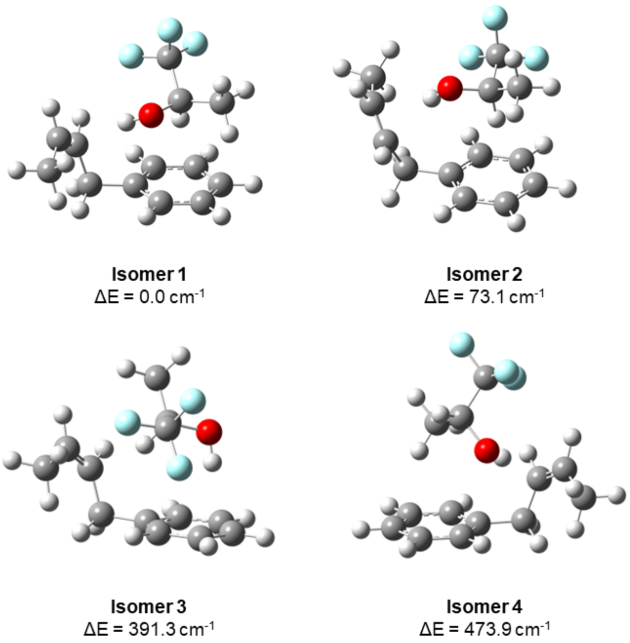

Figure 4S. Methyl allyl / TFIP complex geometries. Geometries were optimized at B3LYP GD3BJ / def2-TZVP level of theory in Gaussian16.

The experimental spectrum, Fig. 5S, is dominated by the MRR spectra of Isomer 1 and Isomer 2. The identification of geometry of the chiral tag complex is made based on agreement between the experimental rotational constants and the rotational constants predicted from the equilibrium geometry (Fig. 4S). It is important to note that (2Z)-2-buten-1-yl-benzene has a low barrier methyl group. All spectra observed in this study show internal rotation splittings. However, since the goal of the work is chiral analysis, we only analyze the A-symmetry spectrum which can be fit with the Watson asymmetric top rotational Hamiltonian. The comparison between experimental and theoretical rotational constants is reported in Table 3S. The full spectrum fit results are provided at the end of the SI report for the MRR analysis. Both spectra are dominated by the a-type transitions – consistent with the dipole moment vector components calculated from theory (and reported in Table 2S). The absolute configuration analysis uses the spectra of both Isomer 1 and Isomer 2 and the structures of these clusters and their equilibrium geometries – reported as the atom positions in the principal axis system for molecular rotation – are given in Figs. 6S and 7S and Tables 4S and 5S below.

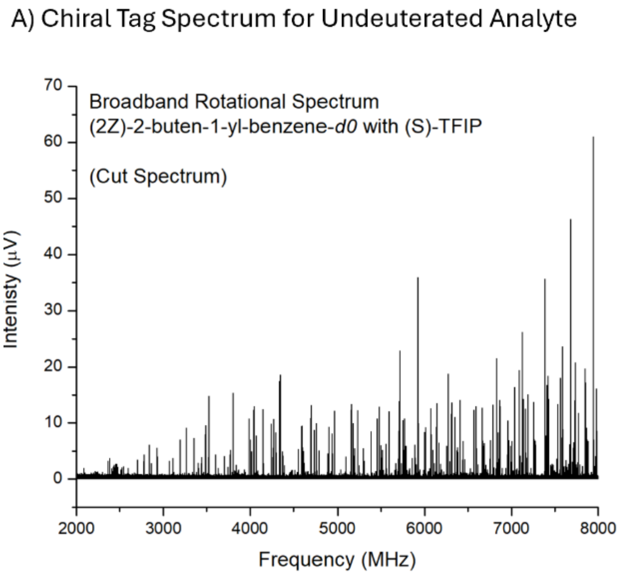

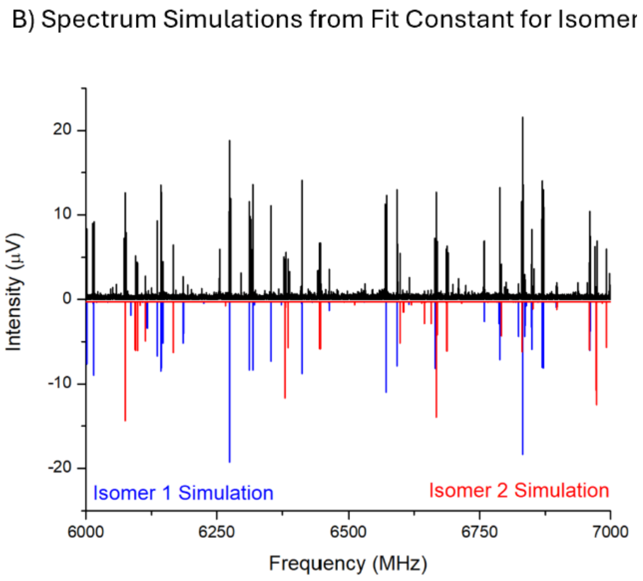

Figure 5S. The broadband MRR spectrum (2-8 GHz) of the complexes formed between the complexes between (2Z)-2-buten-1-yl-benzene-*d0* and TFIP is shown in (A). For this spectrum, the transitions observed in the MRR spectra of the analyte – measured separately – have been cut. Also, transitions observed in a measurement of the spectrum using only the TFIP tag sample have been cut. The spectrum shown in (A) shows the transitions that require the presence of both tag and analyte. The highest intensity transitions in (A) come from the rotational spectra of Isomer 1 and Isomer 2 in Table 1 and Fig.1 A 1 GHz region of the broadband spectrum is shown in (B) where the calculated spectra using the fit rotational constants (A-reduction of the Watson Hamiltonian in the Ir-representation [41]) are shown in the color-coded, negative-going spectra. Careful inspection of the figure shows that additional transitions – not in the calculated spectra – often appear in close frequency proximity to the assigned transitions of the spectrum. These additional transitions are the E-symmetry spectrum for the methyl internal rotation. Analysis of the methyl internal rotation is not needed for the chiral analysis, so only the A-symmetry spectrum is assigned for this work.

Table 3S. The Theoretical and Observed Rotational Constants for the Two Lowest Energy Isomers of the Chiral Tag Complex

Constant	Isomer 1 Theory	Isomer 1 Experiment[(a)]	Percent Error	Scale Factor	Isomer 2 Theory	Isomer 2 Experiment[(a)]	Percent Error	Scale Factor
A (MHz)	495.370	494.86010(34)	0.1	0.9990	501.960	498.86144(33)	0.6	0.9938
B (MHz)	384.821	379.67517(11)	1.4	0.9866	416.384	410.75985(14)	1.4	0.9865
C (MHz)	280.708	278.493011(64)	0.8	0.9921	300.289	296.129976(67)	1.4	0.9862
N_{trans}[(b)]		95				73		
σ (kHz)[(c)]		2.53				2.01		

(a) The 1σ errors in the last two digits of the experimental fit rotational constants are given in parentheses. The full fit results, including distortion constants, are attached at the ends of the MRR SI section.

(b) Number of transitions in the fit.

(c) Root-mean-squared frequency error for the fit. The experimental line width is about 70 kHz (full width at half maximum).

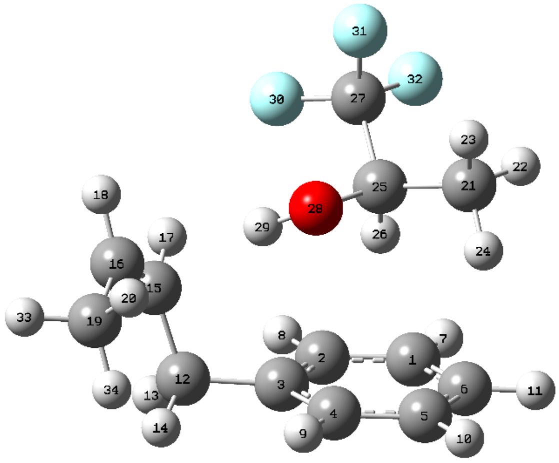

Figure 6S. Labeled optimized geometry of methyl allyl / TFIP Isomer 1 (B3LYP GD3BJ / def2-TZVP).

Table 4S. Principal axis coordinates of optimized geometry methyl allyl / TFIP isomer 1 (B3LYP GD3BJ / def2-TZVP).

Label	Atom	a-coordinate (Å)	b-coordinate (Å)	c-coordinate (Å)
1	C	1.29795	-2.64307	1.02783
2	C	1.77492	-1.38523	1.37643
3	C	2.22083	-0.49427	0.40070
4	C	2.17849	-0.89187	-0.93455
5	C	1.70817	-2.15045	-1.28737
6	C	1.26410	-3.03010	-0.30700
7	H	0.95334	-3.32057	1.79833
8	H	1.80057	-1.09098	2.41899
9	H	2.50787	-0.20645	-1.70558
10	H	1.68419	-2.44226	-2.32941
11	H	0.89348	-4.00918	-0.58077
12	C	2.68906	0.89508	0.77460
13	H	3.22569	0.84757	1.72671
14	H	3.39704	1.25456	0.02929
15	C	1.53173	1.84571	0.92104
16	C	1.25275	2.90637	0.16235
17	H	0.84905	1.61184	1.73198
18	H	0.36451	3.47673	0.41941
19	C	2.01602	3.41621	-1.01941
20	H	1.37912	3.42674	-1.90826
21	C	-1.93429	-1.16882	-1.74768
22	H	-2.39726	-2.04303	-1.29137
23	H	-2.68556	-0.62013	-2.31521
24	H	-1.15332	-1.50105	-2.42921
25	C	-1.31073	-0.27178	-0.69703
26	H	-0.54058	-0.81960	-0.14738
27	C	-2.33344	0.15814	0.35458
28	O	-0.77161	0.86419	-1.33573
29	H	-0.14642	1.29742	-0.73449
30	F	-1.77798	1.00256	1.25588
31	F	-3.39780	0.78809	-0.16931
32	F	-2.79399	-0.90675	1.04752
33	H	2.33786	4.44773	-0.85242
34	H	2.89822	2.81996	-1.24575

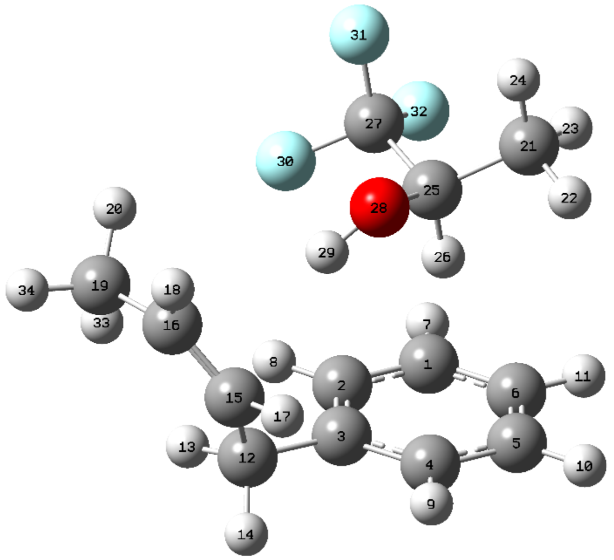

Figure 7S. Labeled optimized geometry of methyl allyl / TFIP Isomer 2 (B3LYP GD3BJ / def2-TZVP).

Table 5S. Principal axis coordinates of optimized geometry methyl allyl / TFIP isomer 2 (B3LYP GD3BJ / def2-TZVP).

Label	Atom	a-coordinate (Å)	b-coordinate (Å)	c-coordinate (Å)
1	C	1.00854	-2.09096	1.41302
2	C	1.54944	-0.81260	1.33556
3	C	2.22523	-0.38944	0.19306
4	C	2.34125	-1.27581	-0.87850
5	C	1.79812	-2.55210	-0.80819
6	C	1.13099	-2.96530	0.34056
7	H	0.48201	-2.39802	2.30706
8	H	1.43768	-0.13239	2.17051
9	H	2.86305	-0.96493	-1.77576
10	H	1.89784	-3.22616	-1.64928
11	H	0.70714	-3.95933	0.39655
12	C	2.79513	1.01046	0.10760
13	H	2.82898	1.44509	1.10525
14	H	3.82893	0.95328	-0.24680
15	C	2.01592	1.89140	-0.83235
16	C	1.22339	2.91335	-0.50692
17	H	2.11805	1.64625	-1.88541
18	H	0.73889	3.44376	-1.32234
19	C	0.90888	3.43048	0.86040
20	H	-0.16598	3.37410	1.04807

21	C	-2.25041	-1.24718	-1.76911
22	H	-1.67473	-1.59701	-2.62467
23	H	-2.60560	-2.11067	-1.20763
24	H	-3.10677	-0.68011	-2.13319
25	C	-1.36271	-0.36787	-0.91048
26	H	-0.49295	-0.93380	-0.56987
27	C	-2.08186	0.08220	0.36144
28	O	-0.97533	0.75873	-1.66543
29	H	-0.21713	1.18504	-1.23792
30	F	-1.30719	0.92158	1.08851
31	F	-3.23387	0.72900	0.11481
32	F	-2.37296	-0.97095	1.15657
33	H	1.41359	2.87752	1.65033
34	H	1.19165	4.48330	0.94586

Deuteration at either of the allyl positions creates an isotopically chiral (2Z)-2-buten-1-yl-benzene-*d1* molecule.[39],[40] When the analyte is complexed with TFIP, two diastereomeric chiral tag complexes are formed that can be distinguished by rotational spectroscopy. These complexes are denoted as homochiral or heterochiral based on the Cahn-Ingold-Prelog designations of the chiral centers in the analyte and tag. For spectroscopy analysis of these complexes, a measurement is first performed using a racemic TFIP tag sample so that both homochiral and heterochiral complexes are guaranteed to be generated regardless of the enantiopurity of the analyte. In this work, this racemic tag measurement was performed using the sample prepared with the (R)-enantiomer of the allene reagent and the (R)-Ph-Garphos ligand. In this spectrum, shown in Fig. 8S, the homochiral and heterochiral tag complexes for both Isomer 1 and Isomer 2 were identified. The assignment of these spectra was guided by predictions of the rotational constants from the equilibrium geometry. The calculation of the predicted rotational constants has two steps. First, the rotational constants for the equilibrium geometry of the homochiral and heterochiral complex are calculated simply by changing the mass of the allylic hydrogen atom that is substituted (atoms 13 and 14 in the structures shown above). Second, the equilibrium rotational constants are multiplied by a correction scale factor – simply the ratio of the experimental rotational constant to its theory prediction - obtained from the analysis of the undeuterated complexes of Isomer 1 and Isomer 2. These scale factors are reported in Table 3S. The comparison between the scaled predicted rotational constants and the experimental fit constants is given in Tables 6S and 7S for the heterochiral and homochiral tag complexes, respectively.

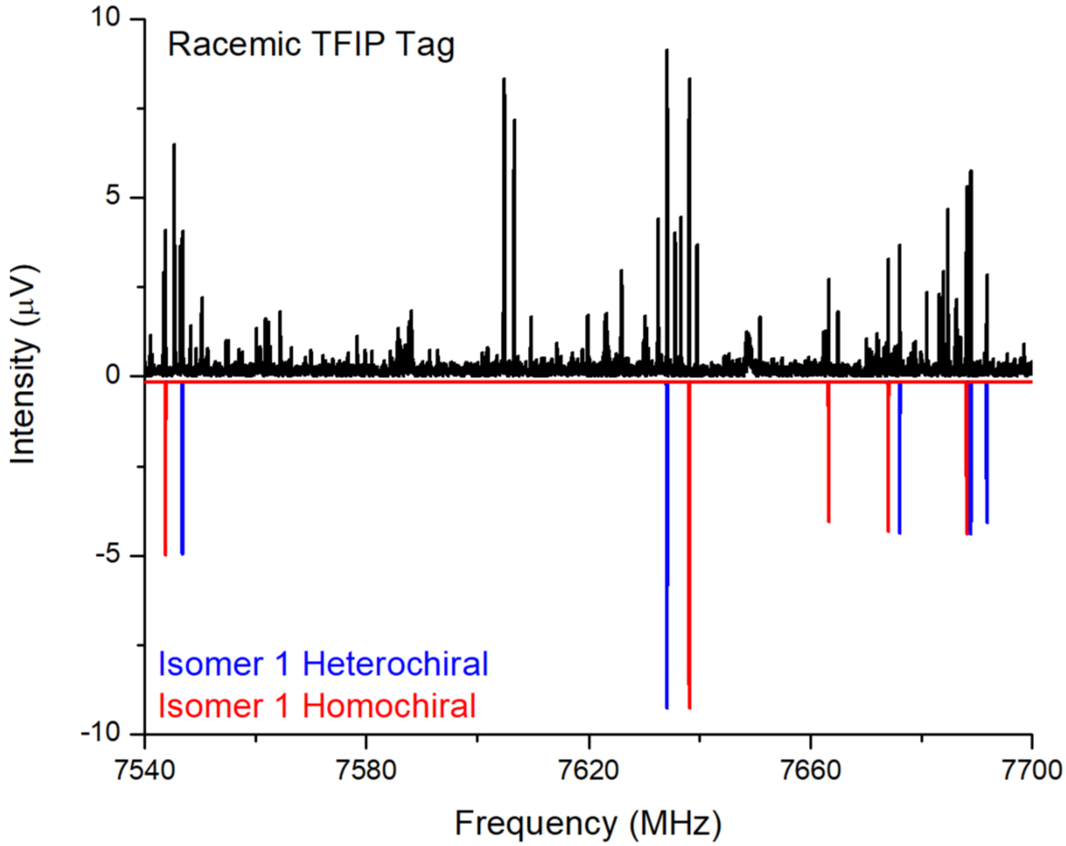

Figure 8S. A small frequency region of the 2-8 GHz broadband spectrum of the (2Z)-2-buten-1-yl-benzene-*d1*analyte prepared in a reaction using a chiral allene sample ((R)-configuration) and the (R)-Ph-Garphos ligand using a neon carrier gas mixture containing 0.1% of racemic TFIP is shown. In this spectrum, the transitions observed in the spectrum of the tag are cut. However, the transitions for the (2Z)-2-buten-1-yl-benzene-*d1* remain in the spectrum. (The two transitions near 7605 MHz are assigned to the monomer of the analyte). The color-coded, negative-going spectra are simulations of the heterochiral and homochiral complexes of Isomer 1 with TFIP calculated using the fit constants for the A-species of complex. As for Fig. 5S, strong transitions near the assigned spectra are observable and are attributed to the E-species spectrum resulting from methyl group internal rotation.

Table 6S: The Theoretical and Observed Rotational Constants and Atom Position Coordinates for the Heterochiral and Homochiral Tag Complexes of Deuterated Isomer 1

Constant	Theory [(a)] Heterochiral	Experiment 1[(b)]	Percent Error	Theory [(a)] Homochiral	Experiment 2 [(b)]	Percent Error
A (MHz)	494.125	494.25746(44)	0.027	493.099	494.01988(37)	0.186
B (MHz)	376.344	376.467026(79)	0.033	375.830	375.252631(74)	-0.154
C (MHz)	276.475	276.604289(55)	0.047	276.772	276.908719(53)	0.049
N_{trans} [(c)]		93			80	
σ (kHz) [(d)]		4.95			4.98	
|a| (Å) [(e)]	3.397	3.343		3.226	3.487	
|b| (Å)	1.254	1.089		0.848	nd[(f)]	
|c| (Å)	0.029	0.316		1.727	1.885	

(a) The theoretical rotational constant predictions include correction of the calculated rotational constants using the equilibrium geometry using the scale factors reported in Table (6S).

(b) The 1σ errors in the last two digits of the experimental fit rotational constants are given in parentheses. The centrifugal distortion constants are fixed at the values of the *d0*-complex.

(c) Number of transitions in the fit.

(d) Root-mean-squared frequency error for the fit. The experimental line width is about 70 kHz (full width at half maximum).

(e) The atom coordinates for the substituted hydrogen atom in the principal axis system for the *d0*-complex.

(f) This coordinate could not be determined due to inertial defect issues. The Kraitchman formulas return the unphysical value of 1.355i Å.

Table 7S: The Theoretical and Observed Rotational Constants and Atom Position Coordinates for the Heterochiral and Homochiral Tag Complexes of Deuterated Isomer 2

Constant	Theory [(a)] Heterochiral	Experiment 1[(b)]	Percent Error	Theory [(a)] Homochiral	Experiment 2 [(b)]	Percent Error
A (MHz)	498.411	499.58485(55)	0.235	497.269	497.26646(51)	0.001
B (MHz)	405.807	404.79660(24)	-0.235	407.630	407.75612(27)	0.031
C (MHz)	293.409	293.425435(76)	0.005	294.358	294.420925(74)	0.021
N_{trans} [(c)]		44			44	
σ (kHz) [(d)]		4.63			4.29	
|a| (Å) [(e)]	3.829	4.216		2.829	2.785	
|b| (Å)	0.953	nd[(f)]		1.445	1.453	
|c| (Å)	0.247	0.685		1.105	1.104	

(a) The theoretical rotational constant predictions include correction of the calculated rotational constants using the equilibrium geometry using the scale factors reported in Table (7S).

(b) The 1σ errors in the last two digits of the experimental fit rotational constants are given in parentheses. The centrifugal distortion constants are fixed at the values of the *d0*-complex.

(c) Number of transitions in the fit.

(d) Root-mean-squared frequency error for the fit. The experimental line width is about 70 kHz (full width at half maximum).

(e) The atom coordinates for the substituted hydrogen atom in the principal axis system for the *d0*-complex.

(f) This coordinate could not be determined due to inertial defect issues. The Kraitchman formulas return the unphysical value of 1.442i Å.

Two additional metrics are used to assess the confidence in the assignment of the homochiral and heterochiral tag complexes to the observed MRR spectra. The first one, reported in the main manuscript, looks at the changes in the principal moments-of-inertia upon deuterium substitution. The rotational constants and principal moments-of-inertia are inversely related. The experimental value for this change is obtained using the fit rotational constants for Isomer 1 and Isomer 2 spectra of the undeuterated analyte reported in Table 3S and the fit constants for the two deuterated forms reported in Table 6S. Based on changes to the spectrum intensities when enantiopure (*S*)-TFIP is used as the tag for the deuterated analytes, the spectra from Isomer 1 and Isomer 2 that share the homochiral and heterochiral structures can be identified. These two cases are denoted Experiment 1 and Experiment 2. The comparison between the experimental and theoretical changes in the moments-of-inertia are shown in Fig. 9S where the homochiral and heterochiral designations are made based on agreement in the predcited patterns for the changes.

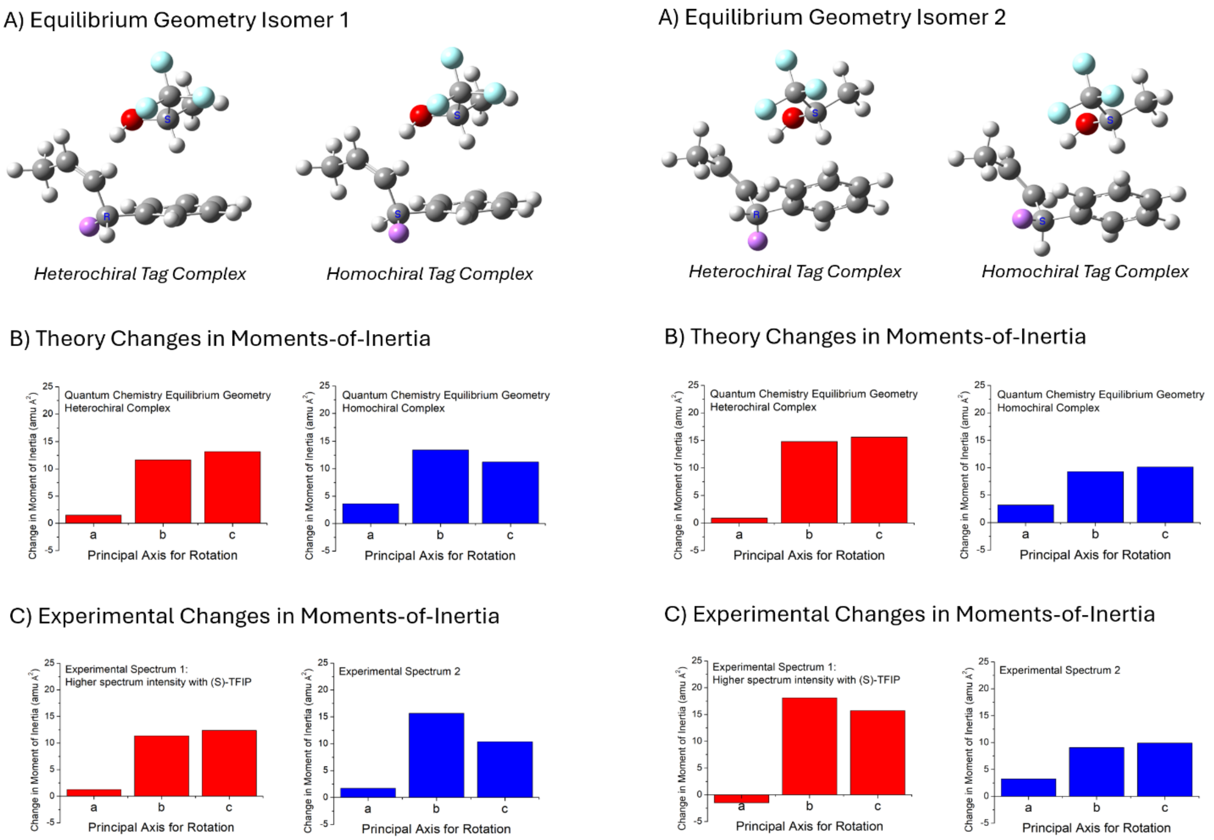

Figure 9S. The assignments of the experimental spectra to homochiral and heterochiral complexes are made based on comparison of the predicted rotational constants using the equilibrium geometry and the experimental constants. One way to visualize this information is to examine the changes in the principal moments-of-inertia of the complexes upon deuteration. These changes are calculated using the rotational constants of the undeuterated analyte complexes, Table 3S, and the deuterated complexes, Tables 6S and 7S. The analysis is performed using both observed isomers. From the intensity changes when enantiopure (*S*)-TFIP tag is used, it is known that the Experiment 1 spectra for both isomers share the same heterochiral/homochiral designation (with the Experiment 2 spectra having the opposite designation). Note that the Isomer 2 results are proetid in the figure in the main manuscript. For all samples prepared using the (R)-enantiomer of the allene reagent, the heterochiral complex has higher transition intensity when (S)-TFIP is used as the tag. This indicates that all reaction products were (*R*)-(2Z)-2-buten-1-yl-benzene-*d1*.

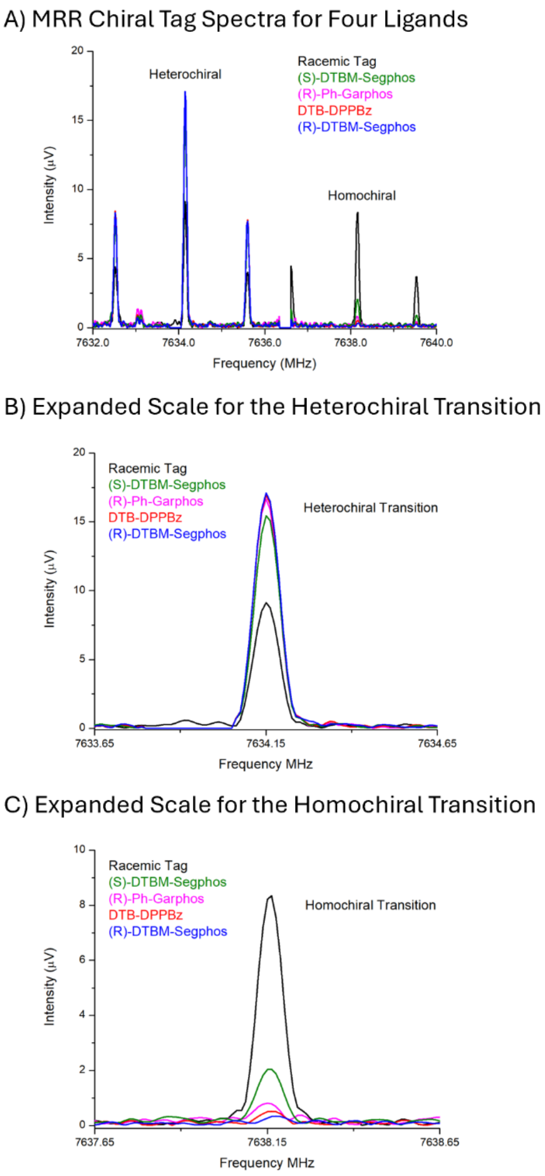

Figure 10S. An expanded scale view of a heterochiral / homochiral transition pair for the Isomer 1 complex is shown for the four samples prepared with the (*R*)-enantiomer of the chiral allene reagent. These spectra are scaled so that the sum of the intensity of the heterochiral and homochiral transitions are the same. In all cases, the heterochiral transition intensity is higher when (*S*)-TFIP is used as the tag. For all four samples, (*R*)-(2Z)-2-buten-1-yl-benzene-*d1*is the higher abudnance enantiomer. The different signal levels of the homochiral transition, panel (C), indicates variable enantiomeric excess in the samples.

The second way to assess the confidence in the assignment of the spectra to the homochiral and heterochiral structures is through a comparison of the atom positions calculated from the experimental spectrum using Kraitchman's method [41],[42] and the equilibrium geometry positions reported in Tables 6S and 7S. The Kraitchman analysis only provides the magnitude of the atom coordinates. Also, the effects of vibrational zero-point motion can cause some atom position determinations to return unphysical results (showing up as imaginary coordinate values when the Kraitchman formulas are used).[41] The magnitude atom coordinates are reported in Tables 6S and 7S. Overall, the spectrum analysis gives high confidence that the rotational spectra are correctly assigned to the homochiral and heterochiral complexes between TFIP and (2Z)-2-buten-1-yl-benzene-*d1*.

With the assignment of the homochiral and heterochiral cluster geometries to the experimental MRR spectra, the determination of the absolute configuration of the enantiomer of the analyte present in higher abundance is straightforward. The chiral tag spectrum is measured using (*S*)-TFIP as the tag. If the homochiral spectrum has higher intensity, then the analyte has the absolute configuration (*S*)-(2Z)-2-buten-1-yl-benzene-*d1*. Broadband MRR spectra were measured for four synthetic methods that all used the (*R*)-enantiomer of the chiral allene but different ligands on the catalyst. A small section of the broadband spectrum for these four measurements is shown in Fig. 10S. In all cases, the intensity of the heterochiral spectrum is higher. Therefore, the absolute

configuration of the higher abundance enantiomer is (*R*)-(2Z)-2-buten-1-yl-benzene-*d1* in all sample preparations. A second set of samples were prepared using the (*S*)-enantiomer of the chiral allene. For these samples, the absolute configuration of the higher abundance enantiomer was assigned using IsoMRR measurements that monitored the homochiral and heterochiral MRR transitions indicated in Fig. 10S. For all of these analytes, the absolute configuration is (*S*)-(2Z)-2-buten-1-yl-benzene-*d1*.

B. Measurement of the Enantiomeric Excess

Broadband MRR Spectroscopy

Table 7. Heterochiral and Homochiral Rotational Transitions used for the Enantiomeric Excess Measurements in the Broadband Spectrum Measurements

Transition Frequencies for the Heterochiral Structure of Isomer 1 (MHz)	Transition Frequencies for the Homochiral Structure of Isomer 1 (MHz)
4564.7500	4560.6250
5126.3000	5130.4750
5147.0375	5144.2375
5679.4500	5684.2375
6232.6000	6237.9875
6785.7375	6791.7375
7338.8625	7345.4750
7546.8750	7543.8375
7634.1500[(a)]	7638.1625[(a)]
7688.9750	7688.2125
7950.8625	7943.9250

[(a)] These transitions are used in the IsoMRR EE measurements

Broadband MRR spectra are available for the four analytes prepared using the (*R*)-enantiomer of the chiral allene. Enantiomeric excess determinations use the methods reported previously. [39],[40] For broadband EE measurements, pairwise EE detemrinations from a set of homochiral and heterochiral transitions are used. The transitions of the Isomer 1 complexes used in the EE measurement are given in Table 7. There are 11 transitions for each spectrum, therefore, there are 121 pairwise EE determinations. These 121 detemrinations are reported as a histogram as shown in Fig. 11S. The mean value of all 121 pairwise determinations is reported as the EE value. The measurement uncertainty is estimated by the standard error.

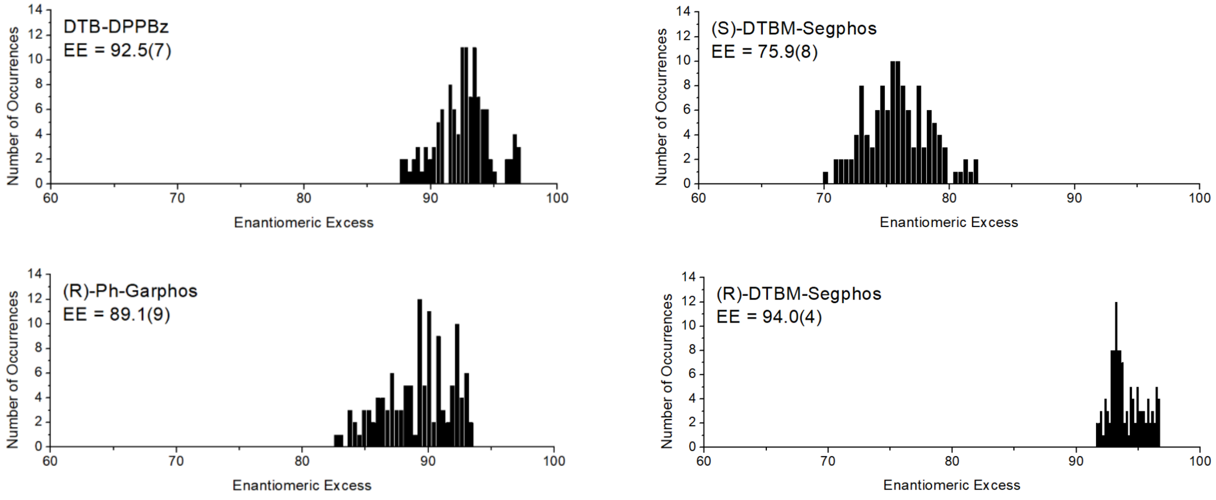

Figure 11S. The enantiomeric excess measurements using broadband rotational spectroscopy of the four samples prepared using the (*R*)-enantiomer of the allene reagent are shown. The measurement results are given in the inset which include the standard error in the last digit given in parenthesis.

IsoMRR Measurements

The enantiomeric excess for the full set of samples, including reaction products when the (*S*)-enantiomer of the allene reagent is used, were measured using cavity-enhanced detection in the BrightSpec IsoMRR instrument which offers reduced measurement time and sample consumption.[43] The EE determinations are reported in Table 8S – which also includes the results from the broadband spectrum analysis shown in Fig. 11S. The EE determinations from the two MRR spectroscopy techniques are in good agreement. More detailed information for the IsoMRR measurements is given in Table 9S. Finally, example spectra form the IsoMRR measurements are shown in Fig. 12S.

Measurement Setup:

Transitions used in analysis: $13_{1\,12} – 12_{1\,11}$ (set of degenerate transitions is monitored)

$13_{2\,12} – 12_{2\,11}$

Heterochiral: 7634.15 MHz

Homochiral: 7638.16 MHz

Table 8S. Summary of enantiomer analysis performed on (2Z)-2-buten-1-yl-benzene-*d1* samples prepared by using different ligands and different stereoisomers of the allene precursor.

Allene AC	Ligand	AC	Broadband	IsoMRR*
R	DTB-DPPBz	R	93(1)	90.9
R	R-Ph-GARPHOS	R	89(1)	86.6
R	S-DTBM-SEGPHOS	R	76(1)	72.5
R	R-DTBM-SEGPHOS	R	>94(0.4)**	95.8
S	DTB-DPPBz	S	-	91.6
S	R-Ph-GARPHOS	S	-	74.8
S	S-DTBM-SEGPHOS	S	-	92.7
S	R-DTBM-SEGPHOS	S	-	78.9

* Based on other measurements with the IsoMRR in this EE range, the expected measurement uncertainty for the enantiomeric excess is +/- 1 as determined from the standard deviation of replicate measurements.

** This sample gives the highest EE. The transition intensity of the homochiral transitions used for broadband EE determination are below 3:1 signal-to-noise ratio. Experience with measurements below the limit of quantitation (10:1 signal-to-noise ratio) shows that the value reported from the histogram analysis is a lower limit.

Table 9S. Raw data of targeted measurements used to determine the enantiomeric excess (EE) of the prepared samples.

Racemic Measurement (Instrument Response)		
Complex	Averages	Intensity (μV)
Homochiral	8000	103.9351
Heterochiral	8000	87.3845
	Ratio	1.1894
R-Allene, DTB-DPPBz		
Complex	Averages	Intensity (μV)
Homochiral	2000	89.01398
Heterochiral	25000	3.735987
	Ratio	23.82609
	EE (%)	-90.9*
R-Allene, R-Ph-GARPHOS		
Complex	Averages	Intensity (μV)
Homochiral	1600	91.67942
Heterochiral	20000	5.6759
	Ratio	16.1524
	EE (%)	-86.6
R-Allene, S-DTBM-SEGPHOS		
Complex	Averages	Intensity (μV)
Homochiral	1200	53.92889
Heterochiral	15000	7.303396
	Ratio	7.384084
	EE (%)	-72.5
R-Allene, R-DTBM-SEGPHOS		
Complex	Averages	Intensity (μV)
Homochiral	4400	110.52
Heterochiral	55000	2.1886
	Ratio	50.49804
	EE (%)	95.8
S-Allene, DTB-DPPBz		
Complex	Averages	Intensity (μV)
Homochiral	40000	7.38
Heterochiral	3200	134.7583
	Ratio	0.054765
	EE (%)	91.6
S-Allene, R-Ph-GARPHOS		
Complex	Averages	Intensity (μV)
Homochiral	15000	22.57046
Heterochiral	1200	129.6221
	Ratio	0.174125
	EE (%)	74.8
S-Allene, S-DTBM-SEGPHOS		
Complex	Averages	Intensity (μV)
Homochiral	45000	6.2849
Heterochiral	3600	132.7795
	Ratio	0.047333
	EE (%)	92.7
S-Allene, R-DTBM-SEGPHOS		

Complex	Averages	Intensity (μV)
Homochiral	15000	24.29693
Heterochiral	1200	170.5327
	Ratio	0.142477
	EE (%)	78.9

* The sign of the EE determination denotes which enantiomer is in excess. The convention used in this table is that for EE > 0, the (*S*)-enantiomer of the reaction product is in excess.

(A) Sample Prepared with (*R*)-allene and DTB-DPPBz Ligand

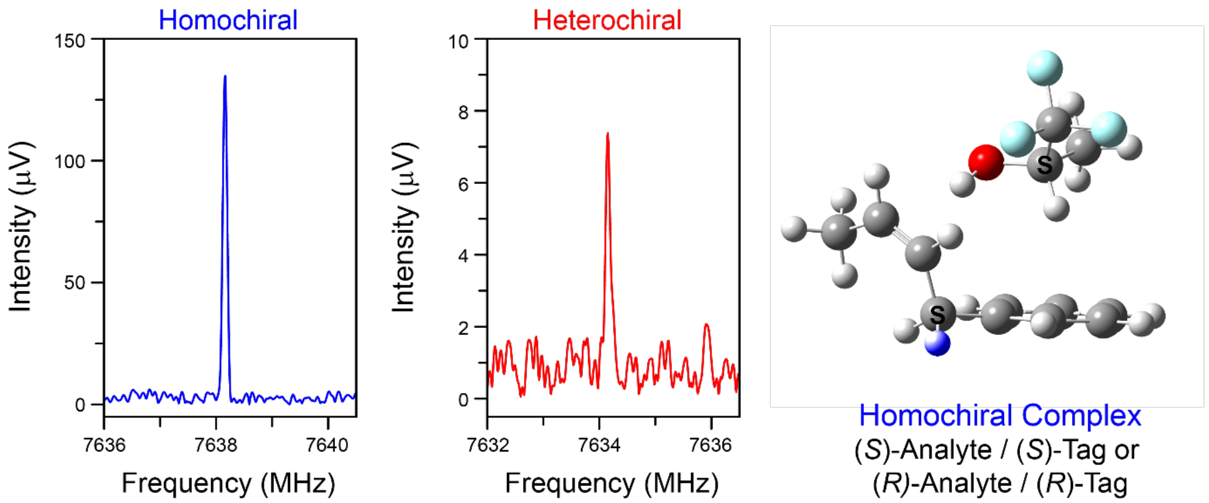

(B) Sample Prepared with (*S*)-allene and DTB-DPPBz Ligand

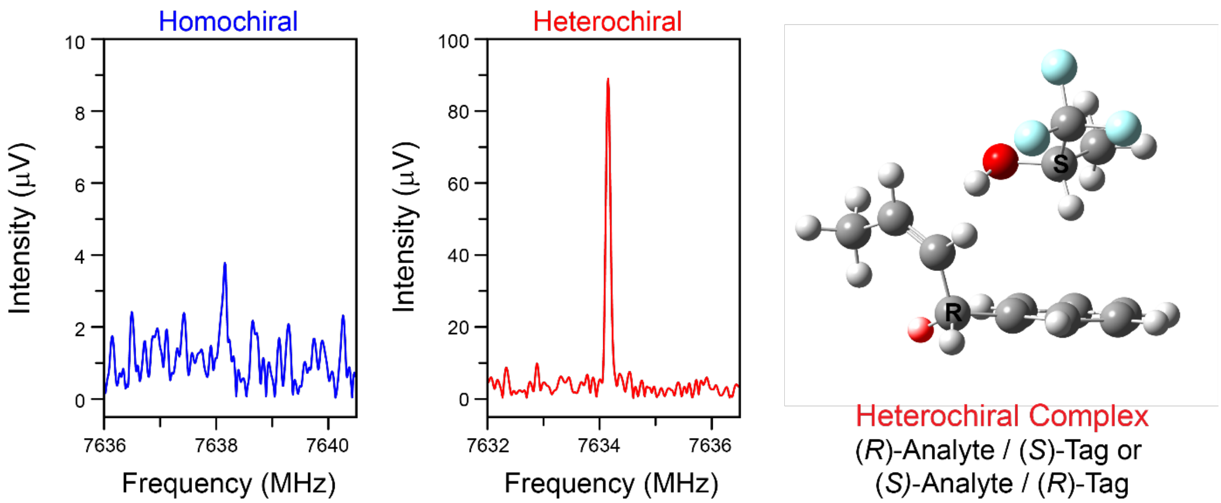

Figure 12S. Enantiomer analysis of methyl-allyl-d1 prepared with the same ligand and different stereoisomers of the allene precursor showing the flip in stereochemistry. Both samples were analyzed using S-TFIP (tag EE = 99.6%).

C. E/Z Isomer Ratio for the Monomer

Rotational spectroscopy was also used to verify that the Z-isomer of 2-buten-1-yl-benzene-*d1*is produced in the synthesis. This analysis also provided an estimate that the isomer ratio (Z:E) is greater than 10:1 in the sample.

The identification of the isomers was first made in a sample of 2-buten-1-yl-benzene-*d0*. The rotational spectrum of this sample is shown in Fig. 13S. Both isomers are observed with an intensity ratio of approximately 7.5:1 (E:Z). The comparison between the theoretical and experimental rotational constants is shown in Table 10S and used to assign the isomer geometry to each spectrum. Evidence for tunneling of the methyl group is observed in the spectrum of (Z)-2-buten-1-yl-benzene. The internal rotation splits each transition into a doublet. The component of the doublet with A-symmetry can be fit with a standard rotational Hamiltonian and only analysis of the A-symmetry spectrum is report. Note that because each transition is a doublet, the total spectrum intensity of the Z:E isomers is approximately 15:1. Also, the calculated dipole moments are about equal for the isomers (μ_a = 0.43D for Z and μ_a = 0.40D for E) so that the total intensity ratio is a good estimate of the population ratio. The 14:1 Z:E ratio is in good agreement with the NMR analysis of this sample which gives a 12:1 isomer ratio.

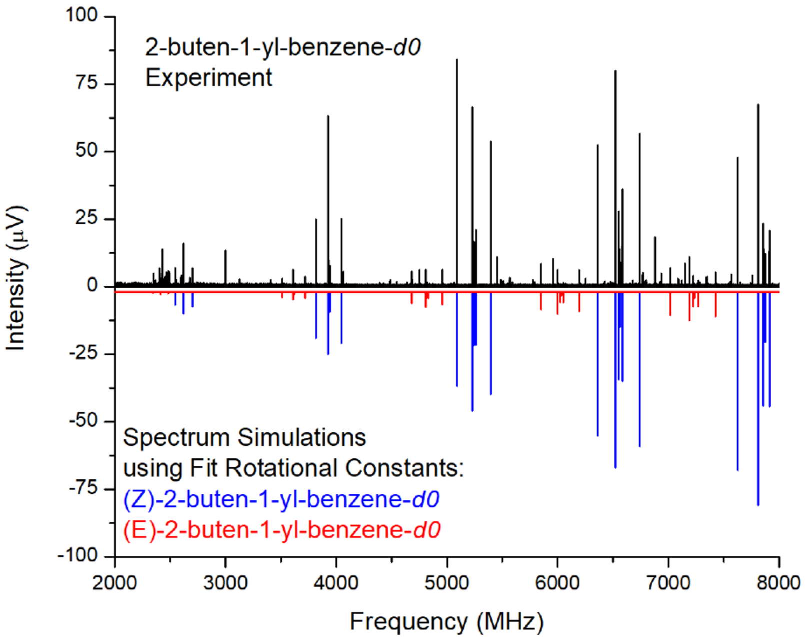

Figure 13S: The broadband rotational spectrum of 2-buten-1-yl-benzene-*d0* is shown (black). Both E and Z stereoisomers of the analyte are detected in the spectrum. The spectrum simulation for (E)-2-buten-1-yl-benzene-*d0* (red) has a factor of 7.5 reduction compared to the (Z)-2-buten-1-yl-benzene-*d0* spectrum simulation (blue).

Table 10S: The theoretical and experimental rotational constants for (Z)-2-buten-1-yl-benzene-*d0* and (E)-2-buten-1-yl-benzene-*d0* are reported. The 1σ standard errors for the experimental parameters are reported in parentheses. The theoretical rotational constants are obtained from the equilibrium geometry using dispersion corrected DFT: B3LYP GD3BJ def2TZVP

Constant	Z-Isomer Exp	Z-Isomer Theory	Percent Error	E-Isomer Exp	E-Isomer Theory	Percent Error
A (MHz)	3368.05(36)	3375.07	-0.21	3460.28(48)	3508.08	-1.38
B (MHz)	693.8465(10)	698.62	-0.69	636.8905(10)	639.13	-0.35
C (MHz)	617.4481(1)	620.34	-0.47	568.2023(10)	568.46	-0.05
D_J (kHz)	0.318(12)			0.115(13)		
D_{JK} (kHz)	-3.87(10)			-1.111(62)		
$N_{transitions}$	24			21		
RMS (kHz)	8.23			8.69		

The analysis of the 2-buten-1-yl-benzene-*d1*sample produced using the (R)-Ph GARPHOS ligand has two challenges. First, the sample from the synthesis has a significant amount of solvent. In the measurement, the spectrum is dominated by two different solvents with the analyte eventually emerging. The experimental spectra acquired during the measurement are shown below in Fig. 14S. The spectrum of the analyte is isolated by cutting the peak positions of the early-eluting solvents and is shown in panel (D) of Fig. 14S.

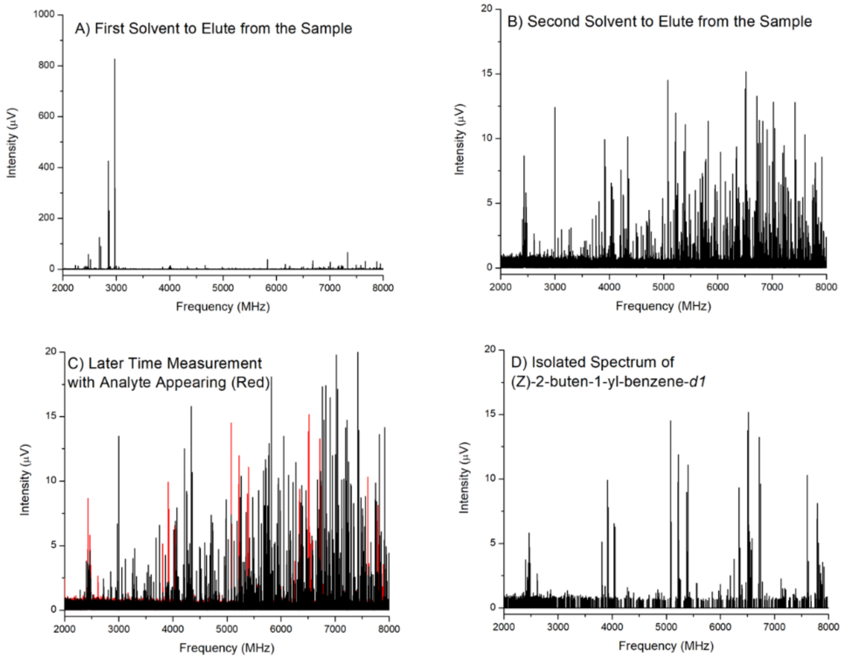

Figure 14S: Measurement of the 2-buten-1-yl-benzene-*d1* rotational spectrum is shown. Two solvents are found to elute from the sample at early times in the measurement as shown in panels (A) and (B). At later times, the spectrum of the analyte emerges as seen in panel (C). The isolated spectrum of the analyte is shown in panel (D).

The second challenge is that the analyte has two distinct spectra when deuterated since the two conformational enantiomers are no longer equivalent. The two distinct geometries of (Z)- 2-buten-1-yl-benzene-*d1* are shown in Fig. 15S.

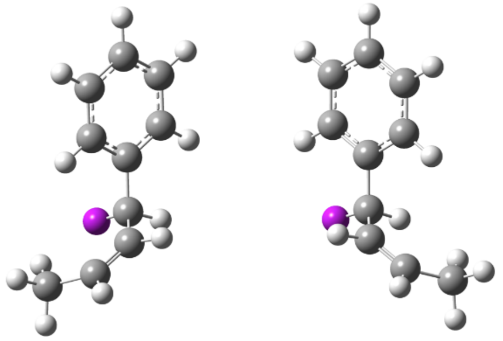

Figure 15S: The two isomers of (Z)- 2-buten-1-yl-benzene-*d1* are shown. The overall structure shows the two equivalent conformations of the molecule. In the absence of deuteration, these conformers are enantiomers and have identical rotational spectra. However, upon deuteration, the hydrogen postion shown in magenta, these structures are inequivalent and tow rotational spectra are observed.

The comparison between the theoretical and experimental rotational constants of the two isomers is shown in Table 11S. In this case, the theoretical rotational constants include a scale factor correction based on the results from the *d0*-sample reported in Table 10S. In the spectrum fitting process, the centrifugal distortion constants (Δ_J and Δ_{JK}) are fixed at the values of the *d0*-sample reported in Table 10S. Also, as for the *d0*-species, only the A-symmetry spectrum from methyl internal rotation of each isomer is analyzed. The spectrum analysis results are shown in Fig. 16S. Note that we do not observe a spectrum for the other stereoisomer, (E)- 2-buten-1-yl-benzene-*d1,* at the signal-to-noise ratio of this measurement. The lack of observation of this isomer indicates that the Z:E ratio is >10:1. The ratio measured by NMR spectroscopy is 11.8:1 consistent with the lack of detection.

Table 11S: The experimental and theoretical rotational constants of the two isomers of (Z)- 2-buten-1-yl-benzene-*d1* (see Fig. 15S) are reported. The 1s standard errors for the experimental parameters are reported in parentheses. The theoretical rotational constants are obtained from the equilibrium geometry using dispersion corrected DFT: B3LYP GD3BJ def2TZVP

Constant	Isomer 1 Exp	Isomer 1 Theory	Percent Error	Isomer 2 Exp	Isomer 2 Theory	Percent Error
A (MHz)	3255.70(42)	3267.6	-0.37	3312.36(47)	3316.3	-0.12
B (MHz)	695.3915(14)	693.0	0.34	691.4448(13)	690.4	0.15
C (MHz)	614.8055(13)	610.6	0.68	616.0983(13)	612.6	0.57
$N_{transitions}$	25			23		
RMS (kHz)	13.9			13.2		

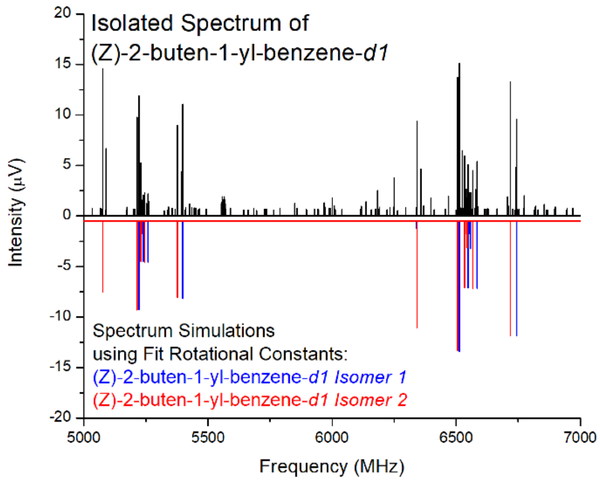

Figure 16S: The isolated rotational spectrum of (Z)- 2-buten-1-yl-benzene-*d1* (see Fig. 14S(D)) is shown with the simulated spectra of the two isomers calculated using the experimental rotational constants reported in Table 11S. This figure uses an expanded frequency scale so that the two spectra can be more easily seen.

The fit results for chiral tag complexes used in the analysis are reported below. The fit was performed using the SPFIT program [44] with output reformatted using the PIFORM routine of Kisiel.[45]

Fit Results for Isomer 1 of (2Z)-2-buten-1-yl-benzene-*d0* complexed with TFIP

```
---------------------------------------------------------------------------
---------==========
                                             obs         o-c     error
blends     Notes

o-c      wt
    / instead of : below denotes (o-c)>3*err
---------------------------------------------------------------------------
---------==========
   1:  4  0  4    3  0  3                    2383.9827  -0.0027  0.001
   2:  5  0  5    4  0  4                    2935.6487   0.0000  0.001
   3:  6  0  6    5  0  5                    3490.7642  -0.0019  0.001
   4/  7  0  7    6  0  6                    4047.1699   0.0032  0.001
   5:  8  0  8    7  0  7                    4603.9531   0.0023  0.001
   6/  9  0  9    8  0  8                    5160.8301  -0.0101  0.001
   7: 10  0 10    9  0  9                    5717.7537  -0.0024  0.001
   8: 11  0 11   10  0 10                    6274.6752  -0.0006  0.001
   9: 12  0 12   11  0 11                    6831.5911  -0.0007  0.001
  10: 13  0 13   12  0 12                    7388.5009   0.0001  0.001
  11: 14  0 14   13  0 13                    7945.4007   0.0002  0.001
  12:  4  1  4    3  1  3                    2369.3793   0.0006  0.001
  13/  4  1  3    3  1  2                    2707.4396   0.0054  0.001
  14:  5  1  5    4  1  4                    2931.1278   0.0015  0.001
  15:  5  1  4    4  1  3                    3267.1875   0.0000  0.001
  16:  6  1  6    5  1  5                    3489.5356   0.0003  0.001
  17:  6  1  5    5  1  4                    3806.3765  -0.0013  0.001
  18/  7  1  7    6  1  6                    4046.8510  -0.0049  0.001
  19:  7  1  6    6  1  5                    4351.4088  -0.0009  0.001
  20:  8  1  7    7  1  6                    4903.3579  -0.0012  0.001
  21/  9  1  9    8  1  8                    5160.8301   0.0072  0.001
  22:  9  1  8    8  1  7                    5458.4538  -0.0013  0.001
  23: 10  1 10    9  1  9                    5717.7537   0.0015  0.001
  24: 10  1  9    9  1  8                    6014.6781  -0.0018  0.001
  25: 11  1 11   10  1 10                    6274.6752   0.0001  0.001
  26/ 11  1 10   10  1  9                    6571.2958   0.0080  0.001
  27: 12  1 12   11  1 11                    6831.5911  -0.0005  0.001
  28/ 12  1 11   11  1 10                    7128.0216  -0.0091  0.001
  29: 13  1 13   12  1 12                    7388.5009   0.0001  0.001
  30: 13  1 12   12  1 11                    7684.8248  -0.0015  0.001
  31: 14  1 14   13  1 13                    7945.4007   0.0002  0.001
  32:  5  2  4    4  2  3                    3195.8071   0.0026  0.001
  33:  6  2  5    5  2  4                    3774.1869  -0.0010  0.001
  34:  7  2  6    6  2  5                    4339.9933   0.0000  0.001
  35:  7  2  5    6  2  4                    4703.1924   0.0003  0.001
  36:  8  2  7    7  2  6                    4899.8377   0.0002  0.001
```

37:	8	2	6	7	2	5	5233.5301	-0.0011	0.001
38:	9	2	8	8	2	7	5457.4615	0.0023	0.001
39:	9	2	7	8	2	6	5770.8458	-0.0002	0.001
40:	10	2	9	9	2	8	6014.4149	0.0001	0.001
41:	10	2	8	9	2	7	6318.4683	-0.0009	0.001
42:	11	2	9	10	2	8	6871.4784	-0.0017	0.001
43/	12	2	11	11	2	10	7128.0216	0.0075	0.001
44:	12	2	10	11	2	9	7426.6734	-0.0015	0.001
45:	13	2	12	12	2	11	7684.8248	0.0024	0.001
46/	13	2	11	12	2	10	7982.7025	-0.0049	0.001
47:	5	3	3	4	3	2	3353.3560	0.0003	0.001
48:	5	3	2	4	3	1	3522.4846	0.0000	0.001
49:	6	3	4	5	3	3	3988.7913	-0.0010	0.001
50:	7	3	5	6	3	4	4596.9738	0.0000	0.001
51:	5	3	2	4	1	3	4670.4493	0.0005	0.001
52:	7	3	4	6	3	3	4969.1044	0.0004	0.001
53:	8	3	6	7	3	5	5181.6705	0.0010	0.001
54:	8	3	5	7	3	4	5591.9261	0.0024	0.001
55:	6	3	3	5	1	4	5675.7204	-0.0005	0.001
56:	9	3	7	8	3	6	5750.7194	-0.0001	0.001
57:	9	3	6	8	3	5	6143.7482	0.0003	0.001
58:	10	3	8	9	3	7	6311.6945	0.0000	0.001
59:	10	3	7	9	3	6	6665.2287	-0.0019	0.001
60:	11	3	9	10	3	8	6869.4003	0.0009	0.001
61:	11	3	8	10	3	7	7194.0229	-0.0011	0.001
62:	12	3	10	11	3	9	7426.0782	0.0023	0.001
63:	12	3	9	11	3	8	7736.3412	-0.0020	0.001
64/	13	3	11	12	3	10	7982.5476	0.0044	0.001
65:	6	4	2	5	4	1	4156.1618	-0.0022	0.001
66:	7	4	4	6	4	3	4741.7114	0.0001	0.001
67:	7	4	3	6	4	2	4936.7930	0.0002	0.001
68:	8	4	5	7	4	4	5386.4592	0.0003	0.001
69:	6	4	2	5	2	3	5673.3392	0.0018	0.001
70:	8	4	4	7	4	3	5704.3276	-0.0003	0.001
71:	9	4	6	8	4	5	6002.0989	-0.0004	0.001
72:	9	4	5	8	4	4	6412.5370	-0.0013	0.001
73/	7	4	3	6	2	4	6462.5083	-0.0041	0.001
74:	10	4	7	9	4	6	6591.8721	0.0007	0.001
75:	10	4	6	9	4	5	7039.3472	0.0007	0.001
76:	11	4	8	10	4	7	7163.5806	0.0001	0.001
77:	11	4	7	10	4	6	7588.2233	-0.0002	0.001
78:	12	4	9	11	4	8	7725.3934	-0.0008	0.001
79:	7	5	3	6	5	2	4763.3039	-0.0002	0.001
80:	7	5	2	6	5	1	4794.8310	-0.0012	0.001
81:	8	5	4	7	5	3	5457.6558	0.0023	0.001
82:	8	5	3	7	5	2	5556.9404	0.0006	0.001
83:	9	5	5	8	5	4	6136.1186	0.0013	0.001
84:	9	5	4	8	5	3	6353.3711	0.0005	0.001
85:	10	5	6	9	5	5	6788.4904	0.0008	0.001
86:	10	5	5	9	5	4	7137.1803	-0.0003	0.001
87:	11	5	7	10	5	6	7410.4034	0.0002	0.001
88:	8	5	3	7	3	4	7483.4980	0.0009	0.001
89:	11	5	6	10	5	5	7857.3100	0.0001	0.001
90:	9	6	4	8	6	3	6147.5175	0.0006	0.001

```
  91:  9  6  3    8  6  2                        6186.1103   0.0002  0.001
  92: 10  6  5    9  6  4                        6849.1852   0.0014  0.001
  93: 10  6  4    9  6  3                        6960.6760  -0.0008  0.001
  94: 11  6  6   10  6  5                        7534.7743   0.0006  0.001
  95: 11  6  5   10  6  4                        7771.0878  -0.0005  0.001
--------------------------------------------------------------------------
------

PARAMETERS IN FIT (values truncated and Nlines statistics):

        10000            A  /MHz          494.86009(13)
1
        20000            B  /MHz          379.675165(43)
2
        30000            C  /MHz          278.493011(24)
3
          200         DELJ  /kHz            0.05821(16)
4
         1100        DELJK  /kHz            0.1530(10)
5
         2000         DELK  /kHz           -0.1372(52)
6
        40100         delJ  /kHz            0.011359(87)
7
        41000         delK  /kHz            0.10127(75)
8

 MICROWAVE AVG =        0.000014 MHz, IR AVG =       0.00000
 MICROWAVE RMS =        0.002526 MHz, IR RMS =       0.00000
 END OF ITERATION  2 OLD, NEW RMS ERROR=        2.52574         2.52574

  distinct frequency lines in fit:    95
       distinct parameters of fit:     8

                                    upper state  lower state
overall
      limits of quantum number  1:      4   14       3   13           3
14
      limits of quantum number  2:      0    6       0    6           0
6
      limits of quantum number  3:      2   14       1   13           1
14

                  frequency range:        2369         7982


 PARAMETERS IN FIT WITH STANDARD ERRORS ON THOSE THAT ARE FITTED:
 (values rounded and degrees of freedom, Ndegf=Nlines-Nconst, statistics)

        10000            A  /MHz          494.86010(34)
1
        20000            B  /MHz          379.67517(11)
2
```

```
        30000             C  /MHz          278.493011(64)
3
          200          DELJ  /kHz            0.05822(43)
4
         1100         DELJK  /kHz            0.1530(28)
5
         2000          DELK  /kHz           -0.137(13)
6
        40100          delJ  /kHz            0.01136(22)
7
        41000          delK  /kHz            0.1013(19)
8

 CORRELATION COEFFICIENTS, C.ij:

           A        B        C        -DELJ    -DELJK   -DELK    -delJ
-delK

A           1.0000
B          -0.1724   1.0000
C          -0.1365  -0.2256   1.0000
-DELJ       0.4113  -0.8060  -0.0483   1.0000
-DELJK     -0.1303  -0.1883  -0.0282  -0.2155   1.0000
-DELK      -0.8011   0.3946   0.0784  -0.4017  -0.3816   1.0000
-delJ       0.4500  -0.8395   0.3270   0.8673  -0.1478  -0.4808   1.0000
-delK      -0.2866  -0.5580   0.3829   0.3736   0.3542   0.0366   0.3228
1.0000

 Mean value of |C.ij|, i.ne.j =   0.3517
 Mean value of  C.ij,  i.ne.j =  -0.0661


 No correlations with absolute value greater than 0.9950


 Worst fitted lines (obs-calc/error):

     6:  -10.1          28:   -9.1          26:    8.0          43:    7.5
    21:    7.2          13:    5.4          18:   -4.9          46:   -4.9
    64:    4.4          73:   -4.1           4:    3.2           1:   -2.7
    32:    2.6          54:    2.4           7:   -2.4          45:    2.4
    62:    2.3           5:    2.3          81:    2.3          38:    2.3
    65:   -2.2          63:   -2.0          59:   -1.9           3:   -1.9
    69:    1.8          24:   -1.8          42:   -1.7          44:   -1.5
    30:   -1.5          14:    1.5          23:    1.5          92:    1.4
    22:   -1.3          17:   -1.3          72:   -1.3          83:    1.3
    80:   -1.2          20:   -1.2          37:   -1.1          61:   -1.1
    49:   -1.0          53:    1.0          33:   -1.0          60:    0.9
    19:   -0.9          88:    0.9          41:   -0.9          78:   -0.8
    85:    0.8          93:   -0.8

     6/  9  0  9    8  0  8                    5160.8301  -0.0101  0.001
    28/ 12  1 11   11  1 10                    7128.0216  -0.0091  0.001
    26/ 11  1 10   10  1  9                    6571.2958   0.0080  0.001
```

```
    43/ 12  2 11   11  2 10                    7128.0216   0.0075  0.001
    21/  9  1  9    8  1  8                    5160.8301   0.0072  0.001
    13/  4  1  3    3  1  2                    2707.4396   0.0054  0.001
    18/  7  1  7    6  1  6                    4046.8510  -0.0049  0.001
    46/ 13  2 11   12  2 10                    7982.7025  -0.0049  0.001
    64/ 13  3 11   12  3 10                    7982.5476   0.0044  0.001
    73/  7  4  3    6  2  4                    6462.5083  -0.0041  0.001
```

____

____/ SPFIT output reformatted with PIFORM

Fit Results for Isomer 2 of (2Z)-2-buten-1-yl-benzene-*d0* complexed with TFIP

```
--------------------------------------------------------------------------------
--------------==========
                                                    obs         o-c     error
blends     Notes

o-c      wt
    / instead of : below denotes (o-c)>3*err
--------------------------------------------------------------------------------
--------------==========
   1:  4  0  4    3  0  3                         2525.6668  -0.0022  0.001
   2:  5  0  5    4  0  4                         3114.8474  -0.0013  0.001
   3/  8  0  8    7  0  7                         4890.5408  -0.0040  0.001
   4:  9  0  9    8  0  8                         5482.7527  -0.0023  0.001
   5: 10  0 10    9  0  9                         6074.9688  -0.0009  0.001
   6: 11  0 11   10  0 10                         6667.1815  -0.0011  0.001
   7: 12  0 12   11  0 11                         7259.3900  -0.0007  0.001
   8: 13  0 13   12  0 12                         7851.5936   0.0010  0.001
   9/  4  1  4    3  1  3                         2518.3373  -0.0042  0.001
  10/  4  1  3    3  1  2                         2865.9183   0.0037  0.001
  11:  5  1  5    4  1  4                         3113.1757   0.0017  0.001
  12:  5  1  4    4  1  3                         3443.2342  -0.0012  0.001
  13:  6  1  6    5  1  5                         3705.9751   0.0019  0.001
  14:  6  1  5    5  1  4                         4020.1233  -0.0007  0.001
  15:  7  1  6    6  1  5                         4606.3701   0.0001  0.001
  16/  8  1  8    7  1  7                         4890.5408   0.0075  0.001
  17:  8  1  7    7  1  6                         5196.7404  -0.0007  0.001
  18:  9  1  9    8  1  8                         5482.7527  -0.0002  0.001
  19:  9  1  8    8  1  7                         5788.3618   0.0001  0.001
  20: 10  1 10    9  1  9                         6074.9688  -0.0006  0.001
  21: 11  1 11   10  1 10                         6667.1815  -0.0010  0.001
  22: 11  1 10   10  1  9                         6972.4152  -0.0018  0.001
  23: 12  1 12   11  1 11                         7259.3900  -0.0007  0.001
  24: 12  1 11   11  1 10                         7564.5399   0.0003  0.001
  25: 13  1 13   12  1 12                         7851.5936   0.0010  0.001
  26:  4  2  3    3  2  2                         2776.3357  -0.0010  0.001
  27:  5  2  4    4  2  3                         3401.7071   0.0018  0.001
  28:  5  2  3    4  2  2                         3768.8523  -0.0019  0.001
  29:  6  2  5    5  2  4                         4006.9556   0.0000  0.001
  30:  6  2  4    5  2  3                         4379.3832   0.0007  0.001
  31:  7  2  6    6  2  5                         4602.9860  -0.0007  0.001
  32:  7  2  5    6  2  4                         4944.5215  -0.0002  0.001
  33:  8  2  7    7  2  6                         5195.9708   0.0004  0.001
  34:  8  2  6    7  2  5                         5515.6099  -0.0006  0.001
  35:  9  2  8    8  2  7                         5788.1998   0.0005  0.001
  36:  9  2  7    8  2  6                         6099.0893  -0.0014  0.001
  37: 10  2  8    9  2  7                         6688.1703  -0.0017  0.001
  38/ 11  2 10   10  2  9                         6972.4152   0.0043  0.001
  39: 11  2  9   10  2  8                         7279.1428  -0.0013  0.001
  40: 12  2 11   11  2 10                         7564.5399   0.0014  0.001
  41:  5  3  3    4  3  2                         3603.8900   0.0027  0.001
  42:  6  3  4    5  3  3                         4264.3371  -0.0006  0.001
  43/  7  3  5    6  3  4                         4891.9720   0.0033  0.001
```

```
  44:  5  3  2    4  1  3                        4929.1378  -0.0005  0.001
  45:  7  3  4    6  3  3                        5301.0118   0.0005  0.001
  46:  8  3  6    7  3  5                        5498.1410  -0.0012  0.001
  47:  8  3  5    7  3  4                        5891.7773  -0.0009  0.001
  48:  9  3  7    8  3  6                        6094.2775   0.0013  0.001
  49:  9  3  6    8  3  5                        6445.8849   0.0019  0.001
  50: 10  3  8    9  3  7                        6686.9872  -0.0016  0.001
  51: 10  3  7    9  3  6                        7012.0298  -0.0018  0.001
  52: 11  3  9   10  3  8                        7278.8767   0.0013  0.001
  53: 11  3  8   10  3  7                        7592.9616  -0.0005  0.001
  54:  6  4  3    5  4  2                        4398.1377   0.0002  0.001
  55:  6  4  2    5  4  1                        4560.0389  -0.0028  0.001
  56:  7  4  4    6  4  3                        5097.6879  -0.0023  0.001
  57:  8  4  5    7  4  4                        5758.7111   0.0016  0.001
  58:  6  4  2    5  2  3                        5832.1591  -0.0003  0.001
  59:  9  4  6    8  4  5                        6385.9503   0.0021  0.001
  60:  9  4  5    8  4  4                        6831.2775   0.0025  0.001
  61:  7  4  3    6  2  4                        6851.9594  -0.0008  0.001
  62: 10  4  7    9  4  6                        6991.5493   0.0016  0.001
  63: 10  4  6    9  4  5                        7402.6533   0.0003  0.001
  64: 11  4  8   10  4  7                        7587.0411  -0.0024  0.001
  65: 11  4  7   10  4  6                        7947.1932  -0.0008  0.001
  66:  7  5  3    6  5  2                        5163.7255   0.0030  0.001
  67:  7  5  2    6  5  1                        5248.7843   0.0016  0.001
  68:  9  5  5    8  5  4                        6597.9014   0.0014  0.001
  69:  7  5  2    6  3  3                        6897.4545   0.0001  0.001
  70/ 10  5  6    9  5  5                        7257.2385  -0.0048  0.001
  71: 11  5  7   10  5  6                        7882.6152   0.0028  0.001
  72:  9  6  4    8  6  3                        6668.8450  -0.0009  0.001
  73:  9  6  3    8  6  2                        6791.0481  -0.0001  0.001
--------------------------------------------------------------------------
------

PARAMETERS IN FIT (values truncated and Nlines statistics):

       10000            A  /MHz          498.86144(15)
1
       20000            B  /MHz          410.759849(68)
2
       30000            C  /MHz          296.129975(31)
3
         200         DELJ  /kHz            0.07873(43)
4
        1100        DELJK  /kHz           -0.0832(25)
5
        2000         DELK  /kHz            0.0753(61)
6
       40100         delJ  /kHz            0.02591(22)
7
       41000         delK  /kHz            0.0289(10)
8

 MICROWAVE AVG =       -0.000006 MHz, IR AVG =        0.00000
 MICROWAVE RMS =        0.002014 MHz, IR RMS =        0.00000
```

```
 END OF ITERATION  2 OLD, NEW RMS ERROR=        2.01364         2.01364

  distinct frequency lines in fit:    73
       distinct parameters of fit:     8

                                   upper state  lower state
overall
      limits of quantum number  1:      4   13       3   12           3
13
      limits of quantum number  2:      0    6       0    6           0
6
      limits of quantum number  3:      2   13       1   12           1
13

                 frequency range:        2518         7947


 PARAMETERS IN FIT WITH STANDARD ERRORS ON THOSE THAT ARE FITTED:
 (values rounded and degrees of freedom, Ndegf=Nlines-Nconst, statistics)

       10000           A  /MHz          498.86144(33)
1
       20000           B  /MHz          410.75985(14)
2
       30000           C  /MHz          296.129976(67)
3
         200        DELJ  /kHz            0.07873(91)
4
        1100       DELJK  /kHz           -0.0832(53)
5
        2000        DELK  /kHz            0.075(13)
6
       40100        delJ  /kHz            0.02592(46)
7
       41000        delK  /kHz            0.0290(22)
8

 CORRELATION COEFFICIENTS, C.ij:

           A        B        C        -DELJ    -DELJK   -DELK    -delJ
-delK

A           1.0000
B          -0.3685   1.0000
C          -0.2356  -0.2143   1.0000
-DELJ       0.5501  -0.7913  -0.0175   1.0000
-DELJK     -0.3029   0.1477   0.0121  -0.6375   1.0000
-DELK      -0.6649   0.4609   0.1233  -0.2442  -0.4106   1.0000
-delJ       0.5490  -0.8254   0.1861   0.9632  -0.5941  -0.2863   1.0000
-delK      -0.2445  -0.5185   0.4715   0.1775   0.3609  -0.1702   0.2036
1.0000

 Mean value of |C.ij|, i.ne.j =   0.3833
 Mean value of  C.ij,  i.ne.j =  -0.0829
```

```
 No correlations with absolute value greater than 0.9950


 Worst fitted lines (obs-calc/error):

   16:    7.5        70:   -4.8        38:    4.3          9:   -4.2
    3:   -4.0        10:    3.7        43:    3.3         66:    3.0
   71:    2.8        55:   -2.8        41:    2.7         60:    2.5
   64:   -2.4        56:   -2.3         4:   -2.3          1:   -2.2
   59:    2.1        28:   -1.9        49:    1.9         13:    1.9
   27:    1.8        22:   -1.8        51:   -1.8         11:    1.7
   37:   -1.7        67:    1.6        57:    1.6         50:   -1.6
   62:    1.6        68:    1.4        36:   -1.4         40:    1.4
   39:   -1.3        52:    1.3        48:    1.3          2:   -1.3
   46:   -1.2        12:   -1.2         6:   -1.1         26:   -1.0
   25:    1.0        21:   -1.0         8:    1.0         47:   -0.9
   72:   -0.9         5:   -0.9        65:   -0.8         61:   -0.8
   17:   -0.7        31:   -0.7

   16/  8  1  8    7  1  7                    4890.5408   0.0075  0.001
   70/ 10  5  6    9  5  5                    7257.2385  -0.0048  0.001
   38/ 11  2 10   10  2  9                    6972.4152   0.0043  0.001
    9/  4  1  4    3  1  3                    2518.3373  -0.0042  0.001
    3/  8  0  8    7  0  7                    4890.5408  -0.0040  0.001
   10/  4  1  3    3  1  2                    2865.9183   0.0037  0.001
   43/  7  3  5    6  3  4                    4891.9720   0.0033  0.001
   66:  7  5  3    6  5  2                    5163.7255   0.0030  0.001
   71: 11  5  7   10  5  6                    7882.6152   0.0028  0.001
   55:  6  4  2    5  4  1                    4560.0389  -0.0028  0.001
```

____
____/ SPFIT output reformatted with PIFORM

Fit Results for the Heterochiral Diastereomer of Isomer 1 for (2Z)-2-buten-1-yl-benzene-*d1* complexed with TFIP

```
-----------------------------------------------------------------------------
---------=========
                                                        obs         o-c     error
blends     Notes

o-c      wt
    / instead of : below denotes (o-c)>3*err
-----------------------------------------------------------------------------
---------=========
   1/  4  0  4    3  0  3                     2368.7598  -0.0129  0.001
   2/  5  0  5    4  0  4                     2916.4357  -0.0126  0.001
   3:  6  0  6    5  0  5                     3467.6546  -0.0013  0.001
   4/  7  0  7    6  0  6                     4020.2189  -0.0064  0.001
   5/  8  0  8    7  0  7                     4573.2041  -0.0090  0.001
   6/  9  0  9    8  0  8                     5126.3045  -0.0140  0.001
   7/ 10  0 10    9  0  9                     5679.4486  -0.0059  0.001
   8: 11  0 11   10  0 10                     6232.5943  -0.0012  0.001
   9: 12  0 12   11  0 11                     6785.7335   0.0001  0.001
  10/ 13  0 13   12  0 12                     7338.8687   0.0044  0.001
  11/ 14  0 14   13  0 13                     7891.9927   0.0064  0.001
  12/  4  1  4    3  1  3                     2353.3338  -0.0046  0.001
  13:  4  1  3    3  1  2                     2689.5884  -0.0021  0.001
  14/  5  1  5    4  1  4                     2911.5350  -0.0060  0.001
  15/  5  1  4    4  1  3                     3247.7036  -0.0063  0.001
  16/  6  1  6    5  1  5                     3466.2900   0.0041  0.001
  17/  6  1  5    5  1  4                     3783.4285  -0.0038  0.001
  18:  7  1  7    6  1  6                     4019.8680  -0.0028  0.001
  19/  7  1  6    6  1  5                     4324.1355   0.0062  0.001
  20:  8  1  8    7  1  7                     4573.1259   0.0000  0.001
  21:  8  1  7    7  1  6                     4871.8903   0.0010  0.001
  22/  9  1  9    8  1  8                     5126.3045   0.0067  0.001
  23:  9  1  8    8  1  7                     5423.0156  -0.0021  0.001
  24: 10  1 10    9  1  9                     5679.4486  -0.0011  0.001
  25/ 10  1  9    9  1  8                     5975.3816  -0.0070  0.001
  26: 11  1 11   10  1 10                     6232.5943  -0.0001  0.001
  27: 11  1 10   10  1  9                     6528.1909   0.0030  0.001
  28: 12  1 12   11  1 11                     6785.7335   0.0004  0.001
  29/ 12  1 11   11  1 10                     7081.1252  -0.0140  0.001
  30/ 13  1 13   12  1 12                     7338.8687   0.0045  0.001
  31: 13  1 12   12  1 11                     7634.1514   0.0016  0.001
  32/ 14  1 14   13  1 13                     7891.9927   0.0064  0.001
  33:  4  2  3    3  2  2                     2578.1640   0.0017  0.001
  34/  4  2  2    3  2  1                     2818.7128  -0.0155  0.001
  35:  5  2  4    4  2  3                     3173.5285  -0.0012  0.001
  36/  5  2  3    4  2  2                     3501.0936  -0.0070  0.001
  37/  6  2  5    5  2  4                     3748.9137  -0.0049  0.001
  38/  6  2  4    5  2  3                     4120.6724  -0.0046  0.001
  39/  7  2  5    6  2  4                     4676.1474  -0.0044  0.001
```

40/	8	2	7	7	2	6	4867.8908	-0.0057	0.001
41:	8	2	6	7	2	5	5203.3623	-0.0020	0.001
42/	9	2	8	8	2	7	5421.8555	-0.0036	0.001
43:	9	2	7	8	2	6	5735.8961	0.0018	0.001
44/	10	2	9	9	2	8	5975.0765	0.0043	0.001
45:	10	2	8	9	2	7	6278.8918	0.0000	0.001
46/	11	2	9	10	2	8	6827.6915	-0.0034	0.001
47/	12	2	11	11	2	10	7081.1252	0.0068	0.001
48:	12	2	10	11	2	9	7378.9220	-0.0006	0.001
49/	13	2	12	12	2	11	7634.1514	0.0068	0.001
50:	13	2	11	12	2	10	7931.0932	-0.0003	0.001
51:	5	3	3	4	3	2	3326.9431	-0.0011	0.001
52:	5	3	2	4	3	1	3489.4472	0.0022	0.001
53:	6	3	4	5	3	3	3959.1087	-0.0018	0.001
54:	7	3	5	6	3	4	4564.7485	-0.0024	0.001
55:	8	3	6	7	3	5	5147.0385	-0.0010	0.001
56/	8	3	5	7	3	4	5555.8378	-0.0043	0.001
57:	9	3	7	8	3	6	5713.3283	-0.0001	0.001
58:	9	3	6	8	3	5	6109.4442	-0.0007	0.001
59:	10	3	8	9	3	7	6271.0743	-0.0024	0.001
60:	10	3	7	9	3	6	6628.3638	0.0021	0.001
61:	11	3	8	10	3	7	7151.8665	0.0009	0.001
62/	12	3	10	11	3	9	7378.1974	0.0031	0.001
63/	12	3	9	11	3	8	7688.9718	0.0038	0.001
64/	13	3	11	12	3	10	7930.8950	0.0063	0.001
65/	11	3	9	10	3	8	6825.2350	0.0052	0.001
66:	6	4	3	5	4	2	4037.6483	-0.0025	0.001
67:	6	4	2	5	4	1	4116.4860	-0.0009	0.001
68:	7	4	4	6	4	3	4703.8535	0.0007	0.001
69/	7	4	3	6	4	2	4888.9803	-0.0044	0.001
70/	8	4	5	7	4	4	5345.9345	0.0039	0.001
71:	8	4	4	7	4	3	5653.1896	0.0011	0.001
72:	9	4	6	8	4	5	5959.7383	-0.0009	0.001
73:	9	4	5	8	4	4	6362.6267	0.0008	0.001
74:	10	4	7	9	4	6	6547.7742	0.0002	0.001
75:	10	4	6	9	4	5	6993.9758	0.0018	0.001
76:	11	4	8	10	4	7	7117.2368	0.0026	0.001
77:	11	4	7	10	4	6	7546.8732	0.0000	0.001
78/	12	4	9	11	4	8	7676.1364	0.0078	0.001
79/	8	5	4	7	5	3	5411.6458	-0.0037	0.001
80:	8	5	3	7	5	2	5502.6109	0.0010	0.001
81/	9	5	5	8	5	4	6086.5441	0.0042	0.001
82:	9	5	4	8	5	3	6290.1320	-0.0022	0.001
83:	10	5	6	9	5	5	6736.8812	0.0009	0.001
84/	10	5	5	9	5	4	7071.4016	0.0042	0.001
85:	11	5	7	10	5	6	7357.7285	-0.0005	0.001
86:	11	5	6	10	5	5	7795.0246	0.0024	0.001
87/	12	5	8	11	5	7	7950.8642	0.0047	0.001
88/	9	6	4	8	6	3	6094.1125	-0.0041	0.001
89/	9	6	3	8	6	2	6128.2819	0.0031	0.001
90/	10	6	5	9	6	4	6790.8079	-0.0048	0.001
91:	10	6	4	9	6	3	6891.2618	-0.0010	0.001
92/	11	6	6	10	6	5	7473.2353	0.0051	0.001
93/	11	6	5	10	6	4	7691.8413	0.0031	0.001

```
---------------------------------------------------------------------------
------

PARAMETERS IN FIT (values truncated and Nlines statistics):

       10000            A  /MHz          494.257459(89)
1
       20000            B  /MHz          376.467026(15)
2
       30000            C  /MHz          276.604288(11)
3
         200         DELJ  /kHz          [ 0.05822]
4
        1100        DELJK  /kHz          [ 0.153]
5
        2000         DELK  /kHz          [-0.137]
6
       40100         delJ  /kHz          [ 0.01136]
7
       41000         delK  /kHz          [ 0.1013]
8

 MICROWAVE AVG =       -0.000733 MHz, IR AVG =        0.00000
 MICROWAVE RMS =        0.004948 MHz, IR RMS =        0.00000
 END OF ITERATION  2 OLD, NEW RMS ERROR=        4.94812         4.94812

  distinct frequency lines in fit:    93
       distinct parameters of fit:     3

                                    upper state  lower state
overall
      limits of quantum number  1:      4   14       3   13            3
14
      limits of quantum number  2:      0    6       0    6            0
6
      limits of quantum number  3:      2   14       1   13            1
14

                  frequency range:        2353        7950


 PARAMETERS IN FIT WITH STANDARD ERRORS ON THOSE THAT ARE FITTED:
 (values rounded and degrees of freedom, Ndegf=Nlines-Nconst, statistics)

       10000            A  /MHz          494.25746(44)
1
       20000            B  /MHz          376.467026(79)
2
       30000            C  /MHz          276.604289(55)
3
         200         DELJ  /kHz          [ 0.05822]
4
        1100        DELJK  /kHz          [ 0.153]
5
```

```
         2000         DELK  /kHz          [-0.137]
6
        40100         delJ  /kHz          [ 0.01136]
7
        41000         delK  /kHz          [ 0.1013]
8

 CORRELATION COEFFICIENTS, C.ij:

           A        B        C

A           1.0000
B           0.4121   1.0000
C          -0.6569  -0.5350   1.0000

 Mean value of |C.ij|, i.ne.j =   0.5347
 Mean value of  C.ij,  i.ne.j =  -0.2599


 No correlations with absolute value greater than 0.9950


 Worst fitted lines (obs-calc/error):

    34:  -15.5          6:  -14.0         29:  -14.0          1:  -12.9
     2:  -12.6          5:   -9.0         78:    7.8         36:   -7.0
    25:   -7.0         47:    6.8         49:    6.8         22:    6.7
     4:   -6.4         11:    6.4         32:    6.4         64:    6.3
    15:   -6.3         19:    6.2         14:   -6.0          7:   -5.9
    40:   -5.7         65:    5.2         92:    5.1         37:   -4.9
    90:   -4.8         87:    4.7         38:   -4.6         12:   -4.6
    30:    4.5         39:   -4.4         10:    4.4         69:   -4.4
    56:   -4.3         44:    4.3         84:    4.2         81:    4.2
    16:    4.1         88:   -4.1         70:    3.9         17:   -3.8
    63:    3.8         79:   -3.7         42:   -3.6         46:   -3.4
    93:    3.1         62:    3.1         89:    3.1         27:    3.0
    18:   -2.8         76:    2.6

    34/  4  2  2    3  2  1                     2818.7128  -0.0155  0.001
     6/  9  0  9    8  0  8                     5126.3045  -0.0140  0.001
    29/ 12  1 11   11  1 10                     7081.1252  -0.0140  0.001
     1/  4  0  4    3  0  3                     2368.7598  -0.0129  0.001
     2/  5  0  5    4  0  4                     2916.4357  -0.0126  0.001
     5/  8  0  8    7  0  7                     4573.2041  -0.0090  0.001
    78/ 12  4  9   11  4  8                     7676.1364   0.0078  0.001
    36/  5  2  3    4  2  2                     3501.0936  -0.0070  0.001
    25/ 10  1  9    9  1  8                     5975.3816  -0.0070  0.001
    47/ 12  2 11   11  2 10                     7081.1252   0.0068  0.001

_____________________________________
__________________________________________/ SPFIT output reformatted with
PIFORM
```

Fit Results for the Homochiral Diastereomer of Isomer 1 for (2Z)-2-buten-1-yl-benzene-*d1* complexed with TFIP

```
-----------------------------------------------------------------------------
-----------=========
                                                   obs          o-c     error
blends     Notes

o-c       wt
    / instead of : below denotes (o-c)>3*err
-----------------------------------------------------------------------------
-----------=========
   1:  5  0  5    4  0  4                          2918.3161  -0.0012  0.001
   2/  6  0  6    5  0  5                          3470.0498  -0.0060  0.001
   3/  7  0  7    6  0  6                          4023.1936  -0.0068  0.001
   4/  9  0  9    8  0  8                          5130.4819  -0.0128  0.001
   5/ 10  0 10    9  0  9                          5684.2342  -0.0037  0.001
   6: 11  0 11   10  0 10                          6237.9867  -0.0004  0.001
   7: 12  0 12   11  0 11                          6791.7344   0.0010  0.001
   8: 13  0 13   12  0 12                          7345.4748   0.0019  0.001
   9/ 14  0 14   13  0 13                          7899.2097   0.0062  0.001
  10/  7  1  7    6  1  6                          4022.8202   0.0039  0.001
  11:  6  1  5    5  1  4                          3783.9097   0.0007  0.001
  12:  5  1  4    4  1  3                          3247.1976   0.0004  0.001
  13/  6  1  6    5  1  5                          3468.5878  -0.0076  0.001
  14/  7  1  6    6  1  5                          4324.9312  -0.0052  0.001
  15:  8  1  7    7  1  6                          4873.0555  -0.0009  0.001
  16/  9  1  9    8  1  8                          5130.4819   0.0104  0.001
  17/  9  1  8    8  1  7                          5424.6641  -0.0081  0.001
  18: 10  1 10    9  1  9                          5684.2342   0.0016  0.001
  19: 10  1  9    9  1  8                          5977.6004  -0.0014  0.001
  20: 11  1 11   10  1 10                          6237.9867   0.0008  0.001
  21/ 12  1 11   11  1 10                          7084.5277  -0.0134  0.001
  22: 13  1 13   12  1 12                          7345.4748   0.0020  0.001
  23: 13  1 12   12  1 11                          7638.1579   0.0020  0.001
  24/ 14  1 14   13  1 13                          7899.2097   0.0062  0.001
  25:  5  2  4    4  2  3                          3171.6599  -0.0016  0.001
  26:  6  2  5    5  2  4                          3748.0297  -0.0009  0.001
  27:  6  2  4    5  2  3                          4116.1217  -0.0028  0.001
  28:  7  2  5    6  2  4                          4674.6970  -0.0027  0.001
  29:  8  2  6    7  2  5                          5203.2643  -0.0021  0.001
  30:  9  2  8    8  2  7                          5423.4030   0.0020  0.001
  31/  9  2  7    8  2  6                          5735.8961  -0.0082  0.001
  32/ 10  2  9    9  2  8                          5977.2369  -0.0118  0.001
  33/ 10  2  8    9  2  7                          6278.9875  -0.0033  0.001
  34/ 11  2  9   10  2  8                          6828.1188  -0.0045  0.001
  35/ 12  2 11   11  2 10                          7084.5277   0.0106  0.001
  36: 12  2 10   11  2  9                          7379.8348   0.0004  0.001
  37/ 13  2 12   12  2 11                          7638.1579   0.0080  0.001
  38:  5  3  3    4  3  2                          3321.3655   0.0012  0.001
  39:  5  3  2    4  3  1                          3478.4658  -0.0008  0.001
  40:  6  3  4    5  3  3                          3953.9264   0.0002  0.001
  41/  6  3  3    5  3  2                          4223.2308  -0.0042  0.001
```

```
  42:  7  3  5    6  3  4                         4560.6250  -0.0019  0.001
  43:  8  3  6    7  3  5                         5144.2425  -0.0001  0.001
  44:  8  3  5    7  3  4                         5549.1468  -0.0029  0.001
  45/  9  3  6    8  3  5                         6107.1655  -0.0071  0.001
  46: 10  3  8    9  3  7                         6270.4653   0.0000  0.001
  47: 10  3  7    9  3  6                         6628.0010   0.0012  0.001
  48/ 11  3  8   10  3  7                         7151.3775  -0.0066  0.001
  49: 12  3 10   11  3  9                         7379.0116   0.0000  0.001
  50/ 12  3  9   11  3  8                         7688.2091   0.0034  0.001
  51/ 13  3 11   12  3 10                         7932.3306   0.0067  0.001
  52: 11  4  8   10  4  7                         7113.9752   0.0014  0.001
  53/ 11  4  7   10  4  6                         7543.8345   0.0040  0.001
  54/ 12  4  9   11  4  8                         7674.0577   0.0065  0.001
  55: 10  4  6    9  4  5                         6984.9438   0.0014  0.001
  56:  9  4  5    8  4  4                         6348.1544  -0.0007  0.001
  57/  7  4  3    6  2  4                         6412.5281   0.0040  0.001
  58: 10  4  7    9  4  6                         6542.9610   0.0005  0.001
  59:  9  4  6    8  4  5                         5953.1896   0.0016  0.001
  60:  8  4  5    7  4  4                         5337.9151   0.0012  0.001
  61/  8  4  4    7  4  3                         5636.0377  -0.0059  0.001
  62/  7  4  4    6  4  3                         4695.1357   0.0037  0.001
  63/  7  4  3    6  4  2                         4872.5558  -0.0045  0.001
  64/  6  4  3    5  4  2                         4029.2258  -0.0051  0.001
  65/  6  4  2    5  4  1                         4103.8778  -0.0064  0.001
  66/  8  5  4    7  5  3                         5399.6946   0.0042  0.001
  67/  8  5  3    7  5  2                         5484.8218  -0.0051  0.001
  68:  9  5  5    8  5  4                         6074.4441  -0.0011  0.001
  69:  9  5  4    8  5  3                         6267.8275   0.0016  0.001
  70/ 10  5  6    9  5  5                         6725.8322   0.0044  0.001
  71: 10  5  5    9  5  4                         7048.5946   0.0009  0.001
  72: 11  5  7   10  5  6                         7348.5916   0.0025  0.001
  73: 11  5  6   10  5  5                         7776.1748   0.0028  0.001
  74/ 12  5  8   11  5  7                         7943.9270   0.0048  0.001
  75/ 10  6  5    9  6  4                         6775.0925  -0.0078  0.001
  76/ 10  6  4    9  6  3                         6868.0397   0.0078  0.001
  77/ 11  6  6   10  6  5                         7457.5621   0.0051  0.001
  78: 11  6  5   10  6  4                         7663.2764   0.0017  0.001
  79:  9  6  4    8  6  3                         6079.6044   0.0019  0.001
  80/  9  6  3    8  6  2                         6110.8548  -0.0036  0.001
--------------------------------------------------------------------------
------

PARAMETERS IN FIT (values truncated and Nlines statistics):

       10000            A   /MHz          494.019876(75)
1
       20000            B   /MHz          375.252631(14)
2
       30000            C   /MHz          276.908719(10)
3
         200         DELJ   /kHz          [ 0.05822]
4
        1100        DELJK   /kHz          [ 0.153]
5
```

```
         2000          DELK  /kHz          [-0.137]
6
        40100          delJ  /kHz          [ 0.01136]
7
        41000          delK  /kHz          [ 0.1013]
8

 MICROWAVE AVG =       -0.000454 MHz, IR AVG =       0.00000
 MICROWAVE RMS =        0.004938 MHz, IR RMS =       0.00000
 END OF ITERATION  2 OLD, NEW RMS ERROR=       4.93826         4.93826

  distinct frequency lines in fit:    80
       distinct parameters of fit:     3

                                    upper state  lower state
overall
      limits of quantum number  1:      5   14       4   13           4
14
      limits of quantum number  2:      0    6       0    6           0
6
      limits of quantum number  3:      2   14       1   13           1
14

                  frequency range:        2918         7943


 PARAMETERS IN FIT WITH STANDARD ERRORS ON THOSE THAT ARE FITTED:
 (values rounded and degrees of freedom, Ndegf=Nlines-Nconst, statistics)

        10000             A  /MHz          494.01988(37)
1
        20000             B  /MHz          375.252631(74)
2
        30000             C  /MHz          276.908719(53)
3
          200          DELJ  /kHz          [ 0.05822]
4
         1100         DELJK  /kHz          [ 0.153]
5
         2000          DELK  /kHz          [-0.137]
6
        40100          delJ  /kHz          [ 0.01136]
7
        41000          delK  /kHz          [ 0.1013]
8

 CORRELATION COEFFICIENTS, C.ij:

           A        B        C

A           1.0000
B           0.2936   1.0000
C          -0.5443  -0.4590   1.0000
```

```
Mean value of |C.ij|, i.ne.j =   0.4323
Mean value of  C.ij,  i.ne.j =  -0.2366


No correlations with absolute value greater than 0.9950


Worst fitted lines (obs-calc/error):

   21:  -13.4          4:  -12.8         32:  -11.8         35:   10.6
   16:   10.4         31:   -8.2         17:   -8.1         37:    8.0
   76:    7.8         75:   -7.8         13:   -7.6         45:   -7.1
    3:   -6.8         51:    6.7         48:   -6.6         54:    6.5
   65:   -6.4          9:    6.2         24:    6.2          2:   -6.0
   61:   -5.9         14:   -5.2         77:    5.1         64:   -5.1
   67:   -5.1         74:    4.8         63:   -4.5         34:   -4.5
   70:    4.4         66:    4.2         41:   -4.2         57:    4.0
   53:    4.0         10:    3.9         62:    3.7          5:   -3.7
   80:   -3.6         50:    3.4         33:   -3.3         44:   -2.9
   73:    2.8         27:   -2.8         28:   -2.7         72:    2.5
   29:   -2.1         23:    2.0         22:    2.0         30:    2.0
   79:    1.9         42:   -1.9

   21/ 12  1 11   11  1 10                     7084.5277  -0.0134  0.001
    4/  9  0  9    8  0  8                     5130.4819  -0.0128  0.001
   32/ 10  2  9    9  2  8                     5977.2369  -0.0118  0.001
   35/ 12  2 11   11  2 10                     7084.5277   0.0106  0.001
   16/  9  1  9    8  1  8                     5130.4819   0.0104  0.001
   31/  9  2  7    8  2  6                     5735.8961  -0.0082  0.001
   17/  9  1  8    8  1  7                     5424.6641  -0.0081  0.001
   37/ 13  2 12   12  2 11                     7638.1579   0.0080  0.001
   76/ 10  6  4    9  6  3                     6868.0397   0.0078  0.001
   75/ 10  6  5    9  6  4                     6775.0925  -0.0078  0.001
```

____
____/ SPFIT output reformatted with PIFORM

Fit Results for the Heterochiral Diastereomer of Isomer 2 for (2Z)-2-buten-1-yl-benzene-*d1* complexed with TFIP

```
-----------------------------------------------------------------------------
----------=========
                                                obs         o-c     error
blends     Notes

o-c       wt
    / instead of : below denotes (o-c)>3*err
-----------------------------------------------------------------------------
----------=========
   1/  6  0  6    5  0  5                      3673.0459   0.0040  0.001
   2/  8  0  8    7  0  7                      4846.3603  -0.0110  0.001
   3:  9  0  9    8  0  8                      5433.1641  -0.0017  0.001
   4: 10  0 10    9  0  9                      6019.9681  -0.0005  0.001
   5: 11  0 11   10  0 10                      6606.7701  -0.0004  0.001
   6: 12  0 12   11  0 11                      7193.5679  -0.0004  0.001
   7/ 13  0 13   12  0 12                      7780.3663   0.0061  0.001
   8/  5  1  4    4  1  3                      3417.1534  -0.0047  0.001
   9/  5  1  5    4  1  4                      3084.9927  -0.0049  0.001
  10/  4  1  3    3  1  2                      2841.9081  -0.0089  0.001
  11:  6  1  5    5  1  4                      3987.2549  -0.0029  0.001
  12/  7  1  6    6  1  5                      4566.7154  -0.0058  0.001
  13/  8  1  7    7  1  6                      5151.0912  -0.0072  0.001
  14:  9  1  8    8  1  7                      5737.1074   0.0027  0.001
  15: 11  1 10   10  1  9                      6910.2343  -0.0020  0.001
  16/ 12  1 11   11  1 10                      7496.9268  -0.0054  0.001
  17:  6  2  5    5  2  4                      3970.3084  -0.0009  0.001
  18:  6  2  4    5  2  3                      4345.9911  -0.0003  0.001
  19:  7  2  5    6  2  4                      4909.3636  -0.0001  0.001
  20:  8  2  7    7  2  6                      5149.9265  -0.0027  0.001
  21:  8  2  6    7  2  5                      5472.1700  -0.0030  0.001
  22/  9  2  8    8  2  7                      5736.8455   0.0073  0.001
  23:  9  2  7    8  2  6                      6047.8094  -0.0018  0.001
  24/ 10  2  8    9  2  7                      6630.4040   0.0053  0.001
  25/ 11  2 10   10  2  9                      6910.2343   0.0098  0.001
  26: 12  2 11   11  2 10                      7496.9268  -0.0030  0.001
  27/  6  3  4    5  3  3                      4217.8467   0.0036  0.001
  28/  6  3  3    5  3  2                      4556.0161  -0.0059  0.001
  29:  7  3  5    6  3  4                      4844.3758   0.0008  0.001
  30/  7  3  4    6  3  3                      5249.0690  -0.0031  0.001
  31:  8  3  6    7  3  5                      5448.3381   0.0028  0.001
  32/  8  3  5    7  3  4                      5850.8681  -0.0089  0.001
  33/  9  3  7    8  3  6                      6040.6443   0.0053  0.001
  34/  9  3  6    8  3  5                      6402.5414  -0.0033  0.001
  35: 10  3  8    9  3  7                      6628.4868   0.0018  0.001
  36: 10  3  7    9  3  6                      6958.5176  -0.0022  0.001
  37: 11  3  9   10  3  8                      7215.0736   0.0021  0.001
  38:  9  4  6    8  4  5                      6324.4548   0.0009  0.001
  39:  9  4  5    8  4  4                      6769.6557   0.0030  0.001
  40: 10  4  7    9  4  6                      6928.9045   0.0027  0.001
  41: 10  4  6    9  4  5                      7355.8791   0.0025  0.001
```

```
  42/ 11  4  7   10  4  6                    7896.3764   0.0068  0.001
  43/  8  4  5    7  4  4                    5696.4478   0.0041  0.001
  44:  8  4  4    7  4  3                    6086.9892   0.0002  0.001
---------------------------------------------------------------------------
------

PARAMETERS IN FIT (values truncated and Nlines statistics):

        10000            A  /MHz          499.58485(11)
1
        20000            B  /MHz          404.796598(51)
2
        30000            C  /MHz          293.425435(16)
3
          200         DELJ  /kHz          [ 0.07873]
4
         1100        DELJK  /kHz          [-0.0832]
5
         2000         DELK  /kHz          [ 0.075]
6
        40100         delJ  /kHz          [ 0.02592]
7
        41000         delK  /kHz          [ 0.029]
8

 MICROWAVE AVG =       -0.000437 MHz, IR AVG =        0.00000
 MICROWAVE RMS =        0.004630 MHz, IR RMS =        0.00000
 END OF ITERATION  2 OLD, NEW RMS ERROR=        4.62987         4.62987

  distinct frequency lines in fit:    44
       distinct parameters of fit:     3

                                    upper state  lower state
overall
      limits of quantum number  1:      4   13       3   12           3
13
      limits of quantum number  2:      0    4       0    4           0
4
      limits of quantum number  3:      3   13       2   12           2
13

                  frequency range:        2841         7896


 PARAMETERS IN FIT WITH STANDARD ERRORS ON THOSE THAT ARE FITTED:
 (values rounded and degrees of freedom, Ndegf=Nlines-Nconst, statistics)

        10000            A  /MHz          499.58485(55)
1
        20000            B  /MHz          404.79660(24)
2
        30000            C  /MHz          293.425435(76)
3
```

```
          200         DELJ  /kHz          [ 0.07873]
4
         1100        DELJK  /kHz          [-0.0832]
5
         2000         DELK  /kHz          [ 0.075]
6
        40100         delJ  /kHz          [ 0.02592]
7
        41000         delK  /kHz          [ 0.029]
8

 CORRELATION COEFFICIENTS, C.ij:

           A        B        C

A           1.0000
B          -0.4300   1.0000
C          -0.5038  -0.2384   1.0000

 Mean value of |C.ij|, i.ne.j =   0.3907
 Mean value of  C.ij,  i.ne.j =  -0.3907


 No correlations with absolute value greater than 0.9950


 Worst fitted lines (obs-calc/error):

     2:  -11.0         25:    9.8         32:   -8.9         10:   -8.9
    22:    7.3         13:   -7.2         42:    6.8          7:    6.1
    28:   -5.9         12:   -5.8         16:   -5.4         33:    5.3
    24:    5.3          9:   -4.9          8:   -4.7         43:    4.1
     1:    4.0         27:    3.6         34:   -3.3         30:   -3.1
    39:    3.0         26:   -3.0         21:   -3.0         11:   -2.9
    31:    2.8         20:   -2.7         14:    2.7         40:    2.7
    41:    2.5         36:   -2.2         37:    2.1         15:   -2.0
    35:    1.8         23:   -1.8          3:   -1.7         17:   -0.9
    38:    0.9         29:    0.8          4:   -0.5          5:   -0.4
     6:   -0.4         18:   -0.3         44:    0.2         19:   -0.1

     2/  8  0  8    7  0  7                    4846.3603  -0.0110  0.001
    25/ 11  2 10   10  2  9                    6910.2343   0.0098  0.001
    32/  8  3  5    7  3  4                    5850.8681  -0.0089  0.001
    10/  4  1  3    3  1  2                    2841.9081  -0.0089  0.001
    22/  9  2  8    8  2  7                    5736.8455   0.0073  0.001
    13/  8  1  7    7  1  6                    5151.0912  -0.0072  0.001
    42/ 11  4  7   10  4  6                    7896.3764   0.0068  0.001
     7/ 13  0 13   12  0 12                    7780.3663   0.0061  0.001
    28/  6  3  3    5  3  2                    4556.0161  -0.0059  0.001
    12/  7  1  6    6  1  5                    4566.7154  -0.0058  0.001

_____________________________________
__________________________________________/ SPFIT output reformatted with
PIFORM
```

Fit Results for the Homochiral Diastereomer of Isomer 2 for (2Z)-2-buten-1-yl-benzene-*d1* complexed with TFIP

```
--------------------------------------------------------------------------------------
----------=========
                                                  obs          o-c      error
blends     Notes

o-c       wt
    / instead of : below denotes (o-c)>3*err
--------------------------------------------------------------------------------------
----------=========
   1/  5  0  5    4  0  4                         3097.0042  -0.0054  0.001
   2/  6  0  6    5  0  5                         3684.9945  -0.0122  0.001
   3/  8  0  8    7  0  7                         4862.3755  -0.0074  0.001
   4/  9  0  9    8  0  8                         5451.1685  -0.0051  0.001
   5: 10  0 10    9  0  9                         6039.9675  -0.0022  0.001
   6: 11  0 11   10  0 10                         6628.7628  -0.0012  0.001
   7/ 12  0 12   11  0 11                         7217.5592   0.0054  0.001
   8: 13  0 13   12  0 12                         7806.3399   0.0026  0.001
   9:  5  1  4    4  1  3                         3424.5663   0.0010  0.001
  10:  6  1  6    5  1  5                         3684.6363   0.0020  0.001
  11/  6  1  5    5  1  4                         3997.6946  -0.0042  0.001
  12:  7  1  6    6  1  5                         4580.2023   0.0003  0.001
  13/  8  1  7    7  1  6                         5167.0147  -0.0056  0.001
  14/ 10  1  9    9  1  8                         6343.7098  -0.0054  0.001
  15: 11  1 10   10  1  9                         6932.3702  -0.0019  0.001
  16/ 12  1 11   11  1 10                         7521.0821   0.0094  0.001
  17/  5  2  4    4  2  3                         3381.3286  -0.0039  0.001
  18/  5  2  3    4  2  2                         3745.5399   0.0050  0.001
  19:  6  2  5    5  2  4                         3983.6478  -0.0020  0.001
  20/  6  2  4    5  2  3                         4355.6436  -0.0072  0.001
  21/  7  2  5    6  2  4                         4918.2398   0.0032  0.001
  22:  8  2  7    7  2  6                         5166.1638   0.0026  0.001
  23:  8  2  6    7  2  5                         5485.1874   0.0022  0.001
  24/  9  2  8    8  2  7                         5754.9960   0.0043  0.001
  25:  9  2  7    8  2  6                         6064.6757   0.0011  0.001
  26/ 11  2  9   10  2  8                         7237.5596   0.0086  0.001
  27/  5  3  2    4  3  1                         3814.9645  -0.0039  0.001
  28/  6  3  4    5  3  3                         4237.5130   0.0040  0.001
  29:  7  3  5    6  3  4                         4862.7294  -0.0006  0.001
  30:  7  3  4    6  3  3                         5269.3876  -0.0014  0.001
  31:  8  3  6    7  3  5                         5466.2487   0.0024  0.001
  32:  9  3  7    8  3  6                         6059.3285  -0.0026  0.001
  33:  9  3  6    8  3  5                         6412.1847   0.0006  0.001
  34: 10  3  8    9  3  7                         6648.7468  -0.0015  0.001
  35: 10  3  7    9  3  6                         6973.7065  -0.0028  0.001
  36: 11  3  9   10  3  8                         7237.2425   0.0030  0.001
  37: 11  3  8   10  3  7                         7550.3742   0.0028  0.001
  38:  9  4  5    8  4  4                         6792.0977  -0.0005  0.001
  39: 10  4  7    9  4  6                         6951.1429  -0.0010  0.001
  40: 10  4  6    9  4  5                         7365.0671   0.0002  0.001
  41/ 11  4  8   10  4  7                         7543.6960   0.0037  0.001
```

```
  42/ 11  4  7   10  4  6                    7906.2177  -0.0037  0.001
  43:  9  4  6    8  4  5                    6347.9471  -0.0015  0.001
  44:  8  4  5    7  4  4                    5722.6457   0.0015  0.001
---------------------------------------------------------------------------
------

PARAMETERS IN FIT (values truncated and Nlines statistics):

       10000           A  /MHz          497.26645(11)
1
       20000           B  /MHz          407.756118(63)
2
       30000           C  /MHz          294.420924(16)
3
         200        DELJ  /kHz          [ 0.07873]
4
        1100       DELJK  /kHz          [-0.0832]
5
        2000        DELK  /kHz          [ 0.075]
6
       40100        delJ  /kHz          [ 0.02592]
7
       41000        delK  /kHz          [ 0.029]
8

 MICROWAVE AVG =       -0.000391 MHz, IR AVG =        0.00000
 MICROWAVE RMS =        0.004288 MHz, IR RMS =        0.00000
 END OF ITERATION  2 OLD, NEW RMS ERROR=        4.28826         4.28826

  distinct frequency lines in fit:    44
       distinct parameters of fit:     3

                                    upper state  lower state
overall
      limits of quantum number  1:      5   13       4   12           4
13
      limits of quantum number  2:      0    4       0    4           0
4
      limits of quantum number  3:      2   13       1   12           1
13

                  frequency range:        3097         7906


 PARAMETERS IN FIT WITH STANDARD ERRORS ON THOSE THAT ARE FITTED:
 (values rounded and degrees of freedom, Ndegf=Nlines-Nconst, statistics)

       10000           A  /MHz          497.26646(51)
1
       20000           B  /MHz          407.75612(27)
2
       30000           C  /MHz          294.420925(74)
3
```

```
          200         DELJ  /kHz          [ 0.07873]
4
         1100        DELJK  /kHz          [-0.0832]
5
         2000         DELK  /kHz          [ 0.075]
6
        40100         delJ  /kHz          [ 0.02592]
7
        41000         delK  /kHz          [ 0.029]
8

 CORRELATION COEFFICIENTS, C.ij:

          A        B        C

A          1.0000
B         -0.4805   1.0000
C         -0.4569  -0.2880   1.0000

 Mean value of |C.ij|, i.ne.j =   0.4085
 Mean value of  C.ij,  i.ne.j =  -0.4085


 No correlations with absolute value greater than 0.9950


 Worst fitted lines (obs-calc/error):

    2:  -12.2         16:    9.4         26:    8.6          3:   -7.4
   20:   -7.2         13:   -5.6         14:   -5.4          1:   -5.4
    7:    5.4          4:   -5.1         18:    5.0         24:    4.3
   11:   -4.2         28:    4.0         27:   -3.9         17:   -3.9
   42:   -3.7         41:    3.7         21:    3.2         36:    3.0
   35:   -2.8         37:    2.8         22:    2.6         32:   -2.6
    8:    2.6         31:    2.4         23:    2.2          5:   -2.2
   19:   -2.0         10:    2.0         15:   -1.9         44:    1.5
   43:   -1.5         34:   -1.5         30:   -1.4          6:   -1.2
   25:    1.1          9:    1.0         39:   -1.0         33:    0.6
   29:   -0.6         38:   -0.5         12:    0.3         40:    0.2

    2/  6  0  6    5  0  5                      3684.9945  -0.0122  0.001
   16/ 12  1 11   11  1 10                      7521.0821   0.0094  0.001
   26/ 11  2  9   10  2  8                      7237.5596   0.0086  0.001
    3/  8  0  8    7  0  7                      4862.3755  -0.0074  0.001
   20/  6  2  4    5  2  3                      4355.6436  -0.0072  0.001
   13/  8  1  7    7  1  6                      5167.0147  -0.0056  0.001
   14/ 10  1  9    9  1  8                      6343.7098  -0.0054  0.001
    1/  5  0  5    4  0  4                      3097.0042  -0.0054  0.001
    7/ 12  0 12   11  0 11                      7217.5592   0.0054  0.001
    4/  9  0  9    8  0  8                      5451.1685  -0.0051  0.001
```

____
____/ SPFIT output reformatted with
PIFORM

References:

New reference list

[1] Lloyd-Jones, G. C.; Harvey, J. N.; Lennox, J.; Mills, B. M. [$(RCN)_2PdCl_2$]-Catalyzed *E/Z* Isomerization of Alkenes: A Non-Hydride Binuclear Addition–Elimination Pathway. *Angew. Chem. Int. Ed.* **2011**, *50*, 9602–9606.

[2] Abramovitch, A.; Marek, I. Zinc Homologation–Elimination Reaction of α-Sulfinyl Carbanions as a New Route to Olefins. *Eur. J Org. Chem.* **2008**, *2008* (29), 4924–4931.

[3] Xu, W.; Tang, L.; Ge, C.; Chen, J.; Zhou, L. Synthesis of Tetrahydroisoindolinones via a Metal-Free Dehydrogenative Diels-Alder Reaction *Adv. Synth. Catal.* **2019**, *361*, 2268–2273.

[4] Peña-López, M.; Ayán-Varela, M.; Sarandeses, Luis A.; Pérez Sestelo, J. Palladium-Catalyzed Cross-Coupling Reactions of Organogold(I) Reagents with Organic Electrophiles *Chem. Eur. J.* **2010**, *16*, 9905–9909.

[5] Cheung, K. P. S.; Fang, J.; Mukherjee, K.; Mihranyan, A.; Gevorgyan, V. Asymmetric Intermolecular Allylic C–H Amination of Alkenes with Aliphatic Amines. *Science* **2022**, *378*, 1207–1213.

[6] Yu, X.; Zhao, H.; Li, P.; Koh, M. J. Iron-Catalyzed Tunable and Site-Selective Olefin Transposition. *J. Am. Chem. Soc.* **2020**, *142*, 18223–18230.

[7] Hayashi, S.; Hirano, K.; Yorimitsu, H.; Oshima, K. Palladium-Catalyzed Stereo- and Regiospecific Allylation of Aryl Halides with Homoallyl Alcohols via Retro-Allylation: Selective Generation and Use of σ-Allylpalladium. *J. Am. Chem. Soc.* **2006**, *128*, 2210–2211.

[8] Suga, T.; Takahashi, Y.; Ukaji, Y. One-Shot Radical Cross Coupling between Benzyl Alcohols and Alkenyl Halides Using Ni/Ti/Mn System. *Adv. Synth. Catal.* **2020**, *362*, 5622–5626.

[9] Mondal, S.; Luca Deiana; Córdova, A.; Wu, H.; Jan-E Bäckvall. Heterogeneous Copper-Catalyzed Grignard Reactions with Allylic Substrates. *Chem. Commun.* **2025**, *61*, 2802

[10] Kawamura, S.; Agata, R.; Nakamura, M. Regio- and Stereoselective Multisubstituted Olefin Synthesis via Hydro/Carboalumination of Alkynes and Subsequent Iron-Catalysed Cross-Coupling Reaction with Alkyl Halides. *Org. Chem. Front.* **2015**, *2*, 1053–1058.

[11] Degl'Innocenti, A.; Mordini, A.; Pagliai, L.; Ricci, A., Allylsilanes by the Regio- and Stereocontrolled Substitution of Metalated Homoallylsilanes. Synlett **1991**, *1991*,155-156

[12] Abramovitch, A.; Marek, I. Zinc Homologation–Elimination Reaction of α-Sulfinyl Carbanions as a New Route to Olefins. *Eur. J Org. Chem.* **2008**, *2008* (29), 4924–4931.

[13] Meng, Q.; Schirmer, T. E.; Katou, K.; König, B. Controllable Isomerization of Alkenes by Dual Visible-Light-Cobalt Catalysis. *Angew. Chem. Int. Ed.* **2019**, *58*, 5723–5728

[14] Bao, H.; Zhou, B.; Luo, S.-P.; Xu, Z.; Jin, H.; Liu, Y. P/N Heteroleptic Cu(I)-Photosensitizer-Catalyzed Deoxygenative Radical Alkylation of Aromatic Alkynes with Alkyl Aldehydes Using Dipropylamine as a Traceless Linker Agent. *ACS Catal.* **2020**, *10*, 7563–7572

[15] Li, Y.; Ye, Z.; Lin, Y.; Liu, Y.; Zhang, Y.; Gong, L. Organophotocatalytic Selective Deuterodehalogenation of Aryl or Alkyl Chlorides. *Nat. Comm.* **2021**, *12* (1).

[16] Tang, L.; Zang, Y.; Guo, W.; Han, Z.; Huang, H.; Sun, J. Reductive Opening of Oxetanes Catalyzed by Frustrated Lewis Pairs: Unexpected Aryl Migration via Neighboring Group Participation. *Org. Lett.* **2022**, *24*, 3259–3264.

[17] Xu, X.; Wang, M.; Peng, L.; Guo, C. Nickel-Catalyzed Asymmetric Propargylation for the Synthesis of Axially Chiral 1,3-Disubstituted Allenes. *J. Am. Chem. Soc.* **2022**, *144*, 21022−21029.

[18] Diagne, A. B.; Li, S.; Perkowski, G. A.; Mrksich, M.; Thomson, R. J. SAMDI Mass Spectrometry-Enabled High-Throughput Optimization of a Traceless Petasis Reaction. *ACS Comb. Sci.* **2015**, *17*, 658−662

[19] Kuang, J.; Tang, X.; Ma, S. Zinc Diiodide-Promoted Synthesis of Trisubstituted Allenes from Propargylic Amines. *Org. Chem. Front.,* **2015**, *2*, 470–475.

[20] Bolte, B.; Yann Odabachian; Fabien Gagosz. Gold(I)-Catalyzed Rearrangement of Propargyl Benzyl Ethers: A Practical Method for the Generation and in Situ Transformation of Substituted Allenes. *J. Am. Chem. Soc.* **2010**, *132*, 7294–7296.

[21] Yoshida, M.; Okada, T.; Shishido, K. Enantiospecific Synthesis of 1,3-Disubstituted Allenes by Palladium-Catalyzed Coupling of Propargylic Compounds with Arylboronic Acids. *Tetrahedron* **2007**, *63*, 6996–7002;

[22] Khrakovsky, D. A.; Tao, C.; Johnson, M. W.; Thornbury, R. T.; Shevick, S. L.; F. Dean Toste. Enantioselective, Stereodivergent Hydroazidation and Hydroamination of Allenes Catalyzed by Acyclic Diaminocarbene (ADC) Gold(I) Complexes. *Angew. Chem. Int. Ed.* **2016**, *55*, 6079–6083.

[23] Long, J.; Gao, J.; Fang, X. Nickel-Catalyzed Asymmetric Hydrocyanation of Allenes. *Org. Lett.* **2020**, *22*, 376–380.

[24] Lustosa, D. M.; Clemens, S.; Rudolph, M.; Hashmi, S. K. Gold-Catalyzed One-Pot Synthesis of 1,3-Disubstituted Allenes from Benzaldehydes and Terminal Alkynes. *Adv. Synth. Catal.* **2019**, *361*, 5050–5056.

[25] Satoh, T.; Sakamoto, T.; Watanabe, M.; Takano, K. Reaction of Magnesium Alkylidene Carbenoids with Lithium a-Sulfonyl Carbanions: A Novel Synthesis of Tri- and Tetra-substituted Allenes from 1-Chlorovinyl p-Tolyl Sulfoxides and Sulfones *Chem. Pharm. Bull.* **2023**, *51,* 966–970.

[26] Yuan, Y.; Zhang, X.; Qian, H.; Ma, S. Catalytic Enantioselective Allene–Anhydride Approach to β,γ-Unsaturated Enones Bearing an α-All-Carbon-Quarternary Center. *Chem. Sci.* **2020**, *11*, 9115–9121.

[27] Kessler, S. N.; Jan-E. Bäckvall. Iron-Catalyzed Cross-Coupling of Propargyl Carboxylates and Grignard Reagents: Synthesis of Substituted Allenes. *Angew. Chem. Int. Ed.* **2016**, *55*, 3734–3738.

[28] Dong, W.; Zhao, Z.; Gu, C.-Z.; Liu, J.-G.; Yang, S.; Fang, X. Copper-Catalyzed Umpolung Reactivity of Propargylic Carbonates in the Presence of Diboronates: One Stone Four Birds. *J. Am. Chem. Soc.* **2023**, *145*, 27539–27554.

[29] Movassaghi, M.; Ahmad, O. K. N-Isopropylidene-N'-2-Nitrobenzenesulfonyl Hydrazine, a Reagent for Reduction of Alcohols via the Corresponding Monoalkyl Diazenes. *J. Org. Chem.* **2007**, *72*, 1838–1841.

[30] Köpfer, A.; Breit, B. Rhodium-Catalyzed Hydroformylation of 1,1-Disubstituted Allenes Employing the Self-Assembling 6-DPPon System. *Angew. Chem. Int. Ed.* **2015**, *54*, 6913–6917.

[31] Lee, D.; Ryu, T.; Park, Y.-C.; Phil Ho Lee. Unmasked Acyl Anion Equivalent from Acid Chloride with Indium: Reversed-Polarity Synthesis of Unsymmetric Aryl Aryl and Alkenyl Aryl Ketone through Palladium-Catalyzed Cross-Coupling Reaction. *Org. Lett.* **2014**, *16*, 1144–1147.

[32] Reynolds, J. F.; Zhang, X.; Chen, W.; Shimada, S. Dinuclear Palladacyclic Complexes Derived from C−N Cleavage of an Imidazolium Salt: Synthesis, Structural Characterization, and Their Uses for C−C Coupling. *Organometallics* **2008**, *27*, 4166–4172.

[33] Niu, G.; Huang, P.; Chuang, G. J. Triphenylphosphine/Triethylamine-Mediated Decarboxylation of α-Oxocarboxylic Acids and Application in a One-Pot Synthesis of Deuterated Aldehydes. *Asian J. Org. Chem.* **2015**, *5*, 57–61.

[34] Liu, C.-F.; Wang, Z.-C.; Luo, X.; Lu, J.; Min, H.; Shi, S.-L.; Koh, M. J. Synthesis of Tri- and Tetrasubstituted Stereocentres by Nickel-Catalysed Enantioselective Olefin Cross-Couplings. *Nat. Catal.* **2022**, *5*, 934–942.

[35] Pracht, P.; Bohle, F.; Grimme, S. Automated exploration of the low-energy chemical space with fast quantum chemical methods. *Phys. Chem. Chem. Phys.* **2020**, *22*, 7169-7192.

[36] Gaussian 16, Revision C.01, M. J. Frisch, G. W. Trucks, H. B. Schlegel, G. E. Scuseria, M. A. Robb, J. R. Cheeseman, G. Scalmani, V. Barone, G. A. Petersson, H. Nakatsuji, X.

Li, M. Caricato, A. V. Marenich, J. Bloino, B. G. Janesko, R. Gomperts, B. Mennucci, H. P. Hratchian, J. V. Ortiz, A. F. Izmaylov, J. L. Sonnenberg, D. Williams-Young, F. Ding, F. Lipparini, F. Egidi, J. Goings, B. Peng, A. Petrone, T. Henderson, D. Ranasinghe, V. G. Zakrzewski, J. Gao, N. Rega, G. Zheng, W. Liang, M. Hada, M. Ehara, K. Toyota, R. Fukuda, J. Hasegawa, M. Ishida, T. Nakajima, Y. Honda, O. Kitao, H. Nakai, T. Vreven, K. Throssell, J. A. Montgomery, Jr., J. E. Peralta, F. Ogliaro, M. J. Bearpark, J. J. Heyd, E. N. Brothers, K. N. Kudin, V. N. Staroverov, T. A. Keith, R. Kobayashi, J. Normand, K. Raghavachari, A. P. Rendell, J. C. Burant, S. S. Iyengar, J. Tomasi, M. Cossi, J. M. Millam, M. Klene, C. Adamo, R. Cammi, J. W. Ochterski, R. L. Martin, K. Morokuma, O. Farkas, J. B. Foresman, and D. J. Fox, Gaussian, Inc., Wallingford CT, 2016.

[37] Pérez, C.; Lobsiger, S.; Seifert, N. A.; Zaleski, D. P.; Berhane Temelso; Shields, G. C.; Kisiel, Z.; Pate, B. H. Broadband Fourier Transform Rotational Spectroscopy for Structure Determination: The Water Heptamer (Frontiers Article) *Chem. Phys. Lett.* 2013, ***571***, 1-15.

[38] Neill, J. L.; Mikhonin, A. V.; Chen, T.; Sonstrom, R. E.; Pate, B.H. Rapid Quantitation of Isomeric and Dehalogenated Impurities in Pharmaceutical Raw Materials Using MRR Spectroscopy. *J. Pharm. Biomed. Anal.* **2020**, 189, 113474.

[39] Sonstrom, R.E.; Neill, J.L.; Mikhonin, A.V.; Doetzer, R.; Pate, B.H. Chiral analysis of pantolactone with molecular rotational resonance spectroscopy. *Chirality*, **2022**, 34, 114-125.

[40] Mills, M. D.; Sonstrom, R. E.; Vang, Z. P.; Neill, J. L.; Scolati, H. N.; West, C. T.; Pate, B. H.; Clark, J. R. Enantioselective Synthesis of Enantioisotopomers with Quantitative Chiral Analysis by Chiral Tag Rotational Spectroscopy. *Angew. Chem. Int. Ed.* **2022***, 61, e202207275*.

[41] Gordy, W.; Cook, R. L. *Microwave Molecular Spectra*; John Wiley & Sons, Inc., 1984.

[42] Kraitchman, J. “Determination of Molecular Structure from Microwave Spectroscopic Data”, *American Journal of Physics* **1953**, *21*, 17-24.

[43] Sonstrom, R. E.; Zoua Pa Vang; Scolati, H.; Neill, J. L.; Pate, B. H.; Clark, J. R. Rapid Enantiomeric Excess Measurements of Enantioisotopomers by Molecular Rotational Resonance Spectroscopy *Org. Process Res. Dev.* **2023**, *27*, 1185–1197.

[44] Drouin, B. J. Practical uses of SPFIT, *Journal of Molecular Spectroscopy* **2017**, *340*, 1-15.

[45] Z. Kisiel, *Programs for Rotational Spectroscopy (PROSPE)*; Institute of Physics, Polish Academy of Sciences, 2022. http://www.ifpan.edu.pl/~kisiel/prospe.htm.

	Parameter	Value
1	Origin	Varian
2	Solvent	cdcl3
3	Temperature	25.0
4	Pulse Sequence	s2pul
5	Experiment	1D
6	Probe	ASWPFG8319
7	Number of Scans	16
8	Receiver Gain	42
9	Relaxation Delay	10.0000
10	Pulse Width	7.7500
11	Spectrometer Frequency	399.73
12	Spectral Width	6410.3
13	Lowest Frequency	-806.7
14	Nucleus	1H
15	Acquired Size	16384
16	Spectral Size	65536
17	Digital Resolution	0.10

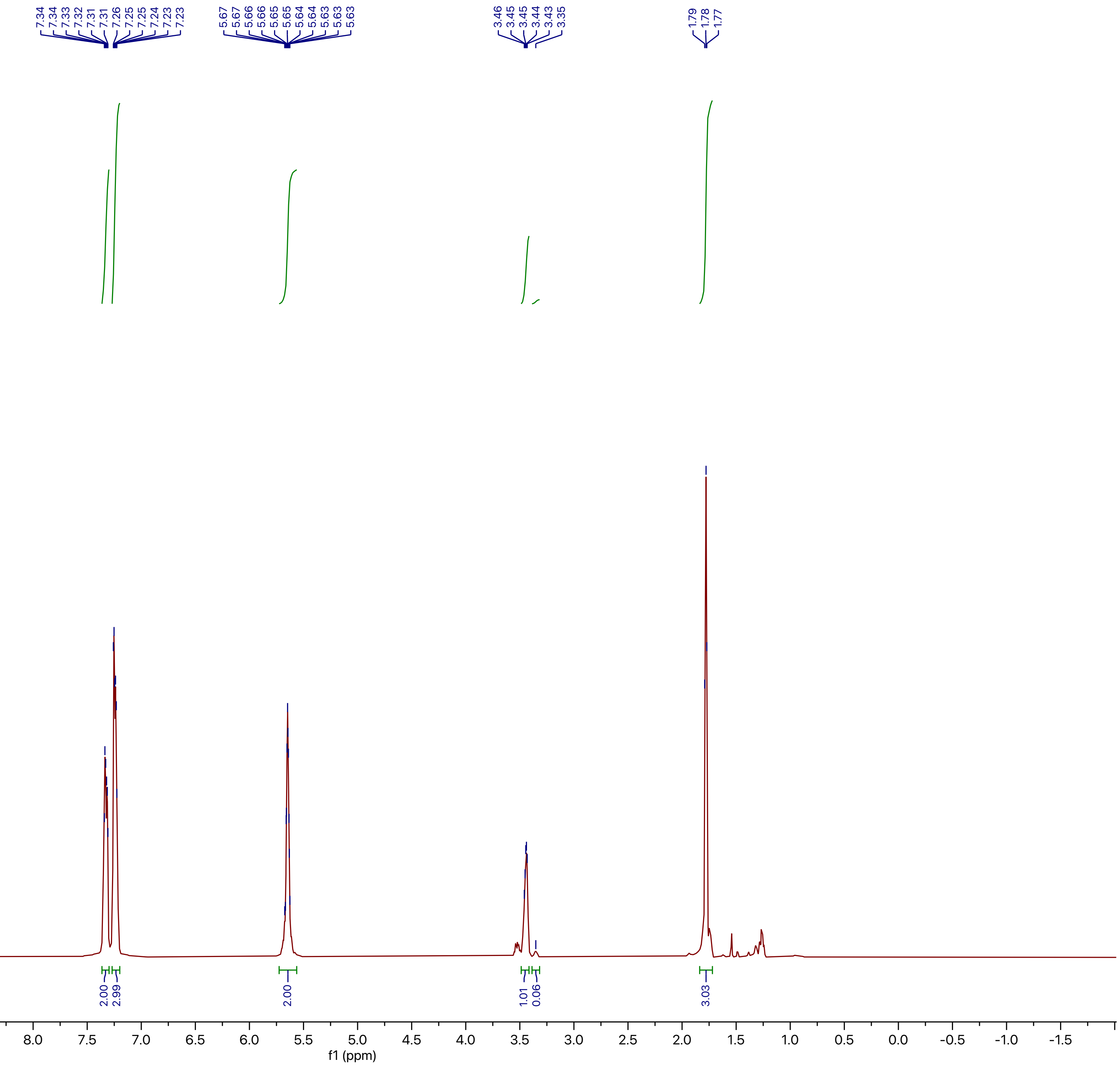

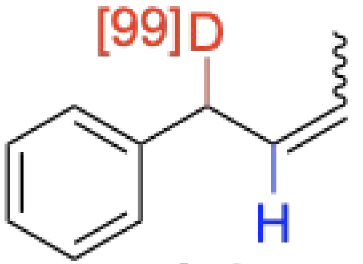

d_1-2a

	Parameter	Value
1	Origin	Varian
2	Solvent	cdcl3
3	Temperature	25.0
4	Pulse Sequence	s2pul
5	Experiment	1D
6	Probe	ASWPFG8319
7	Number of Scans	128
8	Receiver Gain	30
9	Relaxation Delay	0.5000
10	Pulse Width	300.0000
11	Spectrometer Frequency	61.36
12	Spectral Width	552.1
13	Lowest Frequency	4.1
14	Nucleus	lk
15	Acquired Size	614
16	Spectral Size	2048
17	Digital Resolution	0.27

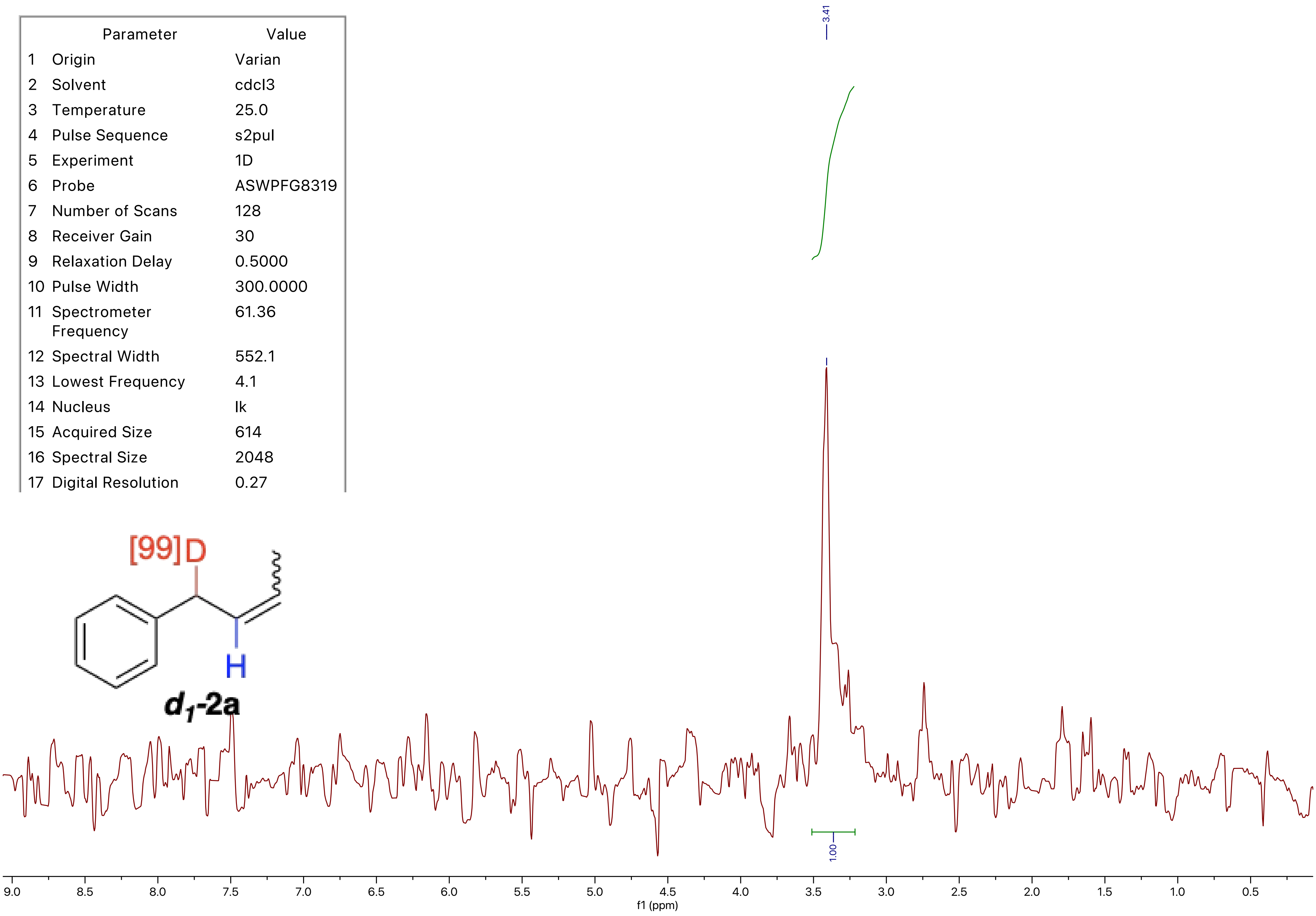

	Parameter	Value
1	Origin	Varian
2	Solvent	cdcl3
3	Temperature	25.0
4	Pulse Sequence	s2pul
5	Experiment	1D
6	Probe	ASWPFG8319
7	Number of Scans	128
8	Receiver Gain	30
9	Relaxation Delay	3.0000
10	Pulse Width	5.7500
11	Spectrometer Frequency	100.52
12	Spectral Width	25000.0
13	Lowest Frequency	-1451.0
14	Nucleus	13C
15	Acquired Size	32768
16	Spectral Size	65536
17	Digital Resolution	0.38

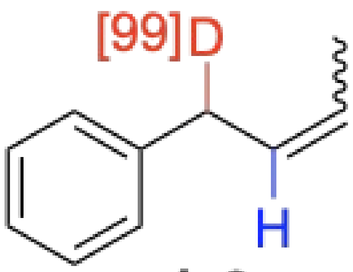

d$_1$-2a

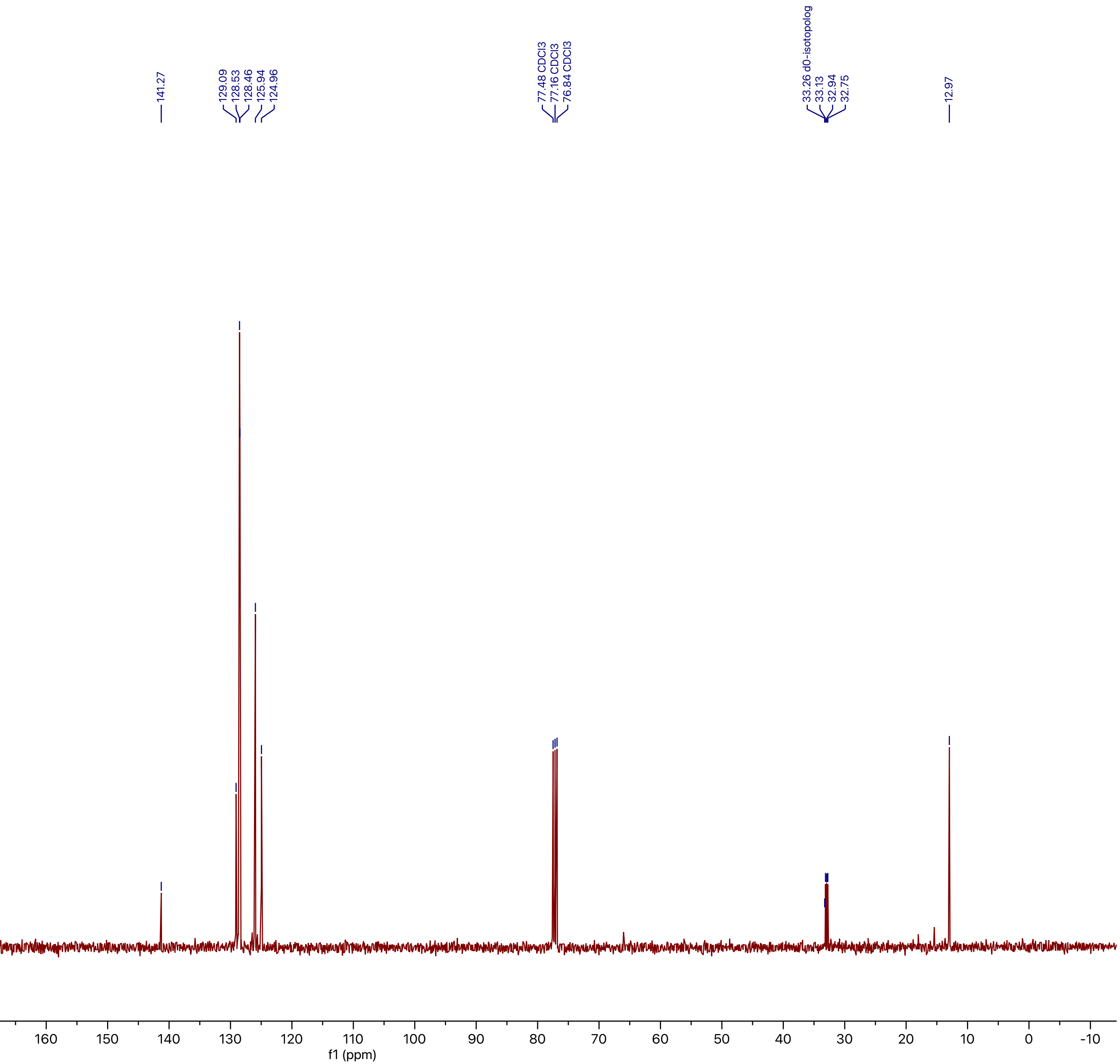

	Parameter	Value
1	Origin	Varian
2	Solvent	cdcl3
3	Temperature	25.0
4	Pulse Sequence	s2pul
5	Experiment	1D
6	Probe	ASWPFG8319
7	Number of Scans	16
8	Receiver Gain	36
9	Relaxation Delay	10.0000
10	Pulse Width	7.7500
11	Spectrometer Frequency	399.73
12	Spectral Width	6410.3
13	Lowest Frequency	-806.7
14	Nucleus	1H
15	Acquired Size	16384
16	Spectral Size	65536
17	Digital Resolution	0.10

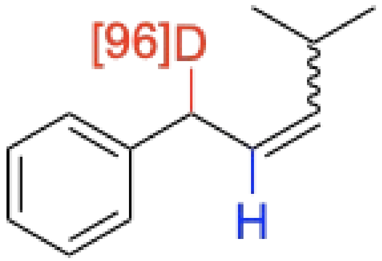

***d*₁-2b**

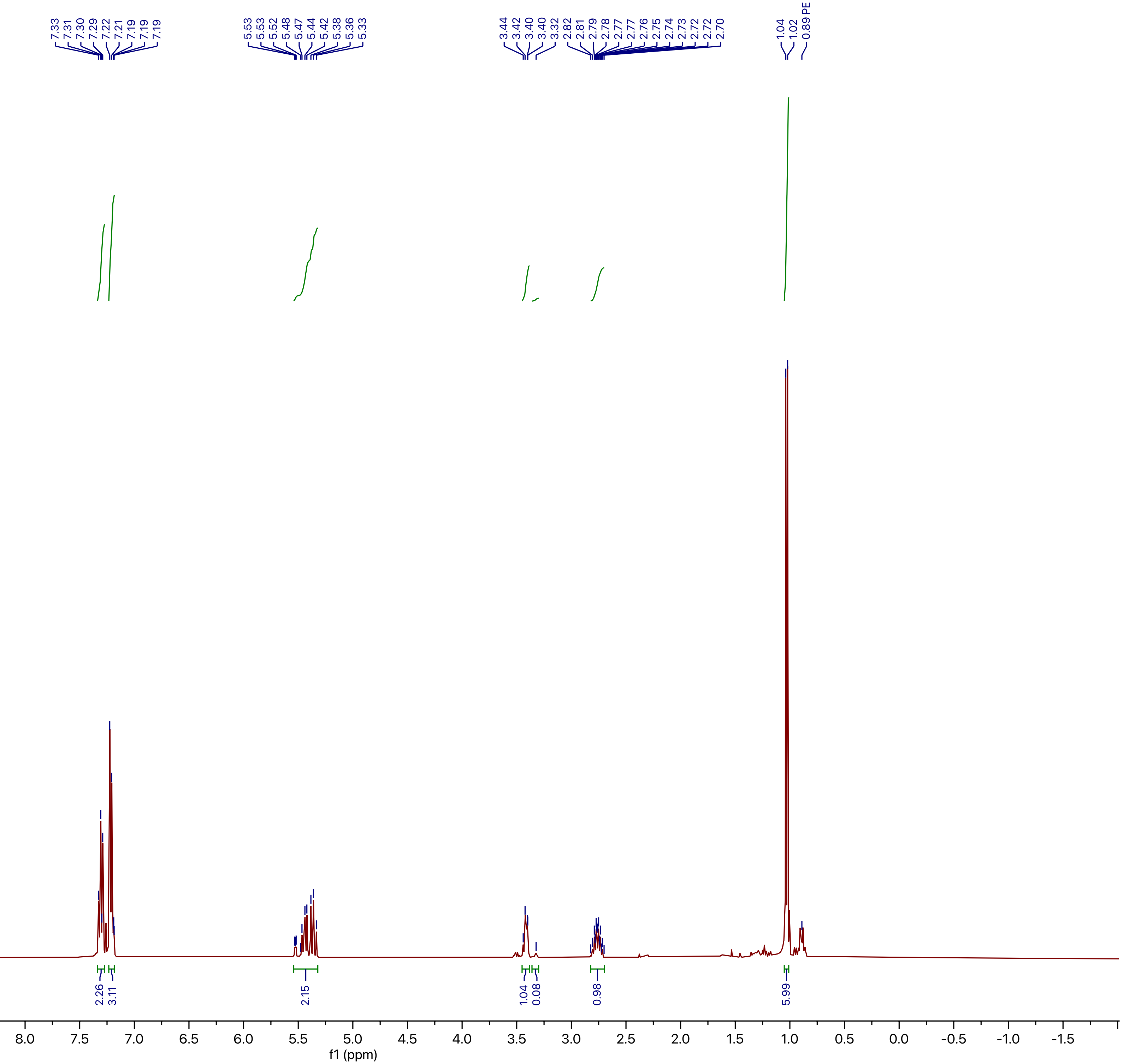

	Parameter	Value
1	Origin	Varian
2	Solvent	cdcl3
3	Temperature	25.0
4	Pulse Sequence	s2pul
5	Experiment	1D
6	Probe	ASWPFG8319
7	Number of Scans	128
8	Receiver Gain	30
9	Relaxation Delay	0.5000
10	Pulse Width	300.0000
11	Spectrometer Frequency	61.36
12	Spectral Width	552.1
13	Lowest Frequency	0.9
14	Nucleus	lk
15	Acquired Size	614
16	Spectral Size	2048
17	Digital Resolution	0.27

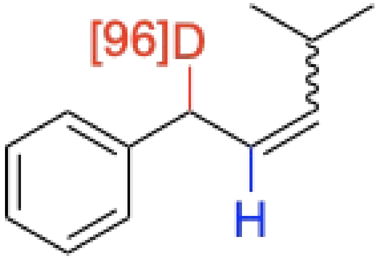

d_1-2b

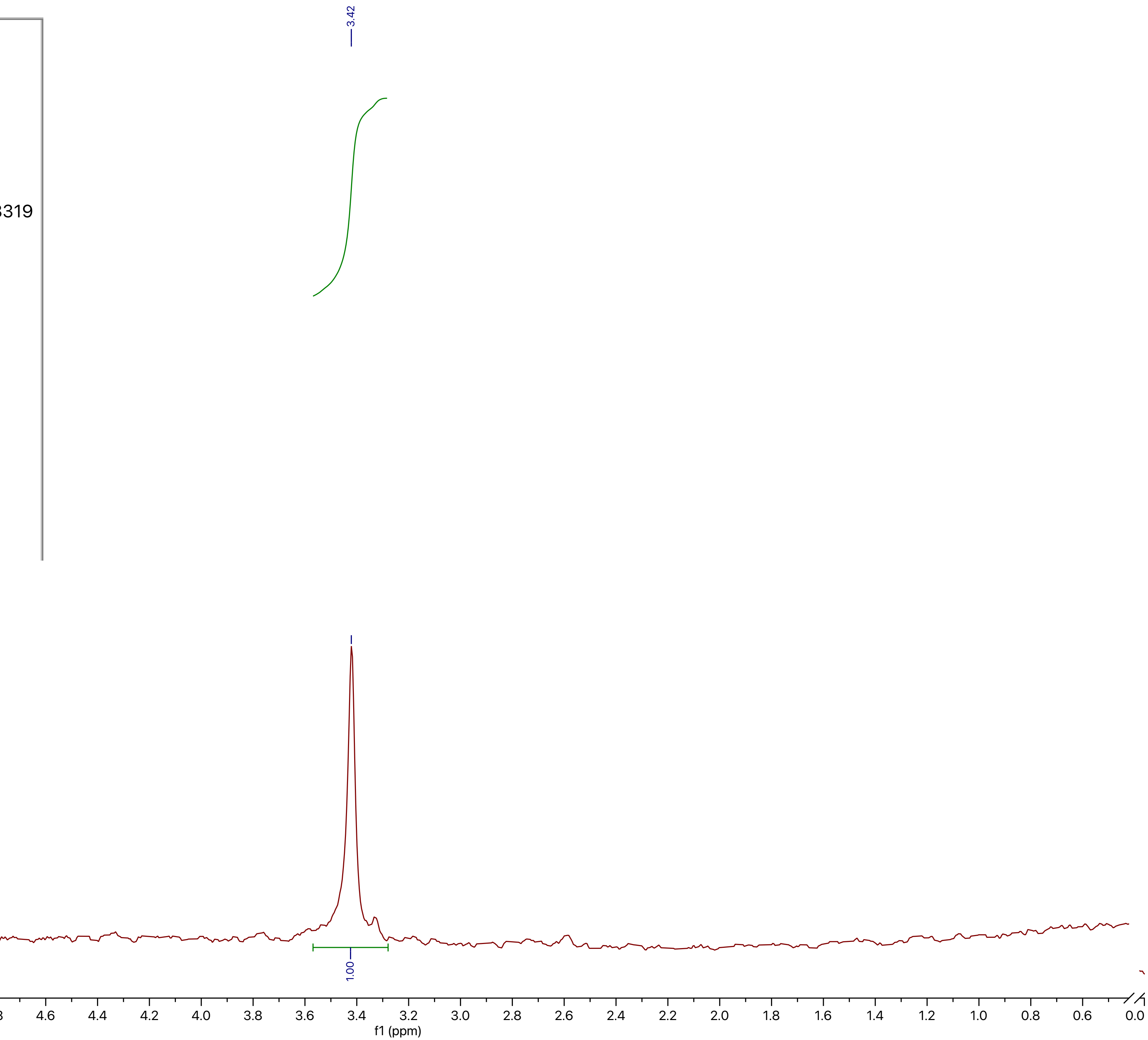

Parameter		Value
1	Origin	Varian
2	Solvent	cdcl3
3	Temperature	25.0
4	Pulse Sequence	s2pul
5	Experiment	1D
6	Probe	ASWPFG8319
7	Number of Scans	256
8	Receiver Gain	30
9	Relaxation Delay	1.0000
10	Pulse Width	5.7500
11	Spectrometer Frequency	100.52
12	Spectral Width	25000.0
13	Lowest Frequency	-1430.7
14	Nucleus	13C
15	Acquired Size	32768
16	Spectral Size	65536
17	Digital Resolution	0.38

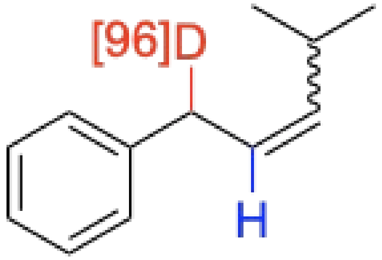

***d*₁-2b**

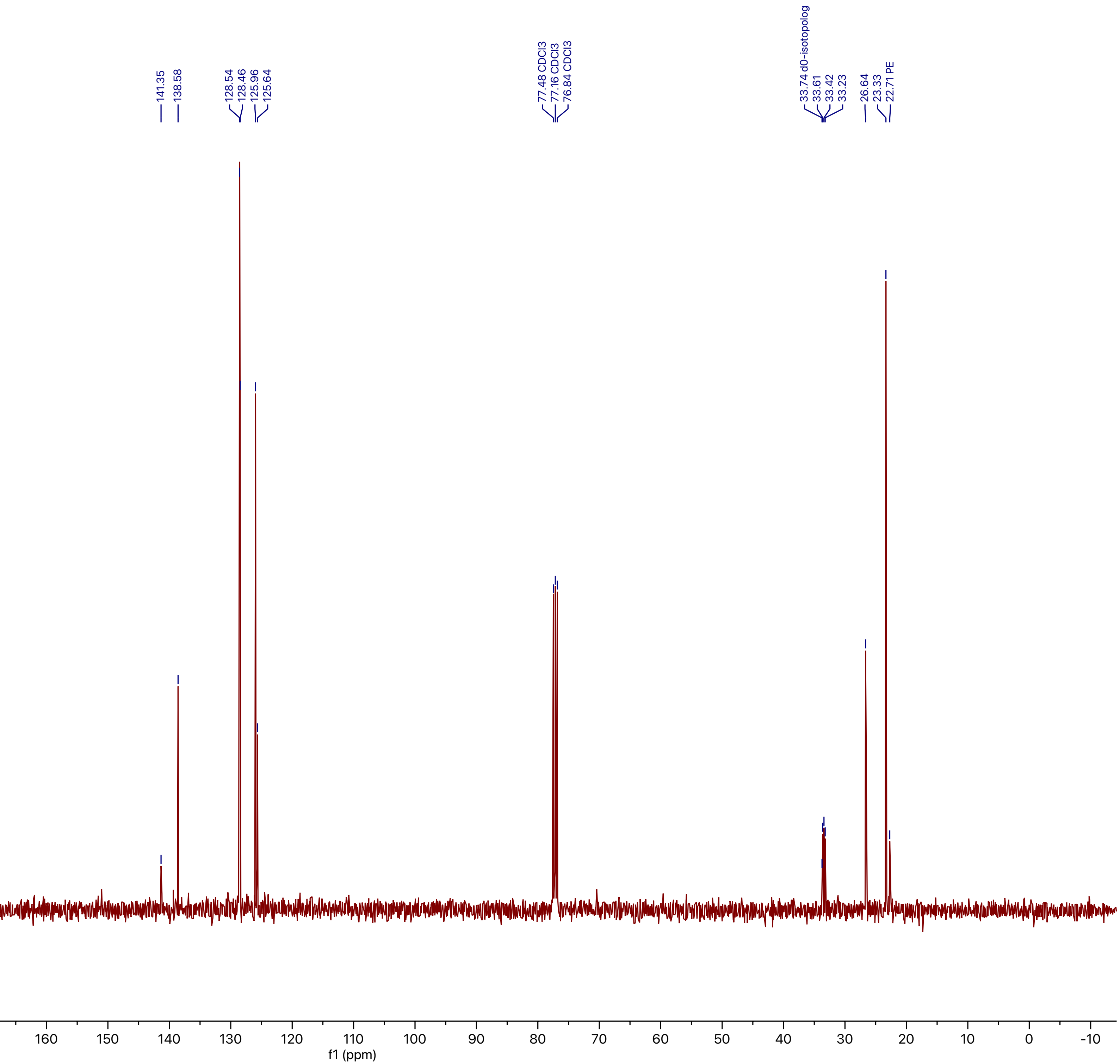

	Parameter	Value
1	Origin	Varian
2	Solvent	cdcl3
3	Temperature	25.0
4	Pulse Sequence	s2pul
5	Experiment	1D
6	Probe	ASWPFG8319
7	Number of Scans	16
8	Receiver Gain	42
9	Relaxation Delay	10.0000
10	Pulse Width	7.7500
11	Spectrometer Frequency	399.73
12	Spectral Width	6410.3
13	Lowest Frequency	-806.2
14	Nucleus	1H
15	Acquired Size	16384
16	Spectral Size	65536
17	Digital Resolution	0.10

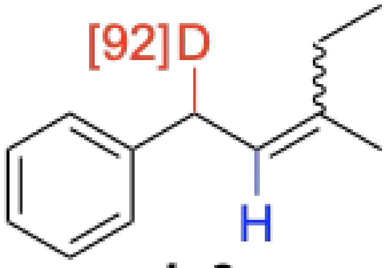

***d*$_1$-2c**

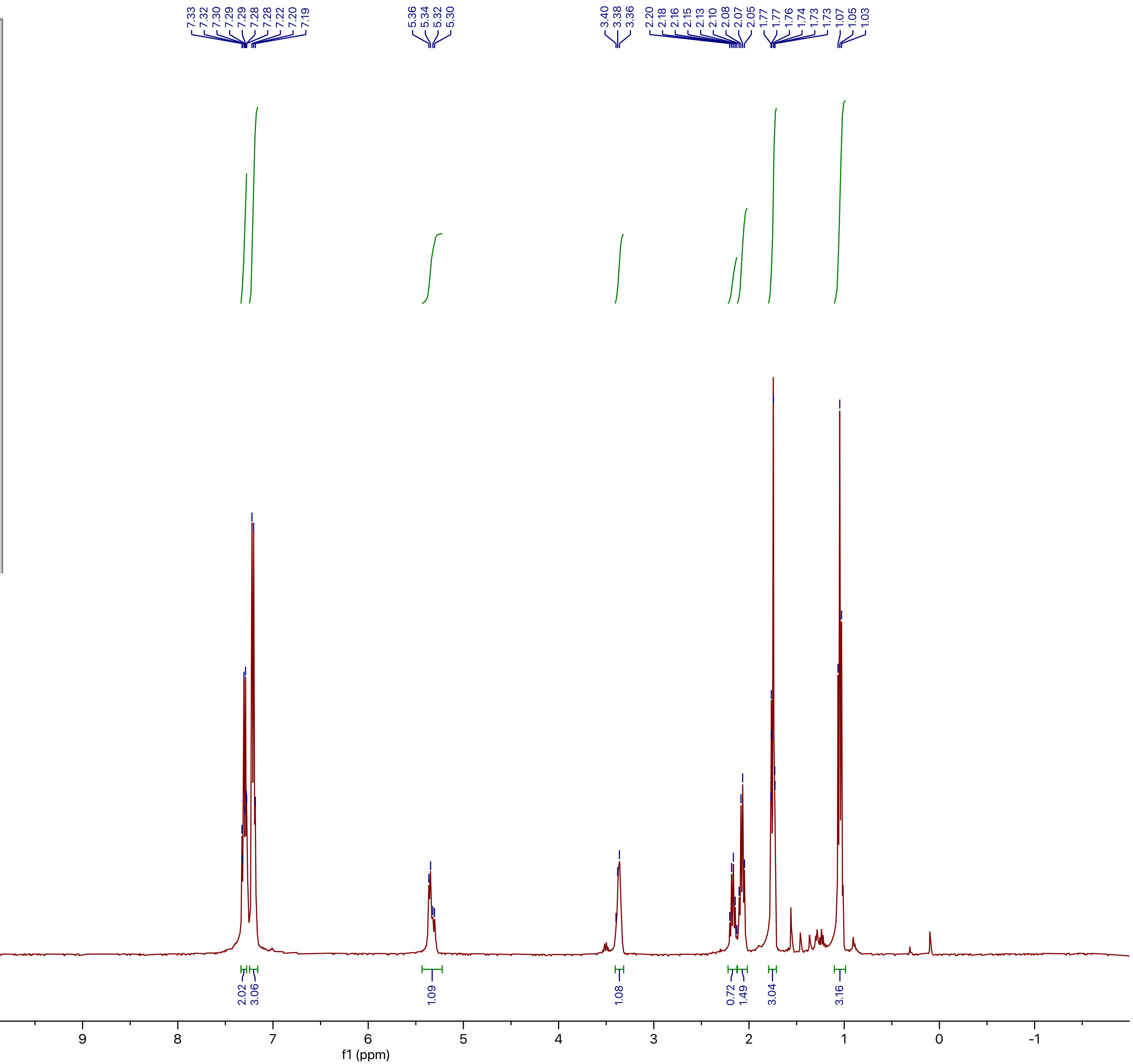

	Parameter	Value
1	Origin	Varian
2	Solvent	cdcl3
3	Temperature	25.0
4	Pulse Sequence	s2pul
5	Experiment	1D
6	Probe	OneNMR_W036
7	Number of Scans	32
8	Receiver Gain	20
9	Relaxation Delay	5.0000
10	Pulse Width	300.0000
11	Spectrometer Frequency	76.71
12	Spectral Width	1535.6
13	Lowest Frequency	-375.2
14	Nucleus	lk
15	Acquired Size	2048
16	Spectral Size	4096
17	Digital Resolution	0.37

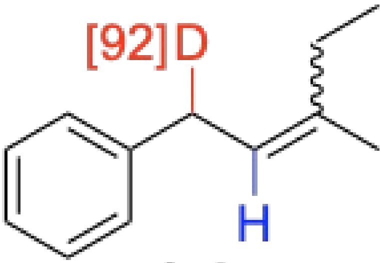

***d*₁-2c**

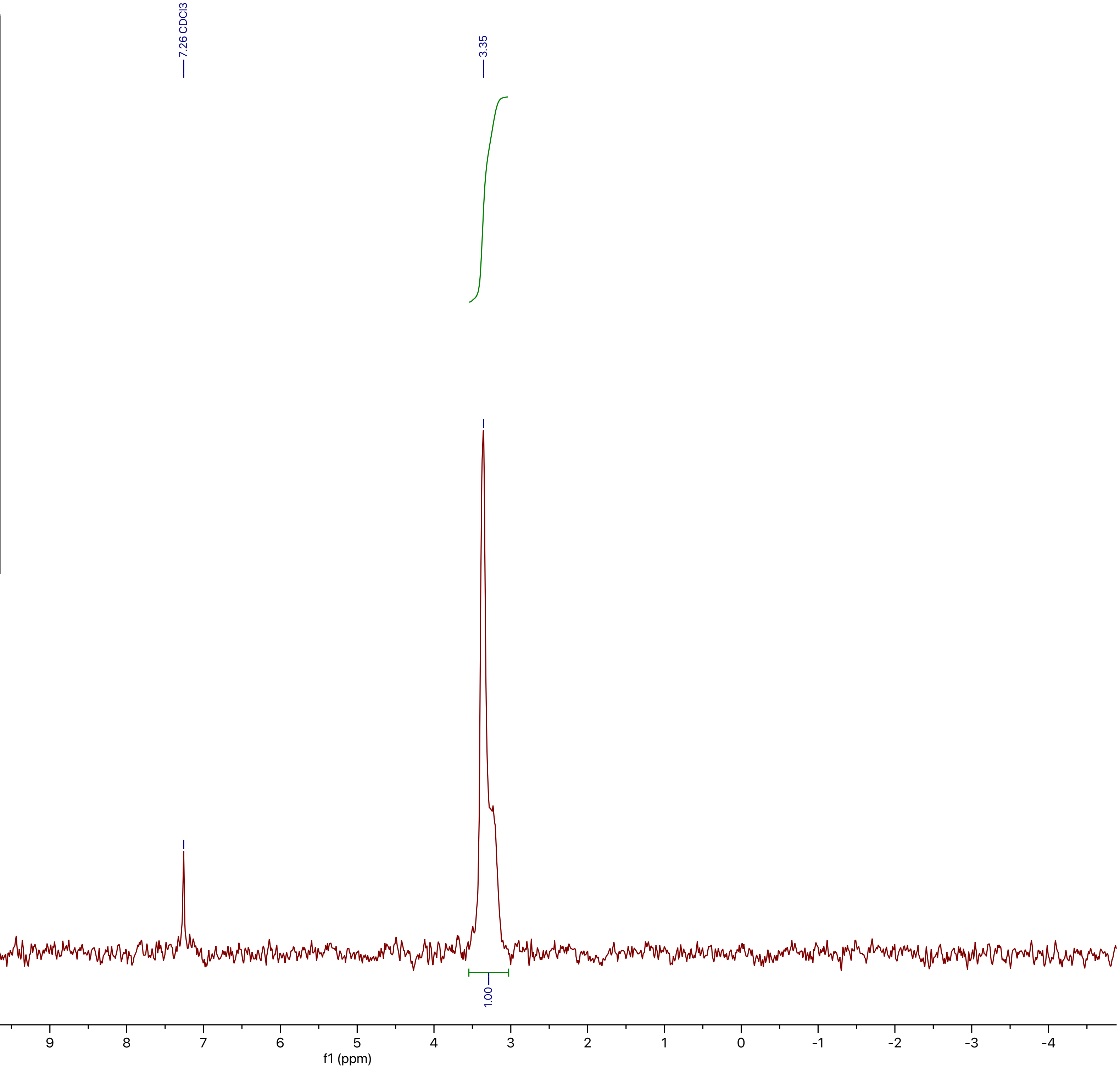

	Parameter	Value
1	Origin	Varian
2	Solvent	cdcl3
3	Temperature	0.0
4	Pulse Sequence	s2pul
5	Experiment	1D
6	Probe	ASWPFG8319
7	Number of Scans	512
8	Receiver Gain	30
9	Relaxation Delay	3.0000
10	Pulse Width	5.7500
11	Spectrometer Frequency	100.52
12	Spectral Width	25000.0
13	Lowest Frequency	-1443.7
14	Nucleus	13C
15	Acquired Size	32768
16	Spectral Size	65536
17	Digital Resolution	0.38

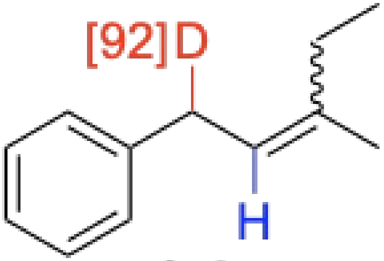

d_1-2c

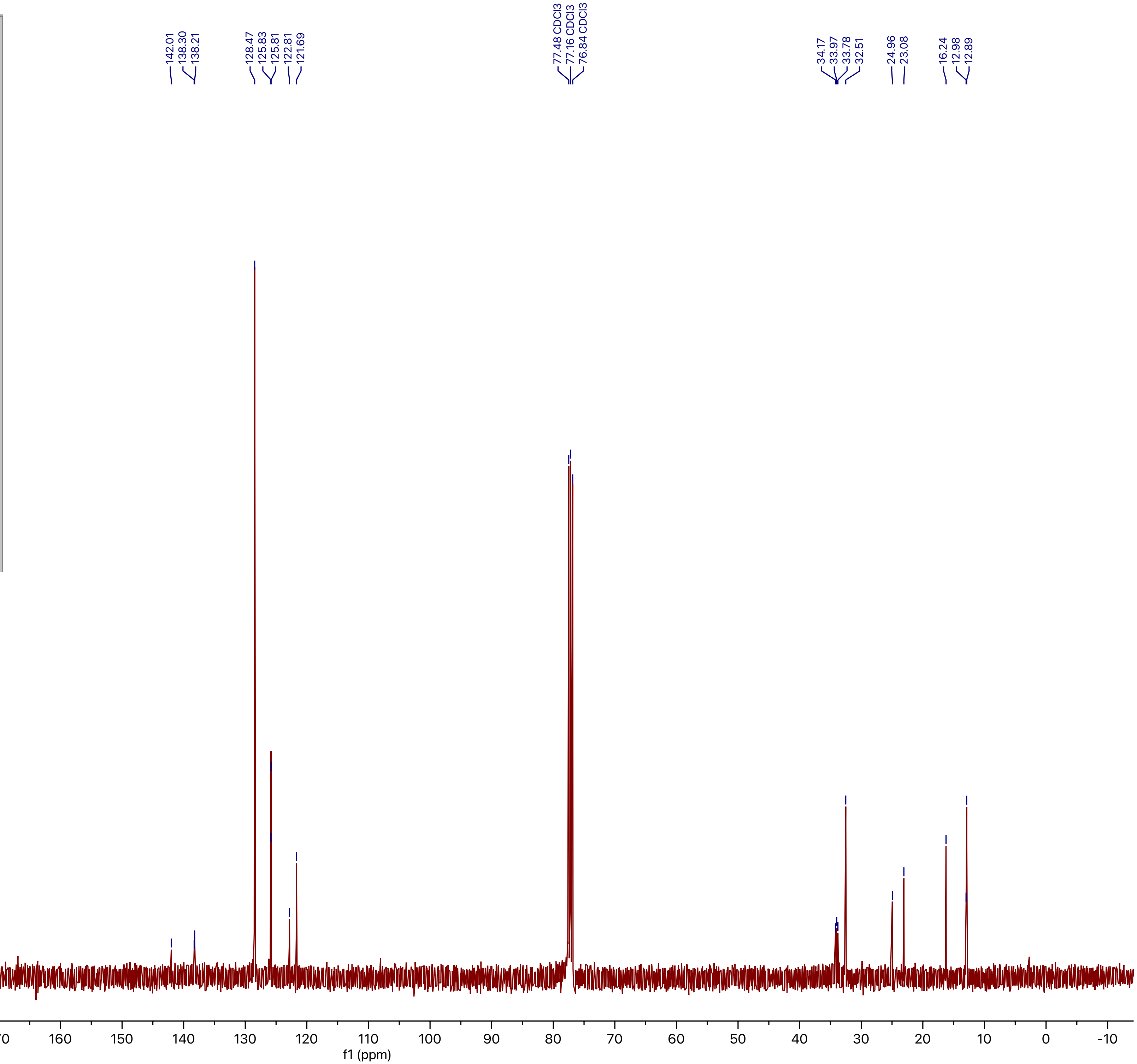

	Parameter	Value
1	Origin	Varian
2	Solvent	cdcl3
3	Temperature	25.0
4	Pulse Sequence	s2pul
5	Experiment	1D
6	Probe	HCN
7	Number of Scans	16
8	Receiver Gain	34
9	Relaxation Delay	10.0000
10	Pulse Width	3.5500
11	Spectrometer Frequency	599.51
12	Spectral Width	9615.4
13	Lowest Frequency	-1209.8
14	Nucleus	1H
15	Acquired Size	16384
16	Spectral Size	65536
17	Digital Resolution	0.15

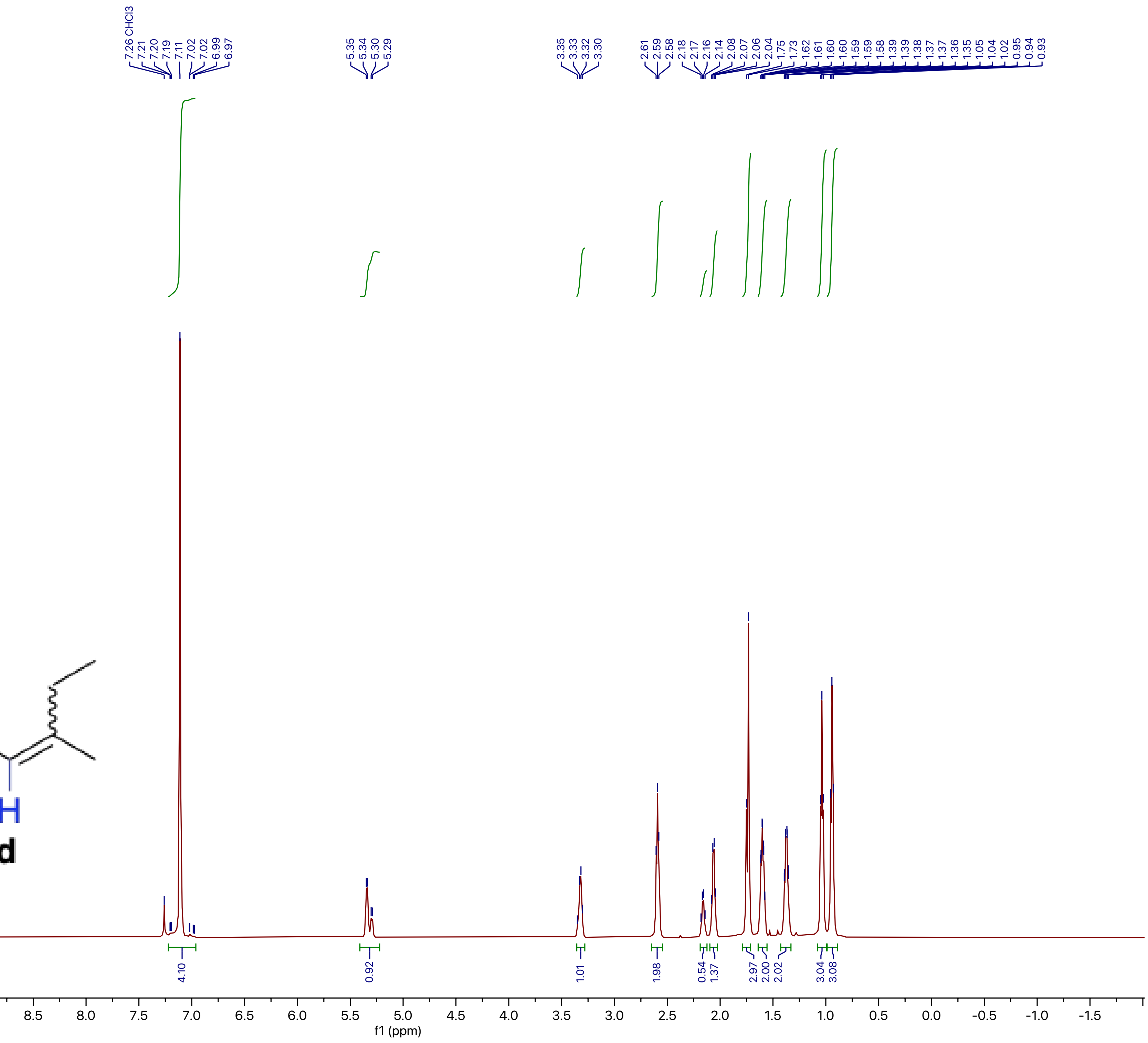

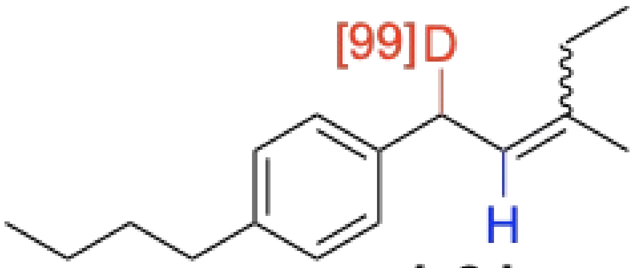

***d*₁-2d**

Parameter		Value
1	Origin	Varian
2	Solvent	cdcl3
3	Temperature	25.0
4	Pulse Sequence	s2pul
5	Experiment	1D
6	Probe	OneNMR_W036
7	Number of Scans	32
8	Receiver Gain	20
9	Relaxation Delay	5.0000
10	Pulse Width	300.0000
11	Spectrometer Frequency	76.71
12	Spectral Width	1535.6
13	Lowest Frequency	-387.5
14	Nucleus	lk
15	Acquired Size	2048
16	Spectral Size	4096
17	Digital Resolution	0.37

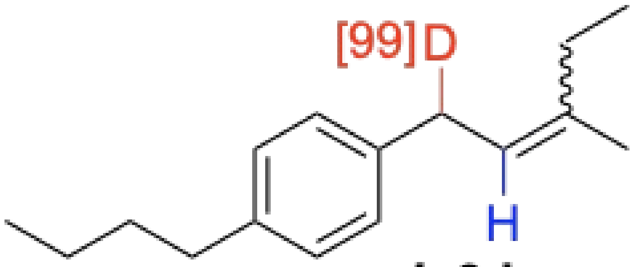

d_1-2d

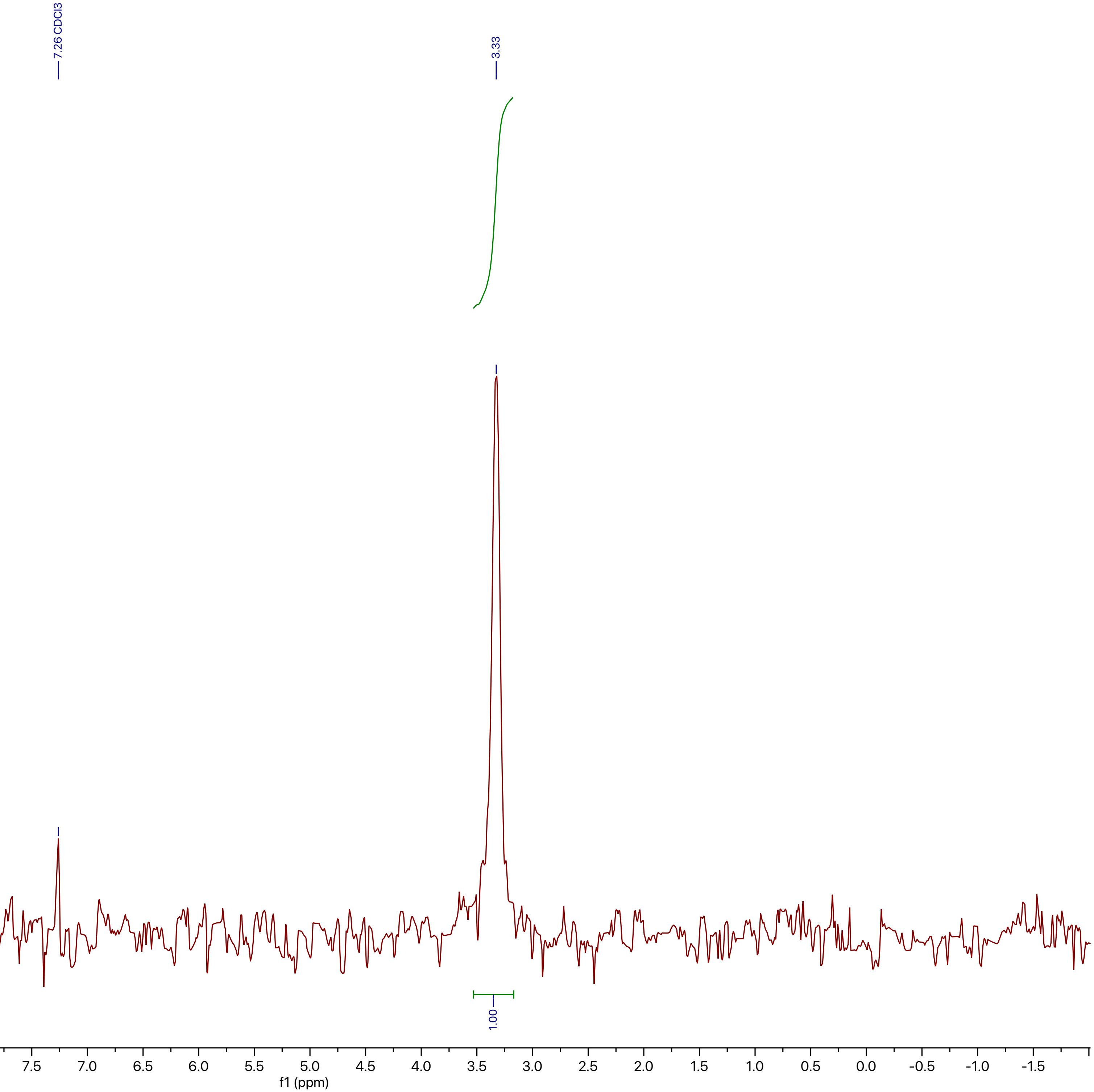

	Parameter	Value
1	Origin	Varian
2	Solvent	cdcl3
3	Temperature	0.0
4	Pulse Sequence	s2pul
5	Experiment	1D
6	Probe	ASWPFG8319
7	Number of Scans	512
8	Receiver Gain	30
9	Relaxation Delay	3.0000
10	Pulse Width	5.7500
11	Spectrometer Frequency	100.52
12	Spectral Width	25000.0
13	Lowest Frequency	-1443.7
14	Nucleus	13C
15	Acquired Size	32768
16	Spectral Size	65536
17	Digital Resolution	0.38

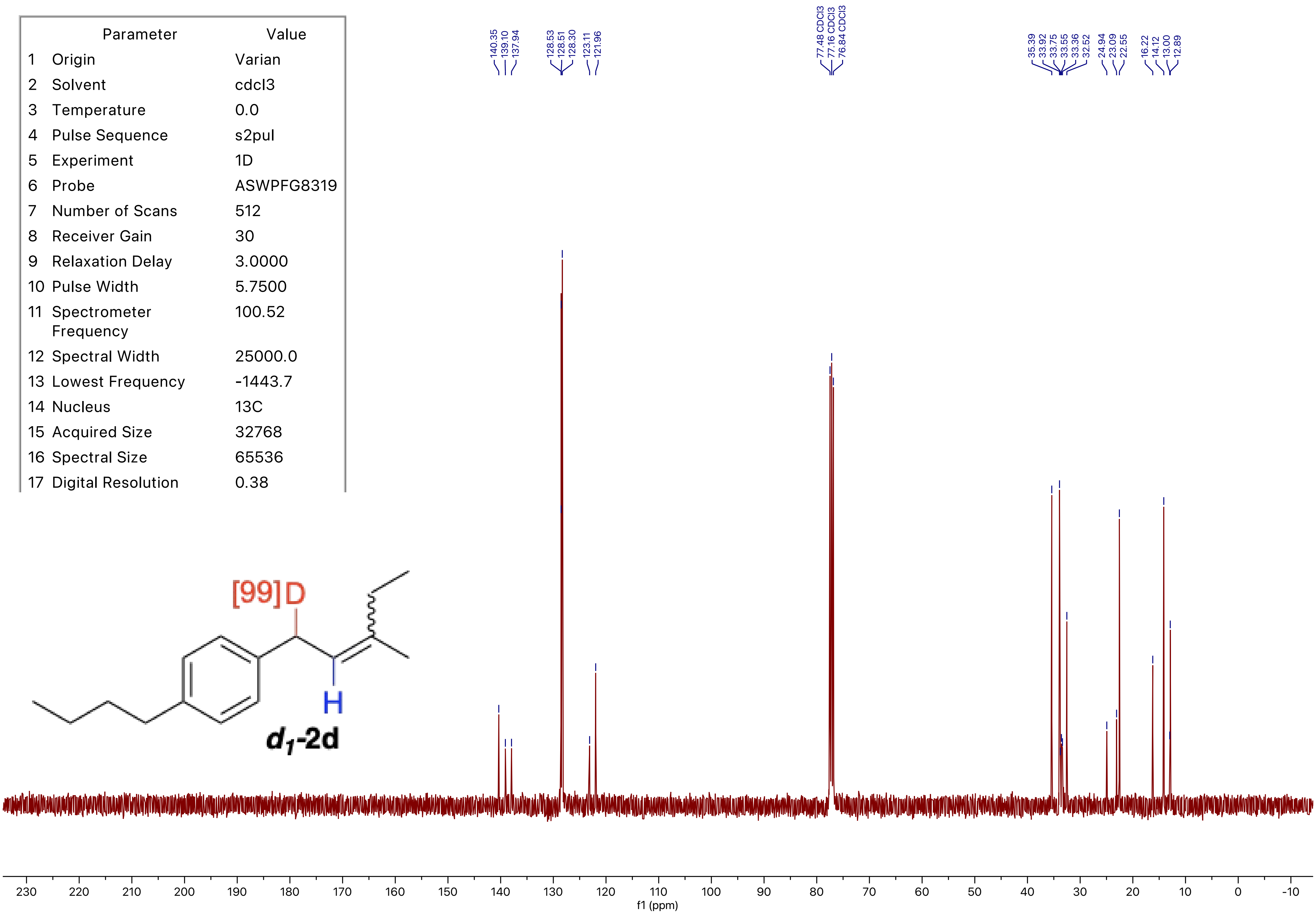

	Parameter	Value
1	Origin	Bruker BioSpin GmbH
2	Instrument	Avance
3	Solvent	CDCl3
4	Temperature	300.0
5	Pulse Sequence	zg30
6	Experiment	1D
7	Probe	Z151574_0073 (PI HR-BBO500S1-BBF/ H/ D-5.0-Z SP)
8	Number of Scans	16
9	Receiver Gain	50.7
10	Relaxation Delay	1.0000
11	Pulse Width	8.0000
12	Spectrometer Frequency	500.21
13	Spectral Width	10000.0
14	Lowest Frequency	-1923.0
15	Nucleus	1H
16	Acquired Size	32768
17	Spectral Size	65536

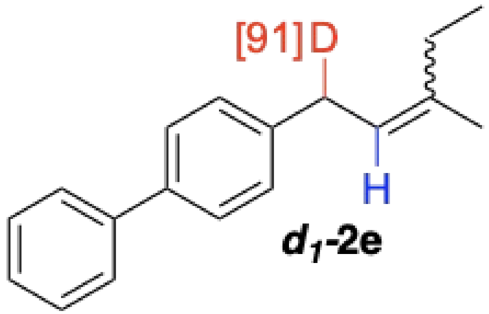

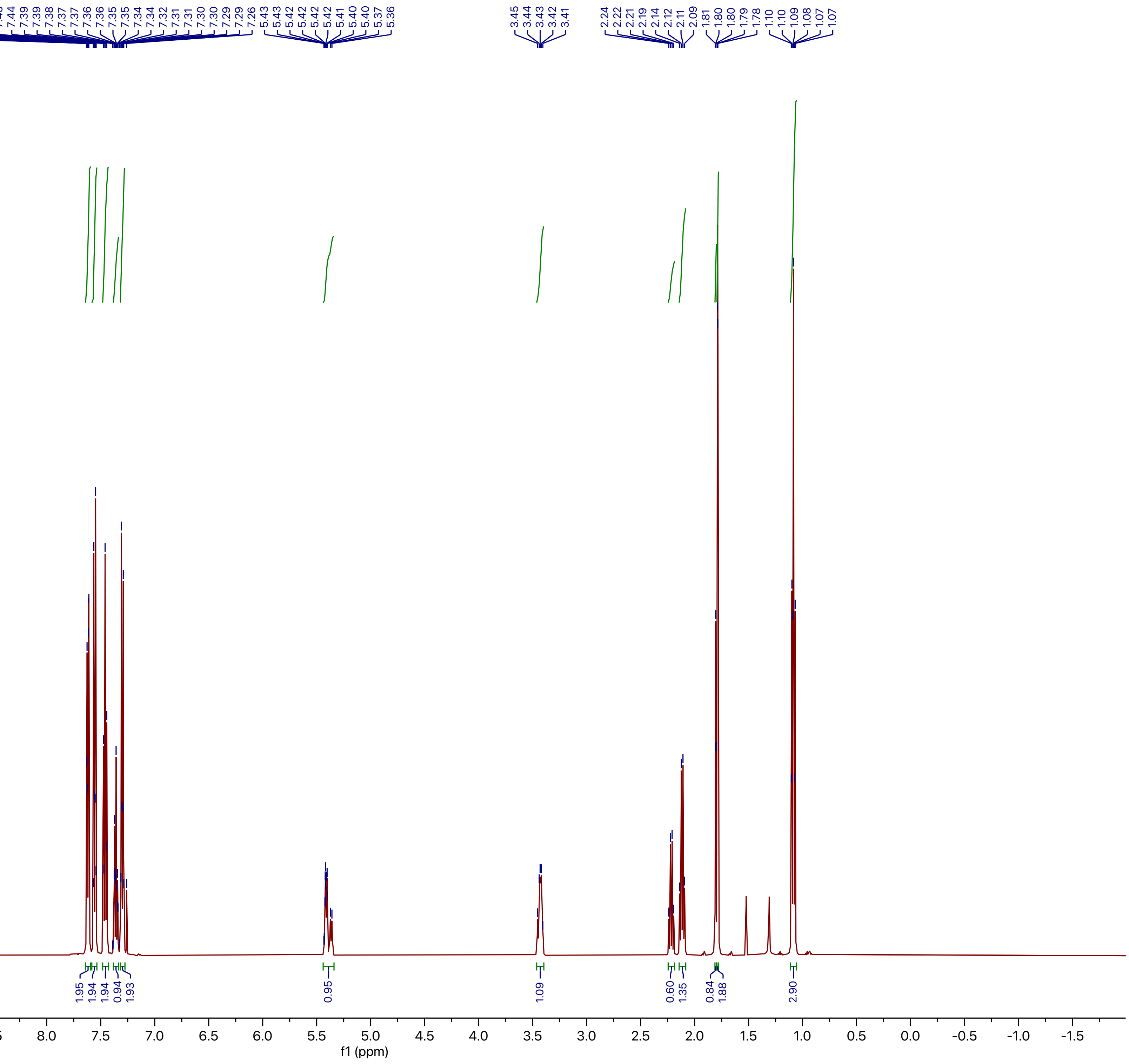

	Parameter	Value
1	Origin	Varian
2	Solvent	cdcl3
3	Temperature	25.0
4	Pulse Sequence	s2pul
5	Experiment	1D
6	Probe	OneNMR_W036
7	Number of Scans	32
8	Receiver Gain	20
9	Relaxation Delay	5.0000
10	Pulse Width	300.0000
11	Spectrometer Frequency	76.71
12	Spectral Width	1535.6
13	Lowest Frequency	-378.9
14	Nucleus	lk
15	Acquired Size	2048
16	Spectral Size	4096
17	Digital Resolution	0.37

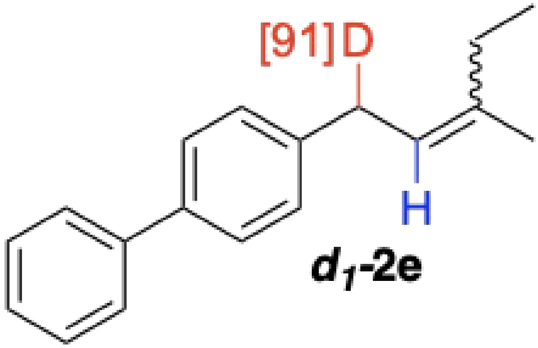

d_1-**2e**

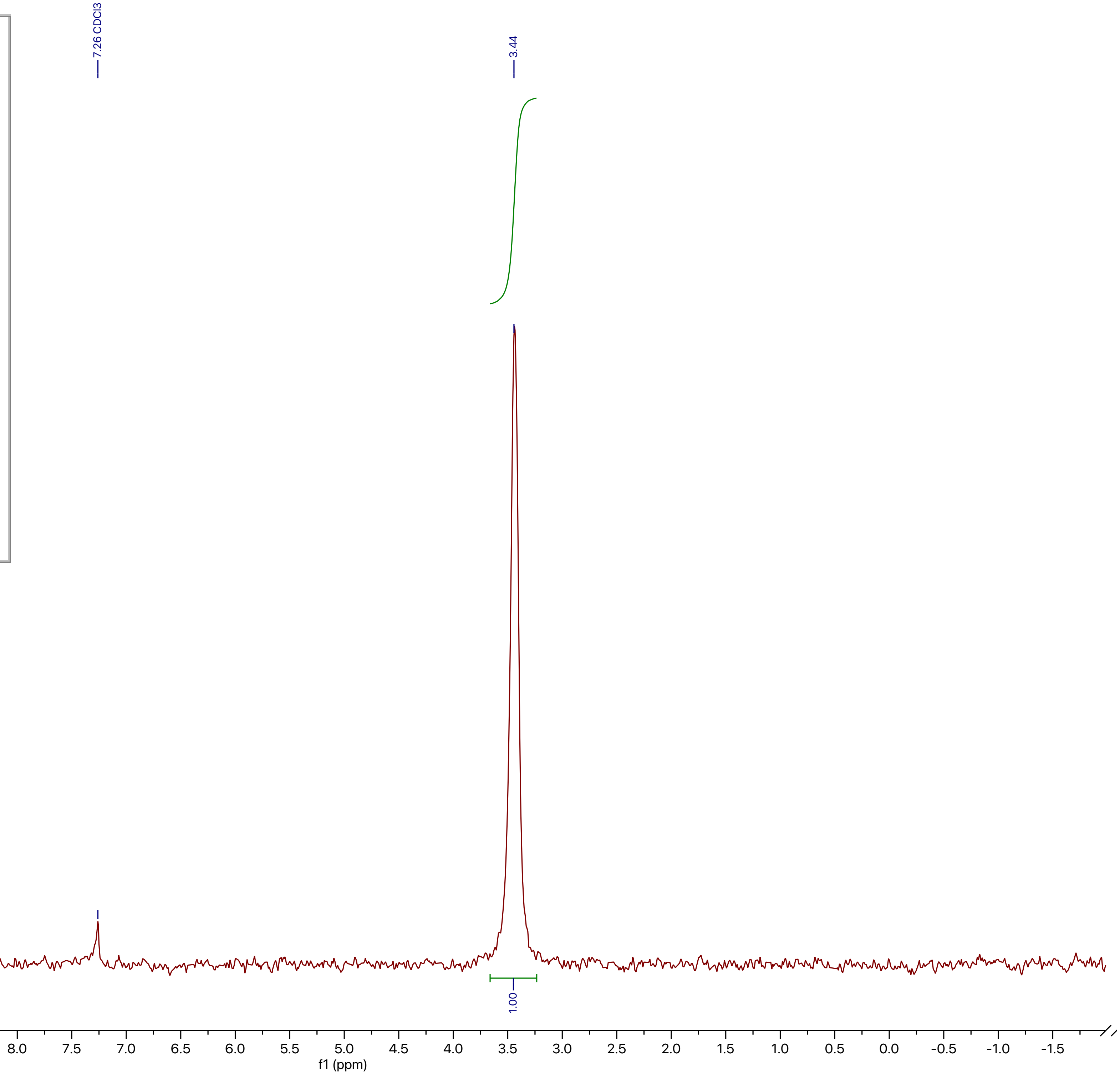

	Parameter	Value
1	Origin	Bruker BioSpin GmbH
2	Instrument	Avance
3	Solvent	CDCl3
4	Temperature	300.0
5	Pulse Sequence	zgpg30
6	Experiment	1D
7	Probe	Z151574_0073 (PI HR-BBO500S1-BBF/ H/ D-5.0-Z SP)
8	Number of Scans	100
9	Receiver Gain	101.0
10	Relaxation Delay	2.0000
11	Pulse Width	9.0000
12	Spectrometer Frequency	125.79
13	Spectral Width	30120.5
14	Lowest Frequency	-2470.3
15	Nucleus	13C
16	Acquired Size	32768
17	Spectral Size	65536

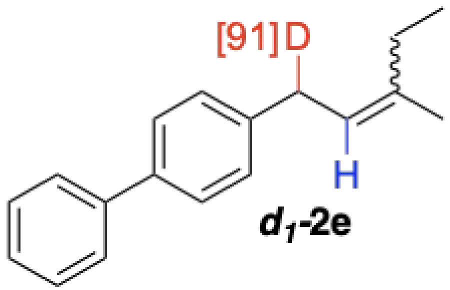

d$_1$-2e

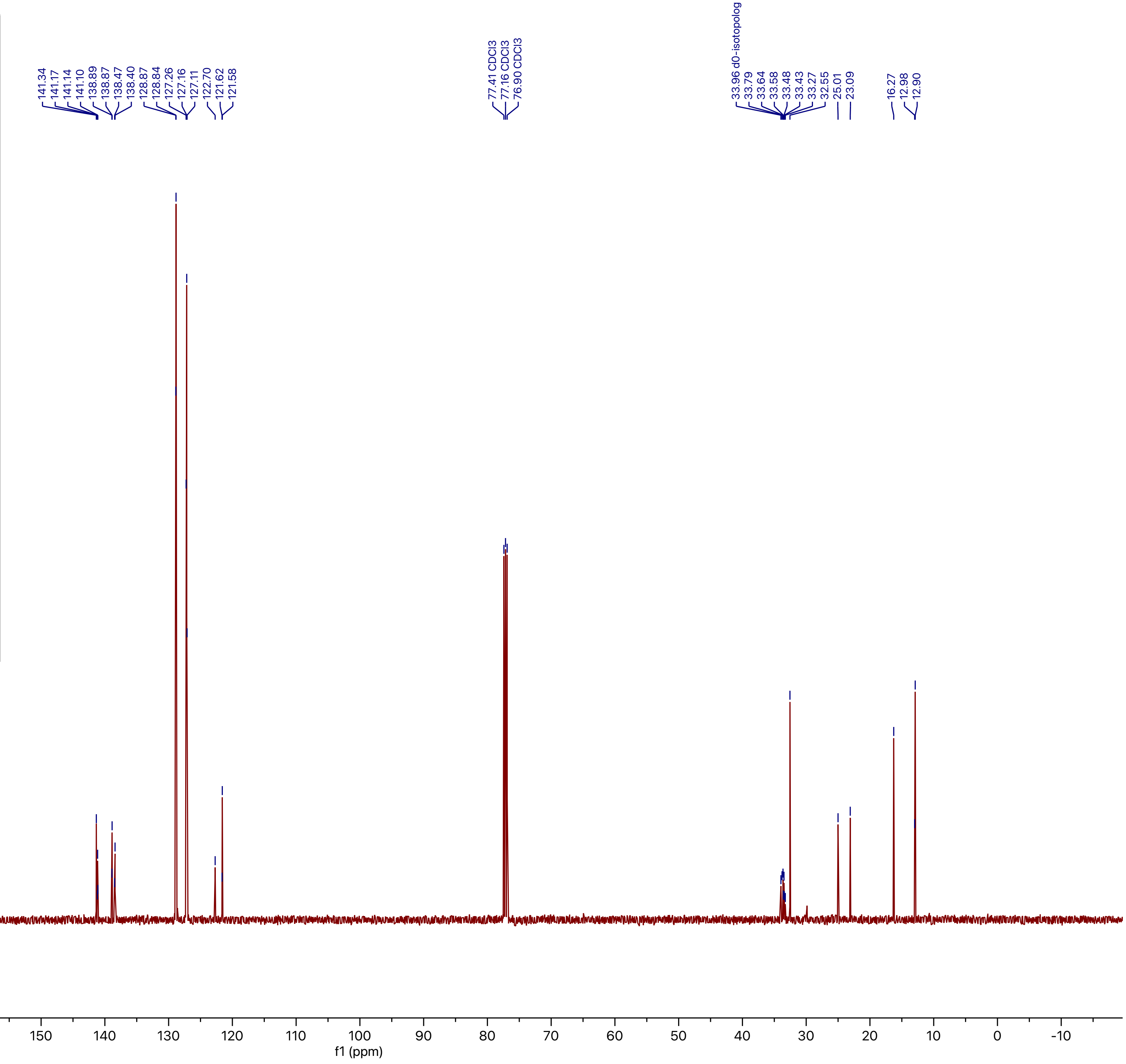

	Parameter	Value
1	Origin	Varian
2	Solvent	cdcl3
3	Temperature	0.0
4	Pulse Sequence	s2pul
5	Experiment	1D
6	Probe	ASWPFG8319
7	Number of Scans	16
8	Receiver Gain	46
9	Relaxation Delay	5.0000
10	Pulse Width	7.7500
11	Spectrometer Frequency	399.73
12	Spectral Width	6410.3
13	Lowest Frequency	-805.1
14	Nucleus	1H
15	Acquired Size	16384
16	Spectral Size	65536
17	Digital Resolution	0.10

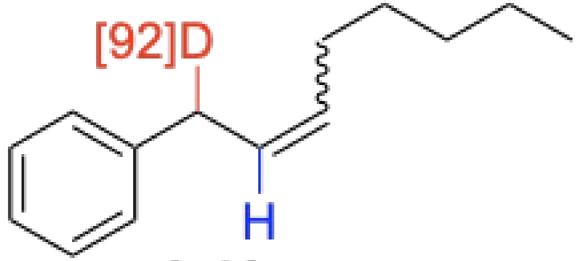

d_1-2f

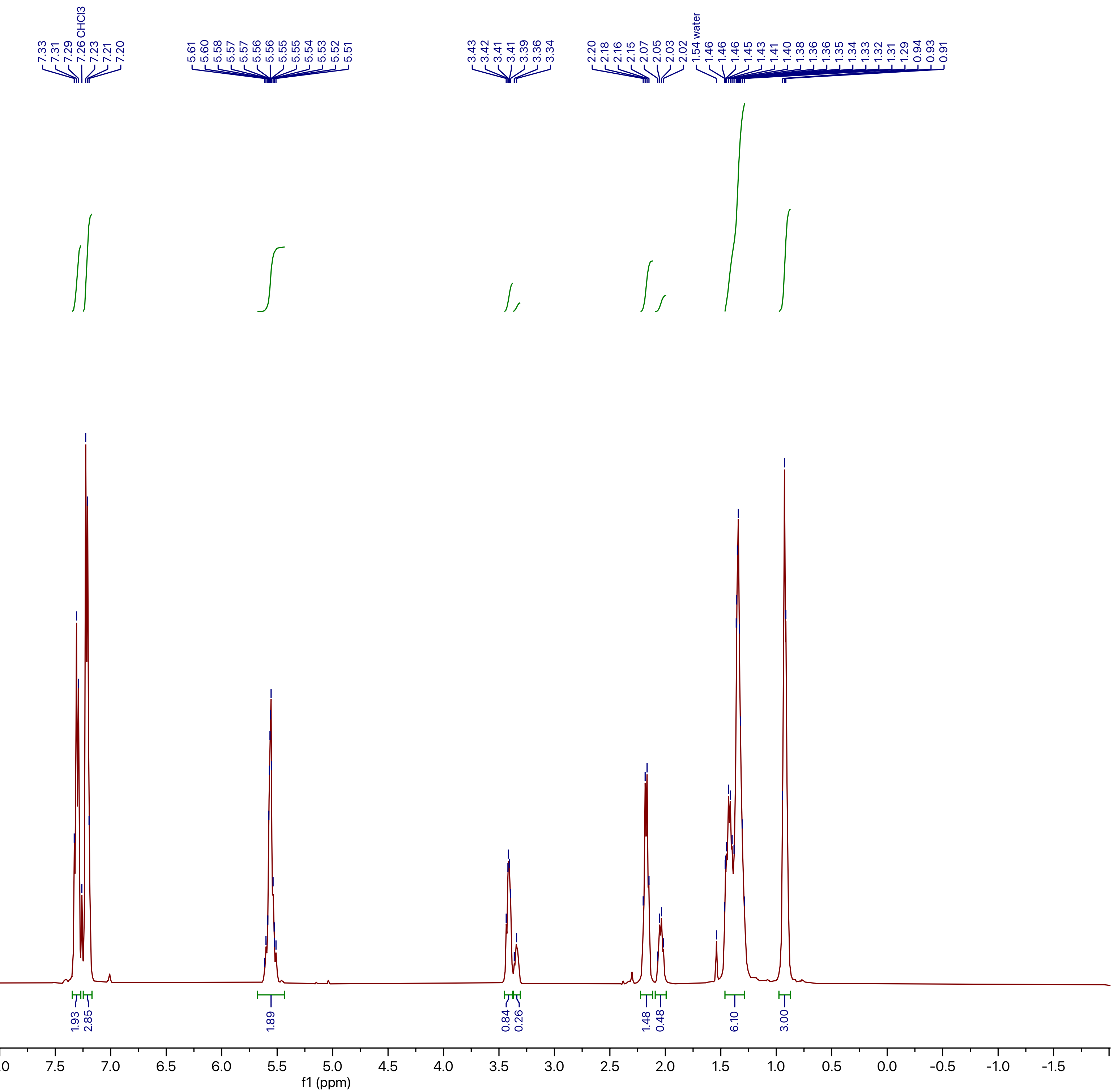

	Parameter	Value
1	Origin	Varian
2	Solvent	cdcl3
3	Temperature	0.0
4	Pulse Sequence	s2pul
5	Experiment	1D
6	Probe	ASWPFG8319
7	Number of Scans	128
8	Receiver Gain	30
9	Relaxation Delay	0.5000
10	Pulse Width	300.0000
11	Spectrometer Frequency	61.36
12	Spectral Width	552.1
13	Lowest Frequency	3.6
14	Nucleus	lk
15	Acquired Size	614
16	Spectral Size	2048
17	Digital Resolution	0.27

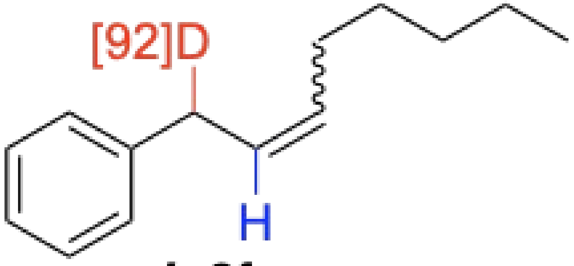

***d*$_1$-2f**

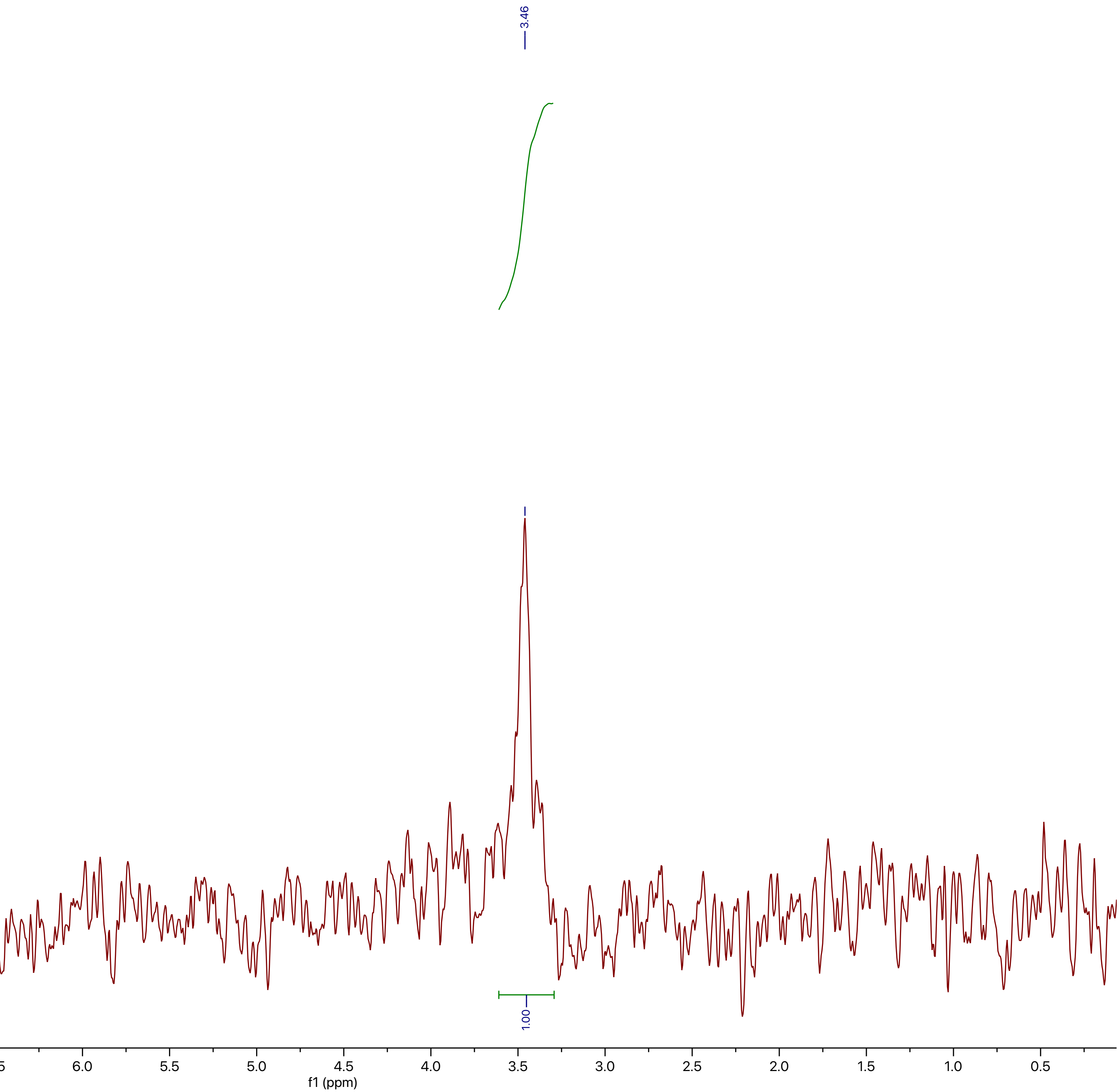

	Parameter	Value
1	Origin	Varian
2	Solvent	cdcl3
3	Temperature	0.0
4	Pulse Sequence	s2pul
5	Experiment	1D
6	Probe	ASWPFG8319
7	Number of Scans	512
8	Receiver Gain	30
9	Relaxation Delay	3.0000
10	Pulse Width	5.7500
11	Spectrometer Frequency	100.52
12	Spectral Width	25000.0
13	Lowest Frequency	-1431.1
14	Nucleus	13C
15	Acquired Size	32768
16	Spectral Size	65536
17	Digital Resolution	0.38

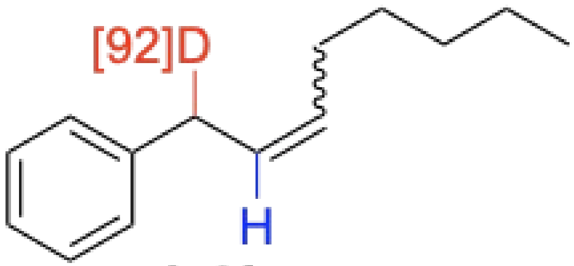

*d*1-2f

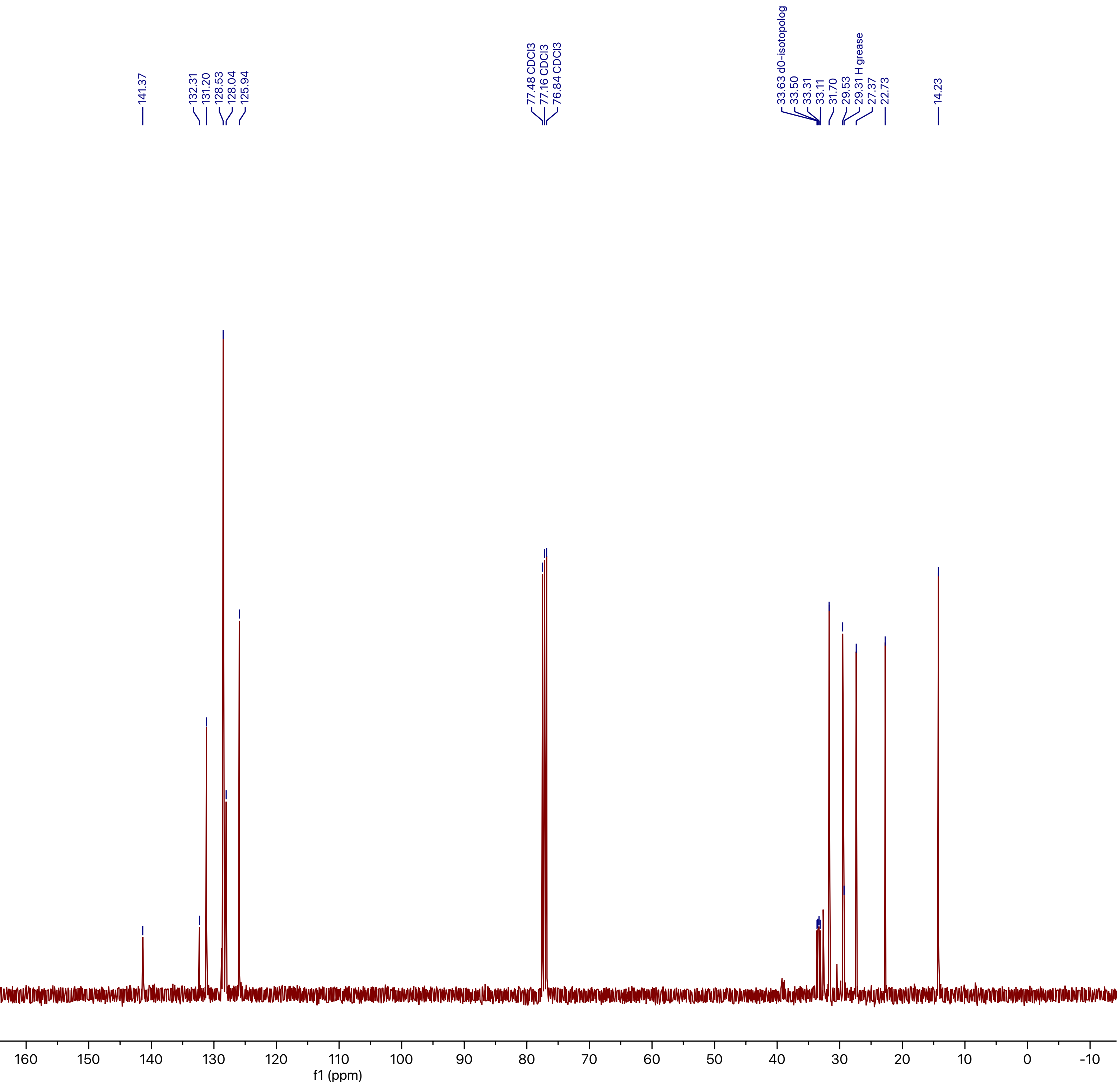

	Parameter	Value
1	Origin	Bruker BioSpin GmbH
2	Instrument	Avance
3	Solvent	CDCl3
4	Temperature	300.0
5	Pulse Sequence	zg30
6	Experiment	1D
7	Probe	Z151574_0073 (PI HR-BBO500S1-BBF/ H/ D-5.0-Z SP)
8	Number of Scans	16
9	Receiver Gain	57.8
10	Relaxation Delay	1.0000
11	Pulse Width	8.0000
12	Spectrometer Frequency	500.21
13	Spectral Width	10000.0
14	Lowest Frequency	-1924.8
15	Nucleus	1H
16	Acquired Size	32768
17	Spectral Size	65536

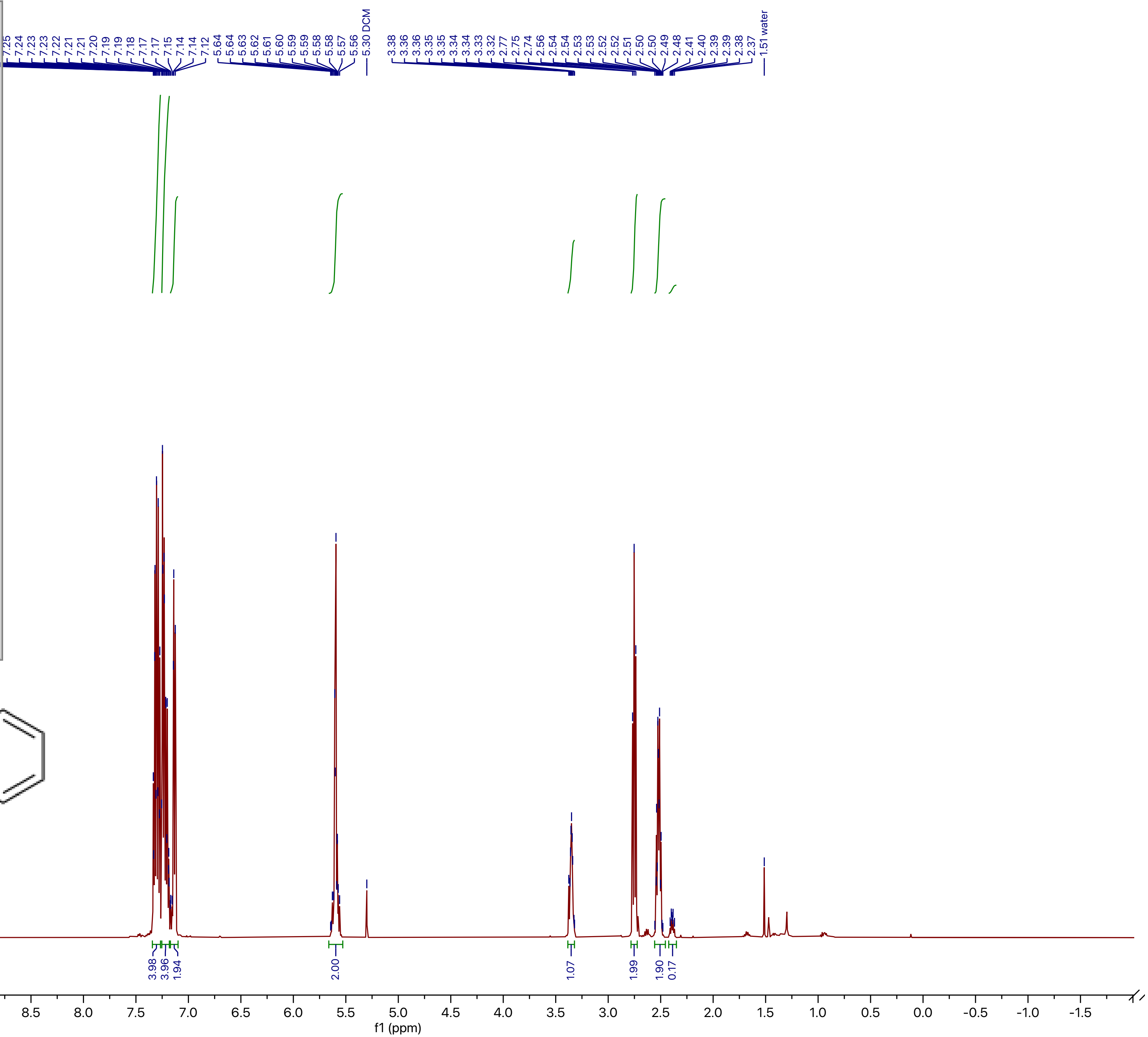

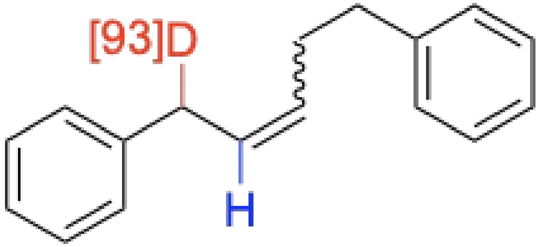

***d*$_1$-2g**

	Parameter	Value
1	Origin	Varian
2	Solvent	cdcl3
3	Temperature	25.0
4	Pulse Sequence	s2pul
5	Experiment	1D
6	Probe	OneNMR_W036
7	Number of Scans	32
8	Receiver Gain	20
9	Relaxation Delay	5.0000
10	Pulse Width	300.0000
11	Spectrometer Frequency	76.71
12	Spectral Width	1535.6
13	Lowest Frequency	-384.2
14	Nucleus	lk
15	Acquired Size	2048
16	Spectral Size	4096
17	Digital Resolution	0.37

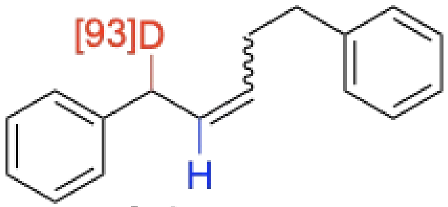

d_1-2g

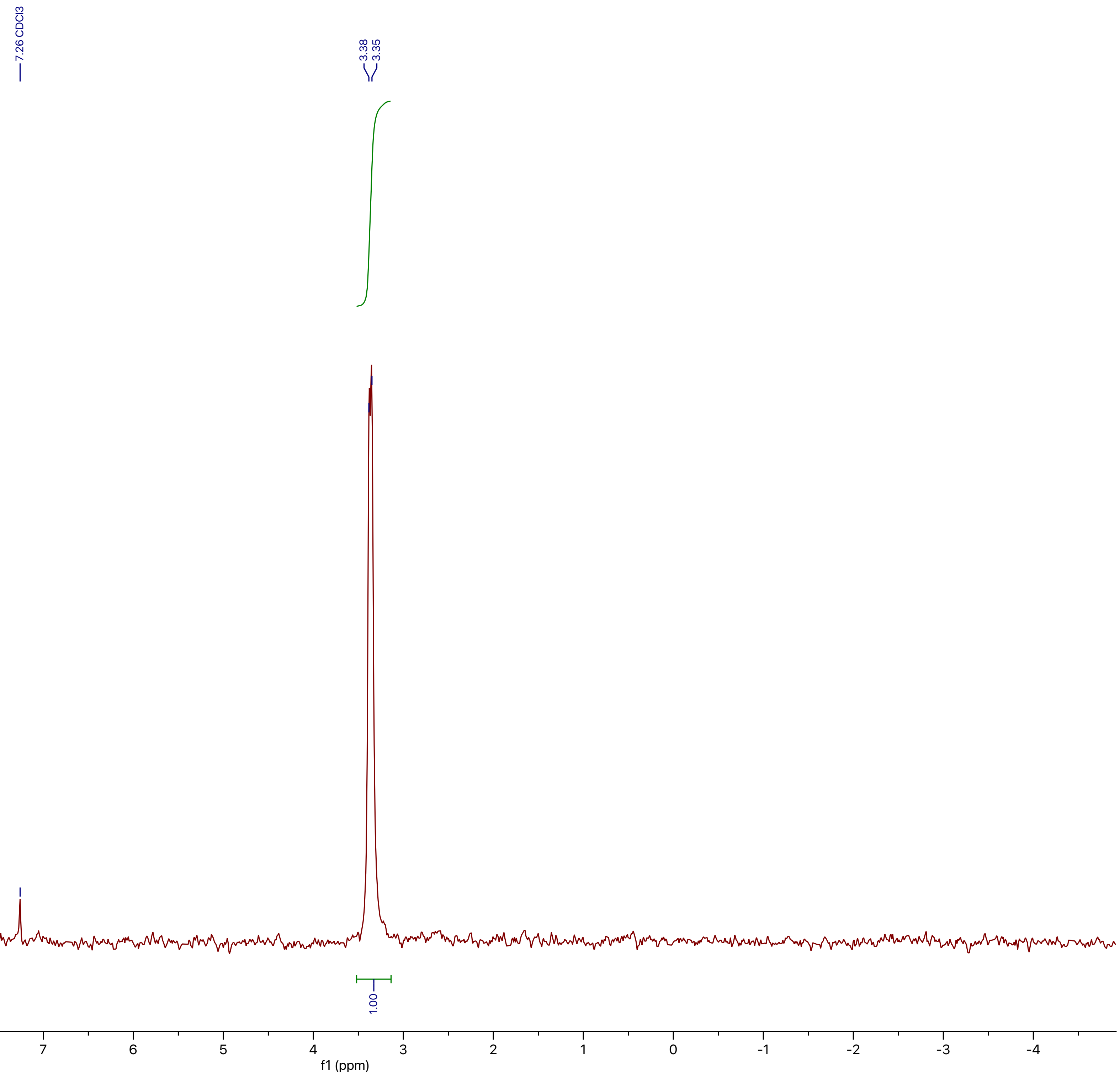

	Parameter	Value
1	Origin	Bruker BioSpin GmbH
2	Instrument	Avance
3	Solvent	CDCl3
4	Temperature	300.0
5	Pulse Sequence	zgpg30
6	Experiment	1D
7	Probe	Z151574_0073 (PI HR-BBO500S1-BBF/ H/ D-5.0-Z SP)
8	Number of Scans	1024
9	Receiver Gain	101.0
10	Relaxation Delay	2.0000
11	Pulse Width	9.0000
12	Spectrometer Frequency	125.79
13	Spectral Width	30120.5
14	Lowest Frequency	-2469.2
15	Nucleus	13C
16	Acquired Size	32768
17	Spectral Size	65536

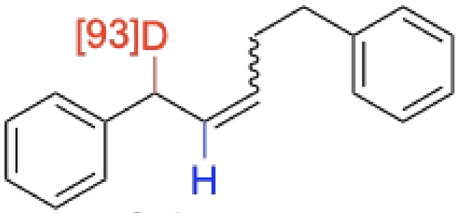

d$_1$-2g

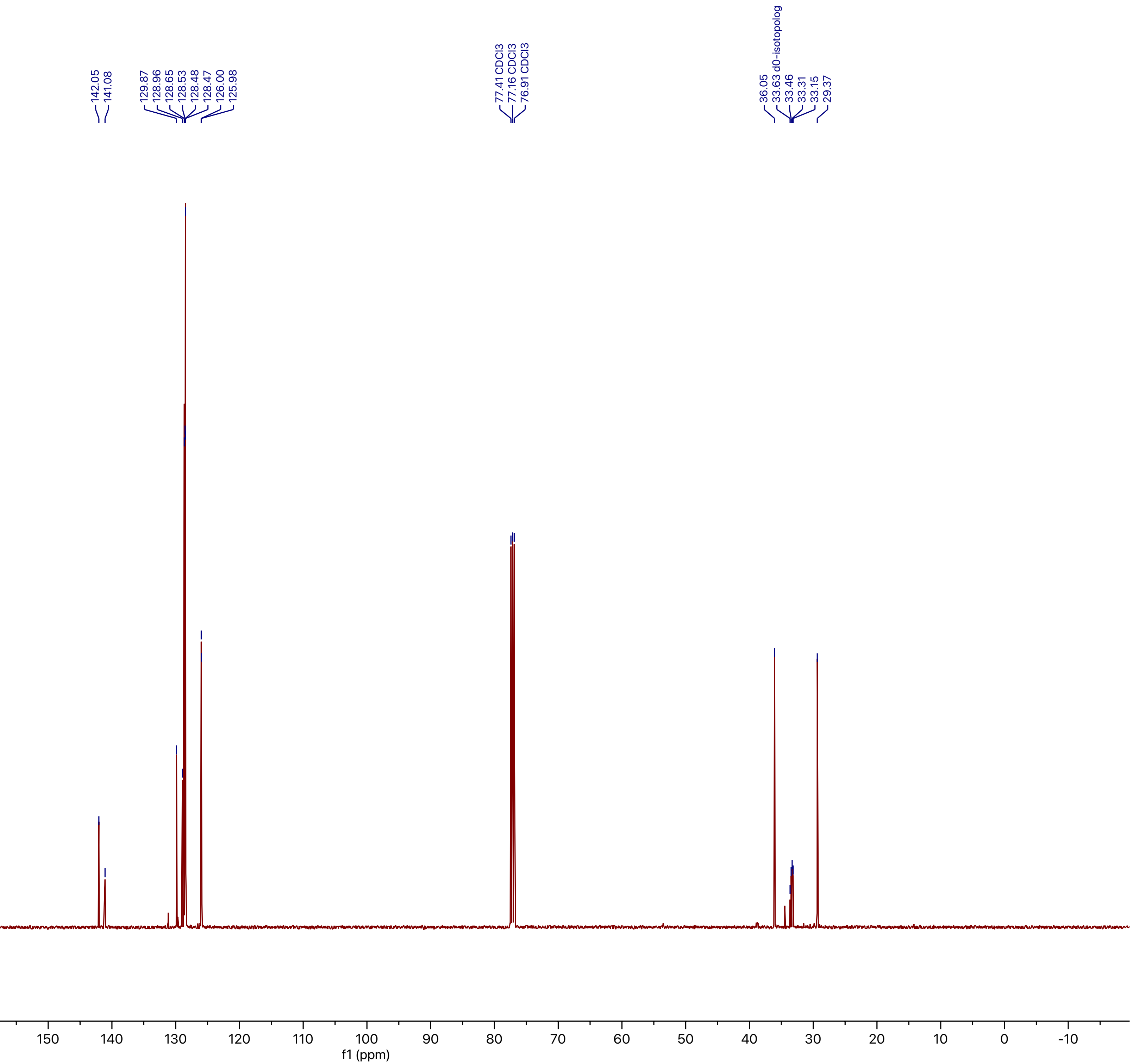

	Parameter	Value
1	Origin	Varian
2	Solvent	cdcl3
3	Temperature	25.0
4	Pulse Sequence	s2pul
5	Experiment	1D
6	Probe	ASWPFG8319
7	Number of Scans	16
8	Receiver Gain	50
9	Relaxation Delay	10.0000
10	Pulse Width	7.7500
11	Spectrometer Frequency	399.73
12	Spectral Width	6410.3
13	Lowest Frequency	-807.2
14	Nucleus	1H
15	Acquired Size	16384
16	Spectral Size	65536
17	Digital Resolution	0.10

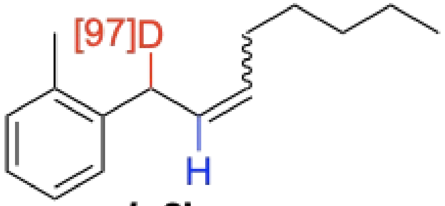

d$_1$-2h

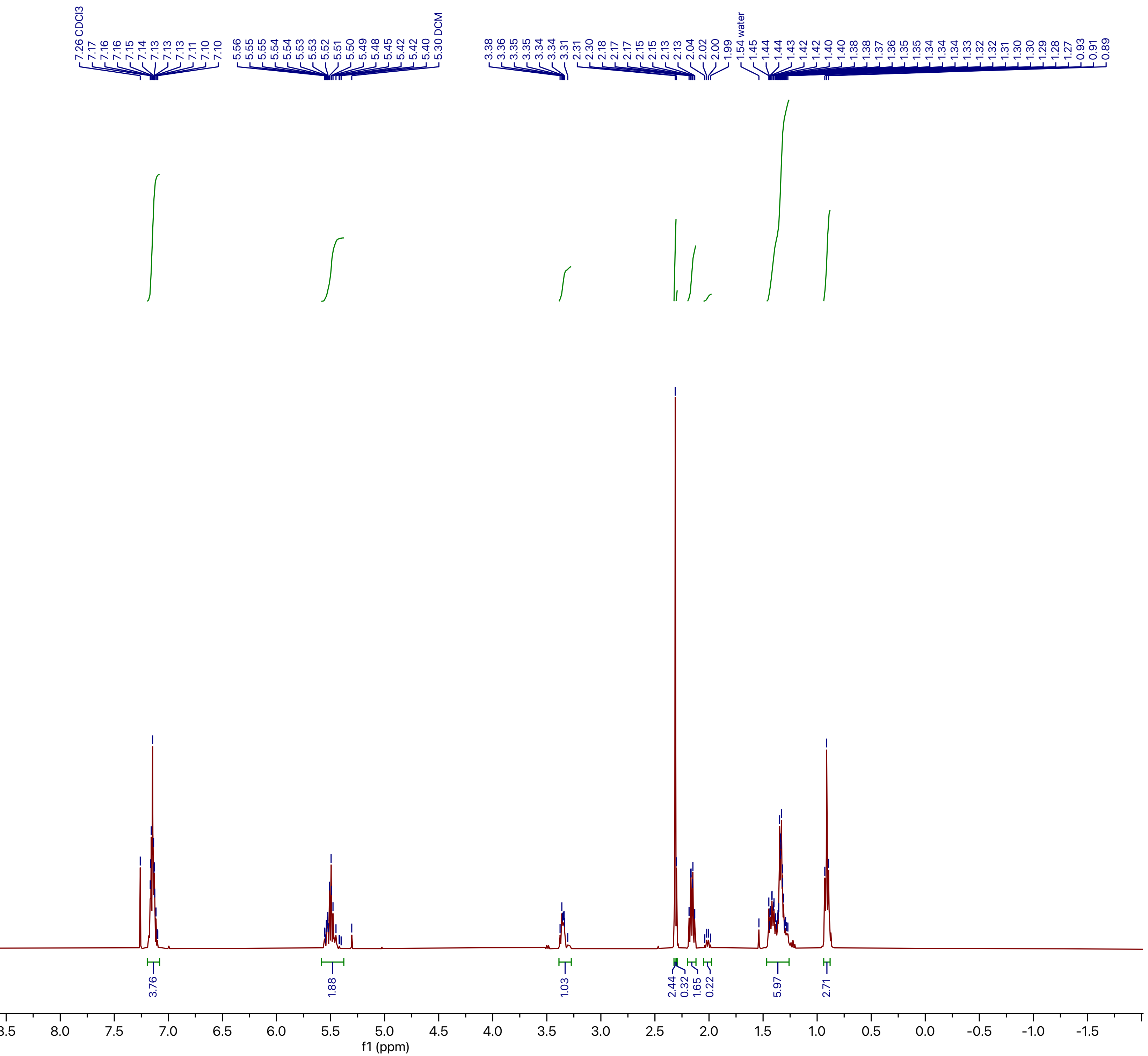

	Parameter	Value
1	Origin	Varian
2	Solvent	cdcl3
3	Temperature	25.0
4	Pulse Sequence	s2pul
5	Experiment	1D
6	Probe	ASWPFG8319
7	Number of Scans	128
8	Receiver Gain	30
9	Relaxation Delay	0.5000
10	Pulse Width	300.0000
11	Spectrometer Frequency	61.36
12	Spectral Width	552.1
13	Lowest Frequency	0.1
14	Nucleus	lk
15	Acquired Size	614
16	Spectral Size	2048

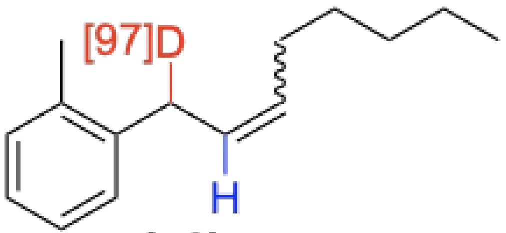

d$_1$-2h

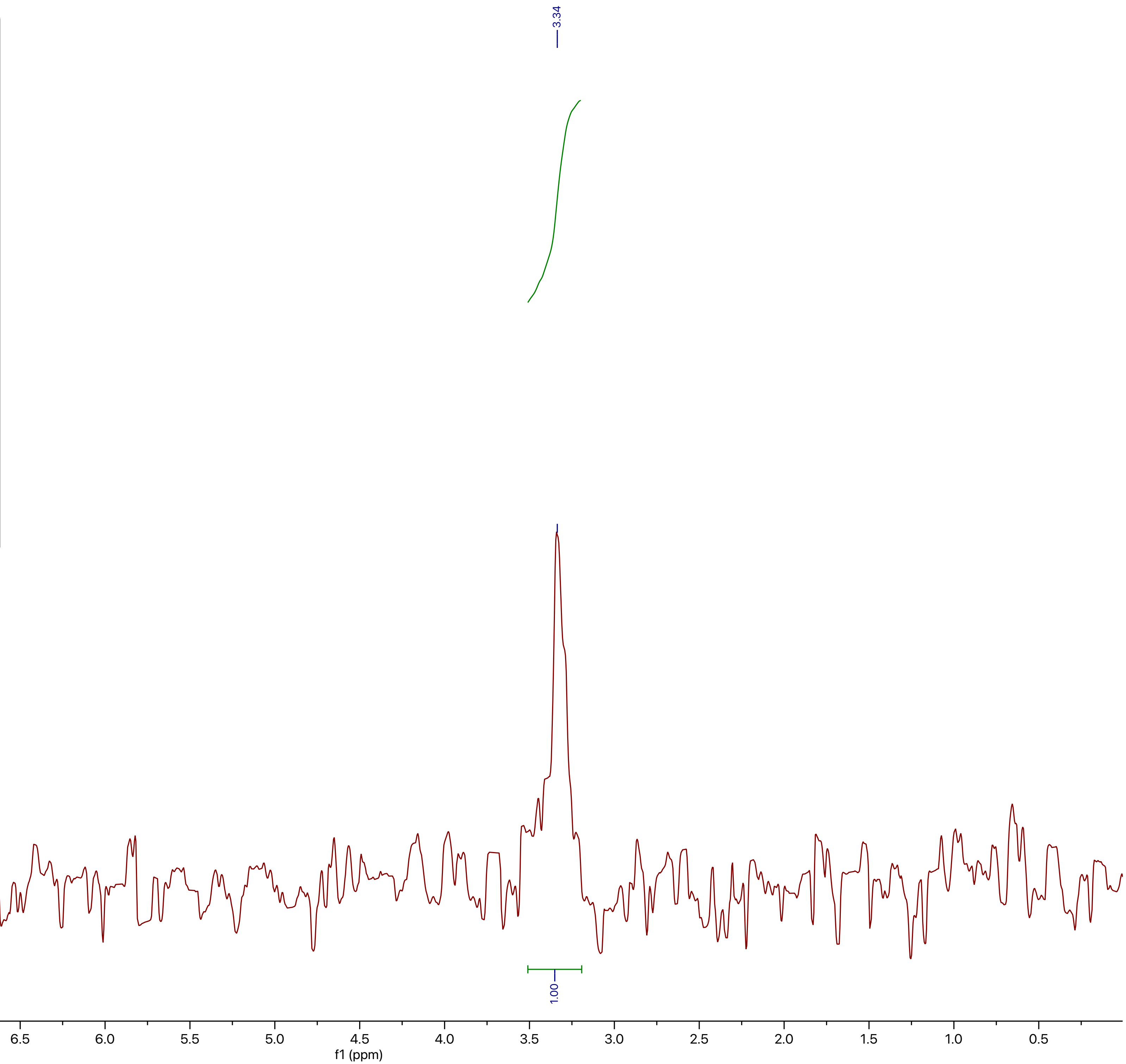

	Parameter	Value
1	Origin	Varian
2	Solvent	cdcl3
3	Temperature	25.0
4	Pulse Sequence	s2pul
5	Experiment	1D
6	Probe	ASWPFG8319
7	Number of Scans	512
8	Receiver Gain	30
9	Relaxation Delay	3.0000
10	Pulse Width	5.7500
11	Spectrometer Frequency	100.52
12	Spectral Width	25000.0
13	Lowest Frequency	-1429.9
14	Nucleus	13C
15	Acquired Size	32768
16	Spectral Size	65536
17	Digital Resolution	0.38

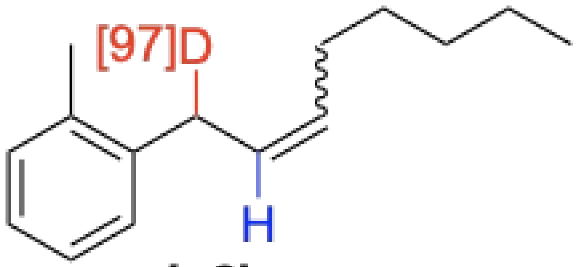

d_1-2h

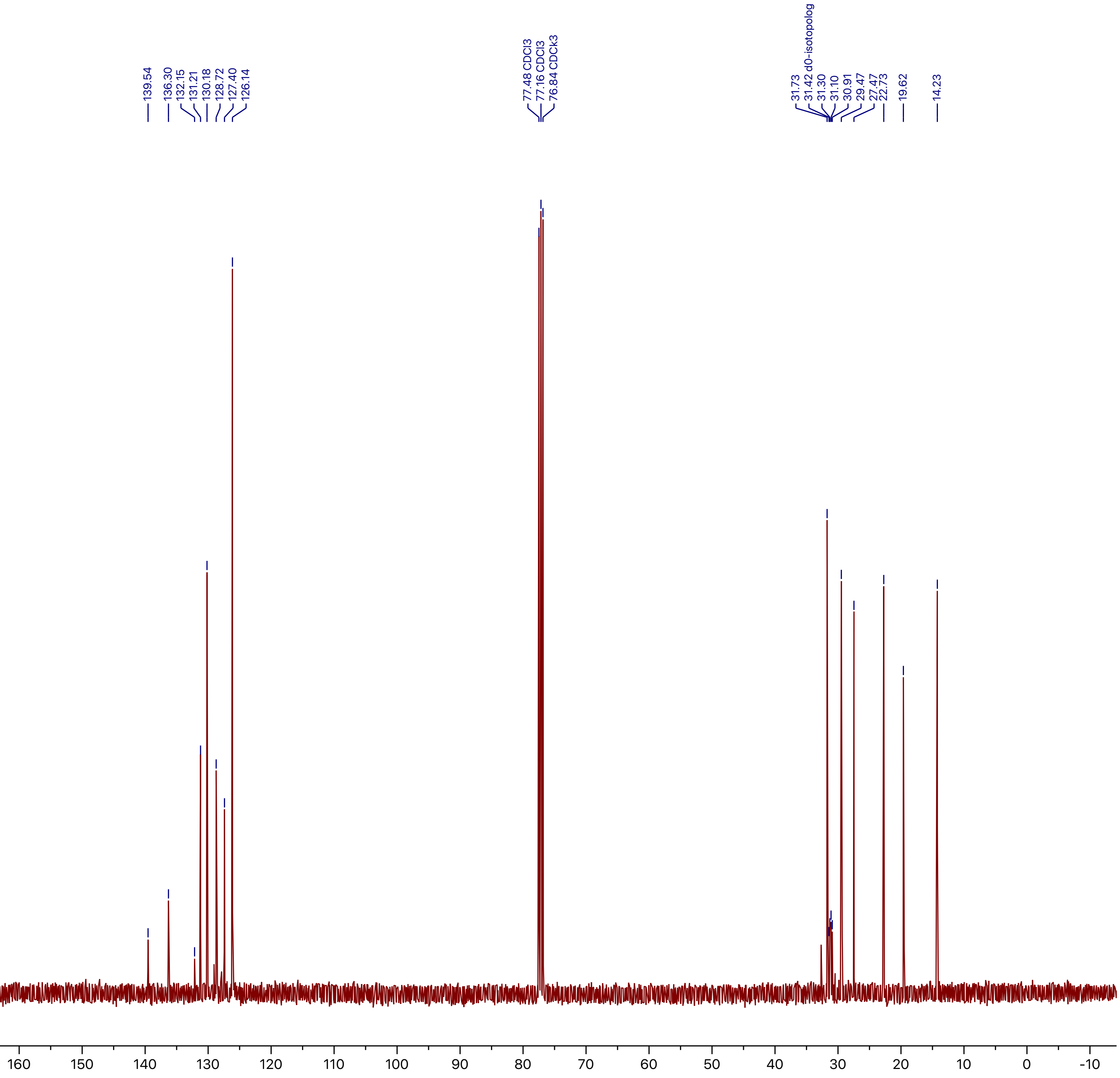

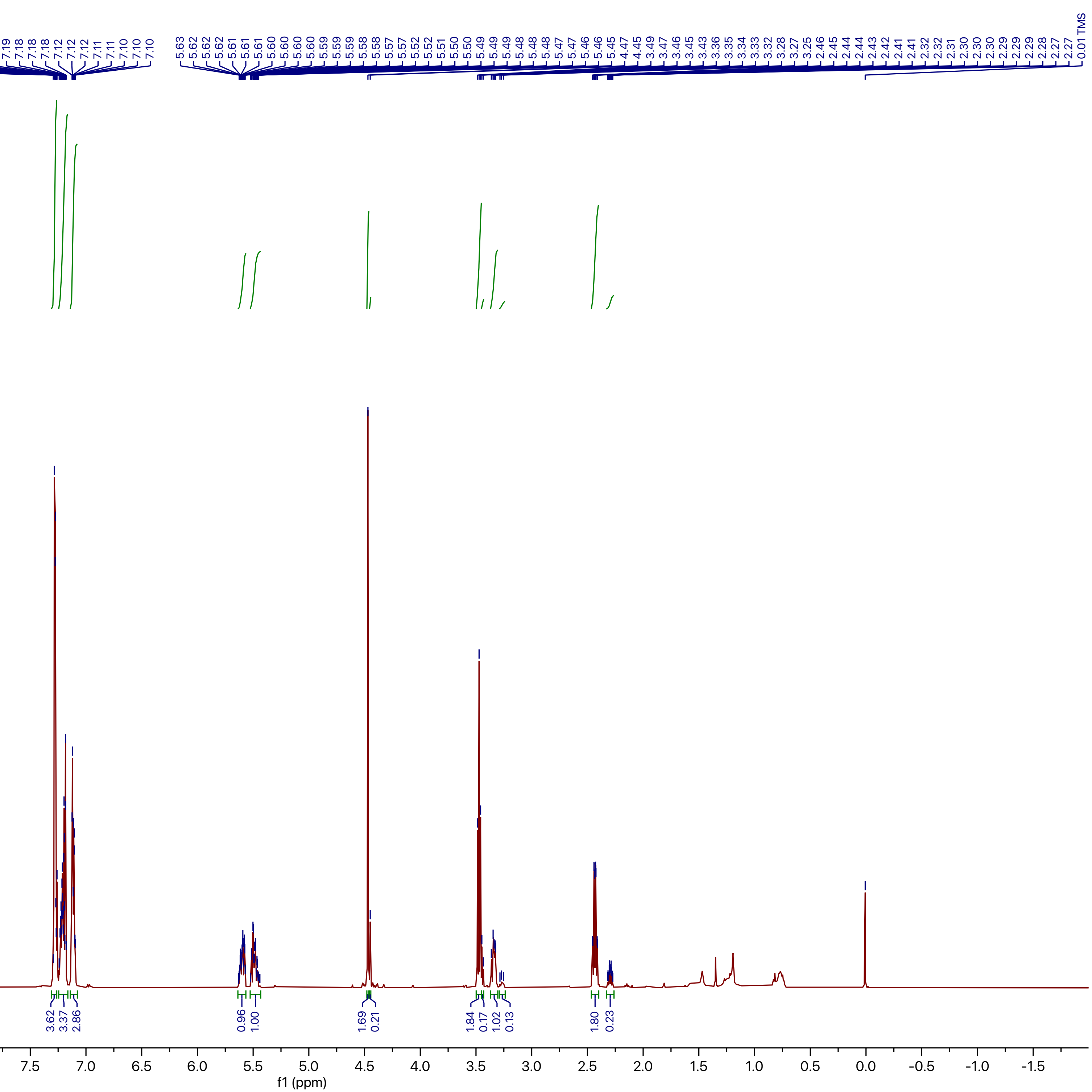

	Parameter	Value
1	Origin	Bruker BioSpin GmbH
2	Instrument	Avance
3	Solvent	CDCl3
4	Temperature	300.0
5	Pulse Sequence	zg30
6	Experiment	1D
7	Probe	Z151574_0073 (PI HR-BBO500S1-BBF/ H/ D-5.0-Z SP)
8	Number of Scans	16
9	Receiver Gain	101.0
10	Relaxation Delay	1.0000
11	Pulse Width	8.0000
12	Spectrometer Frequency	500.21
13	Spectral Width	10000.0
14	Lowest Frequency	-1960.9
15	Nucleus	1H
16	Acquired Size	32768
17	Spectral Size	65536

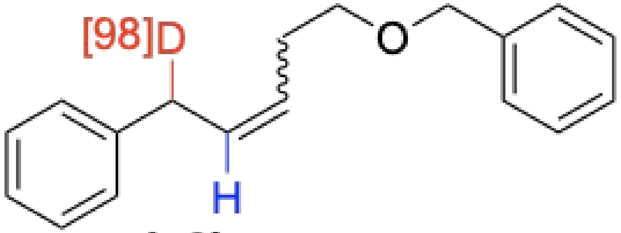

***d_1*-2i**

	Parameter	Value
1	Origin	Varian
2	Solvent	cdcl3
3	Temperature	25.0
4	Pulse Sequence	s2pul
5	Experiment	1D
6	Probe	OneNMR_W036
7	Number of Scans	32
8	Receiver Gain	20
9	Relaxation Delay	5.0000
10	Pulse Width	300.0000
11	Spectrometer Frequency	76.71
12	Spectral Width	1535.6
13	Lowest Frequency	-381.4
14	Nucleus	lk
15	Acquired Size	2048
16	Spectral Size	4096
17	Digital Resolution	0.37

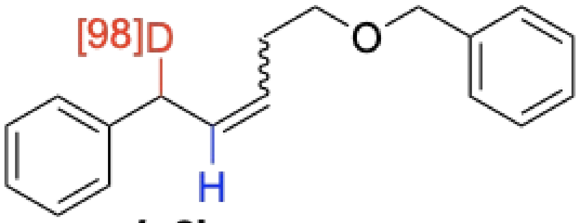

***d_1*-2i**

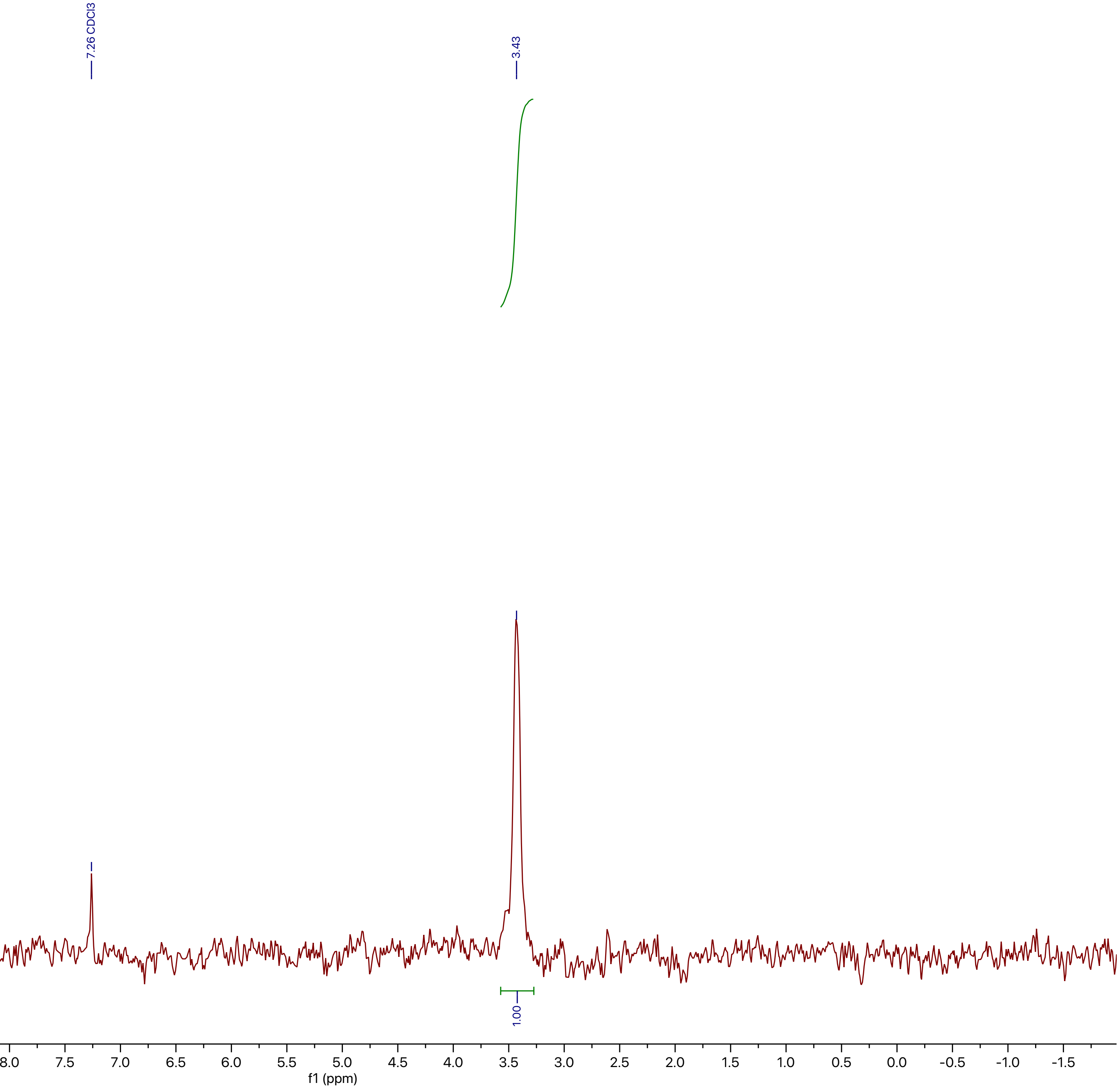

	Parameter	Value
1	Origin	Bruker BioSpin GmbH
2	Instrument	Avance
3	Solvent	CDCl3
4	Temperature	300.0
5	Pulse Sequence	zgpg30
6	Experiment	1D
7	Probe	Z151574_0073 (PI HR-BBO500S1-BBF/ H/ D-5.0-Z SP)
8	Number of Scans	100
9	Receiver Gain	101.0
10	Relaxation Delay	2.0000
11	Pulse Width	9.0000
12	Spectrometer Frequency	125.79
13	Spectral Width	30120.5
14	Lowest Frequency	-2465.7
15	Nucleus	13C
16	Acquired Size	32768
17	Spectral Size	65536

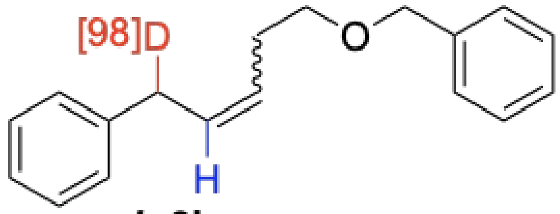

d_1-2i

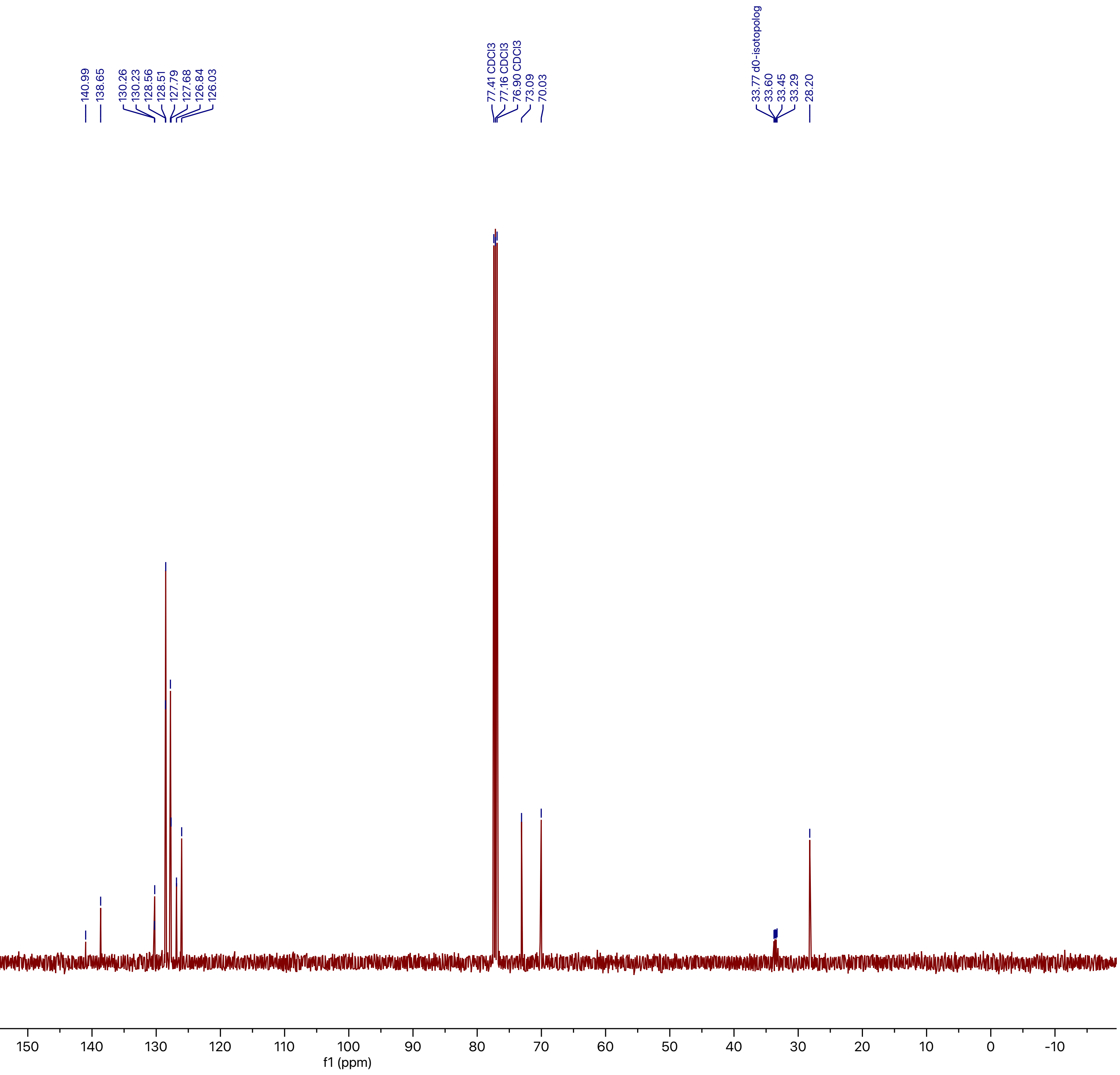

	Parameter	Value
1	Origin	Varian
2	Solvent	cdcl3
3	Temperature	0.0
4	Pulse Sequence	s2pul
5	Experiment	1D
6	Probe	ASWPFG8319
7	Number of Scans	16
8	Receiver Gain	52
9	Relaxation Delay	10.0000
10	Pulse Width	7.7500
11	Spectrometer Frequency	399.73
12	Spectral Width	6410.3
13	Lowest Frequency	-805.2
14	Nucleus	1H
15	Acquired Size	16384
16	Spectral Size	65536
17	Digital Resolution	0.10

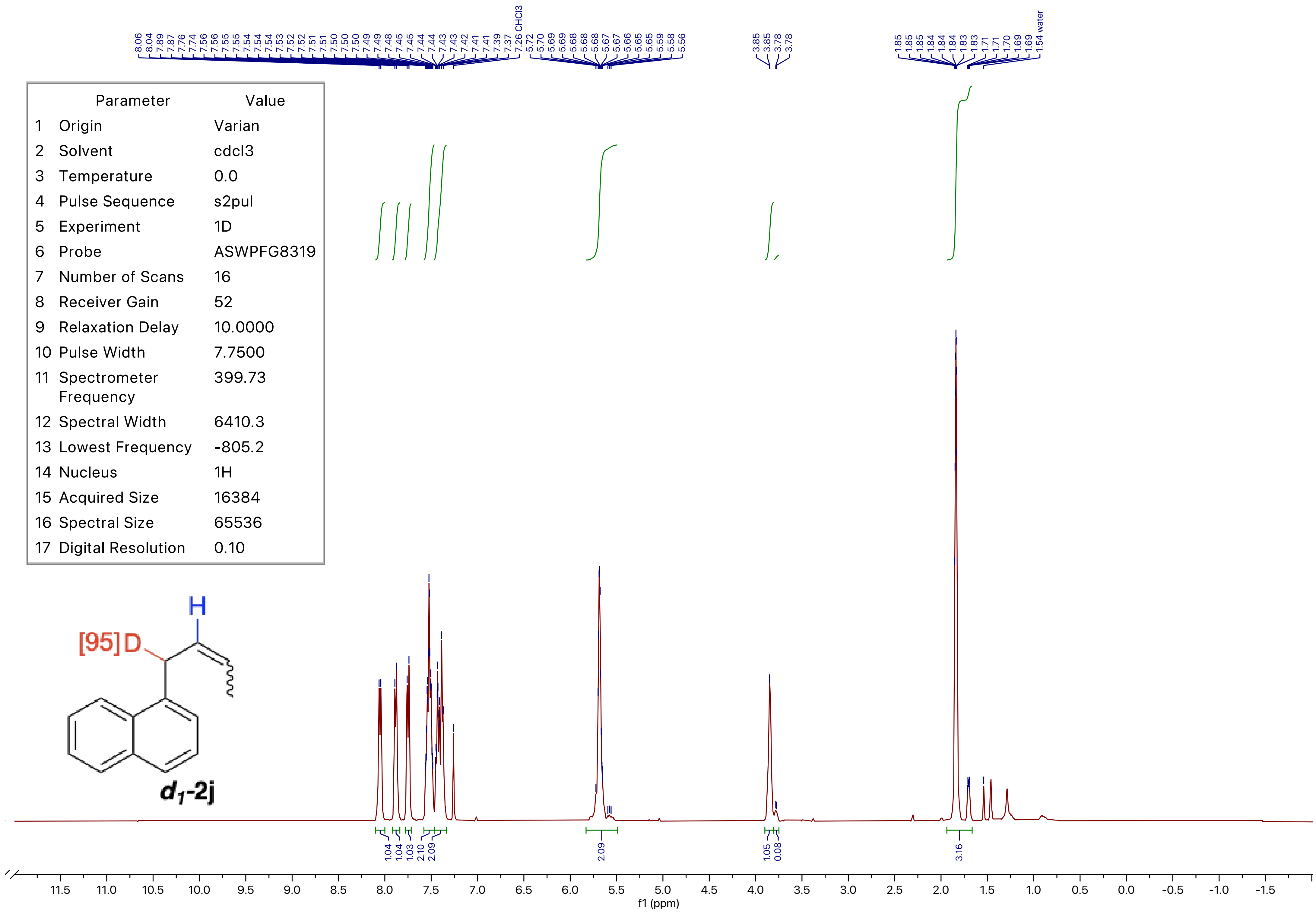

Parameter		Value
1	Origin	Varian
2	Solvent	cdcl3
3	Temperature	0.0
4	Pulse Sequence	s2pul
5	Experiment	1D
6	Probe	ASWPFG8319
7	Number of Scans	128
8	Receiver Gain	30
9	Relaxation Delay	0.5000
10	Pulse Width	300.0000
11	Spectrometer Frequency	61.36
12	Spectral Width	552.1
13	Lowest Frequency	-13.1
14	Nucleus	lk
15	Acquired Size	614
16	Spectral Size	2048
17	Digital Resolution	0.27

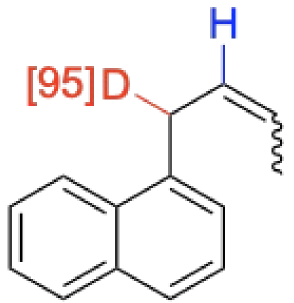

d_1-2j

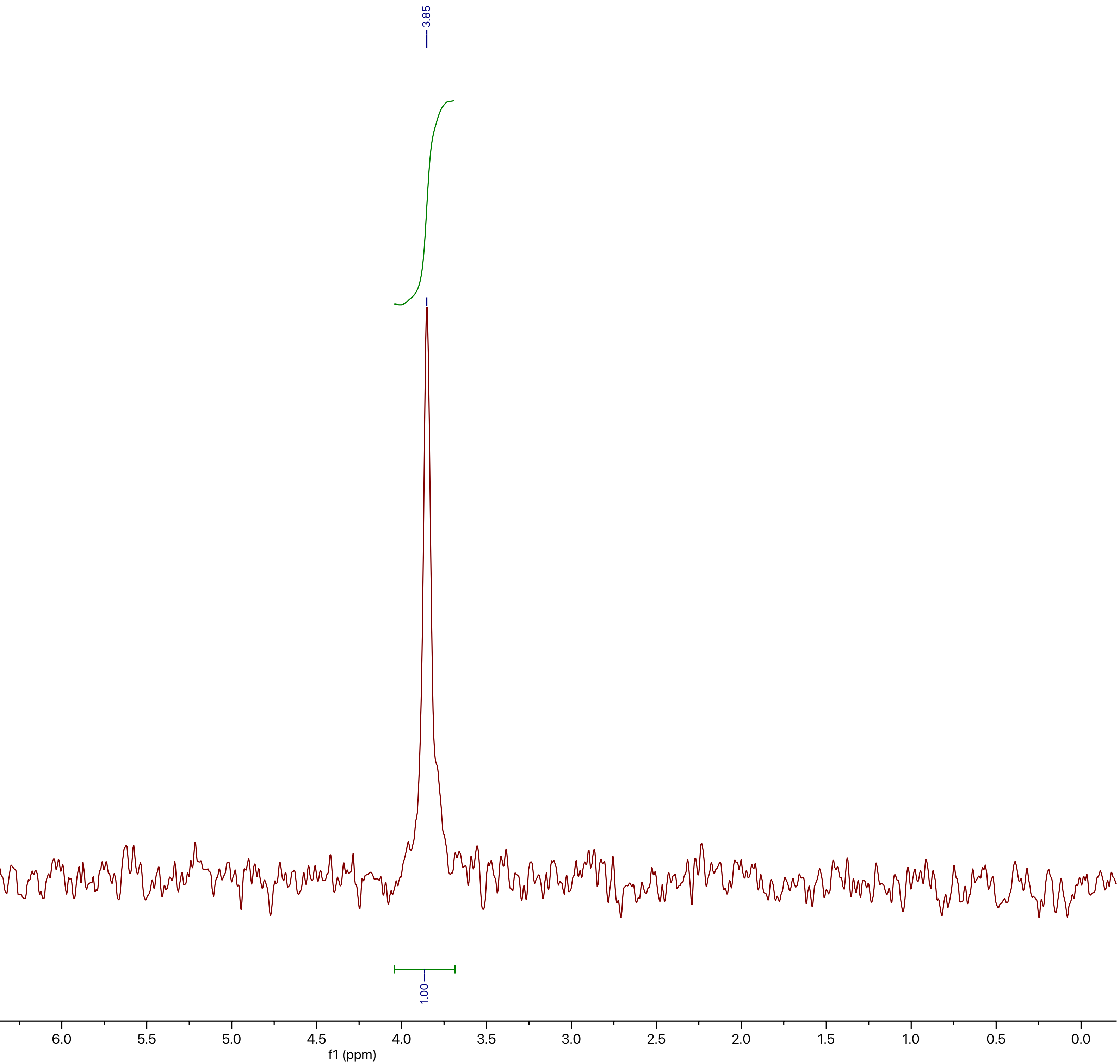

	Parameter	Value
1	Origin	Varian
2	Solvent	cdcl3
3	Temperature	0.0
4	Pulse Sequence	s2pul
5	Experiment	1D
6	Probe	ASWPFG8319
7	Number of Scans	512
8	Receiver Gain	30
9	Relaxation Delay	3.0000
10	Pulse Width	5.7500
11	Spectrometer Frequency	100.52
12	Spectral Width	25000.0
13	Lowest Frequency	-1431.8
14	Nucleus	13C
15	Acquired Size	32768
16	Spectral Size	65536
17	Digital Resolution	0.38

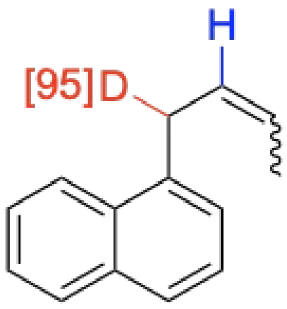

d$_1$-2j

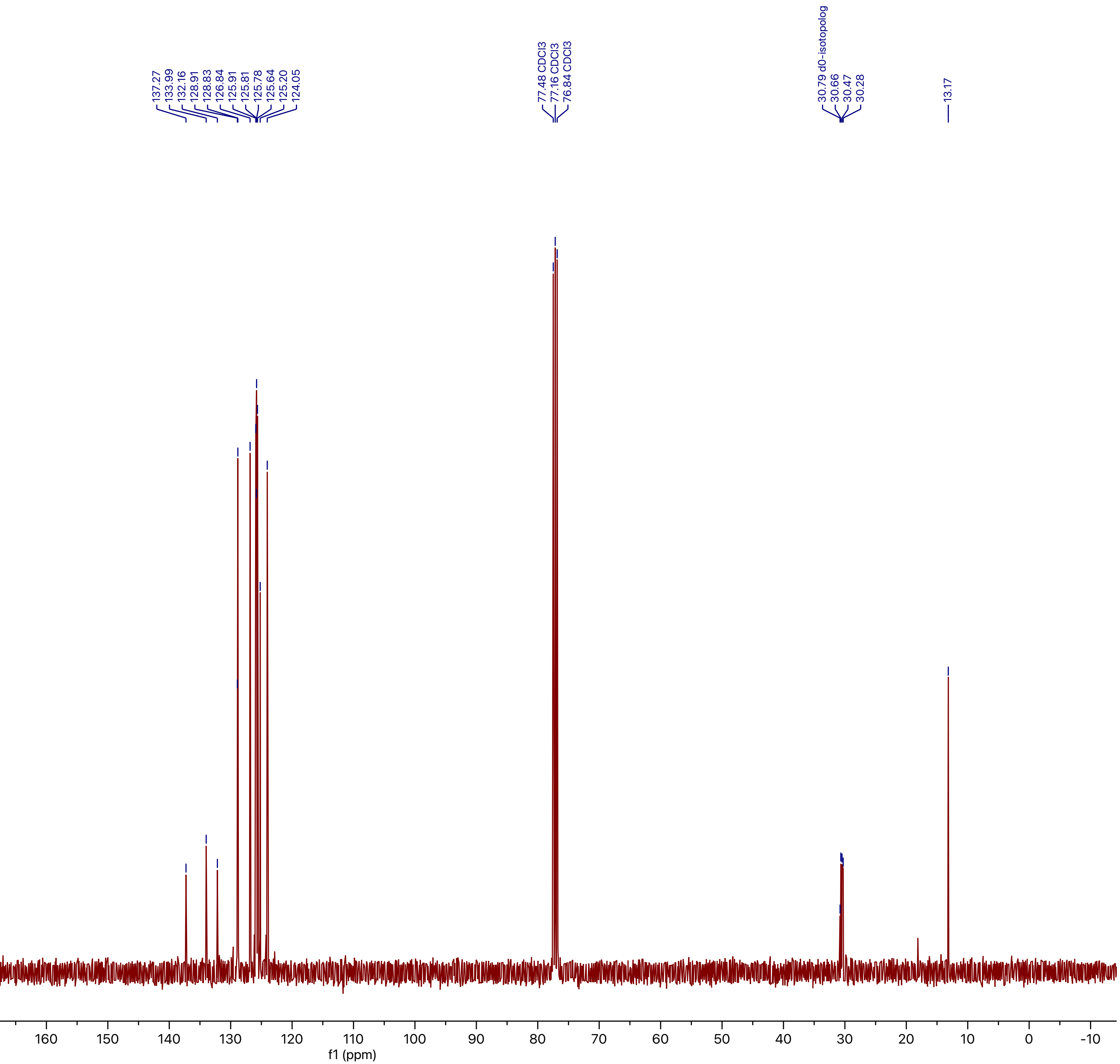

	Parameter	Value
1	Origin	Varian
2	Solvent	cdcl3
3	Temperature	25.0
4	Pulse Sequence	s2pul
5	Experiment	1D
6	Probe	ASWPFG8319
7	Number of Scans	16
8	Receiver Gain	48
9	Relaxation Delay	10.0000
10	Pulse Width	7.7500
11	Spectrometer Frequency	399.73
12	Spectral Width	6410.3
13	Lowest Frequency	-807.5
14	Nucleus	1H
15	Acquired Size	16384
16	Spectral Size	65536
17	Digital Resolution	0.10

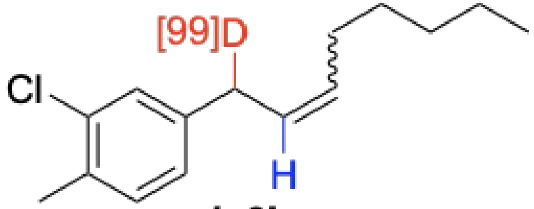

***d*$_1$-2k**

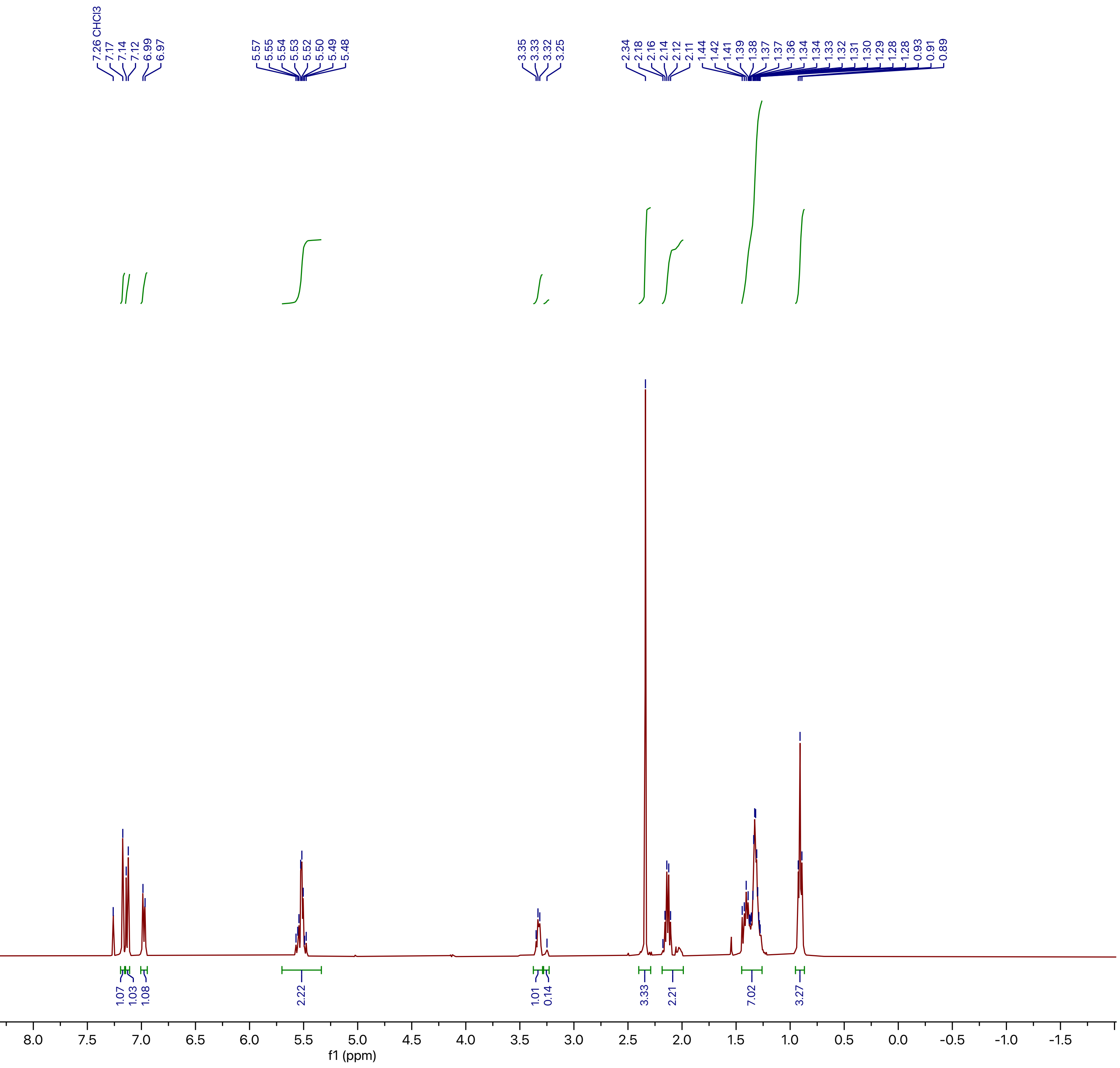

	Parameter	Value
1	Origin	Varian
2	Solvent	cdcl3
3	Temperature	25.0
4	Pulse Sequence	s2pul
5	Experiment	1D
6	Probe	ASWPFG8319
7	Number of Scans	128
8	Receiver Gain	30
9	Relaxation Delay	0.5000
10	Pulse Width	300.0000
11	Spectrometer Frequency	61.36
12	Spectral Width	552.1
13	Lowest Frequency	19.1
14	Nucleus	lk
15	Acquired Size	614
16	Spectral Size	2048
17	Digital Resolution	0.27

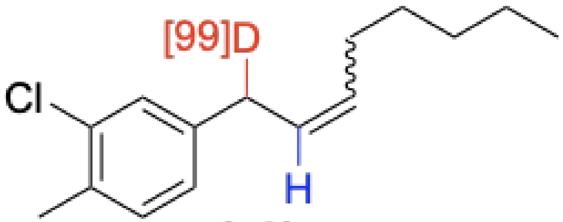

d_1-2k

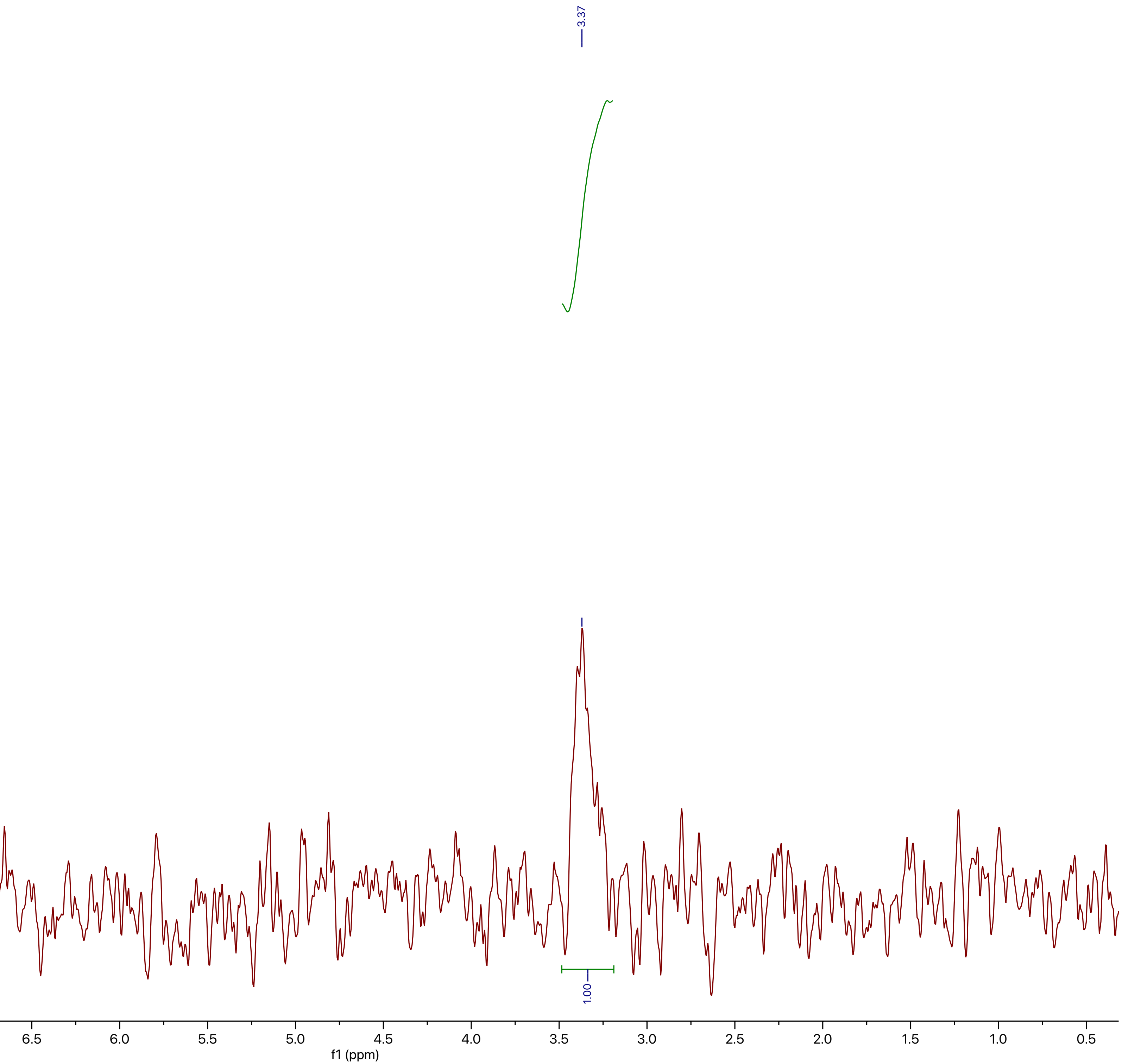

Parameter		Value
1	Origin	Varian
2	Solvent	cdcl3
3	Temperature	25.0
4	Pulse Sequence	s2pul
5	Experiment	1D
6	Probe	ASWPFG8319
7	Number of Scans	512
8	Receiver Gain	30
9	Relaxation Delay	2.0000
10	Pulse Width	5.7500
11	Spectrometer Frequency	100.52
12	Spectral Width	25000.0
13	Lowest Frequency	-1429.6
14	Nucleus	13C
15	Acquired Size	32768
16	Spectral Size	65536
17	Digital Resolution	0.38

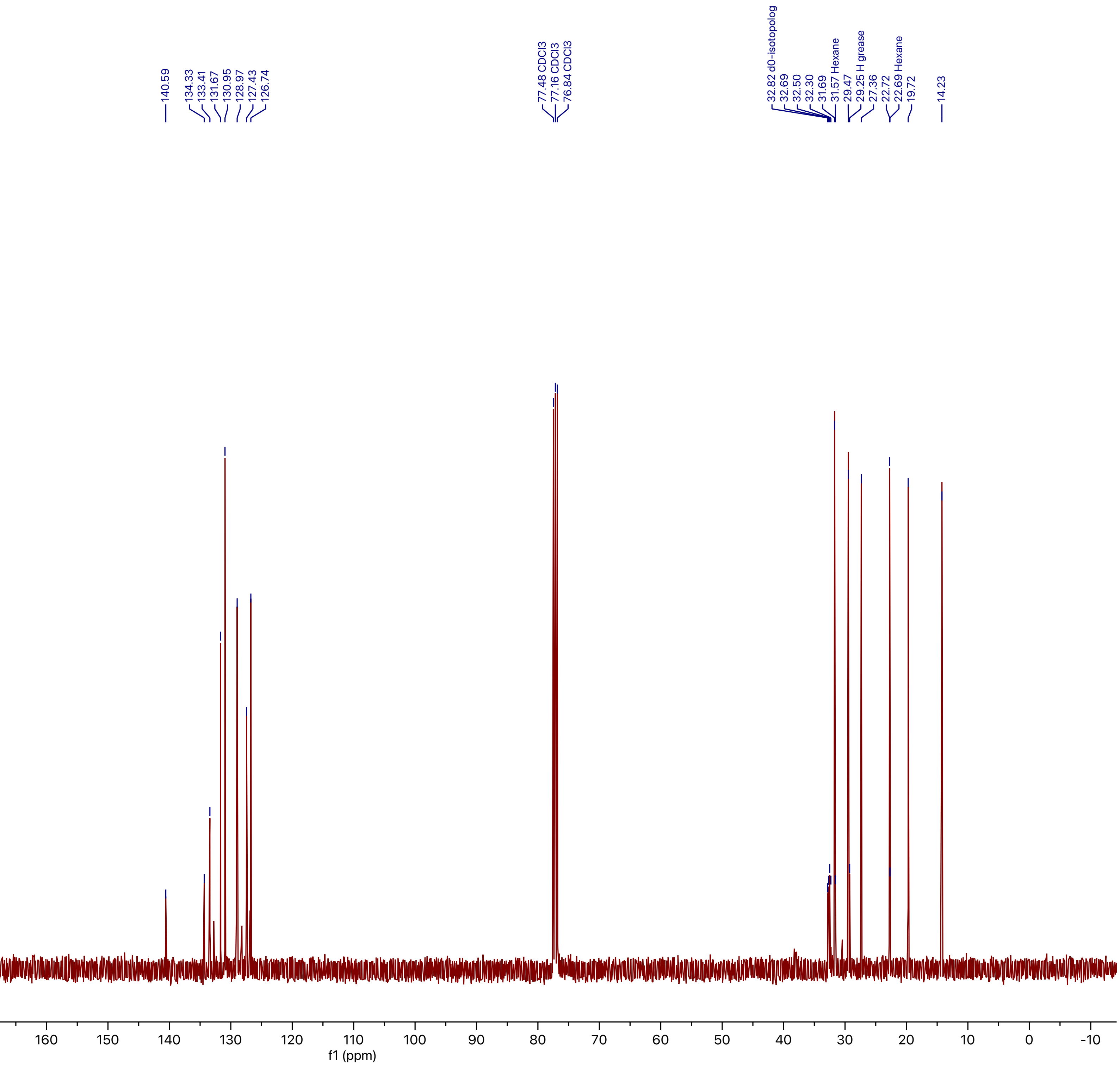

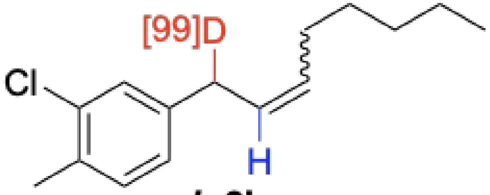

d_1-2k

Parameter	Value
1 Origin	Varian
2 Solvent	cdcl3
3 Temperature	0.0
4 Pulse Sequence	s2pul
5 Experiment	1D
6 Probe	ASWPFG8319
7 Number of Scans	32
8 Receiver Gain	50
9 Relaxation Delay	10.0000
10 Pulse Width	6.4000
11 Spectrometer Frequency	399.73
12 Spectral Width	6410.3
13 Lowest Frequency	-806.3
14 Nucleus	1H
15 Acquired Size	16384
16 Spectral Size	65536
17 Digital Resolution	0.10

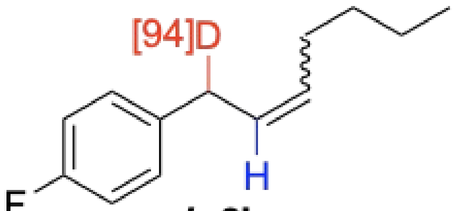

d_1-2l

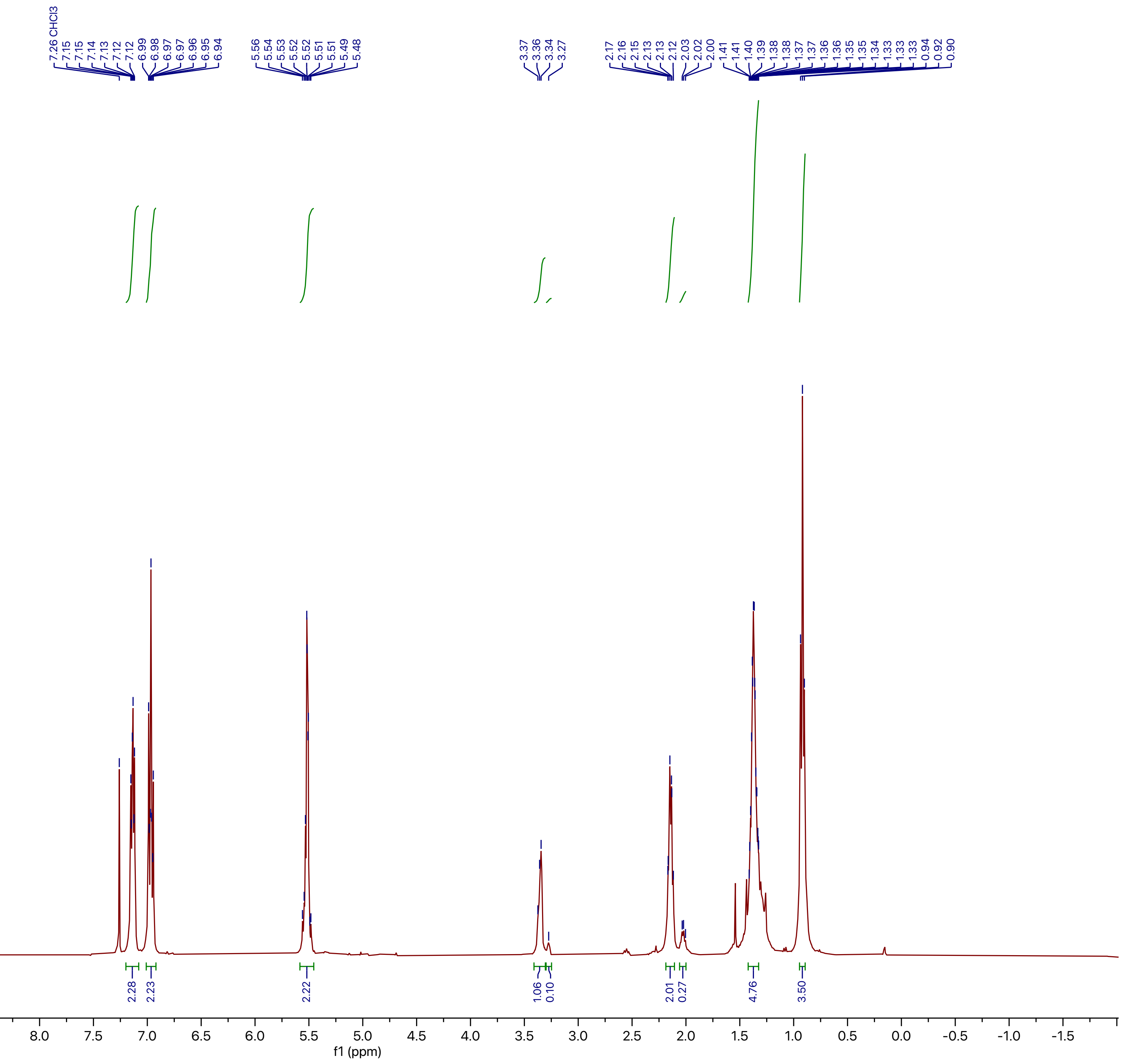

	Parameter	Value
1	Origin	Varian
2	Solvent	cdcl3
3	Temperature	0.0
4	Pulse Sequence	s2pul
5	Experiment	1D
6	Probe	ASWPFG8319
7	Number of Scans	128
8	Receiver Gain	30
9	Relaxation Delay	0.5000
10	Pulse Width	300.0000
11	Spectrometer Frequency	61.36
12	Spectral Width	552.1
13	Lowest Frequency	0.1
14	Nucleus	lk
15	Acquired Size	614
16	Spectral Size	2048
17	Digital Resolution	0.27

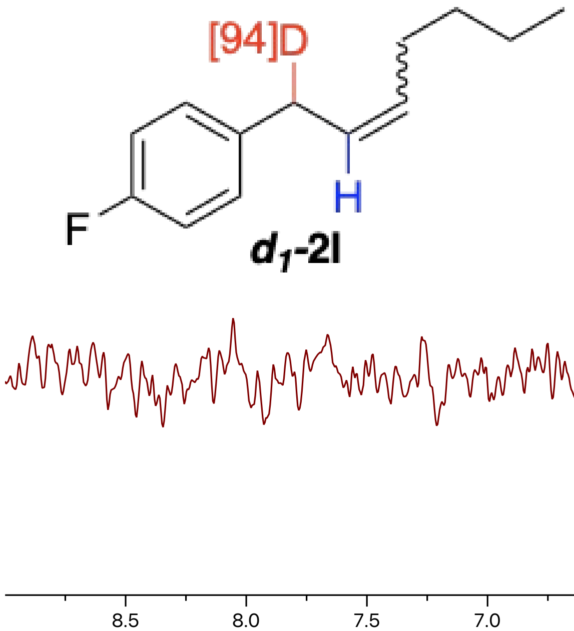

d_1-2l

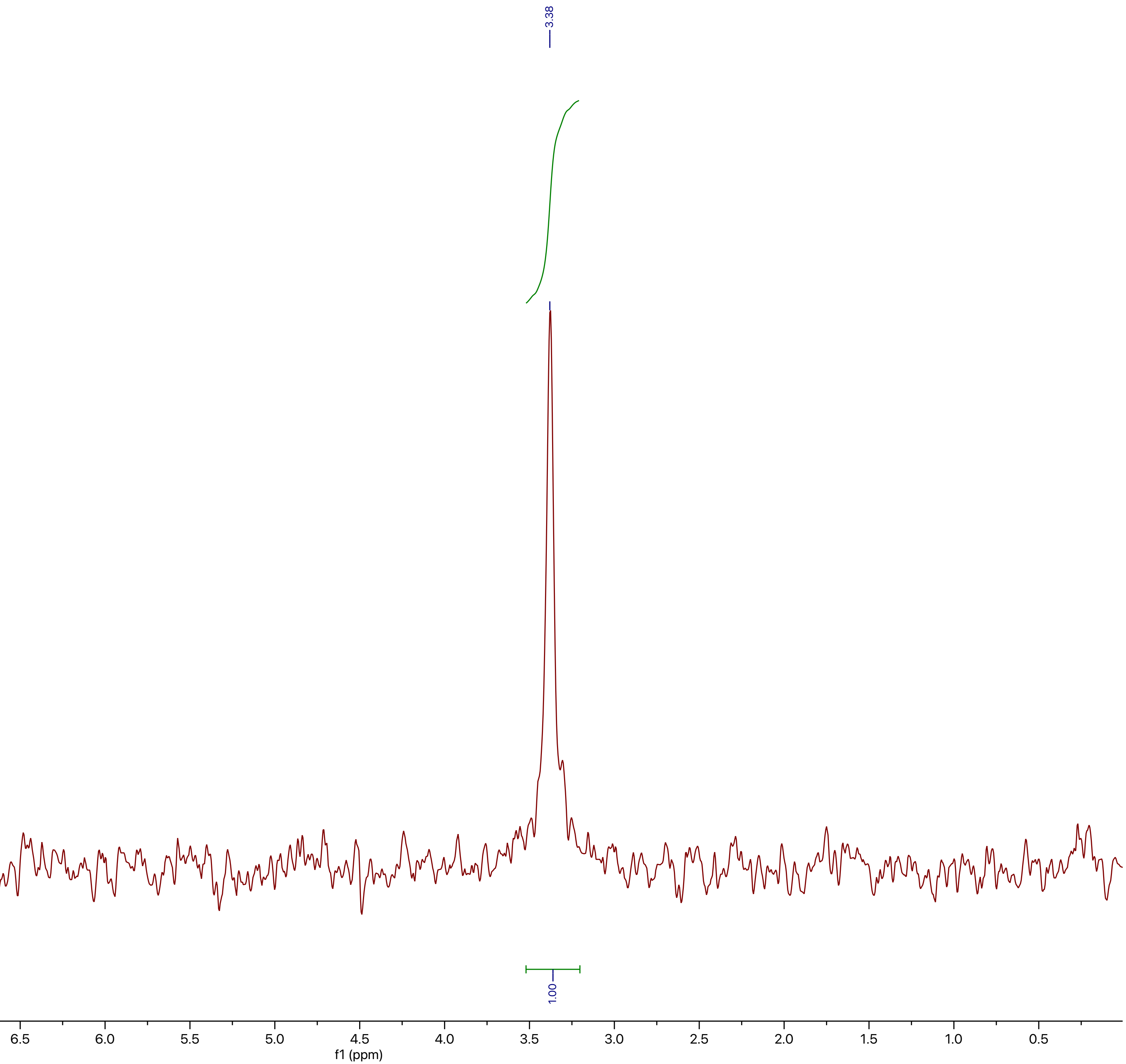

	Parameter	Value
1	Origin	Varian
2	Solvent	cdcl3
3	Temperature	0.0
4	Pulse Sequence	s2pul
5	Experiment	1D
6	Probe	ASWPFG8319
7	Number of Scans	128
8	Receiver Gain	30
9	Relaxation Delay	30.0000
10	Pulse Width	4.5625
11	Spectrometer Frequency	100.52
12	Spectral Width	25000.0
13	Lowest Frequency	-1429.9
14	Nucleus	13C
15	Acquired Size	32768
16	Spectral Size	65536
17	Digital Resolution	0.38

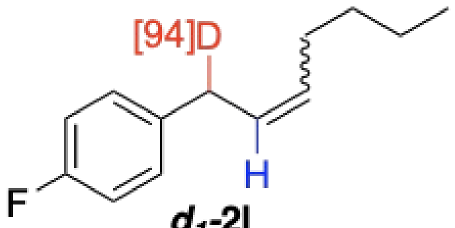

***d_1*-2l**

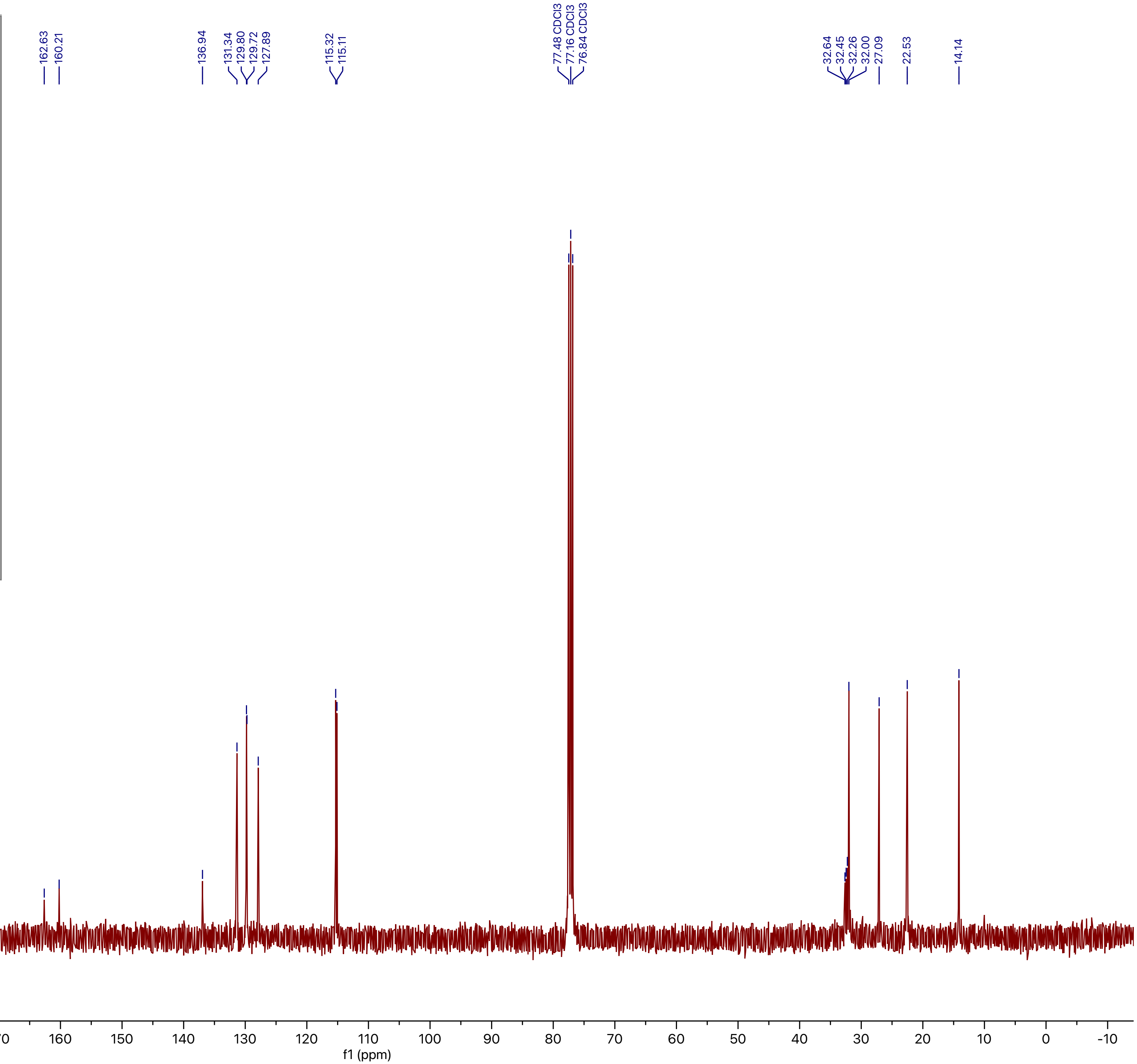

	Parameter	Value
1	Origin	Varian
2	Solvent	cdcl3
3	Temperature	0.0
4	Pulse Sequence	s2pul
5	Experiment	1D
6	Probe	ASWPFG8319
7	Number of Scans	16
8	Receiver Gain	60
9	Relaxation Delay	1.0000
10	Pulse Width	5.0000
11	Spectrometer Frequency	376.09
12	Spectral Width	89285.7
13	Lowest Frequency	-76613.1
14	Nucleus	19F
15	Acquired Size	65536
16	Spectral Size	131072
17	Digital Resolution	0.68

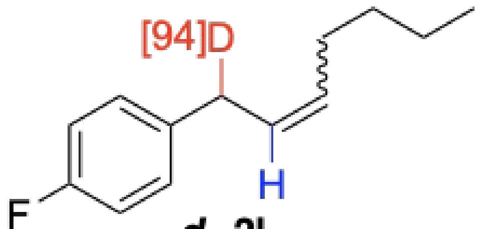

d_1-2l

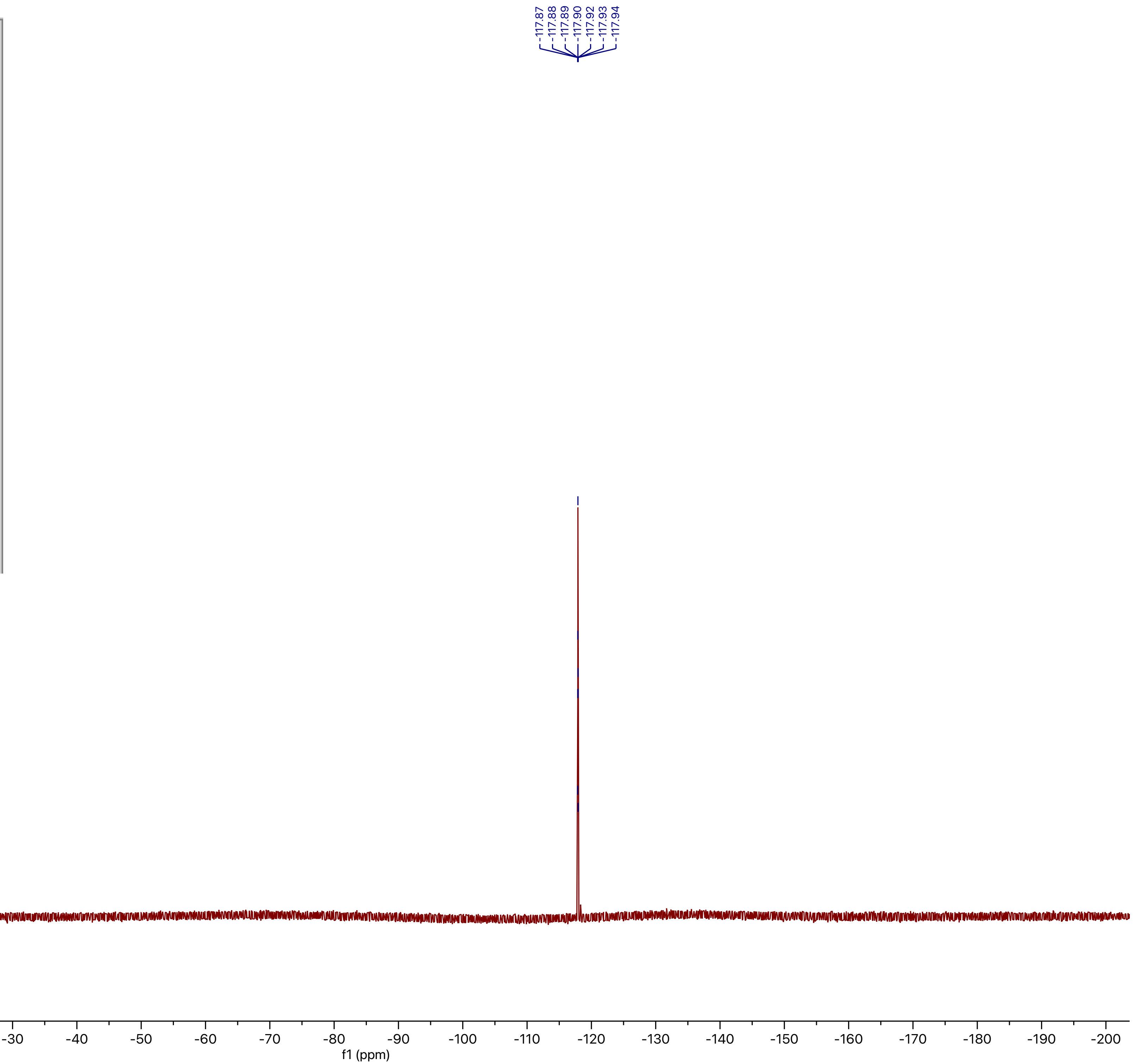

	Parameter	Value
1	Origin	Bruker BioSpin GmbH
2	Instrument	Avance
3	Solvent	CDCl3
4	Temperature	300.0
5	Pulse Sequence	zg30
6	Experiment	1D
7	Probe	Z151574_0073 (PI HR-BBO500S1-BBF/ H/ D-5.0-Z SP)
8	Number of Scans	16
9	Receiver Gain	48.4
10	Relaxation Delay	10.0000
11	Pulse Width	8.0000
12	Spectrometer Frequency	500.21
13	Spectral Width	10000.0
14	Lowest Frequency	-1922.5
15	Nucleus	1H
16	Acquired Size	32768
17	Spectral Size	65536

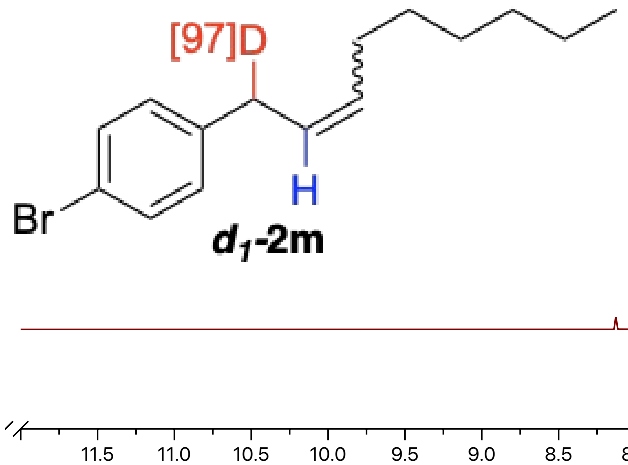

***d*₁-2m**

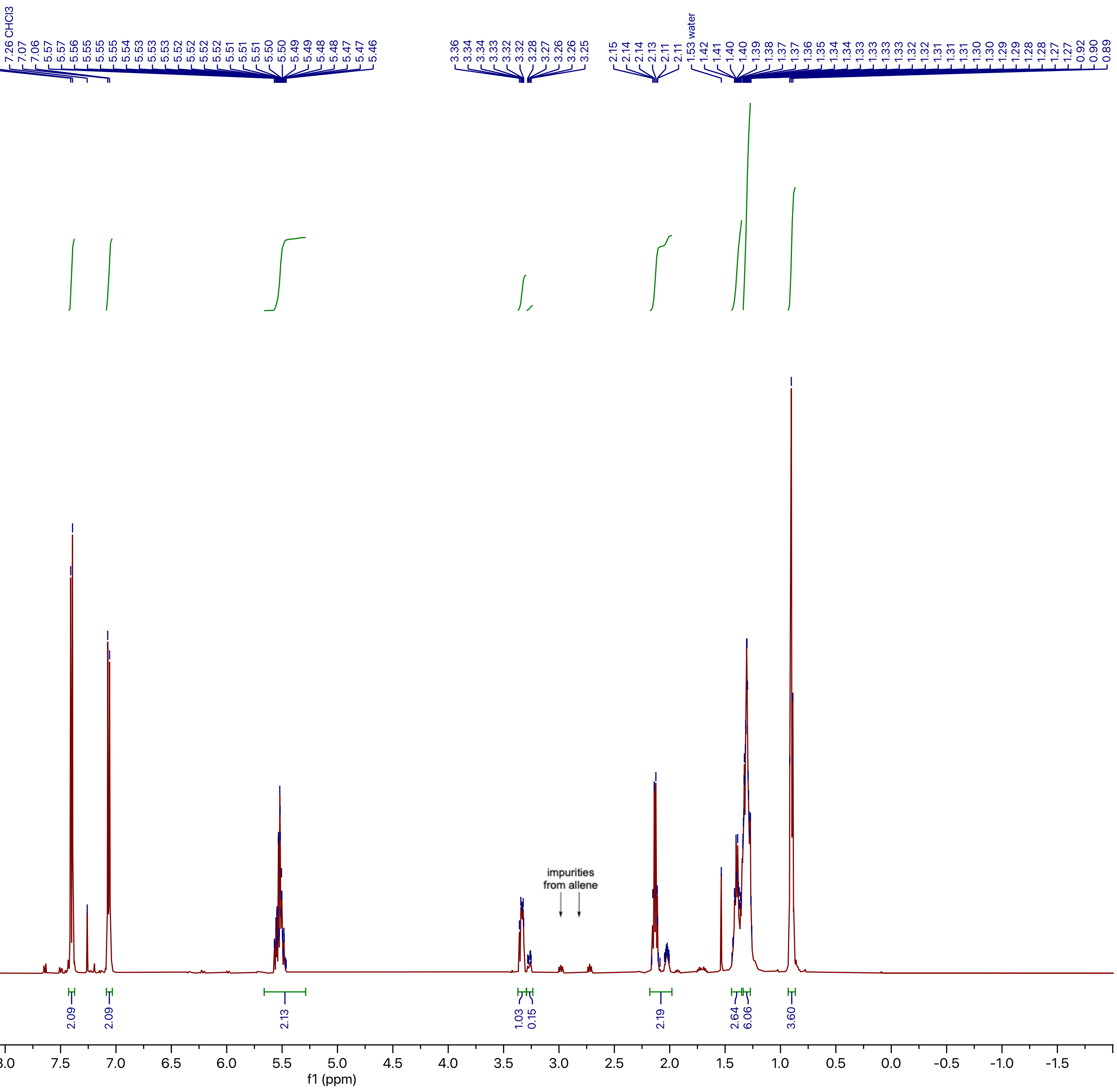

	Parameter	Value
1	Origin	Varian
2	Solvent	cdcl3
3	Temperature	25.0
4	Pulse Sequence	s2pul
5	Experiment	1D
6	Probe	OneNMR_W036
7	Number of Scans	32
8	Receiver Gain	20
9	Relaxation Delay	5.0000
10	Pulse Width	300.0000
11	Spectrometer Frequency	76.71
12	Spectral Width	1535.6
13	Lowest Frequency	-379.5
14	Nucleus	lk
15	Acquired Size	2048
16	Spectral Size	4096
17	Digital Resolution	0.37

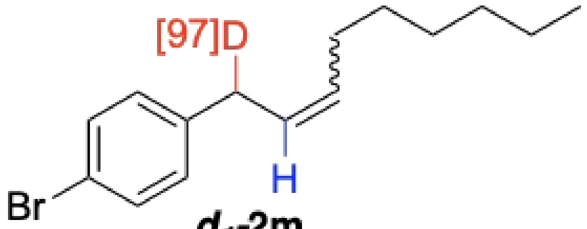

d_1-2m

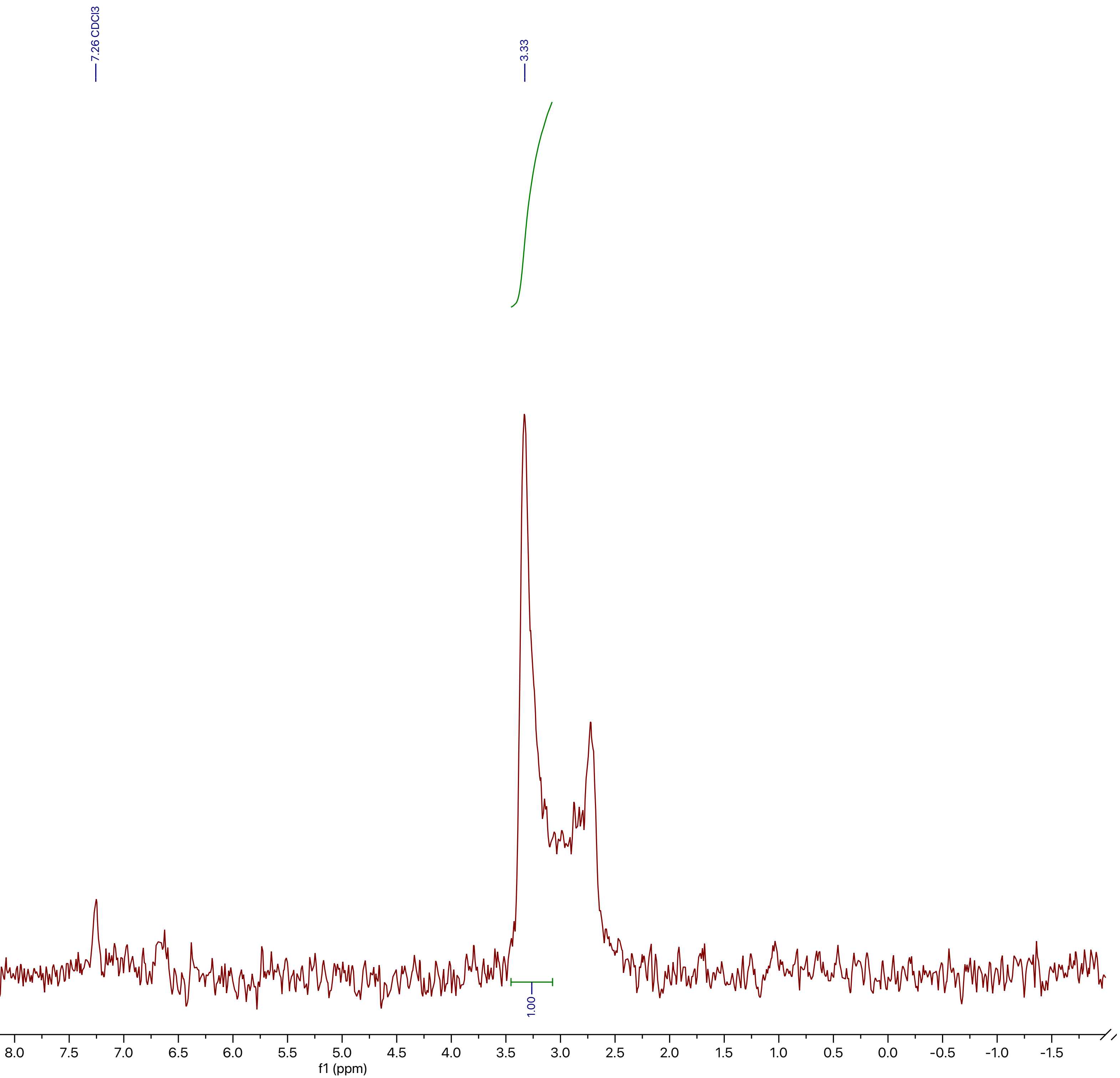

	Parameter	Value
1	Origin	Bruker BioSpin GmbH
2	Instrument	Avance
3	Solvent	CDCl3
4	Temperature	300.0
5	Pulse Sequence	zgpg30
6	Experiment	1D
7	Probe	Z151574_0073 (PI HR-BBO500S1-BBF/ H/ D-5.0-Z SP)
8	Number of Scans	1500
9	Receiver Gain	101.0
10	Relaxation Delay	2.0000
11	Pulse Width	9.0000
12	Spectrometer Frequency	125.79
13	Spectral Width	30120.5
14	Lowest Frequency	-2482.4
15	Nucleus	13C
16	Acquired Size	32768

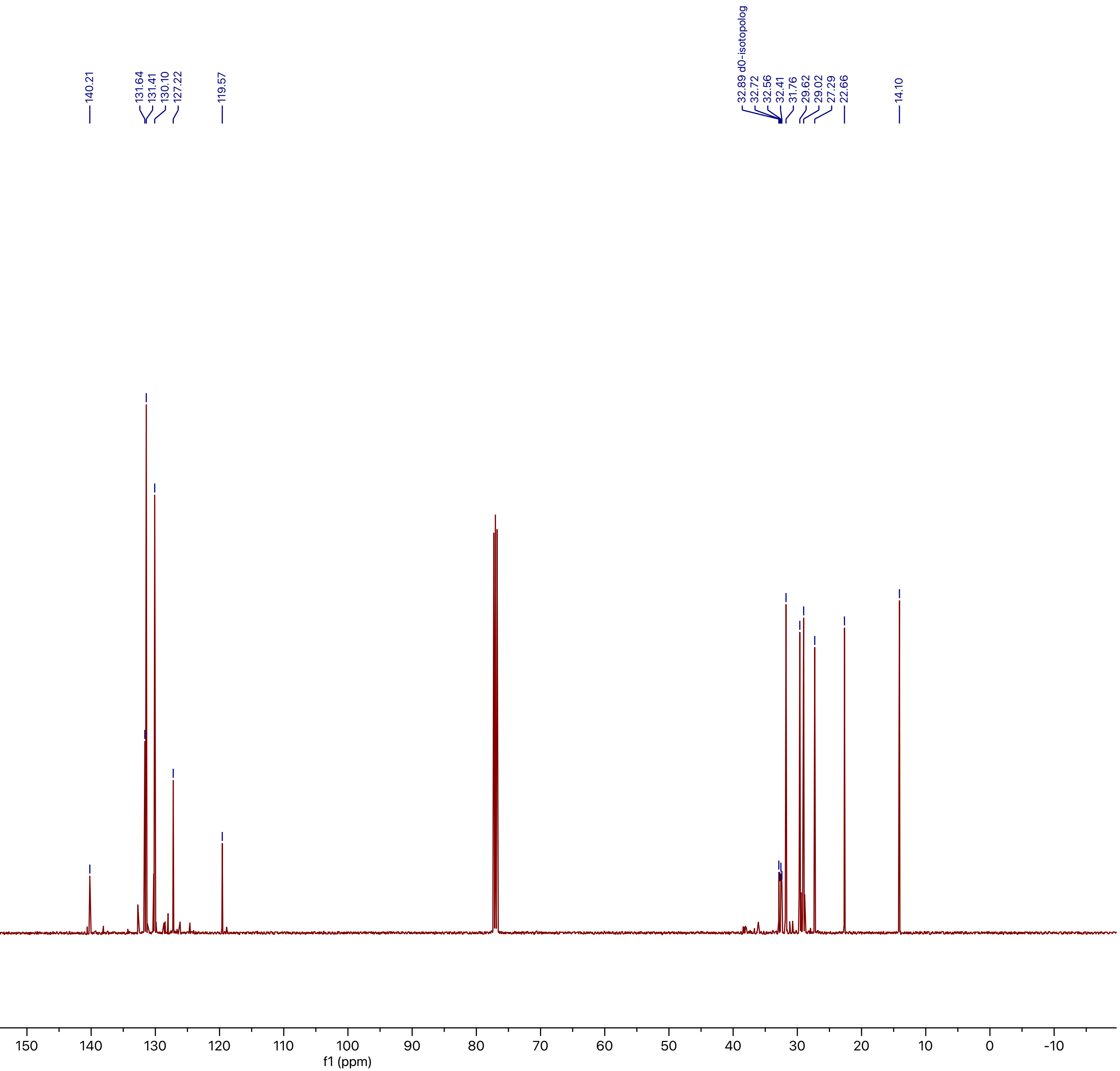

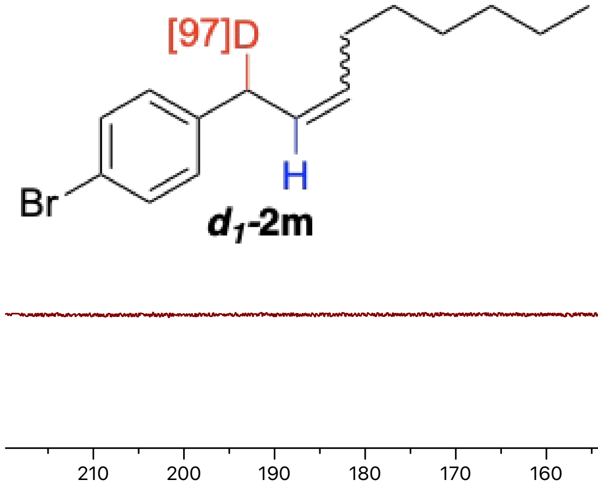

d_1-2m

	Parameter	Value
1	Origin	Varian
2	Solvent	cdcl3
3	Temperature	0.0
4	Pulse Sequence	s2pul
5	Experiment	1D
6	Probe	ASWPFG8319
7	Number of Scans	16
8	Receiver Gain	56
9	Relaxation Delay	10.0000
10	Pulse Width	7.7500
11	Spectrometer Frequency	399.73
12	Spectral Width	6410.3
13	Lowest Frequency	-805.6
14	Nucleus	1H
15	Acquired Size	16384
16	Spectral Size	65536
17	Digital Resolution	0.10

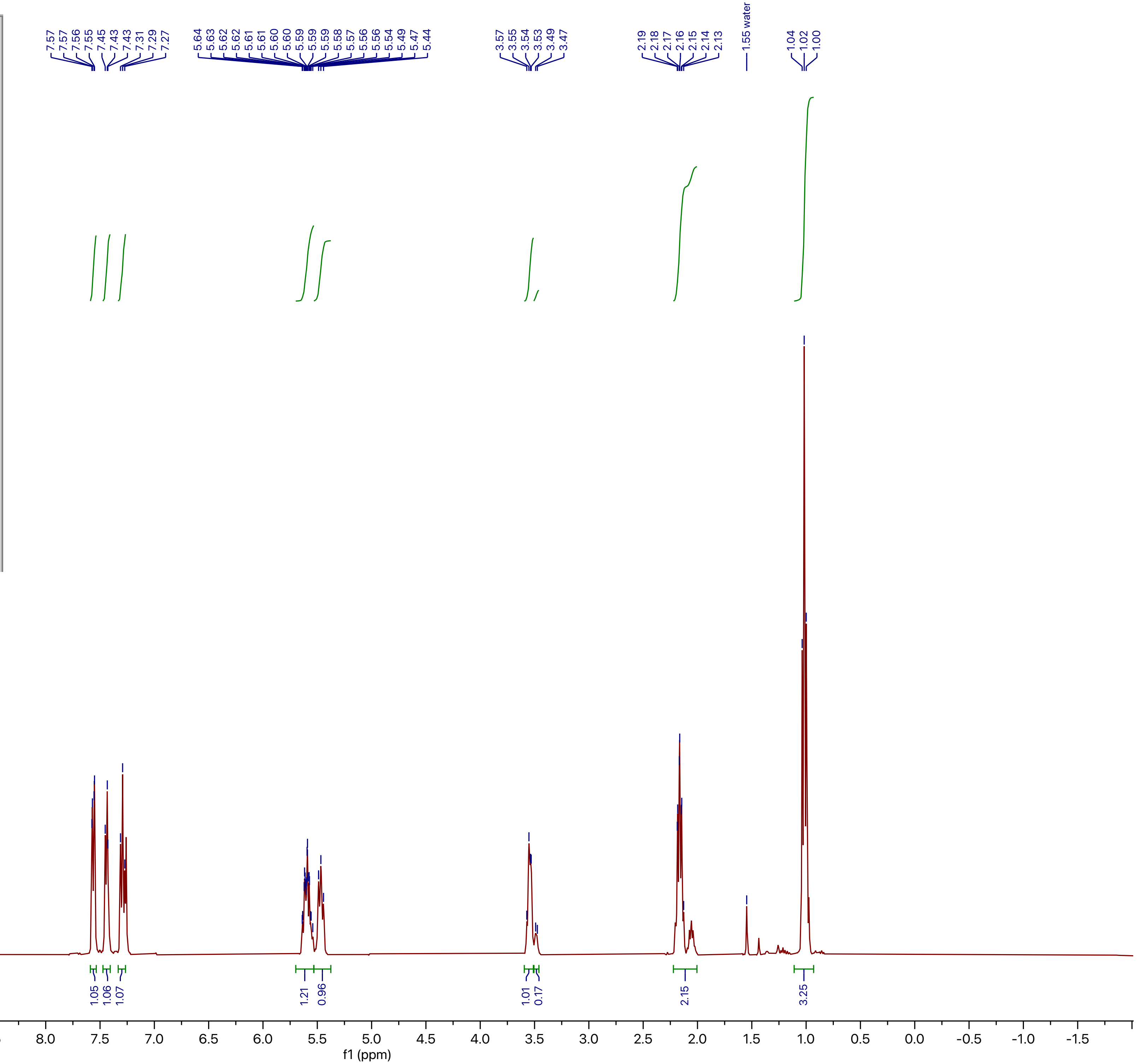

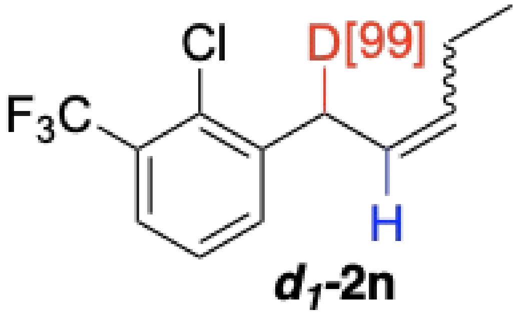

***d*₁-2n**

	Parameter	Value
1	Origin	Varian
2	Solvent	cdcl3
3	Temperature	0.0
4	Pulse Sequence	s2pul
5	Experiment	1D
6	Probe	ASWPFG8319
7	Number of Scans	128
8	Receiver Gain	30
9	Relaxation Delay	0.5000
10	Pulse Width	300.0000
11	Spectrometer Frequency	61.36
12	Spectral Width	552.1
13	Lowest Frequency	-8.2
14	Nucleus	lk
15	Acquired Size	614
16	Spectral Size	2048
17	Digital Resolution	0.27

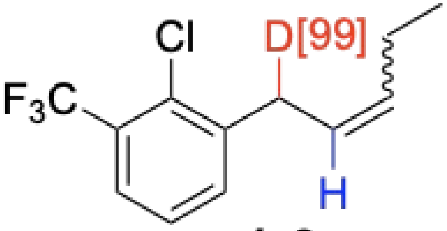

***d*$_1$-2n**

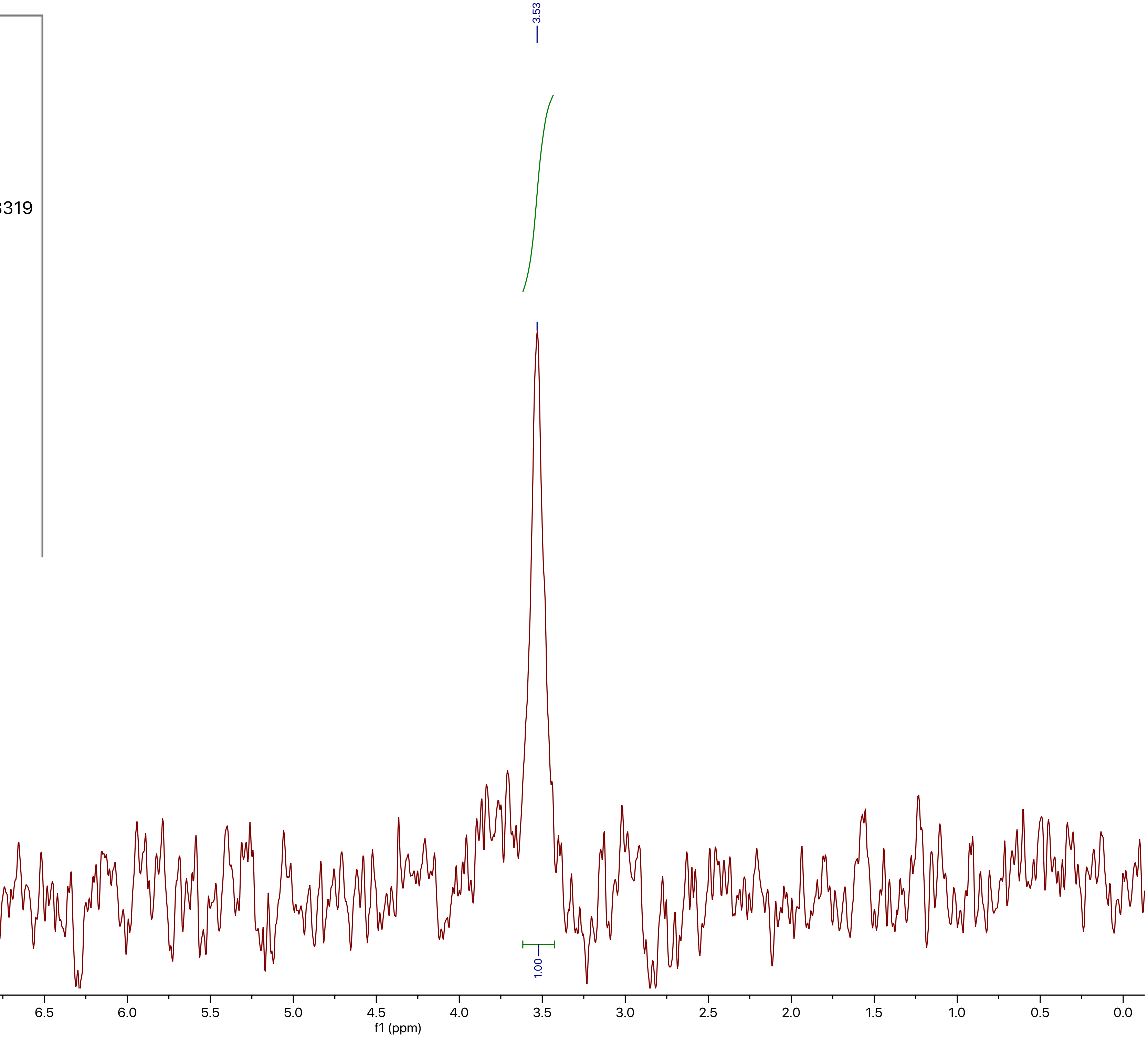

Parameter		Value
1	Origin	Varian
2	Solvent	cdcl3
3	Temperature	0.0
4	Pulse Sequence	s2pul
5	Experiment	1D
6	Probe	ASWPFG8319
7	Number of Scans	512
8	Receiver Gain	30
9	Relaxation Delay	3.0000
10	Pulse Width	5.7500
11	Spectrometer Frequency	100.52
12	Spectral Width	25000.0
13	Lowest Frequency	-1444.1
14	Nucleus	13C
15	Acquired Size	32768
16	Spectral Size	65536
17	Digital Resolution	0.38

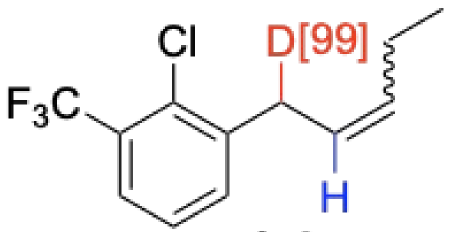

***d*₁-2n**

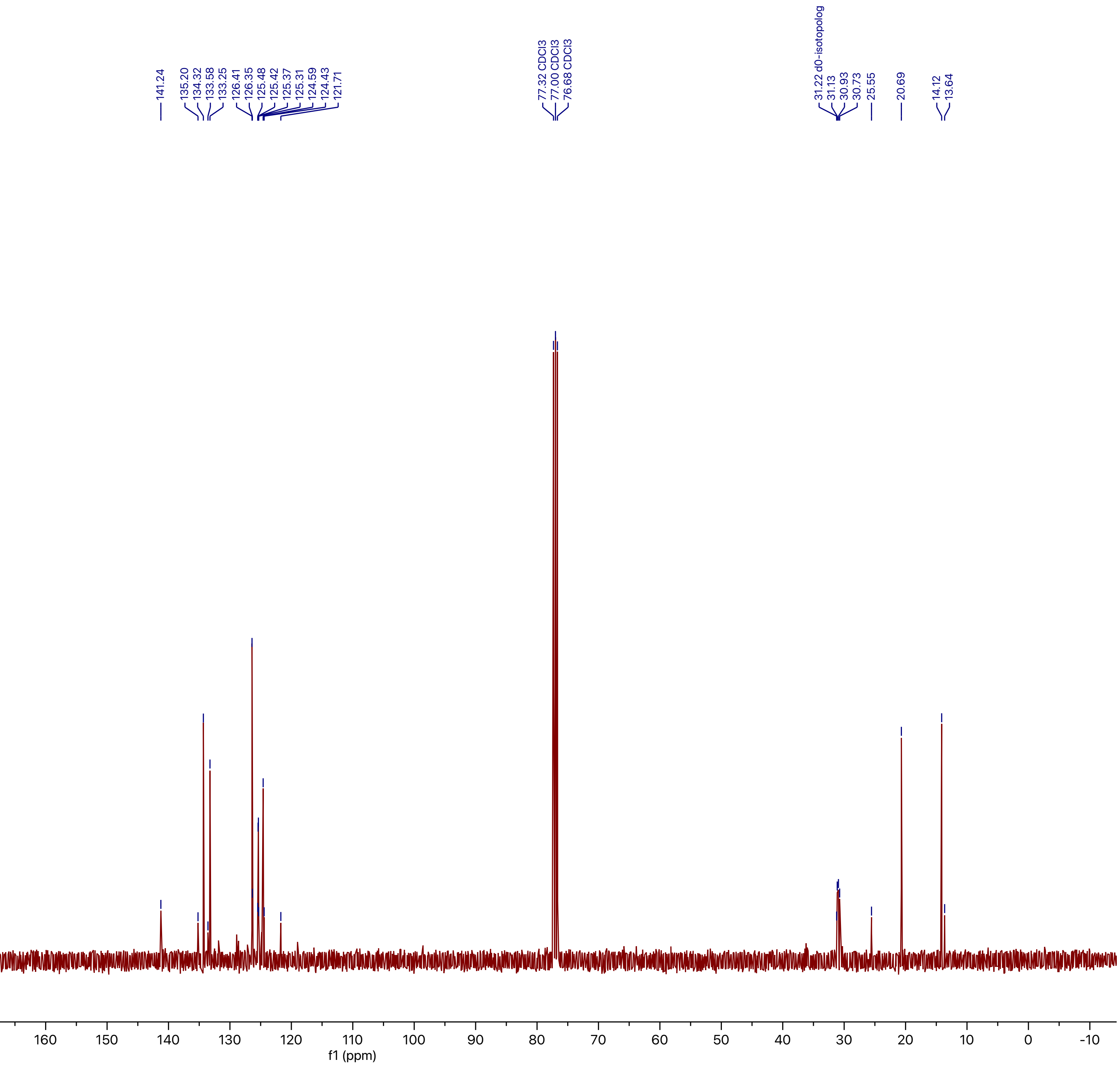

	Parameter	Value
1	Origin	Varian
2	Solvent	cdcl3
3	Temperature	0.0
4	Pulse Sequence	s2pul
5	Experiment	1D
6	Probe	ASWPFG8319
7	Number of Scans	16
8	Receiver Gain	60
9	Relaxation Delay	1.0000
10	Pulse Width	5.0000
11	Spectrometer Frequency	376.09
12	Spectral Width	89285.7
13	Lowest Frequency	-76613.1
14	Nucleus	19F
15	Acquired Size	65536
16	Spectral Size	131072
17	Digital Resolution	0.68

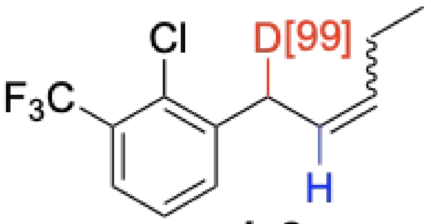

***d*$_1$-2n**

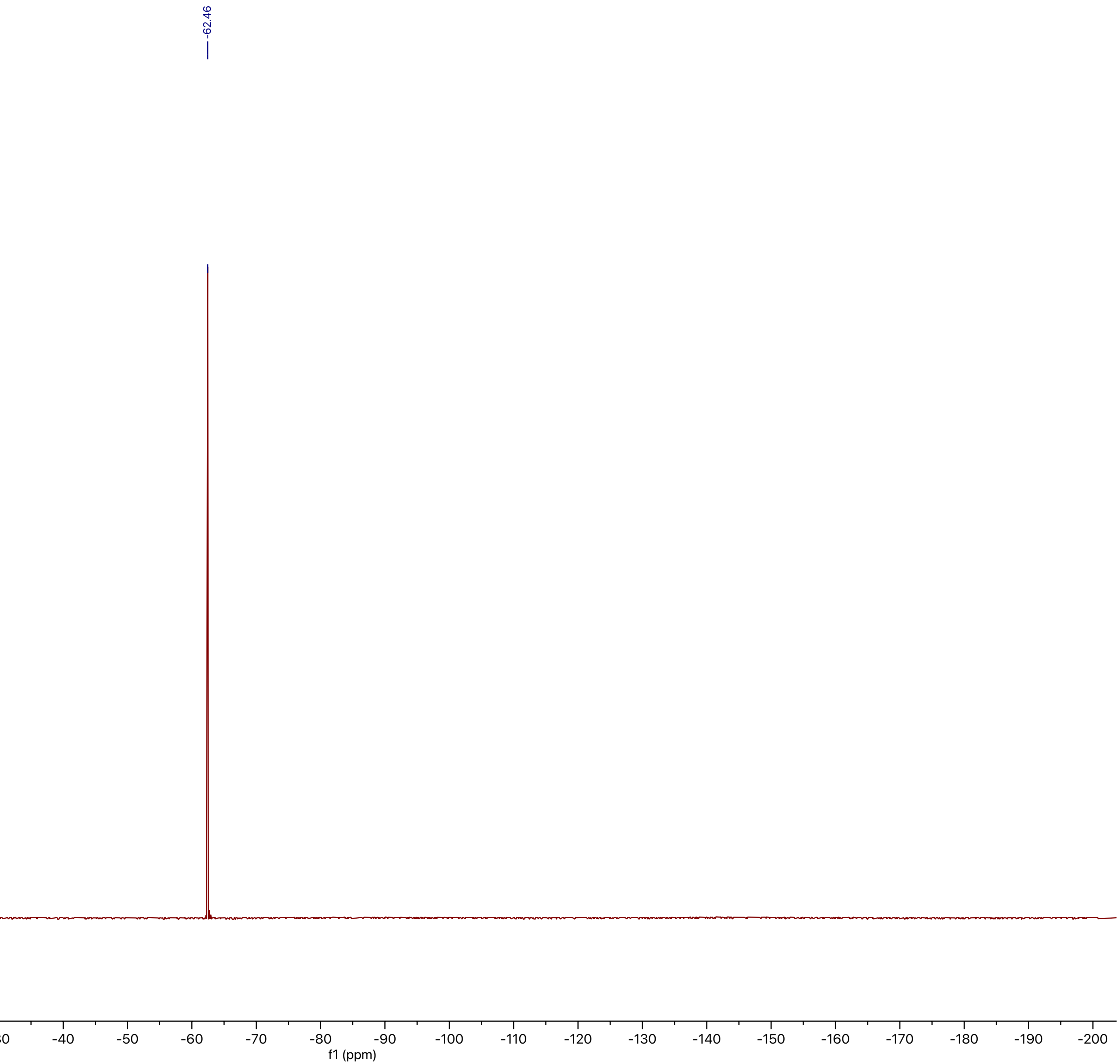

	Parameter	Value
1	Origin	Varian
2	Solvent	cdcl3
3	Temperature	25.0
4	Pulse Sequence	s2pul
5	Experiment	1D
6	Probe	ASWPFG8319
7	Number of Scans	16
8	Receiver Gain	48
9	Relaxation Delay	10.0000
10	Pulse Width	7.7500
11	Spectrometer Frequency	399.73
12	Spectral Width	6410.3
13	Lowest Frequency	-806.6
14	Nucleus	1H
15	Acquired Size	16384
16	Spectral Size	65536
17	Digital Resolution	0.10

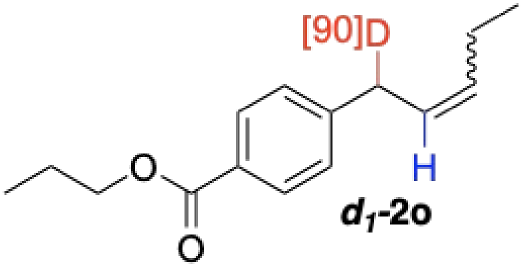

d$_1$-**2o**

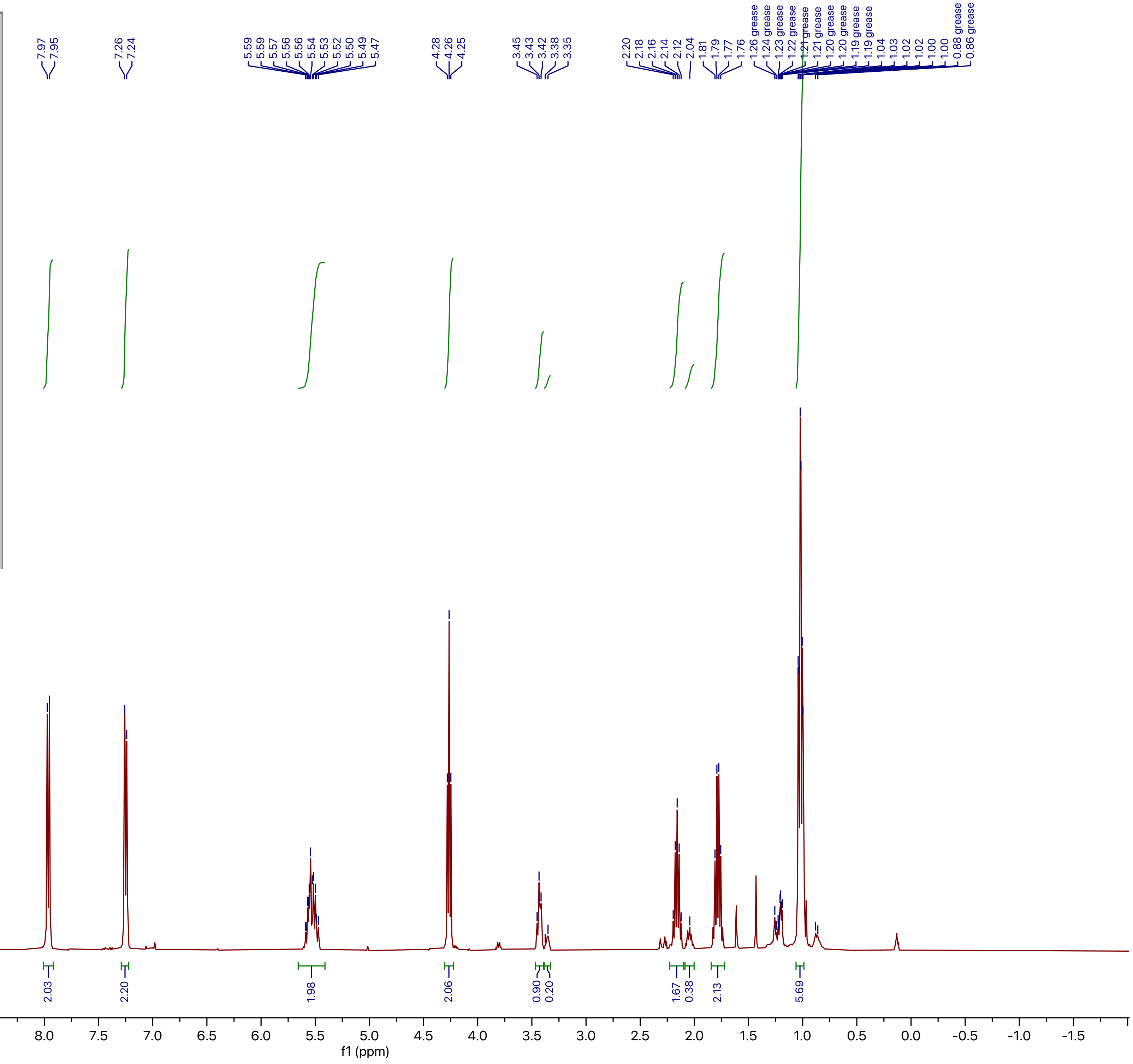

	Parameter	Value
1	Origin	Varian
2	Solvent	cdcl3
3	Temperature	25.0
4	Pulse Sequence	s2pul
5	Experiment	1D
6	Probe	ASWPFG8319
7	Number of Scans	128
8	Receiver Gain	30
9	Relaxation Delay	0.5000
10	Pulse Width	300.0000
11	Spectrometer Frequency	61.36
12	Spectral Width	552.1
13	Lowest Frequency	-1.4
14	Nucleus	lk
15	Acquired Size	614
16	Spectral Size	2048
17	Digital Resolution	0.27

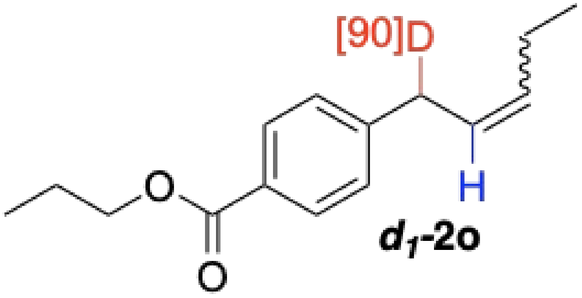

d_1-**2o**

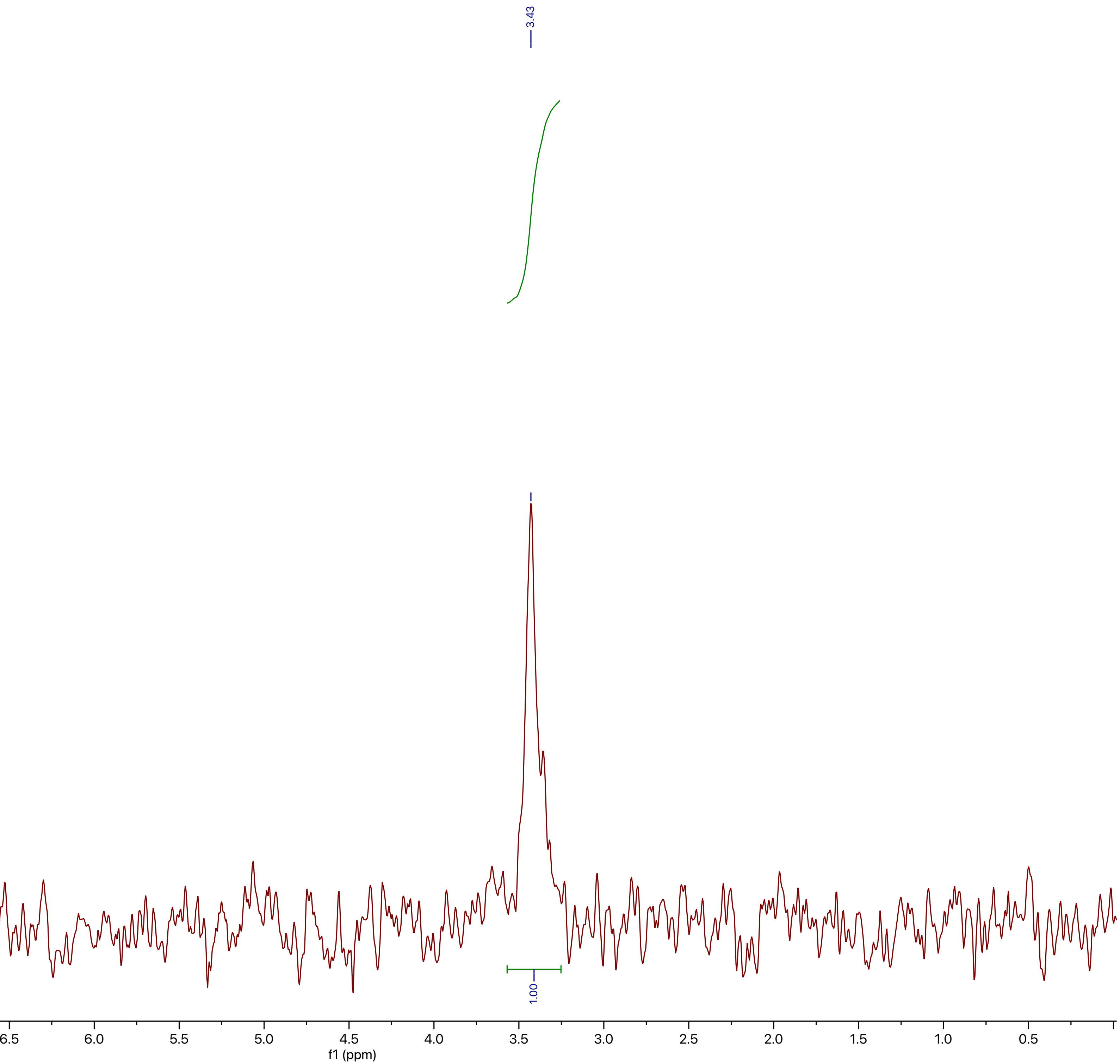

Parameter		Value
1	Origin	Varian
2	Solvent	cdcl3
3	Temperature	25.0
4	Pulse Sequence	s2pul
5	Experiment	1D
6	Probe	ASWPFG8319
7	Number of Scans	512
8	Receiver Gain	30
9	Relaxation Delay	2.0000
10	Pulse Width	5.7500
11	Spectrometer Frequency	100.52
12	Spectral Width	25000.0
13	Lowest Frequency	-1443.7
14	Nucleus	13C
15	Acquired Size	32768
16	Spectral Size	65536
17	Digital Resolution	0.38

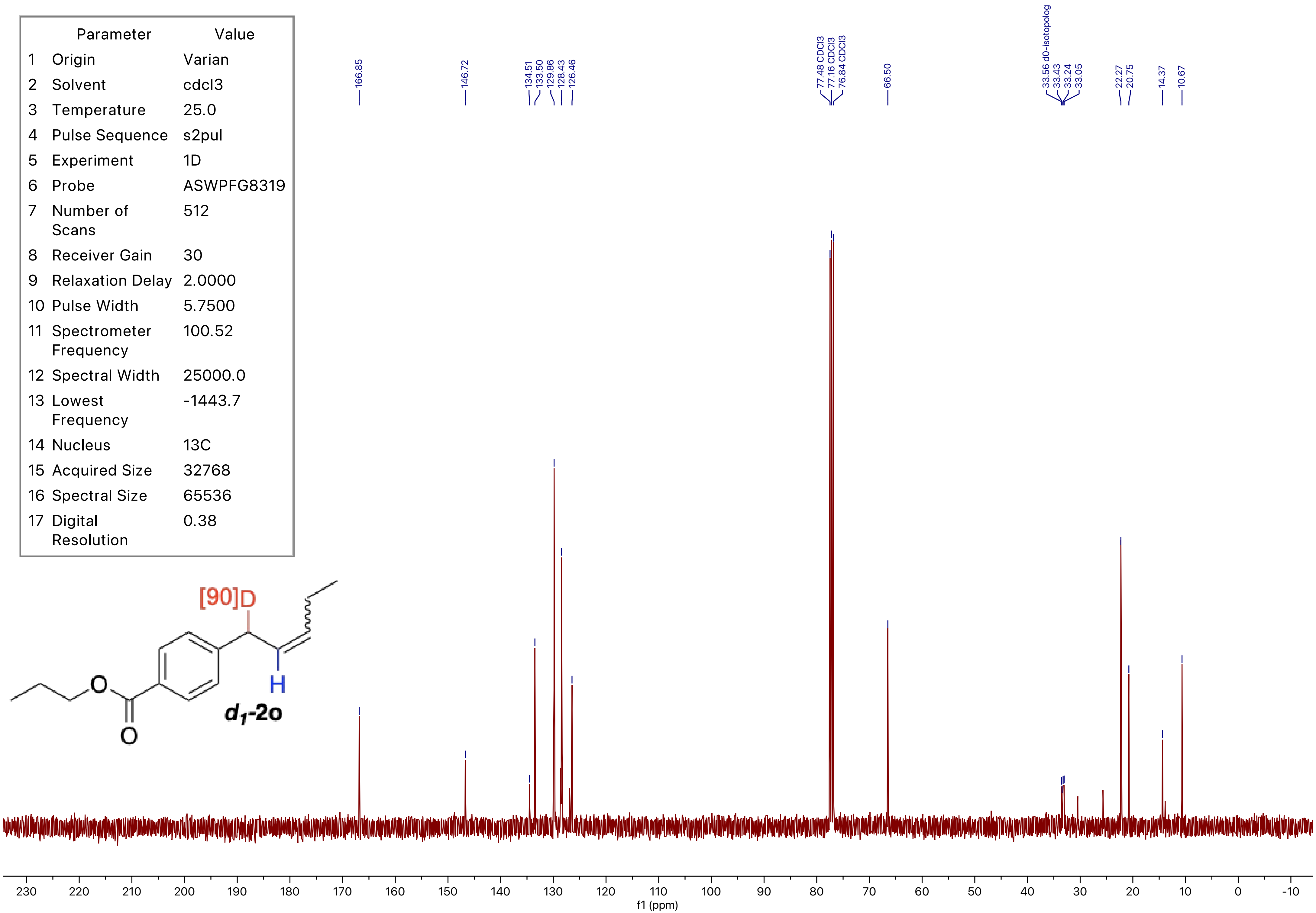

d₁-2o

	Parameter	Value
1	Origin	Bruker BioSpin GmbH
2	Instrument	Avance
3	Solvent	CDCl3
4	Temperature	298.0
5	Pulse Sequence	zg30
6	Experiment	1D
7	Probe	Z163739_0400 (PI HR-BBO400S1-BBF/ H/ D-5.0-Z SP)
8	Number of Scans	16
9	Receiver Gain	101.0
10	Relaxation Delay	1.0000
11	Pulse Width	8.0000
12	Spectrometer Frequency	400.13
13	Spectral Width	8196.7
14	Lowest Frequency	-1636.9
15	Nucleus	1H
16	Acquired Size	32768
17	Spectral Size	65536

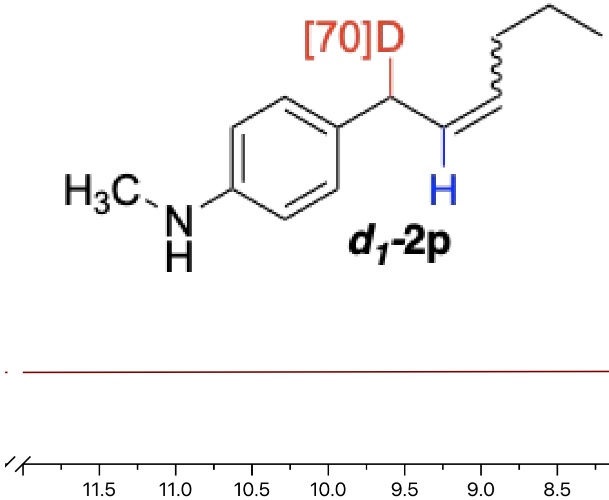

d_1-2p

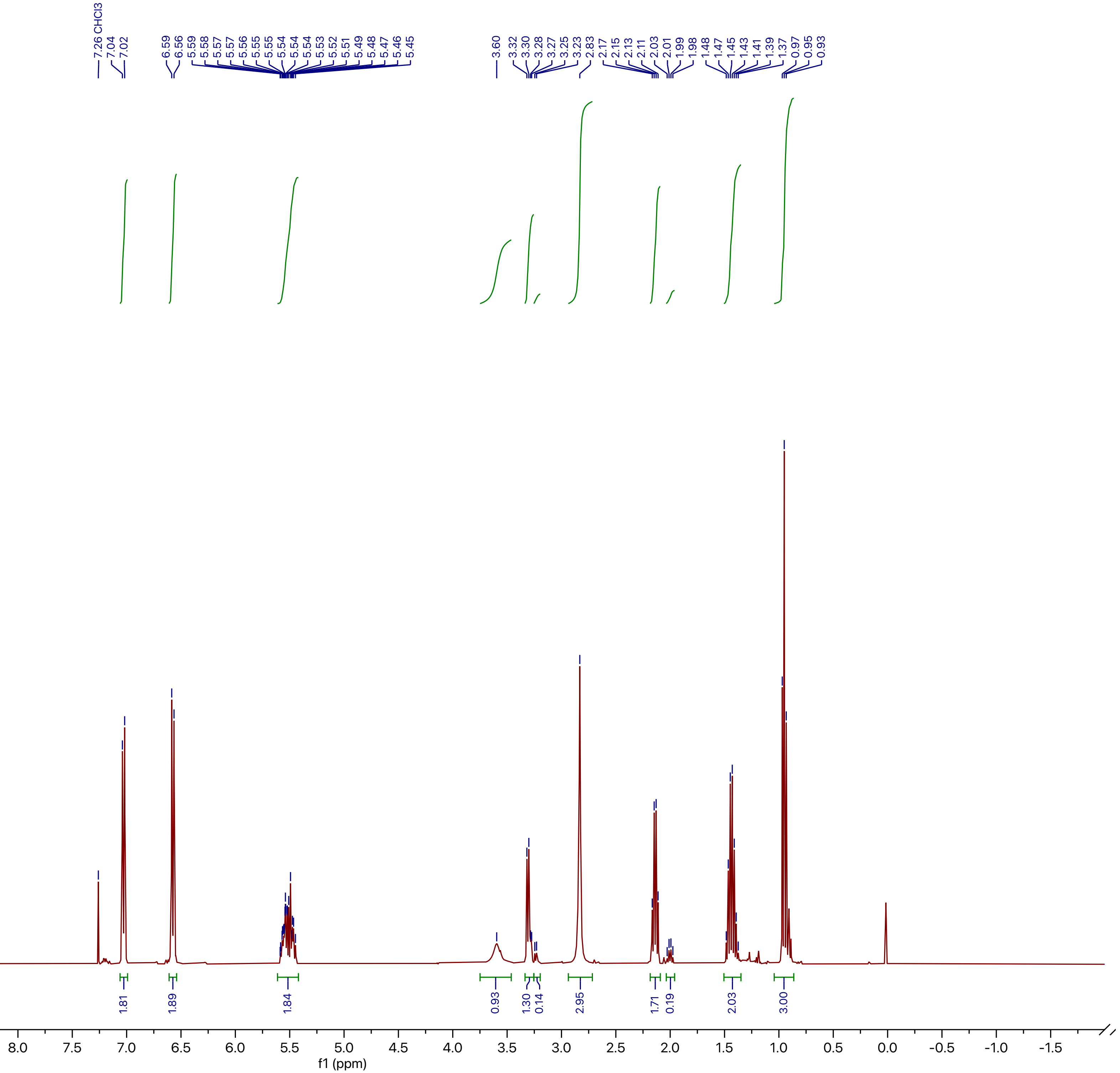

	Parameter	Value
1	Origin	Varian
2	Instrument	vnmrs
3	Solvent	cdcl3
4	Temperature	25.0
5	Pulse Sequence	s2pul
6	Experiment	1D
7	Probe	ASWPFG8319
8	Number of Scans	256
9	Receiver Gain	30
10	Relaxation Delay	0.5000
11	Pulse Width	300.0000
12	Spectrometer Frequency	61.36
13	Spectral Width	552.1
14	Lowest Frequency	0.1
15	Nucleus	"lk"
16	Acquired Size	614
17	Spectral Size	2048

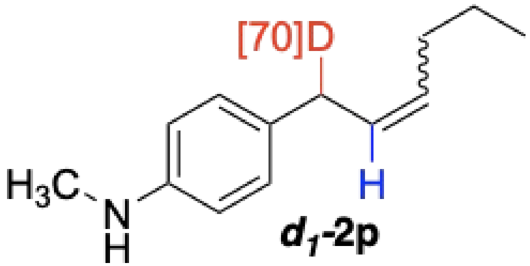

*d*1-2p

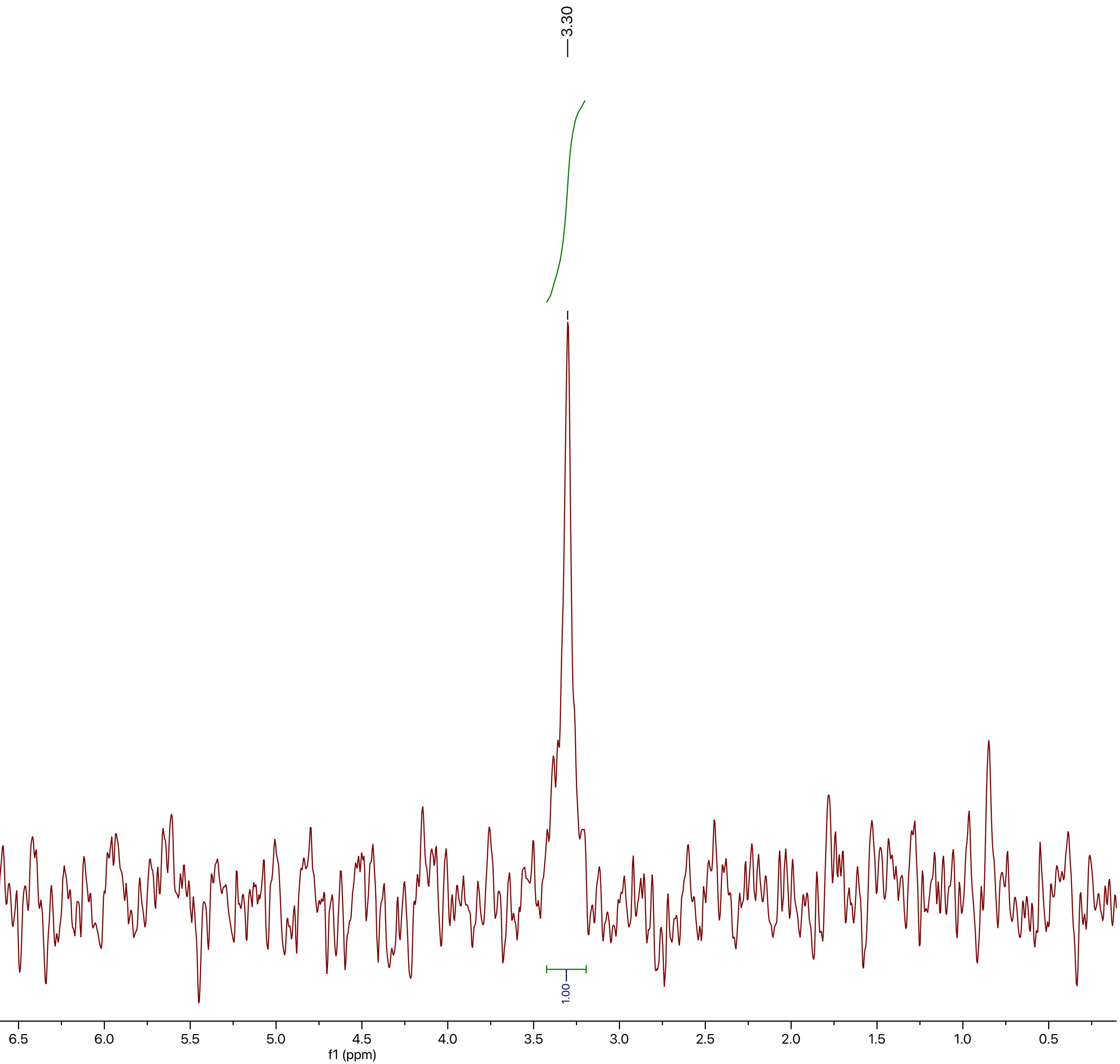

	Parameter	Value
1	Origin	Bruker BioSpin GmbH
2	Instrument	Avance
3	Solvent	CDCl3
4	Temperature	300.0
5	Pulse Sequence	zgpg30
6	Experiment	1D
7	Probe	Z151574_0073 (PI HR-BBO500S1-BBF/ H/ D-5.0-Z SP)
8	Number of Scans	1024
9	Receiver Gain	101.0
10	Relaxation Delay	2.0000
11	Pulse Width	9.0000
12	Spectrometer Frequency	125.79
13	Spectral Width	30120.5
14	Lowest Frequency	-2482.4
15	Nucleus	13C
16	Acquired Size	32768
17	Spectral Size	65536

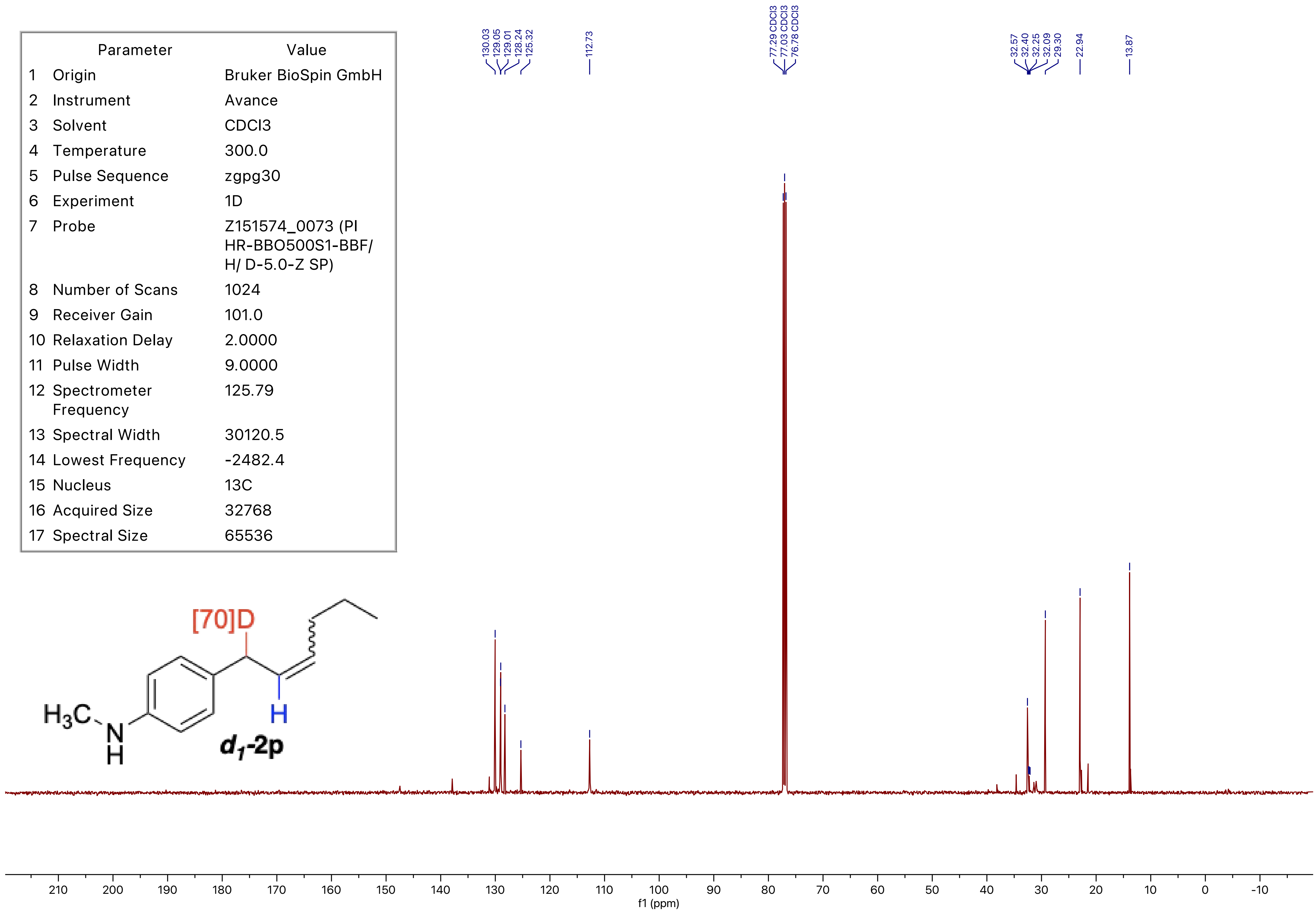

d_1-2p

	Parameter	Value
1	Origin	Bruker BioSpin GmbH
2	Instrument	Avance
3	Solvent	CDCl3
4	Temperature	298.0
5	Pulse Sequence	zg30
6	Experiment	1D
7	Probe	Z163739_0400 (PI HR-BBO400S1-BBF/ H/ D-5.0-Z SP)
8	Number of Scans	16
9	Receiver Gain	101.0
10	Relaxation Delay	1.0000
11	Pulse Width	8.0000
12	Spectrometer Frequency	400.13
13	Spectral Width	8196.7
14	Lowest Frequency	-1636.9
15	Nucleus	1H
16	Acquired Size	32768
17	Spectral Size	65536

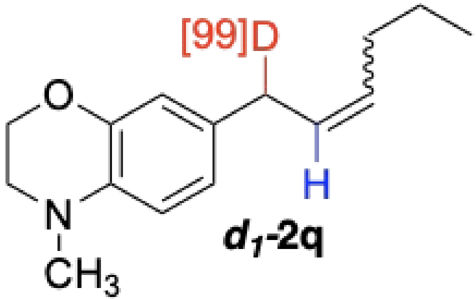

d_1-2q

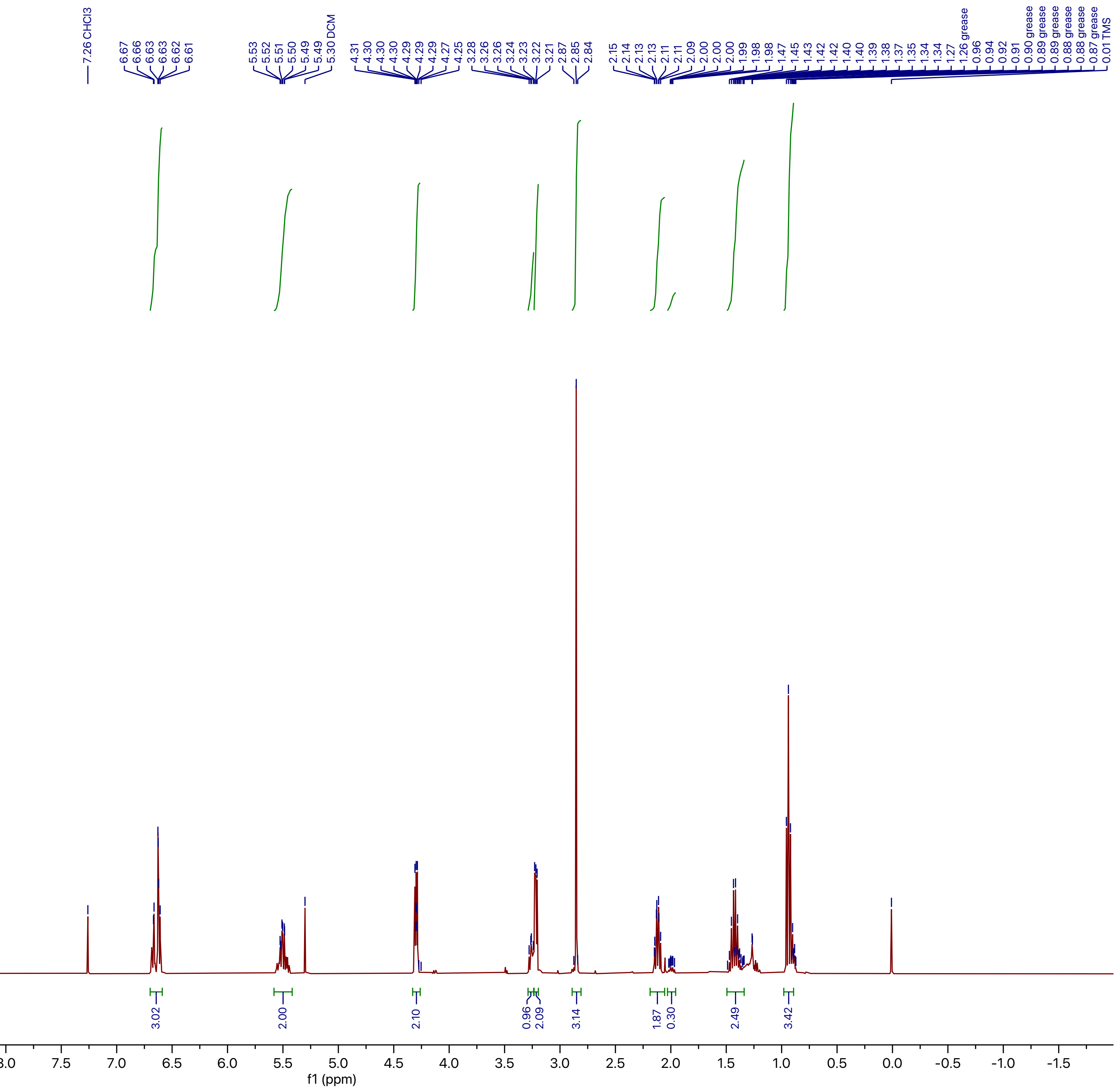

	Parameter	Value
1	Origin	Varian
2	Instrument	vnmrs
3	Solvent	cdcl3
4	Temperature	25.0
5	Pulse Sequence	s2pul
6	Experiment	1D
7	Probe	ASWPFG8319
8	Number of Scans	256
9	Receiver Gain	30
10	Relaxation Delay	0.5000
11	Pulse Width	300.0000
12	Spectrometer Frequency	61.36
13	Spectral Width	552.1
14	Lowest Frequency	5.5
15	Nucleus	"lk"
16	Acquired Size	614
17	Spectral Size	2048

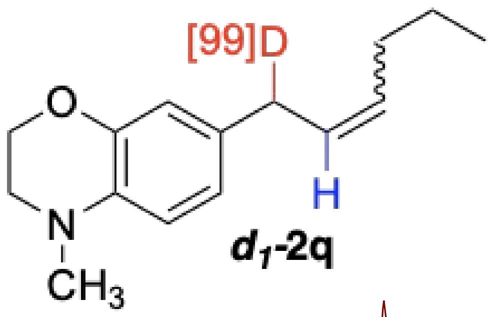

d_1-2q

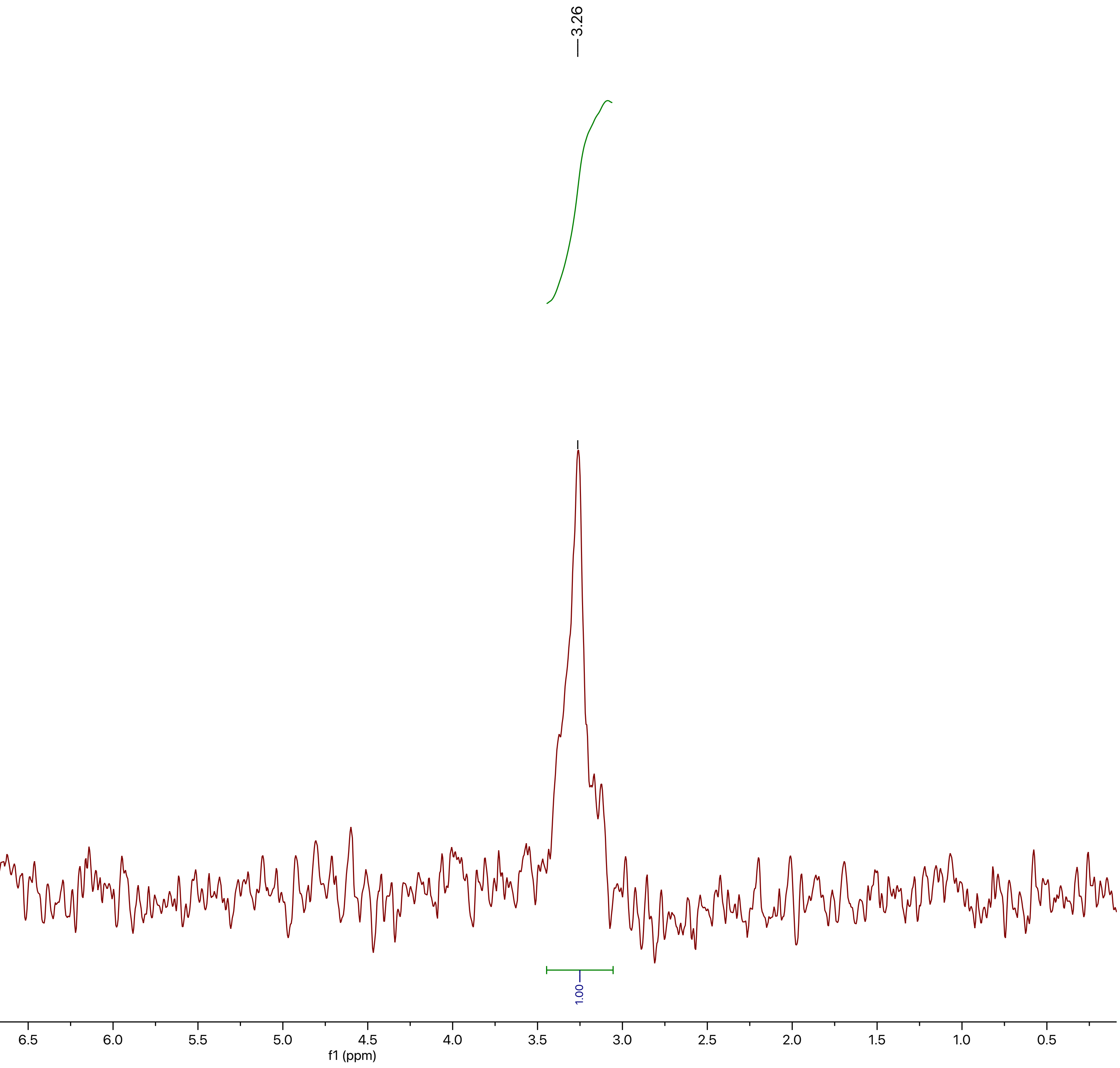

	Parameter	Value
1	Origin	Bruker BioSpin GmbH
2	Instrument	Avance
3	Solvent	CDCl3
4	Temperature	298.0
5	Pulse Sequence	zgpg30
6	Experiment	1D
7	Probe	Z163739_0400 (PI HR-BBO400S1-BBF/ H/ D-5.0-Z SP)
8	Number of Scans	1024
9	Receiver Gain	101.0
10	Relaxation Delay	2.0000
11	Pulse Width	8.0000
12	Spectrometer Frequency	100.62
13	Spectral Width	23809.5
14	Lowest Frequency	-1830.0
15	Nucleus	13C
16	Acquired Size	32768
17	Spectral Size	65536

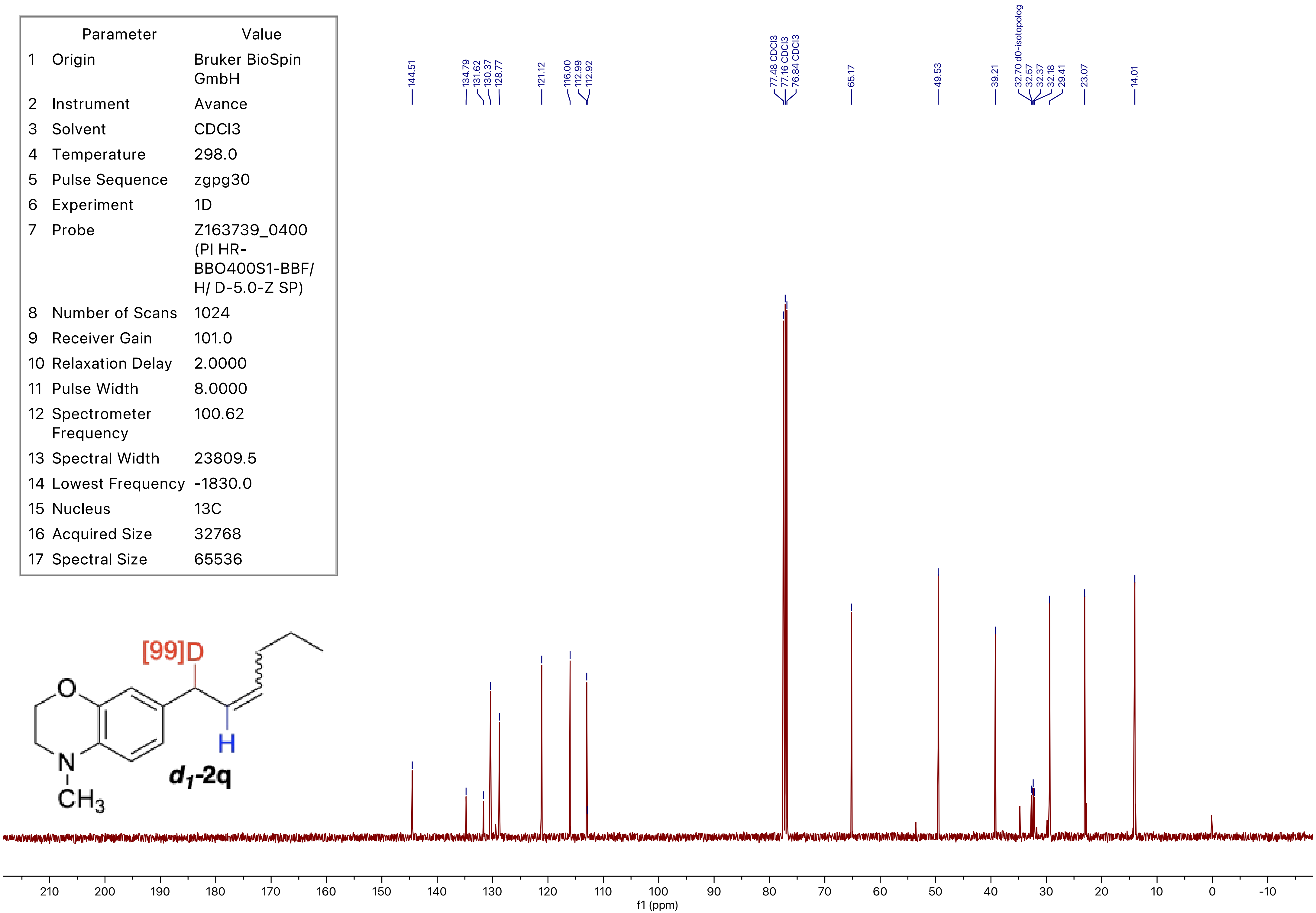

	Parameter	Value
1	Origin	Bruker BioSpin GmbH
2	Instrument	Avance
3	Solvent	CDCl3
4	Temperature	298.0
5	Pulse Sequence	zg30
6	Experiment	1D
7	Probe	Z163739_0400 (PI HR-BBO400S1-BBF/ H/ D-5.0-Z SP)
8	Number of Scans	16
9	Receiver Gain	101.0
10	Relaxation Delay	1.0000
11	Pulse Width	8.0000
12	Spectrometer Frequency	400.13
13	Spectral Width	8196.7
14	Lowest Frequency	-1636.9
15	Nucleus	1H
16	Acquired Size	32768
17	Spectral Size	65536

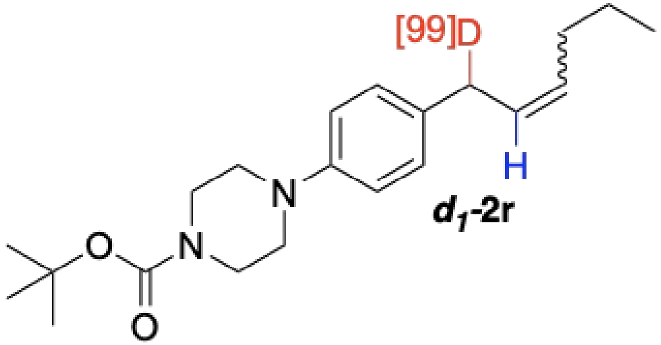

***d*$_1$-2r**

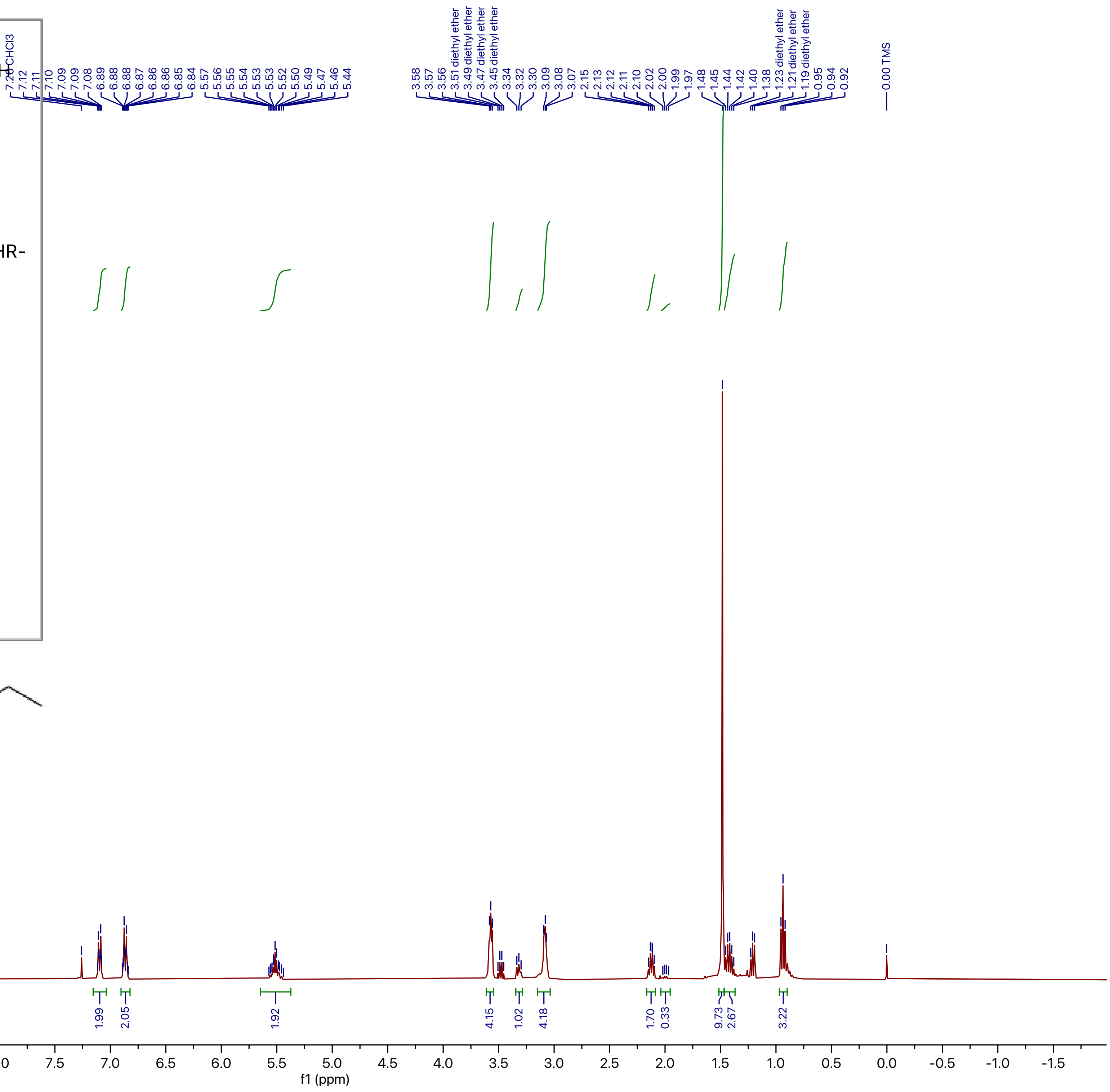

	Parameter	Value
1	Origin	Varian
2	Instrument	vnmrs
3	Solvent	cdcl3
4	Temperature	25.0
5	Pulse Sequence	s2pul
6	Experiment	1D
7	Probe	ASWPFG8319
8	Number of Scans	256
9	Receiver Gain	30
10	Relaxation Delay	0.5000
11	Pulse Width	300.0000
12	Spectrometer Frequency	61.36
13	Spectral Width	552.1
14	Lowest Frequency	-8.5
15	Nucleus	"lk"
16	Acquired Size	614
17	Spectral Size	2048

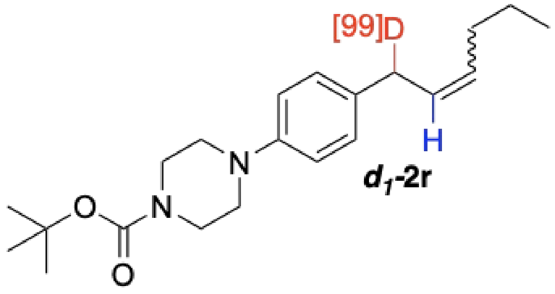

d_1-2r

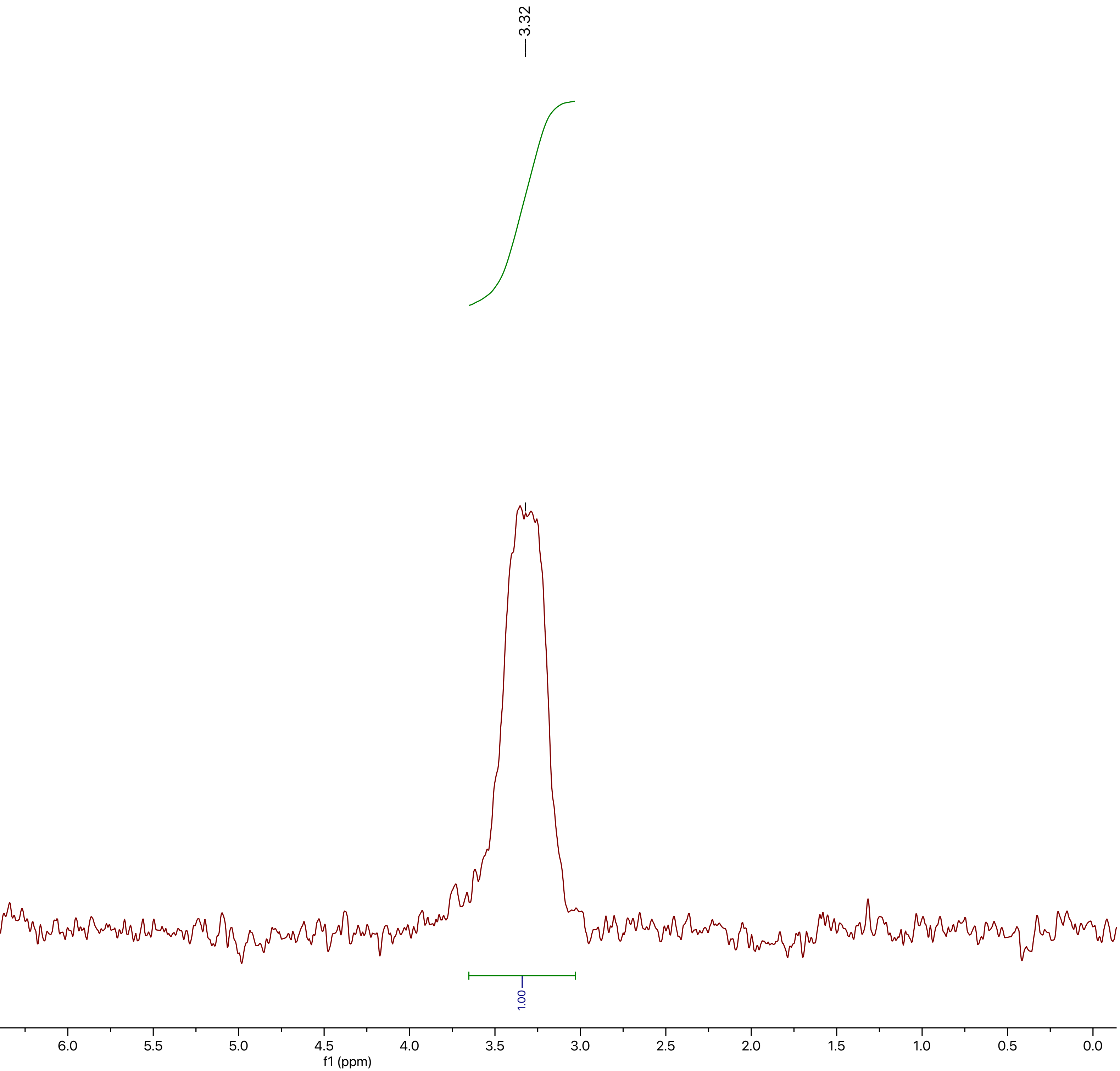

	Parameter	Value
1	Origin	Bruker BioSpin GmbH
2	Instrument	Avance
3	Solvent	CDCl3
4	Temperature	298.0
5	Pulse Sequence	zgpg30
6	Experiment	1D
7	Probe	Z163739_0400 (PI HR-BBO400S1-BBF/ H/ D-5.0-Z SP)
8	Number of Scans	1024
9	Receiver Gain	101.0
10	Relaxation Delay	2.0000
11	Pulse Width	8.0000
12	Spectrometer Frequency	100.62
13	Spectral Width	23809.5
14	Lowest Frequency	-1830.1
15	Nucleus	13C
16	Acquired Size	32768
17	Spectral Size	65536

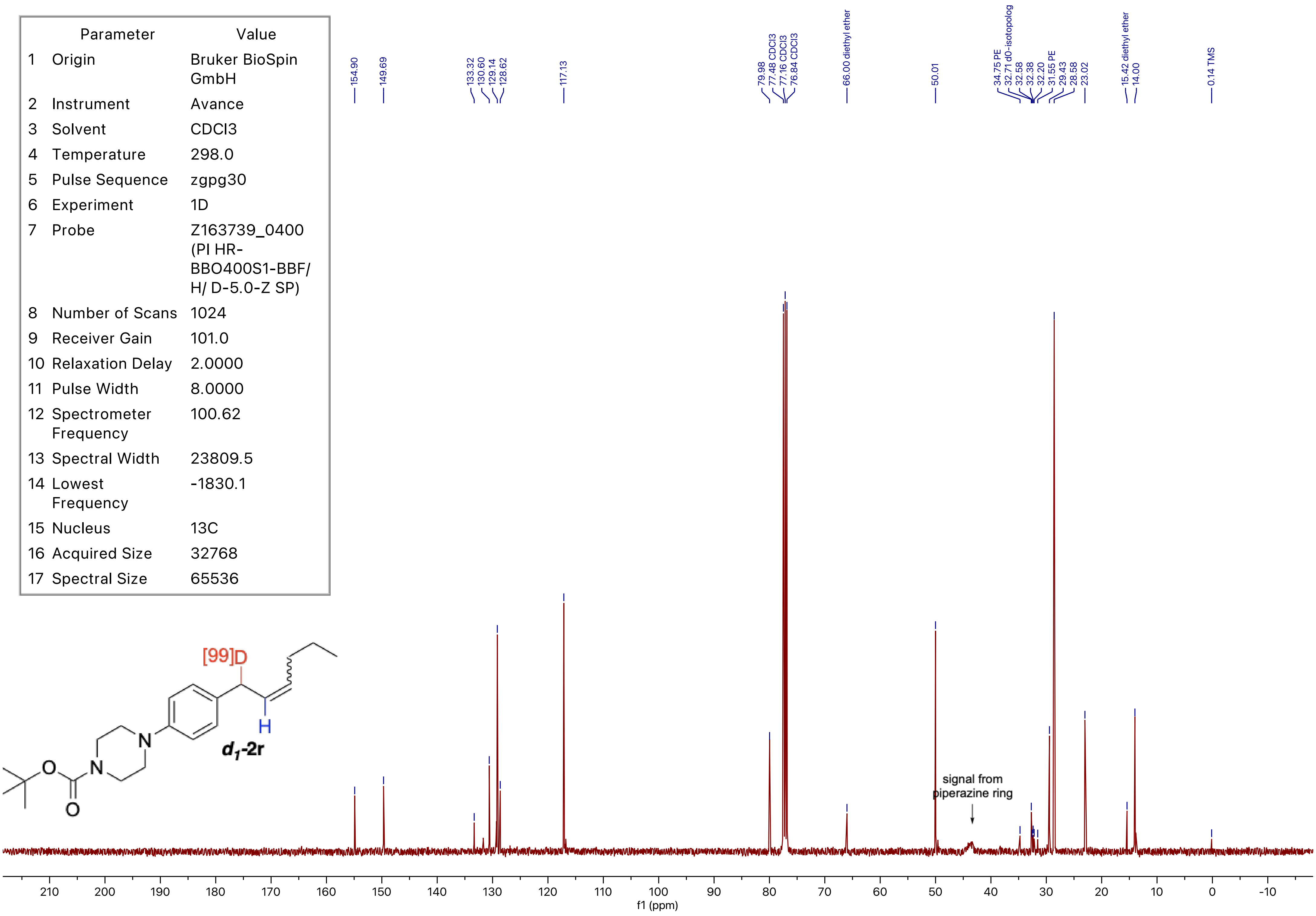

	Parameter	Value
1	Origin	Bruker BioSpin GmbH
2	Instrument	Avance
3	Solvent	CDCl3
4	Temperature	300.0
5	Pulse Sequence	zg30
6	Experiment	1D
7	Probe	Z151574_0073 (PI HR-BBO500S1-BBF/ H/ D-5.0-Z SP)
8	Number of Scans	16
9	Receiver Gain	101.0
10	Relaxation Delay	1.0000
11	Pulse Width	8.0000
12	Spectrometer Frequency	500.21
13	Spectral Width	10000.0
14	Lowest Frequency	-1914.3
15	Nucleus	1H
16	Acquired Size	32768
17	Spectral Size	65536

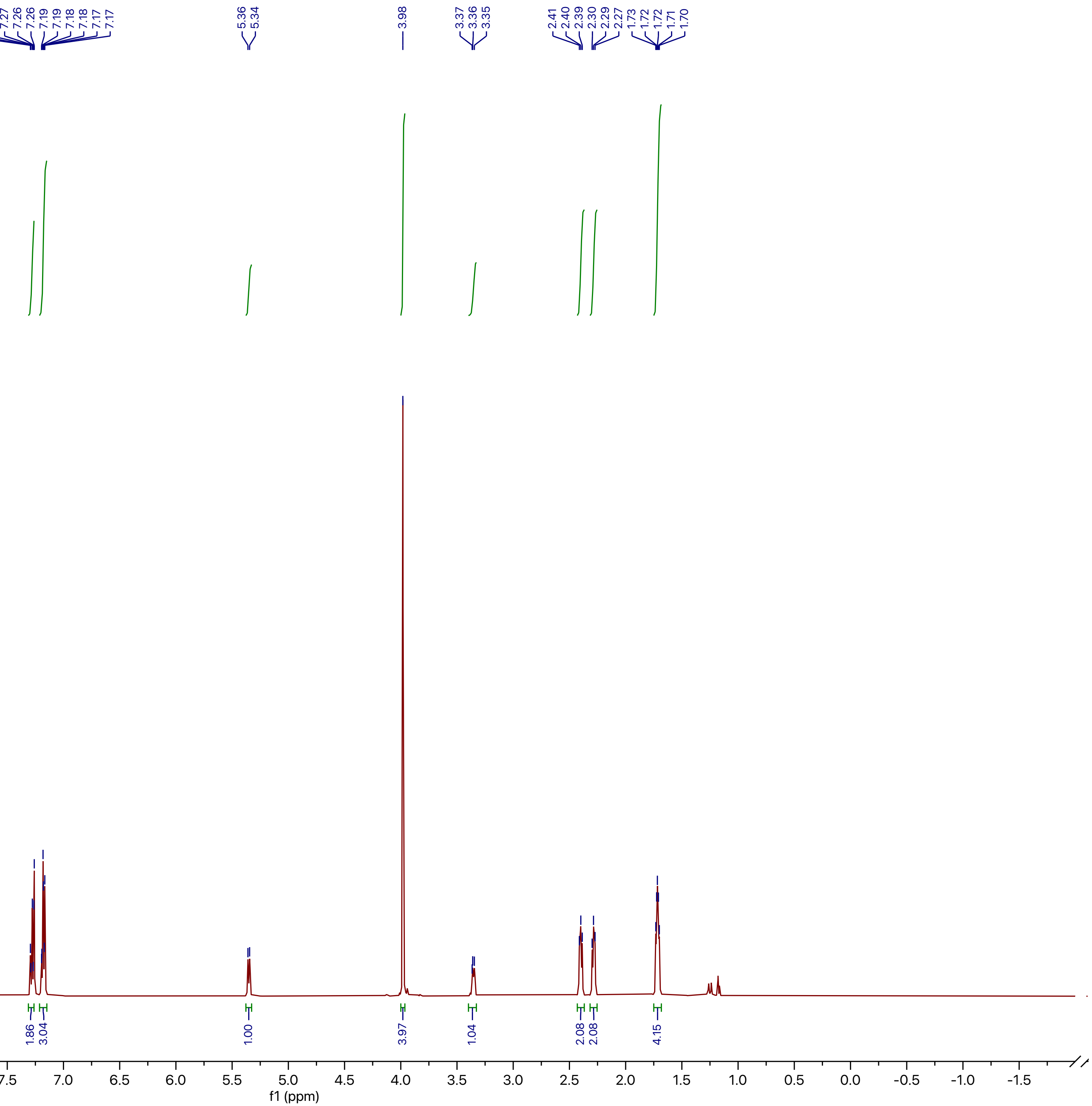

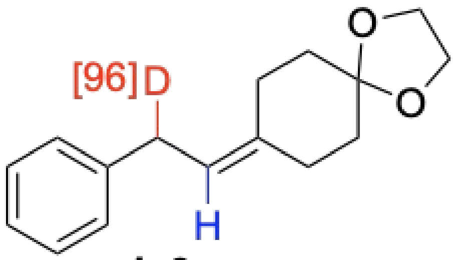

***d*$_1$-2s**

	Parameter	Value
1	Origin	Varian
2	Solvent	cdcl3
3	Temperature	25.0
4	Pulse Sequence	s2pul
5	Experiment	1D
6	Probe	OneNMR_W036
7	Number of Scans	32
8	Receiver Gain	20
9	Relaxation Delay	5.0000
10	Pulse Width	300.0000
11	Spectrometer Frequency	76.71
12	Spectral Width	1535.6
13	Lowest Frequency	-384.2
14	Nucleus	lk
15	Acquired Size	2048
16	Spectral Size	4096
17	Digital Resolution	0.37

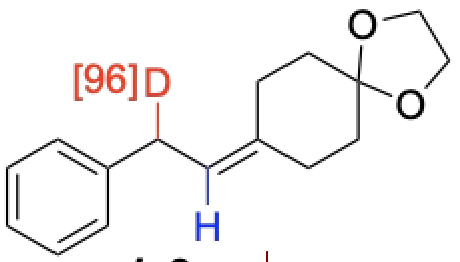

d_1-2s

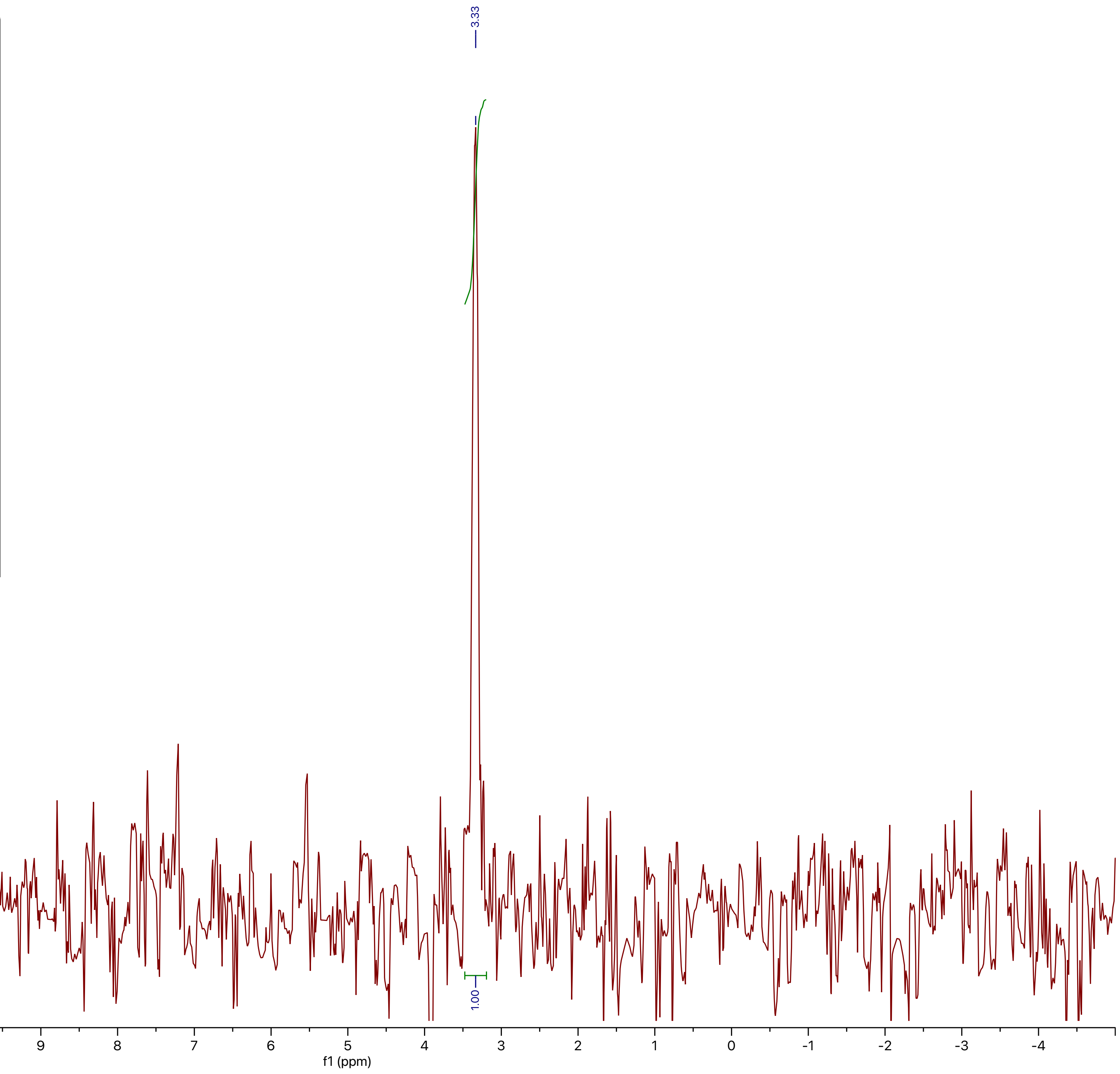

	Parameter	Value
1	Origin	Bruker BioSpin GmbH
2	Instrument	Avance
3	Solvent	CDCl3
4	Temperature	300.0
5	Pulse Sequence	zgpg30
6	Experiment	1D
7	Probe	Z151574_0073 (PI HR-BBO500S1-BBF/ H/ D-5.0-Z SP)
8	Number of Scans	4000
9	Receiver Gain	101.0
10	Relaxation Delay	10.0000
11	Pulse Width	9.0000
12	Spectrometer Frequency	125.79
13	Spectral Width	30120.5
14	Lowest Frequency	-2482.4
15	Nucleus	13C
16	Acquired Size	32768
17	Spectral Size	65536

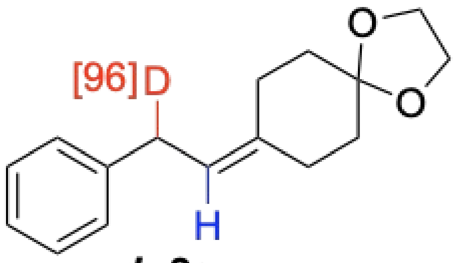

***d*$_1$-2s**

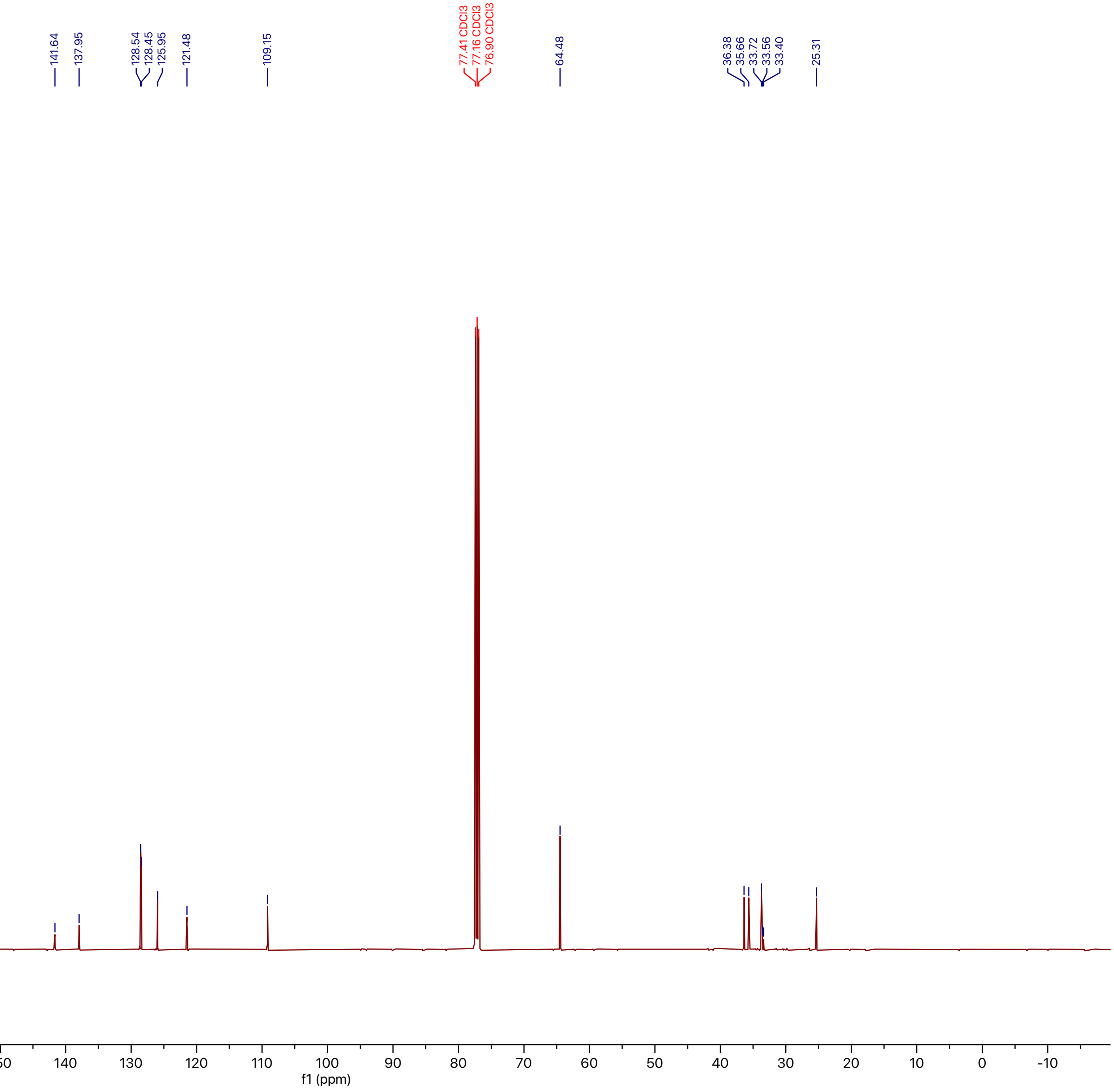

	Parameter	Value
1	Origin	Varian
2	Solvent	cdcl3
3	Temperature	25.0
4	Pulse Sequence	s2pul
5	Experiment	1D
6	Probe	ASWPFG8319
7	Number of Scans	16
8	Receiver Gain	26
9	Relaxation Delay	10.0000
10	Pulse Width	7.7500
11	Spectrometer Frequency	399.73
12	Spectral Width	6410.3
13	Lowest Frequency	-805.6
14	Nucleus	1H
15	Acquired Size	16384
16	Spectral Size	65536
17	Digital Resolution	0.10

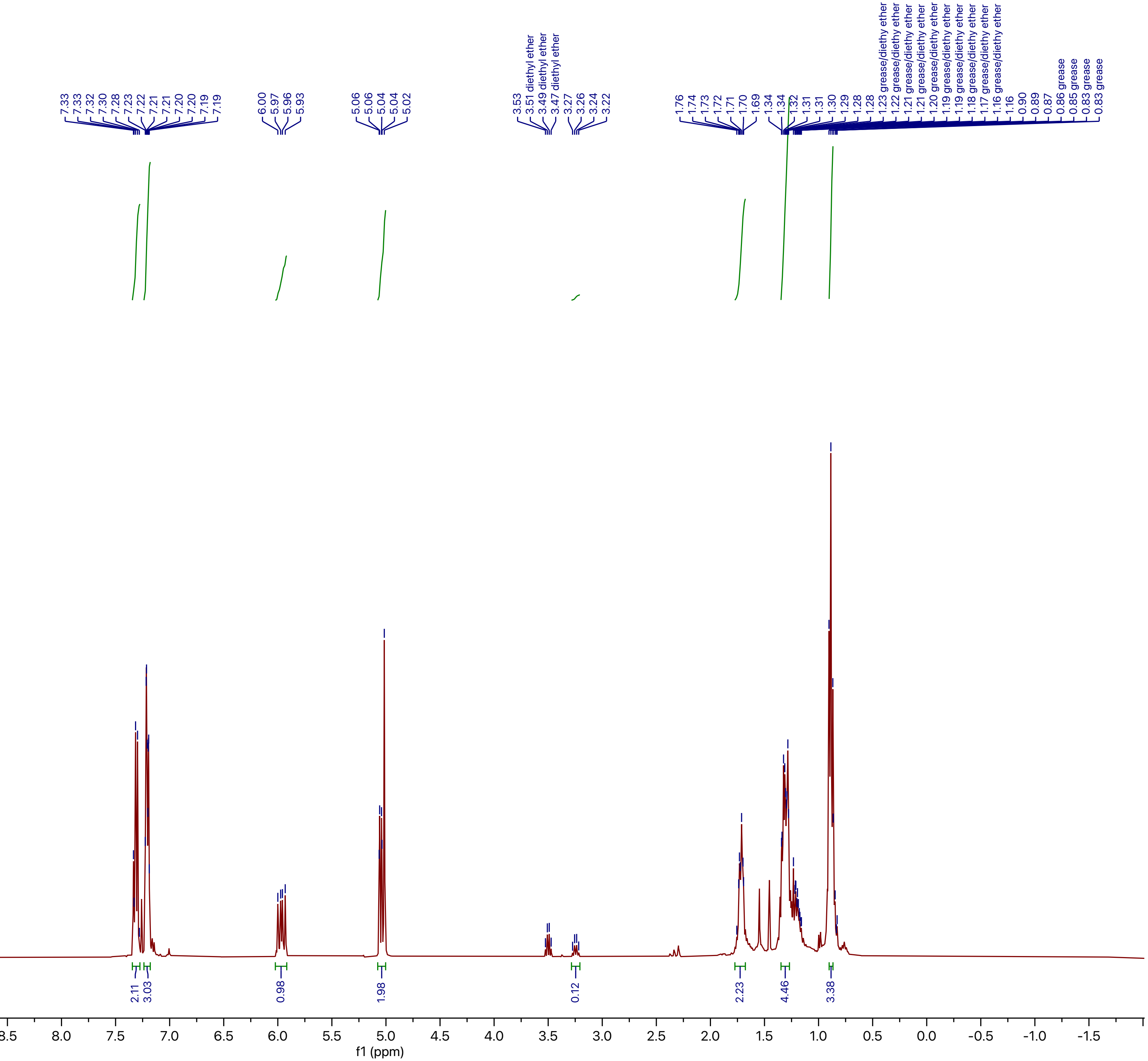

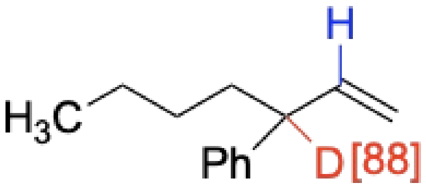

d_1-2t

	Parameter	Value
1	Origin	Varian
2	Solvent	cdcl3
3	Temperature	25.0
4	Pulse Sequence	s2pul
5	Experiment	1D
6	Probe	ASWPFG8319
7	Number of Scans	128
8	Receiver Gain	30
9	Relaxation Delay	0.5000
10	Pulse Width	300.0000
11	Spectrometer Frequency	61.36
12	Spectral Width	552.1
13	Lowest Frequency	0.9
14	Nucleus	lk
15	Acquired Size	614
16	Spectral Size	2048
17	Digital Resolution	0.27

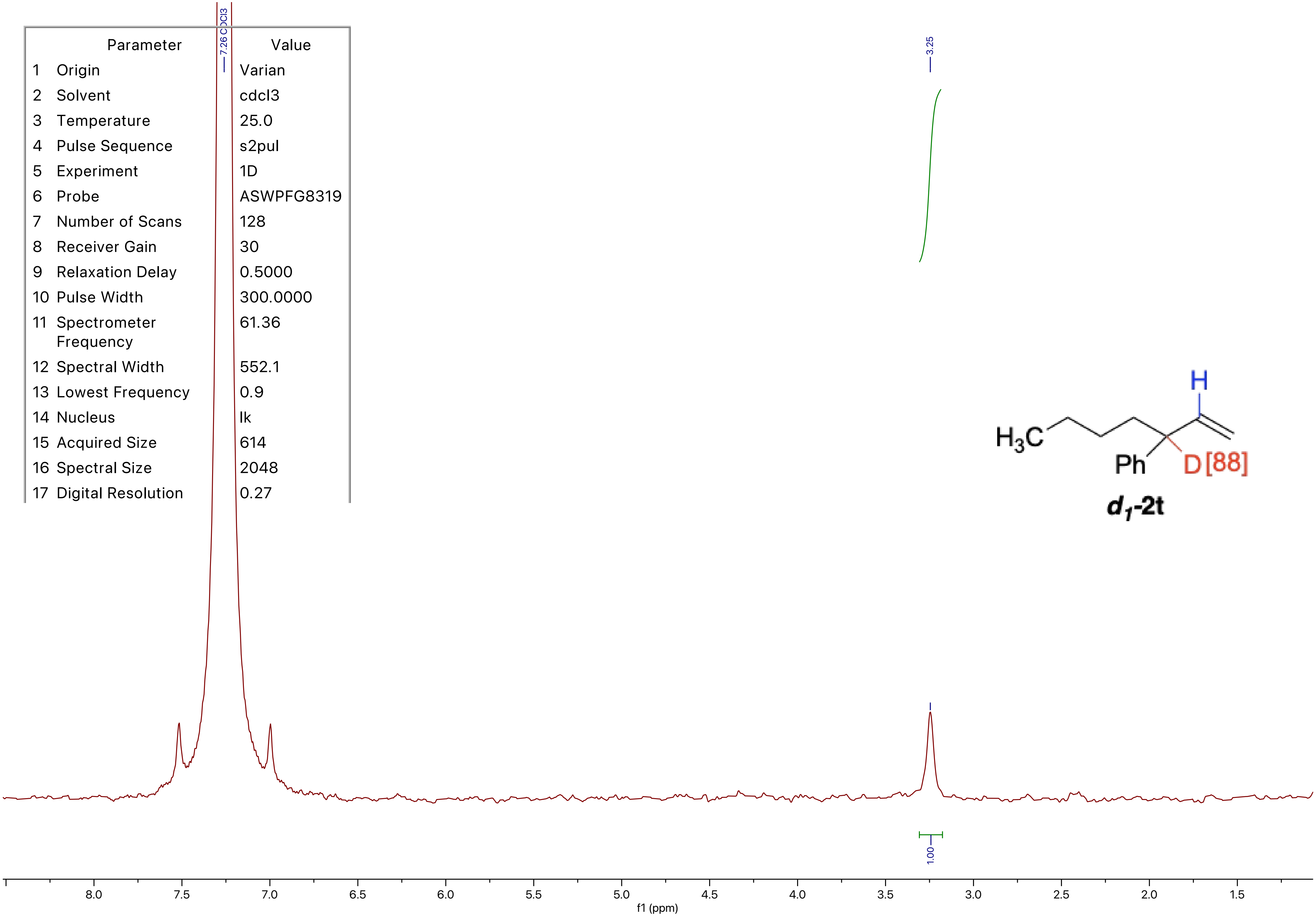

***d*$_1$-2t**

	Parameter	Value
1	Origin	Varian
2	Solvent	cdcl3
3	Temperature	25.0
4	Pulse Sequence	s2pul
5	Experiment	1D
6	Probe	ASWPFG8319
7	Number of Scans	10000
8	Receiver Gain	30
9	Relaxation Delay	3.0000
10	Pulse Width	5.7500
11	Spectrometer Frequency	100.52
12	Spectral Width	25000.0
13	Lowest Frequency	-1443.7
14	Nucleus	13C
15	Acquired Size	32768
16	Spectral Size	65536
17	Digital Resolution	0.38

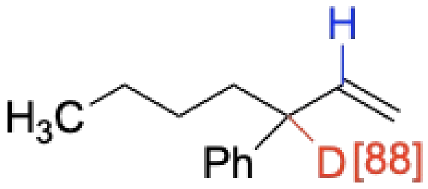

d_1-2t

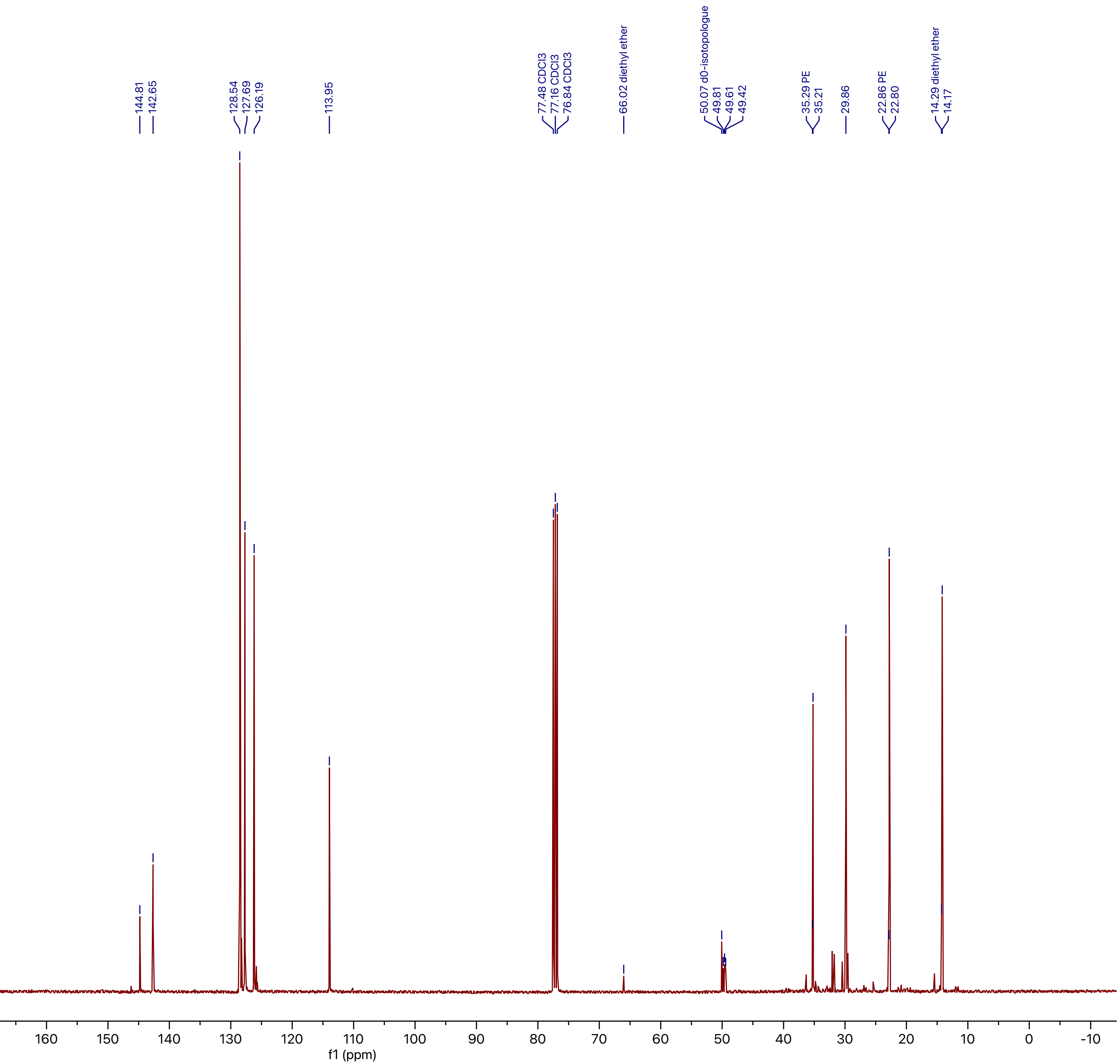

	Parameter	Value
1	Origin	Varian
2	Solvent	cdcl3
3	Temperature	25.0
4	Pulse Sequence	s2pul
5	Experiment	1D
6	Probe	ASWPFG8319
7	Number of Scans	16
8	Receiver Gain	44
9	Relaxation Delay	5.0000
10	Pulse Width	7.7500
11	Spectrometer Frequency	399.73
12	Spectral Width	6410.3
13	Lowest Frequency	-806.7
14	Nucleus	1H
15	Acquired Size	16384
16	Spectral Size	65536
17	Digital Resolution	0.10

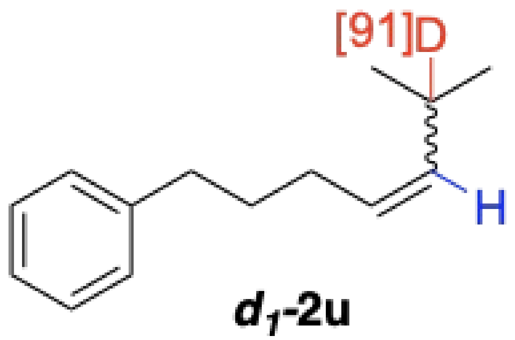

d$_1$-**2u**

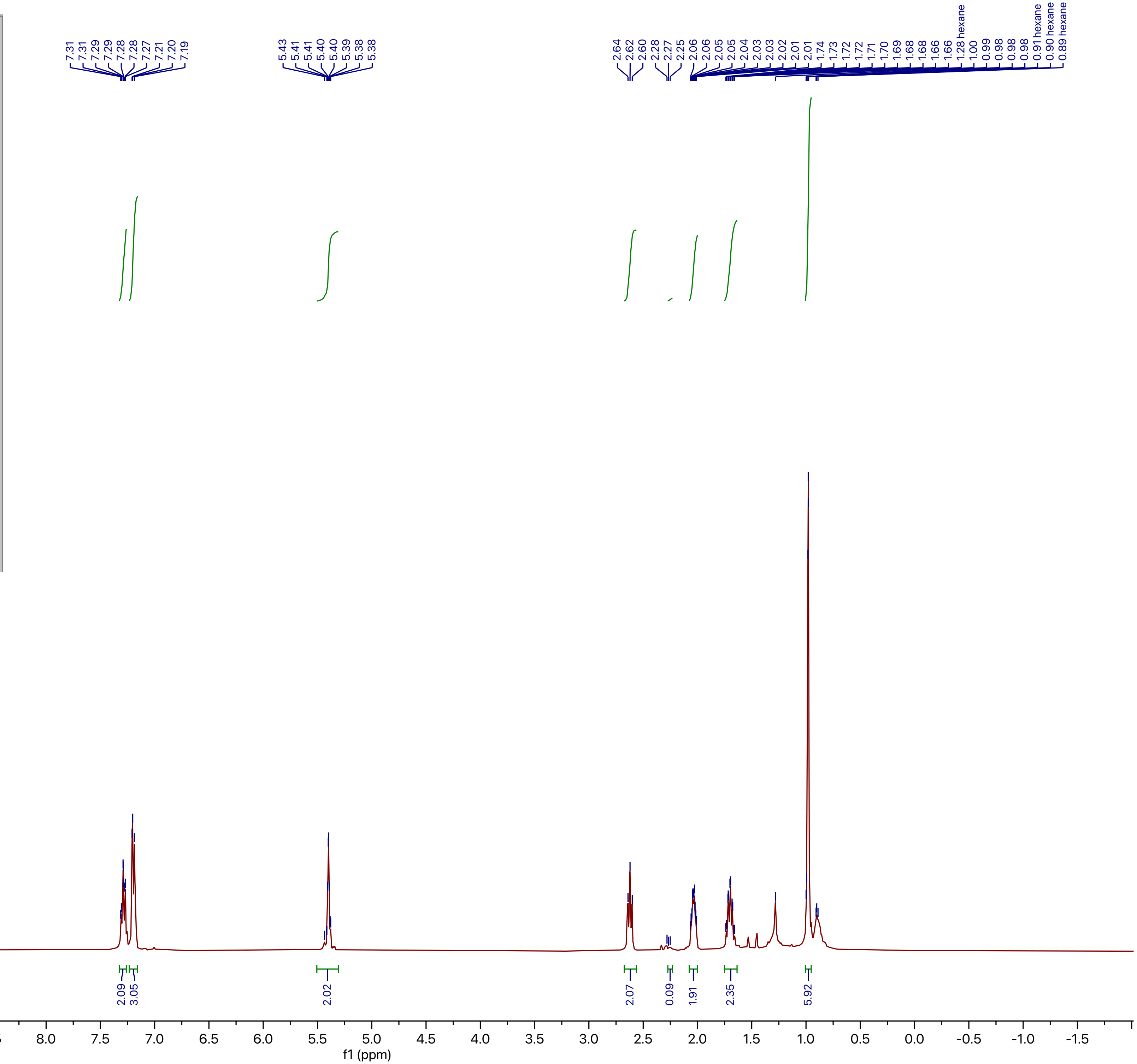

	Parameter	Value
1	Origin	Varian
2	Solvent	cdcl3
3	Temperature	25.0
4	Pulse Sequence	s2pul
5	Experiment	1D
6	Probe	ASWPFG8319
7	Number of Scans	128
8	Receiver Gain	30
9	Relaxation Delay	0.5000
10	Pulse Width	300.0000
11	Spectrometer Frequency	61.36
12	Spectral Width	552.1
13	Lowest Frequency	1.4
14	Nucleus	lk
15	Acquired Size	614
16	Spectral Size	2048
17	Digital Resolution	0.27

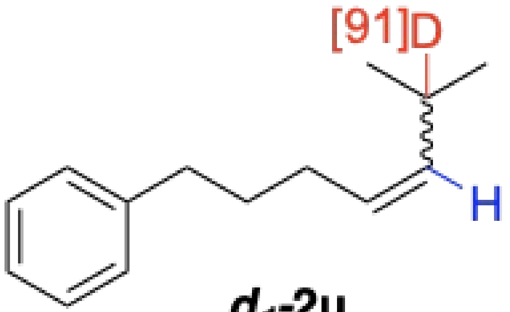

d_1-2u

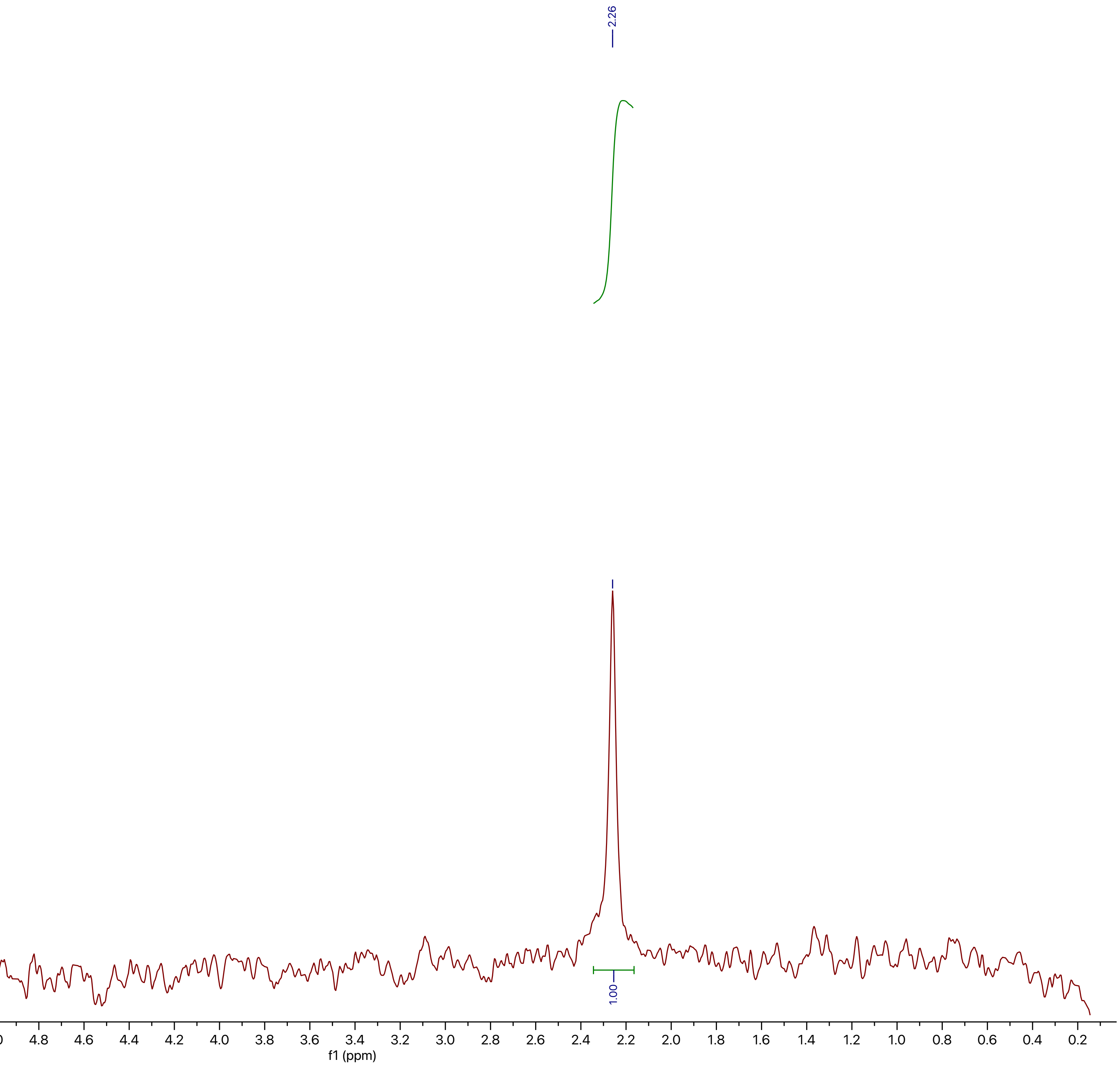

Parameter		Value
1	Origin	Varian
2	Solvent	cdcl3
3	Temperature	25.0
4	Pulse Sequence	s2pul
5	Experiment	1D
6	Probe	ASWPFG8319
7	Number of Scans	512
8	Receiver Gain	30
9	Relaxation Delay	3.0000
10	Pulse Width	5.7500
11	Spectrometer Frequency	100.52
12	Spectral Width	25000.0
13	Lowest Frequency	-1429.9
14	Nucleus	13C
15	Acquired Size	32768
16	Spectral Size	65536
17	Digital Resolution	0.38

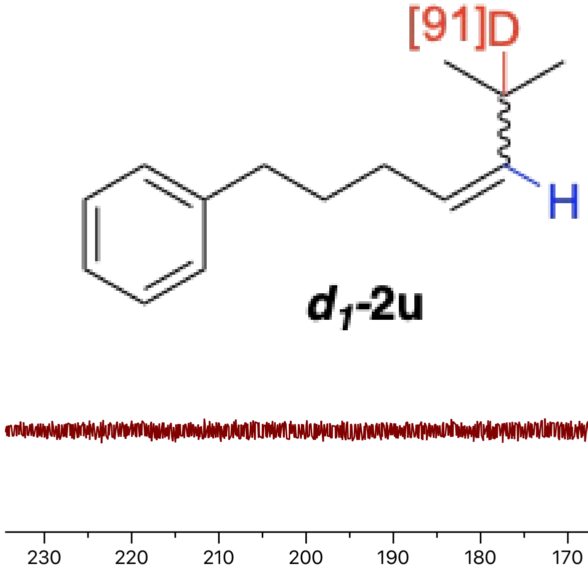

d$_1$-2u

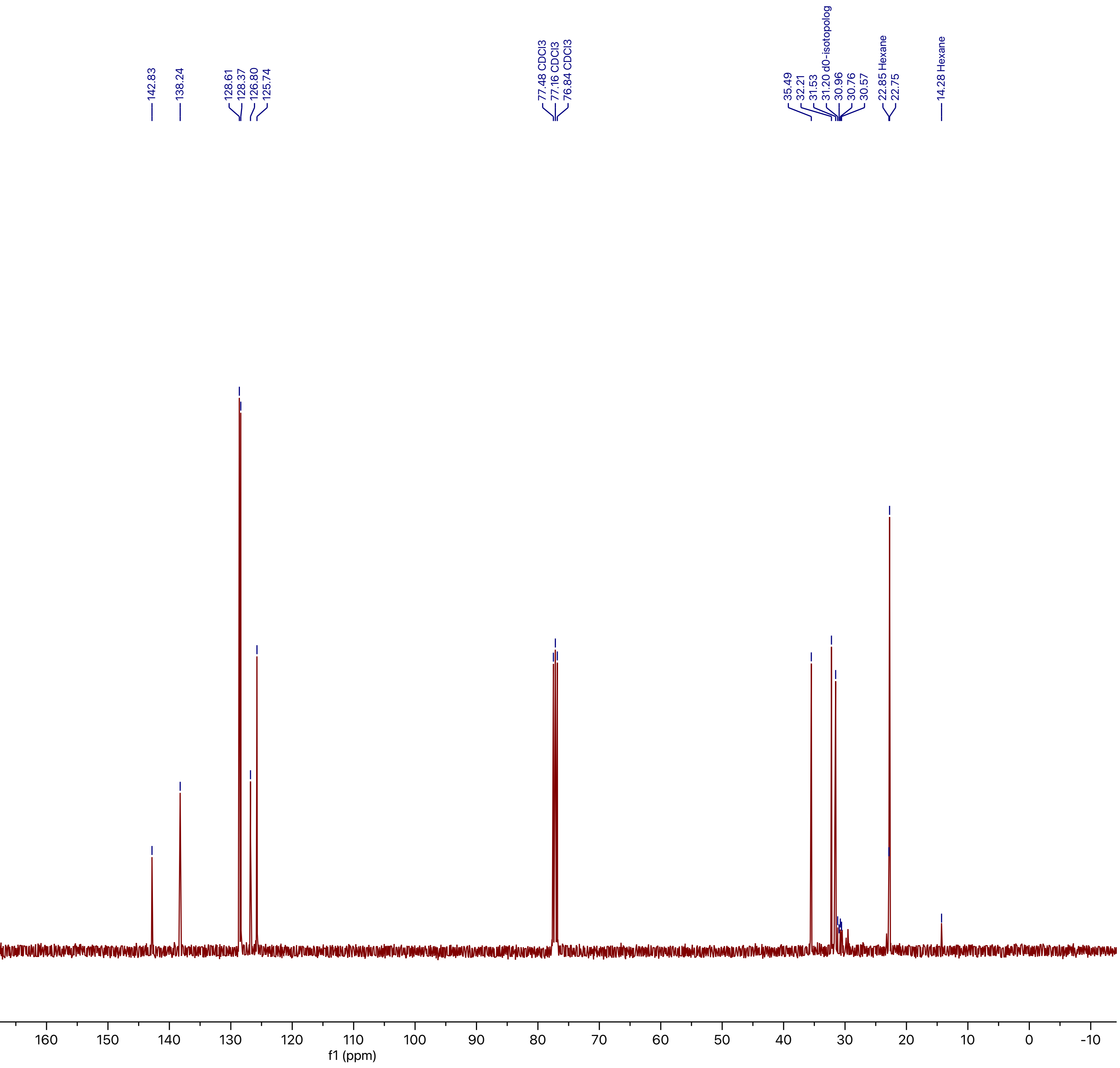

Parameter		Value
1	Origin	Varian
2	Solvent	cdcl3
3	Temperature	25.0
4	Pulse Sequence	s2pul
5	Experiment	1D
6	Probe	ASWPFG8319
7	Number of Scans	16
8	Receiver Gain	38
9	Relaxation Delay	10.0000
10	Pulse Width	7.7500
11	Spectrometer Frequency	399.73
12	Spectral Width	6410.3
13	Lowest Frequency	-806.7
14	Nucleus	1H
15	Acquired Size	16384
16	Spectral Size	65536
17	Digital Resolution	0.10

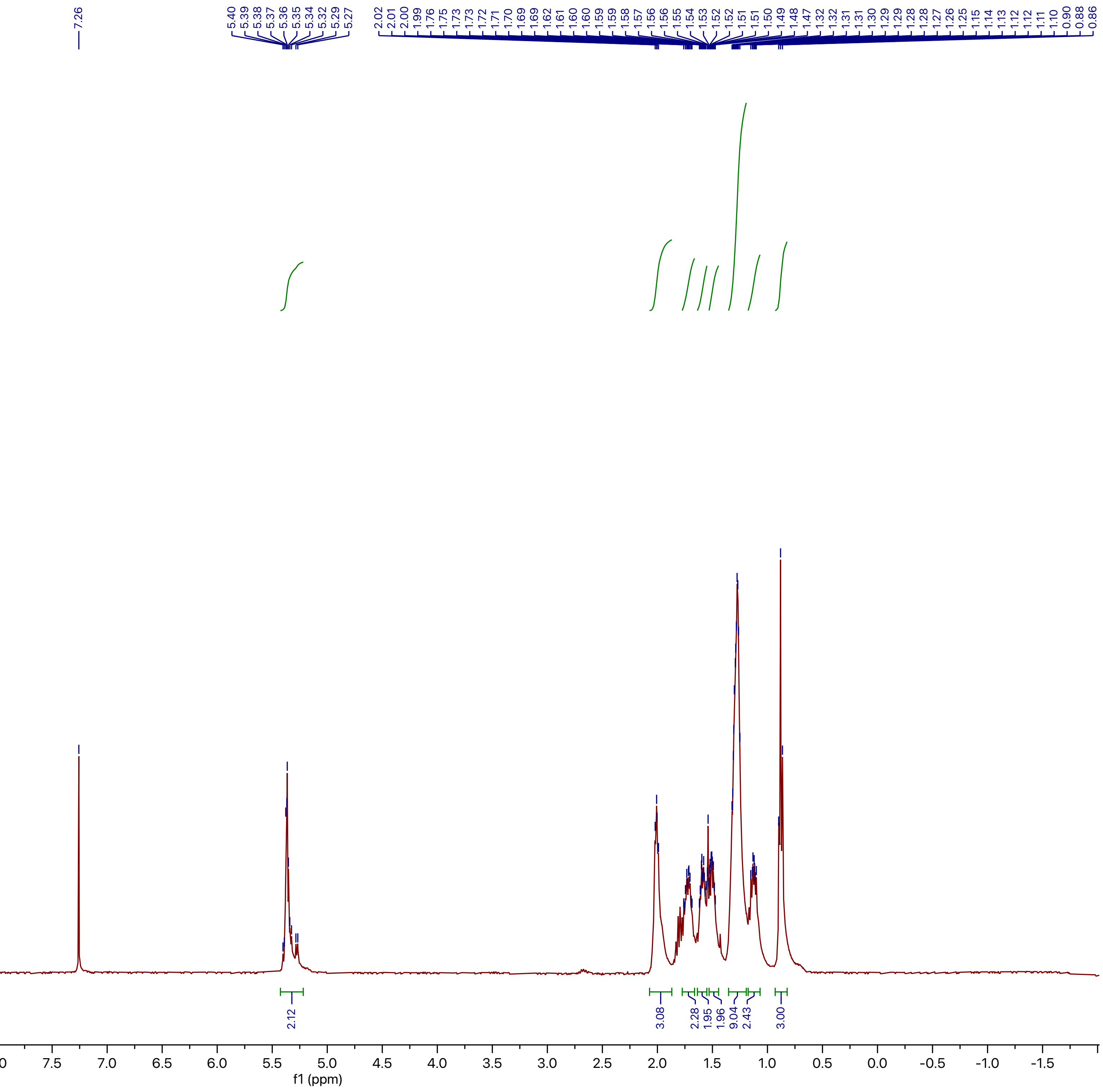

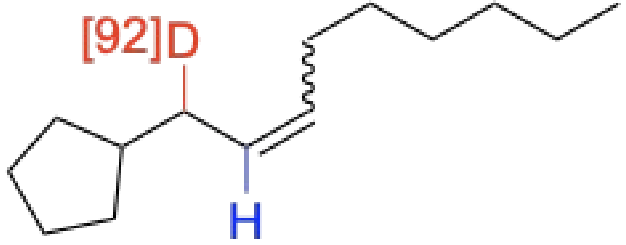

d$_1$-**2v**

	Parameter	Value
1	Origin	Varian
2	Solvent	cdcl3
3	Temperature	25.0
4	Pulse Sequence	s2pul
5	Experiment	1D
6	Probe	OneNMR_W036
7	Number of Scans	32
8	Receiver Gain	20
9	Relaxation Delay	5.0000
10	Pulse Width	300.0000
11	Spectrometer Frequency	76.71
12	Spectral Width	1535.6
13	Lowest Frequency	-382.3
14	Nucleus	lk
15	Acquired Size	2048
16	Spectral Size	4096
17	Digital Resolution	0.37

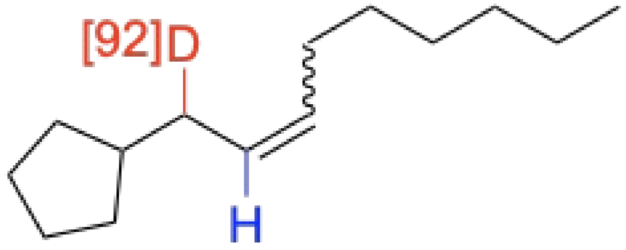

d_1-2v

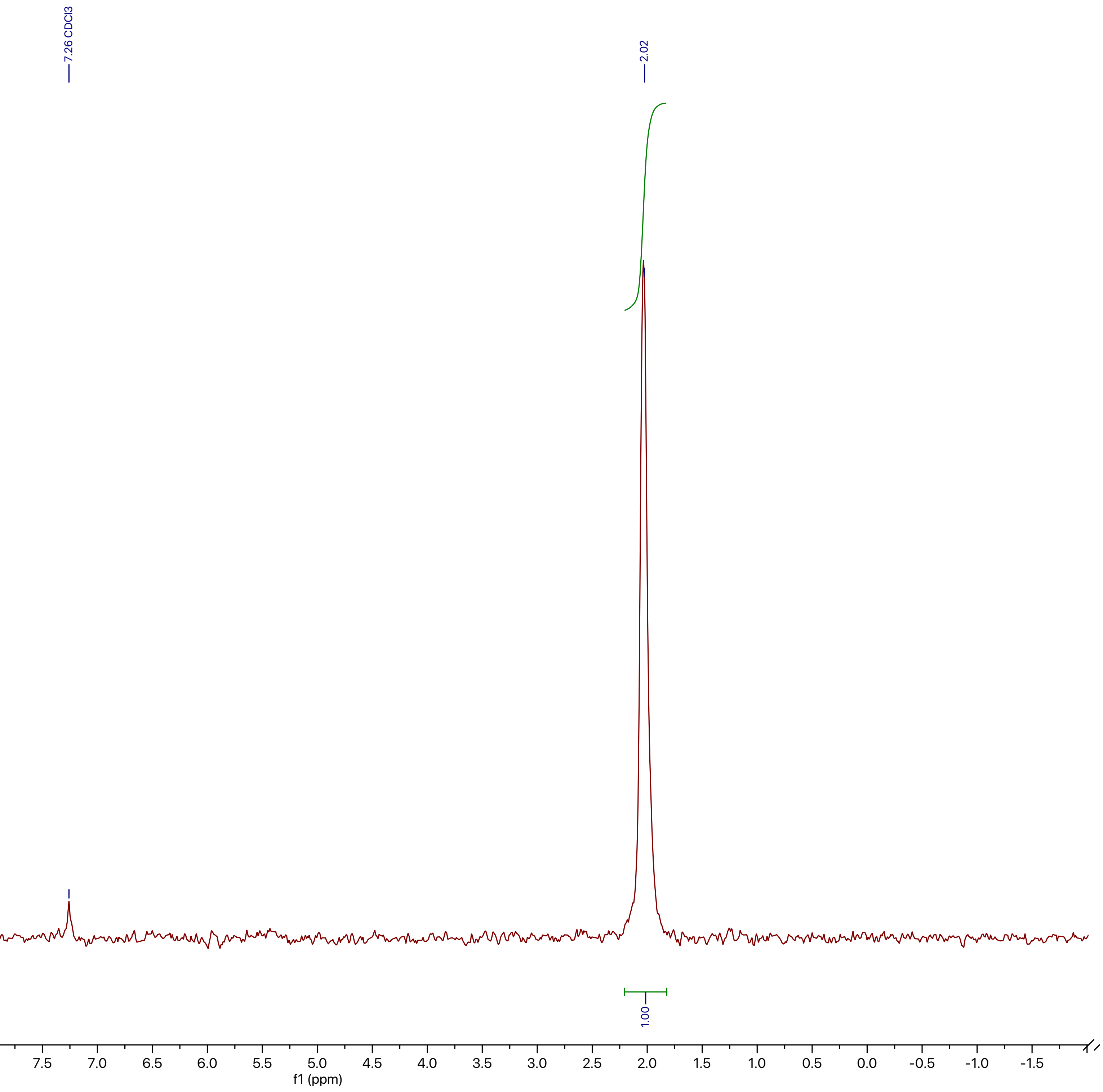

	Parameter	Value
1	Origin	Varian
2	Solvent	cdcl3
3	Temperature	25.0
4	Pulse Sequence	s2pul
5	Experiment	1D
6	Probe	ASWPFG8319
7	Number of Scans	512
8	Receiver Gain	30
9	Relaxation Delay	2.0000
10	Pulse Width	5.7500
11	Spectrometer Frequency	100.52
12	Spectral Width	25000.0
13	Lowest Frequency	-1443.7
14	Nucleus	13C
15	Acquired Size	32768
16	Spectral Size	65536
17	Digital Resolution	0.38

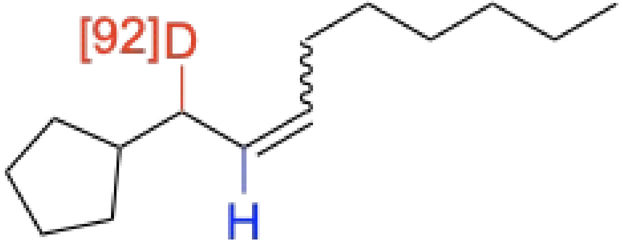

d$_1$-2v

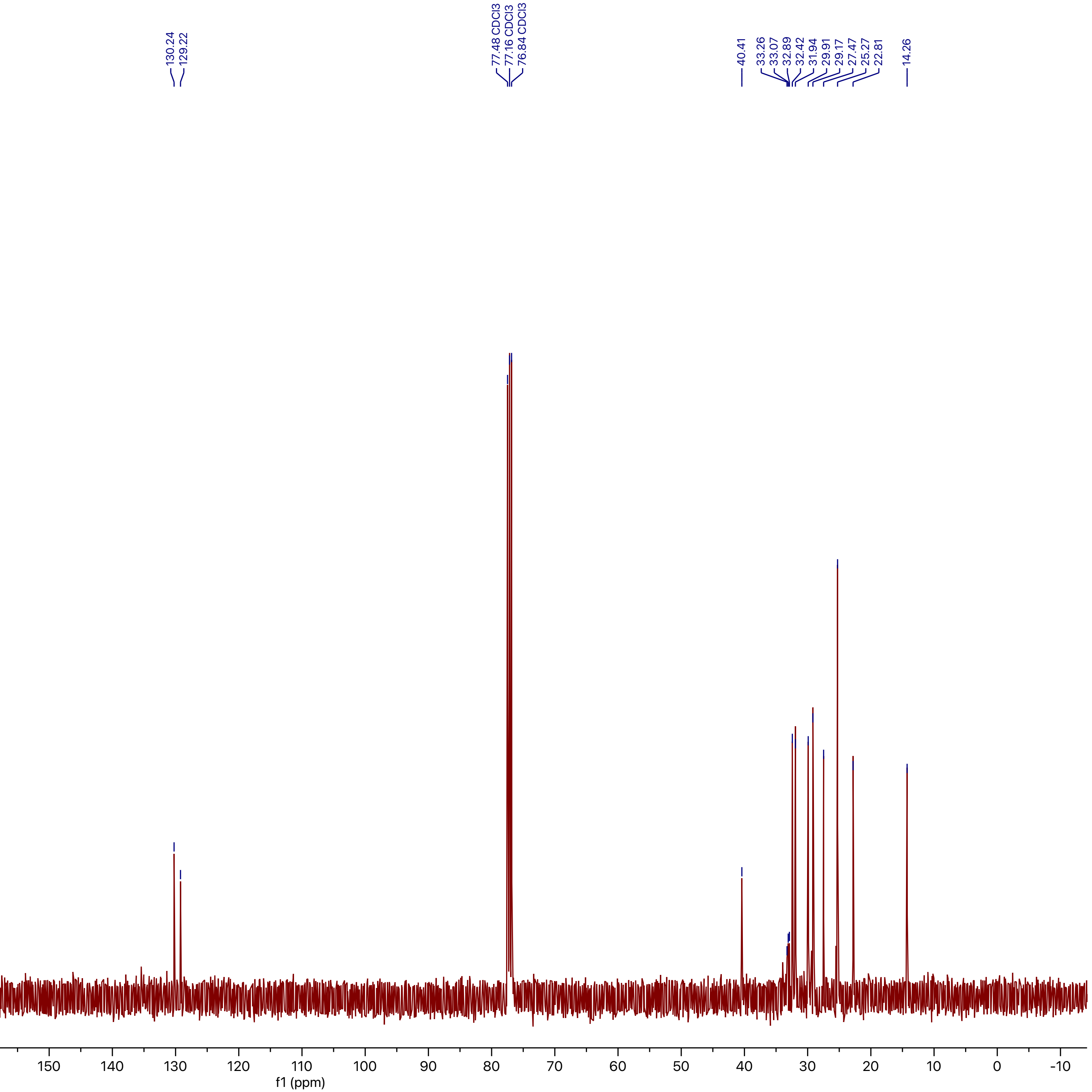

	Parameter	Value
1	Origin	Bruker BioSpin GmbH
2	Instrument	Avance
3	Solvent	CDCl3
4	Temperature	300.0
5	Pulse Sequence	zg30
6	Experiment	1D
7	Probe	Z151574_0073 (PI HR-BBO500S1-BBF/ H/ D-5.0-Z SP)
8	Number of Scans	16
9	Receiver Gain	61.2
10	Relaxation Delay	1.0000
11	Pulse Width	8.0000
12	Spectrometer Frequency	500.21
13	Spectral Width	10000.0
14	Lowest Frequency	-1923.2
15	Nucleus	1H
16	Acquired Size	32768
17	Spectral Size	65536

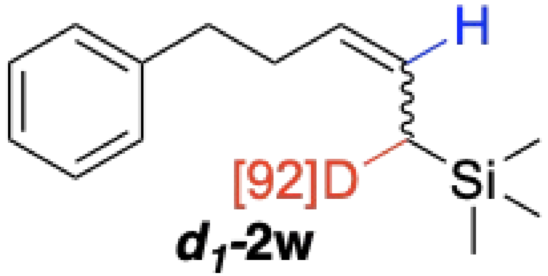

***d*₁-2w**

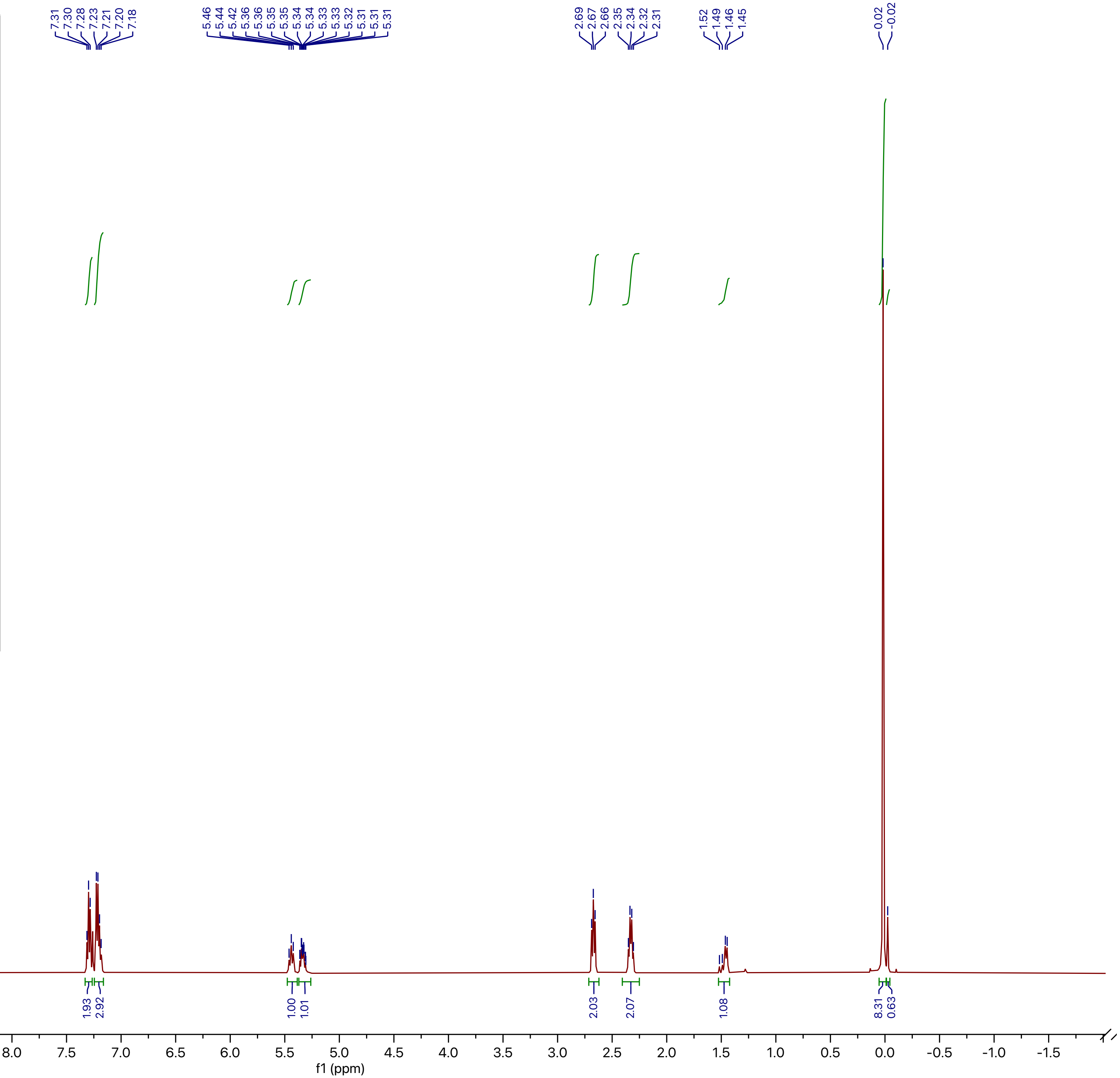

	Parameter	Value
1	Origin	Varian
2	Solvent	cdcl3
3	Temperature	25.0
4	Pulse Sequence	s2pul
5	Experiment	1D
6	Probe	OneNMR_W036
7	Number of Scans	32
8	Receiver Gain	20
9	Relaxation Delay	5.0000
10	Pulse Width	300.0000
11	Spectrometer Frequency	76.71
12	Spectral Width	1535.6
13	Lowest Frequency	-384.2
14	Nucleus	lk
15	Acquired Size	2048
16	Spectral Size	4096
17	Digital Resolution	0.37

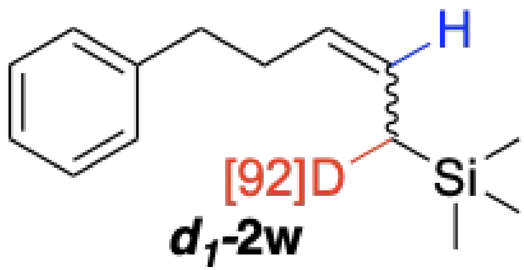

d_1-2w

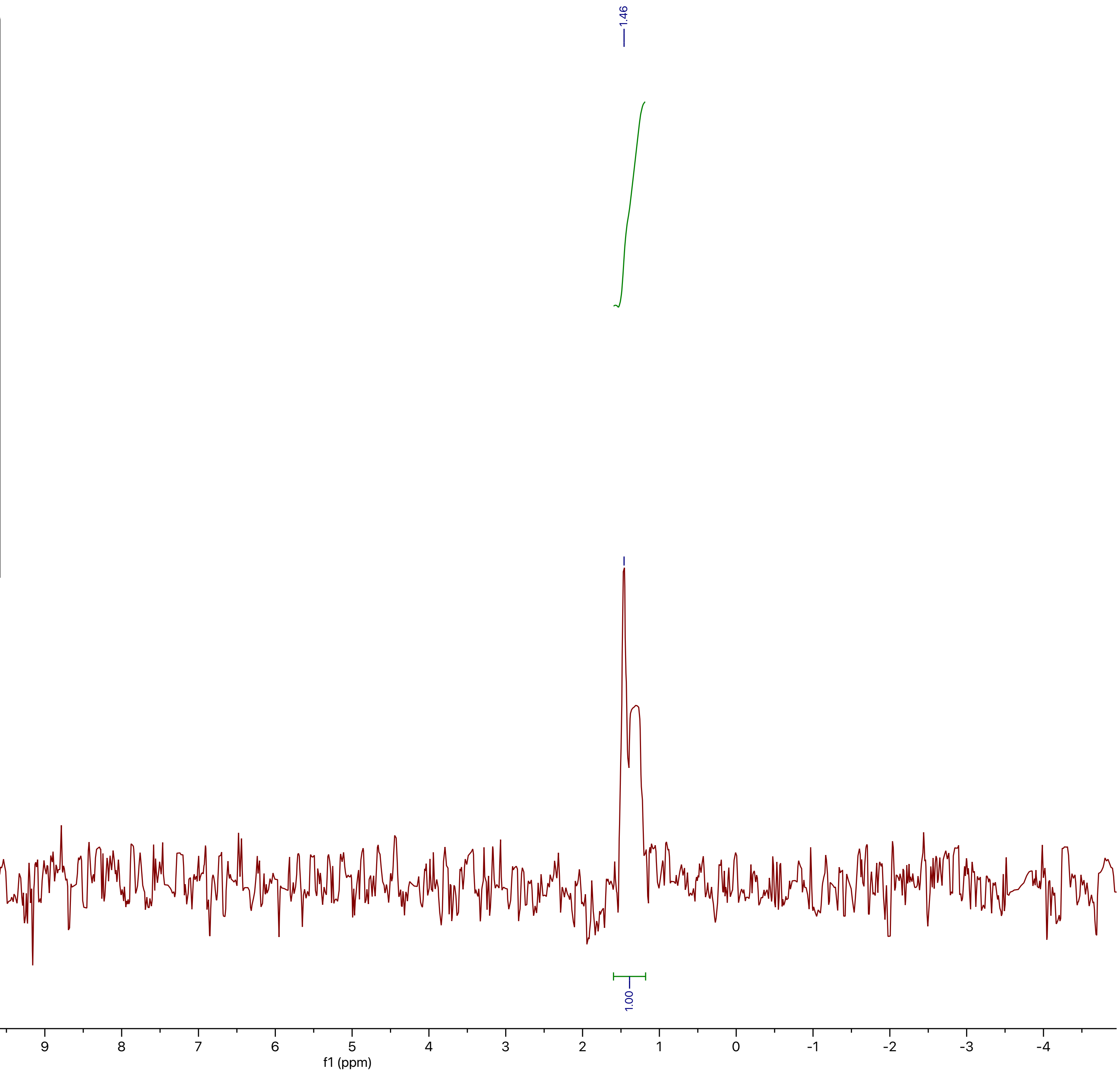

	Parameter	Value
1	Origin	Bruker BioSpin GmbH
2	Instrument	Avance
3	Solvent	CDCl3
4	Temperature	300.0
5	Pulse Sequence	zgpg30
6	Experiment	1D
7	Probe	Z151574_0073 (PI HR-BBO500S1-BBF/ H/ D-5.0-Z SP)
8	Number of Scans	100
9	Receiver Gain	101.0
10	Relaxation Delay	2.0000
11	Pulse Width	9.0000
12	Spectrometer Frequency	125.79
13	Spectral Width	30120.5
14	Lowest Frequency	-2465.7
15	Nucleus	13C
16	Acquired Size	32768
17	Spectral Size	65536

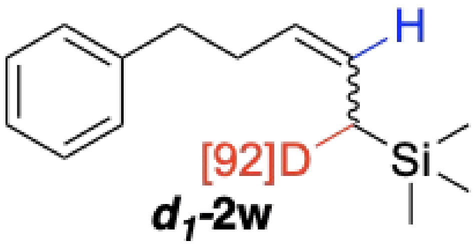

d_1-2w

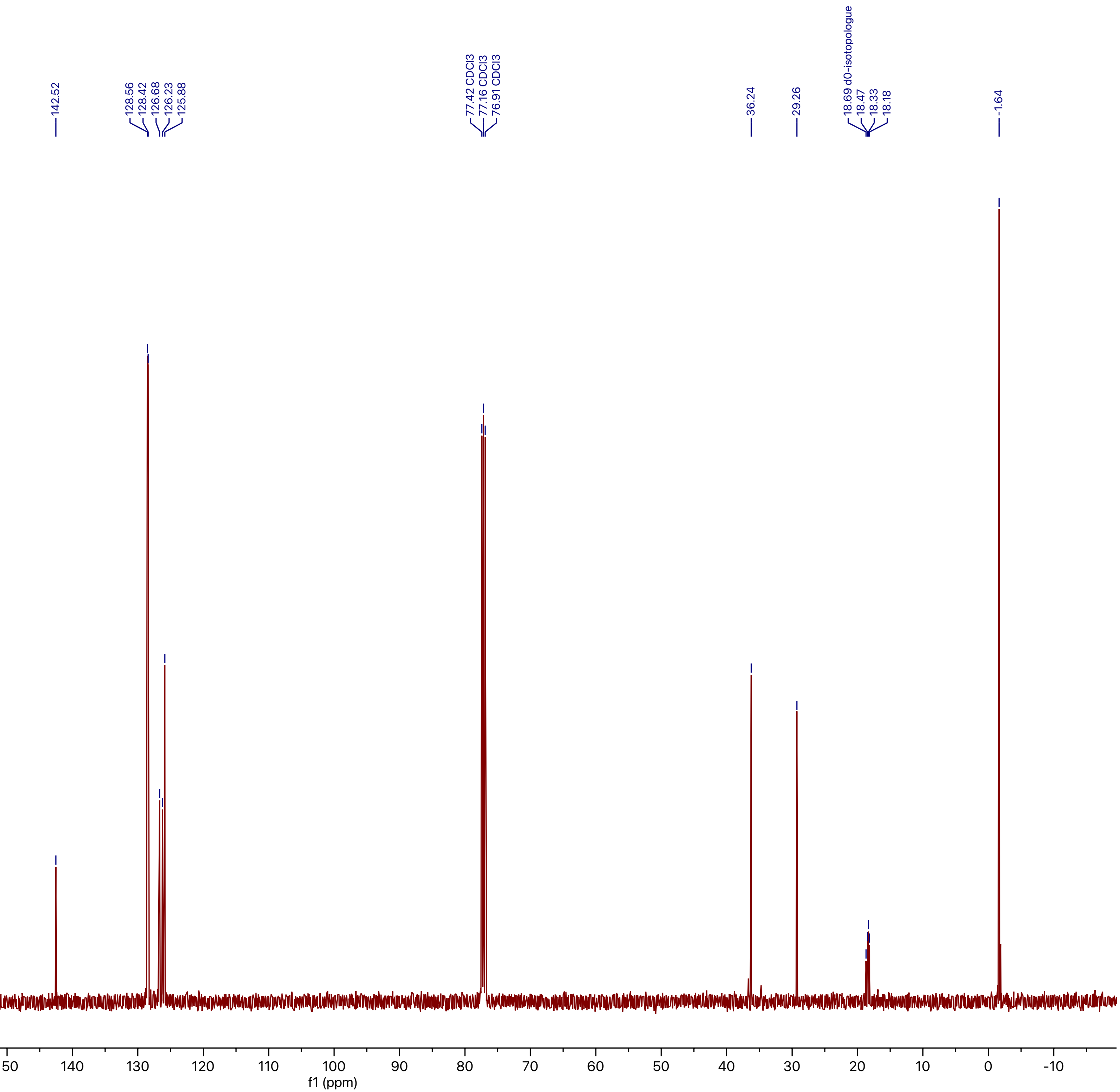

	Parameter	Value
1	Origin	Bruker BioSpin GmbH
2	Instrument	Avance
3	Solvent	CDCl3
4	Temperature	300.0
5	Pulse Sequence	zg30
6	Experiment	1D
7	Probe	Z151574_0073 (PI HR-BBO500S1-BBF/ H/ D-5.0-Z SP)
8	Number of Scans	16
9	Receiver Gain	70.7
10	Relaxation Delay	1.0000
11	Pulse Width	8.0000
12	Spectrometer Frequency	500.21
13	Spectral Width	10000.0
14	Lowest Frequency	-1907.9
15	Nucleus	1H
16	Acquired Size	32768
17	Spectral Size	65536

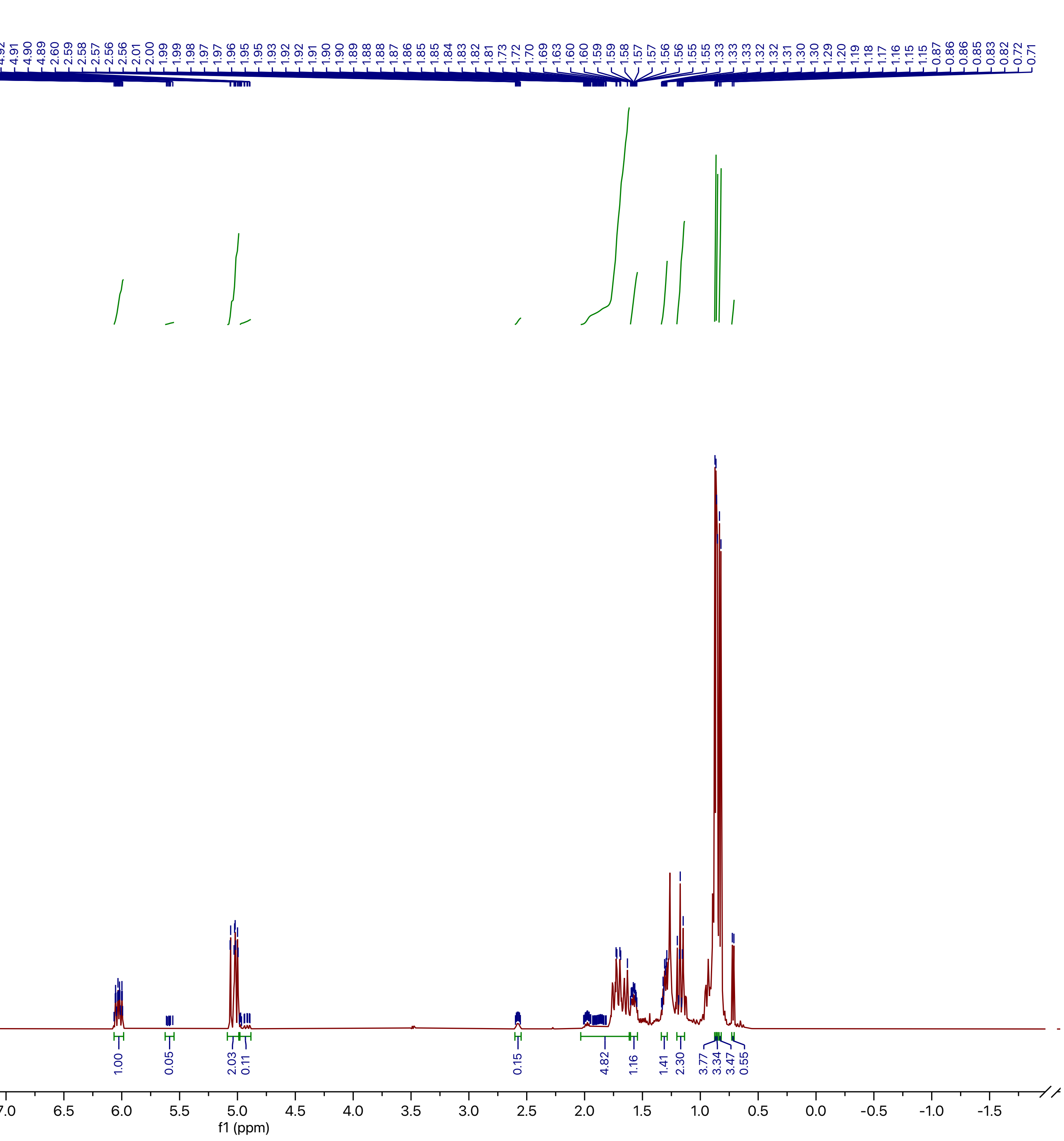

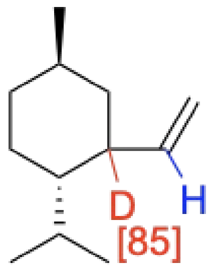

20:1 d.r.

d*$_1$*-2x

	Parameter	Value
1	Origin	Varian
2	Solvent	cdcl3
3	Temperature	25.0
4	Pulse Sequence	s2pul
5	Experiment	1D
6	Probe	OneNMR_W036
7	Number of Scans	32
8	Receiver Gain	20
9	Relaxation Delay	2.0000
10	Pulse Width	300.0000
11	Spectrometer Frequency	76.71
12	Spectral Width	1535.6
13	Lowest Frequency	-384.2
14	Nucleus	lk
15	Acquired Size	2048
16	Spectral Size	4096
17	Digital Resolution	0.37

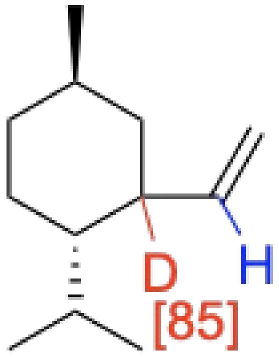

20:1 d.r.

***d*$_1$-2x**

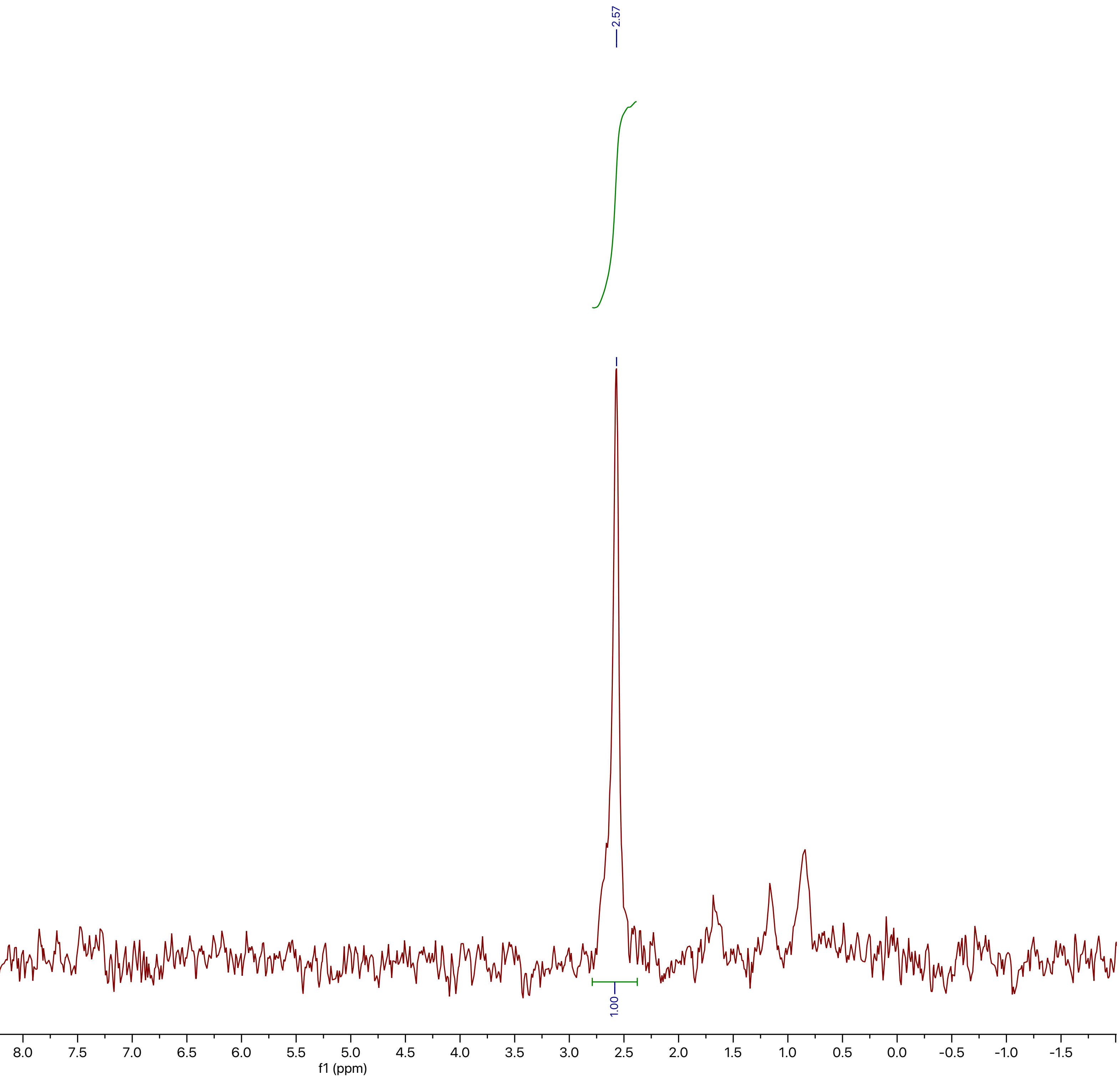

	Parameter	Value
1	Origin	Bruker BioSpin GmbH
2	Instrument	Avance
3	Solvent	CDCl3
4	Temperature	300.0
5	Pulse Sequence	zgpg30
6	Experiment	1D
7	Probe	Z151574_0073 (PI HR-BBO500S1-BBF/ H/ D-5.0-Z SP)
8	Number of Scans	512
9	Receiver Gain	101.0
10	Relaxation Delay	2.0000
11	Pulse Width	9.0000
12	Spectrometer Frequency	125.79
13	Spectral Width	30120.5
14	Lowest Frequency	-2462.5
15	Nucleus	13C
16	Acquired Size	32768
17	Spectral Size	65536

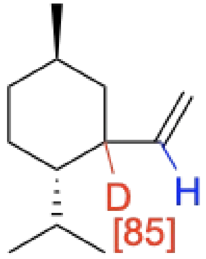

20:1 d.r.

***d*₁-2x**

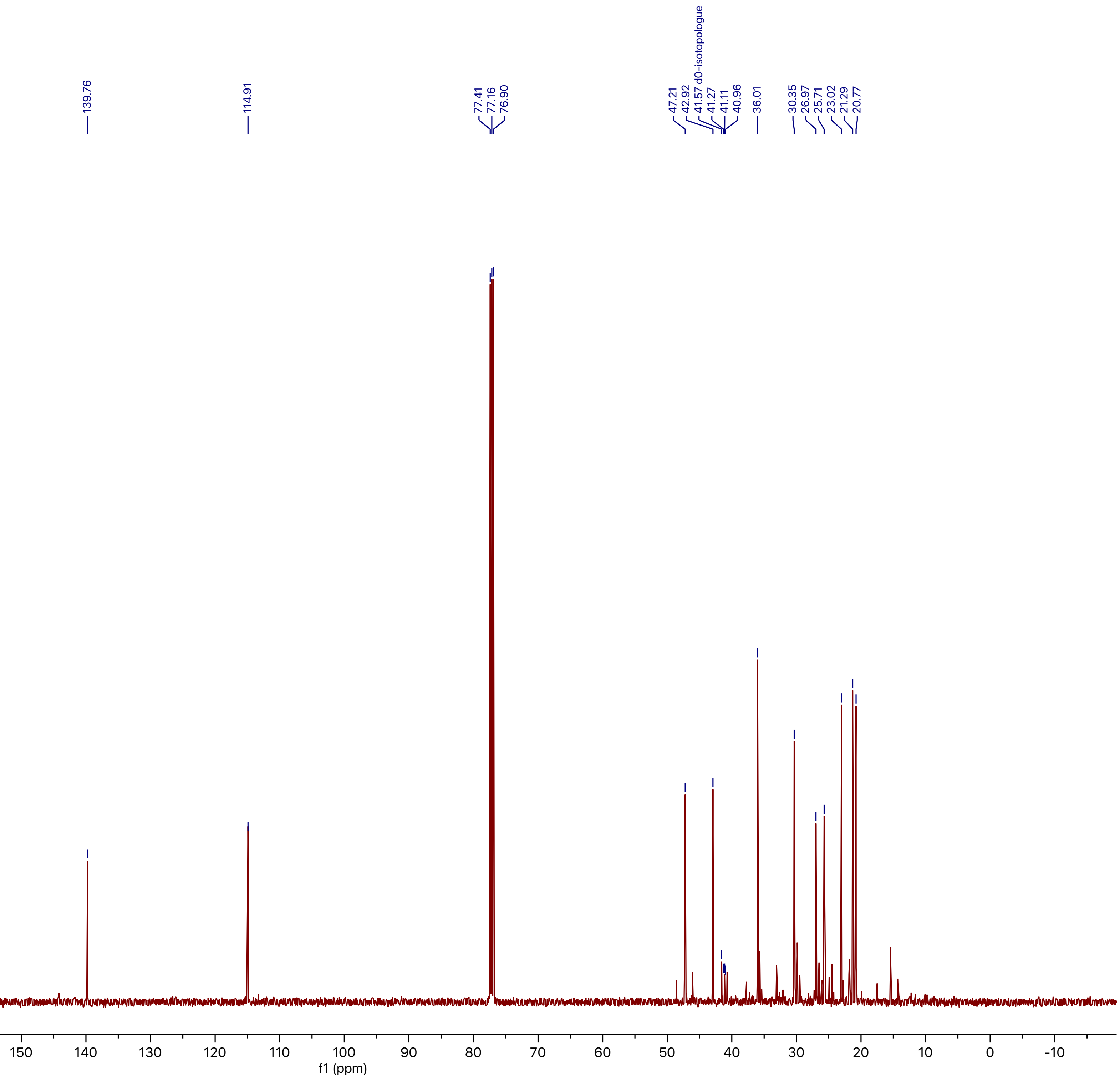

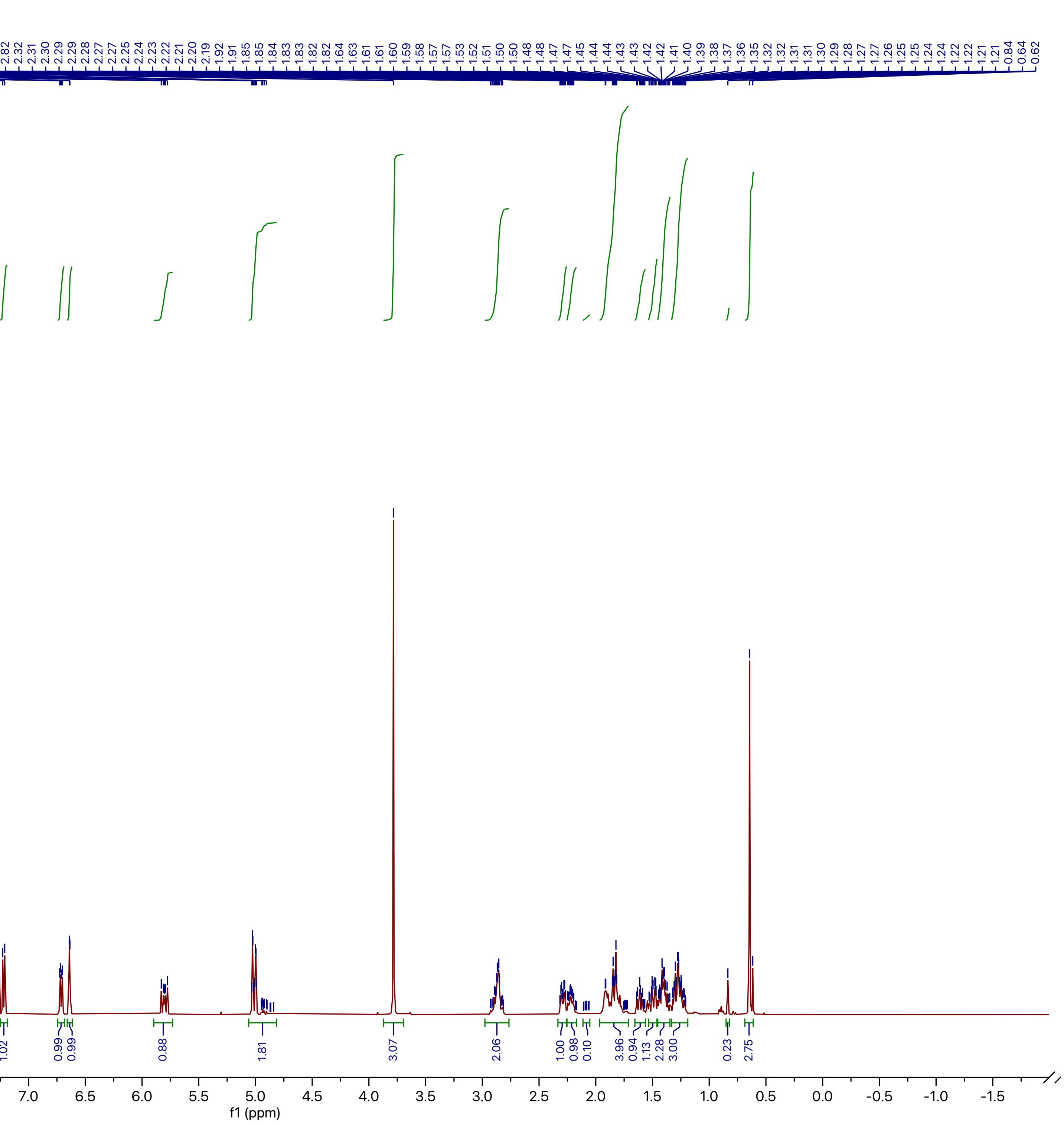

	Parameter	Value
1	Origin	Bruker BioSpin GmbH
2	Instrument	Avance
3	Solvent	CDCl3
4	Temperature	300.0
5	Pulse Sequence	zg30
6	Experiment	1D
7	Probe	Z151574_0073 (PI HR-BBO500S1-BBF/ H/ D-5.0-Z SP)
8	Number of Scans	64
9	Receiver Gain	101.0
10	Relaxation Delay	1.0000
11	Pulse Width	8.0000
12	Spectrometer Frequency	500.21
13	Spectral Width	10000.0
14	Lowest Frequency	-1923.5
15	Nucleus	1H
16	Acquired Size	32768
17	Spectral Size	65536

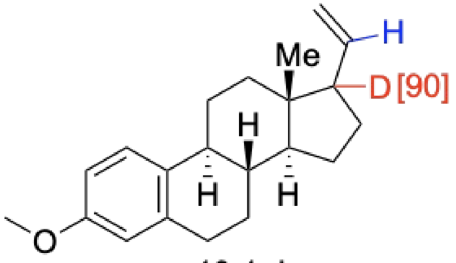

10:1 d.r.

d*$_1$*-2y

	Parameter	Value
1	Origin	Varian
2	Solvent	cdcl3
3	Temperature	25.0
4	Pulse Sequence	s2pul
5	Experiment	1D
6	Probe	OneNMR_W036
7	Number of Scans	32
8	Receiver Gain	20
9	Relaxation Delay	3.0000
10	Pulse Width	300.0000
11	Spectrometer Frequency	76.71
12	Spectral Width	1535.6
13	Lowest Frequency	-382.1
14	Nucleus	lk
15	Acquired Size	2048
16	Spectral Size	4096
17	Digital Resolution	0.37

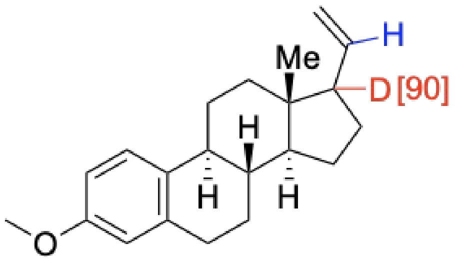

10:1 d.r.

***d_1*-2y**

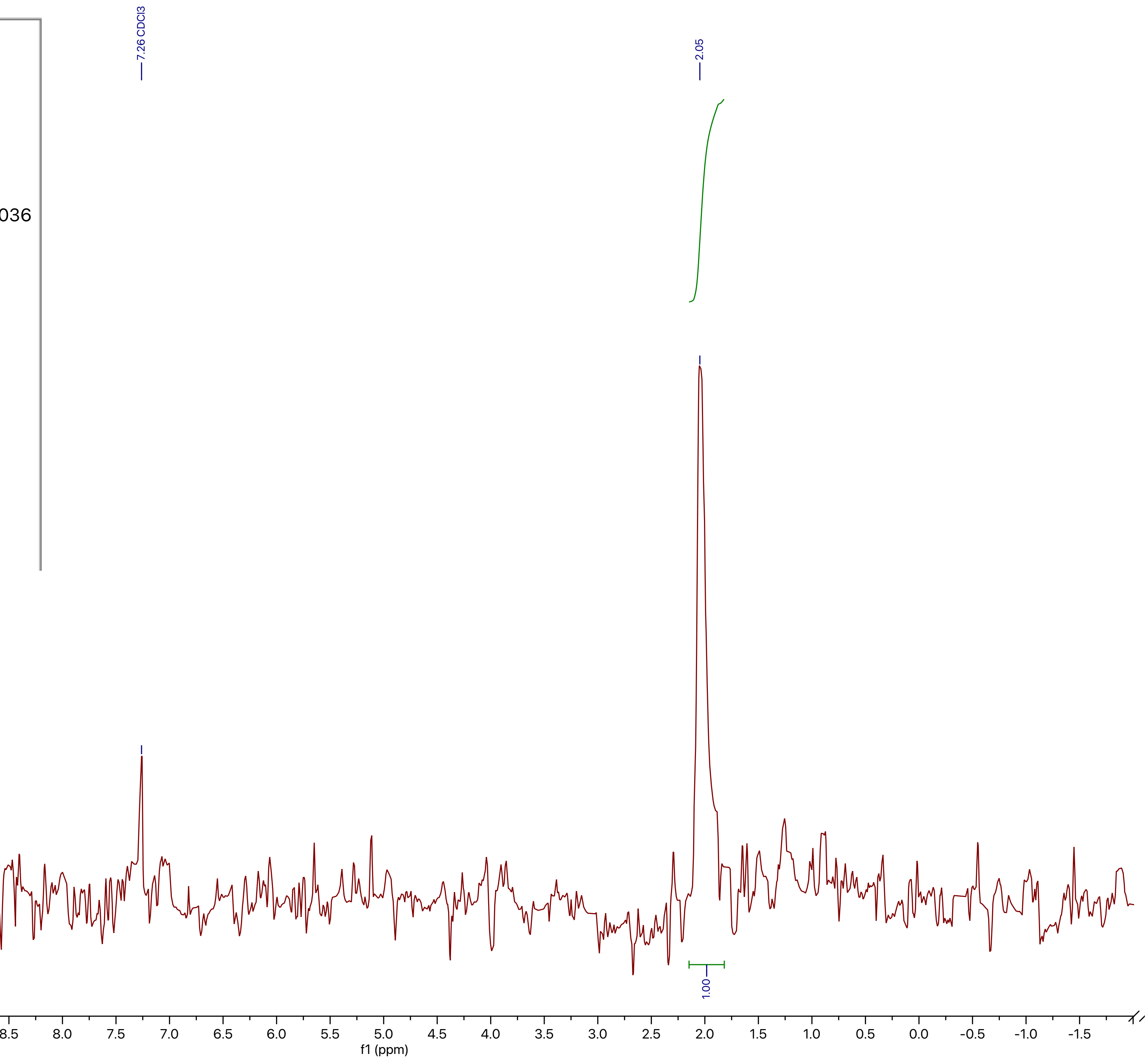

	Parameter	Value
1	Origin	Bruker BioSpin GmbH
2	Instrument	Avance
3	Solvent	CDCl3
4	Temperature	300.0
5	Pulse Sequence	zgpg30
6	Experiment	1D
7	Probe	Z151574_0073 (PI HR-BBO500S1-BBF/ H/ D-5.0-Z SP)
8	Number of Scans	5000
9	Receiver Gain	101.0
10	Relaxation Delay	5.0000
11	Pulse Width	9.0000
12	Spectrometer Frequency	125.79
13	Spectral Width	30120.5
14	Lowest Frequency	-2465.7
15	Nucleus	13C
16	Acquired Size	32768
17	Spectral Size	65536

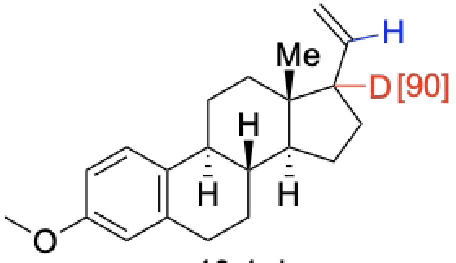

10:1 d.r.

***d*₁-2y**

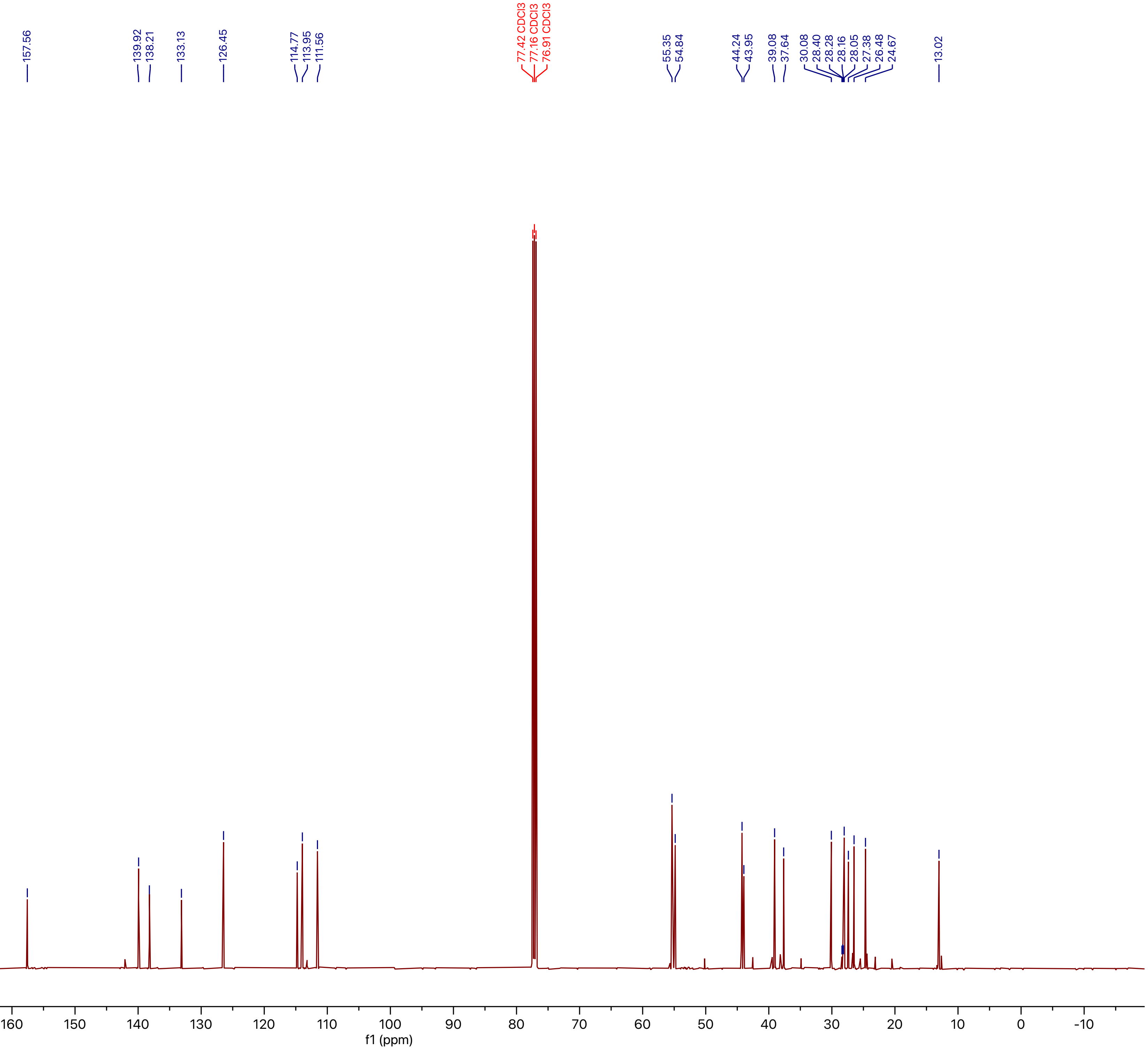

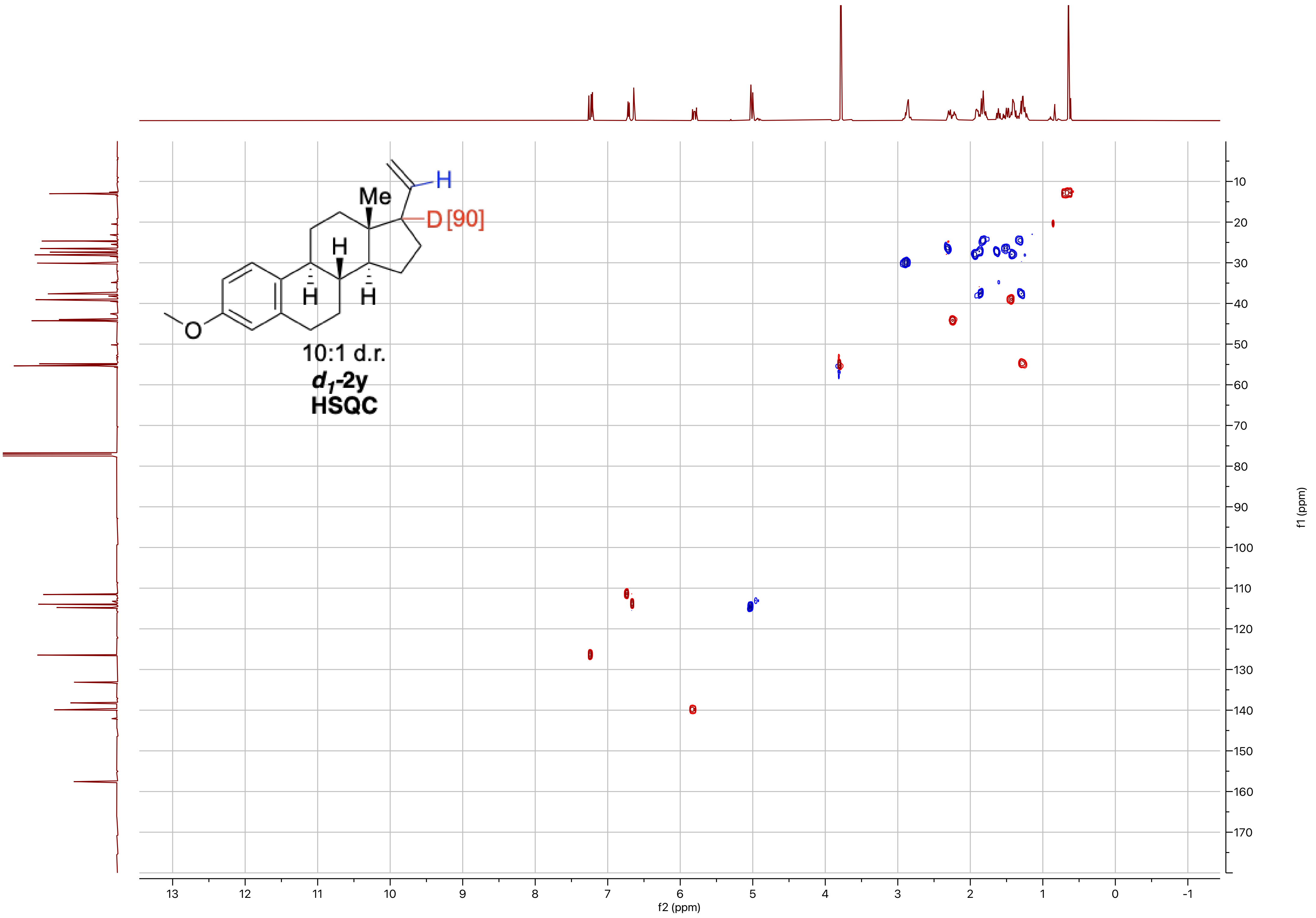

Me
H
D [90]
H
H
H
O
10:1 d.r.
d1-2y
HSQC
f2 (ppm)
f1 (ppm)

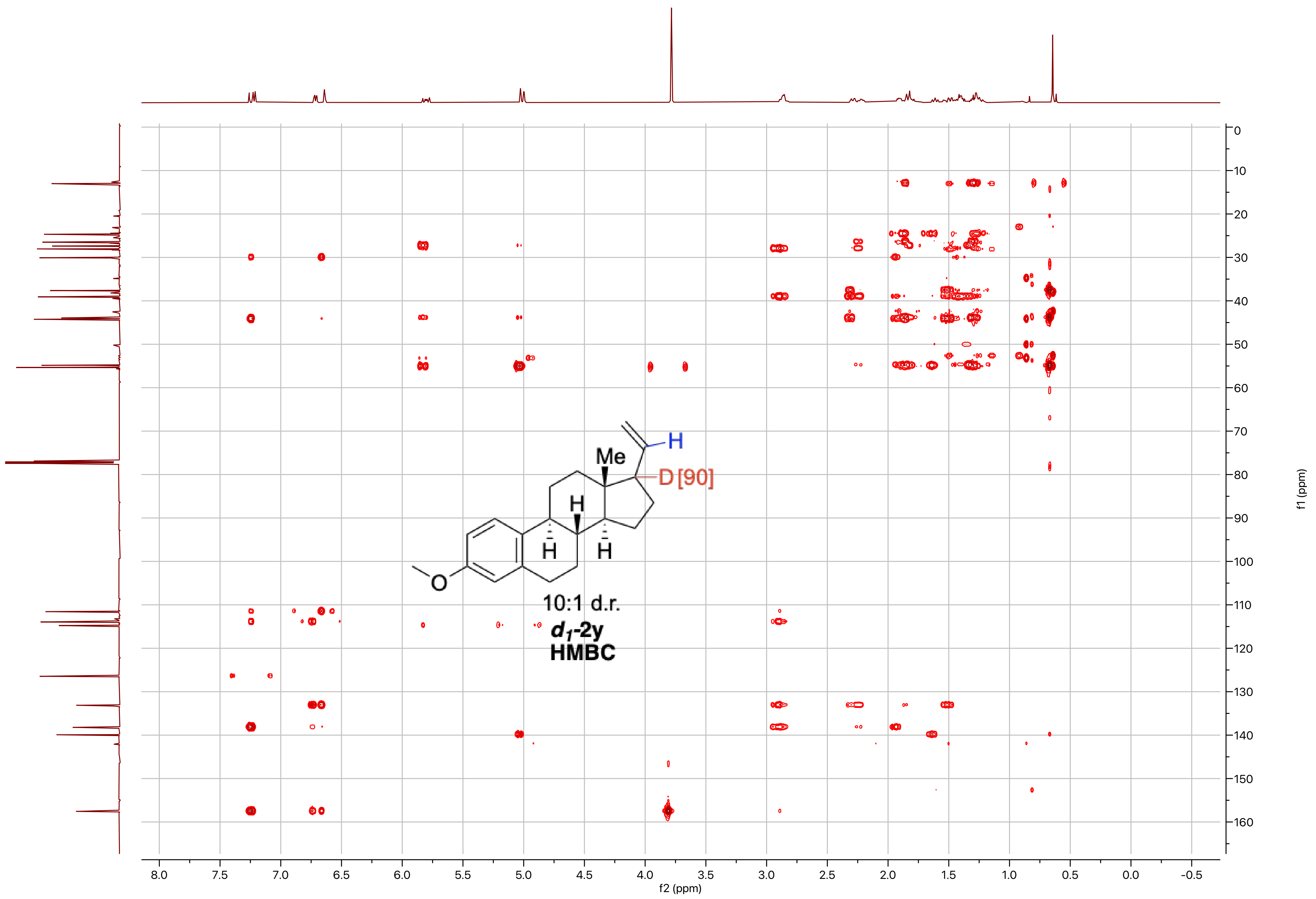

Me
H
D [90]
H
H
H
O
10:1 d.r.
d₁-2y
HMBC
f2 (ppm)
f1 (ppm)

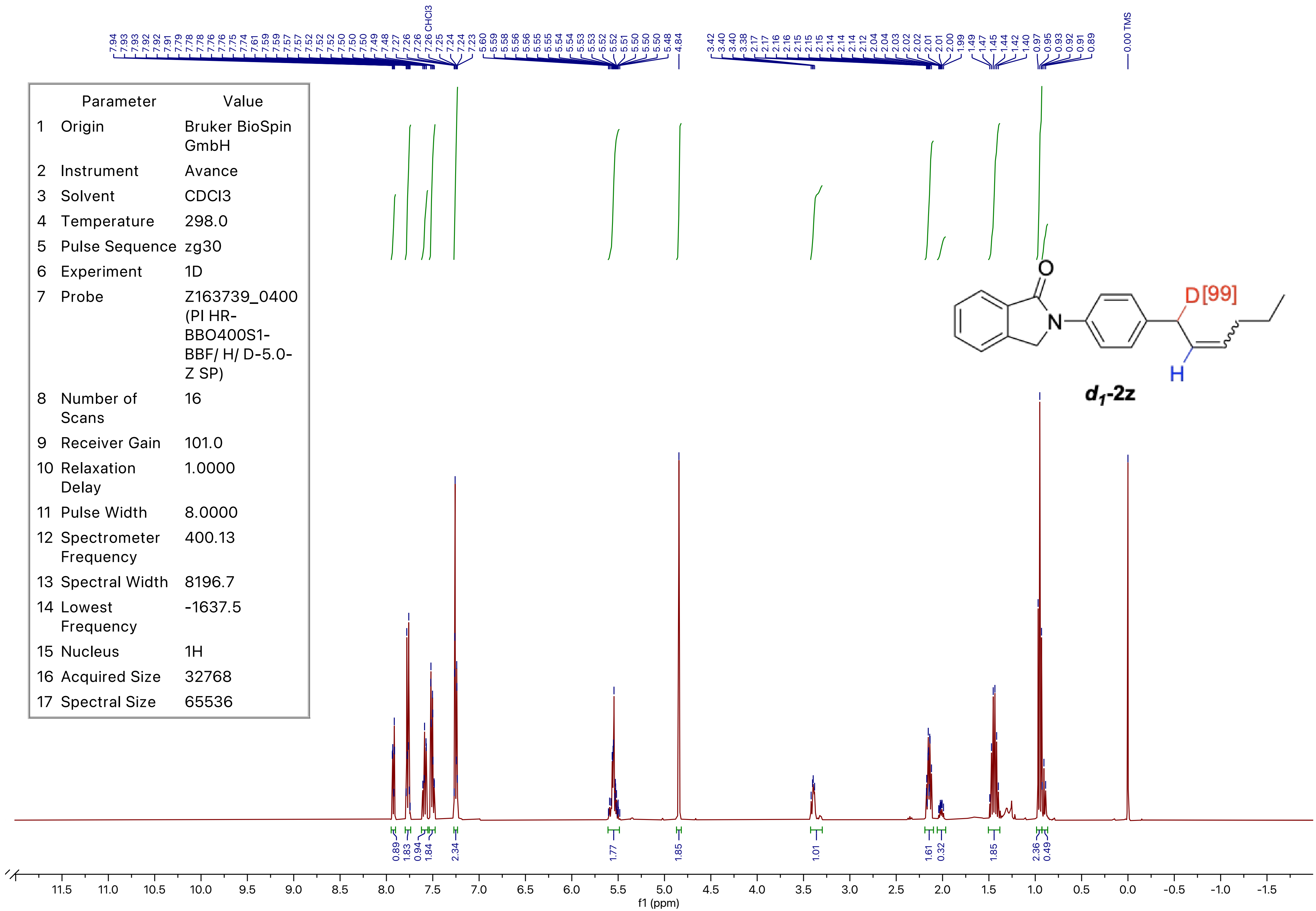

	Parameter	Value
1	Origin	Bruker BioSpin GmbH
2	Instrument	Avance
3	Solvent	CDCl3
4	Temperature	298.0
5	Pulse Sequence	zg30
6	Experiment	1D
7	Probe	Z163739_0400 (PI HR-BBO400S1-BBF/ H/ D-5.0-Z SP)
8	Number of Scans	16
9	Receiver Gain	101.0
10	Relaxation Delay	1.0000
11	Pulse Width	8.0000
12	Spectrometer Frequency	400.13
13	Spectral Width	8196.7
14	Lowest Frequency	-1637.5
15	Nucleus	1H
16	Acquired Size	32768
17	Spectral Size	65536

	Parameter	Value
1	Origin	Varian
2	Solvent	cdcl3
3	Temperature	25.0
4	Pulse Sequence	s2pul
5	Experiment	1D
6	Probe	ASWPFG8319
7	Number of Scans	256
8	Receiver Gain	30
9	Relaxation Delay	0.5000
10	Pulse Width	300.0000
11	Spectrometer Frequency	61.36
12	Spectral Width	552.1
13	Lowest Frequency	-26.0
14	Nucleus	"lk"
15	Acquired Size	614
16	Spectral Size	2048
17	Digital Resolution	0.27

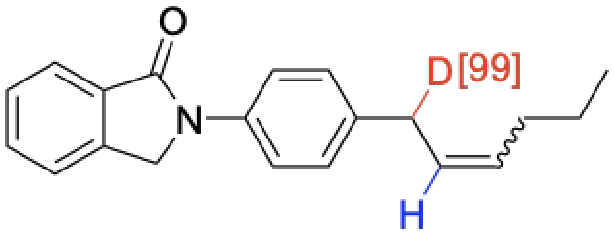

***d*$_1$-2z**

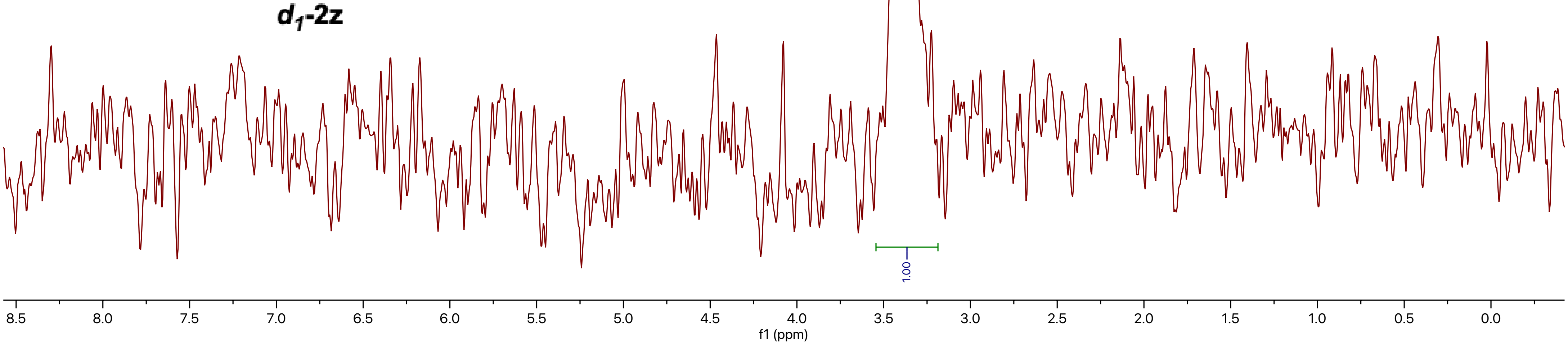

	Parameter	Value
1	Origin	Bruker BioSpin GmbH
2	Instrument	Avance
3	Solvent	CDCl3
4	Temperature	298.0
5	Pulse Sequence	zgpg30
6	Experiment	1D
7	Probe	Z163739_0400 (PI HR-BBO400S1-BBF/ H/ D-5.0-Z SP)
8	Number of Scans	1024
9	Receiver Gain	101.0
10	Relaxation Delay	2.0000
11	Pulse Width	8.0000
12	Spectrometer Frequency	100.62
13	Spectral Width	23809.5
14	Lowest Frequency	-1843.5
15	Nucleus	13C
16	Acquired Size	32768
17	Spectral Size	65536

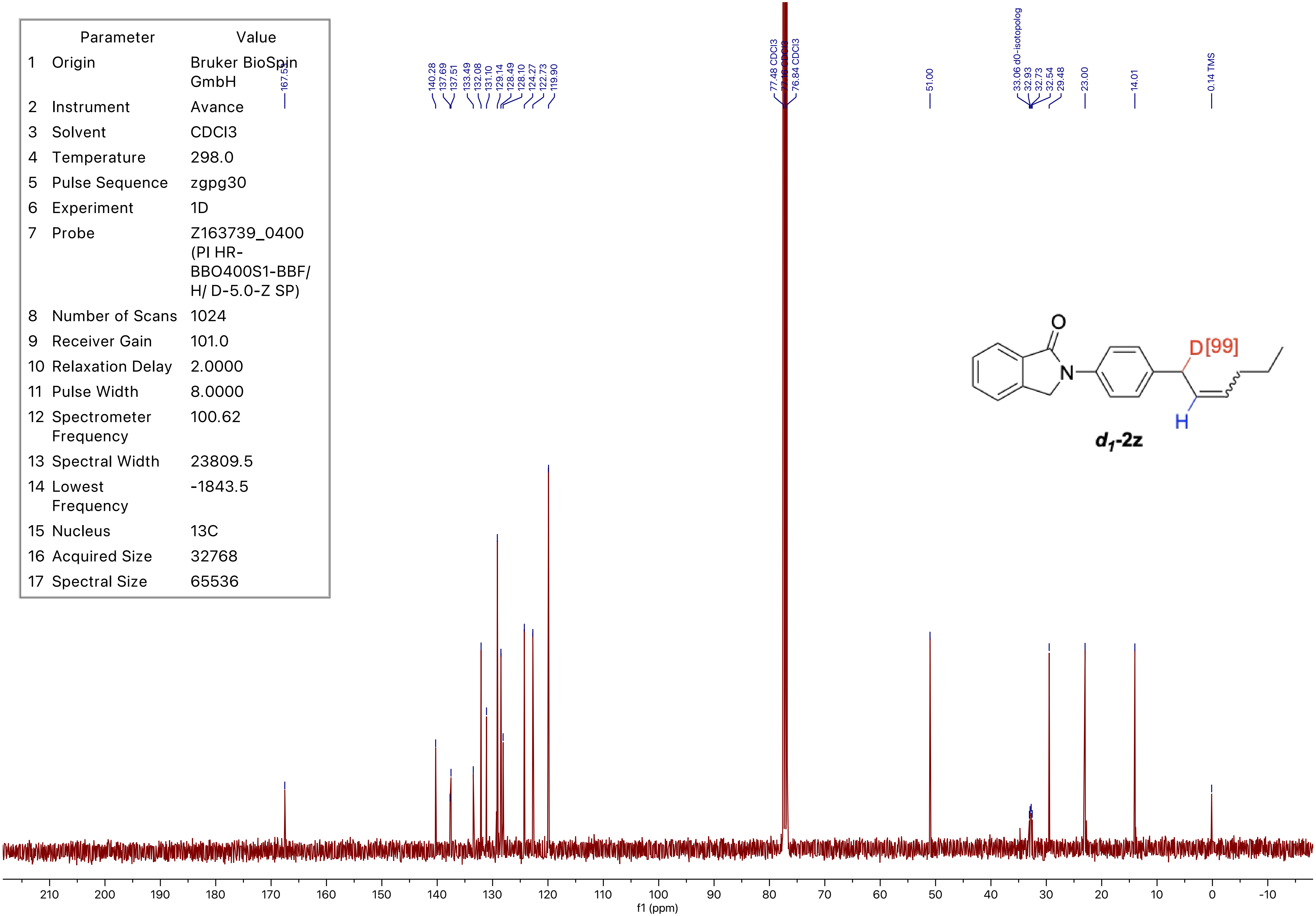

***d*$_1$-2z**

Parameter	Value
1 Origin	Varian
2 Solvent	cdcl3
3 Temperature	25.0
4 Pulse Sequence	s2pul
5 Experiment	1D
6 Probe	OneNMR_W036
7 Number of Scans	8
8 Receiver Gain	28
9 Relaxation Delay	1.0000
10 Pulse Width	4.8000
11 Spectrometer Frequency	499.73
12 Spectral Width	8012.8
13 Lowest Frequency	-1006.3
14 Nucleus	1H
15 Acquired Size	16384
16 Spectral Size	65536
17 Digital Resolution	0.12

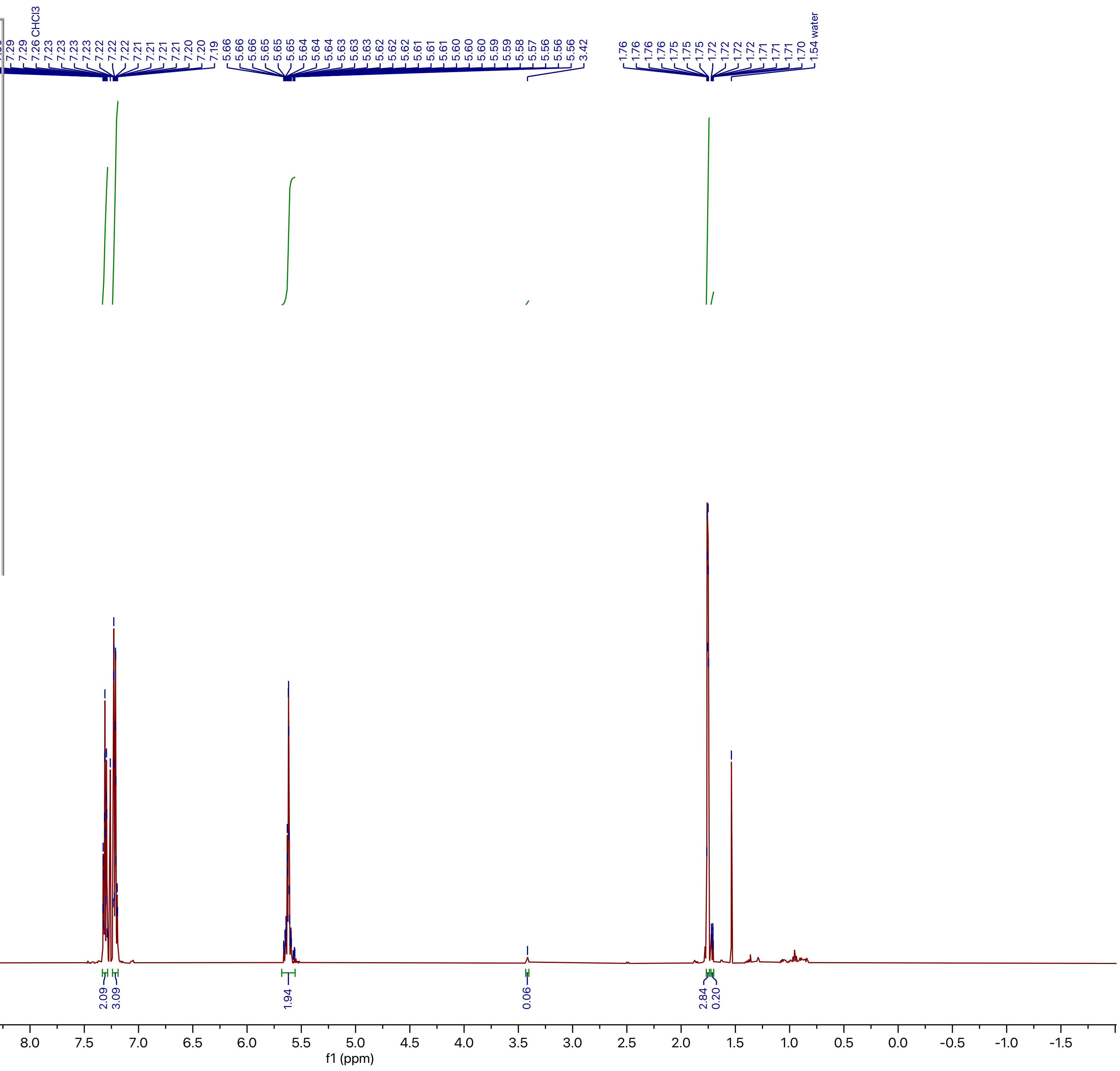

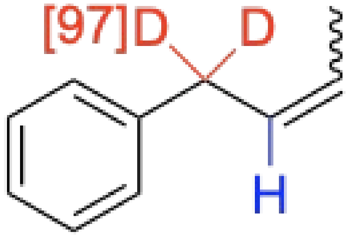

d_2-2a

	Parameter	Value
1	Origin	Varian
2	Solvent	cdcl3
3	Temperature	25.0
4	Pulse Sequence	s2pul
5	Experiment	1D
6	Probe	OneNMR_W036
7	Number of Scans	64
8	Receiver Gain	20
9	Relaxation Delay	5.0000
10	Pulse Width	300.0000
11	Spectrometer Frequency	76.71
12	Spectral Width	1535.6
13	Lowest Frequency	-384.2
14	Nucleus	lk
15	Acquired Size	2048
16	Spectral Size	4096
17	Digital Resolution	0.37

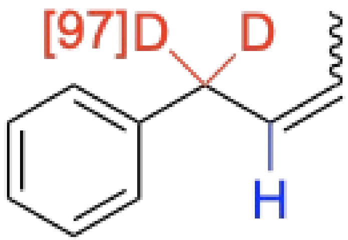

d_2-2a

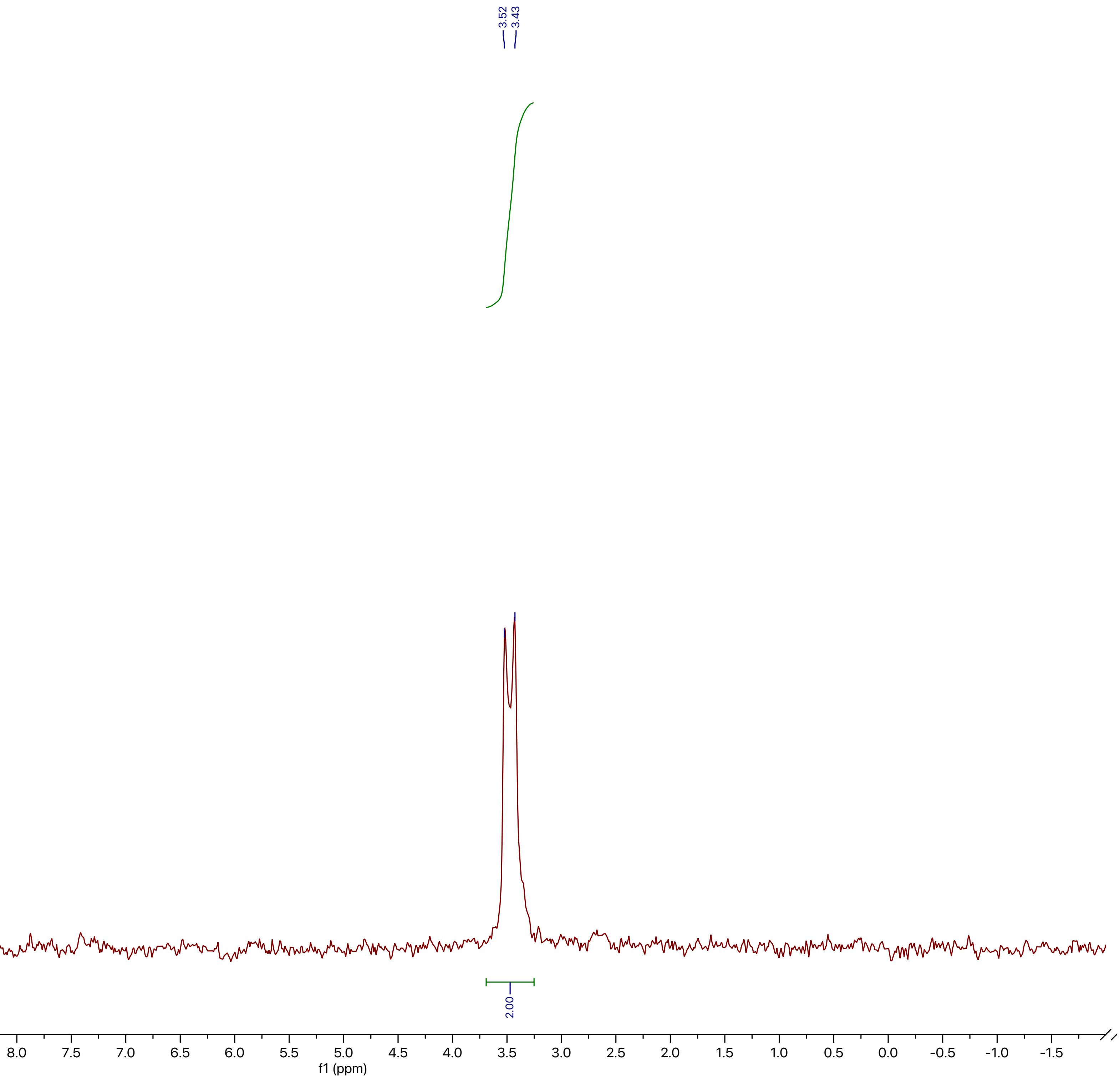

	Parameter	Value
1	Origin	Bruker BioSpin GmbH
2	Instrument	Avance
3	Solvent	CDCl3
4	Temperature	300.0
5	Pulse Sequence	zgpg30
6	Experiment	1D
7	Probe	Z151574_0073 (PI HR-BBO500S1-BBF/ H/ D-5.0-Z SP)
8	Number of Scans	1024
9	Receiver Gain	101.0
10	Relaxation Delay	2.0000
11	Pulse Width	9.0000
12	Spectrometer Frequency	125.79
13	Spectral Width	30120.5
14	Lowest Frequency	-2482.4
15	Nucleus	13C
16	Acquired Size	32768
17	Spectral Size	65536

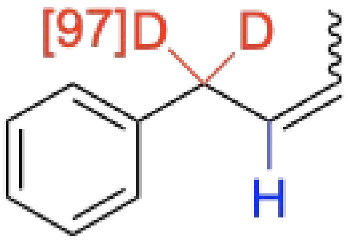

*d*₂-2a

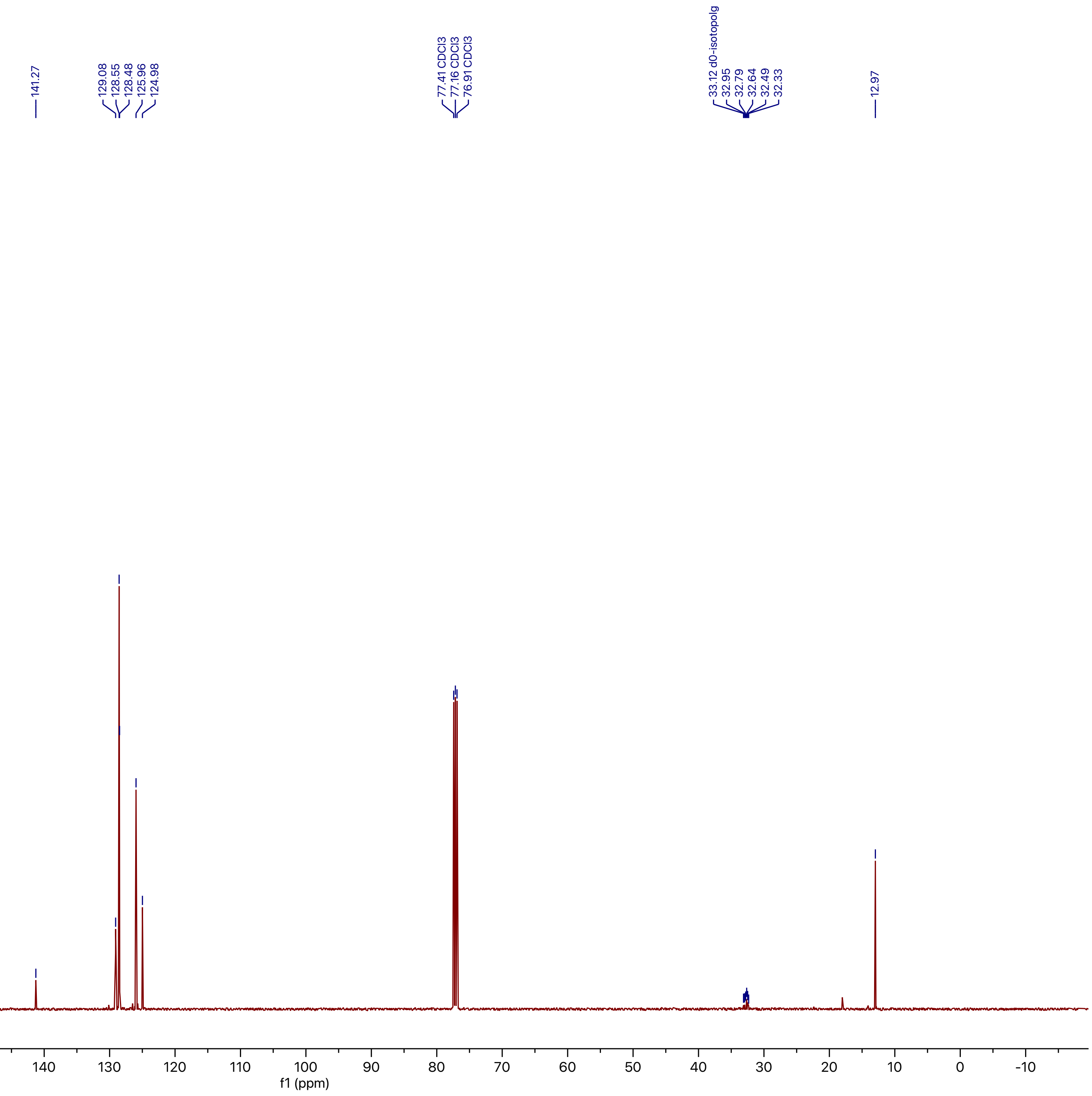

	Parameter	Value
1	Origin	Bruker BioSpin GmbH
2	Instrument	Avance
3	Solvent	CDCl3
4	Temperature	300.0
5	Pulse Sequence	zg30
6	Experiment	1D
7	Probe	Z151574_0073 (PI HR-BBO500S1-BBF/ H/ D-5.0-Z SP)
8	Number of Scans	32
9	Receiver Gain	63.0
10	Relaxation Delay	1.0000
11	Pulse Width	8.0000
12	Spectrometer Frequency	500.21
13	Spectral Width	10000.0
14	Lowest Frequency	-1922.6
15	Nucleus	1H
16	Acquired Size	32768
17	Spectral Size	65536

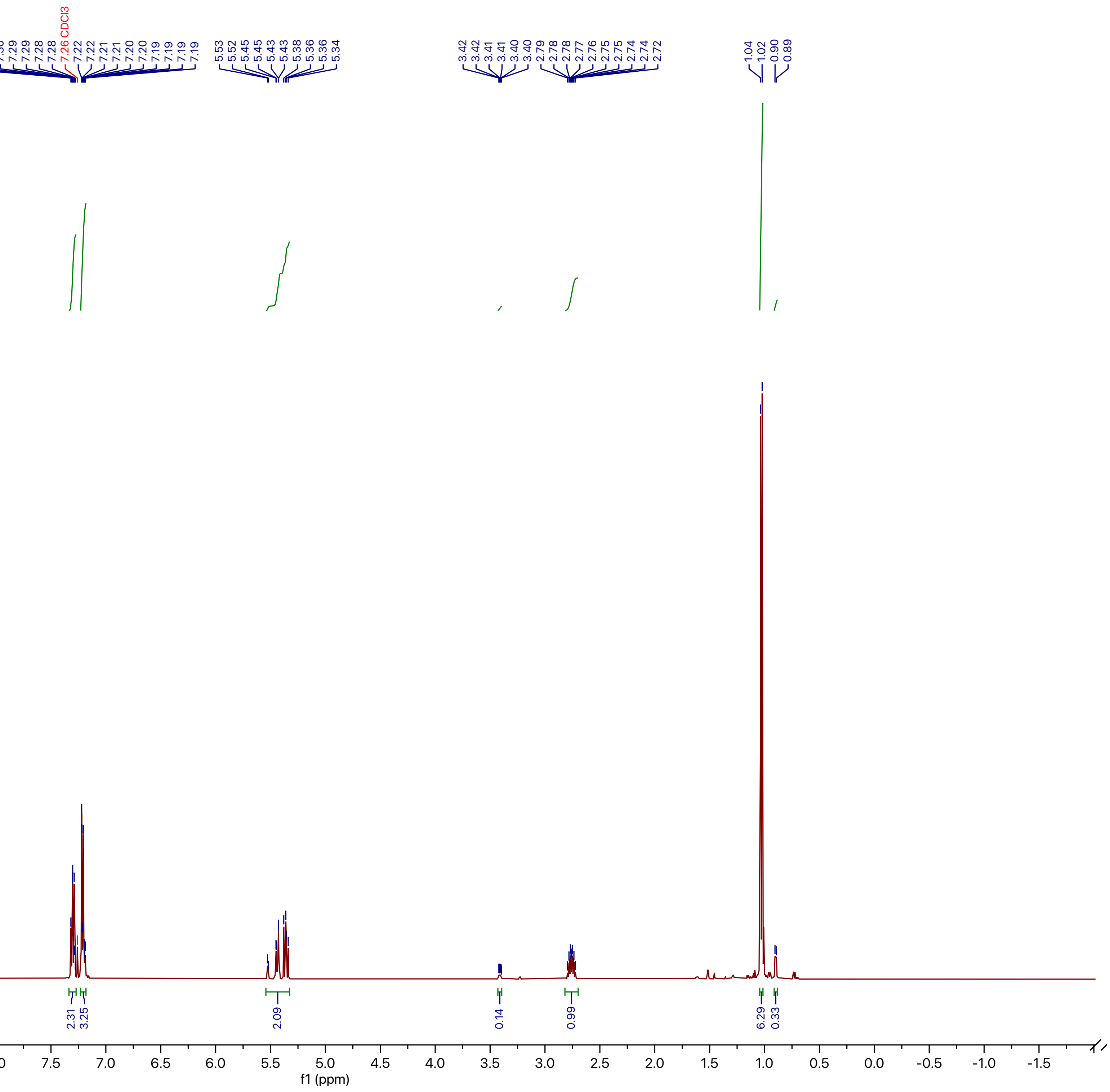

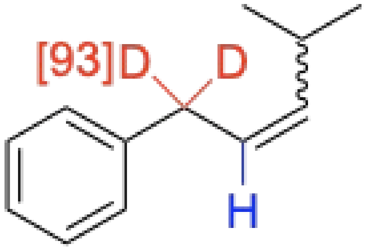

***d*$_2$-2b**

Parameter		Value
1	Origin	Varian
2	Solvent	cdcl3
3	Temperature	25.0
4	Pulse Sequence	s2pul
5	Experiment	1D
6	Probe	OneNMR_W036
7	Number of Scans	32
8	Receiver Gain	20
9	Relaxation Delay	5.0000
10	Pulse Width	300.0000
11	Spectrometer Frequency	76.71
12	Spectral Width	1535.6
13	Lowest Frequency	-384.2
14	Nucleus	lk
15	Acquired Size	2048
16	Spectral Size	4096
17	Digital Resolution	0.37

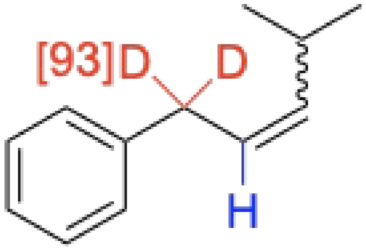

***d*$_2$-2b**

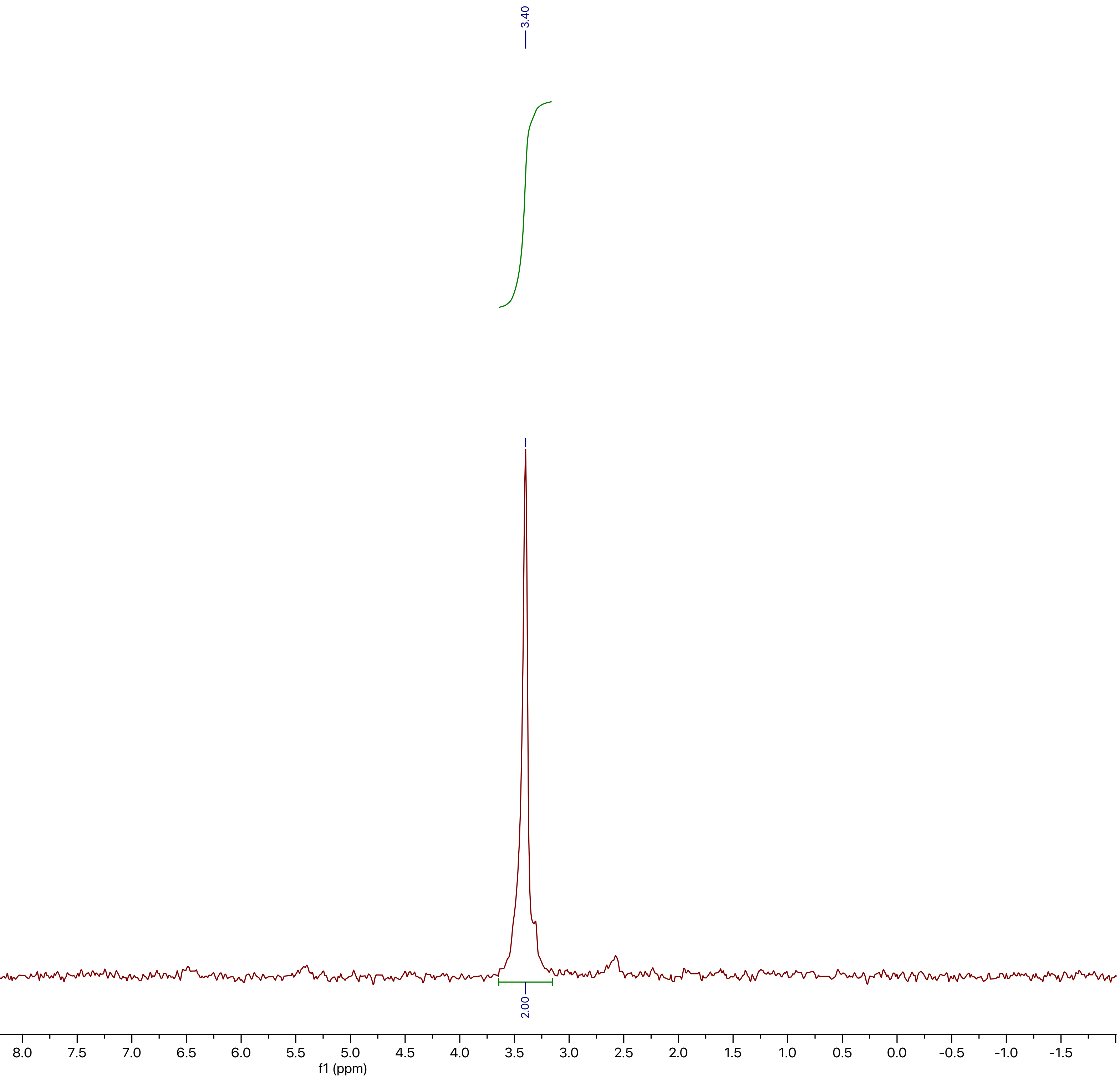

	Parameter	Value
1	Origin	Bruker BioSpin GmbH
2	Instrument	Avance
3	Solvent	CDCl3
4	Temperature	300.0
5	Pulse Sequence	zgpg30
6	Experiment	1D
7	Probe	Z151574_0073 (PI HR-BBO500S1-BBF/ H/ D-5.0-Z SP)
8	Number of Scans	500
9	Receiver Gain	101.0
10	Relaxation Delay	2.0000
11	Pulse Width	9.0000
12	Spectrometer Frequency	125.79
13	Spectral Width	30120.5
14	Lowest Frequency	-2465.7
15	Nucleus	13C
16	Acquired Size	32768
17	Spectral Size	65536

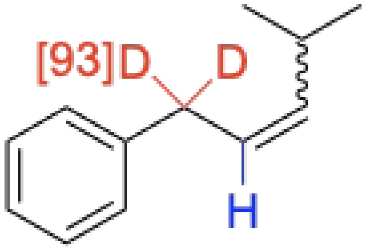

***d*$_2$-2b**

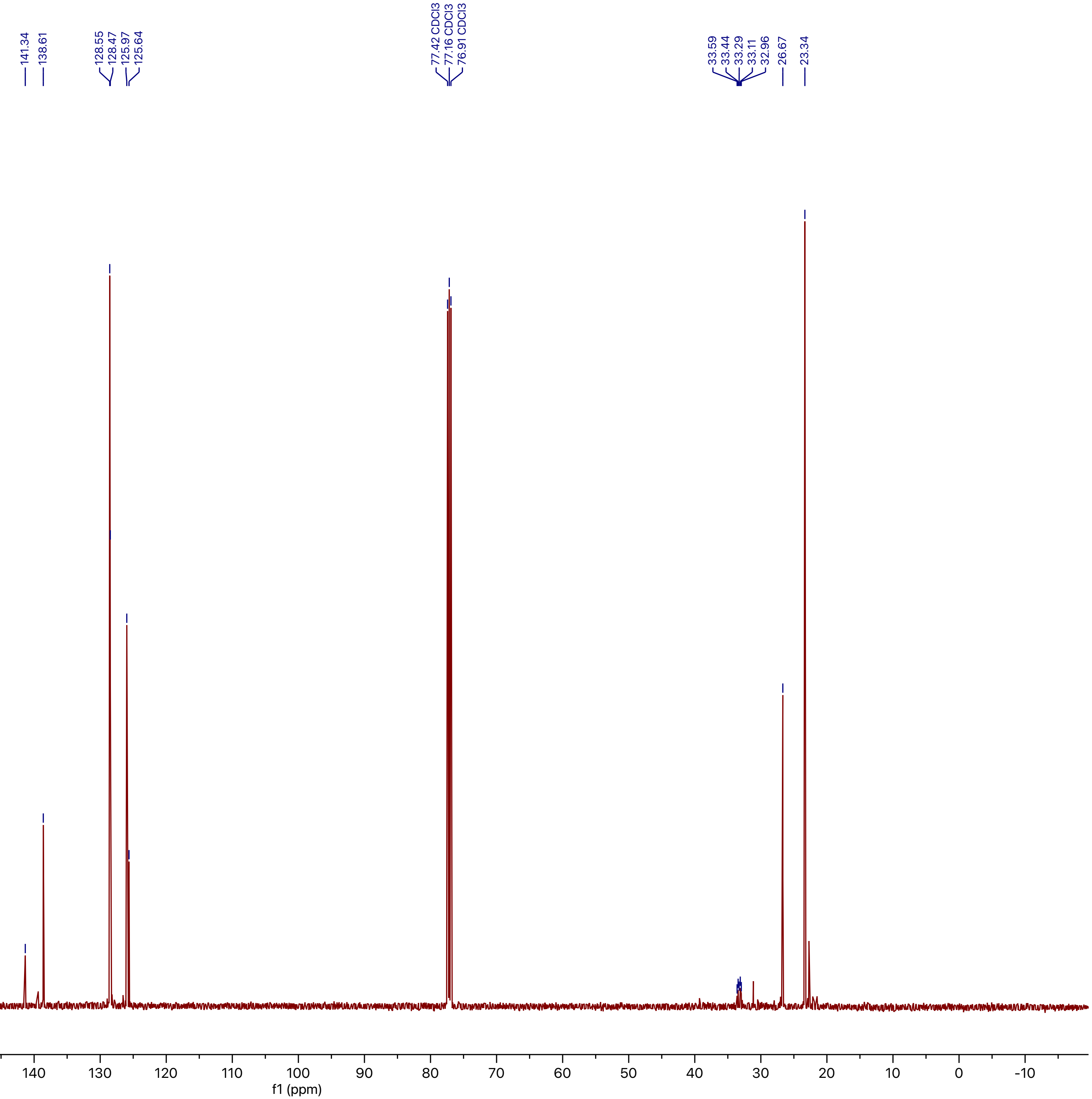

	Parameter	Value
1	Origin	Bruker BioSpin GmbH
2	Instrument	Avance
3	Solvent	CDCl3
4	Temperature	300.0
5	Pulse Sequence	zg30
6	Experiment	1D
7	Probe	Z151574_0073 (PI HR-BBO500S1-BBF/ H/ D-5.0-Z SP)
8	Number of Scans	16
9	Receiver Gain	65.0
10	Relaxation Delay	1.0000
11	Pulse Width	8.0000
12	Spectrometer Frequency	500.21
13	Spectral Width	10000.0
14	Lowest Frequency	-1982.6
15	Nucleus	1H
16	Acquired Size	32768
17	Spectral Size	65536

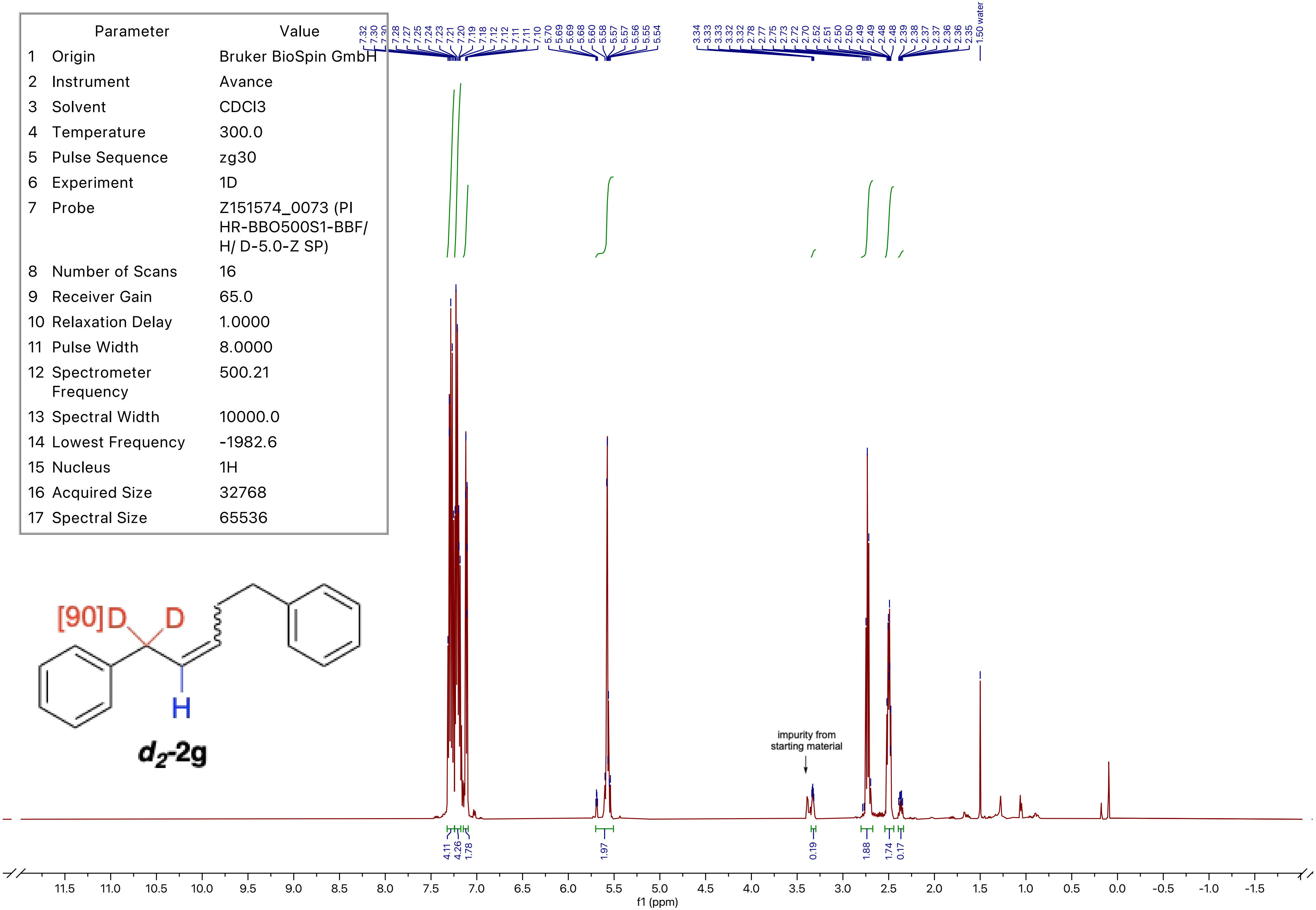

***d*$_2$-2g**

	Parameter	Value
1	Origin	Varian
2	Solvent	cdcl3
3	Temperature	25.0
4	Pulse Sequence	s2pul
5	Experiment	1D
6	Probe	OneNMR_W036
7	Number of Scans	32
8	Receiver Gain	20
9	Relaxation Delay	5.0000
10	Pulse Width	300.0000
11	Spectrometer Frequency	76.71
12	Spectral Width	1535.6
13	Lowest Frequency	-384.2
14	Nucleus	lk
15	Acquired Size	2048
16	Spectral Size	4096
17	Digital Resolution	0.37

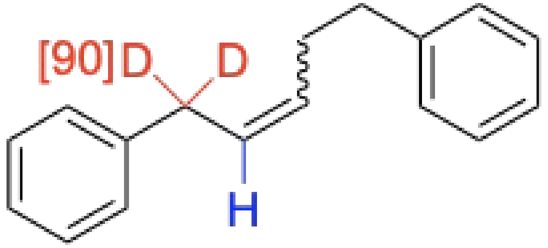

d_2-2g

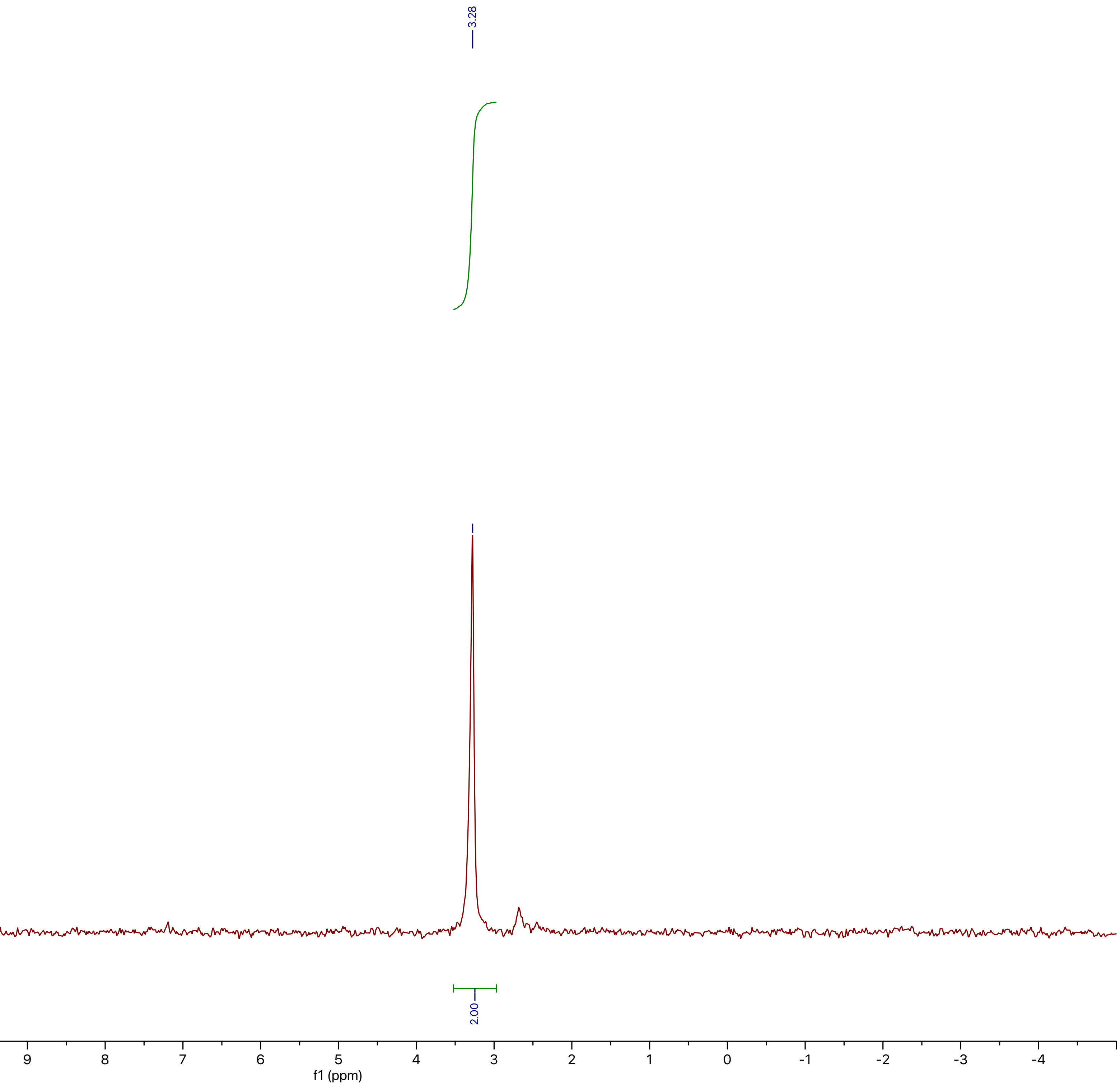

Parameter		Value
1	Origin	Bruker BioSpin GmbH
2	Instrument	Avance
3	Solvent	CDCl3
4	Temperature	300.0
5	Pulse Sequence	zgpg30
6	Experiment	1D
7	Probe	Z151574_0073 (PI HR-BBO500S1-BBF/ H/ D-5.0-Z SP)
8	Number of Scans	1024
9	Receiver Gain	101.0
10	Relaxation Delay	2.0000
11	Pulse Width	9.0000
12	Spectrometer Frequency	125.79
13	Spectral Width	30120.5
14	Lowest Frequency	-2482.4
15	Nucleus	13C
16	Acquired Size	32768
17	Spectral Size	65536

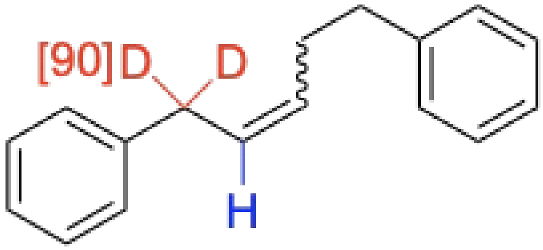

d$_2$-2g

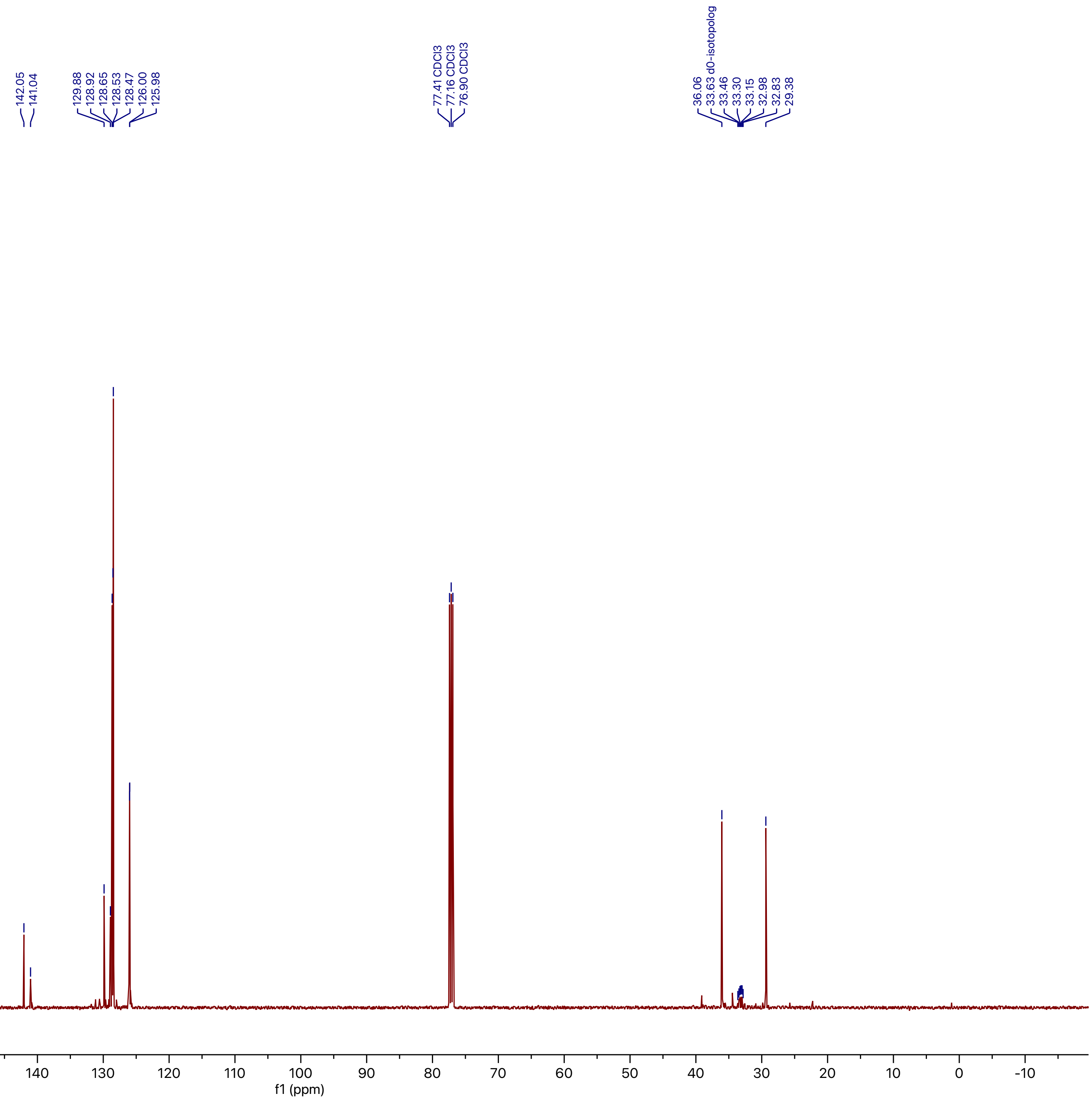

	Parameter	Value
1	Origin	Bruker BioSpin GmbH
2	Instrument	Avance
3	Solvent	CDCl3
4	Temperature	300.0
5	Pulse Sequence	zg30
6	Experiment	1D
7	Probe	Z151574_0073 (PI HR-BBO500S1-BBF/ H/ D-5.0-Z SP)
8	Number of Scans	16
9	Receiver Gain	46.2
10	Relaxation Delay	1.0000
11	Pulse Width	8.0000
12	Spectrometer Frequency	500.21
13	Spectral Width	10000.0
14	Lowest Frequency	-1905.4
15	Nucleus	1H
16	Acquired Size	32768
17	Spectral Size	65536

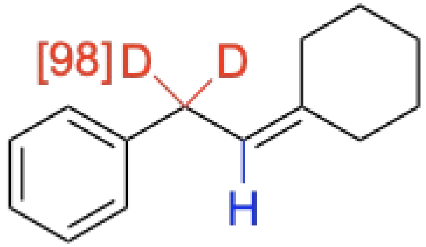

***d*$_2$-4a**

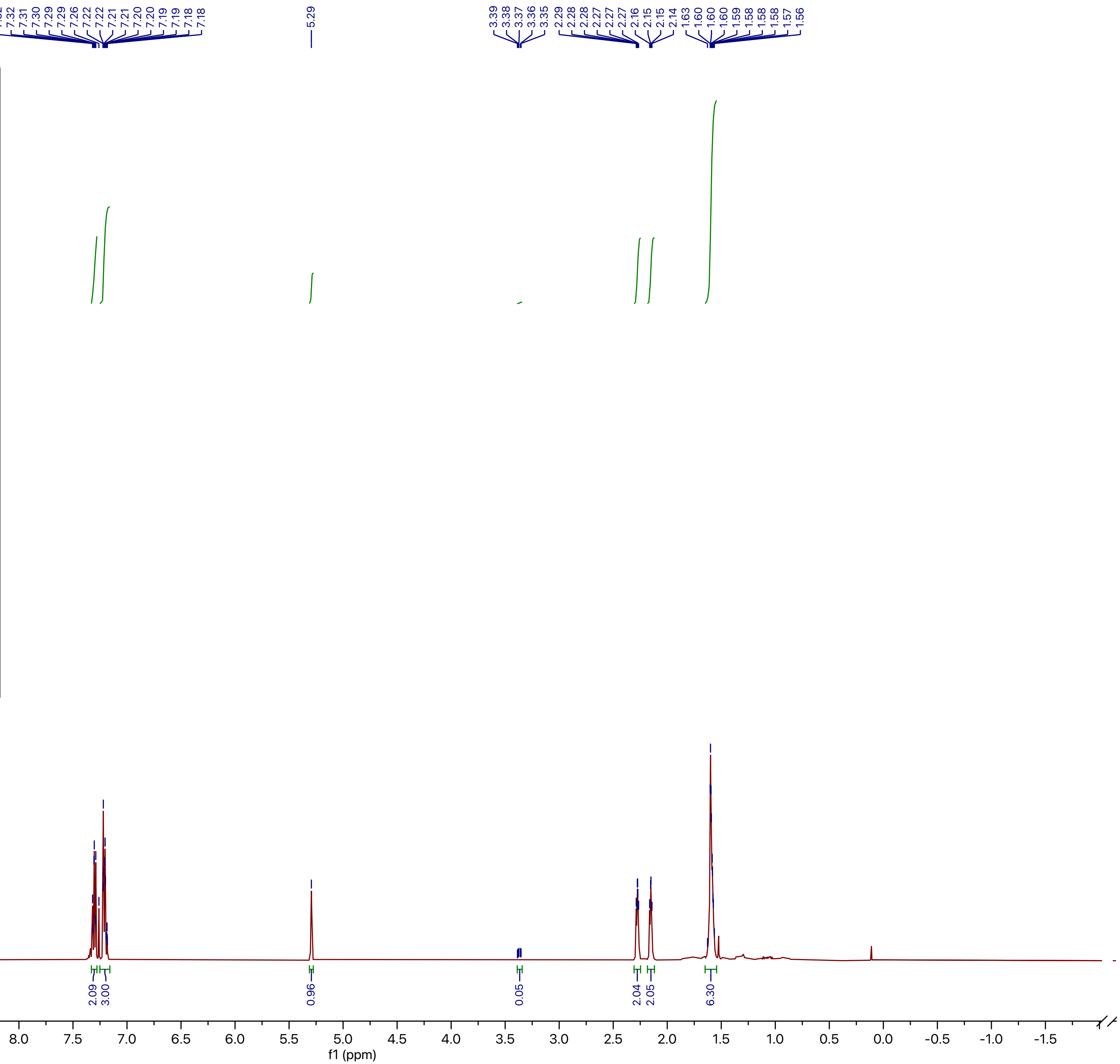

	Parameter	Value
1	Origin	Varian
2	Solvent	cdcl3
3	Temperature	25.0
4	Pulse Sequence	s2pul
5	Experiment	1D
6	Probe	OneNMR_W036
7	Number of Scans	32
8	Receiver Gain	20
9	Relaxation Delay	5.0000
10	Pulse Width	300.0000
11	Spectrometer Frequency	76.71
12	Spectral Width	1535.6
13	Lowest Frequency	-379.9
14	Nucleus	lk
15	Acquired Size	2048
16	Spectral Size	4096
17	Digital Resolution	0.37

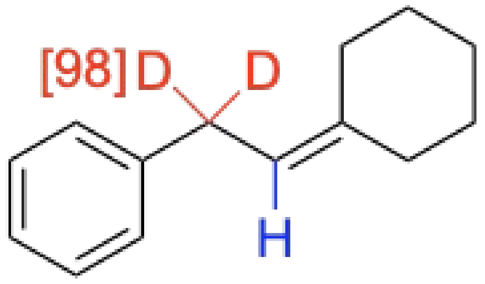

d_2-4a

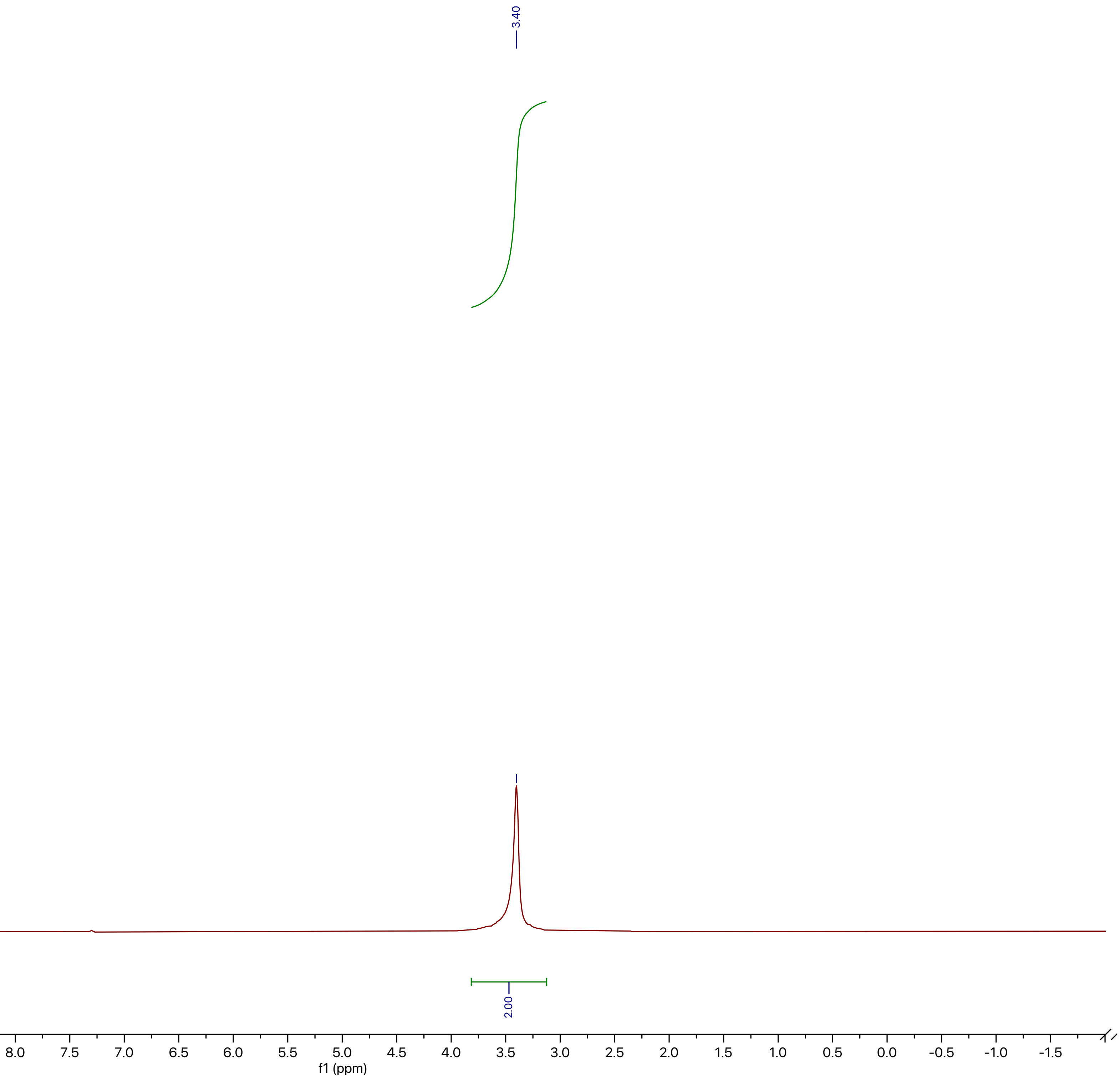

Parameter		Value
1	Origin	Bruker BioSpin GmbH
2	Instrument	Avance
3	Solvent	CDCl3
4	Temperature	300.0
5	Pulse Sequence	zgpg30
6	Experiment	1D
7	Probe	Z151574_0073 (PI HR-BBO500S1-BBF/ H/ D-5.0-Z SP)
8	Number of Scans	1024
9	Receiver Gain	101.0
10	Relaxation Delay	2.0000
11	Pulse Width	9.0000
12	Spectrometer Frequency	125.79
13	Spectral Width	30120.5
14	Lowest Frequency	-2482.4
15	Nucleus	13C
16	Acquired Size	32768
17	Spectral Size	65536

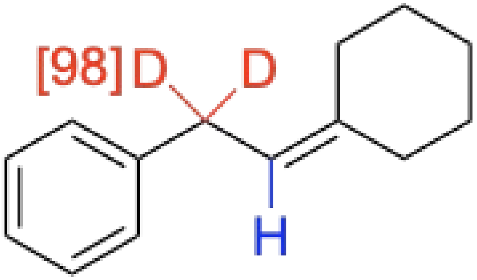

***d*$_2$-4a**

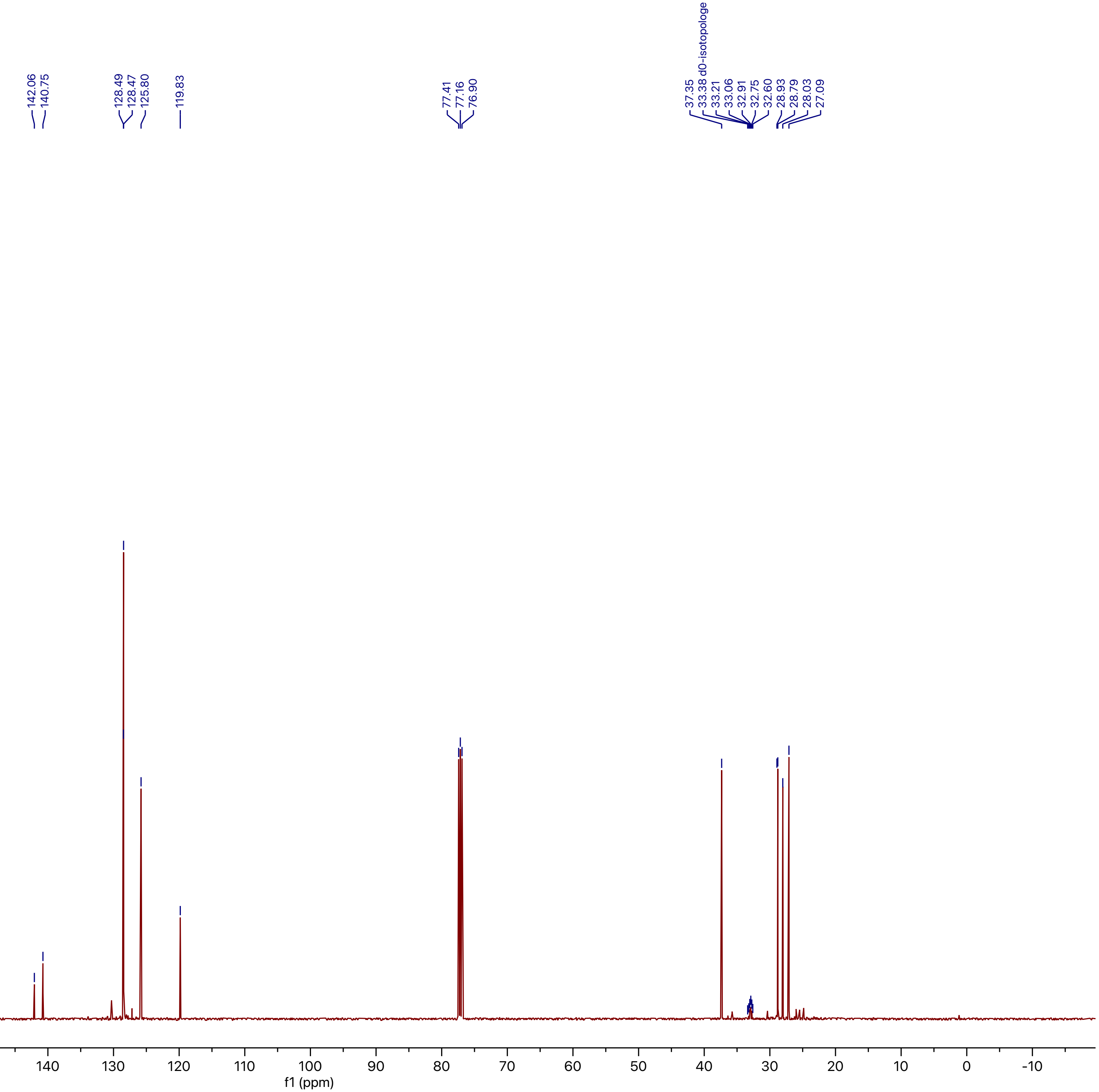

	Parameter	Value
1	Origin	Bruker BioSpin GmbH
2	Instrument	Avance
3	Solvent	CDCl3
4	Temperature	300.0
5	Pulse Sequence	zg30
6	Experiment	1D
7	Probe	Z151574_0073 (PI HR-BBO500S1-BBF/ H/ D-5.0-Z SP)
8	Number of Scans	16
9	Receiver Gain	101.0
10	Relaxation Delay	1.0000
11	Pulse Width	8.0000
12	Spectrometer Frequency	500.21
13	Spectral Width	10000.0
14	Lowest Frequency	-1911.2
15	Nucleus	1H
16	Acquired Size	32768
17	Spectral Size	65536

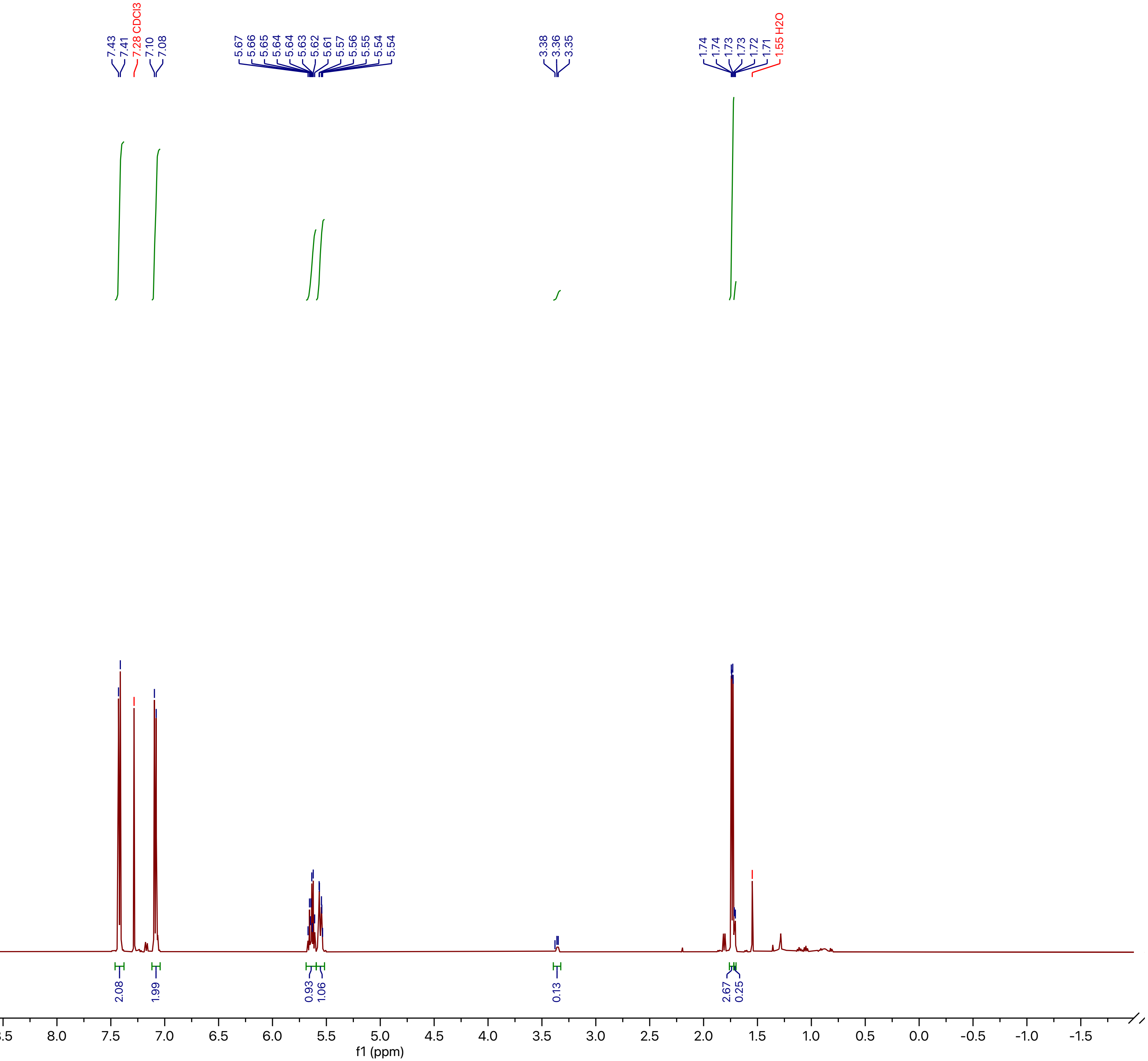

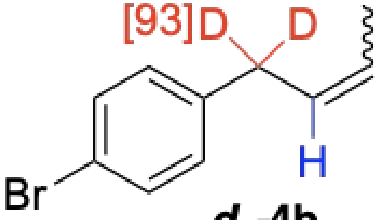

d$_2$-**4b**

	Parameter	Value
1	Origin	Varian
2	Solvent	cdcl3
3	Temperature	25.0
4	Pulse Sequence	s2pul
5	Experiment	1D
6	Probe	OneNMR_W036
7	Number of Scans	32
8	Receiver Gain	20
9	Relaxation Delay	5.0000
10	Pulse Width	300.0000
11	Spectrometer Frequency	76.71
12	Spectral Width	1535.6
13	Lowest Frequency	-381.2
14	Nucleus	lk
15	Acquired Size	2048
16	Spectral Size	4096
17	Digital Resolution	0.37

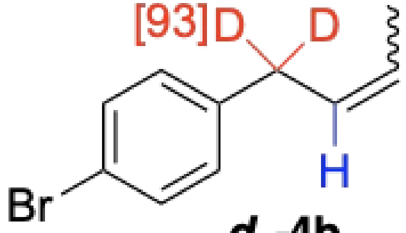

d_2-4b

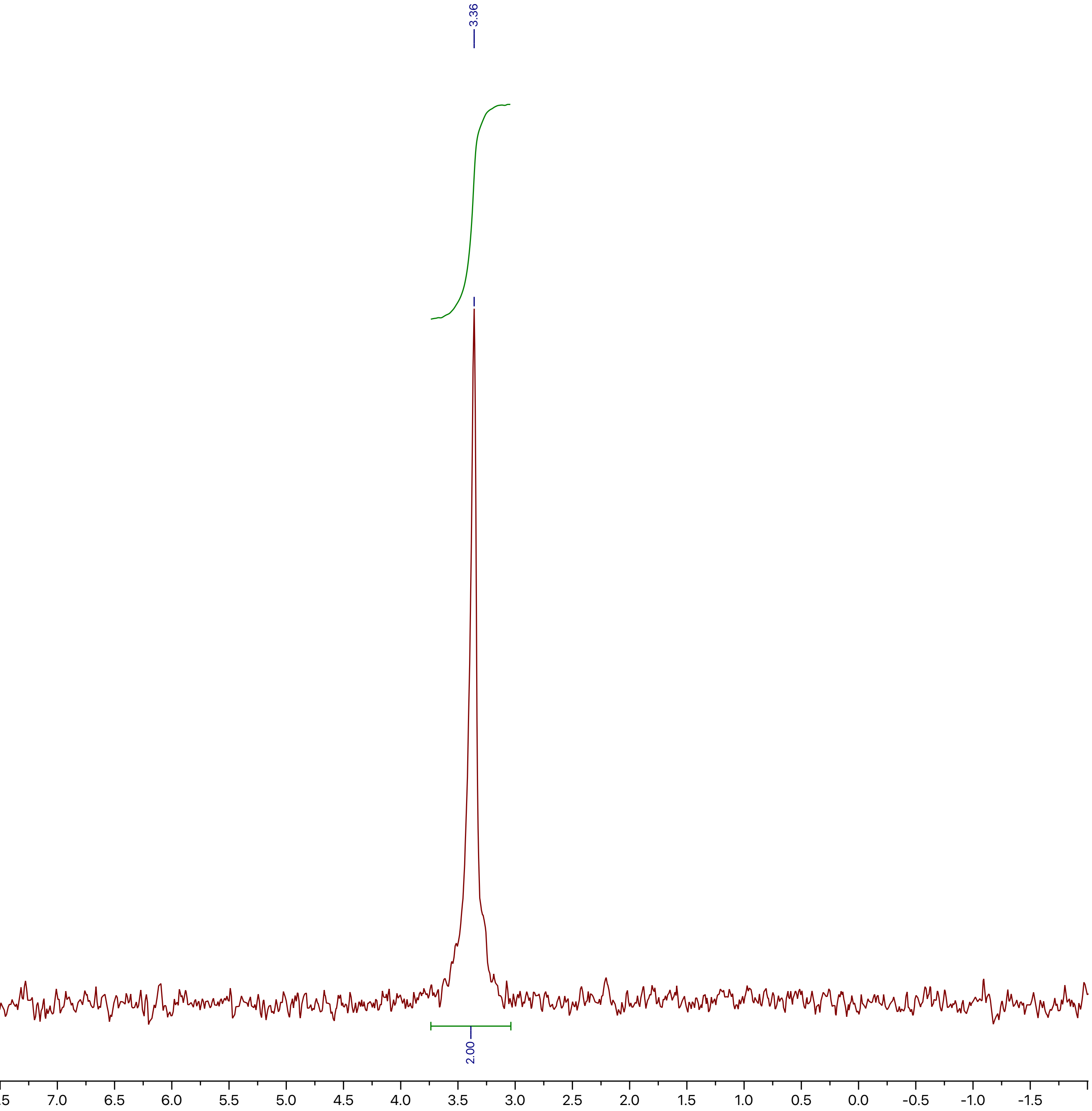

	Parameter	Value
1	Origin	Bruker BioSpin GmbH
2	Instrument	Avance
3	Solvent	CDCl3
4	Temperature	300.0
5	Pulse Sequence	zgpg30
6	Experiment	1D
7	Probe	Z151574_0073 (PI HR-BBO500S1-BBF/H/ D-5.0-Z SP)
8	Number of Scans	6000
9	Receiver Gain	101.0
10	Relaxation Delay	2.0000
11	Pulse Width	9.0000
12	Spectrometer Frequency	125.79
13	Spectral Width	30120.5
14	Lowest Frequency	-2482.4
15	Nucleus	13C
16	Acquired Size	32768
17	Spectral Size	65536

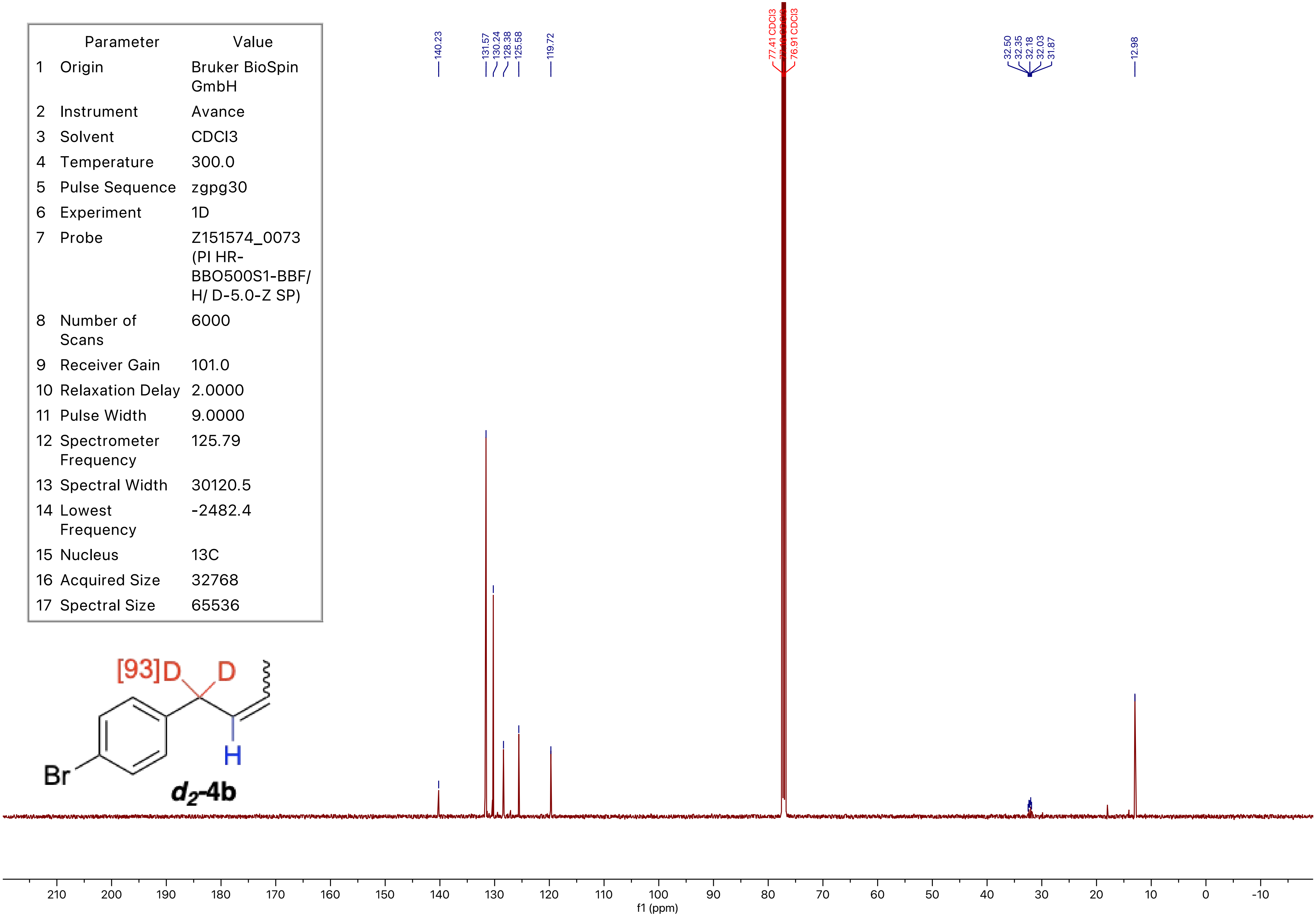

	Parameter	Value
1	Origin	Bruker BioSpin GmbH
2	Instrument	Avance
3	Solvent	CDCl3
4	Temperature	300.0
5	Pulse Sequence	zg30
6	Experiment	1D
7	Probe	Z151574_0073 (PI HR-BBO500S1-BBF/ H/ D-5.0-Z SP)
8	Number of Scans	16
9	Receiver Gain	44.3
10	Relaxation Delay	1.0000
11	Pulse Width	8.0000
12	Spectrometer Frequency	500.21
13	Spectral Width	10000.0
14	Lowest Frequency	-1954.1
15	Nucleus	1H
16	Acquired Size	32768
17	Spectral Size	65536

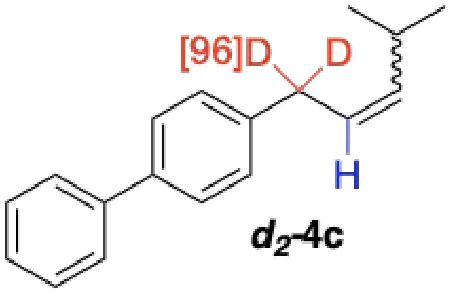

d_2-4c

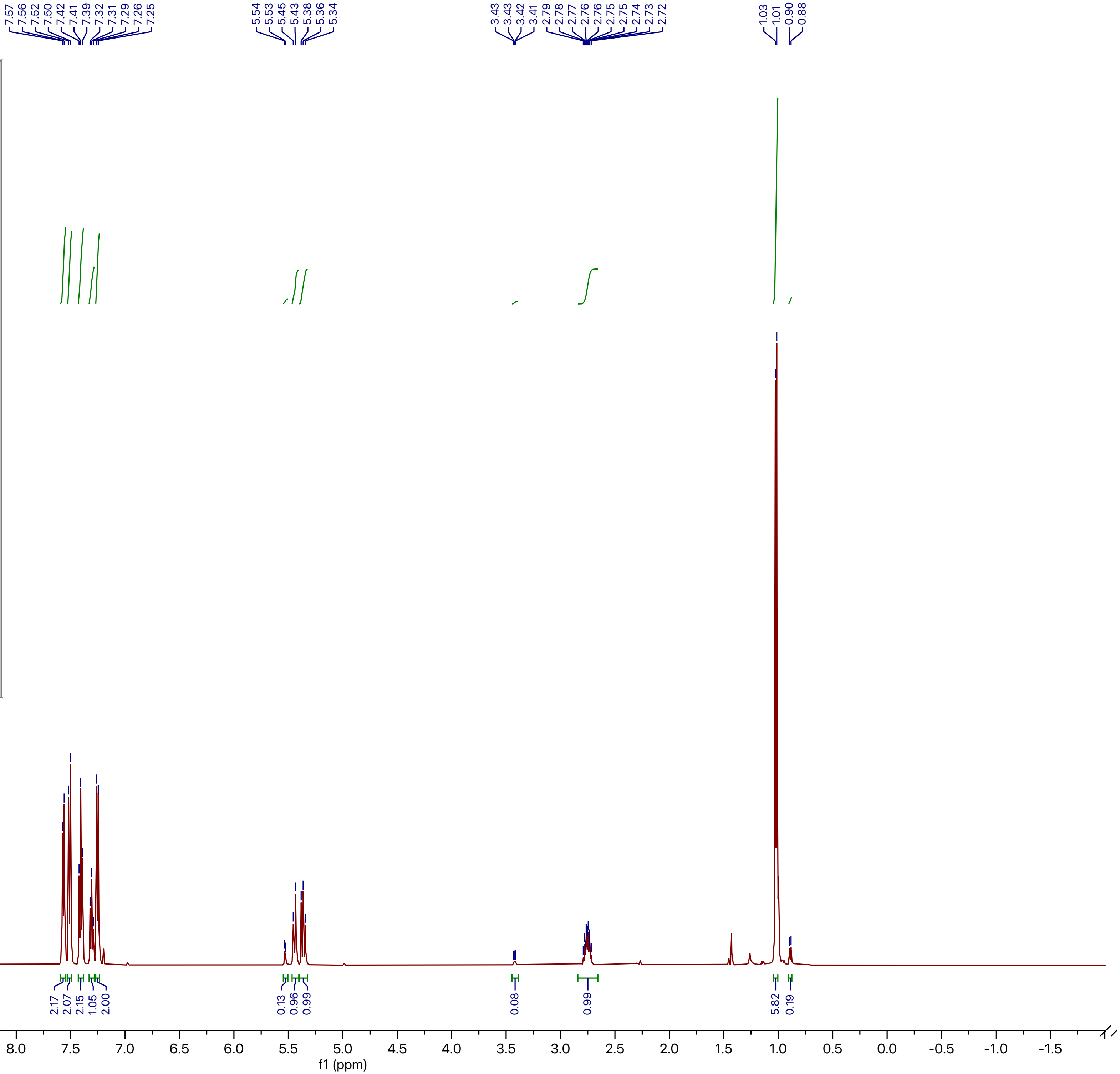

	Parameter	Value
1	Origin	Varian
2	Solvent	cdcl3
3	Temperature	25.0
4	Pulse Sequence	s2pul
5	Experiment	1D
6	Probe	OneNMR_W036
7	Number of Scans	32
8	Receiver Gain	20
9	Relaxation Delay	5.0000
10	Pulse Width	300.0000
11	Spectrometer Frequency	76.71
12	Spectral Width	1535.6
13	Lowest Frequency	-384.2
14	Nucleus	lk
15	Acquired Size	2048
16	Spectral Size	4096
17	Digital Resolution	0.37

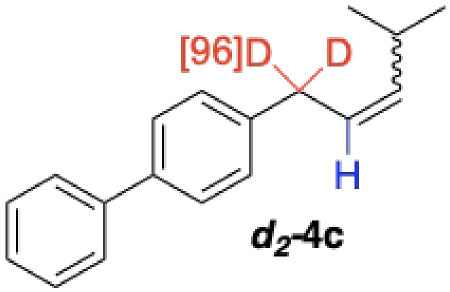

d_2-4c

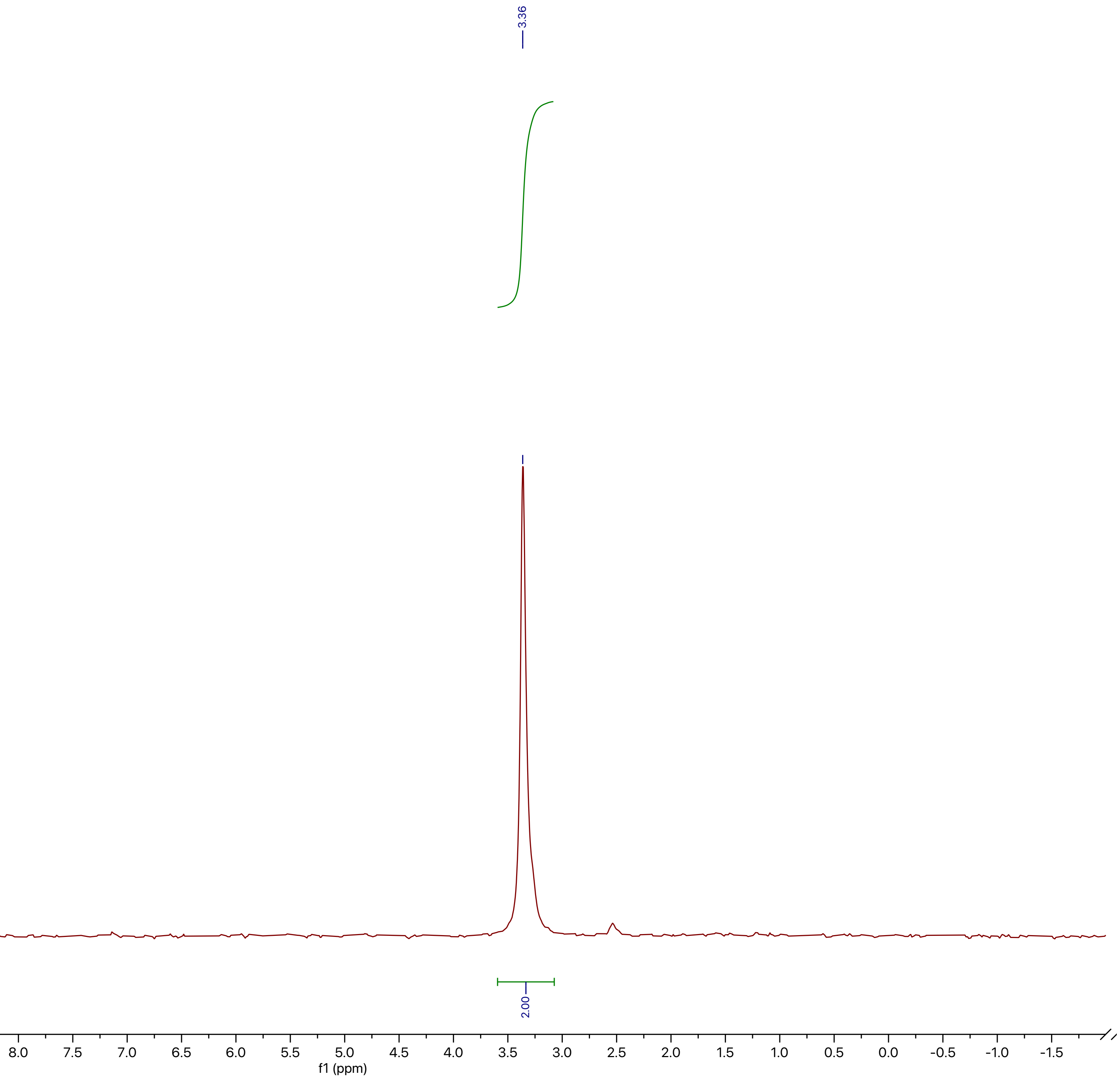

	Parameter	Value
1	Origin	Bruker BioSpin GmbH
2	Instrument	Avance
3	Solvent	CDCl3
4	Temperature	300.0
5	Pulse Sequence	zgpg30
6	Experiment	1D
7	Probe	Z151574_0073 (PI HR-BBO500S1-BBF/ H/ D-5.0-Z SP)
8	Number of Scans	4000
9	Receiver Gain	101.0
10	Relaxation Delay	10.0000
11	Pulse Width	9.0000
12	Spectrometer Frequency	125.79
13	Spectral Width	30120.5
14	Lowest Frequency	-2471.2
15	Nucleus	13C
16	Acquired Size	32768
17	Spectral Size	65536

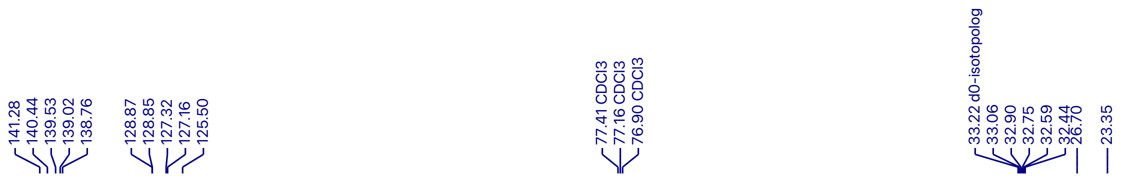

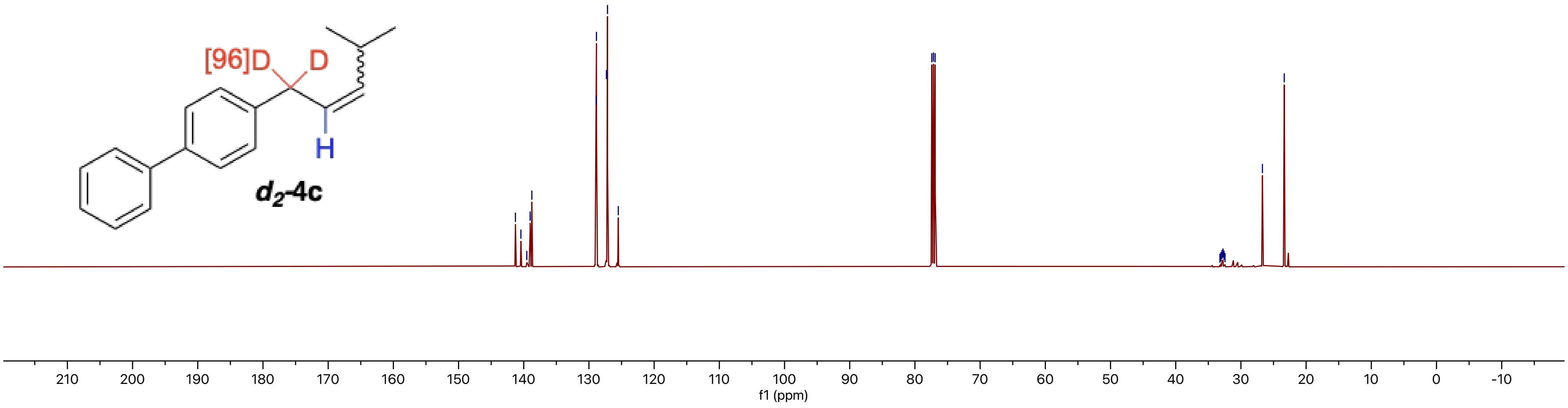

	Parameter	Value
1	Origin	Varian
2	Solvent	cdcl3
3	Temperature	25.0
4	Pulse Sequence	s2pul
5	Experiment	1D
6	Probe	ASWPFG8319
7	Number of Scans	16
8	Receiver Gain	52
9	Relaxation Delay	10.0000
10	Pulse Width	7.7500
11	Spectrometer Frequency	399.73
12	Spectral Width	6410.3
13	Lowest Frequency	-806.0
14	Nucleus	1H
15	Acquired Size	16384
16	Spectral Size	65536
17	Digital Resolution	0.10

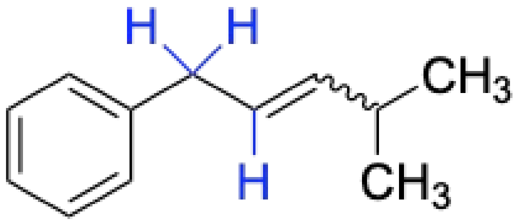

***d*$_0$-2b**

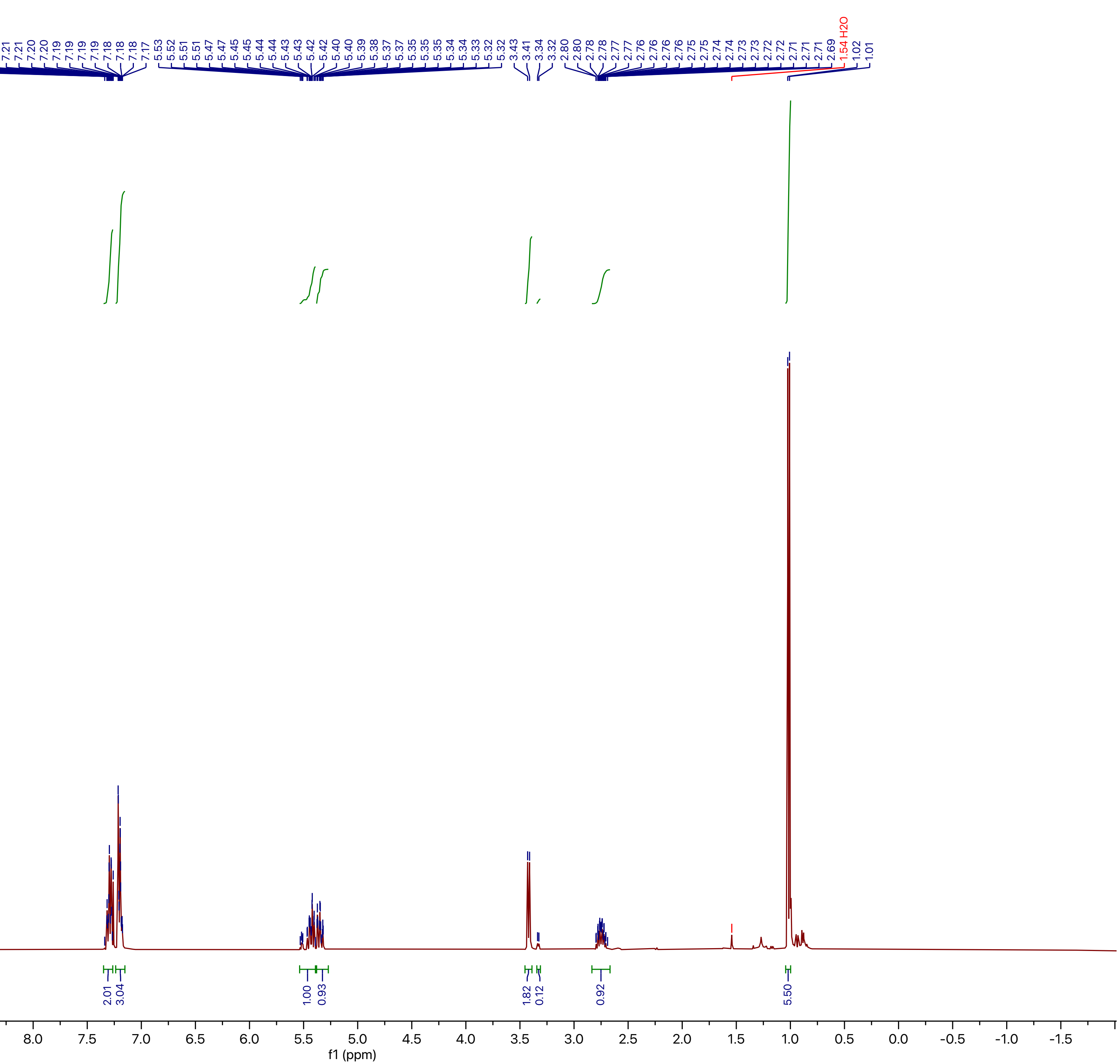

	Parameter	Value
1	Origin	Bruker BioSpin GmbH
2	Instrument	Avance
3	Solvent	CDCl3
4	Temperature	300.0
5	Pulse Sequence	zg30
6	Experiment	1D
7	Probe	Z151574_0073 (PI HR-BBO500S1-BBF/ H/ D-5.0-Z SP)
8	Number of Scans	16
9	Receiver Gain	63.0
10	Relaxation Delay	1.0000
11	Pulse Width	8.0000
12	Spectrometer Frequency	500.21
13	Spectral Width	10000.0
14	Lowest Frequency	-1922.7
15	Nucleus	1H
16	Acquired Size	32768
17	Spectral Size	65536

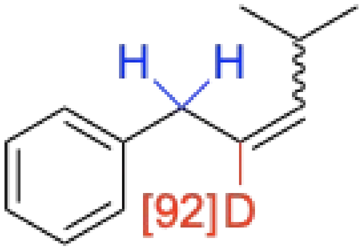

***d*$_1$-2b**

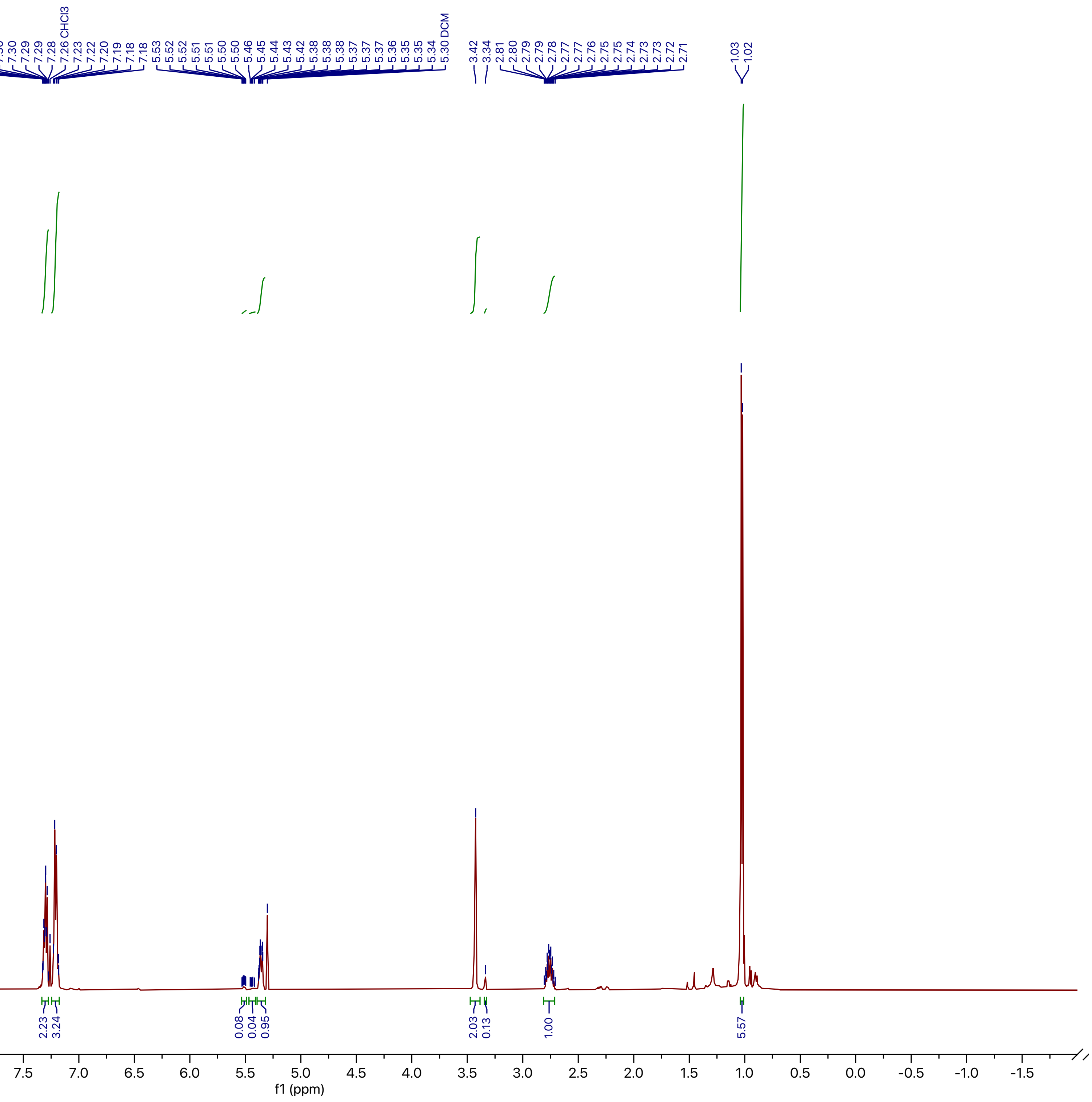

	Parameter	Value
1	Origin	Varian
2	Solvent	cdcl3
3	Temperature	25.0
4	Pulse Sequence	s2pul
5	Experiment	1D
6	Probe	OneNMR_W036
7	Number of Scans	32
8	Receiver Gain	20
9	Relaxation Delay	5.0000
10	Pulse Width	300.0000
11	Spectrometer Frequency	76.71
12	Spectral Width	1535.6
13	Lowest Frequency	-379.6
14	Nucleus	lk
15	Acquired Size	2048
16	Spectral Size	4096
17	Digital Resolution	0.37

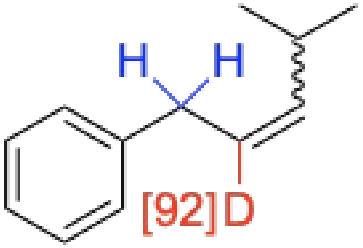

***d*$_1$-2b**

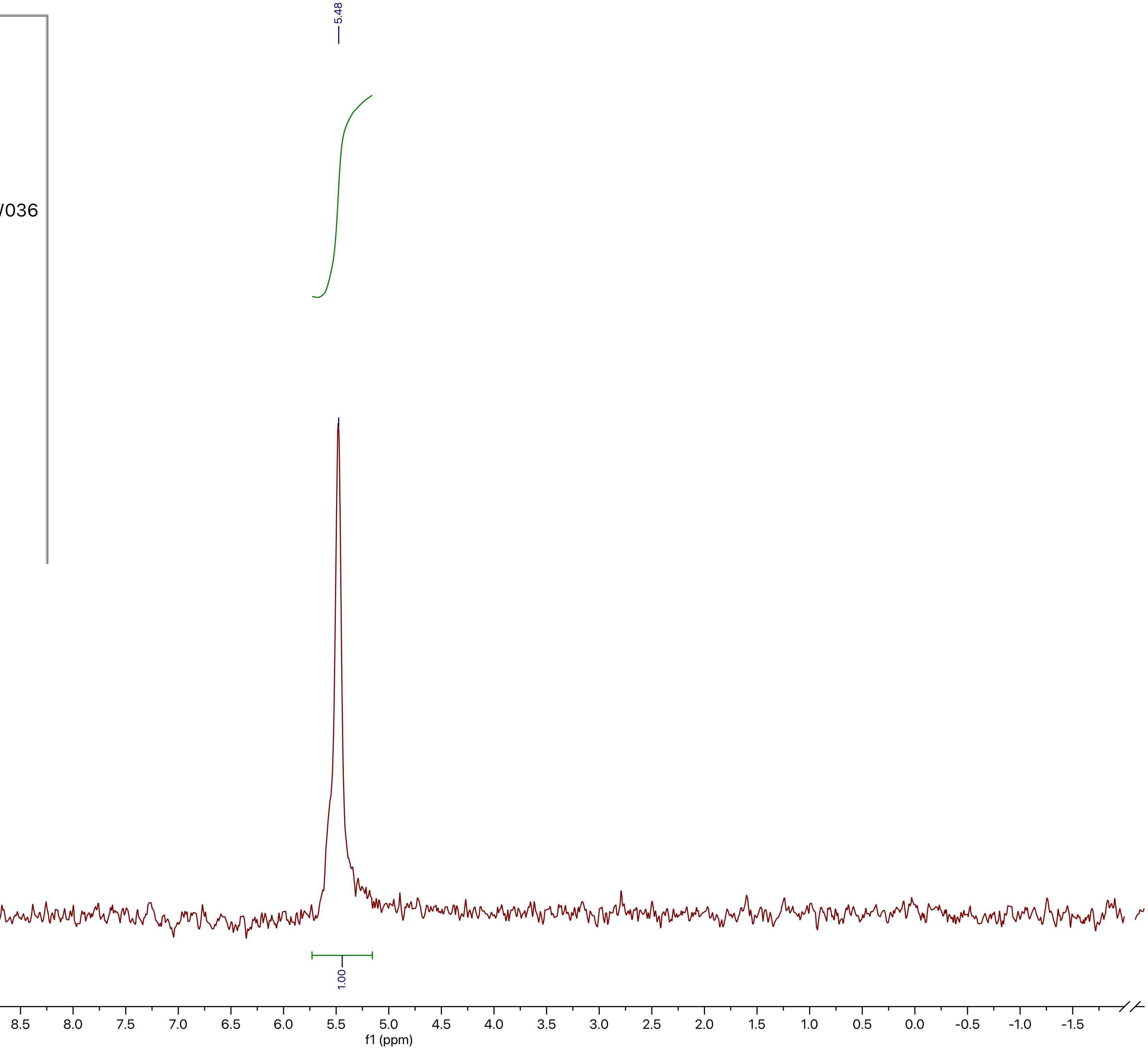

	Parameter	Value
1	Origin	Bruker BioSpin GmbH
2	Instrument	Avance
3	Solvent	CDCl3
4	Temperature	300.0
5	Pulse Sequence	zgpg30
6	Experiment	1D
7	Probe	Z151574_0073 (PI HR-BBO500S1-BBF/H/ D-5.0-Z SP)
8	Number of Scans	512
9	Receiver Gain	101.0
10	Relaxation Delay	2.0000
11	Pulse Width	9.0000
12	Spectrometer Frequency	125.79
13	Spectral Width	30120.5
14	Lowest Frequency	-2465.7
15	Nucleus	13C
16	Acquired Size	32768
17	Spectral Size	65536

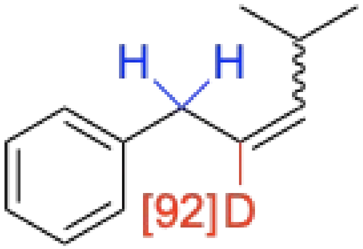

d_1-2b

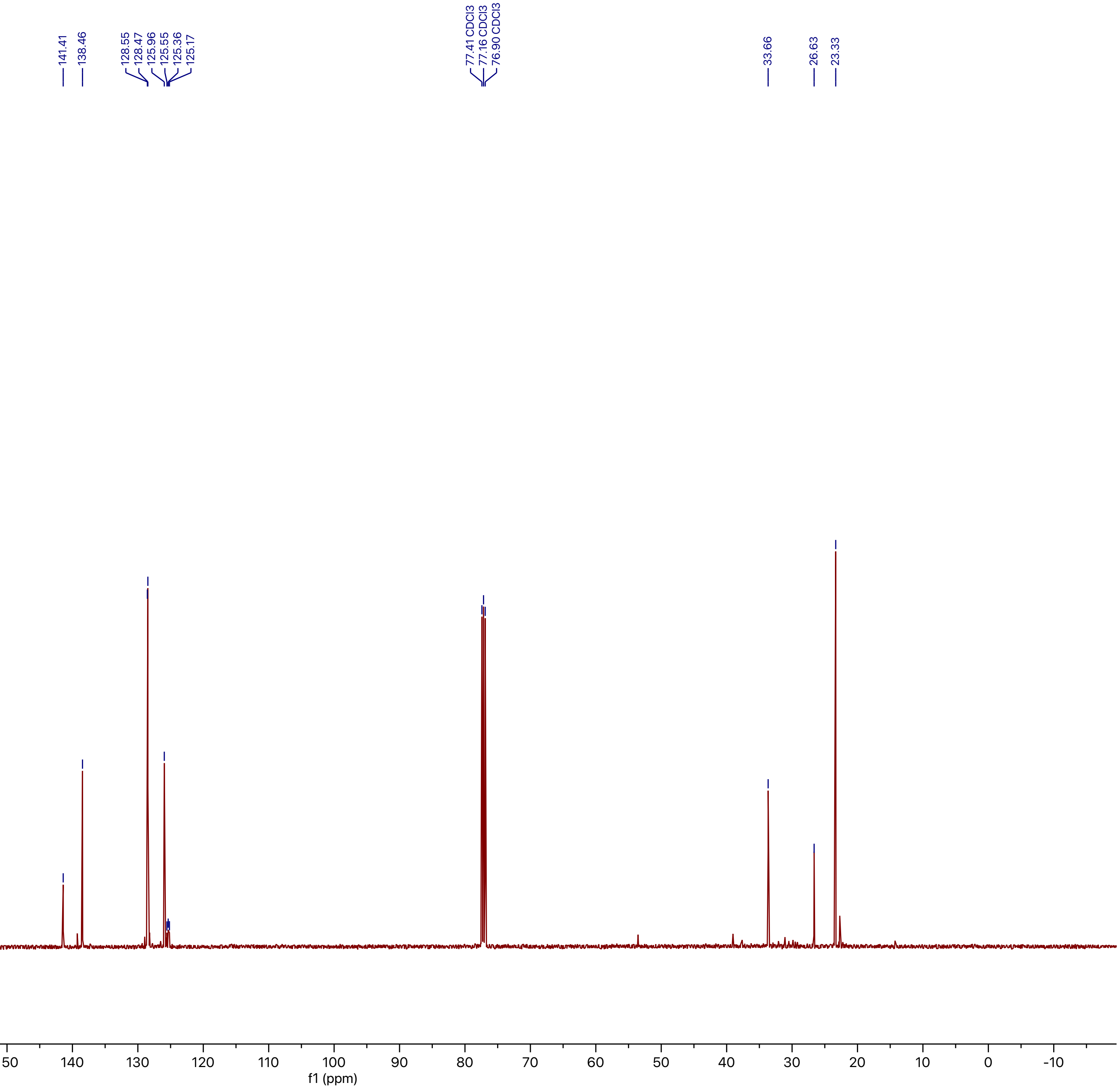

Parameter		Value
1	Origin	Bruker BioSpin GmbH
2	Instrument	Avance
3	Solvent	CDCl3
4	Temperature	300.0
5	Pulse Sequence	zg30
6	Experiment	1D
7	Probe	Z151574_0073 (PI HR-BBO500S1-BBF/ H/ D-5.0-Z SP)
8	Number of Scans	16
9	Receiver Gain	101.0
10	Relaxation Delay	1.0000
11	Pulse Width	8.0000
12	Spectrometer Frequency	500.21
13	Spectral Width	10000.0
14	Lowest Frequency	-1911.2
15	Nucleus	1H
16	Acquired Size	32768
17	Spectral Size	65536

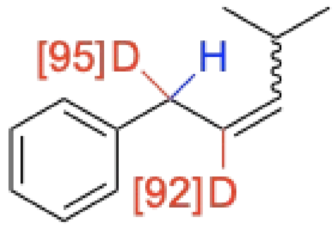

allyl-d₁, vinyl-d₁-2b

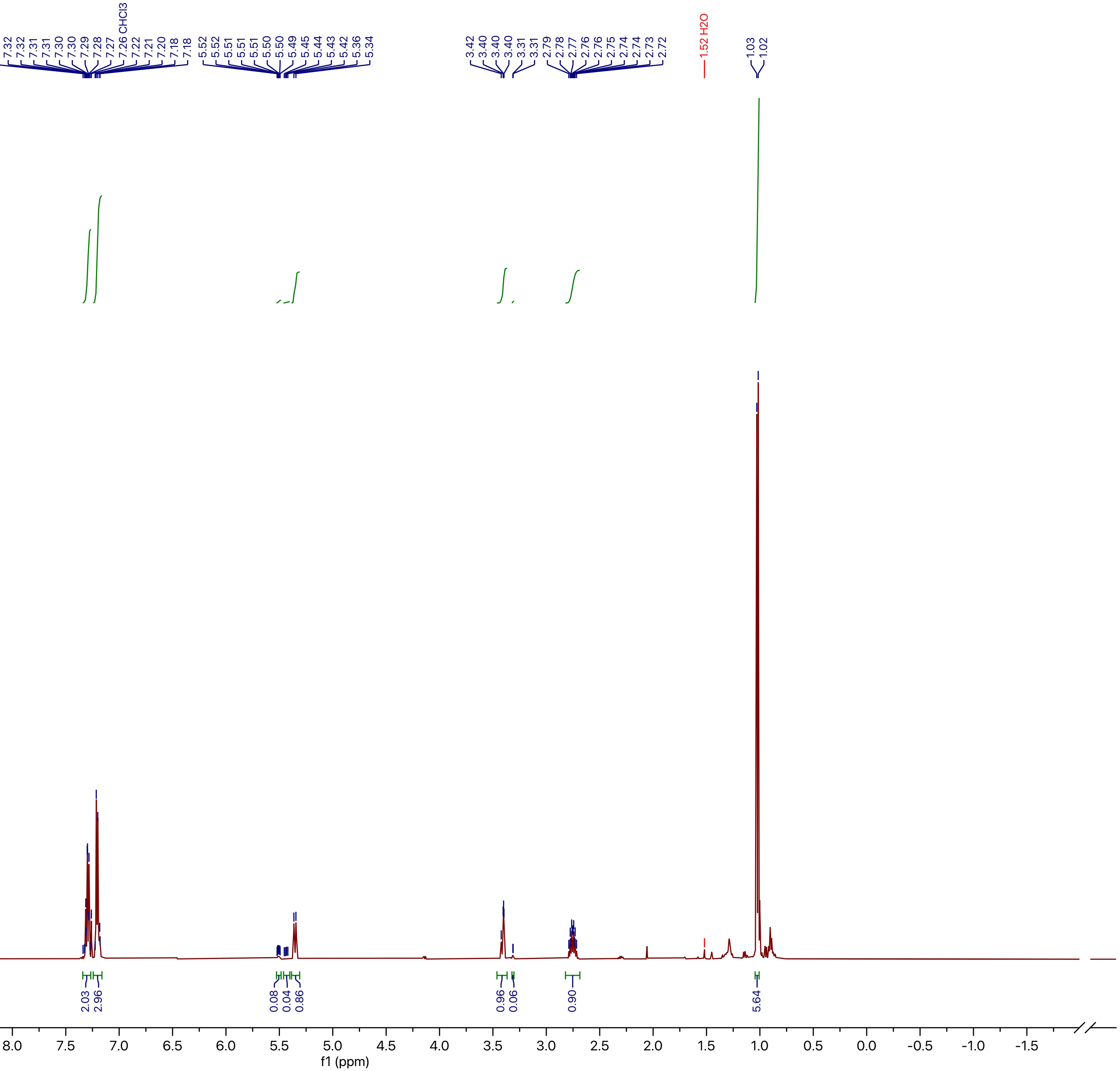

	Parameter	Value
1	Origin	Varian
2	Solvent	cdcl3
3	Temperature	25.0
4	Pulse Sequence	s2pul
5	Experiment	1D
6	Probe	OneNMR_W036
7	Number of Scans	32
8	Receiver Gain	20
9	Relaxation Delay	5.0000
10	Pulse Width	300.0000
11	Spectrometer Frequency	76.71
12	Spectral Width	1535.6
13	Lowest Frequency	-382.8
14	Nucleus	lk
15	Acquired Size	2048
16	Spectral Size	4096
17	Digital Resolution	0.37

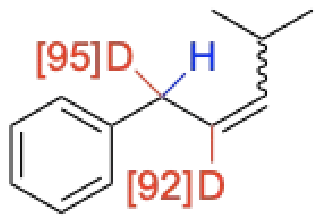

***allyl-d*$_1$, vinyl-*d*$_1$-2b**

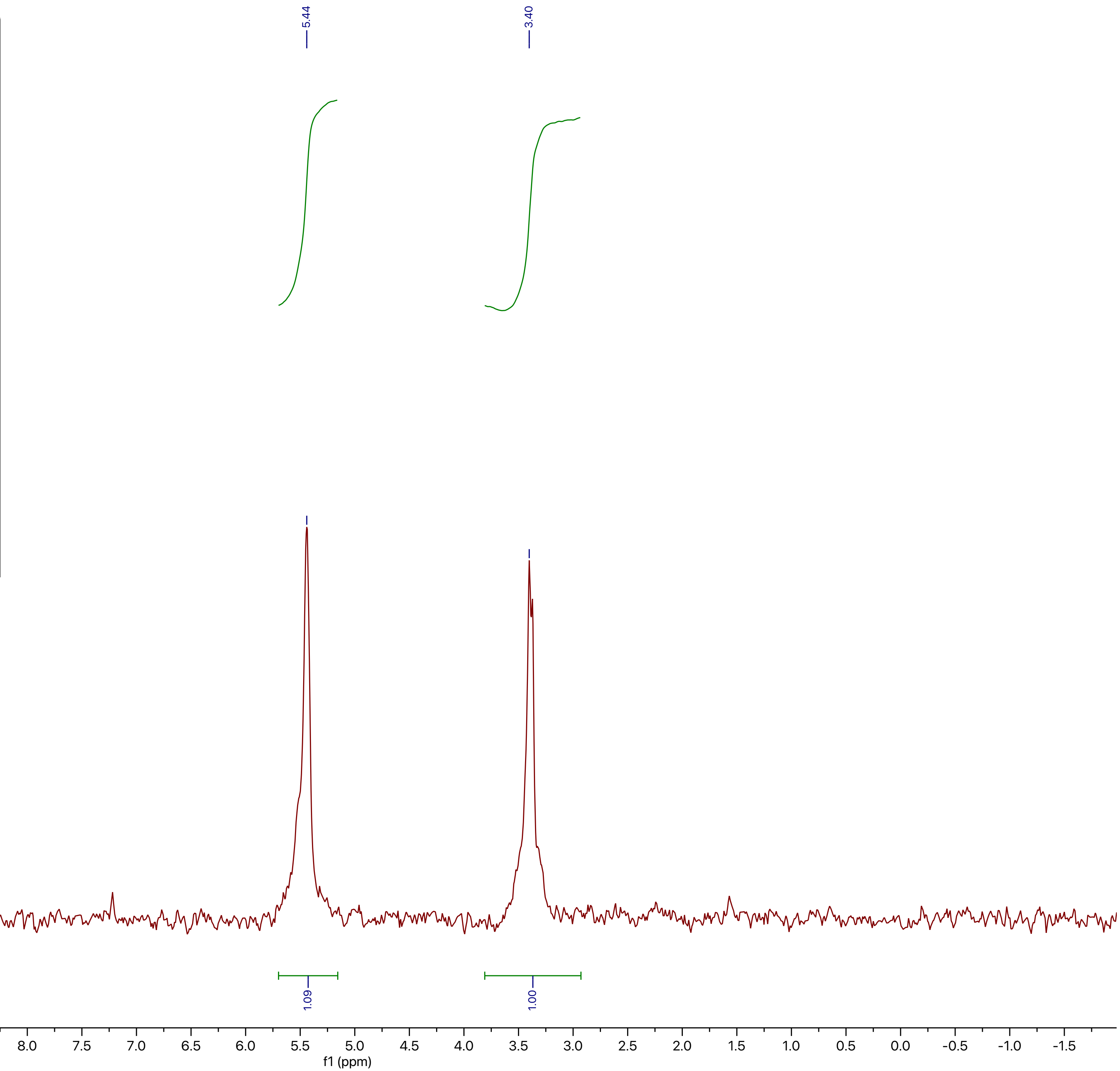

	Parameter	Value
1	Origin	Bruker BioSpin GmbH
2	Instrument	Avance
3	Solvent	CDCl3
4	Temperature	300.0
5	Pulse Sequence	zgpg30
6	Experiment	1D
7	Probe	Z151574_0073 (PI HR-BBO500S1-BBF/ H/ D-5.0-Z SP)
8	Number of Scans	512
9	Receiver Gain	101.0
10	Relaxation Delay	2.0000
11	Pulse Width	9.0000
12	Spectrometer Frequency	125.79
13	Spectral Width	30120.5
14	Lowest Frequency	-2465.0
15	Nucleus	13C
16	Acquired Size	32768
17	Spectral Size	65536

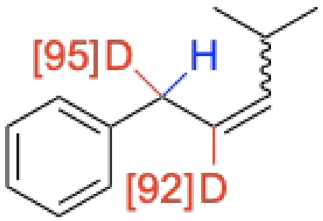

allyl-d_1, vinyl-d_1-2b

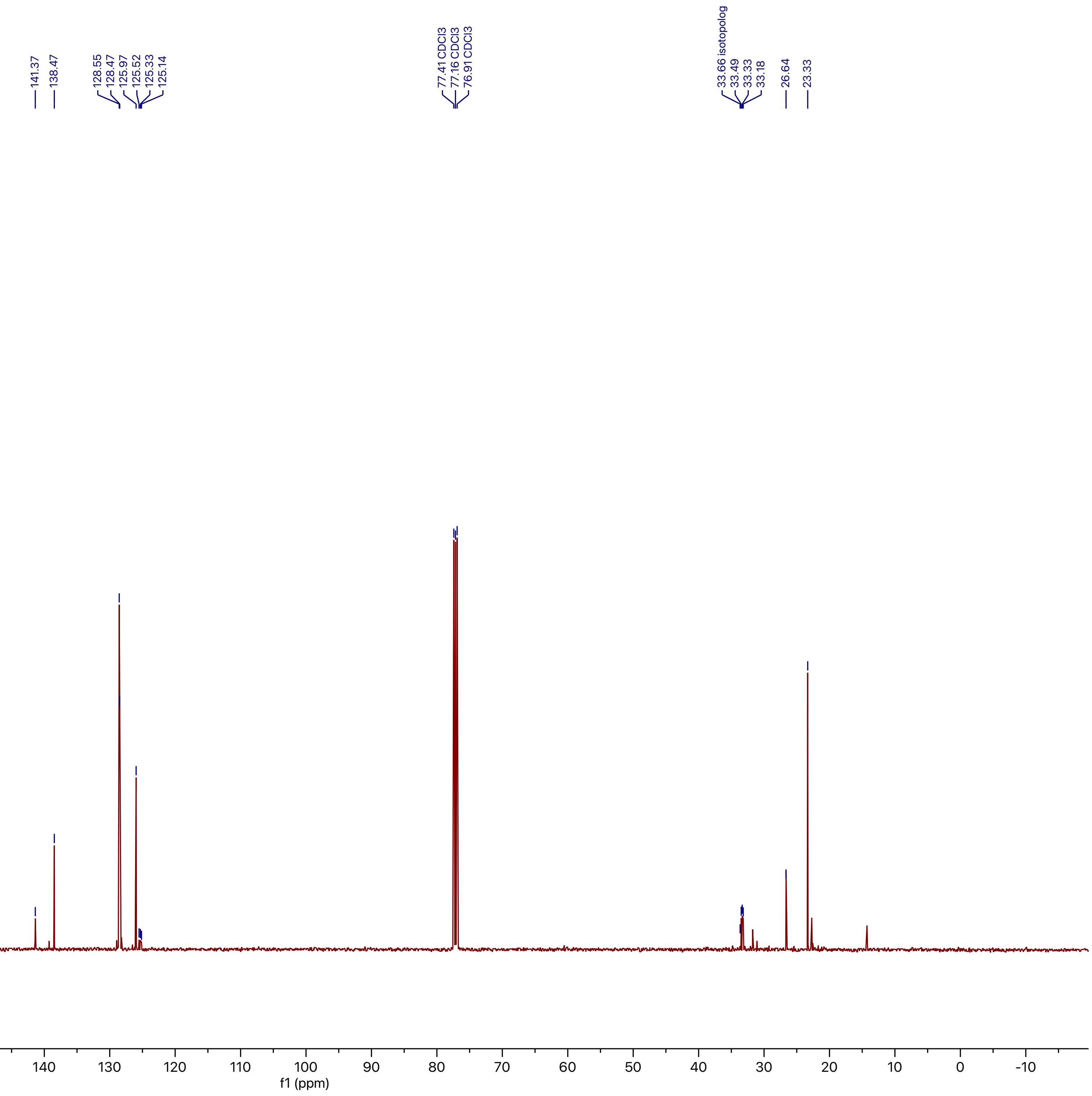

	Parameter	Value
1	Origin	Bruker BioSpin GmbH
2	Instrument	Avance
3	Solvent	CDCl3
4	Temperature	300.0
5	Pulse Sequence	zg30
6	Experiment	1D
7	Probe	Z151574_0073 (PI HR-BBO500S1-BBF/ H/ D-5.0-Z SP)
8	Number of Scans	64
9	Receiver Gain	61.2
10	Relaxation Delay	1.0000
11	Pulse Width	8.0000
12	Spectrometer Frequency	500.21
13	Spectral Width	10000.0
14	Lowest Frequency	-1922.7
15	Nucleus	1H
16	Acquired Size	32768
17	Spectral Size	65536

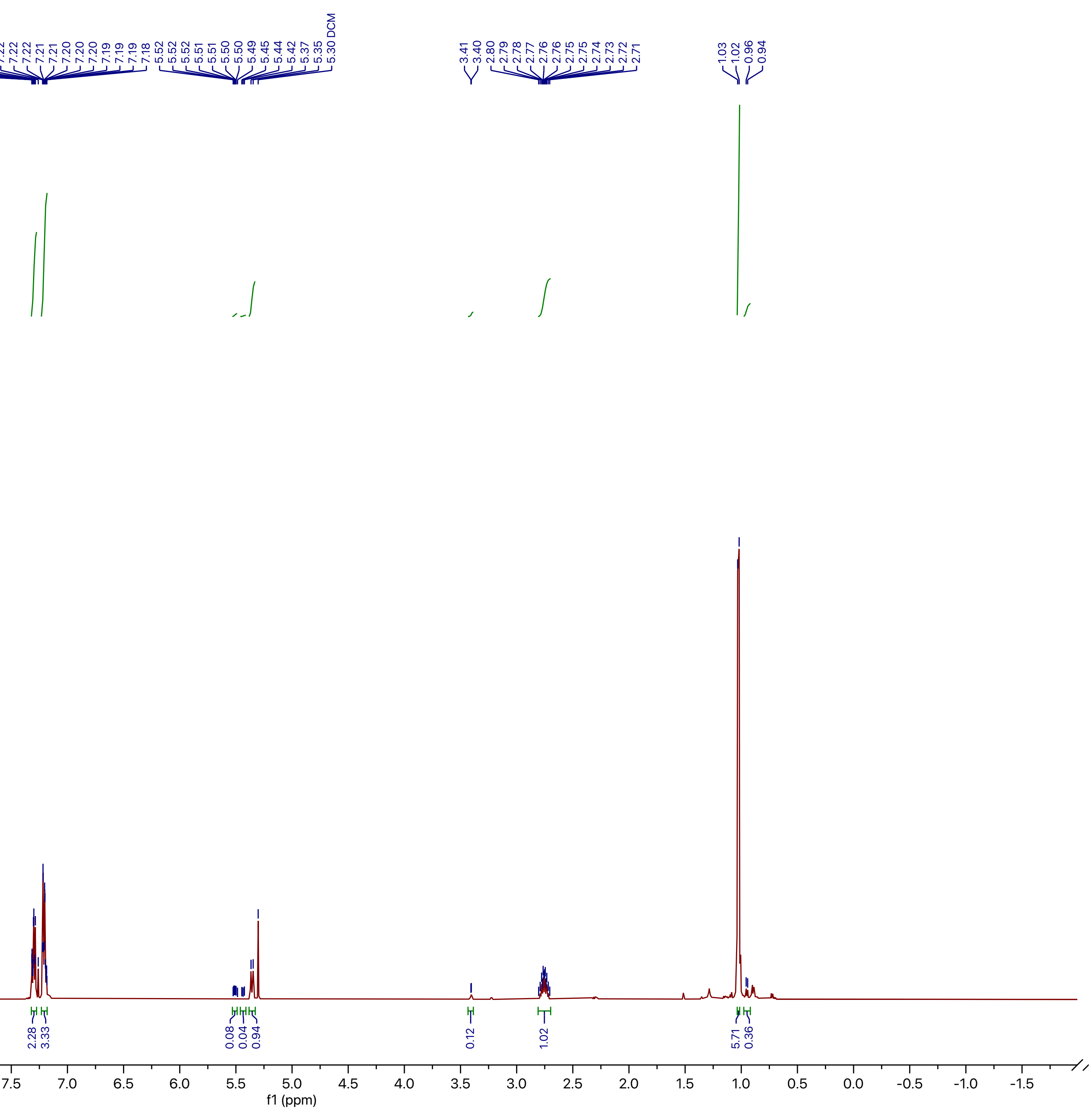

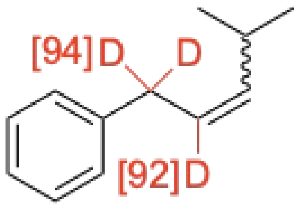

***allyl-d*$_2$, vinyl-*d*$_1$-2b**

	Parameter	Value
1	Origin	Varian
2	Solvent	cdcl3
3	Temperature	25.0
4	Pulse Sequence	s2pul
5	Experiment	1D
6	Probe	OneNMR_W036
7	Number of Scans	32
8	Receiver Gain	20
9	Relaxation Delay	5.0000
10	Pulse Width	300.0000
11	Spectrometer Frequency	76.71
12	Spectral Width	1535.6
13	Lowest Frequency	-380.5
14	Nucleus	lk
15	Acquired Size	2048
16	Spectral Size	4096
17	Digital Resolution	0.37

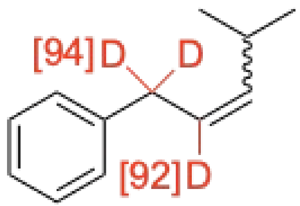

***allyl-d*$_2$, vinyl-*d*$_1$-2b**

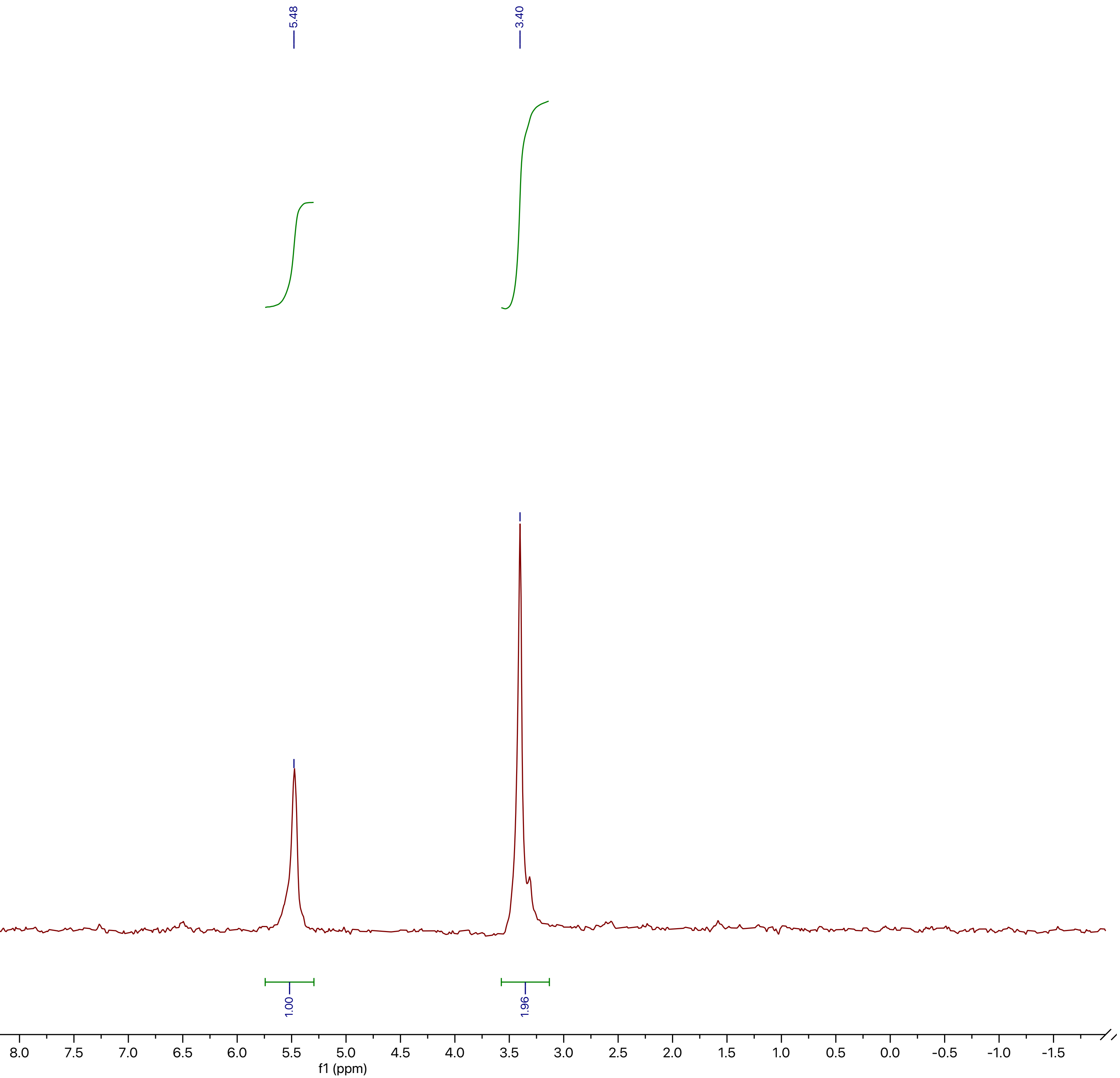

	Parameter	Value
1	Origin	Bruker BioSpin GmbH
2	Instrument	Avance
3	Solvent	CDCl3
4	Temperature	300.0
5	Pulse Sequence	zgpg30
6	Experiment	1D
7	Probe	Z151574_0073 (PI HR-BBO500S1-BBF/ H/ D-5.0-Z SP)
8	Number of Scans	512
9	Receiver Gain	101.0
10	Relaxation Delay	2.0000
11	Pulse Width	9.0000
12	Spectrometer Frequency	125.79
13	Spectral Width	30120.5
14	Lowest Frequency	-2482.4
15	Nucleus	13C
16	Acquired Size	32768
17	Spectral Size	65536

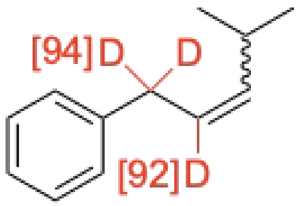

allyl-d_2, vinyl-d_1-2b

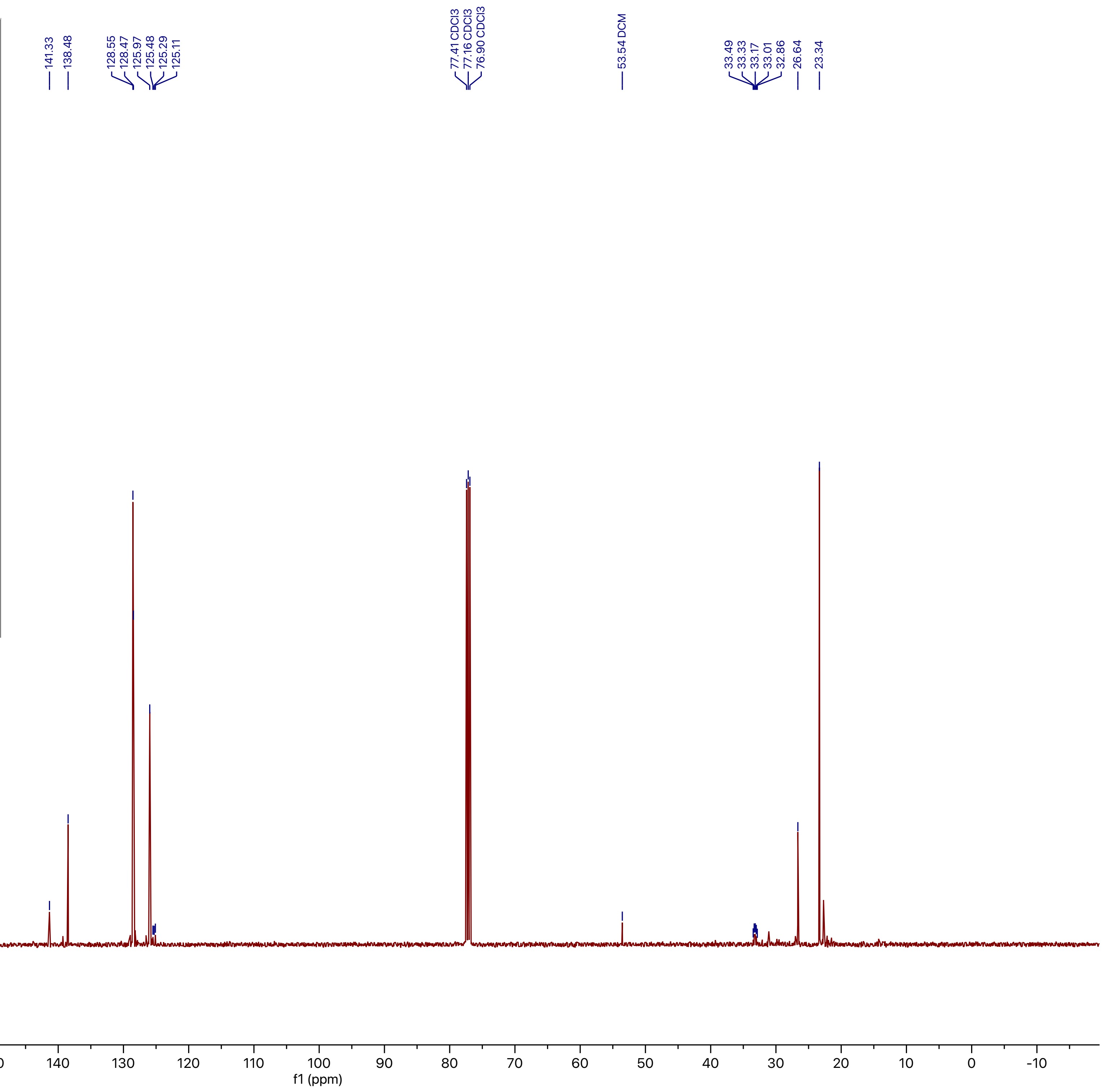

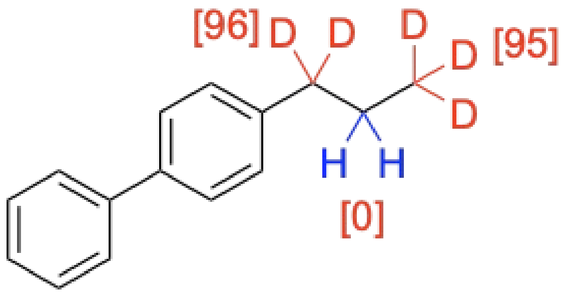

***d*₅-4d**

	Parameter	Value
1	Origin	Bruker BioSpin GmbH
2	Instrument	Avance
3	Solvent	CDCl3
4	Temperature	300.0
5	Pulse Sequence	zg30
6	Experiment	1D
7	Probe	Z151574_0073 (PI HR-BBO500S1-BBF/ H/ D-5.0-Z SP)
8	Number of Scans	16
9	Receiver Gain	101.0
10	Relaxation Delay	1.0000
11	Pulse Width	8.0000
12	Spectrometer Frequency	500.21
13	Spectral Width	10000.0
14	Lowest Frequency	-1923.5
15	Nucleus	1H
16	Acquired Size	32768
17	Spectral Size	65536

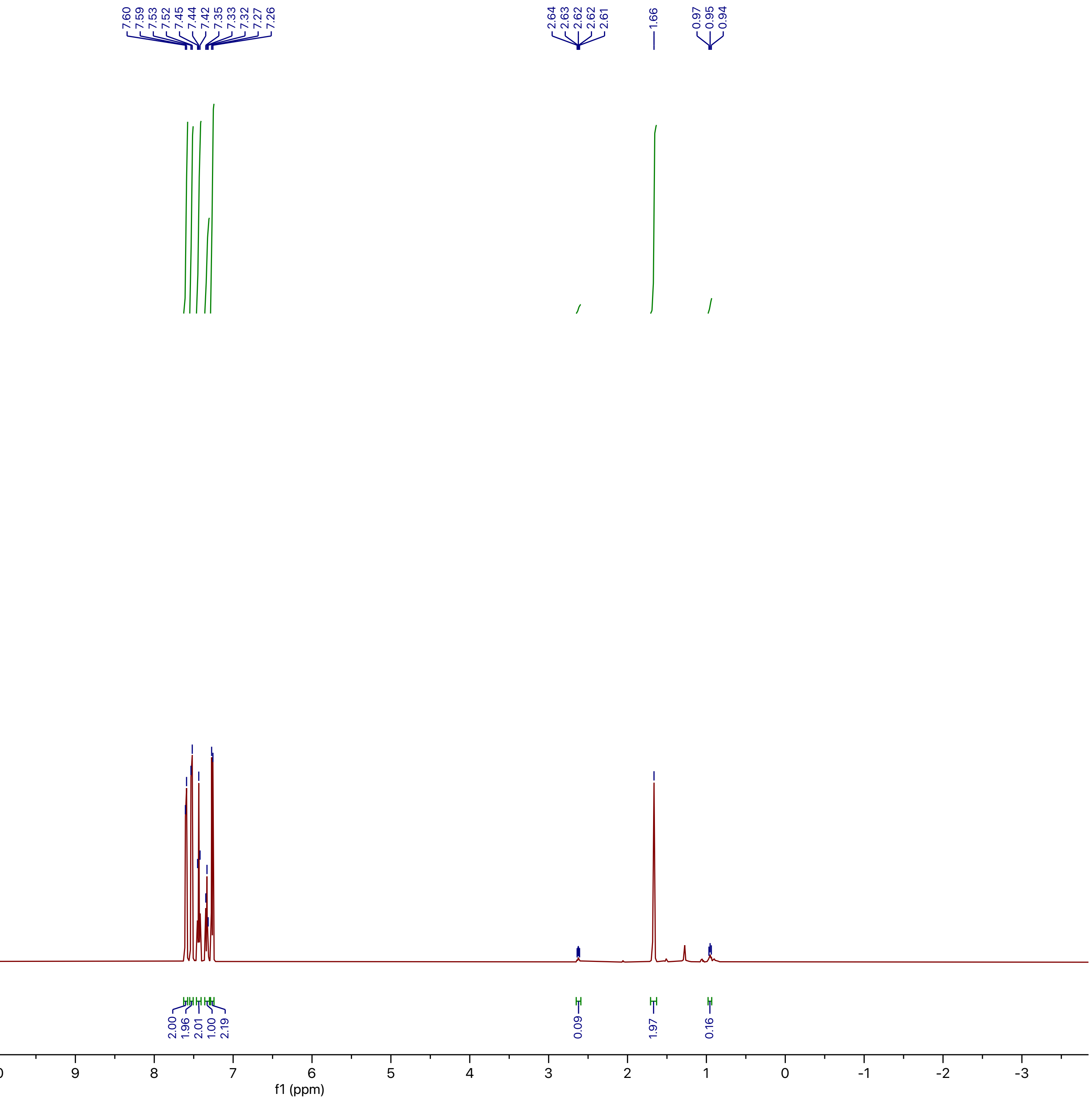

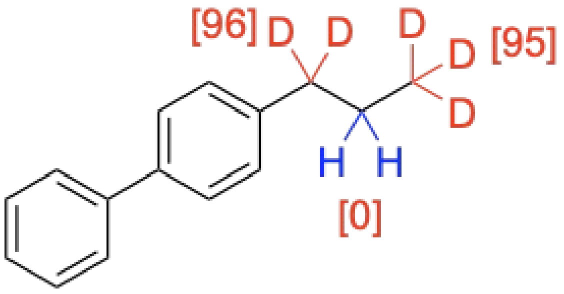

***d*$_5$-4d**

	Parameter	Value
1	Origin	Varian
2	Solvent	cdcl3
3	Temperature	25.0
4	Pulse Sequence	s2pul
5	Experiment	1D
6	Probe	OneNMR_W036
7	Number of Scans	32
8	Receiver Gain	20
9	Relaxation Delay	5.0000
10	Pulse Width	300.0000
11	Spectrometer Frequency	76.71
12	Spectral Width	1535.6
13	Lowest Frequency	-384.2
14	Nucleus	lk
15	Acquired Size	2048
16	Spectral Size	4096
17	Digital Resolution	0.37

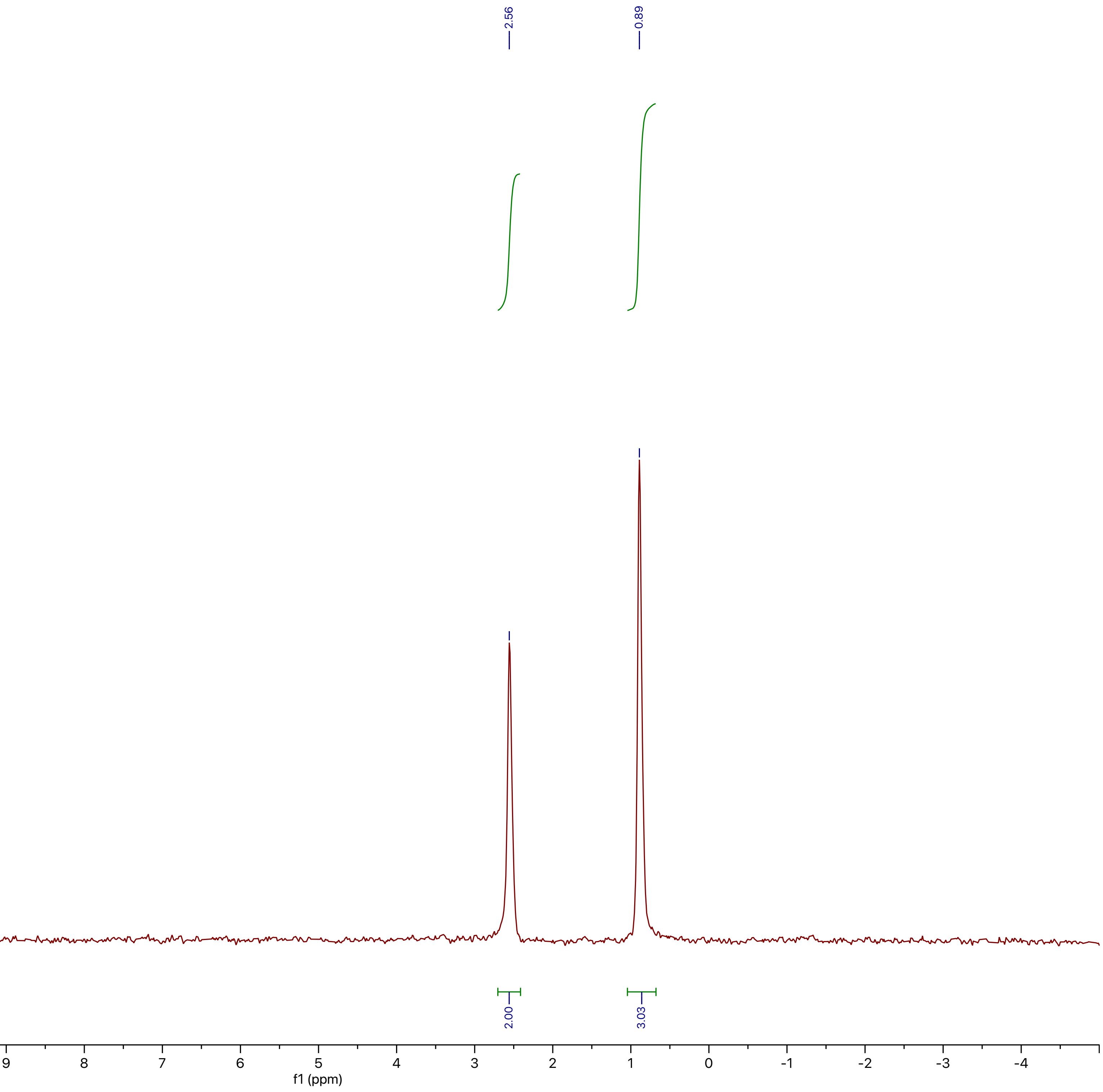

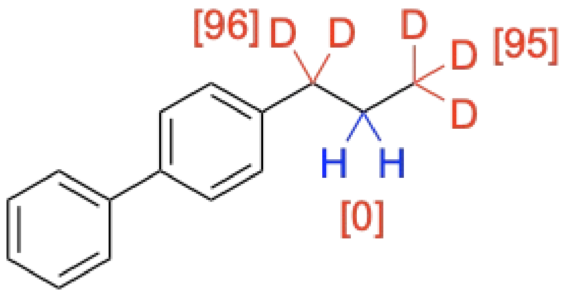

***d*$_5$-4d**

Parameter		Value
1	Origin	Bruker BioSpin GmbH
2	Instrument	Avance
3	Solvent	CDCl3
4	Temperature	300.0
5	Pulse Sequence	zgpg30
6	Experiment	1D
7	Probe	Z151574_0073 (PI HR-BBO500S1-BBF/ H/ D-5.0-Z SP)
8	Number of Scans	2048
9	Receiver Gain	101.0
10	Relaxation Delay	10.0000
11	Pulse Width	9.0000
12	Spectrometer Frequency	125.79
13	Spectral Width	30120.5
14	Lowest Frequency	-2465.7
15	Nucleus	13C
16	Acquired Size	32768
17	Spectral Size	65536

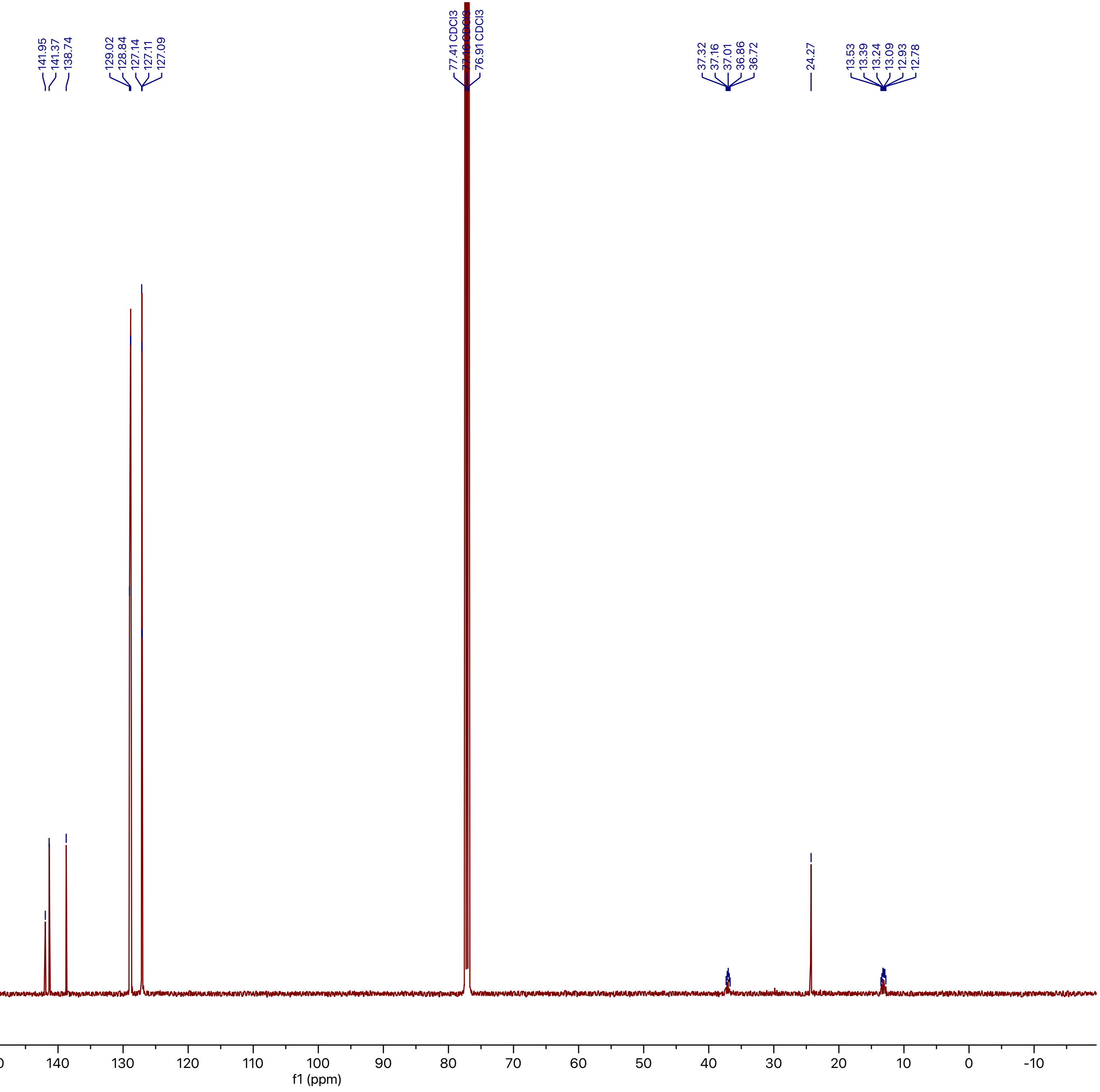

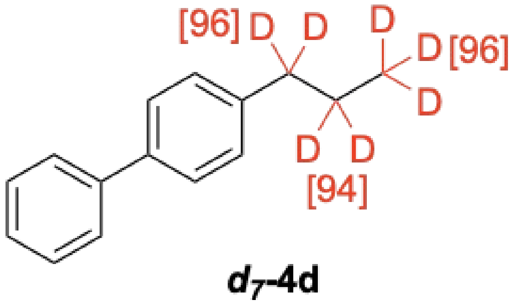

***d*$_7$-4d**

	Parameter	Value
1	Origin	Bruker BioSpin GmbH
2	Instrument	Avance
3	Solvent	CDCl3
4	Temperature	298.0
5	Pulse Sequence	zg30
6	Experiment	1D
7	Probe	Z151574_0073 (PI HR-BBO500S1-BBF/ H/ D-5.0-Z SP)
8	Number of Scans	32
9	Receiver Gain	101.0
10	Relaxation Delay	10.0000
11	Pulse Width	8.0000
12	Spectrometer Frequency	500.21
13	Spectral Width	10000.0
14	Lowest Frequency	-1924.6
15	Nucleus	1H
16	Acquired Size	32768
17	Spectral Size	65536

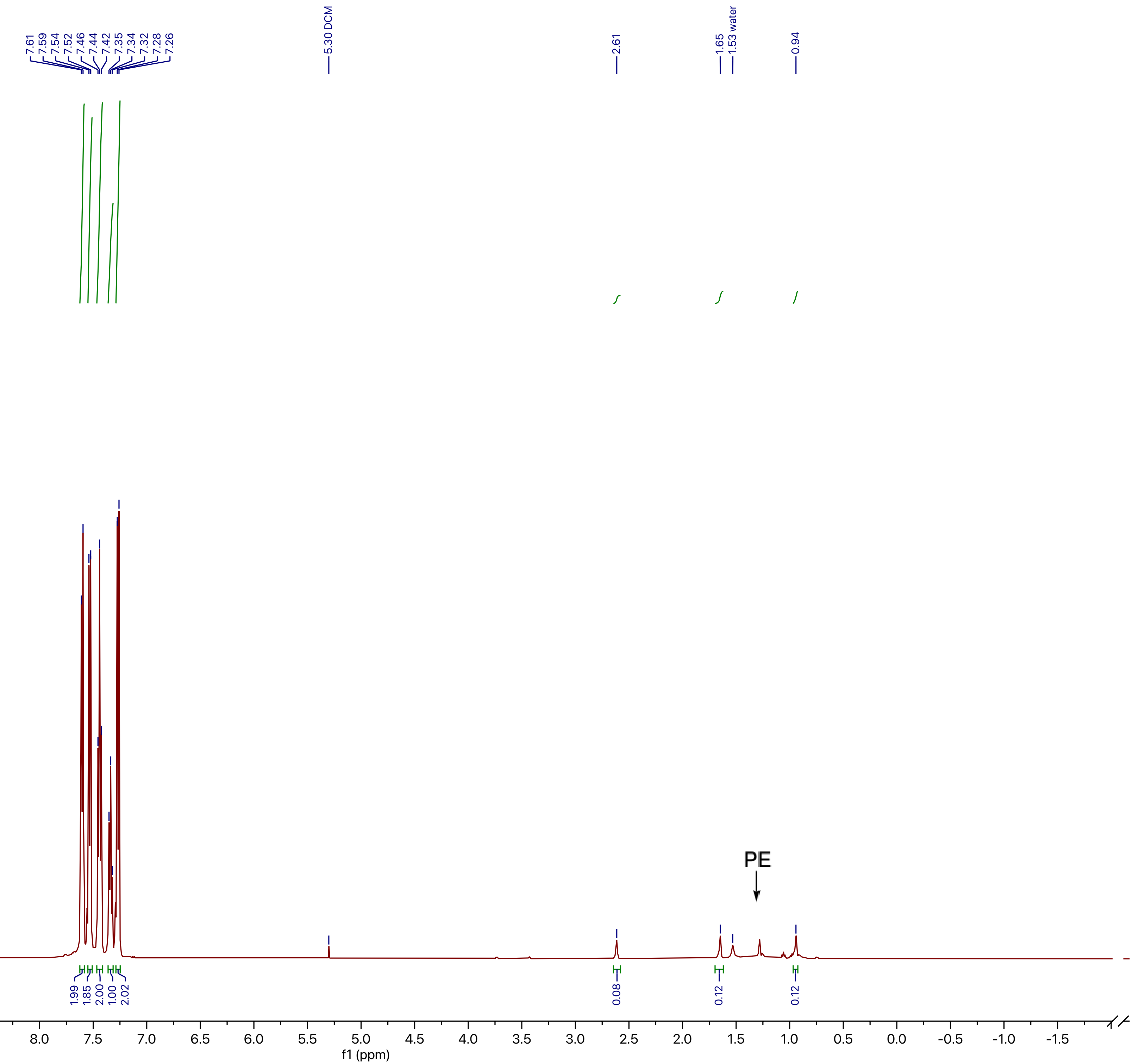

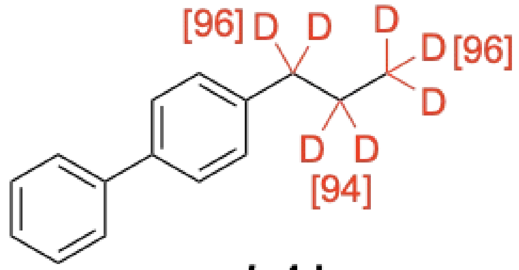

d$_7$-**4d**

	Parameter	Value
1	Origin	Varian
2	Solvent	cdcl3
3	Temperature	25.0
4	Pulse Sequence	s2pul
5	Experiment	1D
6	Probe	OneNMR_W036
7	Number of Scans	64
8	Receiver Gain	20
9	Relaxation Delay	2.0000
10	Pulse Width	300.0000
11	Spectrometer Frequency	76.71
12	Spectral Width	1535.6
13	Lowest Frequency	-384.2
14	Nucleus	lk
15	Acquired Size	2048
16	Spectral Size	4096
17	Digital Resolution	0.37

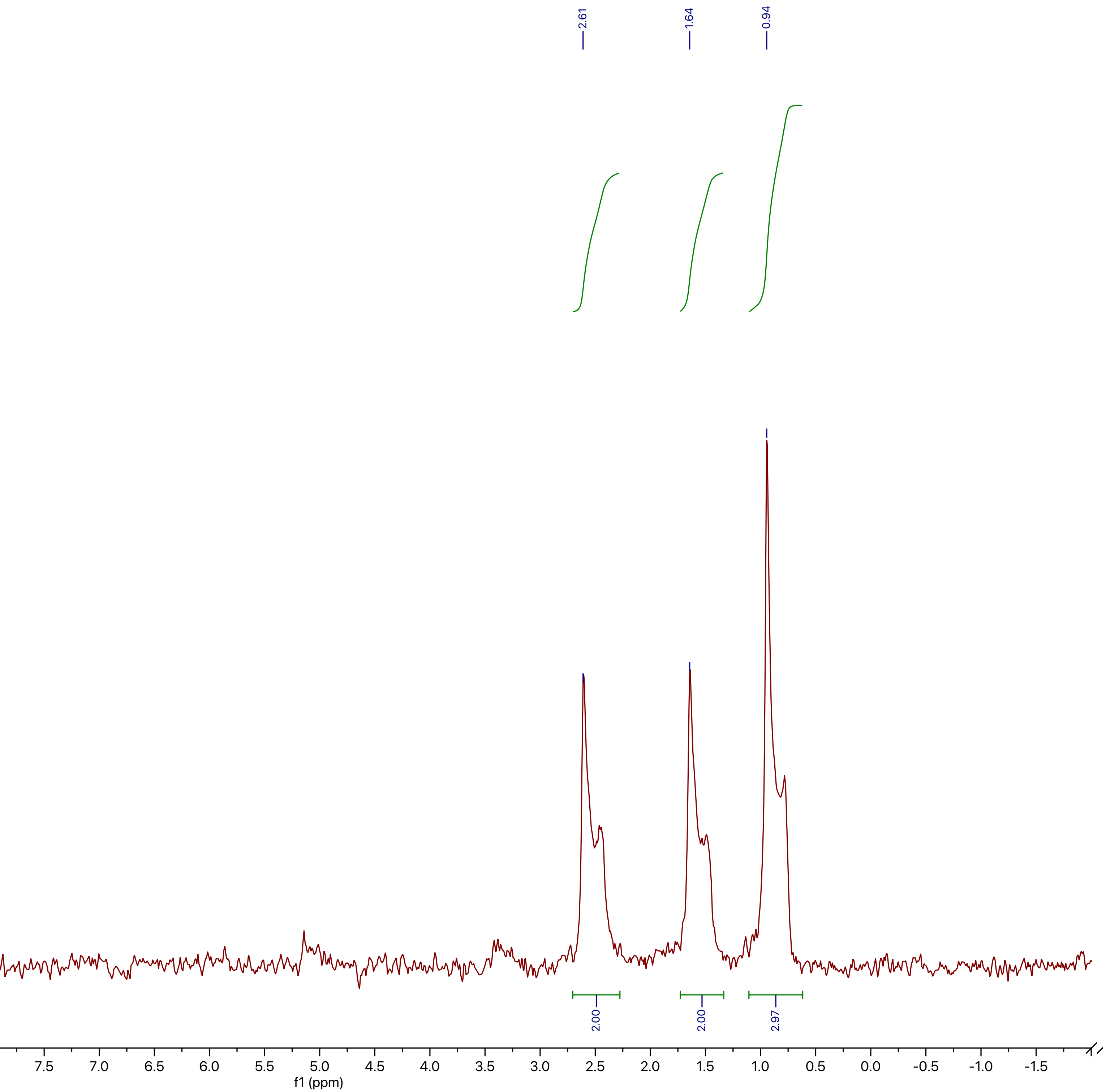

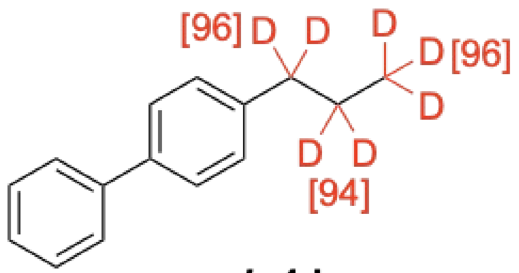

d$_7$-4d

Parameter		Value
1	Origin	Bruker BioSpin GmbH
2	Instrument	Avance
3	Solvent	CDCl3
4	Temperature	298.0
5	Pulse Sequence	zgpg30
6	Experiment	1D
7	Probe	Z151574_0073 (PI HR-BBO500S1-BBF/ H/ D-5.0-Z SP)
8	Number of Scans	4000
9	Receiver Gain	101.0
10	Relaxation Delay	10.0000
11	Pulse Width	9.0000
12	Spectrometer Frequency	125.79
13	Spectral Width	30120.5
14	Lowest Frequency	-2467.7
15	Nucleus	13C
16	Acquired Size	32768
17	Spectral Size	65536

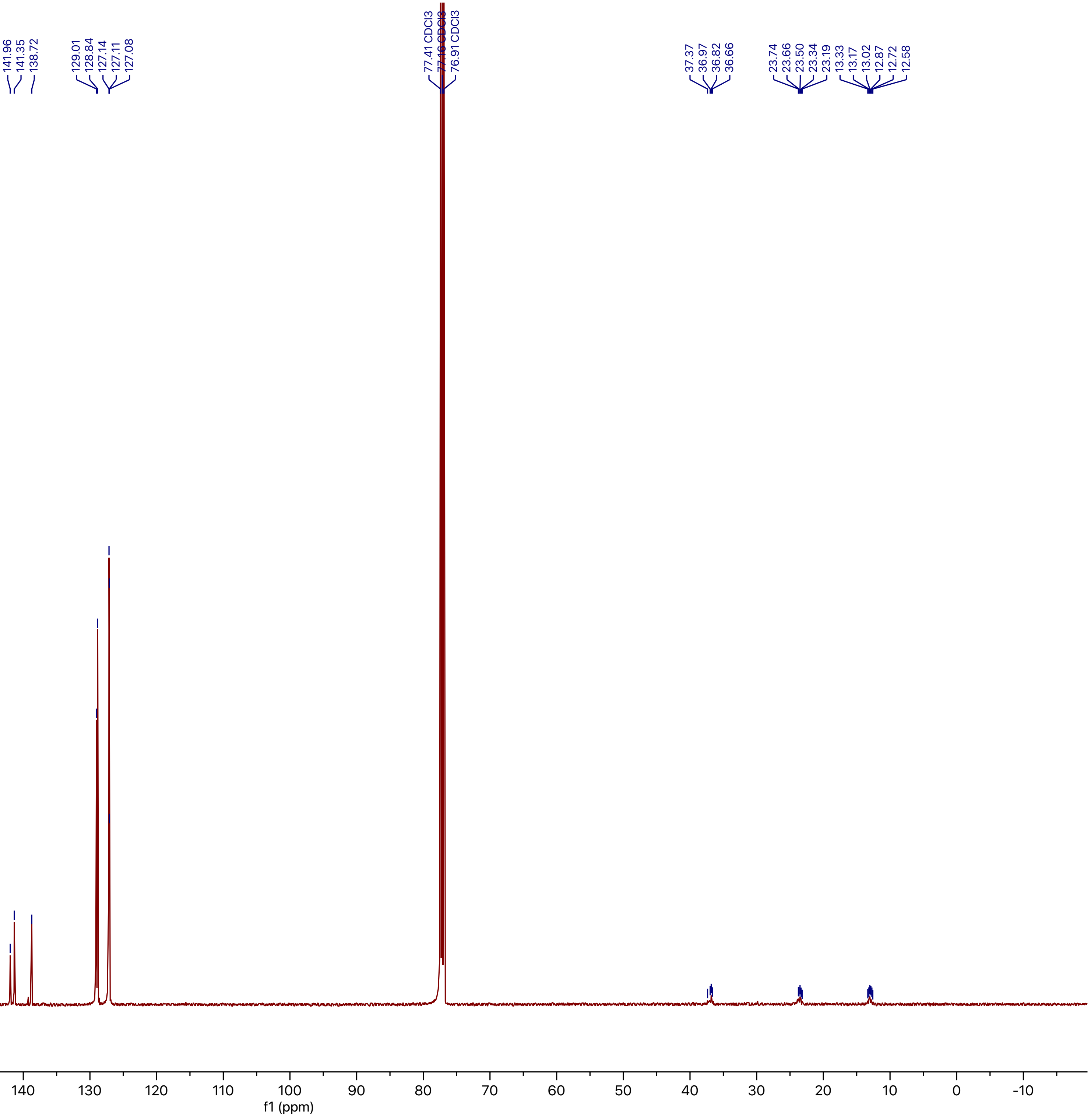

	Parameter	Value
1	Origin	Bruker BioSpin GmbH
2	Instrument	Avance
3	Solvent	CDCl3
4	Temperature	298.0
5	Pulse Sequence	zg30
6	Experiment	1D
7	Probe	Z163739_0400 (PI HR-BBO400S1-BBF/H/ D-5.0-Z SP)
8	Number of Scans	16
9	Receiver Gain	101.0
10	Relaxation Delay	1.0000
11	Pulse Width	8.0000
12	Spectrometer Frequency	400.13
13	Spectral Width	8196.7
14	Lowest Frequency	-1638.3
15	Nucleus	1H
16	Acquired Size	32768
17	Spectral Size	65536

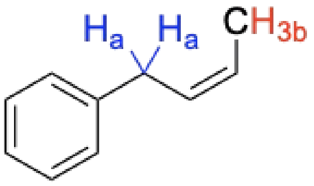

(Z)-d_0-2a

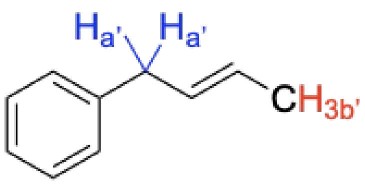

(E)-d_0-2a

Z/E ~ 12:1

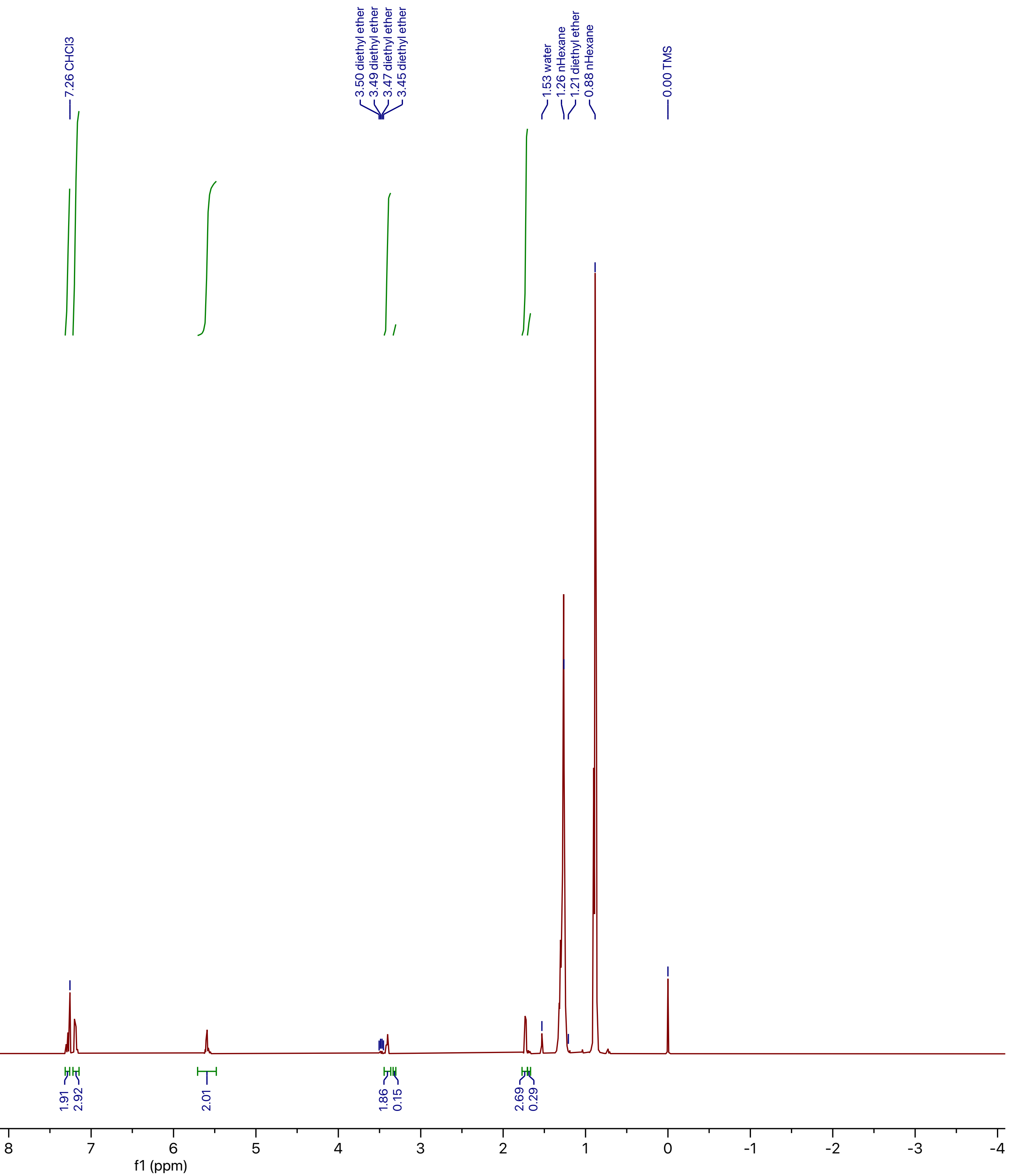

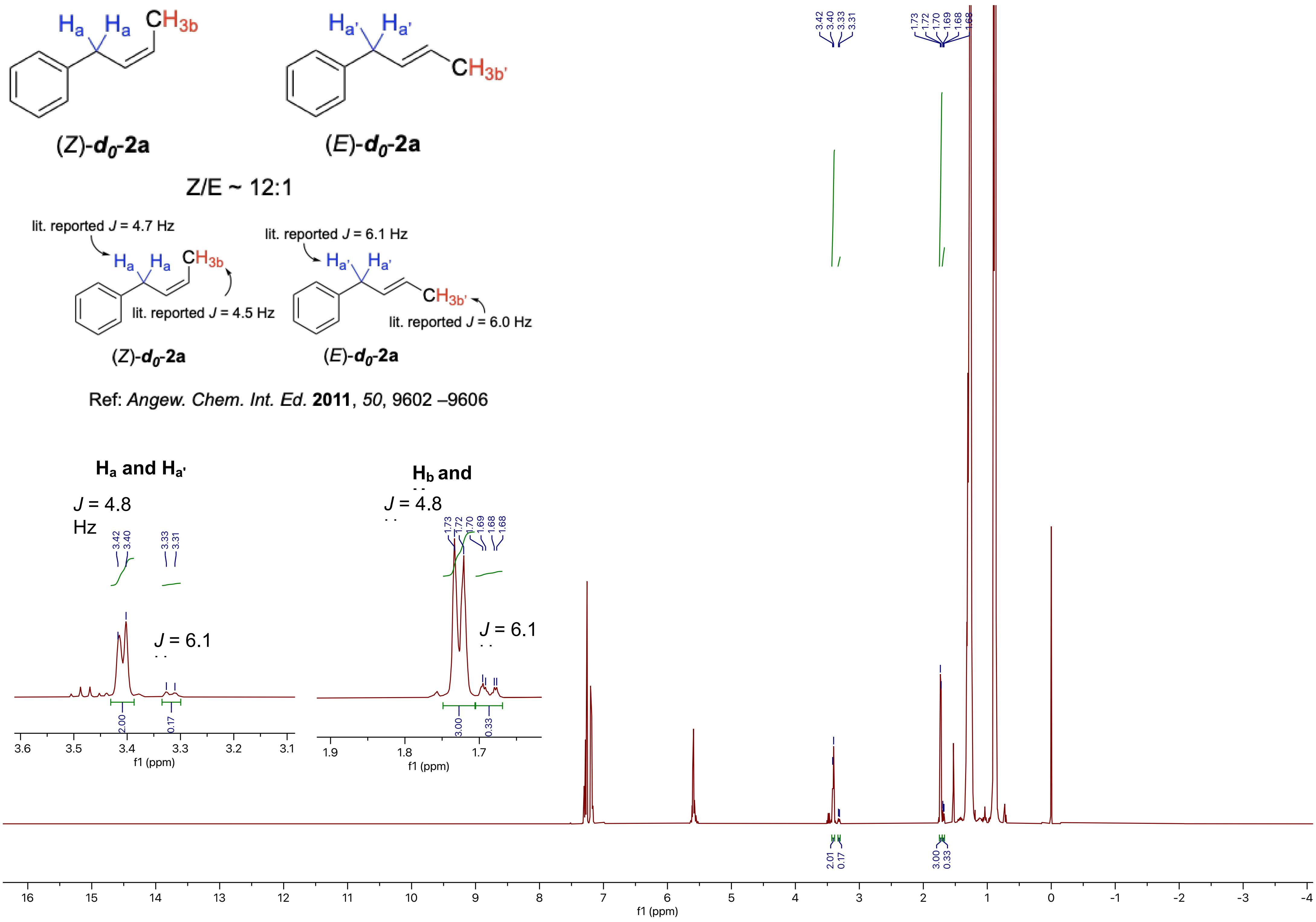

(Z)-d0-2a
(E)-d0-2a
Z/E ~ 12:1
lit. reported J = 4.7 Hz
lit. reported J = 4.5 Hz
lit. reported J = 6.1 Hz
lit. reported J = 6.0 Hz
Ref: Angew. Chem. Int. Ed. 2011, 50, 9602 –9606
Ha and Ha'
J = 4.8 Hz
J = 6.1
Hb and
J = 4.8
J = 6.1
f1 (ppm)

	Parameter	Value
1	Origin	Bruker BioSpin GmbH
2	Instrument	Avance
3	Solvent	CDCl3
4	Temperature	298.0
5	Pulse Sequence	zg30
6	Experiment	1D
7	Probe	Z163739_0400 (PI HR-BBO400S1-BBF/ H/ D-5.0-Z SP)
8	Number of Scans	16
9	Receiver Gain	95.1
10	Relaxation Delay	1.0000
11	Pulse Width	8.0000
12	Spectrometer Frequency	400.13
13	Spectral Width	8196.7
14	Lowest Frequency	-1636.3
15	Nucleus	1H
16	Acquired Size	32768
17	Spectral Size	65536

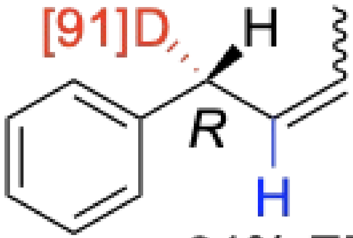

91% EE

(*R*)-**2a** with **L1**

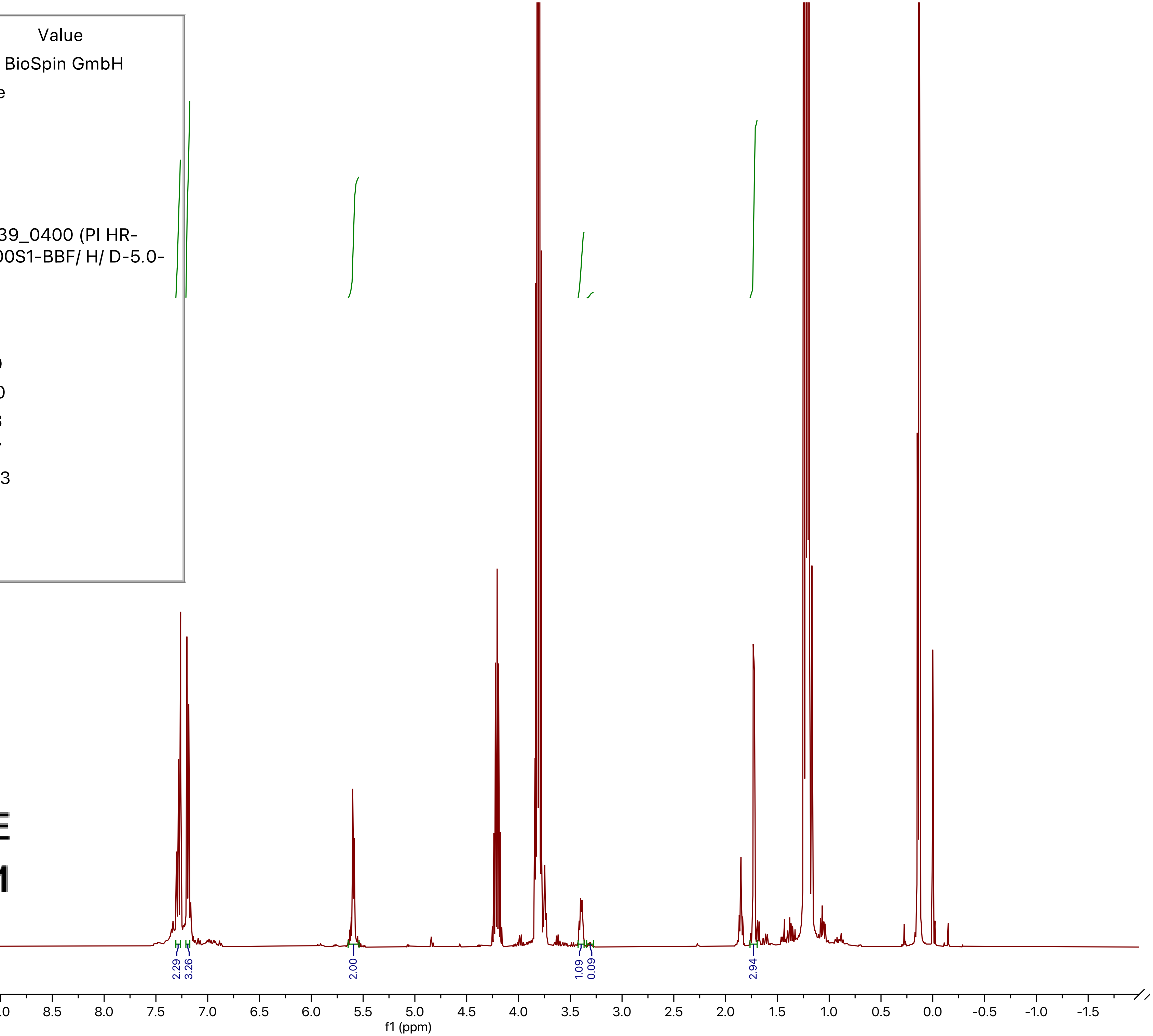

	Parameter	Value
1	Origin	Bruker BioSpin GmbH
2	Instrument	Avance
3	Solvent	CDCl3
4	Temperature	298.0
5	Pulse Sequence	zg30
6	Experiment	1D
7	Probe	Z163739_0400 (PI HR-BBO400S1-BBF/ H/ D-5.0-Z SP)
8	Number of Scans	16
9	Receiver Gain	80.5
10	Relaxation Delay	1.0000
11	Pulse Width	8.0000
12	Spectrometer Frequency	400.13
13	Spectral Width	8196.7
14	Lowest Frequency	-1635.8
15	Nucleus	1H
16	Acquired Size	32768
17	Spectral Size	65536

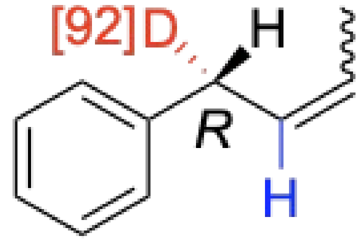

72% EE

(R)-**2a** with (S)-**L2**

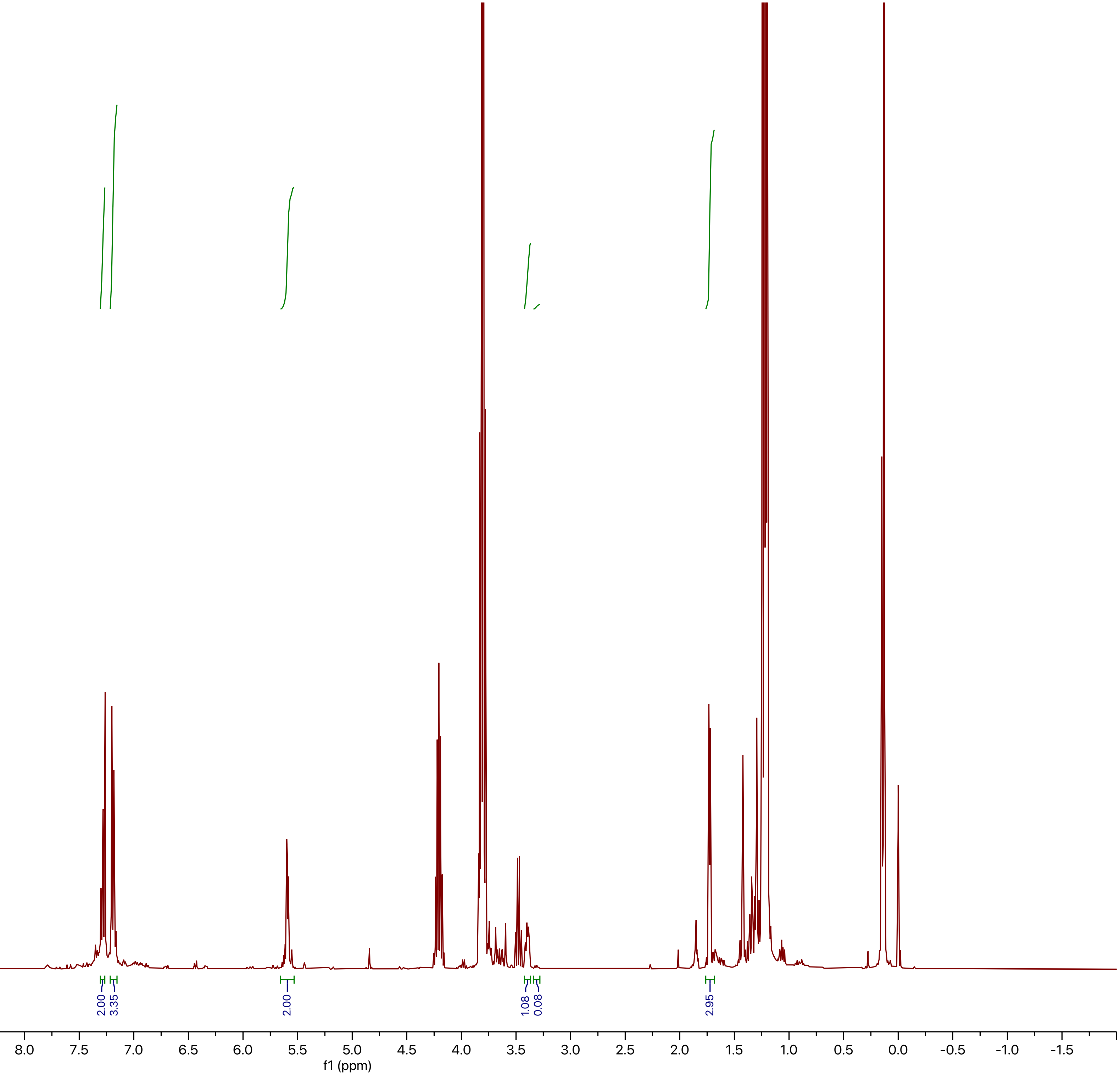

	Parameter	Value
1	Origin	Bruker BioSpin GmbH
2	Instrument	Avance
3	Solvent	CDCl3
4	Temperature	298.0
5	Pulse Sequence	zg30
6	Experiment	1D
7	Probe	Z163739_0400 (PI HR-BBO400S1-BBF/ H/ D-5.0-Z SP)
8	Number of Scans	16
9	Receiver Gain	101.0
10	Relaxation Delay	1.0000
11	Pulse Width	8.0000
12	Spectrometer Frequency	400.13
13	Spectral Width	8196.7
14	Lowest Frequency	-1688.5
15	Nucleus	1H
16	Acquired Size	32768
17	Spectral Size	65536

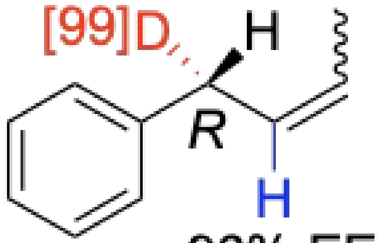

96% EE

(*R*)-**2a** with (*R*)-**L2**

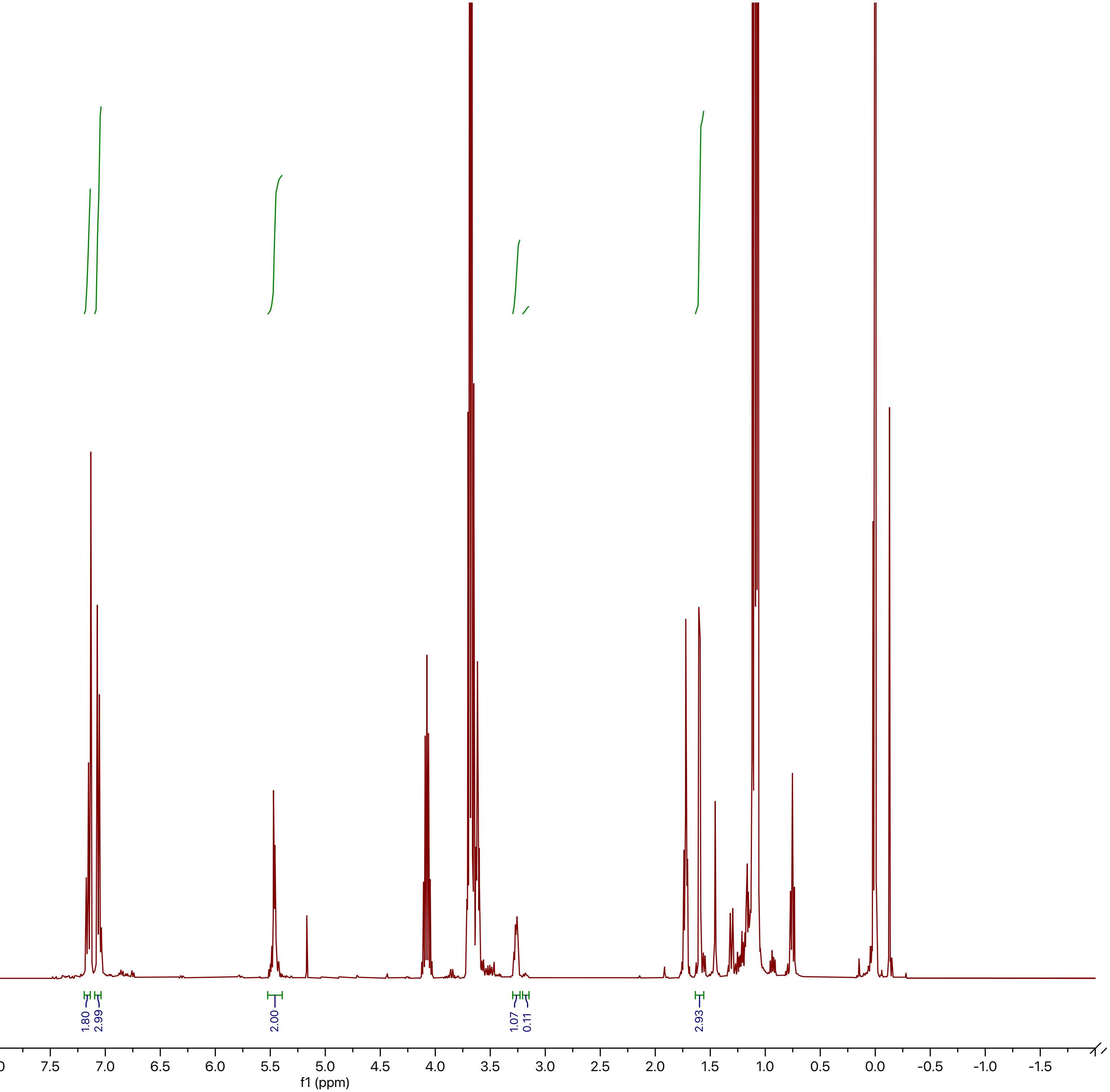

	Parameter	Value
1	Origin	Bruker BioSpin GmbH
2	Instrument	Avance
3	Solvent	CDCl3
4	Temperature	298.0
5	Pulse Sequence	zg30
6	Experiment	1D
7	Probe	Z163739_0400 (PI HR-BBO400S1-BBF/ H/ D-5.0-Z SP)
8	Number of Scans	16
9	Receiver Gain	101.0
10	Relaxation Delay	1.0000
11	Pulse Width	8.0000
12	Spectrometer Frequency	400.13
13	Spectral Width	8196.7
14	Lowest Frequency	-1688.3
15	Nucleus	1H
16	Acquired Size	32768

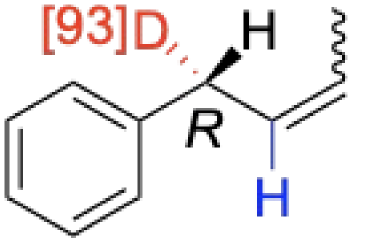

87% EE

(*R*)-2a with **L3**

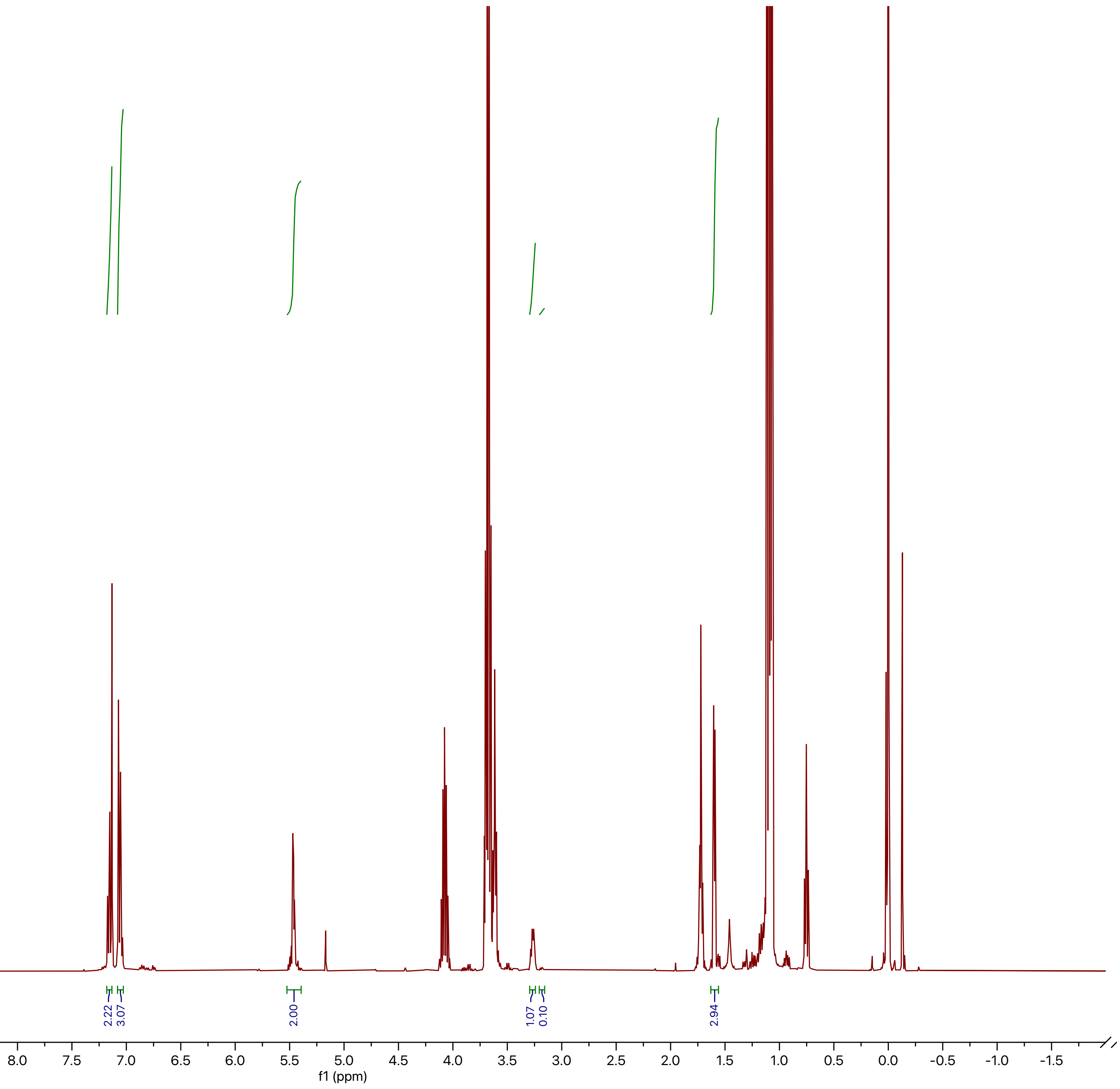

	Parameter	Value
1	Origin	Bruker BioSpin GmbH
2	Instrument	Avance
3	Solvent	CDCl3
4	Temperature	298.0
5	Pulse Sequence	zg30
6	Experiment	1D
7	Probe	Z163739_0400 (PI HR-BBO400S1-BBF/ H/ D-5.0-Z SP)
8	Number of Scans	16
9	Receiver Gain	101.0
10	Relaxation Delay	1.0000
11	Pulse Width	8.0000
12	Spectrometer Frequency	400.13
13	Spectral Width	8196.7
14	Lowest Frequency	-1627.6
15	Nucleus	1H
16	Acquired Size	32768
17	Spectral Size	65536

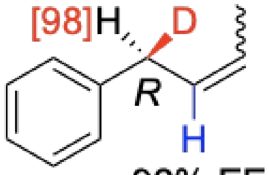

92% EE

(*S*)-**2a** with **L1**

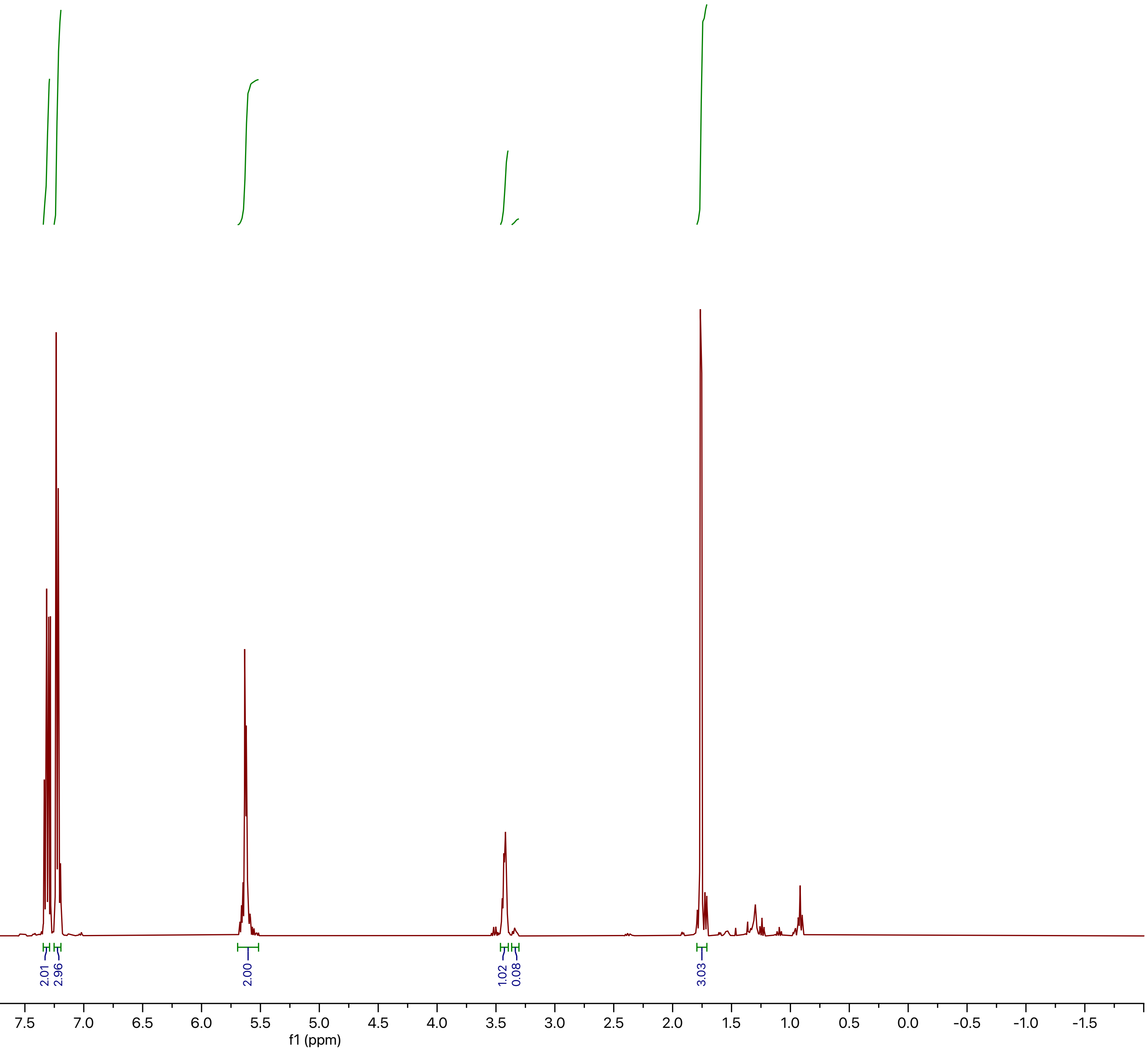

	Parameter	Value
1	Origin	Bruker BioSpin GmbH
2	Instrument	Avance
3	Solvent	CDCl3
4	Temperature	298.0
5	Pulse Sequence	zg30
6	Experiment	1D
7	Probe	Z163739_0400 (PI HR-BBO400S1-BBF/ H/ D-5.0-Z SP)
8	Number of Scans	16
9	Receiver Gain	101.0
10	Relaxation Delay	1.0000
11	Pulse Width	8.0000
12	Spectrometer Frequency	400.13
13	Spectral Width	8196.7
14	Lowest Frequency	-1637.0
15	Nucleus	1H
16	Acquired Size	32768
17	Spectral Size	65536

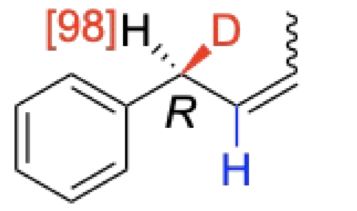

93% EE

(*S*)-**2a** with (*S*)-**L2**

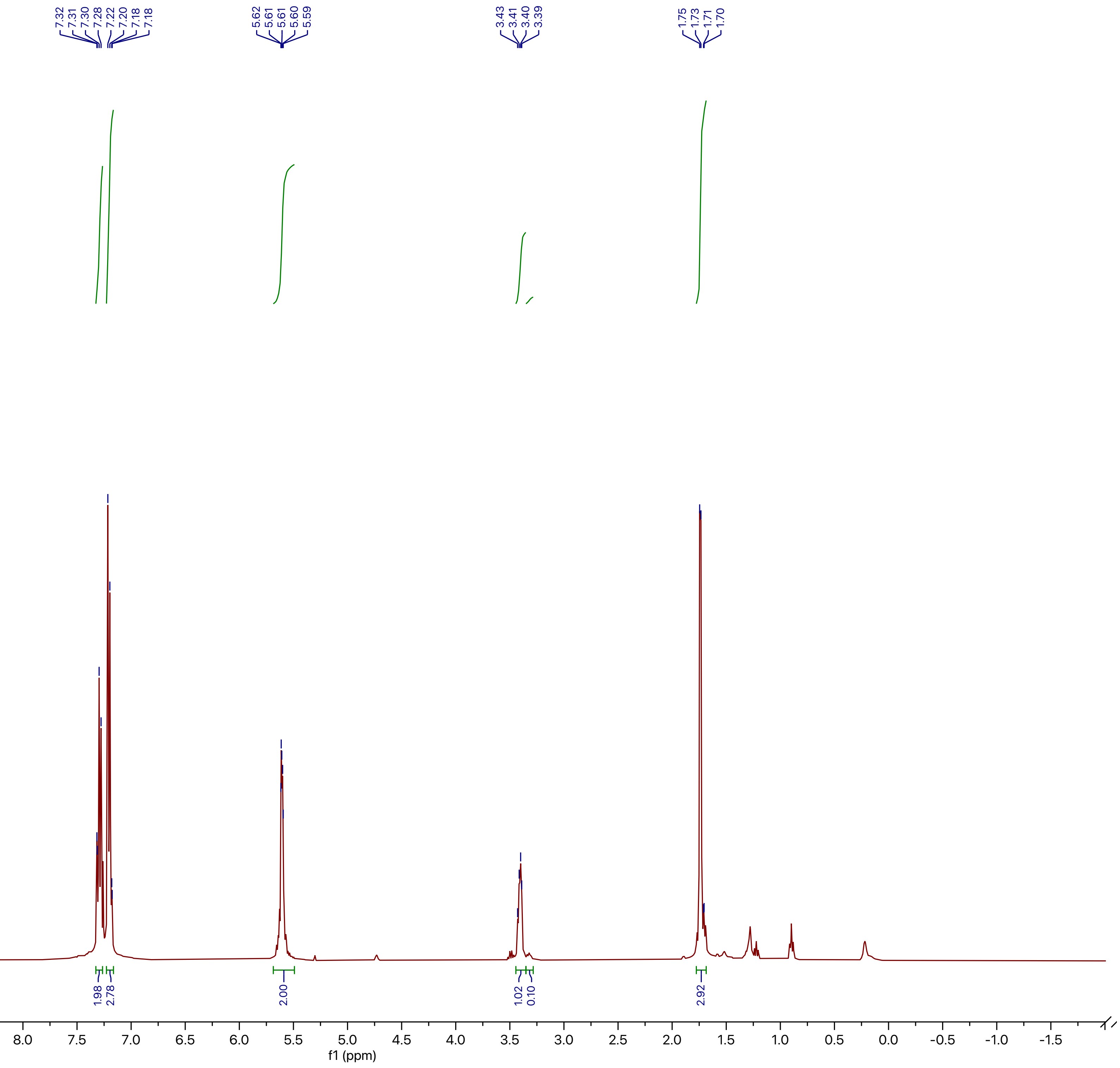

	Parameter	Value
1	Origin	Bruker BioSpin GmbH
2	Instrument	Avance
3	Solvent	CDCl3
4	Temperature	298.0
5	Pulse Sequence	zg30
6	Experiment	1D
7	Probe	Z163739_0400 (PI HR-BBO400S1-BBF/ H/ D-5.0-Z SP)
8	Number of Scans	16
9	Receiver Gain	101.0
10	Relaxation Delay	1.0000
11	Pulse Width	8.0000
12	Spectrometer Frequency	400.13
13	Spectral Width	8196.7
14	Lowest Frequency	-1637.0
15	Nucleus	1H
16	Acquired Size	32768
17	Spectral Size	65536

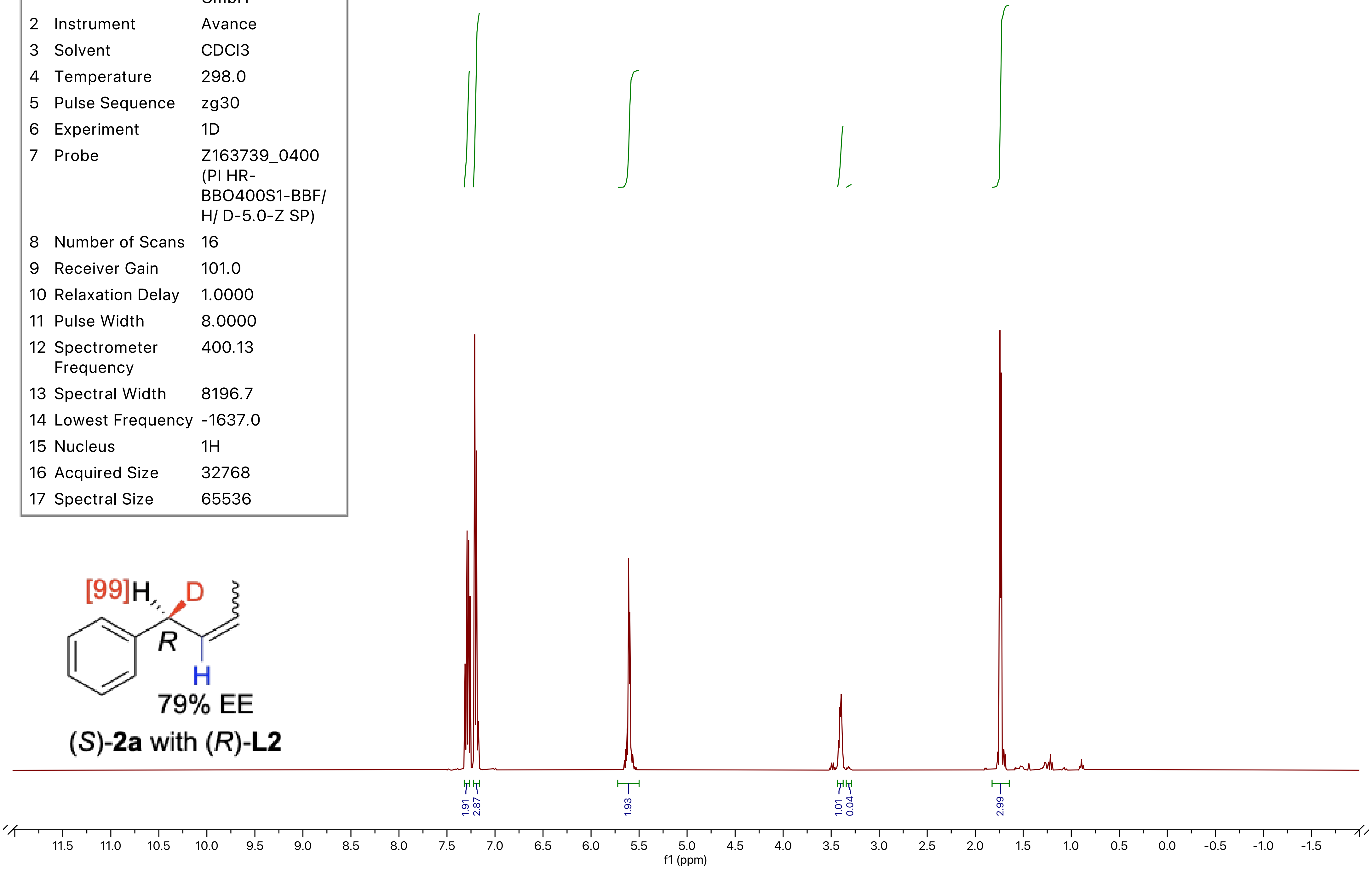

	Parameter	Value
1	Origin	Bruker BioSpin GmbH
2	Instrument	Avance
3	Solvent	CDCl3
4	Temperature	298.0
5	Pulse Sequence	zg30
6	Experiment	1D
7	Probe	Z163739_0400 (PI HR-BBO400S1-BBF/ H/ D-5.0-Z SP)
8	Number of Scans	16
9	Receiver Gain	101.0
10	Relaxation Delay	1.0000
11	Pulse Width	8.0000
12	Spectrometer Frequency	400.13
13	Spectral Width	8196.7
14	Lowest Frequency	-1671.2
15	Nucleus	1H
16	Acquired Size	32768
17	Spectral Size	65536

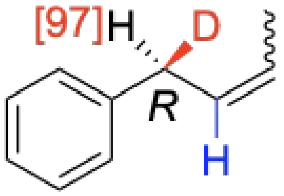

75% EE

(S)-**2a** with **L3**

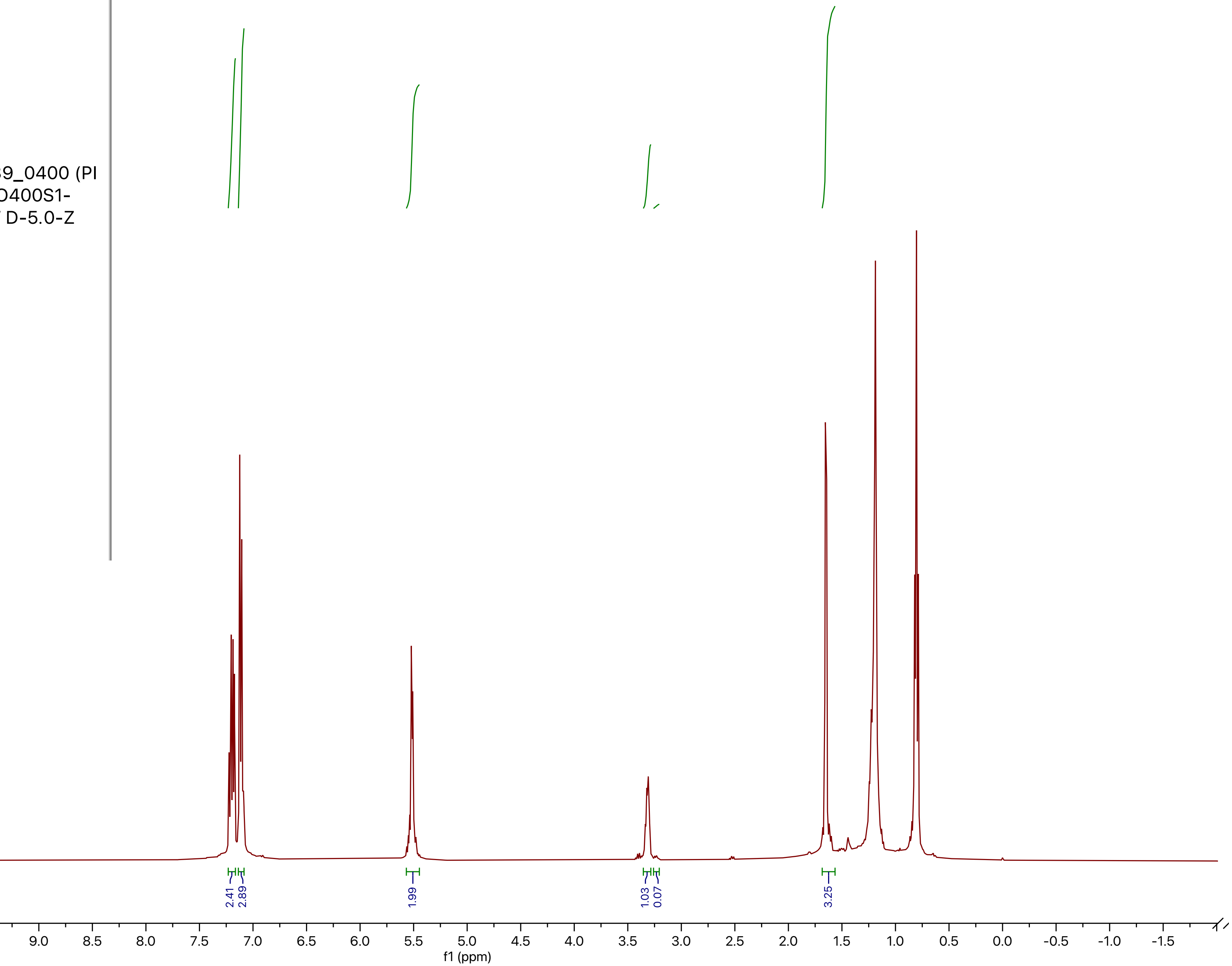

	Parameter	Value
1	Origin	Bruker BioSpin GmbH
2	Instrument	Avance
3	Solvent	CDCl3
4	Temperature	300.0
5	Pulse Sequence	zg30
6	Experiment	1D
7	Probe	Z151574_0073 (PI HR-BBO500S1-BBF/H/ D-5.0-Z SP)
8	Number of Scans	16
9	Receiver Gain	101.0
10	Relaxation Delay	1.0000
11	Pulse Width	8.0000
12	Spectrometer Frequency	500.21
13	Spectral Width	10000.0
14	Lowest Frequency	-1922.9
15	Nucleus	1H
16	Acquired Size	32768
17	Spectral Size	65536

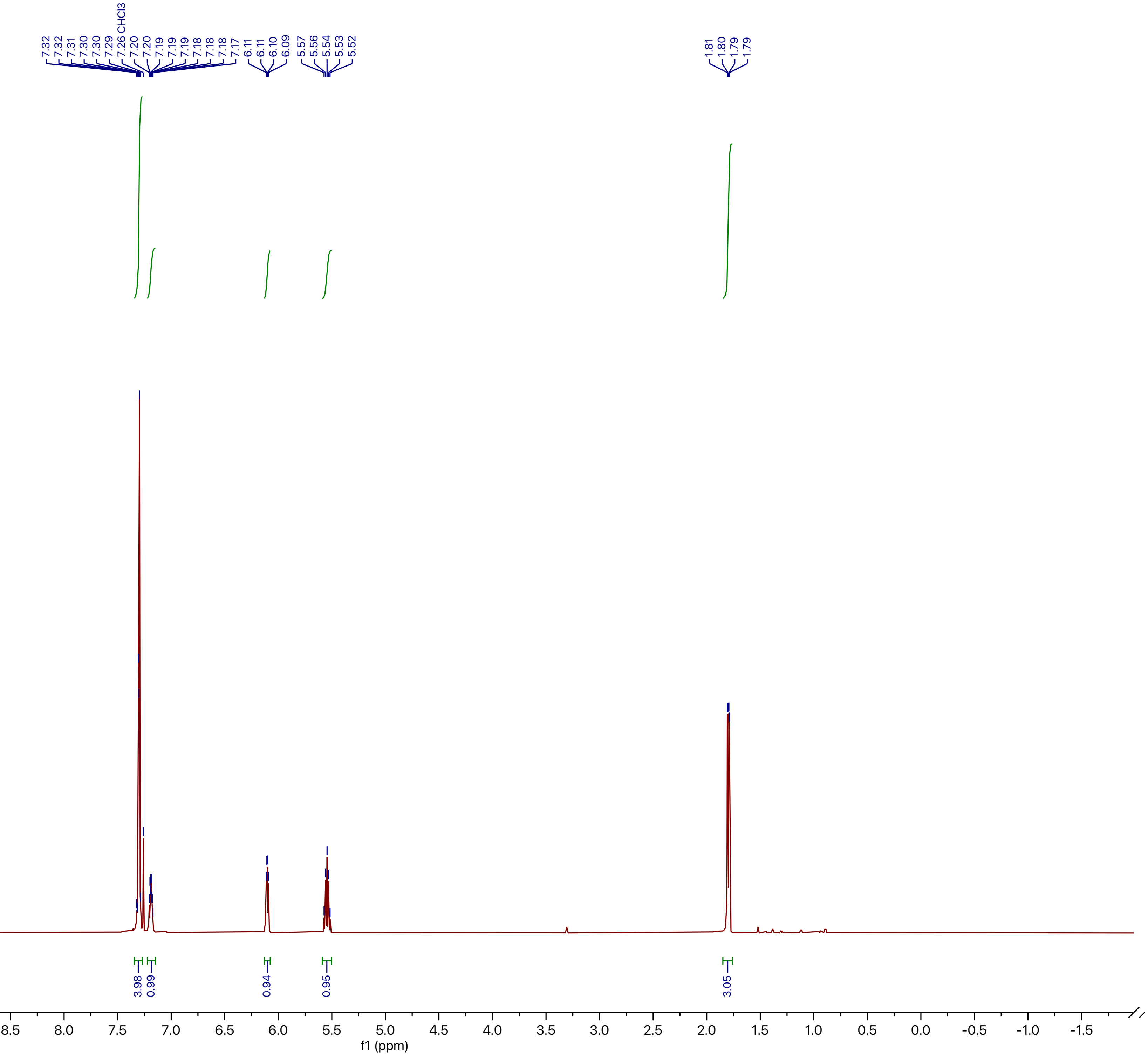

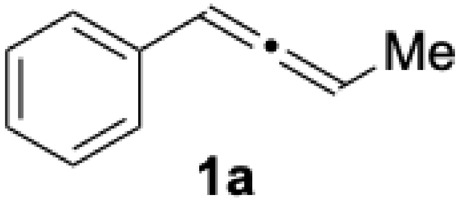

1a

	Parameter	Value
1	Origin	Bruker BioSpin GmbH
2	Instrument	Avance
3	Solvent	CDCl3
4	Temperature	300.0
5	Pulse Sequence	zgpg30
6	Experiment	1D
7	Probe	Z151574_0073 (PI HR-BBO500S1-BBF/H/ D-5.0-Z SP)
8	Number of Scans	1024
9	Receiver Gain	101.0
10	Relaxation Delay	2.0000
11	Pulse Width	9.0000
12	Spectrometer Frequency	125.79
13	Spectral Width	30120.5
14	Lowest Frequency	-2482.4
15	Nucleus	13C
16	Acquired Size	32768
17	Spectral Size	65536

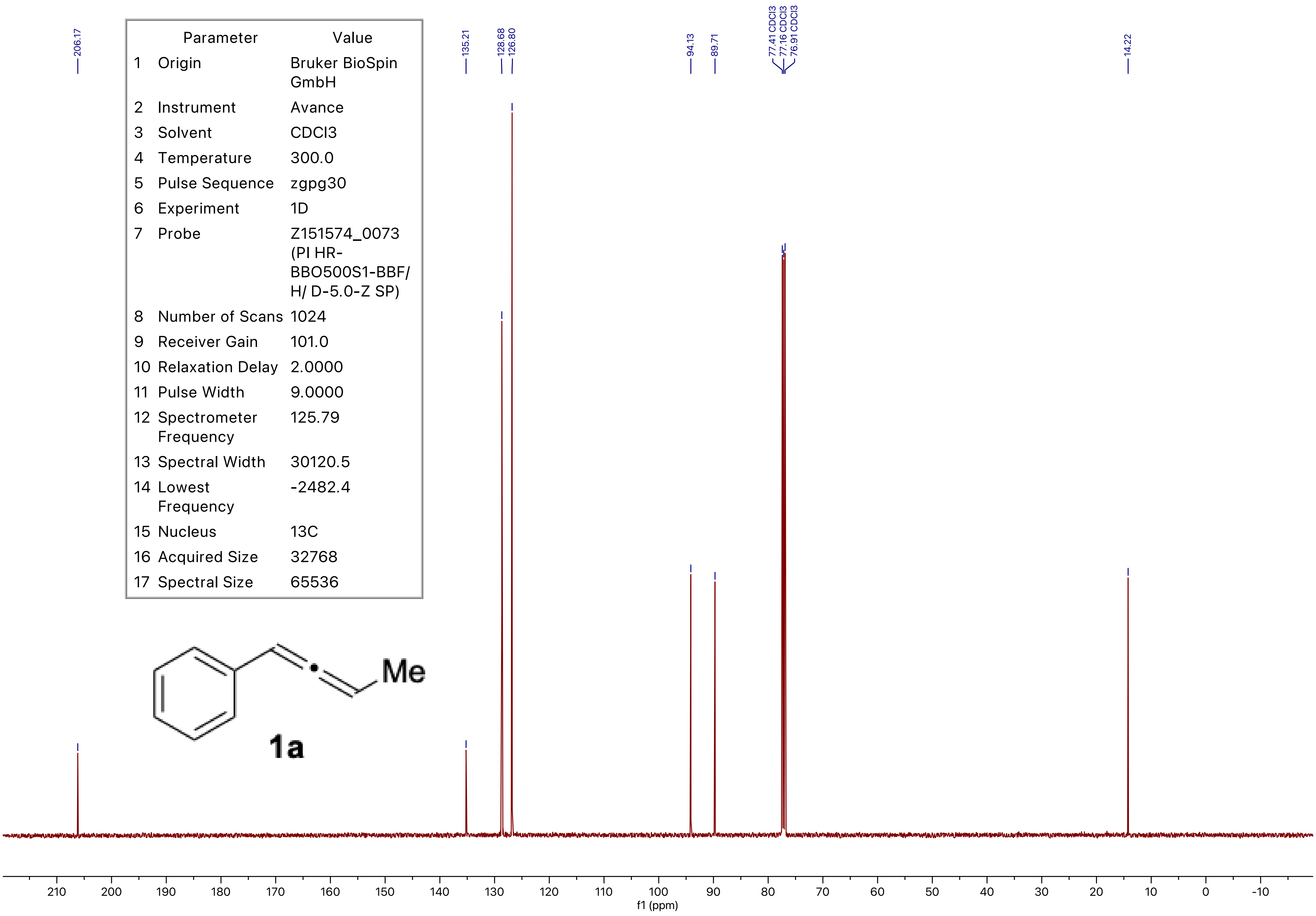

1a

	Parameter	Value
1	Origin	Bruker BioSpin GmbH
2	Instrument	Avance
3	Solvent	CDCl3
4	Temperature	298.0
5	Pulse Sequence	zg30
6	Experiment	1D
7	Probe	Z163739_0400 (PI HR-BBO400S1-BBF/ H/ D-5.0-Z SP)
8	Number of Scans	16
9	Receiver Gain	101.0
10	Relaxation Delay	1.0000
11	Pulse Width	8.0000
12	Spectrometer Frequency	400.13
13	Spectral Width	8196.7
14	Lowest Frequency	-1636.9
15	Nucleus	1H
16	Acquired Size	32768
17	Spectral Size	65536

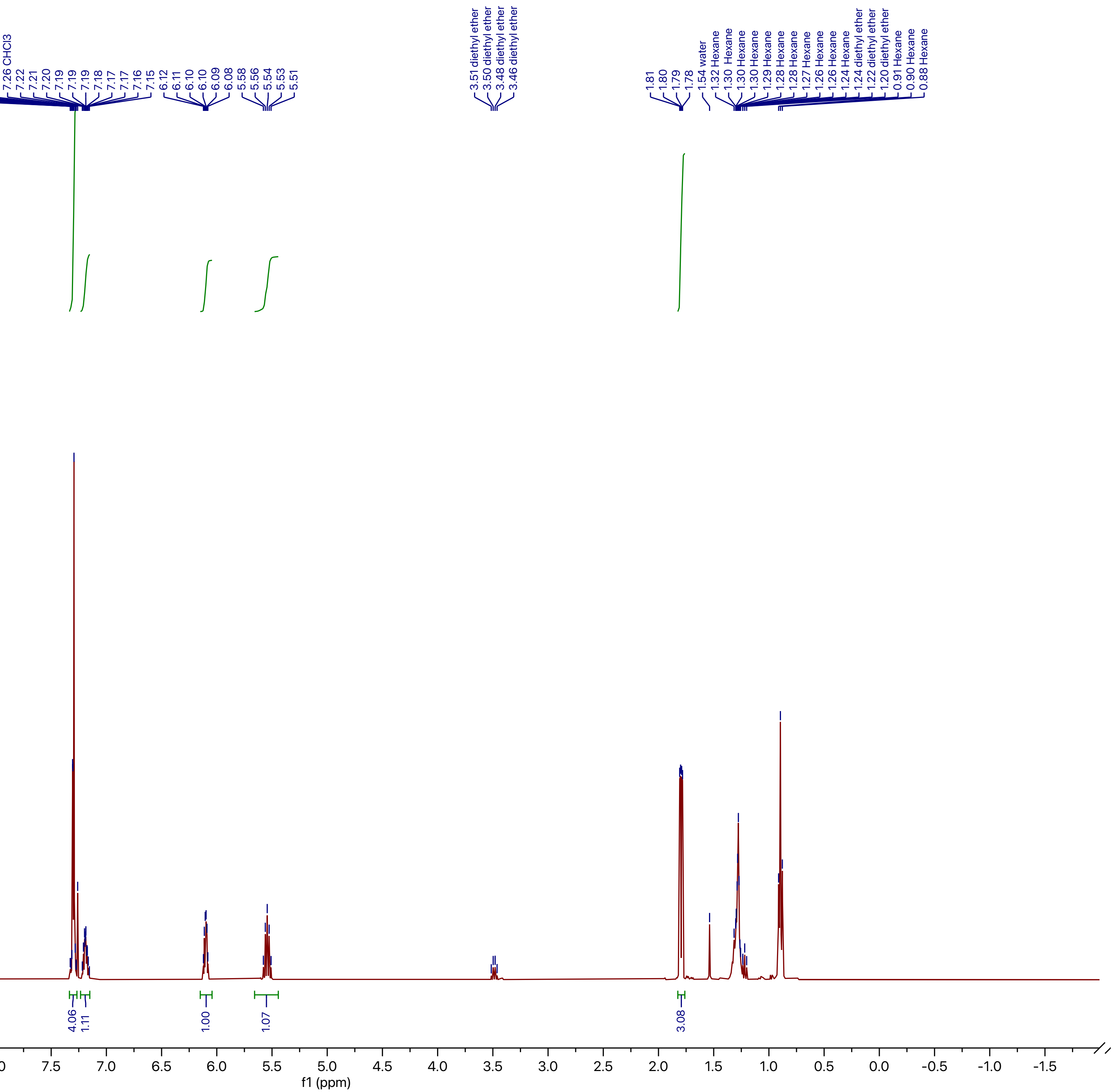

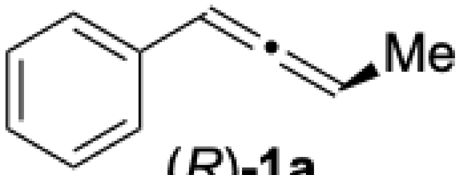

(*R*)-**1a**

	Parameter	Value
1	Origin	Bruker BioSpin GmbH
2	Instrument	Avance
3	Solvent	CDCl3
4	Temperature	298.0
5	Pulse Sequence	zg30
6	Experiment	1D
7	Probe	Z163739_0400 (PI HR-BBO400S1-BBF/ H/ D-5.0-Z SP)
8	Number of Scans	16
9	Receiver Gain	101.0
10	Relaxation Delay	1.0000
11	Pulse Width	8.0000
12	Spectrometer Frequency	400.13
13	Spectral Width	8196.7
14	Lowest Frequency	-1636.9
15	Nucleus	1H
16	Acquired Size	32768
17	Spectral Size	65536

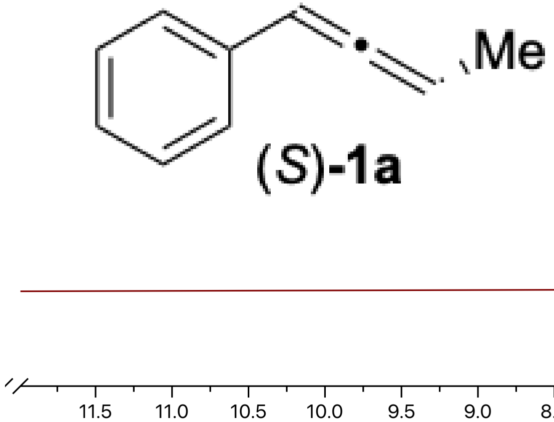

(S)-1a

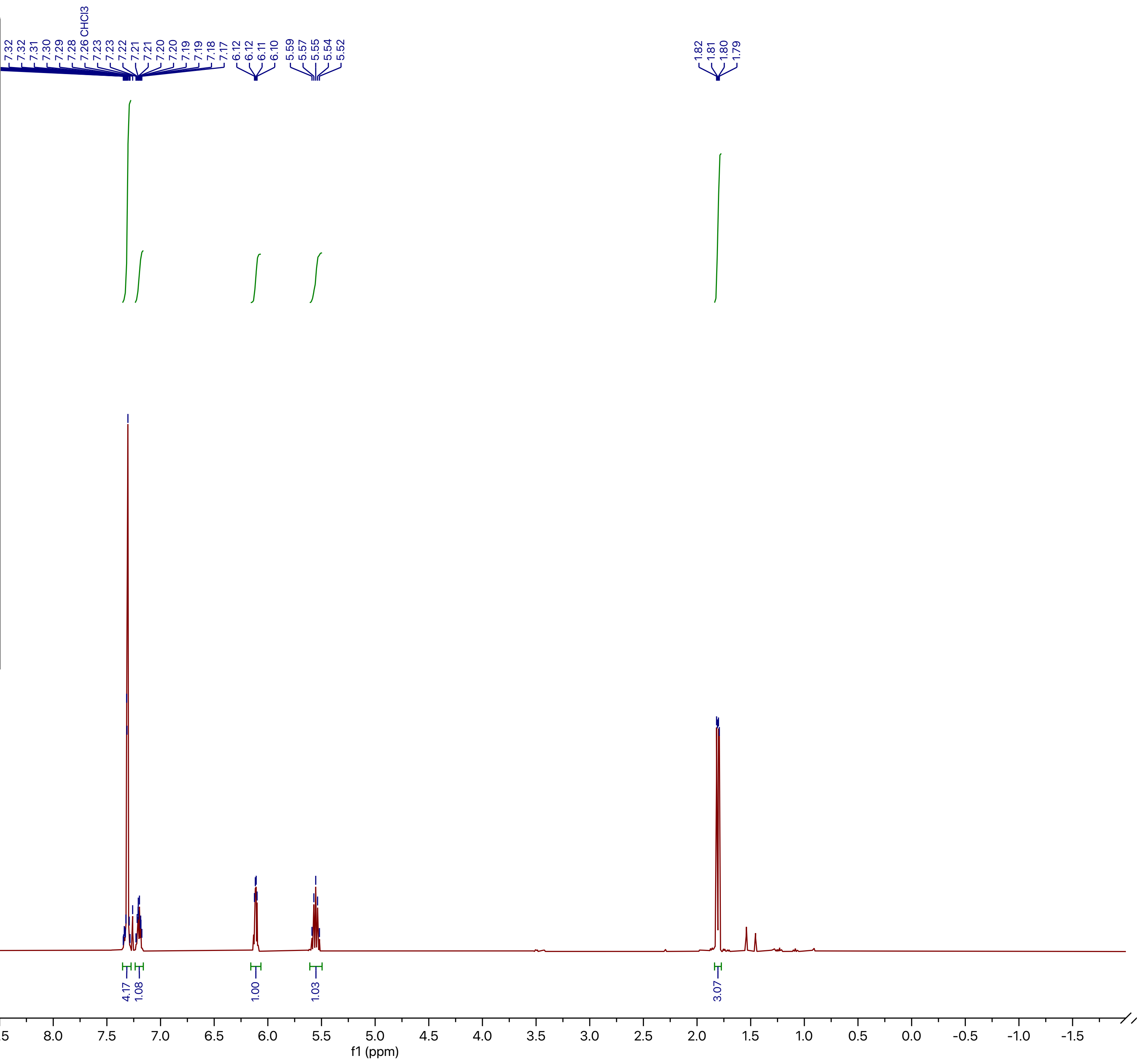

	Parameter	Value
1	Origin	Bruker BioSpin GmbH
2	Instrument	Avance
3	Solvent	CDCl3
4	Temperature	298.0
5	Pulse Sequence	zg30
6	Experiment	1D
7	Probe	Z163739_0400 (PI HR-BBO400S1-BBF/ H/ D-5.0-Z SP)
8	Number of Scans	16
9	Receiver Gain	101.0
10	Relaxation Delay	1.0000
11	Pulse Width	8.0000
12	Spectrometer Frequency	400.13
13	Spectral Width	8196.7
14	Lowest Frequency	-1637.0
15	Nucleus	1H
16	Acquired Size	32768
17	Spectral Size	65536

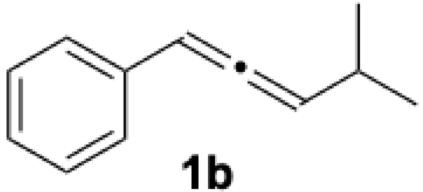

1b

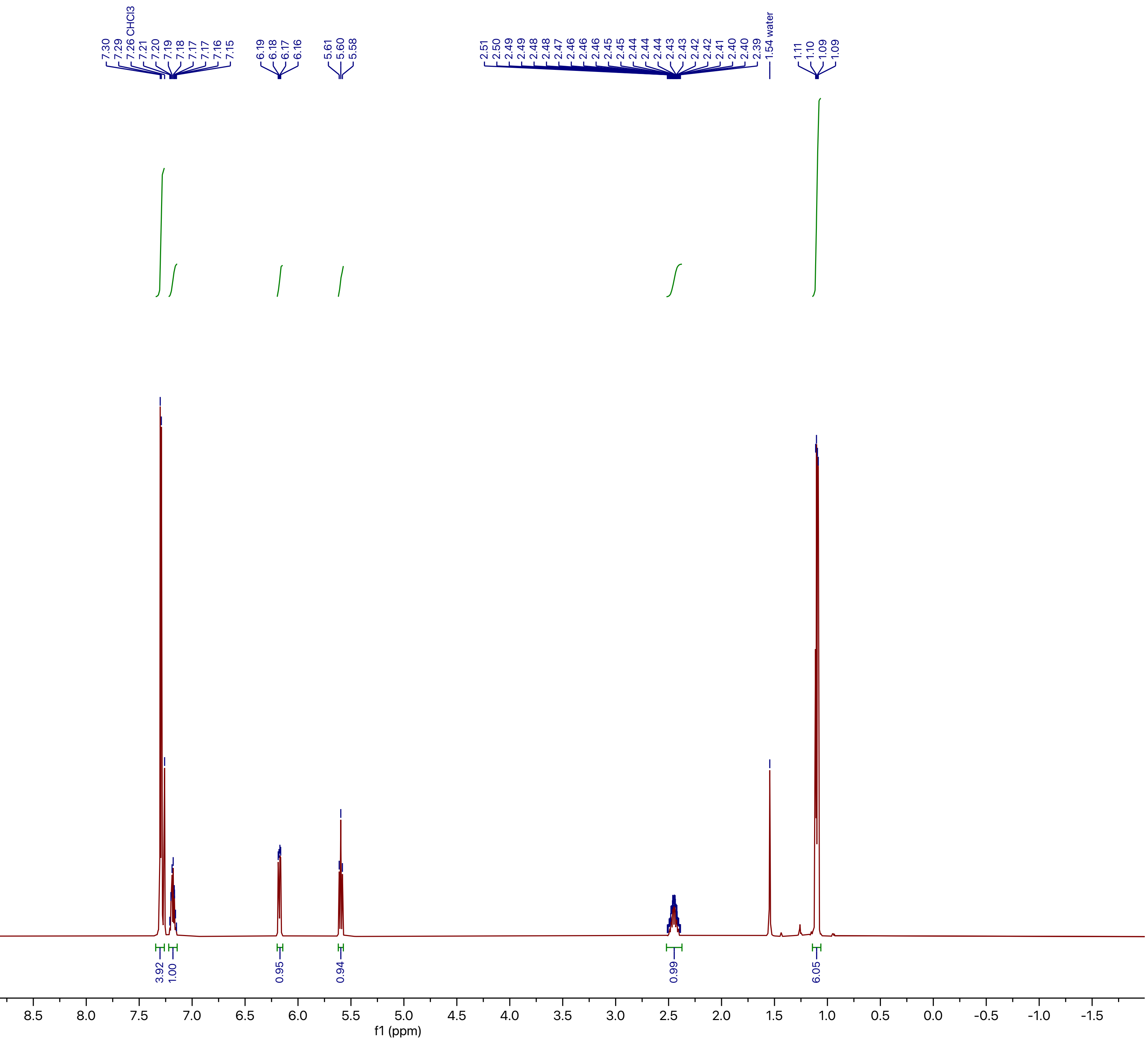

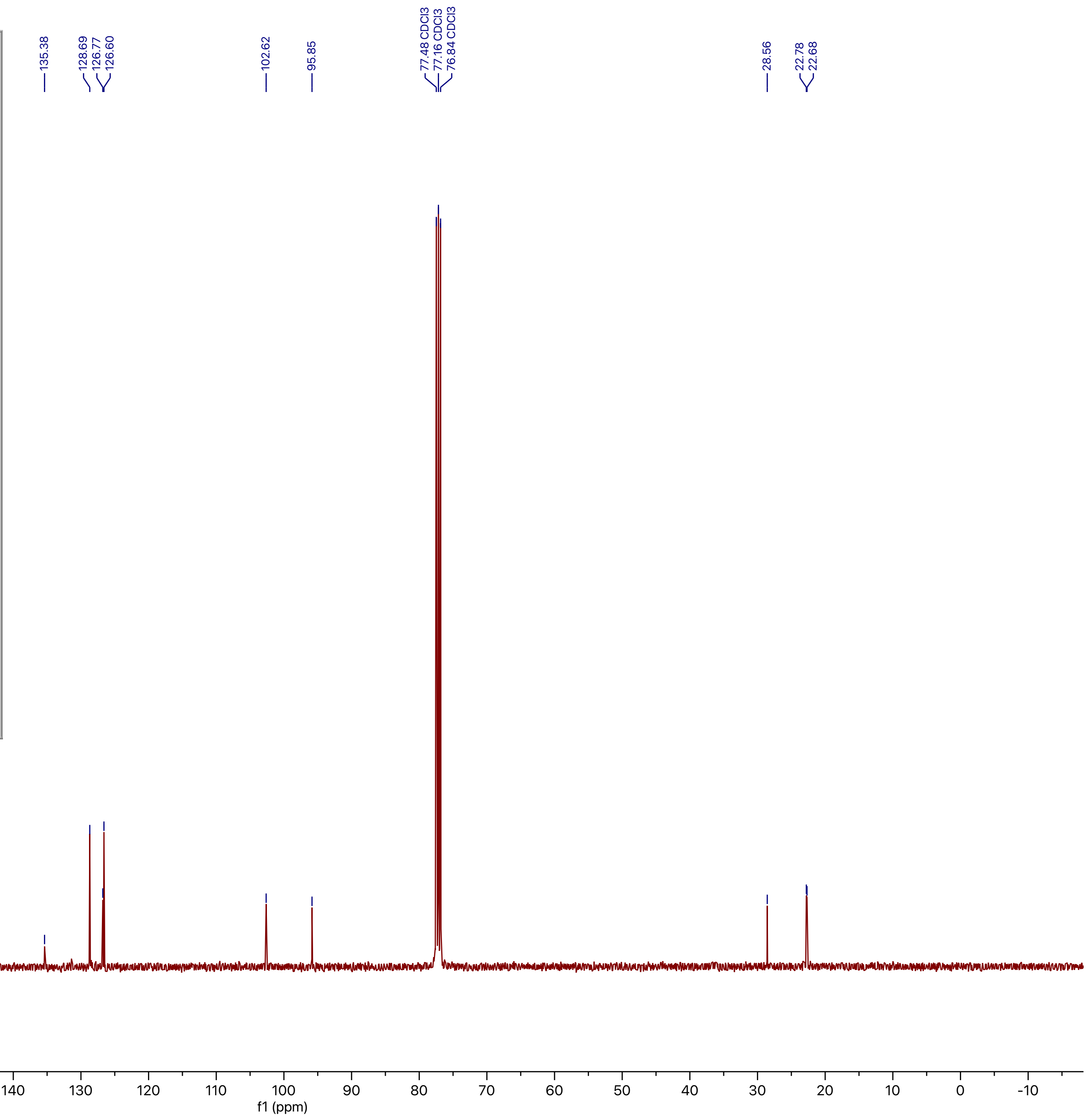

	Parameter	Value
1	Origin	Bruker BioSpin GmbH
2	Instrument	Avance
3	Solvent	CDCl3
4	Temperature	298.0
5	Pulse Sequence	zgpg30
6	Experiment	1D
7	Probe	Z163739_0400 (PI HR-BBO400S1-BBF/H/ D-5.0-Z SP)
8	Number of Scans	1024
9	Receiver Gain	101.0
10	Relaxation Delay	2.0000
11	Pulse Width	8.0000
12	Spectrometer Frequency	100.62
13	Spectral Width	23809.5
14	Lowest Frequency	-1843.5
15	Nucleus	13C
16	Acquired Size	32768
17	Spectral Size	65536

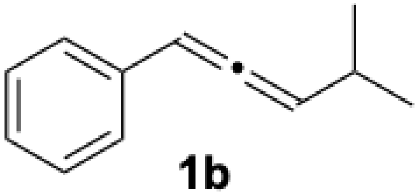

1b

	Parameter	Value
1	Origin	Varian
2	Solvent	cdcl3
3	Temperature	-30.0
4	Pulse Sequence	s2pul
5	Experiment	1D
6	Probe	ASWPFG8319
7	Number of Scans	16
8	Receiver Gain	42
9	Relaxation Delay	2.0000
10	Pulse Width	7.7500
11	Spectrometer Frequency	399.73
12	Spectral Width	6410.3
13	Lowest Frequency	-806.4
14	Nucleus	1H
15	Acquired Size	16384
16	Spectral Size	65536
17	Digital Resolution	0.10

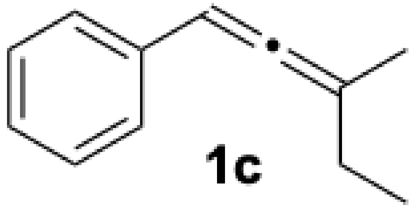

1c

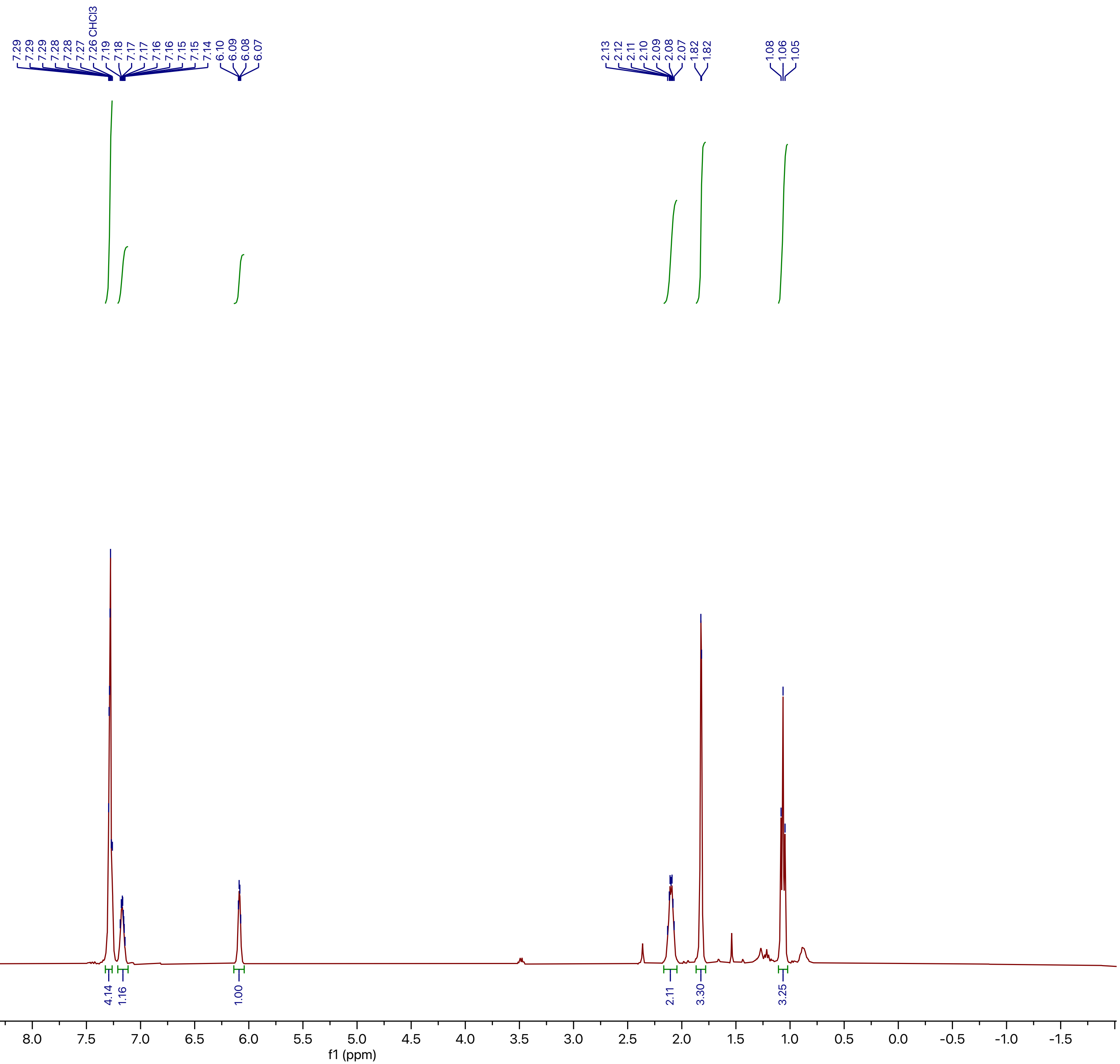

	Parameter	Value
1	Origin	Varian
2	Solvent	cdcl3
3	Temperature	-30.0
4	Pulse Sequence	s2pul
5	Experiment	1D
6	Probe	ASWPFG8319
7	Number of Scans	256
8	Receiver Gain	30
9	Relaxation Delay	1.0000
10	Pulse Width	5.7500
11	Spectrometer Frequency	100.52
12	Spectral Width	25000.0
13	Lowest Frequency	-1443.7
14	Nucleus	13C
15	Acquired Size	32768
16	Spectral Size	65536
17	Digital Resolution	0.38

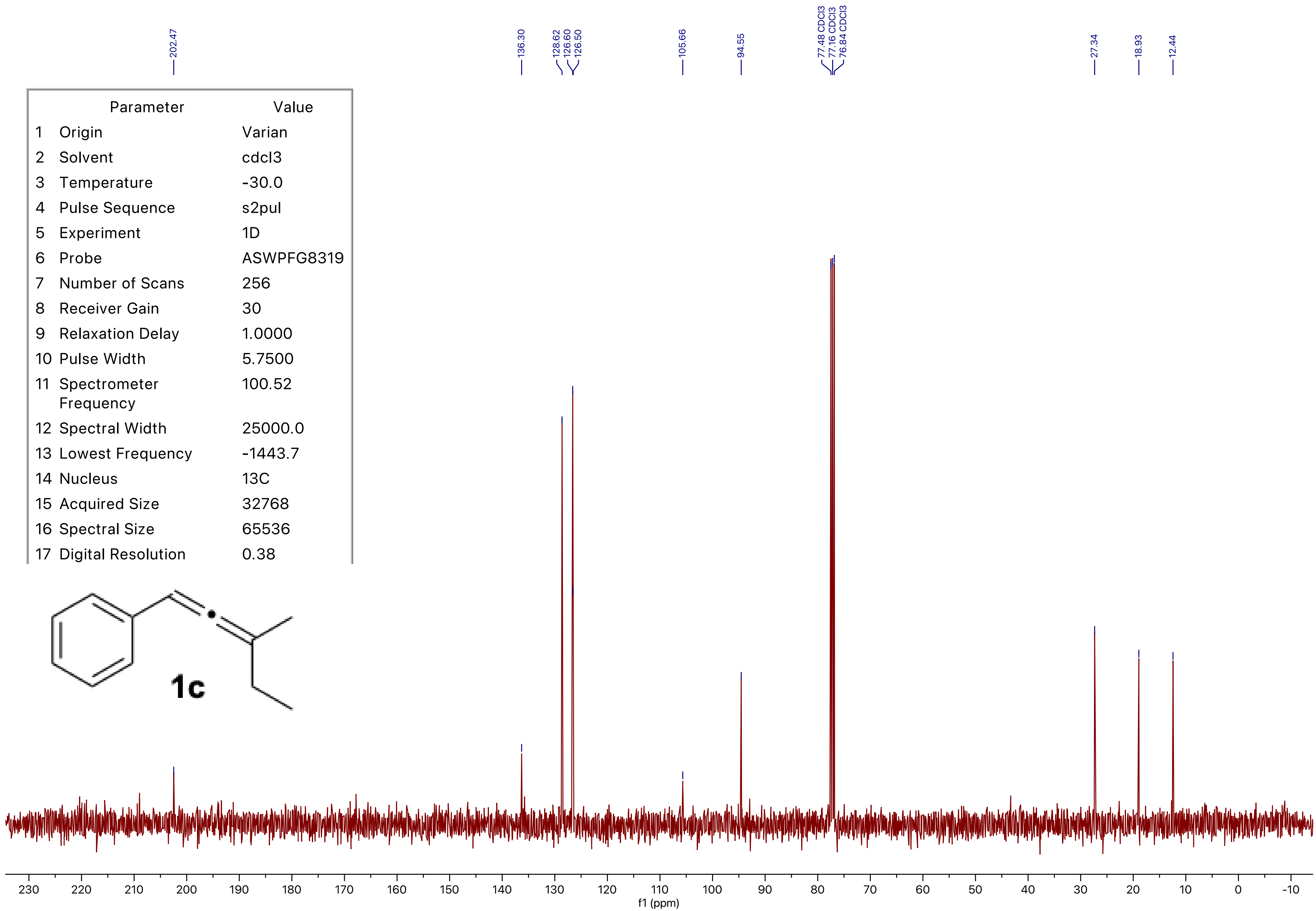

1c

	Parameter	Value
1	Origin	Varian
2	Solvent	cdcl3
3	Temperature	-30.0
4	Pulse Sequence	s2pul
5	Experiment	1D
6	Probe	ASWPFG8319
7	Number of Scans	16
8	Receiver Gain	52
9	Relaxation Delay	2.0000
10	Pulse Width	7.7500
11	Spectrometer Frequency	399.73
12	Spectral Width	6410.3
13	Lowest Frequency	-805.9
14	Nucleus	1H
15	Acquired Size	16384
16	Spectral Size	65536
17	Digital Resolution	0.10

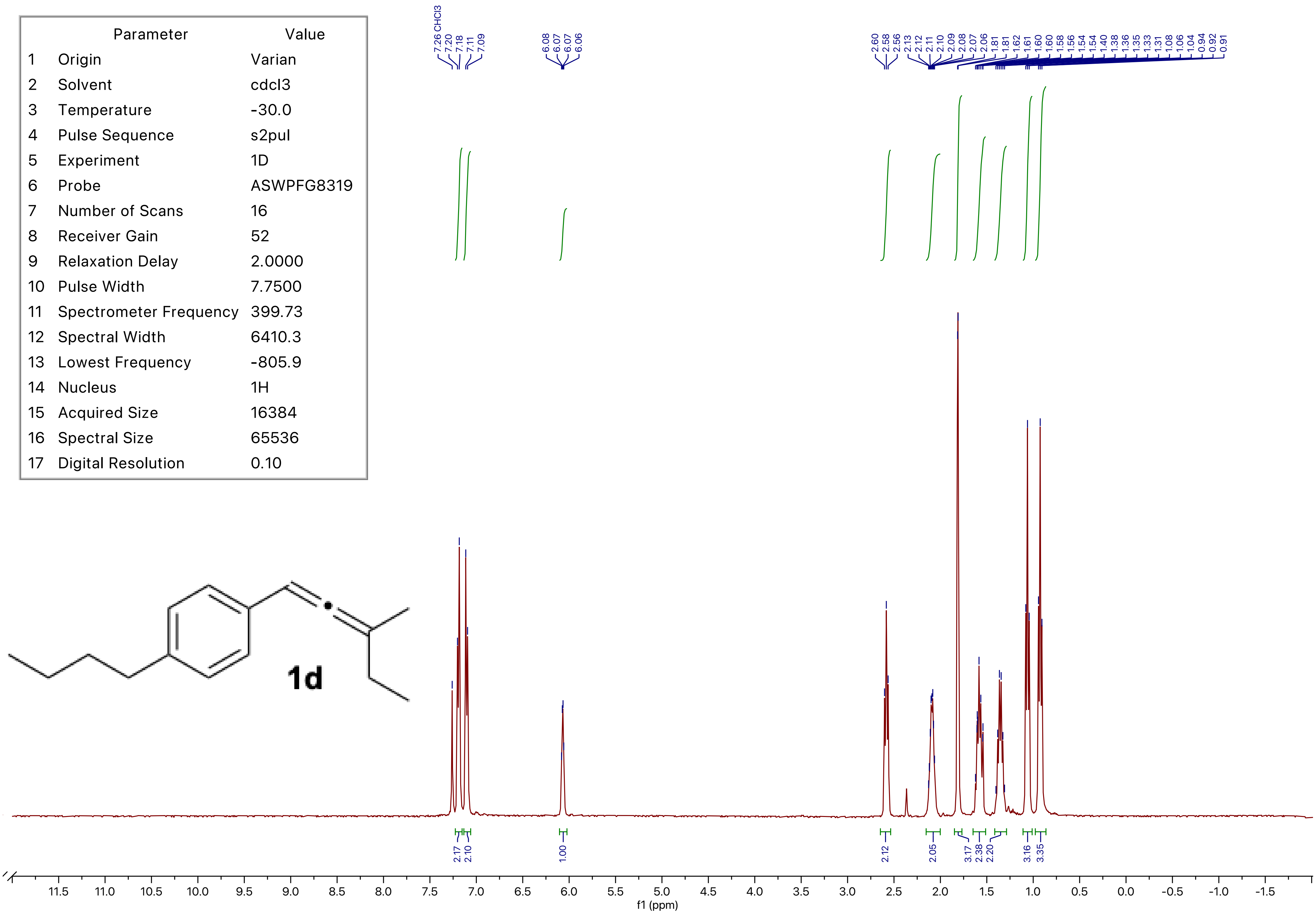

	Parameter	Value
1	Origin	Varian
2	Solvent	cdcl3
3	Temperature	-30.0
4	Pulse Sequence	s2pul
5	Experiment	1D
6	Probe	ASWPFG8319
7	Number of Scans	256
8	Receiver Gain	30
9	Relaxation Delay	1.0000
10	Pulse Width	5.7500
11	Spectrometer Frequency	100.52
12	Spectral Width	25000.0
13	Lowest Frequency	-1443.7
14	Nucleus	13C
15	Acquired Size	32768
16	Spectral Size	65536
17	Digital Resolution	0.38

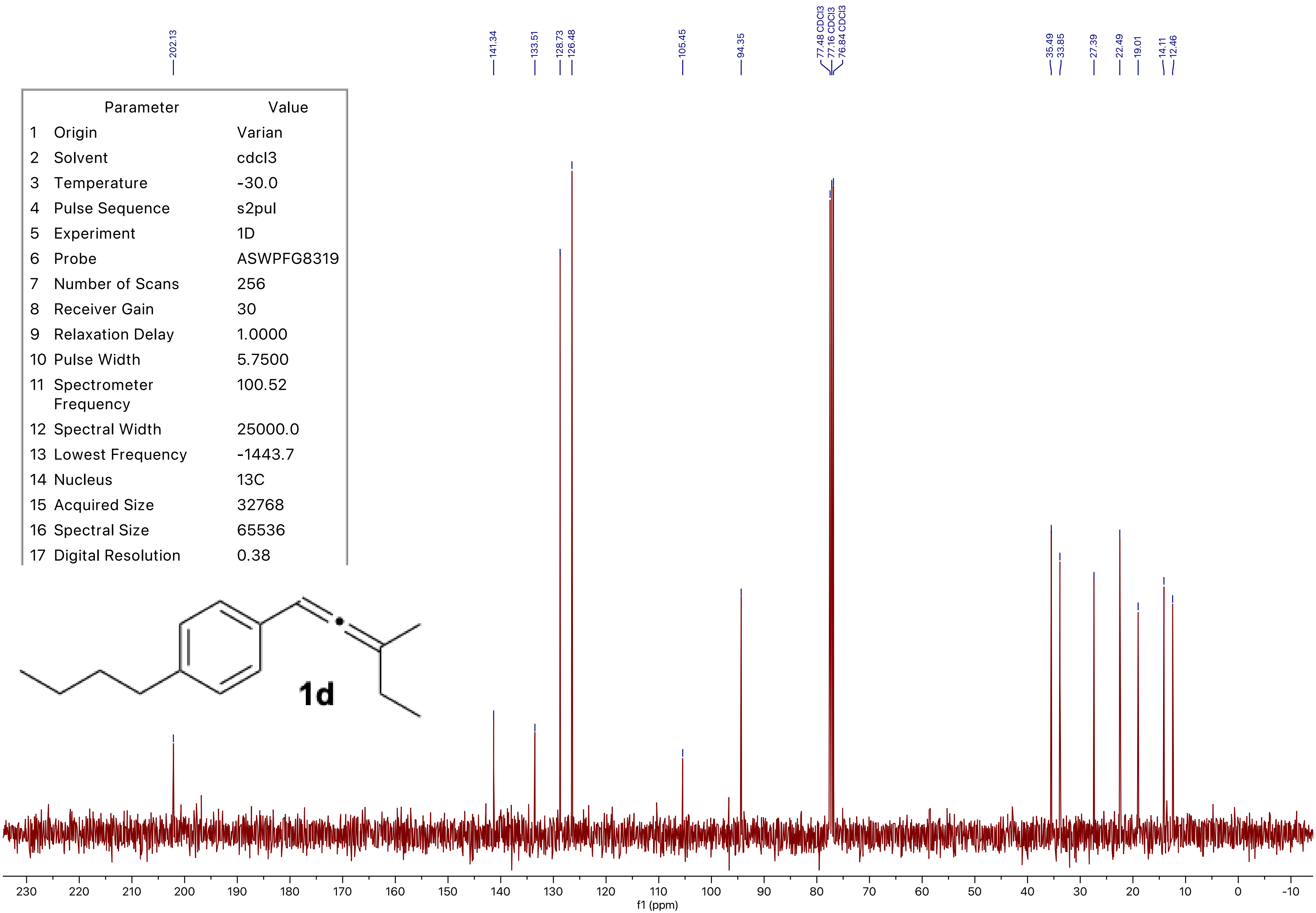

1d

	Parameter	Value
1	Origin	Varian
2	Solvent	cdcl3
3	Temperature	-30.0
4	Pulse Sequence	s2pul
5	Experiment	1D
6	Probe	ASWPFG8319
7	Number of Scans	16
8	Receiver Gain	60
9	Relaxation Delay	5.0000
10	Pulse Width	7.7500
11	Spectrometer Frequency	399.73
12	Spectral Width	6410.3
13	Lowest Frequency	-806.3
14	Nucleus	1H
15	Acquired Size	16384
16	Spectral Size	65536
17	Digital Resolution	0.10

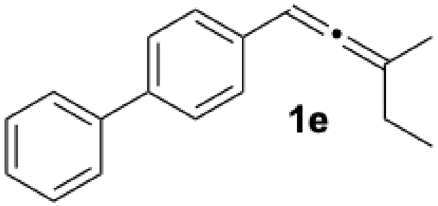

1e

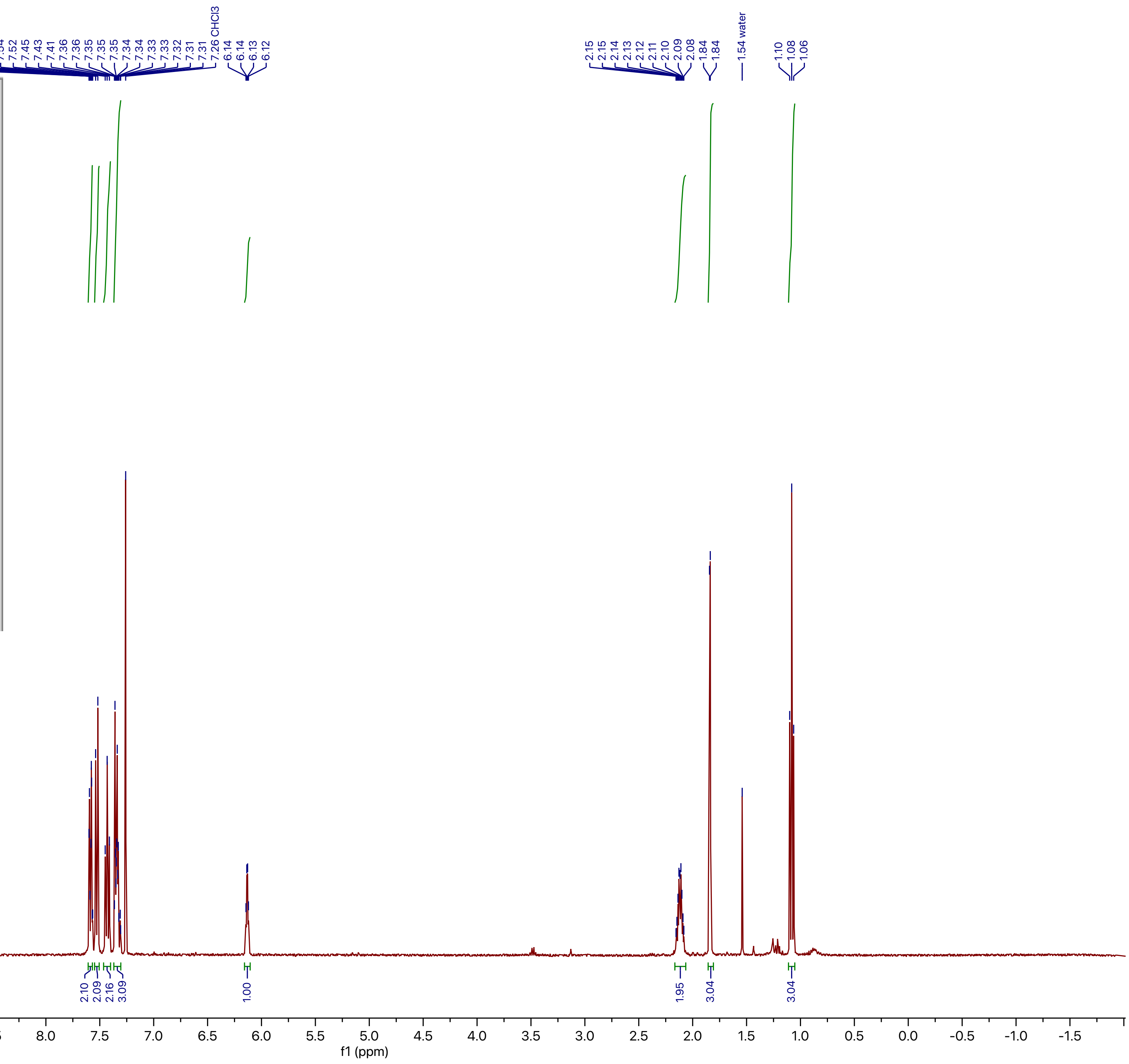

	Parameter	Value
1	Origin	Bruker BioSpin GmbH
2	Instrument	Avance
3	Solvent	CDCl3
4	Temperature	300.0
5	Pulse Sequence	zgpg30
6	Experiment	1D
7	Probe	Z151574_0073 (PI HR-BBO500S1-BBF/H/ D-5.0-Z SP)
8	Number of Scans	104
9	Receiver Gain	101.0
10	Relaxation Delay	2.0000
11	Pulse Width	9.0000
12	Spectrometer Frequency	125.79
13	Spectral Width	30120.5
14	Lowest Frequency	-2466.2
15	Nucleus	13C
16	Acquired Size	32768
17	Spectral Size	65536

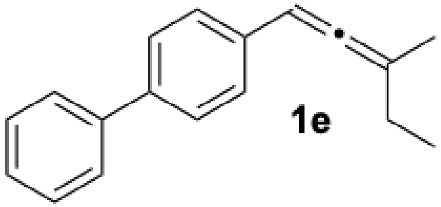

1e

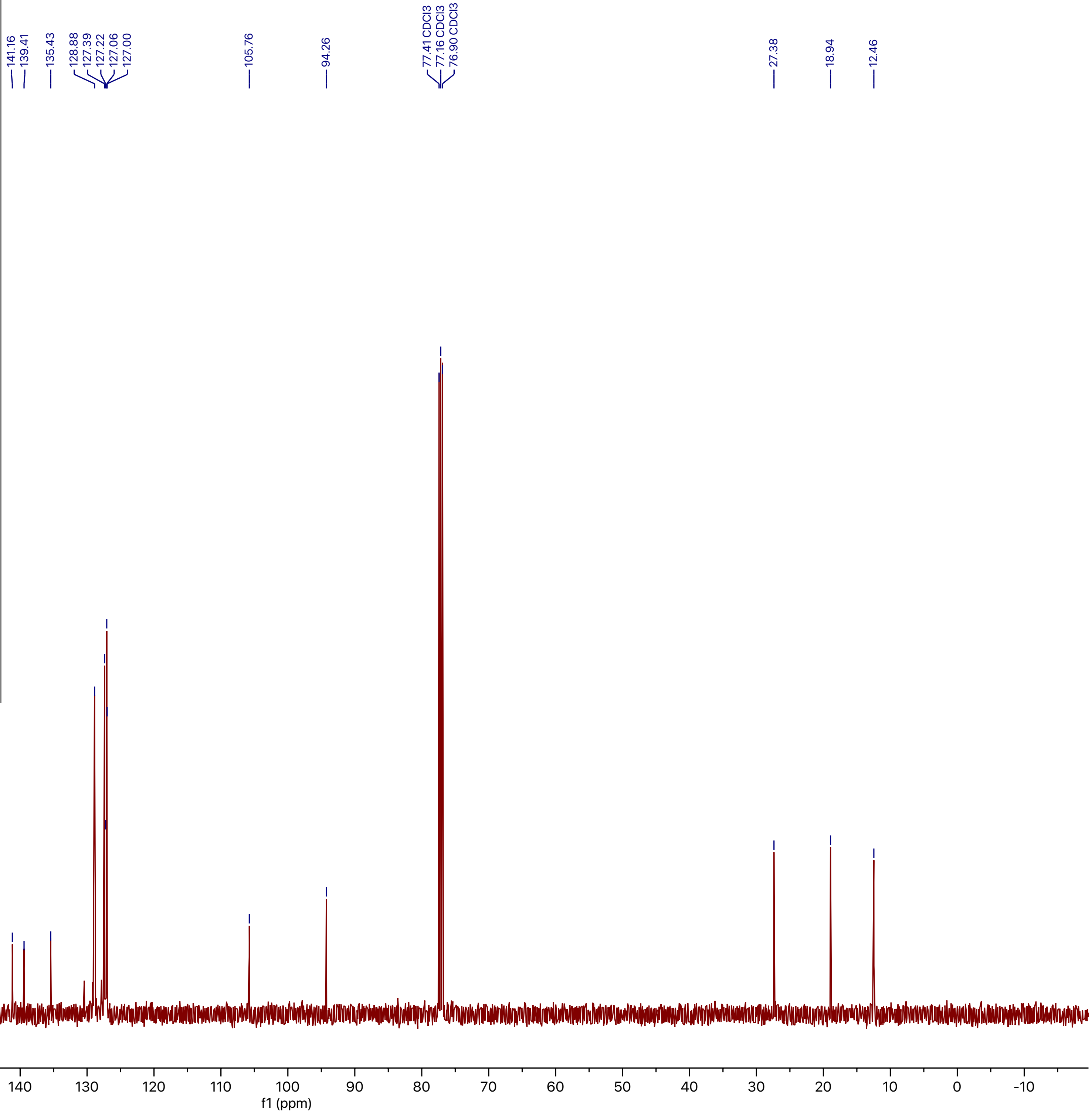

	Parameter	Value
1	Origin	Varian
2	Solvent	cdcl3
3	Temperature	0.0
4	Pulse Sequence	s2pul
5	Experiment	1D
6	Probe	ASWPFG8319
7	Number of Scans	16
8	Receiver Gain	52
9	Relaxation Delay	5.0000
10	Pulse Width	7.7500
11	Spectrometer Frequency	399.73
12	Spectral Width	6410.3
13	Lowest Frequency	-805.7
14	Nucleus	1H
15	Acquired Size	16384
16	Spectral Size	65536
17	Digital Resolution	0.10

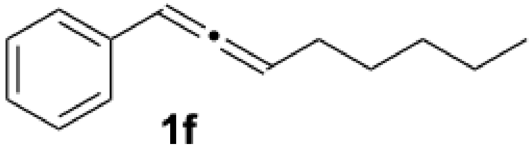

1f

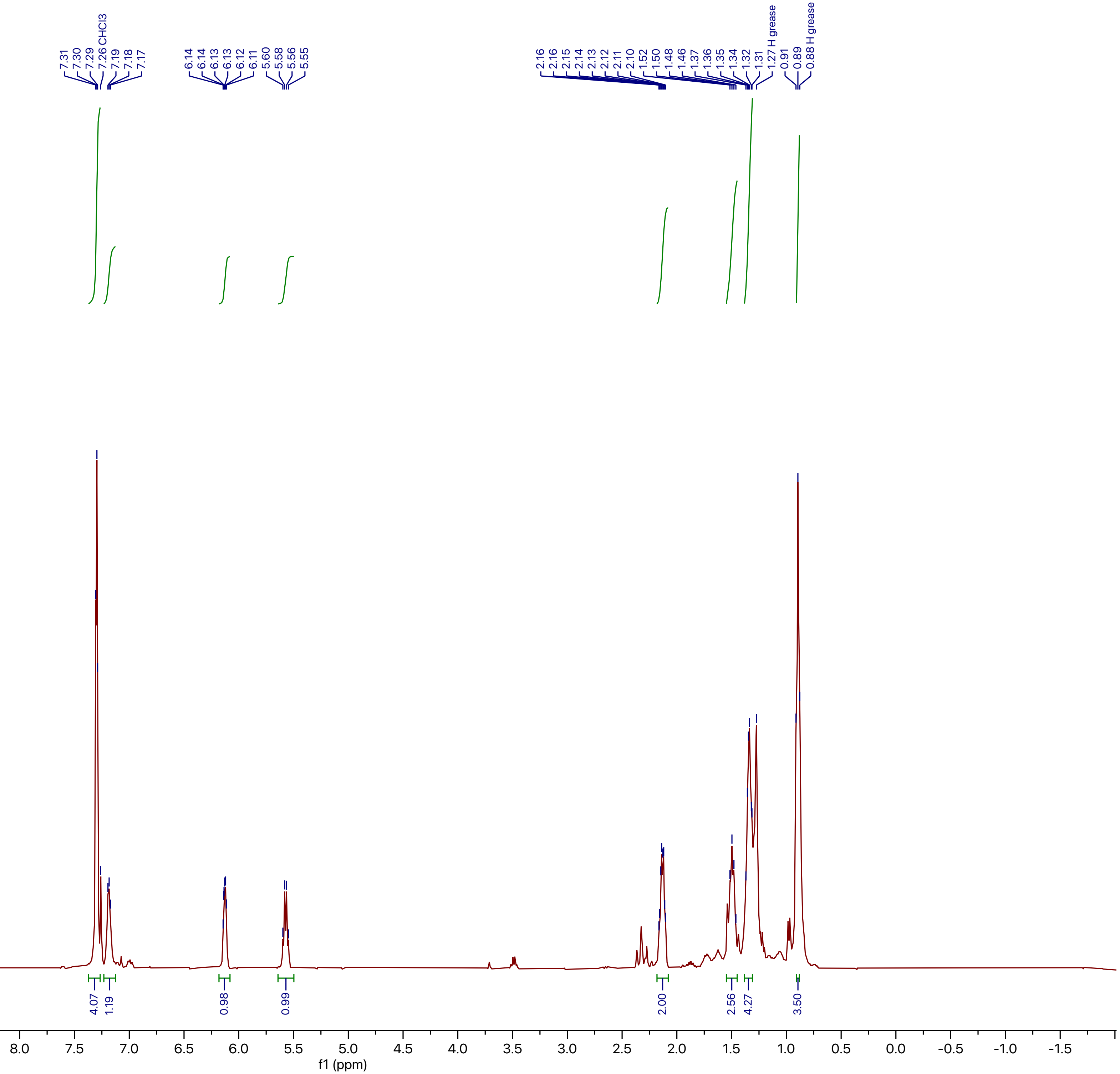

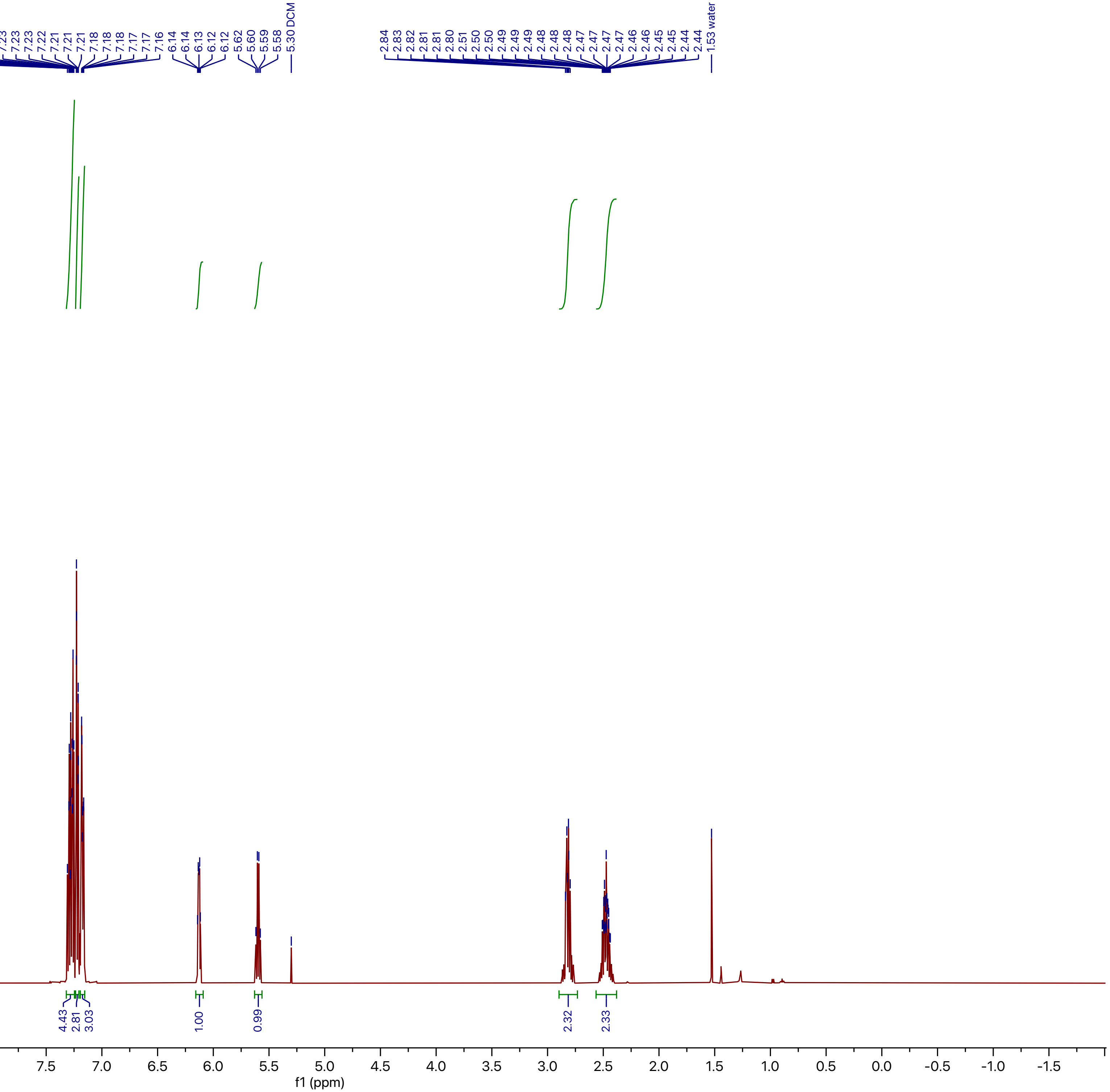

	Parameter	Value
1	Origin	Varian
2	Solvent	cdcl3
3	Temperature	25.0
4	Pulse Sequence	s2pul
5	Experiment	1D
6	Probe	OneNMR_W036
7	Number of Scans	8
8	Receiver Gain	36
9	Relaxation Delay	1.0000
10	Pulse Width	5.1500
11	Spectrometer Frequency	499.73
12	Spectral Width	8012.8
13	Lowest Frequency	-1008.0
14	Nucleus	1H
15	Acquired Size	16384
16	Spectral Size	65536
17	Digital Resolution	0.12

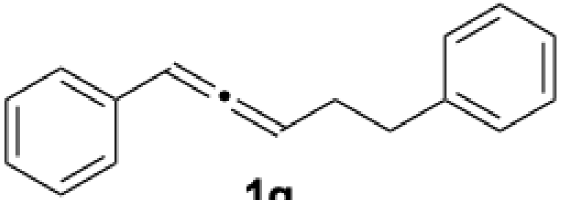

1g

Parameter	Value
1 Origin	Varian
2 Solvent	cdcl3
3 Temperature	25.0
4 Pulse Sequence	s2pul
5 Experiment	1D
6 Probe	ASWPFG8319
7 Number of Scans	16
8 Receiver Gain	46
9 Relaxation Delay	5.0000
10 Pulse Width	7.7500
11 Spectrometer Frequency	399.73
12 Spectral Width	6410.3
13 Lowest Frequency	-806.7
14 Nucleus	1H
15 Acquired Size	16384
16 Spectral Size	65536
17 Digital Resolution	0.10

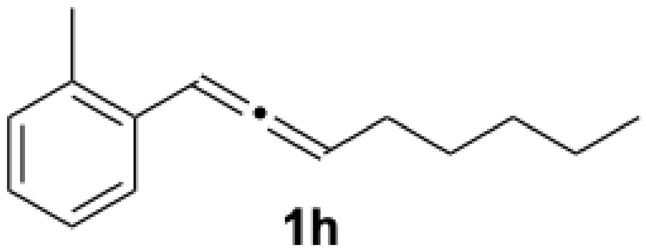

1h

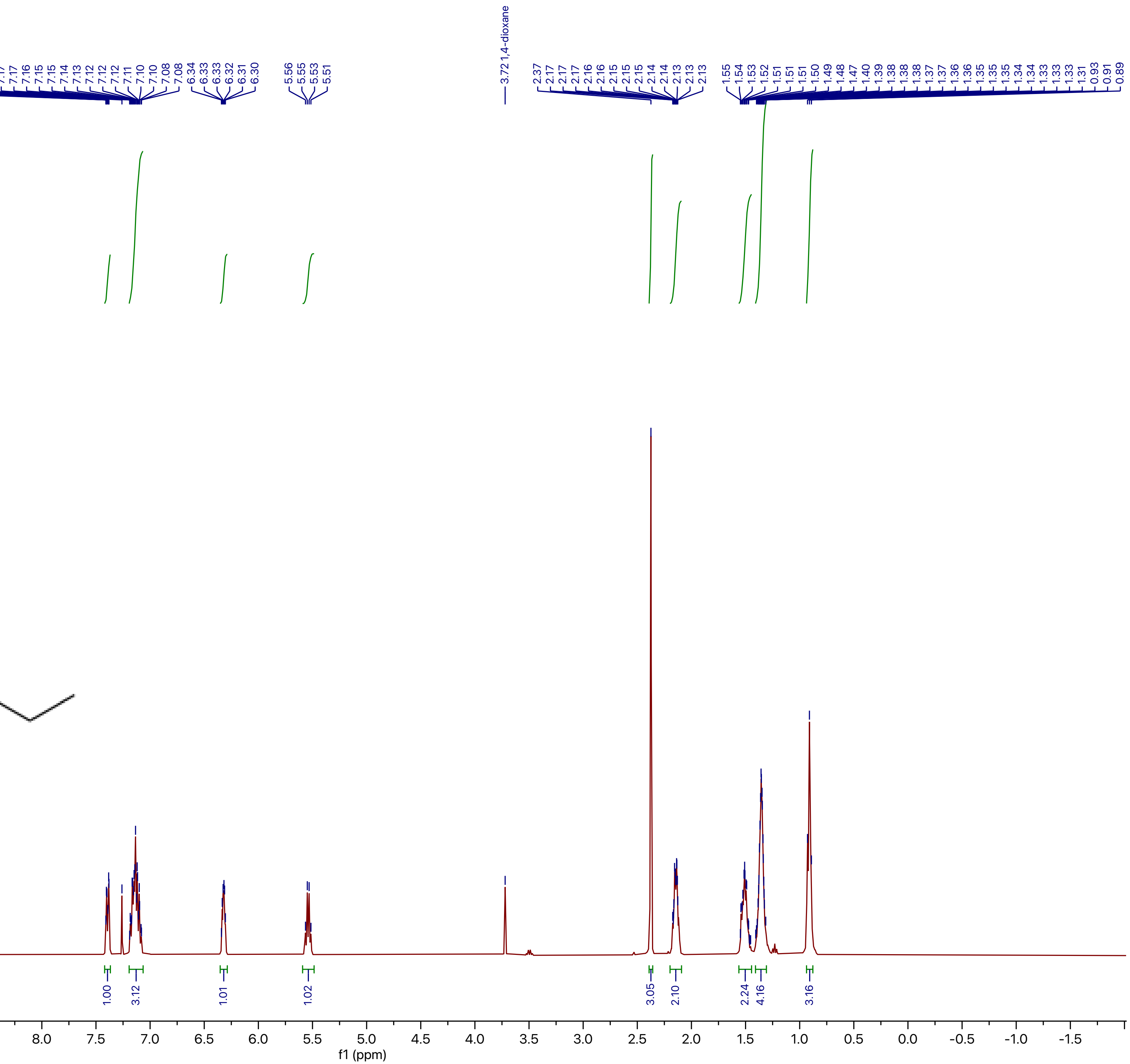

	Parameter	Value
1	Origin	Varian
2	Solvent	cdcl3
3	Temperature	25.0
4	Pulse Sequence	s2pul
5	Experiment	1D
6	Probe	ASWPFG8319
7	Number of Scans	512
8	Receiver Gain	30
9	Relaxation Delay	2.0000
10	Pulse Width	5.7500
11	Spectrometer Frequency	100.52
12	Spectral Width	25000.0
13	Lowest Frequency	-1430.7
14	Nucleus	13C
15	Acquired Size	32768
16	Spectral Size	65536
17	Digital Resolution	0.38

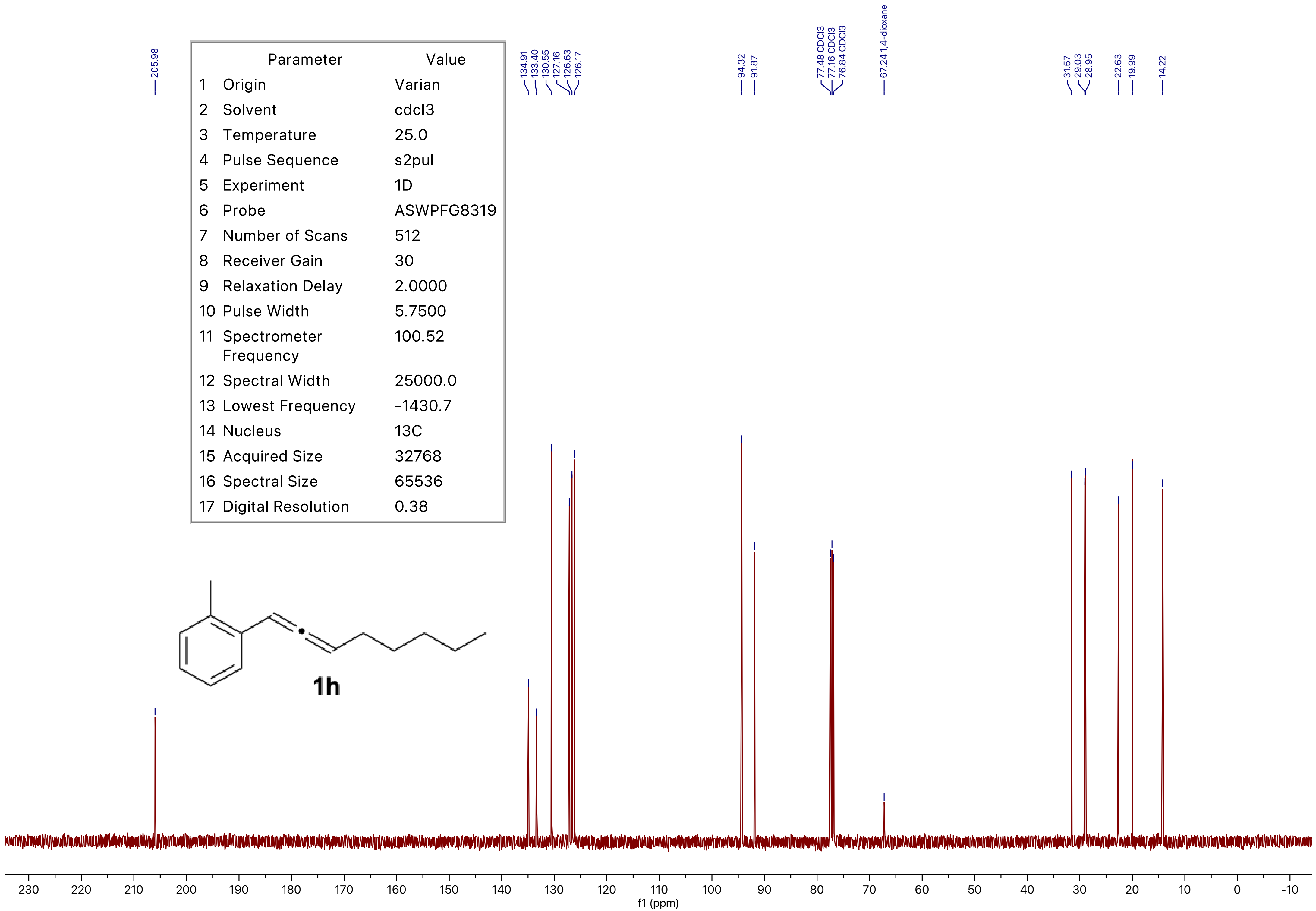

Parameter		Value
1	Origin	Varian
2	Solvent	cdcl3
3	Temperature	5.0
4	Pulse Sequence	s2pul
5	Experiment	1D
6	Probe	ASWPFG8319
7	Number of Scans	16
8	Receiver Gain	50
9	Relaxation Delay	5.0000
10	Pulse Width	7.7500
11	Spectrometer Frequency	399.73
12	Spectral Width	6410.3
13	Lowest Frequency	-804.7
14	Nucleus	1H
15	Acquired Size	16384
16	Spectral Size	65536
17	Digital Resolution	0.10

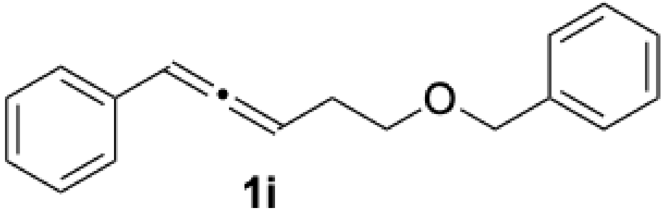

1i

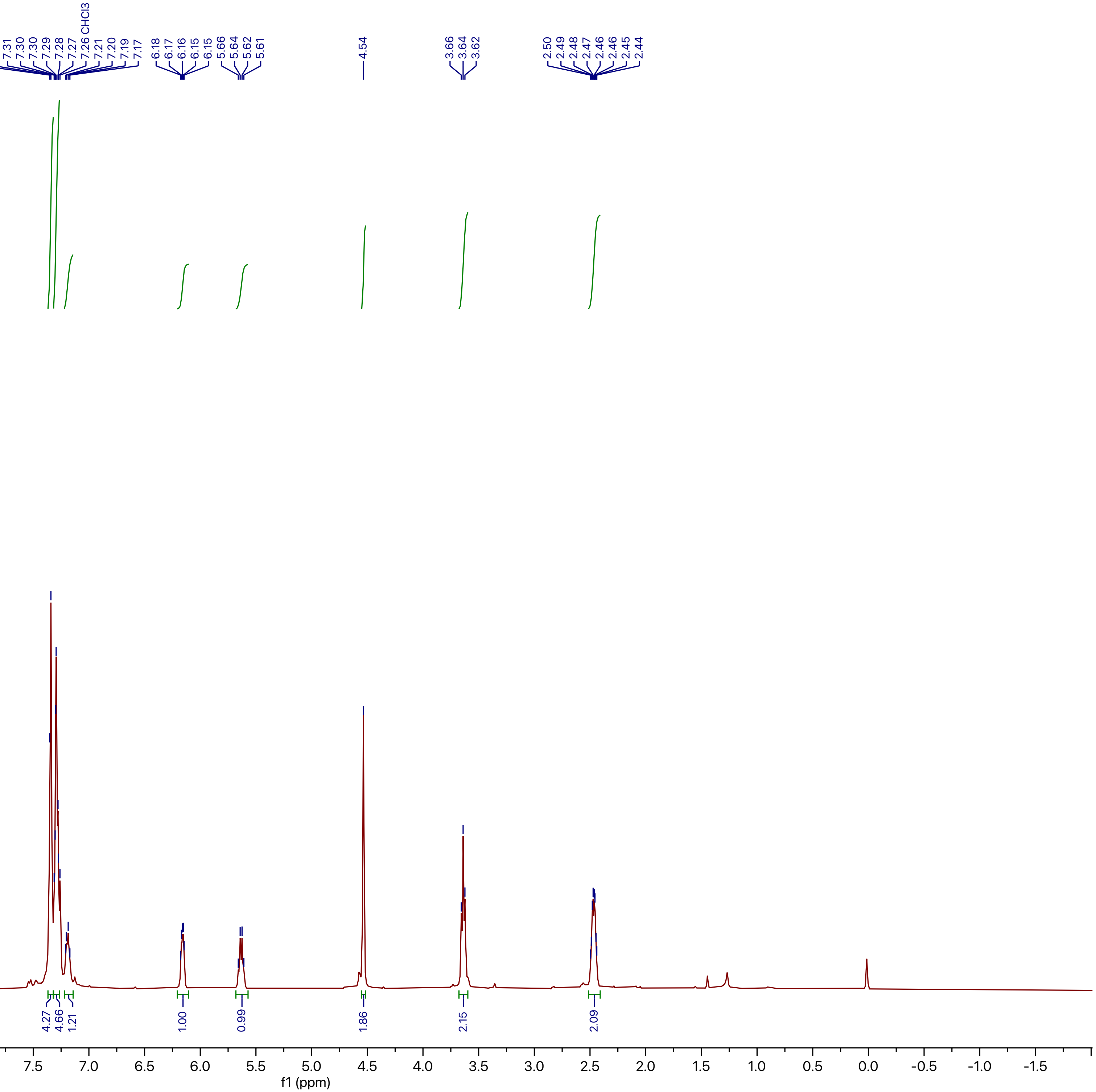

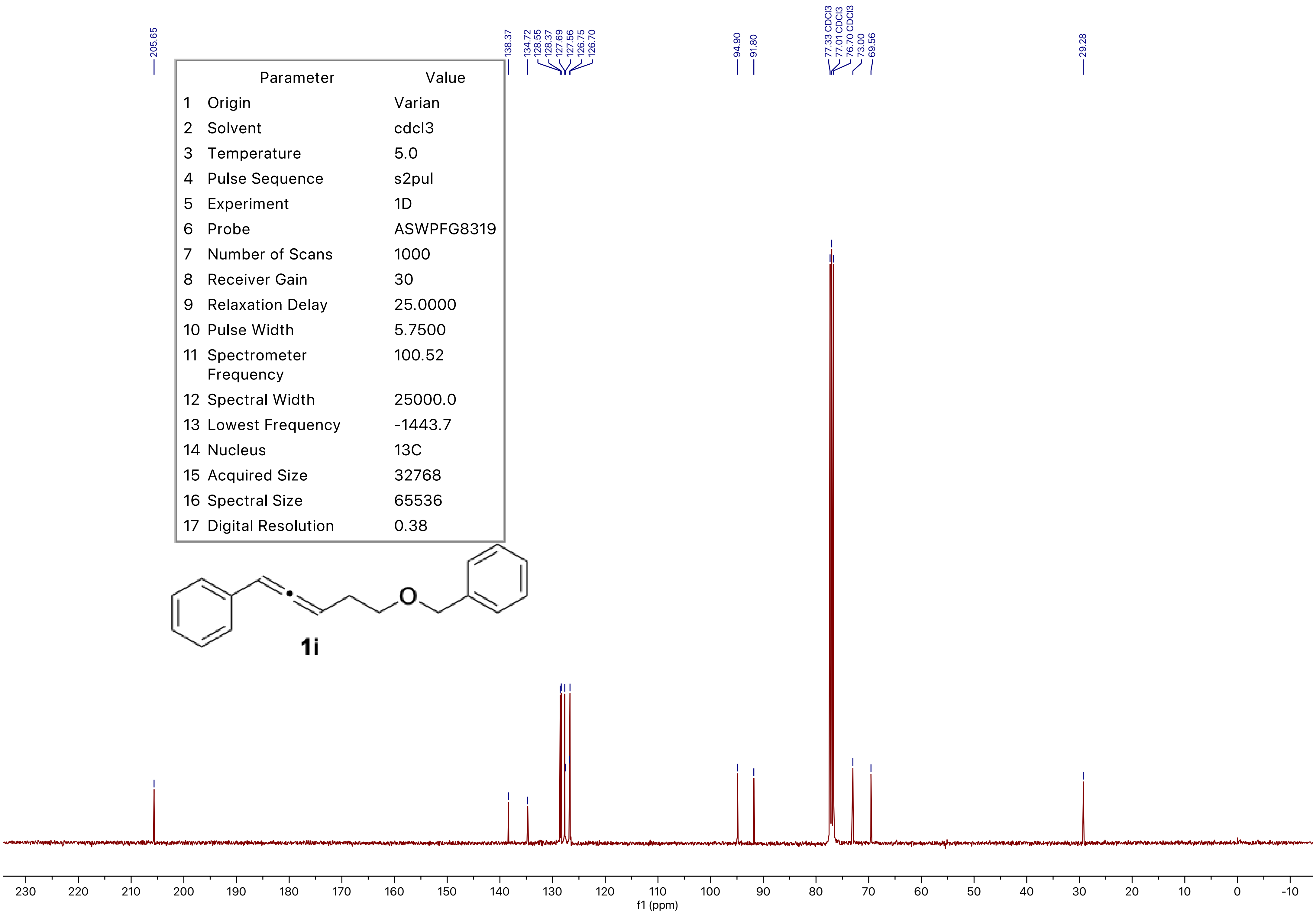

	Parameter	Value
1	Origin	Varian
2	Solvent	cdcl3
3	Temperature	5.0
4	Pulse Sequence	s2pul
5	Experiment	1D
6	Probe	ASWPFG8319
7	Number of Scans	1000
8	Receiver Gain	30
9	Relaxation Delay	25.0000
10	Pulse Width	5.7500
11	Spectrometer Frequency	100.52
12	Spectral Width	25000.0
13	Lowest Frequency	-1443.7
14	Nucleus	13C
15	Acquired Size	32768
16	Spectral Size	65536
17	Digital Resolution	0.38

1i

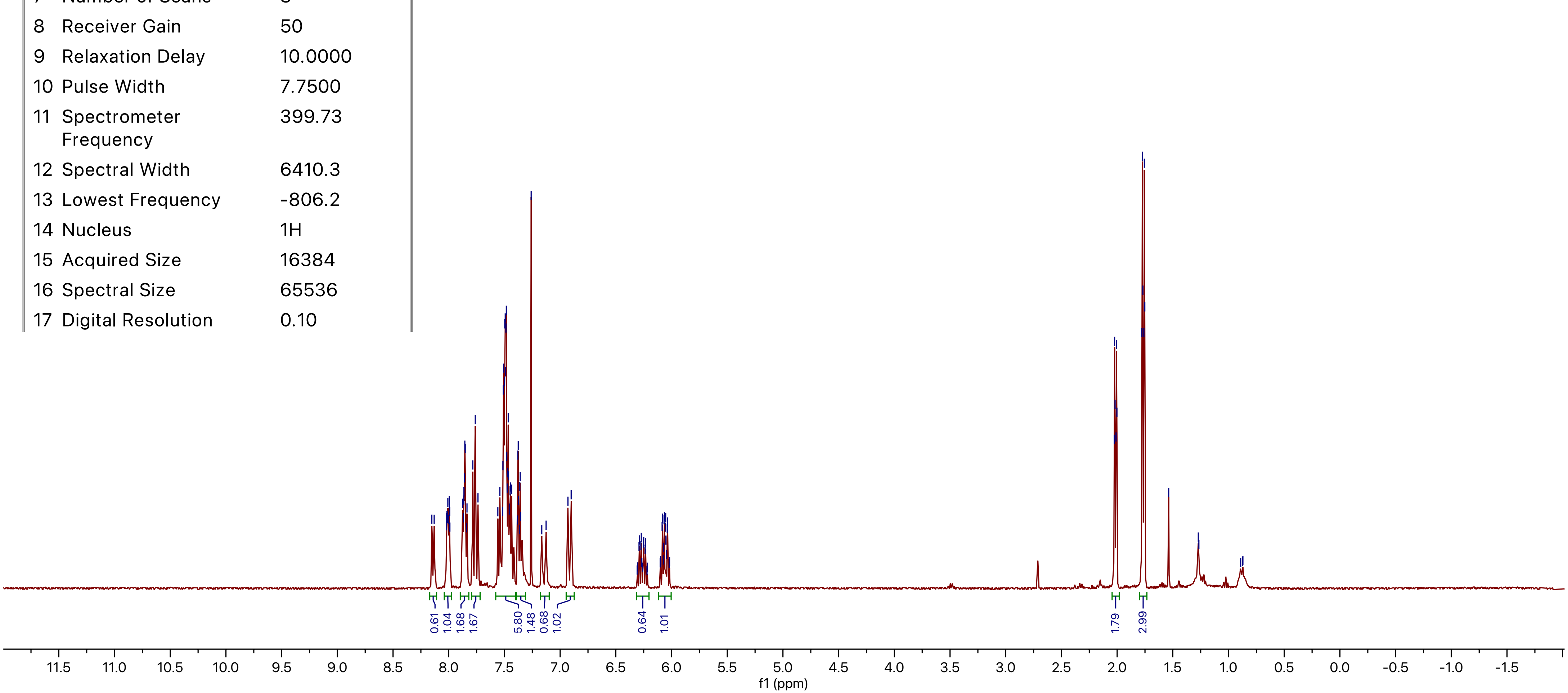

	Parameter	Value
1	Origin	Varian
2	Solvent	cdcl3
3	Temperature	-30.0
4	Pulse Sequence	s2pul
5	Experiment	1D
6	Probe	ASWPFG8319
7	Number of Scans	8
8	Receiver Gain	50
9	Relaxation Delay	10.0000
10	Pulse Width	7.7500
11	Spectrometer Frequency	399.73
12	Spectral Width	6410.3
13	Lowest Frequency	-806.2
14	Nucleus	1H
15	Acquired Size	16384
16	Spectral Size	65536
17	Digital Resolution	0.10

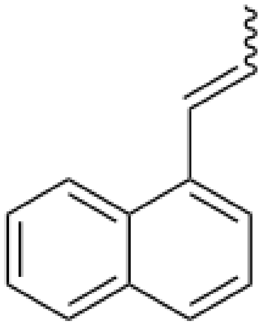

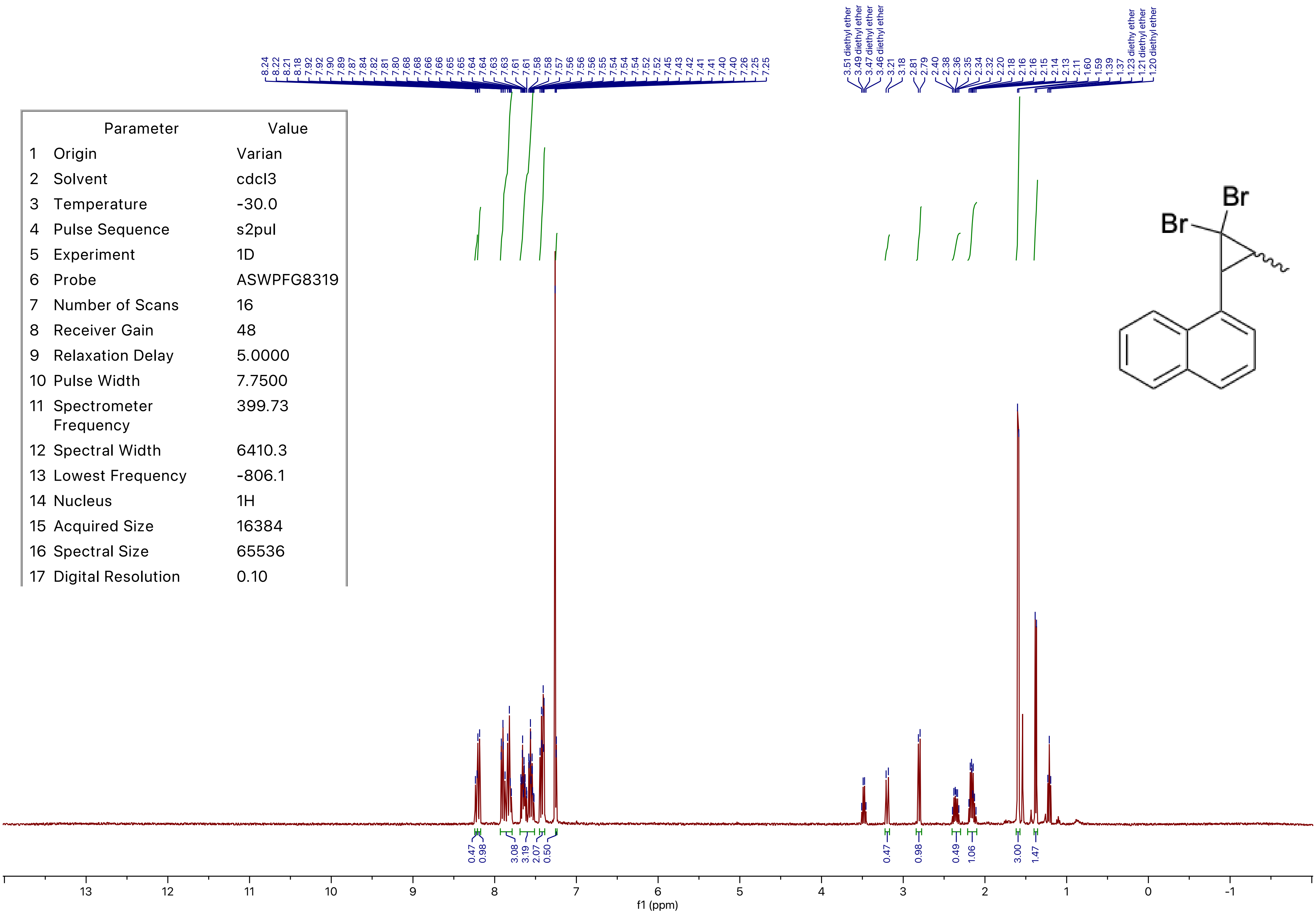
Parameter Value
1 Origin Varian
2 Solvent cdcl3
3 Temperature -30.0
4 Pulse Sequence s2pul
5 Experiment 1D
6 Probe ASWPFG8319
7 Number of Scans 16
8 Receiver Gain 48
9 Relaxation Delay 5.0000
10 Pulse Width 7.7500
11 Spectrometer Frequency 399.73
12 Spectral Width 6410.3
13 Lowest Frequency -806.1
14 Nucleus 1H
15 Acquired Size 16384
16 Spectral Size 65536
17 Digital Resolution 0.10
8.24 8.22 8.21 8.18 7.92 7.92 7.90 7.89 7.87 7.84 7.82 7.81 7.80 7.68 7.68 7.66 7.66 7.65 7.65 7.64 7.64 7.63 7.63 7.61 7.61 7.58 7.58 7.57 7.56 7.56 7.55 7.54 7.54 7.54 7.52 7.52 7.45 7.43 7.42 7.41 7.41 7.40 7.40 7.26 7.25 7.25
3.51 diethyl ether
3.49 diethyl ether
3.47 diethyl ether
3.46 diethyl ether
3.21 3.18 2.81 2.79 2.40 2.38 2.36 2.35 2.34 2.32 2.20 2.18 2.16 2.16 2.15 2.14 2.13 2.11 1.60 1.59 1.39 1.37
1.23 diethy ether
1.21 diethyl ether
1.20 diethyl ether
Br
Br
0.47 0.98 3.08 3.19 2.07 0.50 0.47 0.98 0.49 1.06 3.00 1.47
13 12 11 10 9 8 7 6 5 4 3 2 1 0 -1
f1 (ppm)

	Parameter	Value
1	Origin	Varian
2	Solvent	cdcl3
3	Temperature	-30.0
4	Pulse Sequence	s2pul
5	Experiment	1D
6	Probe	ASWPFG8319
7	Number of Scans	16
8	Receiver Gain	54
9	Relaxation Delay	2.0000
10	Pulse Width	7.7500
11	Spectrometer Frequency	399.73
12	Spectral Width	6410.3
13	Lowest Frequency	-805.9
14	Nucleus	1H
15	Acquired Size	16384
16	Spectral Size	65536
17	Digital Resolution	0.10

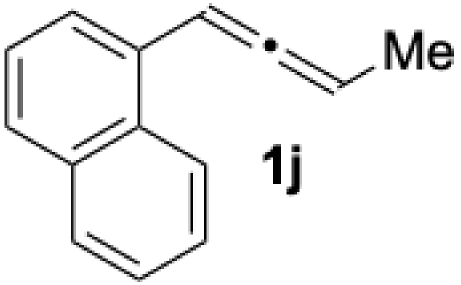

1j

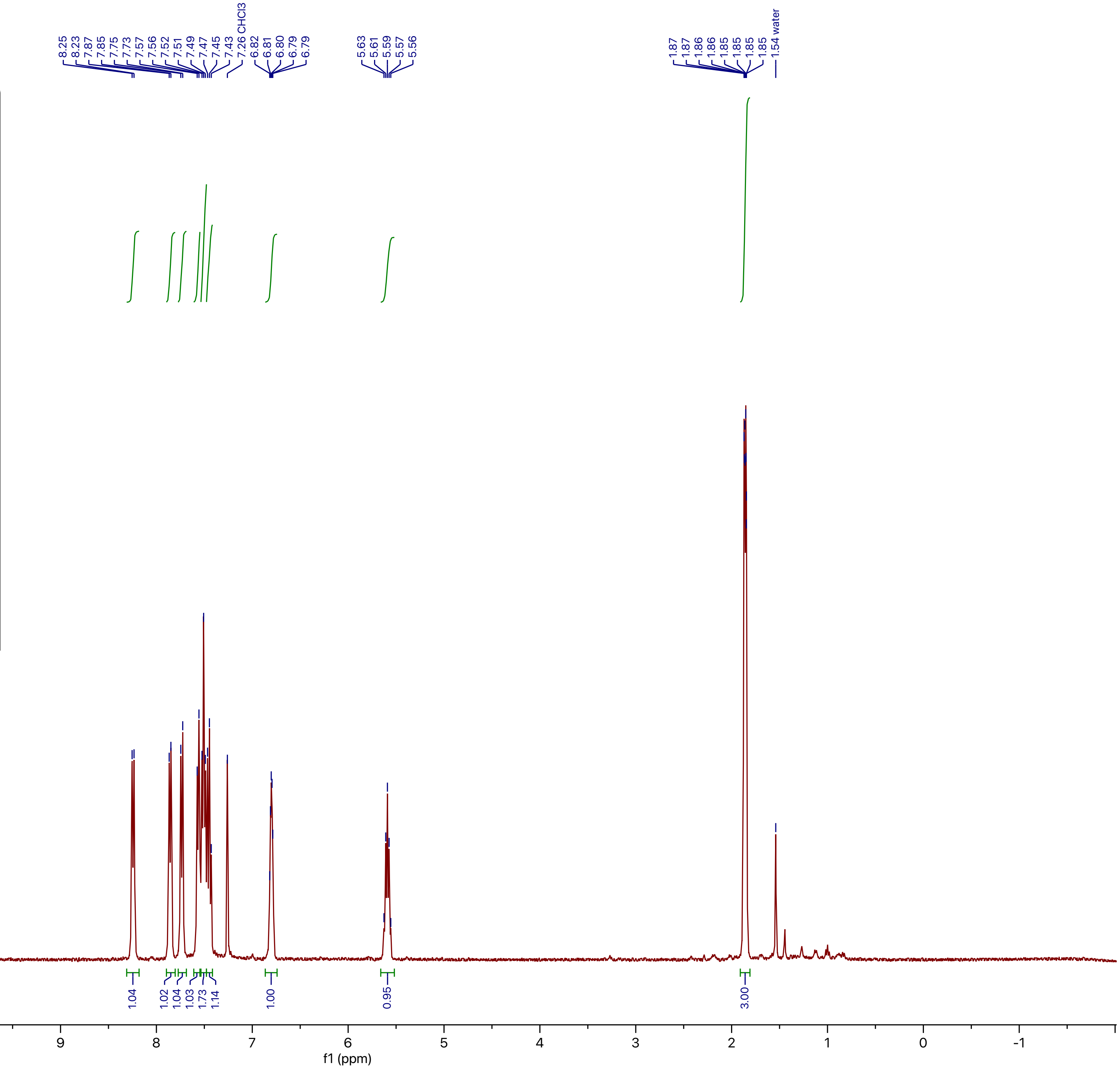

	Parameter	Value
1	Origin	Varian
2	Solvent	cdcl3
3	Temperature	-30.0
4	Pulse Sequence	s2pul
5	Experiment	1D
6	Probe	ASWPFG8319
7	Number of Scans	256
8	Receiver Gain	30
9	Relaxation Delay	1.0000
10	Pulse Width	5.7500
11	Spectrometer Frequency	100.52
12	Spectral Width	25000.0
13	Lowest Frequency	-1443.7
14	Nucleus	13C
15	Acquired Size	32768
16	Spectral Size	65536
17	Digital Resolution	0.38

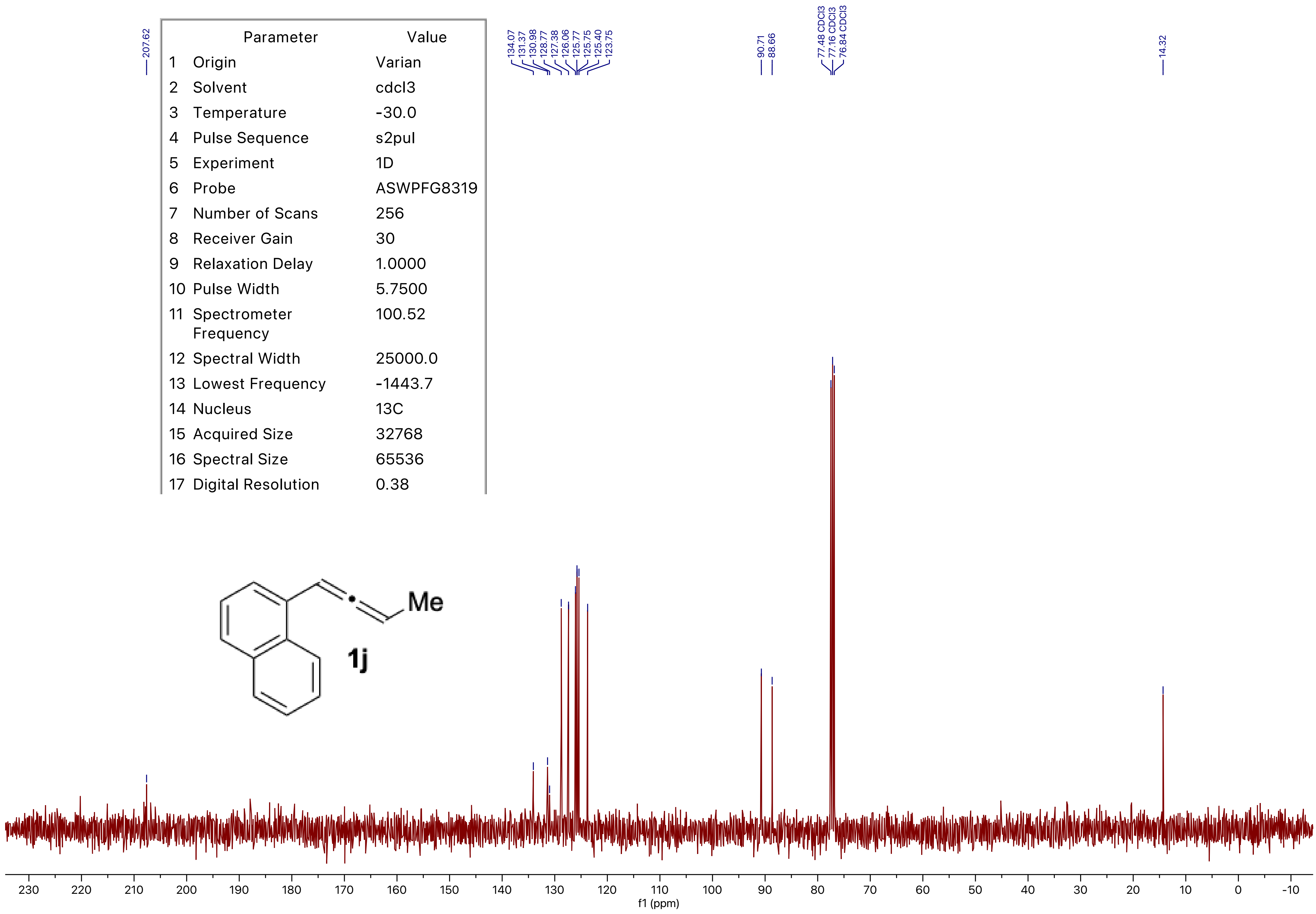

	Parameter	Value
1	Origin	Varian
2	Solvent	cdcl3
3	Temperature	25.0
4	Pulse Sequence	s2pul
5	Experiment	1D
6	Probe	ASWPFG8319
7	Number of Scans	16
8	Receiver Gain	38
9	Relaxation Delay	5.0000
10	Pulse Width	7.7500
11	Spectrometer Frequency	399.73
12	Spectral Width	6410.3
13	Lowest Frequency	-806.7
14	Nucleus	1H
15	Acquired Size	16384
16	Spectral Size	65536
17	Digital Resolution	0.10

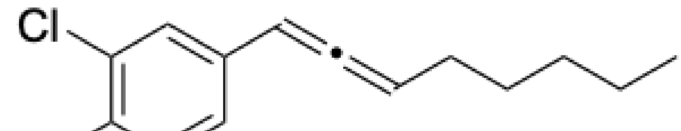

1k

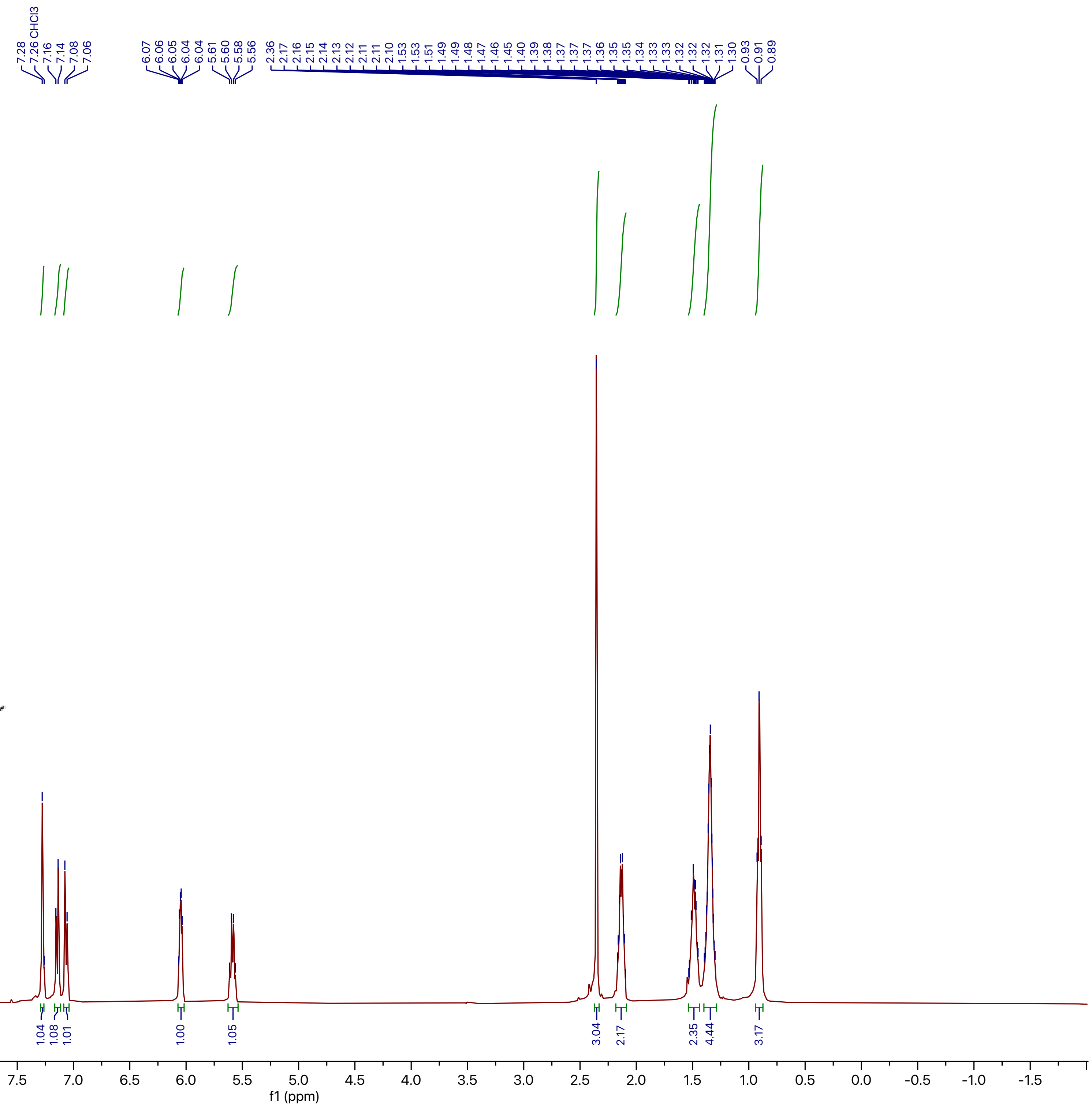

	Parameter	Value
1	Origin	Varian
2	Solvent	cdcl3
3	Temperature	25.0
4	Pulse Sequence	s2pul
5	Experiment	1D
6	Probe	ASWPFG8319
7	Number of Scans	512
8	Receiver Gain	30
9	Relaxation Delay	3.0000
10	Pulse Width	5.7500
11	Spectrometer Frequency	100.52
12	Spectral Width	25000.0
13	Lowest Frequency	-1443.7
14	Nucleus	13C
15	Acquired Size	32768
16	Spectral Size	65536
17	Digital Resolution	0.38

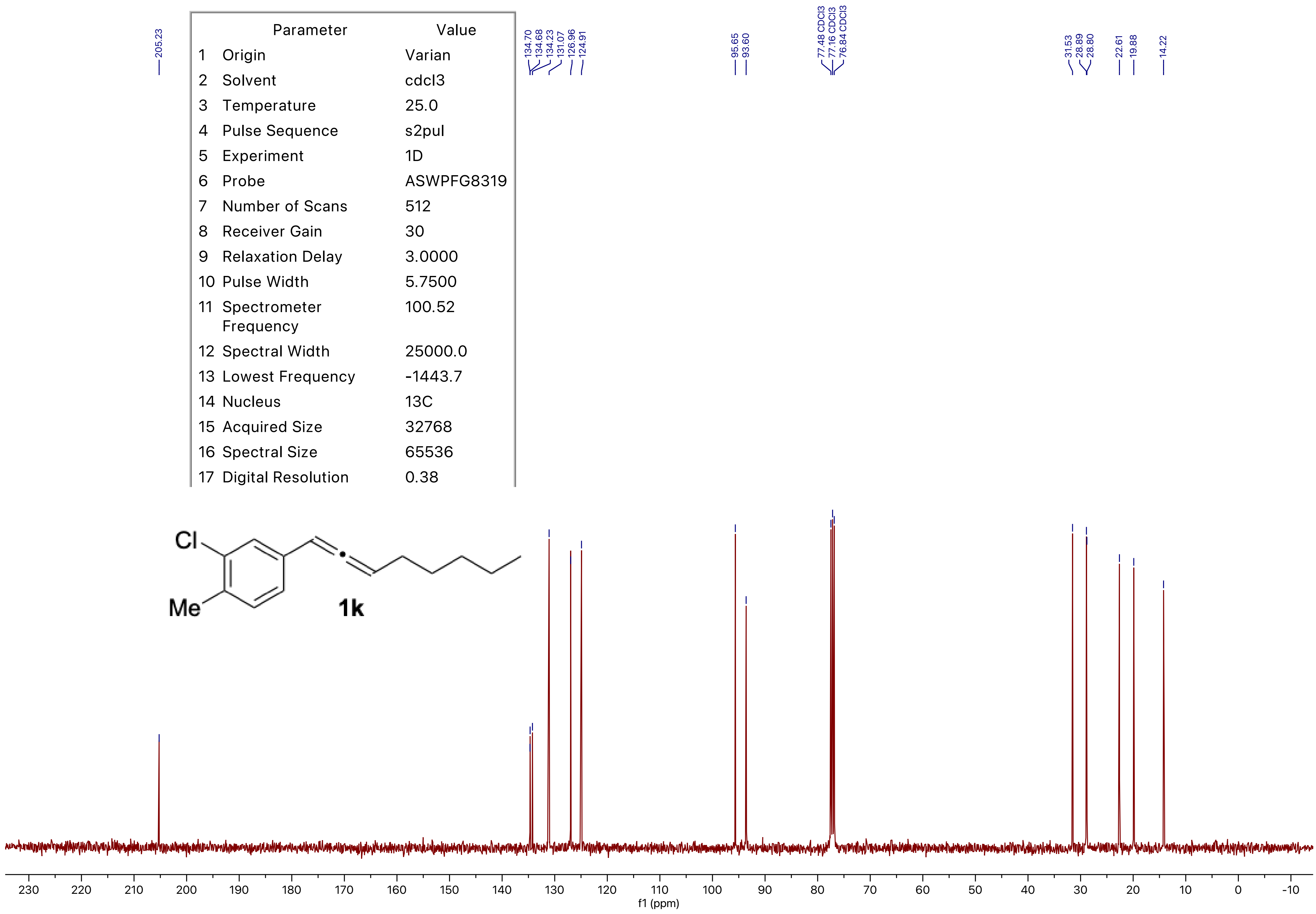

	Parameter	Value
1	Origin	Varian
2	Solvent	cdcl3
3	Temperature	0.0
4	Pulse Sequence	s2pul
5	Experiment	1D
6	Probe	ASWPFG8319
7	Number of Scans	32
8	Receiver Gain	44
9	Relaxation Delay	5.0000
10	Pulse Width	6.4000
11	Spectrometer Frequency	399.73
12	Spectral Width	6410.3
13	Lowest Frequency	-806.3
14	Nucleus	1H
15	Acquired Size	16384
16	Spectral Size	65536
17	Digital Resolution	0.10

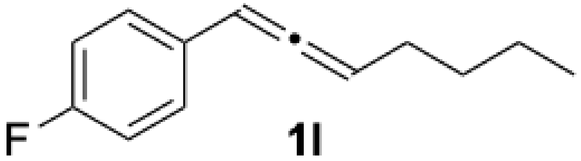

1l

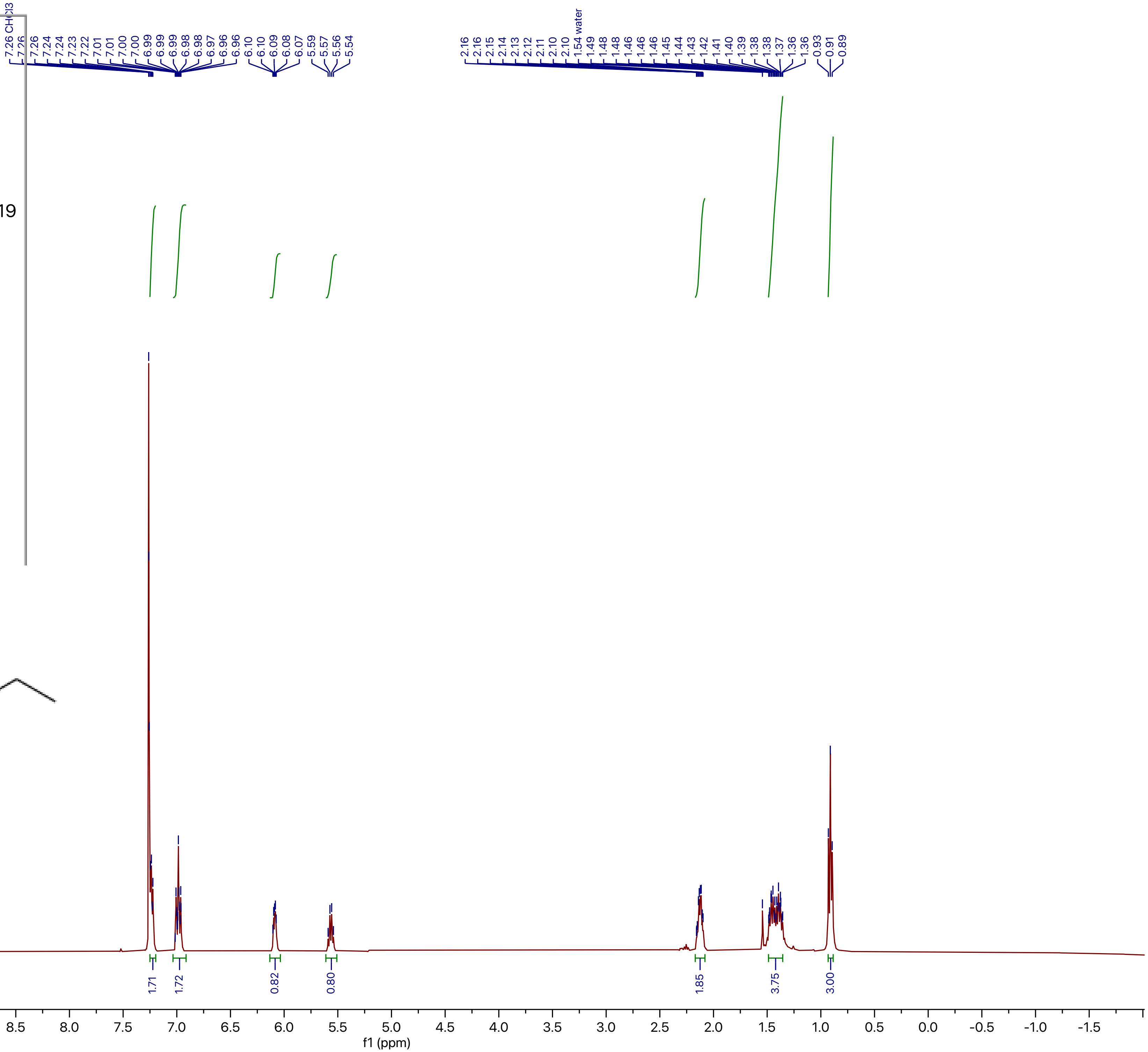

	Parameter	Value
1	Origin	Bruker BioSpin GmbH
2	Instrument	Avance
3	Solvent	CDCl3
4	Temperature	300.0
5	Pulse Sequence	zg30
6	Experiment	1D
7	Probe	Z151574_0073 (PI HR-BBO500S1-BBF/ H/ D-5.0-Z SP)
8	Number of Scans	16
9	Receiver Gain	101.0
10	Relaxation Delay	1.0000
11	Pulse Width	8.0000
12	Spectrometer Frequency	500.21
13	Spectral Width	10000.0
14	Lowest Frequency	-1922.5
15	Nucleus	1H
16	Acquired Size	32768
17	Spectral Size	65536

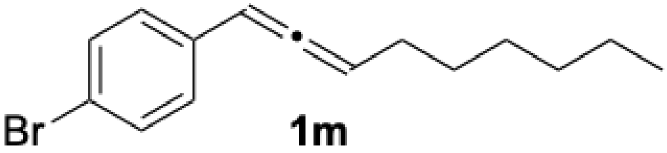

1m

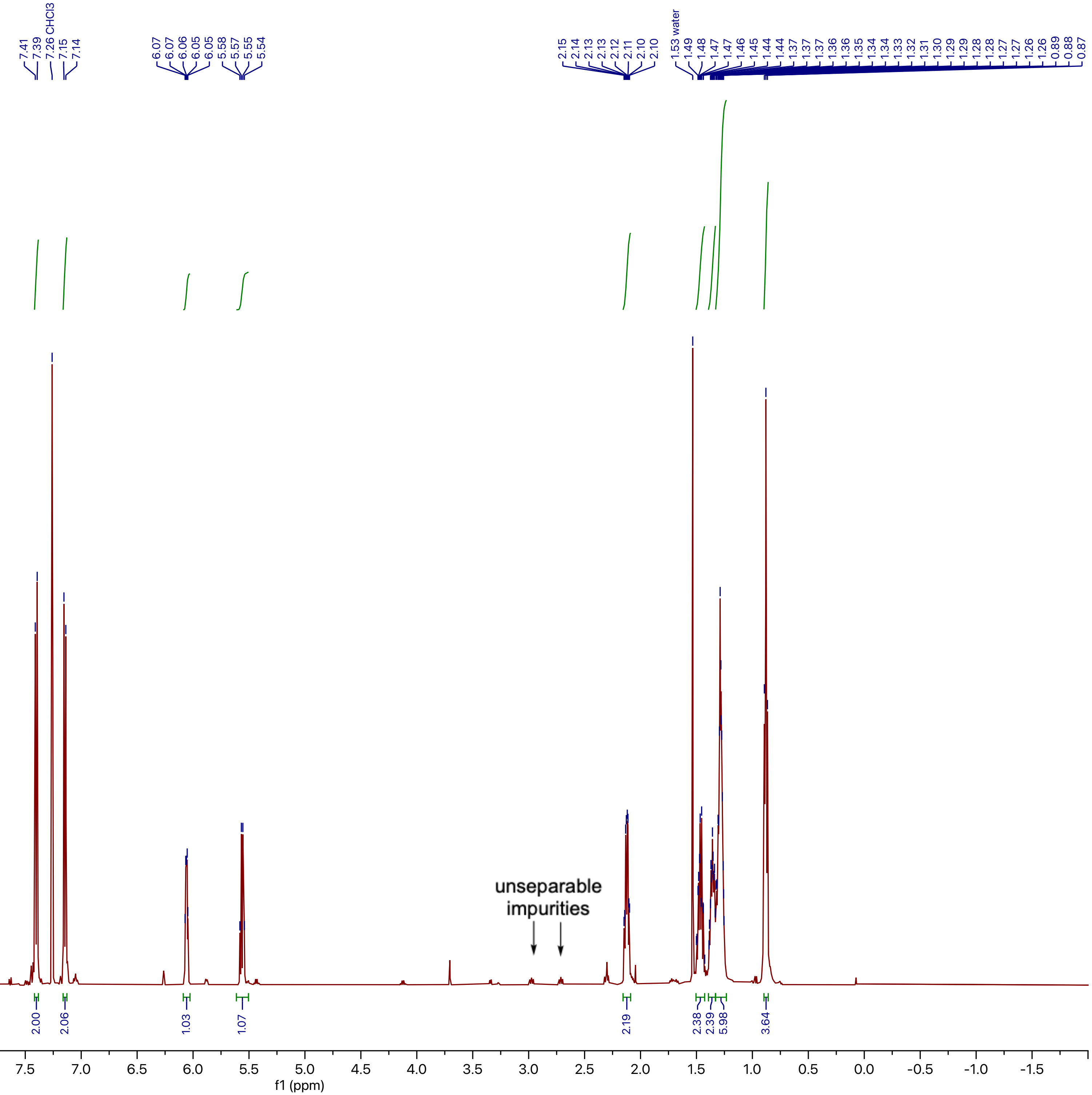

	Parameter	Value
1	Origin	Bruker BioSpin GmbH
2	Instrument	Avance
3	Solvent	CDCl3
4	Temperature	300.0
5	Pulse Sequence	zgpg30
6	Experiment	1D
7	Probe	Z151574_0073 (PI HR-BBO500S1-BBF/ H/ D-5.0-Z SP)
8	Number of Scans	1024
9	Receiver Gain	101.0
10	Relaxation Delay	2.0000
11	Pulse Width	9.0000
12	Spectrometer Frequency	125.79
13	Spectral Width	30120.5
14	Lowest Frequency	-2463.4
15	Nucleus	13C
16	Acquired Size	32768
17	Spectral Size	65536

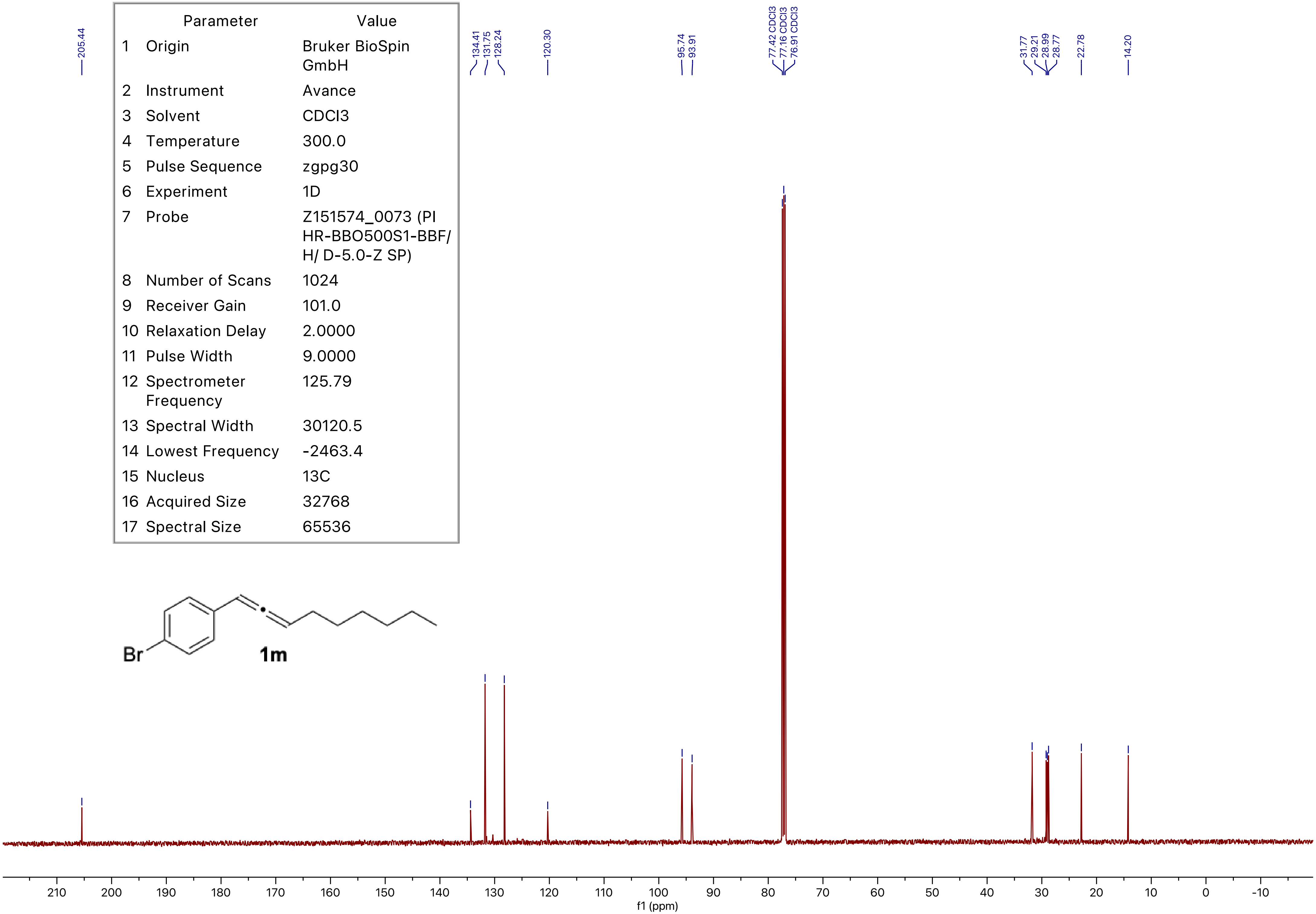

	Parameter	Value
1	Origin	Varian
2	Solvent	cdcl3
3	Temperature	0.0
4	Pulse Sequence	s2pul
5	Experiment	1D
6	Probe	ASWPFG8319
7	Number of Scans	16
8	Receiver Gain	54
9	Relaxation Delay	5.0000
10	Pulse Width	7.7500
11	Spectrometer Frequency	399.73
12	Spectral Width	6410.3
13	Lowest Frequency	-800.5
14	Nucleus	1H
15	Acquired Size	16384
16	Spectral Size	65536
17	Digital Resolution	0.10

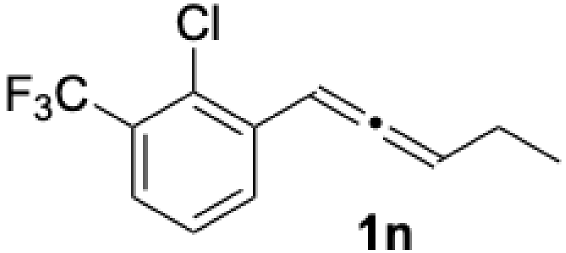

1n

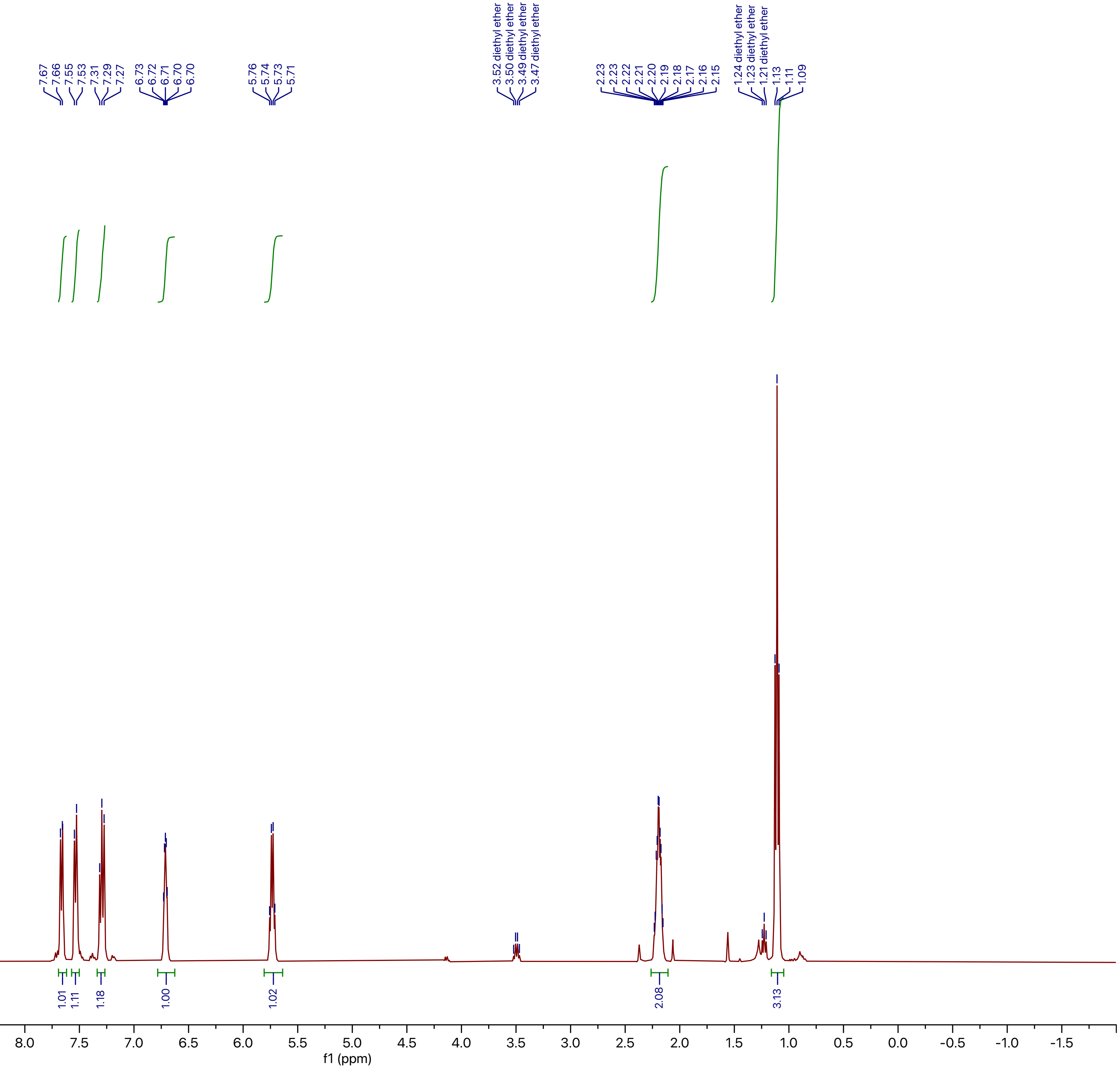

	Parameter	Value
1	Origin	Varian
2	Solvent	cdcl3
3	Temperature	0.0
4	Pulse Sequence	s2pul
5	Experiment	1D
6	Probe	ASWPFG8319
7	Number of Scans	256
8	Receiver Gain	30
9	Relaxation Delay	2.0000
10	Pulse Width	5.7500
11	Spectrometer Frequency	100.52
12	Spectral Width	25000.0
13	Lowest Frequency	-1443.7
14	Nucleus	13C
15	Acquired Size	32768
16	Spectral Size	65536
17	Digital Resolution	0.38

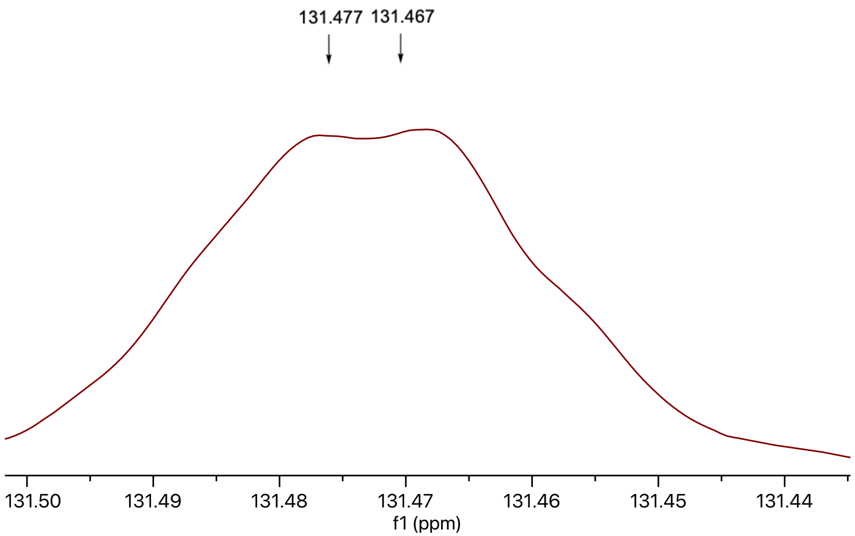

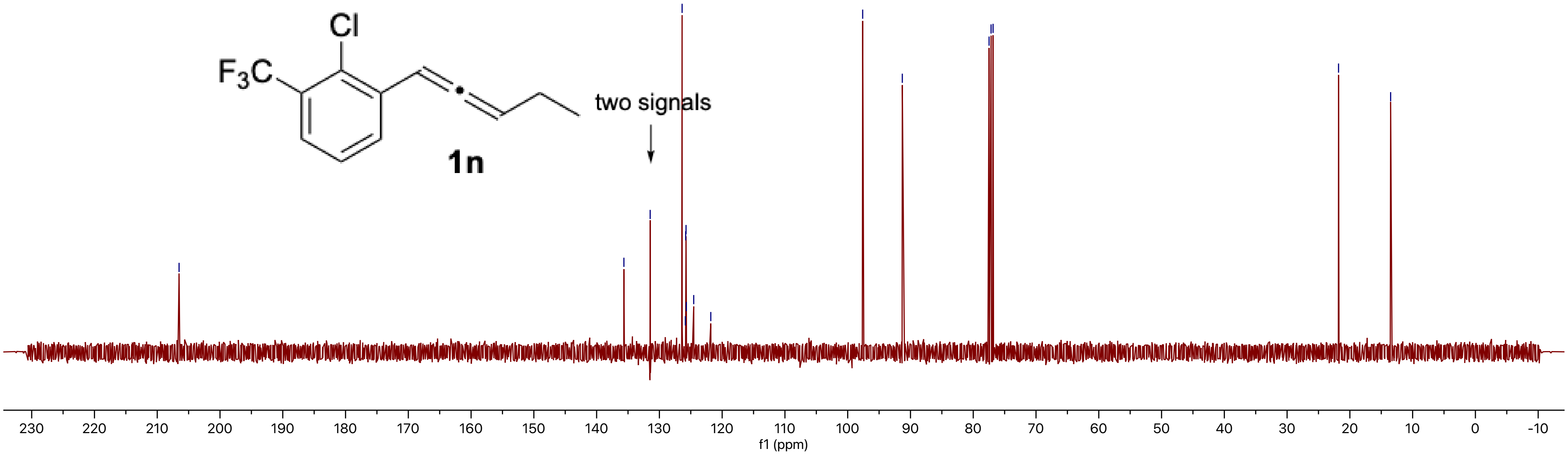

	Parameter	Value
1	Origin	Varian
2	Solvent	cdcl3
3	Temperature	0.0
4	Pulse Sequence	s2pul
5	Experiment	1D
6	Probe	ASWPFG8319
7	Number of Scans	16
8	Receiver Gain	60
9	Relaxation Delay	1.0000
10	Pulse Width	5.0000
11	Spectrometer Frequency	376.09
12	Spectral Width	89285.7
13	Lowest Frequency	-76613.1
14	Nucleus	19F
15	Acquired Size	65536
16	Spectral Size	131072
17	Digital Resolution	0.68

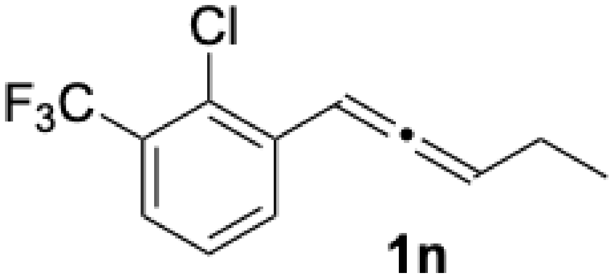

1n

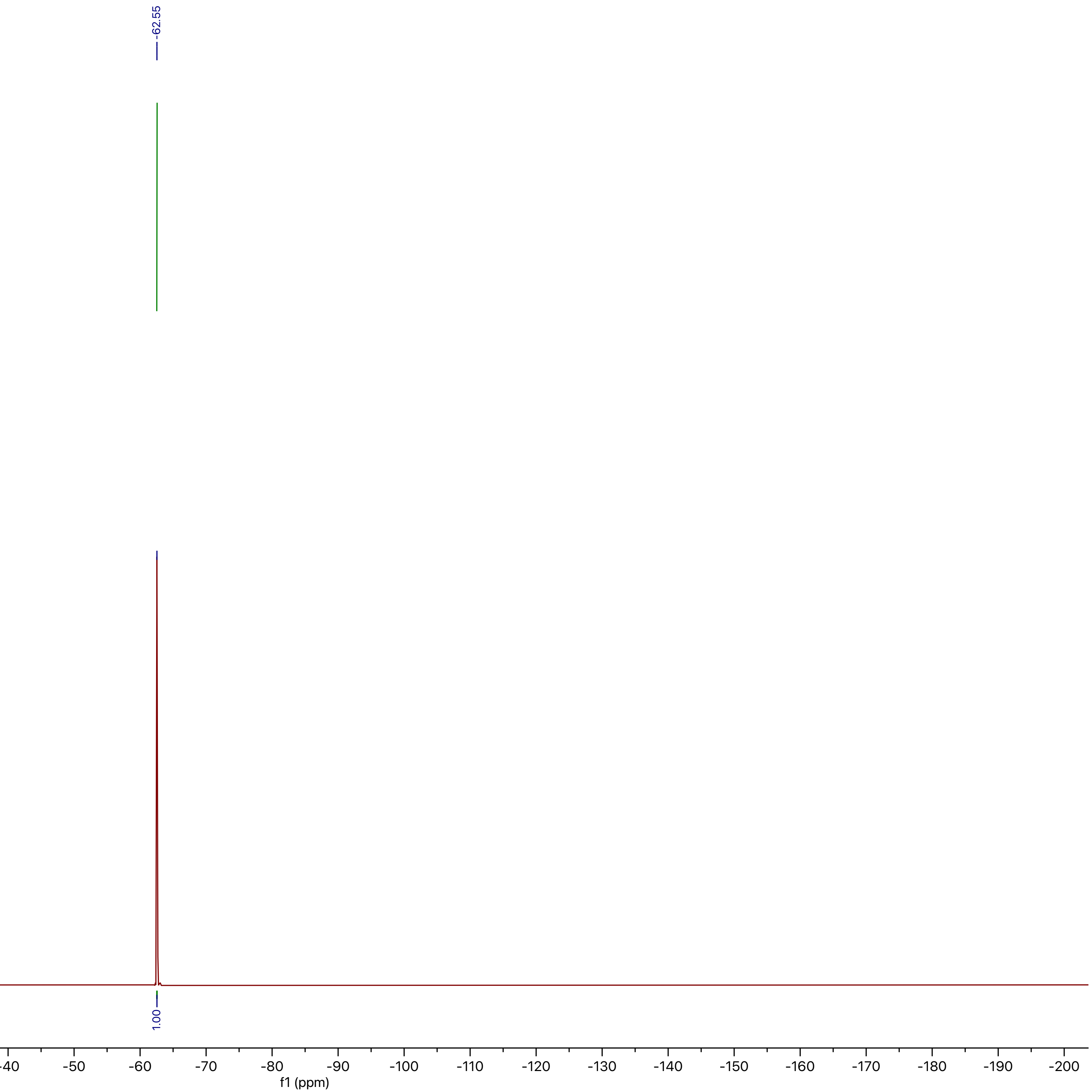

	Parameter	Value
1	Origin	Varian
2	Solvent	cdcl3
3	Temperature	25.0
4	Pulse Sequence	s2pul
5	Experiment	1D
6	Probe	ASWPFG8319
7	Number of Scans	16
8	Receiver Gain	42
9	Relaxation Delay	5.0000
10	Pulse Width	7.7500
11	Spectrometer Frequency	399.73
12	Spectral Width	6410.3
13	Lowest Frequency	-806.1
14	Nucleus	1H
15	Acquired Size	16384
16	Spectral Size	65536
17	Digital Resolution	0.10

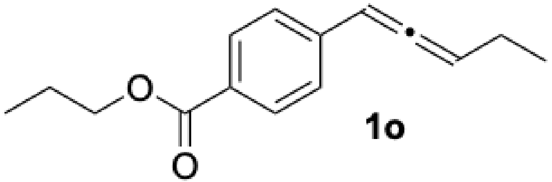

1o

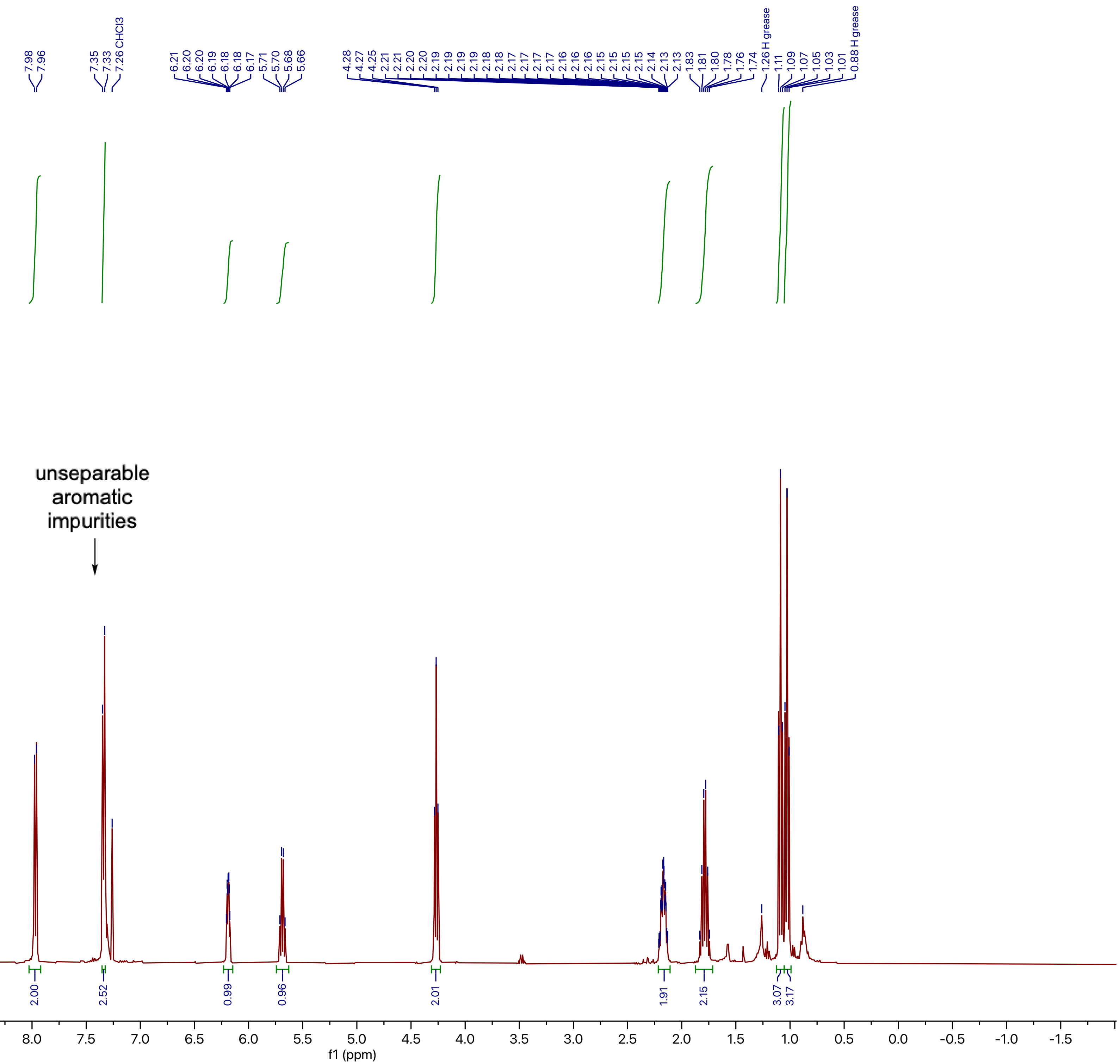

	Parameter	Value
1	Origin	Varian
2	Solvent	cdcl3
3	Temperature	25.0
4	Pulse Sequence	s2pul
5	Experiment	1D
6	Probe	ASWPFG8319
7	Number of Scans	512
8	Receiver Gain	30
9	Relaxation Delay	3.0000
10	Pulse Width	5.7500
11	Spectrometer Frequency	100.52
12	Spectral Width	25000.0
13	Lowest Frequency	-1428.8
14	Nucleus	13C
15	Acquired Size	32768
16	Spectral Size	65536
17	Digital Resolution	0.38

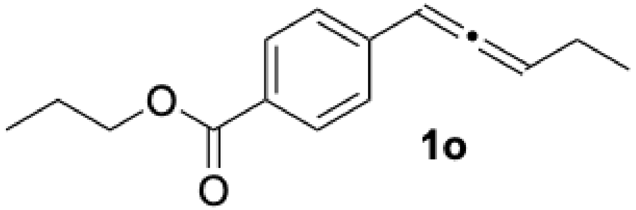

1o

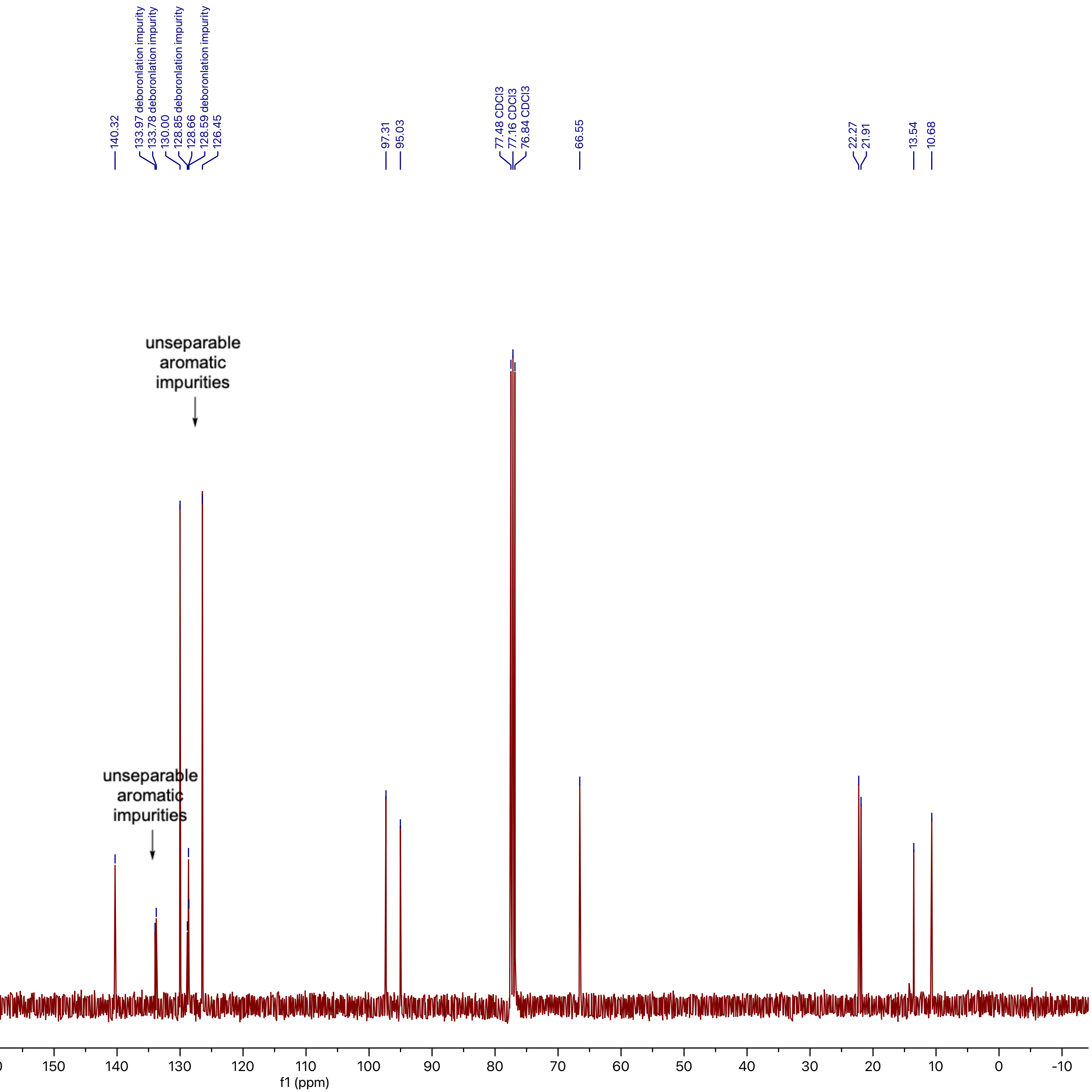

Parameter		Value
1	Origin	Bruker BioSpin GmbH
2	Instrument	Avance
3	Solvent	CDCl3
4	Temperature	298.0
5	Pulse Sequence	zg30
6	Experiment	1D
7	Probe	Z163739_0400 (PI HR-BBO400S1-BBF/ H/ D-5.0-Z SP)
8	Number of Scans	16
9	Receiver Gain	101.0
10	Relaxation Delay	1.0000
11	Pulse Width	8.0000
12	Spectrometer Frequency	400.13
13	Spectral Width	8196.7
14	Lowest Frequency	-1637.0
15	Nucleus	1H
16	Acquired Size	32768
17	Spectral Size	65536

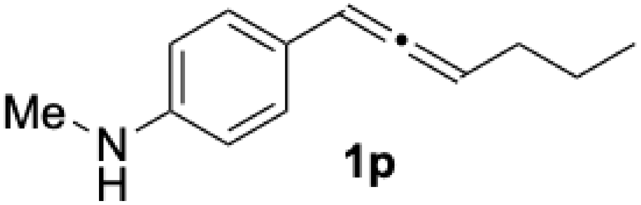

1p

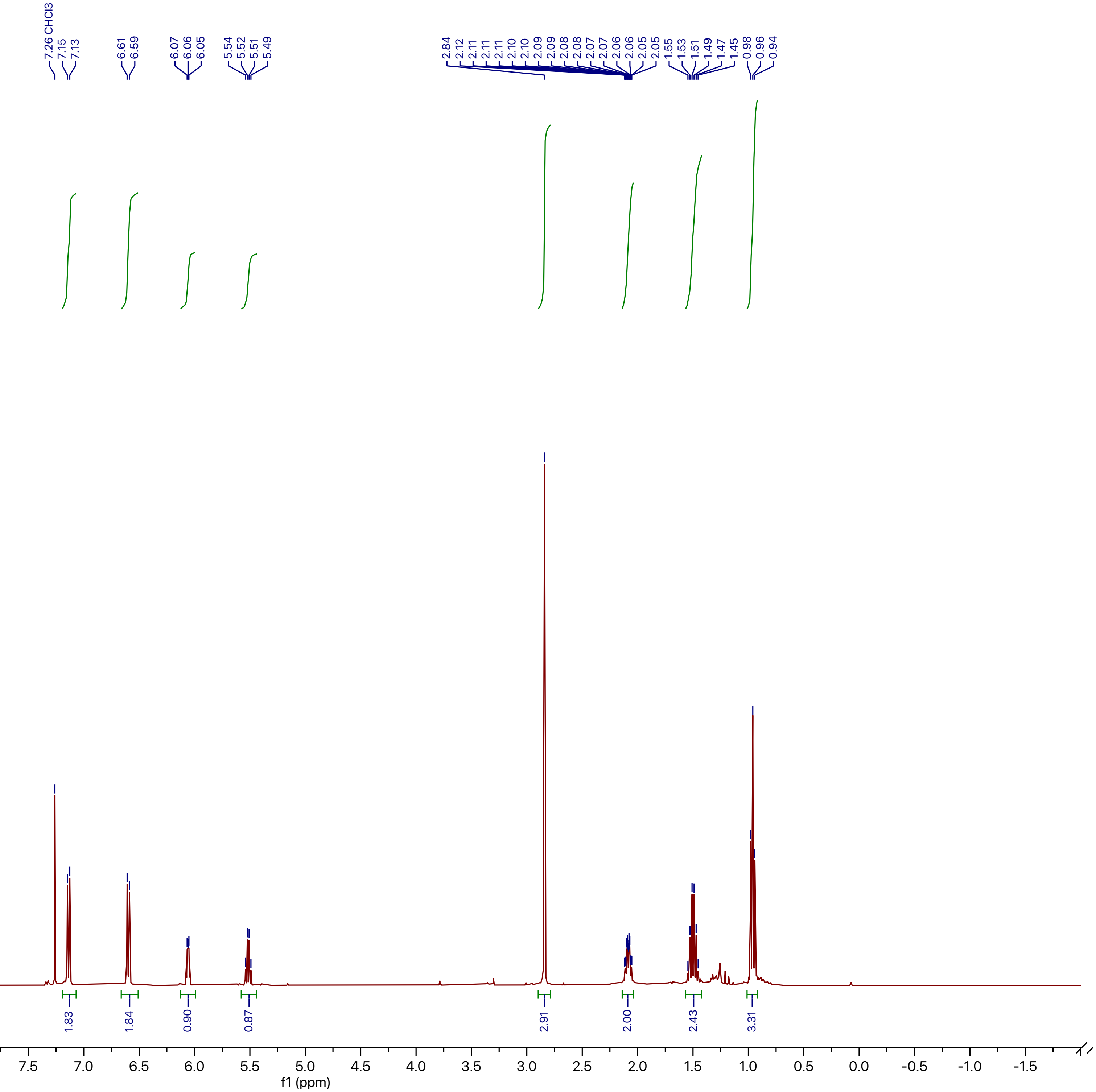

	Parameter	Value
1	Origin	Bruker BioSpin GmbH
2	Instrument	Avance
3	Solvent	CDCl3
4	Temperature	298.0
5	Pulse Sequence	zgpg30
6	Experiment	1D
7	Probe	Z163739_0400 (PI HR-BBO400S1-BBF/H/ D-5.0-Z SP)
8	Number of Scans	1024
9	Receiver Gain	101.0
10	Relaxation Delay	2.0000
11	Pulse Width	8.0000
12	Spectrometer Frequency	100.62
13	Spectral Width	23809.5
14	Lowest Frequency	-1843.5
15	Nucleus	13C
16	Acquired Size	32768
17	Spectral Size	65536

1p

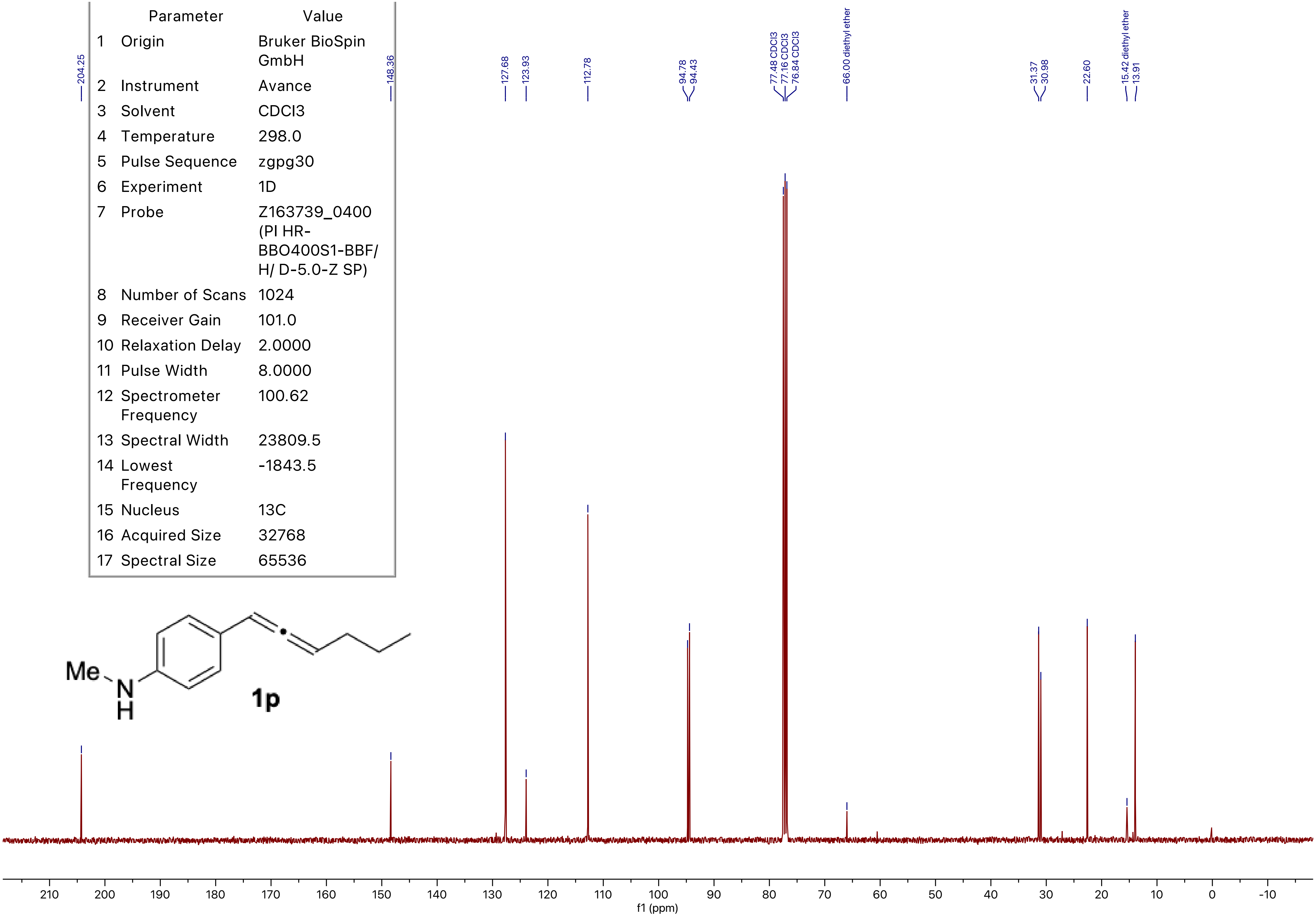

	Parameter	Value
1	Origin	Bruker BioSpin GmbH
2	Instrument	Avance
3	Solvent	CDCl3
4	Temperature	298.0
5	Pulse Sequence	zg30
6	Experiment	1D
7	Probe	Z163739_0400 (PI HR-BBO400S1-BBF/ H/ D-5.0-Z SP)
8	Number of Scans	16
9	Receiver Gain	101.0
10	Relaxation Delay	1.0000
11	Pulse Width	8.0000
12	Spectrometer Frequency	400.13
13	Spectral Width	8196.7
14	Lowest Frequency	-1637.0
15	Nucleus	1H
16	Acquired Size	32768
17	Spectral Size	65536

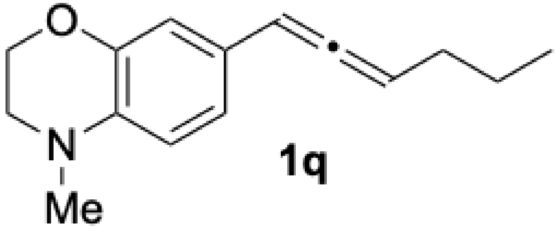

1q

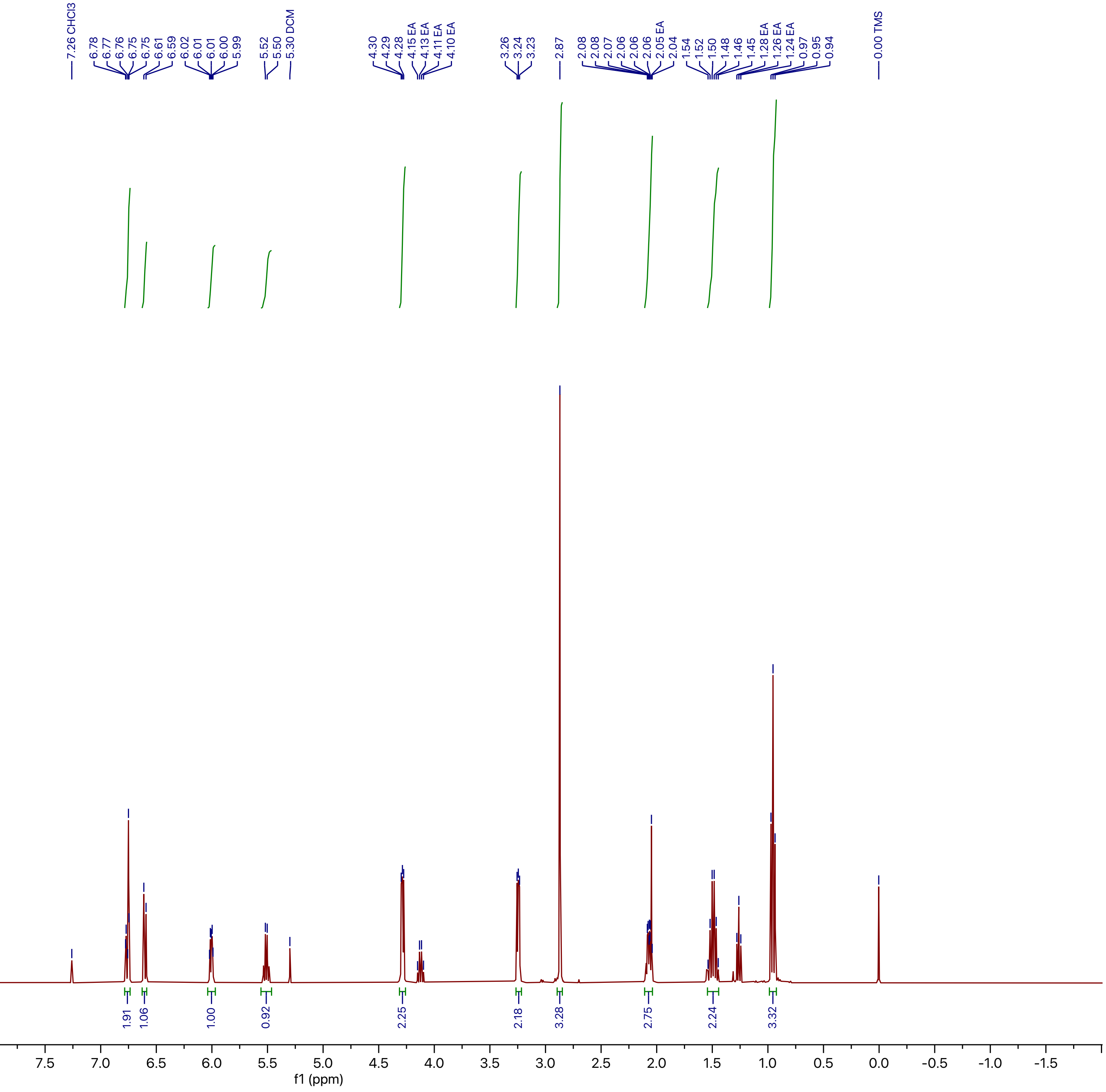

	Parameter	Value
1	Origin	Bruker BioSpin GmbH
2	Instrument	Avance
3	Solvent	CDCl3
4	Temperature	298.0
5	Pulse Sequence	zgpg30
6	Experiment	1D
7	Probe	Z163739_0400 (PI HR-BBO400S1-BBF/H/ D-5.0-Z SP)
8	Number of Scans	1024
9	Receiver Gain	101.0
10	Relaxation Delay	2.0000
11	Pulse Width	8.0000
12	Spectrometer Frequency	100.62
13	Spectral Width	23809.5
14	Lowest Frequency	-1843.5
15	Nucleus	13C
16	Acquired Size	32768
17	Spectral Size	65536

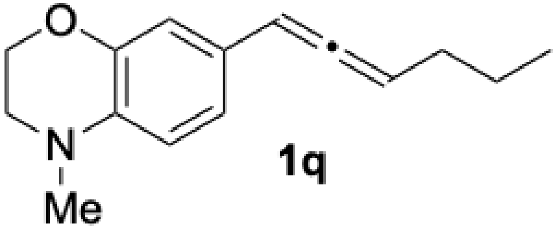

1q

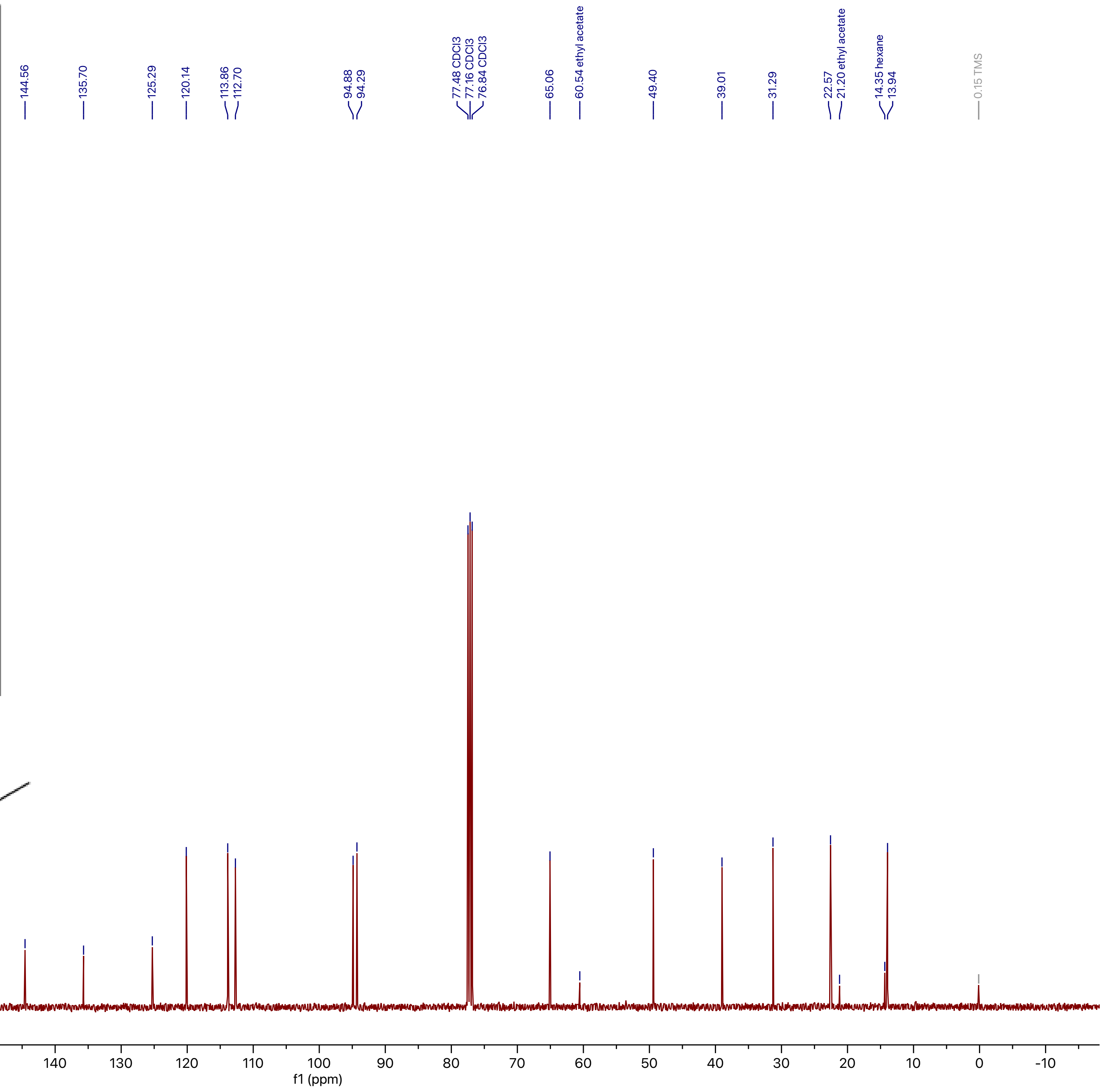

	Parameter	Value
1	Origin	Bruker BioSpin GmbH
2	Instrument	Avance
3	Solvent	CDCl3
4	Temperature	298.0
5	Pulse Sequence	zg30
6	Experiment	1D
7	Probe	Z163739_0400 (PI HR-BBO400S1-BBF/ H/ D-5.0-Z SP)
8	Number of Scans	16
9	Receiver Gain	101.0
10	Relaxation Delay	1.0000
11	Pulse Width	8.0000
12	Spectrometer Frequency	400.13
13	Spectral Width	8196.7
14	Lowest Frequency	-1667.6
15	Nucleus	1H
16	Acquired Size	32768
17	Spectral Size	65536

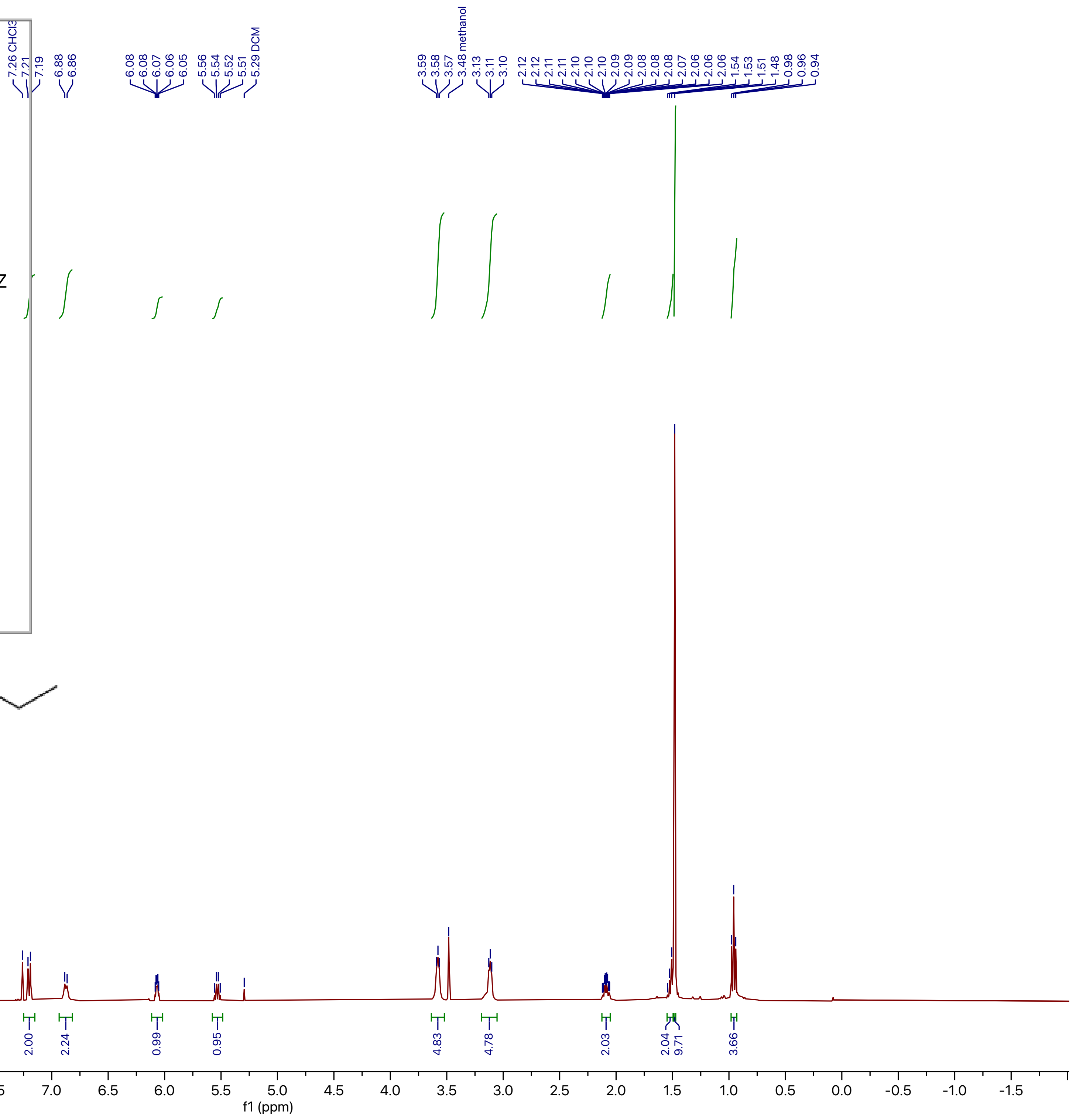

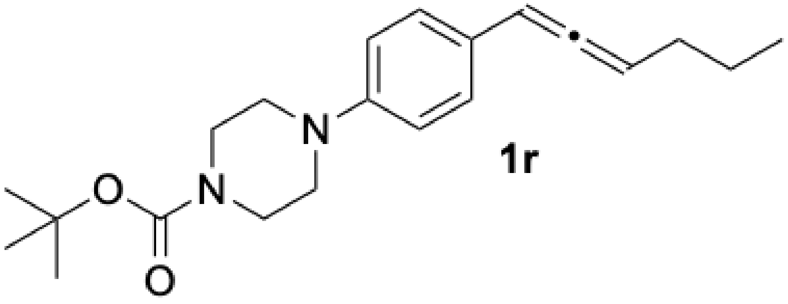

1r

	Parameter	Value
1	Origin	Bruker BioSpin GmbH
2	Instrument	Avance
3	Solvent	CDCl3
4	Temperature	298.0
5	Pulse Sequence	zgpg30
6	Experiment	1D
7	Probe	Z163739_0400 (PI HR-BBO400S1-BBF/ H/ D-5.0-Z SP)
8	Number of Scans	1024
9	Receiver Gain	101.0
10	Relaxation Delay	2.0000
11	Pulse Width	8.0000
12	Spectrometer Frequency	100.62
13	Spectral Width	23809.5
14	Lowest Frequency	-1830.0
15	Nucleus	13C
16	Acquired Size	32768
17	Spectral Size	65536

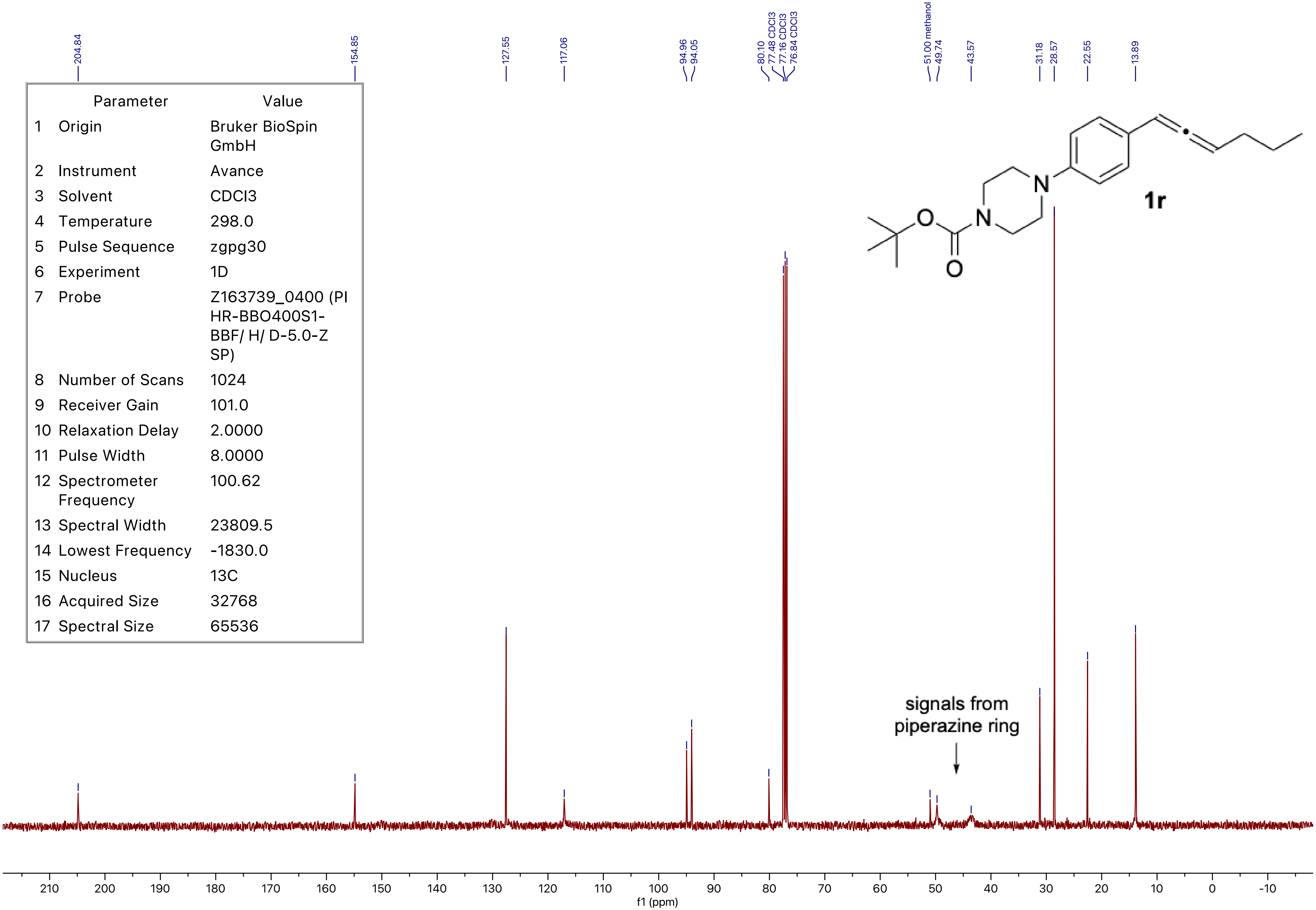

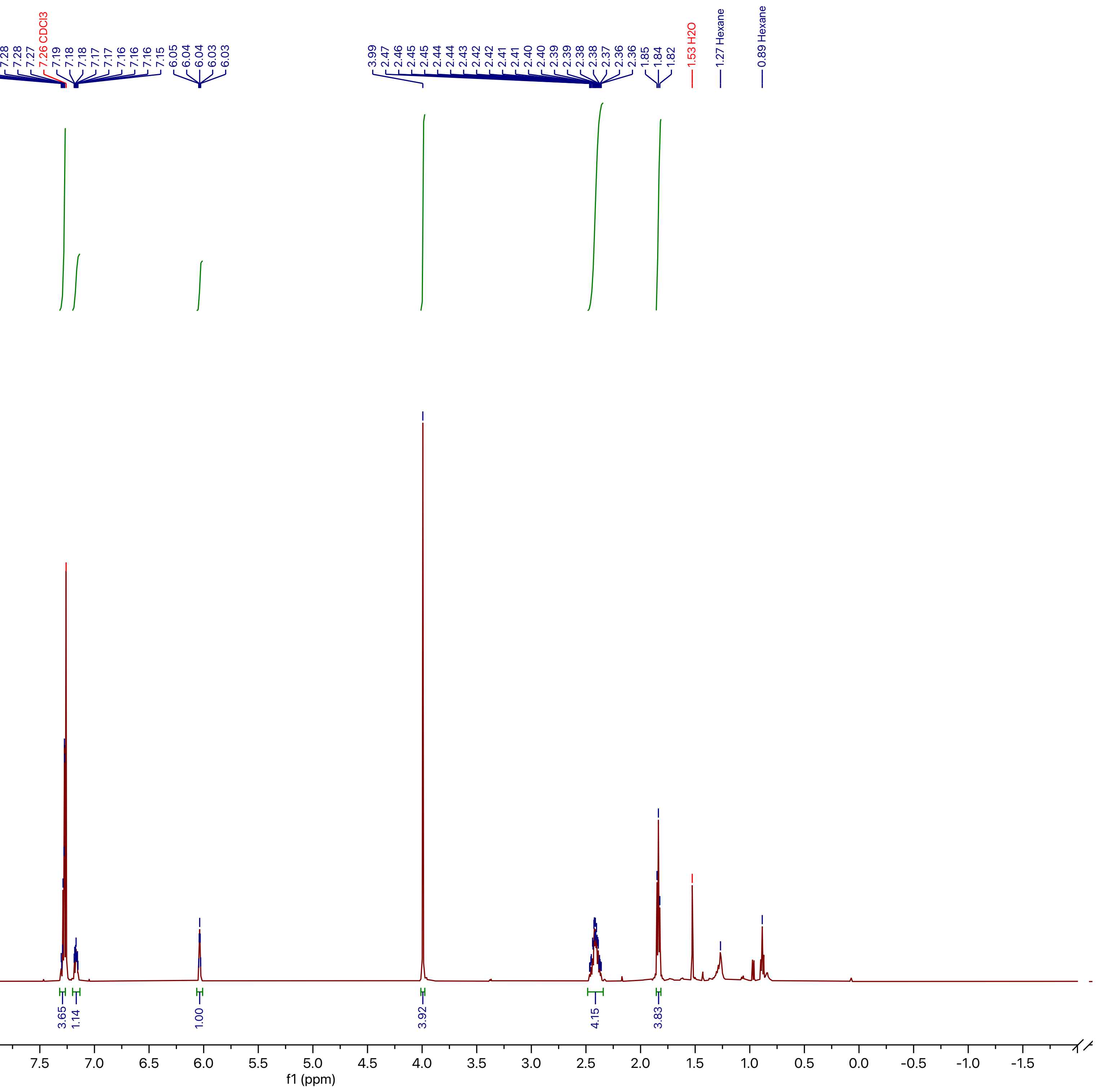

	Parameter	Value
1	Origin	Bruker BioSpin GmbH
2	Instrument	Avance
3	Solvent	CDCl3
4	Temperature	300.0
5	Pulse Sequence	zg30
6	Experiment	1D
7	Probe	Z151574_0073 (PI HR-BBO500S1-BBF/ H/ D-5.0-Z SP)
8	Number of Scans	16
9	Receiver Gain	101.0
10	Relaxation Delay	1.0000
11	Pulse Width	8.0000
12	Spectrometer Frequency	500.21
13	Spectral Width	10000.0
14	Lowest Frequency	-1922.8
15	Nucleus	1H
16	Acquired Size	32768
17	Spectral Size	65536

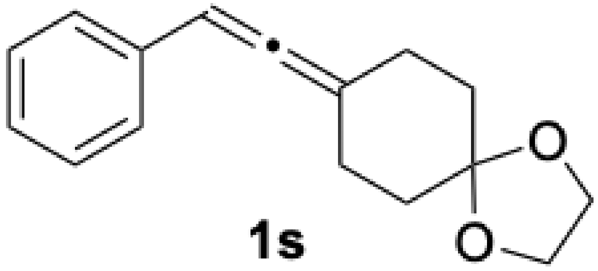

1s

	Parameter	Value
1	Origin	Bruker BioSpin GmbH
2	Instrument	Avance
3	Solvent	CDCl3
4	Temperature	300.0
5	Pulse Sequence	zgpg30
6	Experiment	1D
7	Probe	Z151574_0073 (PI HR-BBO500S1-BBF/ H/ D-5.0-Z SP)
8	Number of Scans	512
9	Receiver Gain	101.0
10	Relaxation Delay	2.0000
11	Pulse Width	9.0000
12	Spectrometer Frequency	125.79
13	Spectral Width	30120.5
14	Lowest Frequency	-2463.4
15	Nucleus	13C
16	Acquired Size	32768
17	Spectral Size	65536

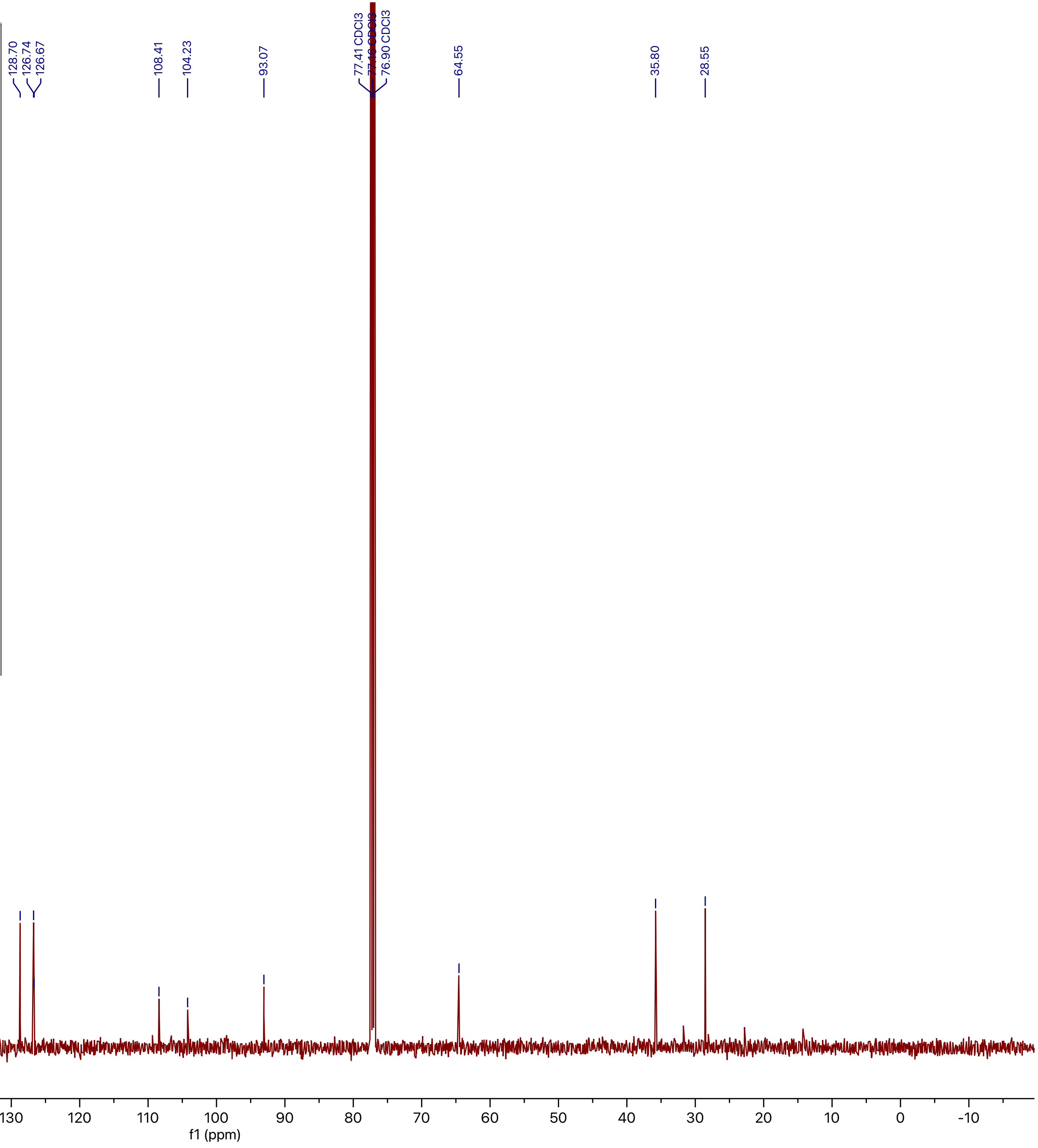

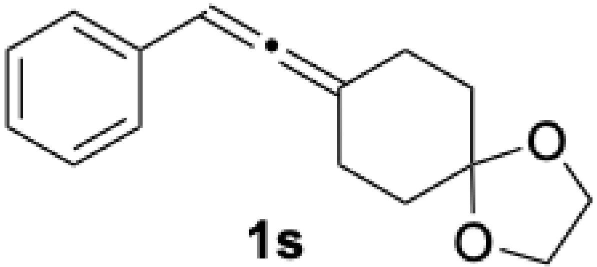

1s

	Parameter	Value
1	Origin	Varian
2	Solvent	cdcl3
3	Temperature	25.0
4	Pulse Sequence	s2pul
5	Experiment	1D
6	Probe	ASWPFG8319
7	Number of Scans	16
8	Receiver Gain	46
9	Relaxation Delay	5.0000
10	Pulse Width	7.7500
11	Spectrometer Frequency	399.73
12	Spectral Width	6410.3
13	Lowest Frequency	-808.0
14	Nucleus	1H
15	Acquired Size	16384
16	Spectral Size	65536
17	Digital Resolution	0.10

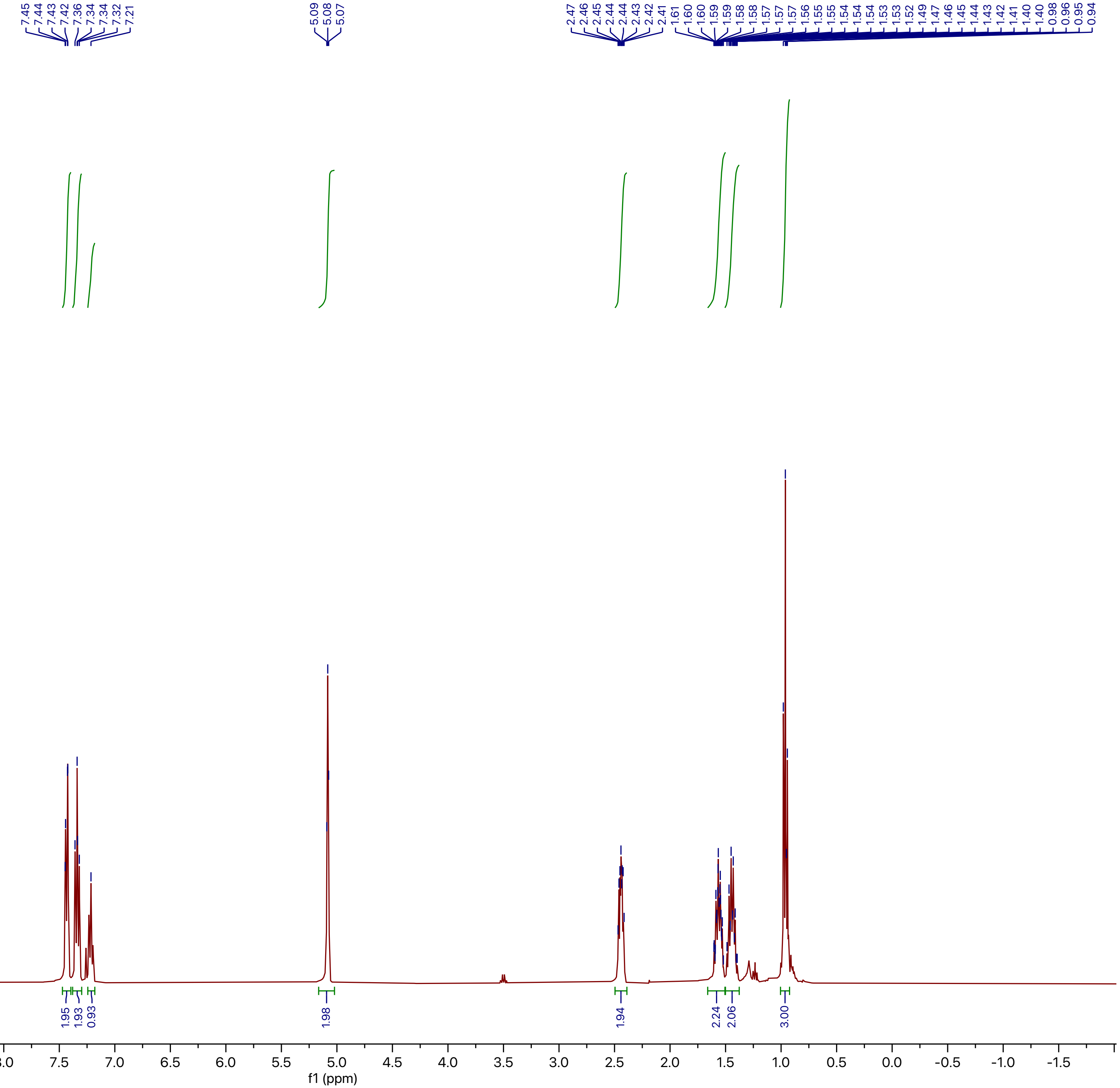

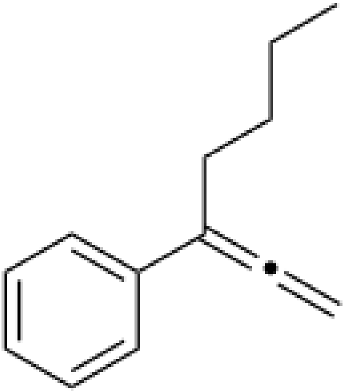

1t

Parameter		Value
1	Origin	Varian
2	Solvent	cdcl3
3	Temperature	25.0
4	Pulse Sequence	s2pul
5	Experiment	1D
6	Probe	ASWPFG8319
7	Number of Scans	512
8	Receiver Gain	30
9	Relaxation Delay	2.0000
10	Pulse Width	5.7500
11	Spectrometer Frequency	100.52
12	Spectral Width	25000.0
13	Lowest Frequency	-1431.1
14	Nucleus	13C
15	Acquired Size	32768
16	Spectral Size	65536
17	Digital Resolution	0.38

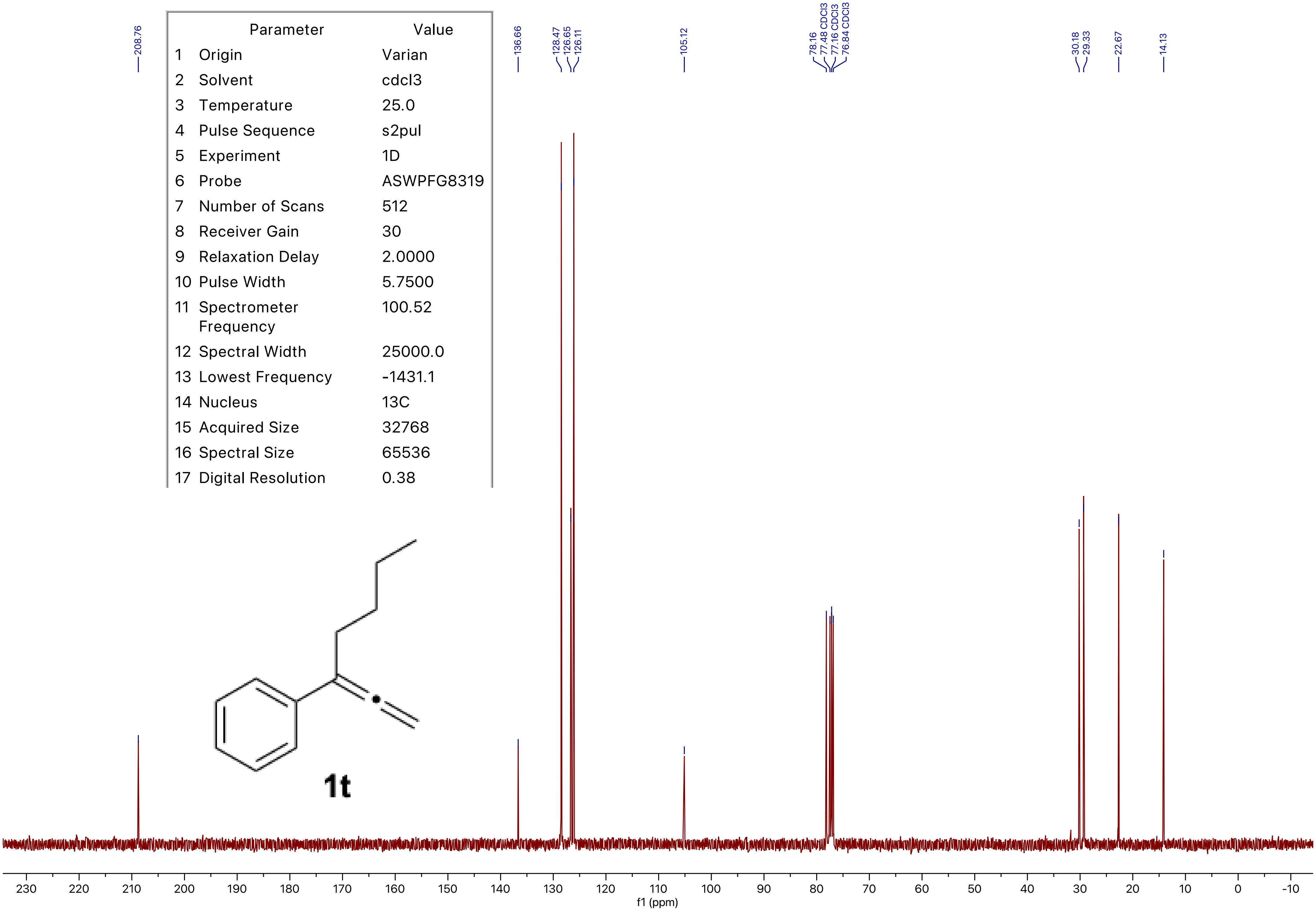

1t

Parameter		Value
1	Origin	Varian
2	Solvent	cdcl3
3	Temperature	25.0
4	Pulse Sequence	s2pul
5	Experiment	1D
6	Probe	ASWPFG8319
7	Number of Scans	16
8	Receiver Gain	50
9	Relaxation Delay	10.0000
10	Pulse Width	7.7500
11	Spectrometer Frequency	399.73
12	Spectral Width	6410.3
13	Lowest Frequency	-796.9
14	Nucleus	1H
15	Acquired Size	16384
16	Spectral Size	65536
17	Digital Resolution	0.10

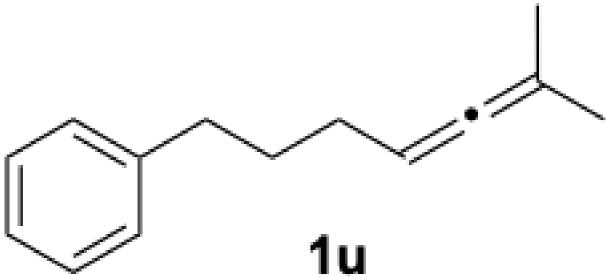

1u

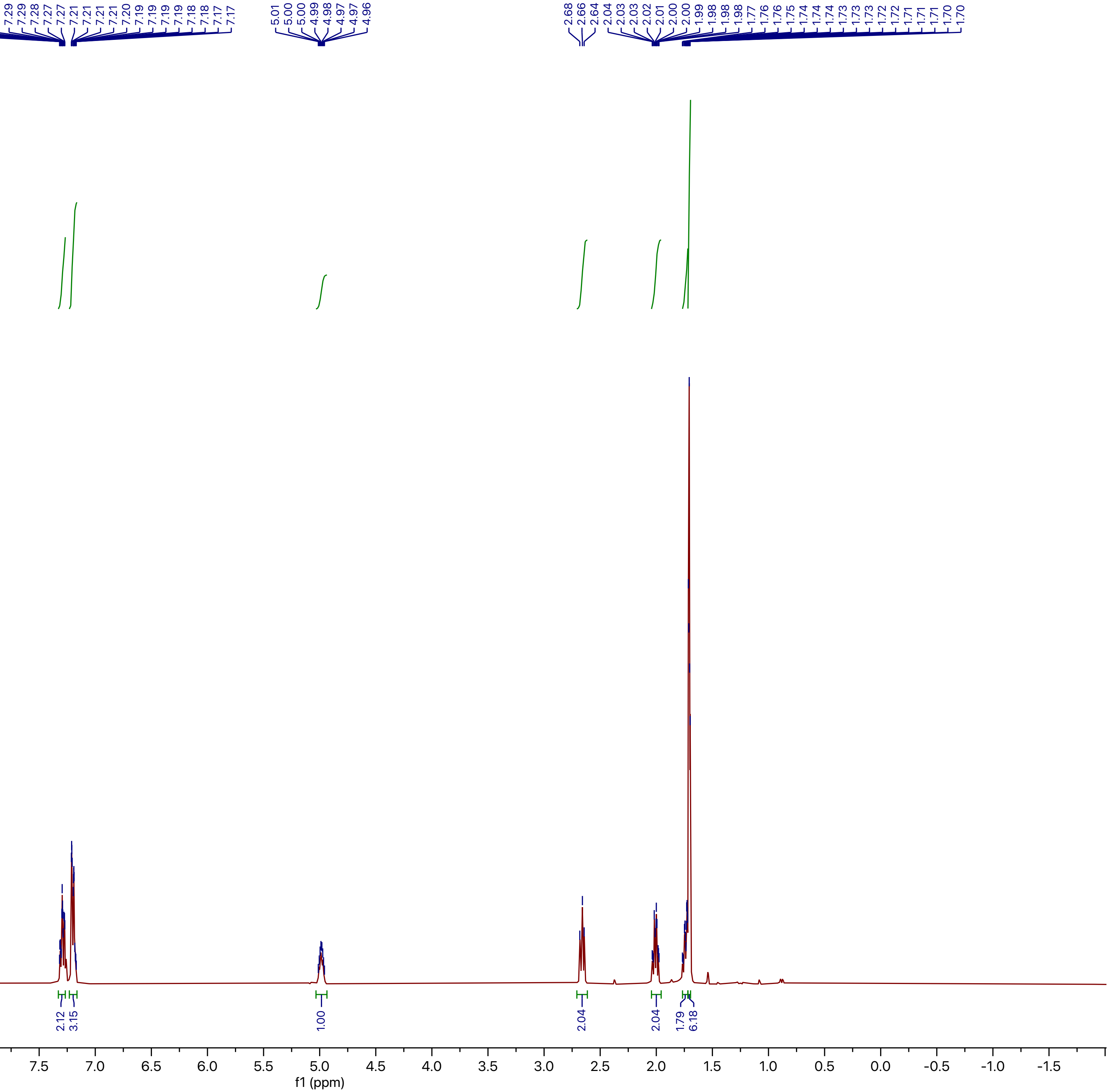

Parameter		Value
1	Origin	Varian
2	Solvent	cdcl3
3	Temperature	25.0
4	Pulse Sequence	s2pul
5	Experiment	1D
6	Probe	ASWPFG8319
7	Number of Scans	16
8	Receiver Gain	54
9	Relaxation Delay	10.0000
10	Pulse Width	7.7500
11	Spectrometer Frequency	399.73
12	Spectral Width	6410.3
13	Lowest Frequency	-805.8
14	Nucleus	1H
15	Acquired Size	16384
16	Spectral Size	65536
17	Digital Resolution	0.10

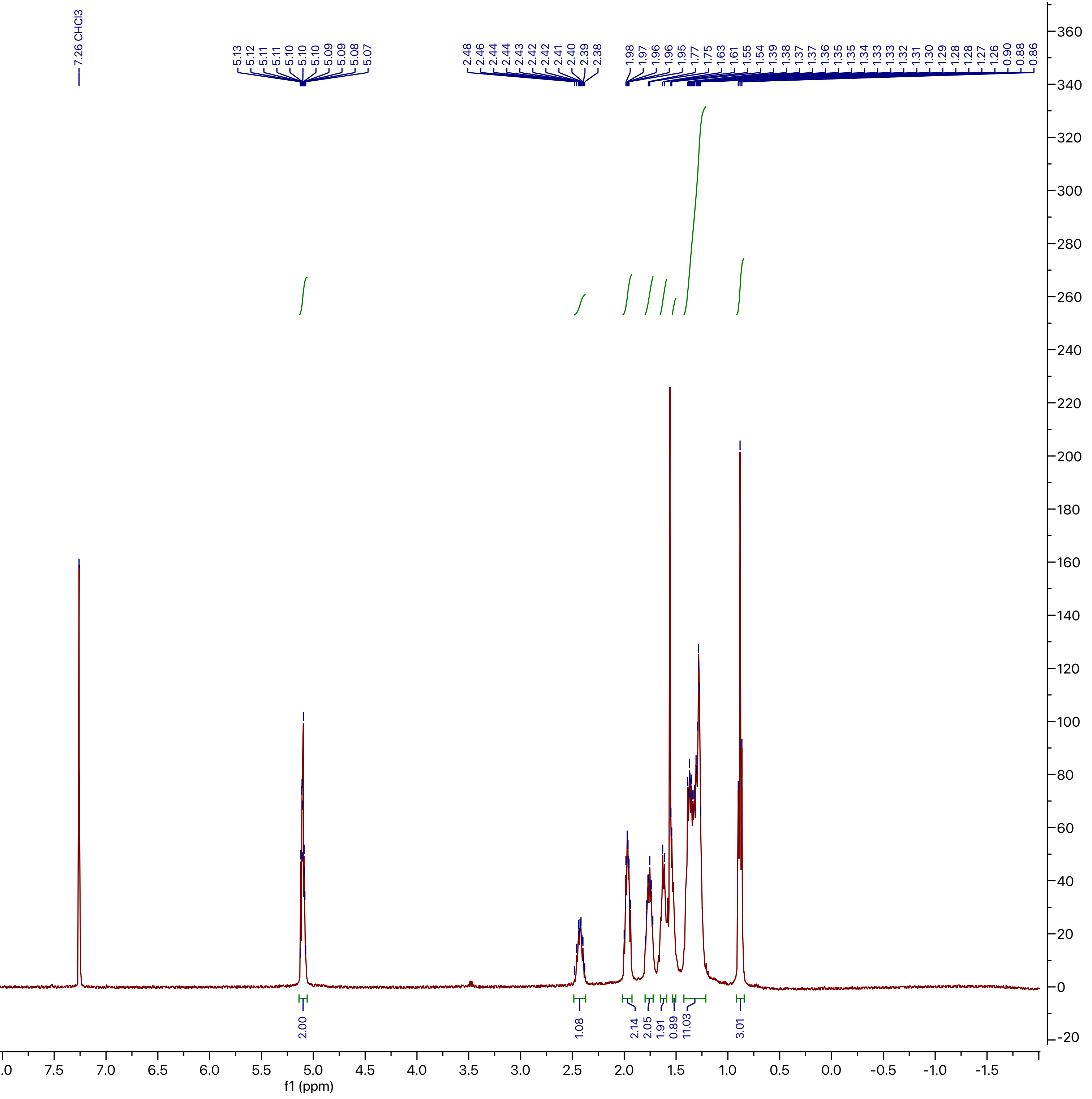

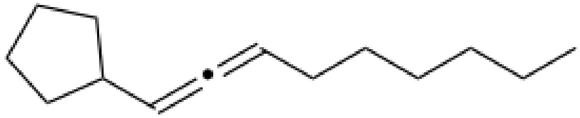

1v

	Parameter	Value
1	Origin	Varian
2	Solvent	cdcl3
3	Temperature	25.0
4	Pulse Sequence	s2pul
5	Experiment	1D
6	Probe	ASWPFG8319
7	Number of Scans	512
8	Receiver Gain	30
9	Relaxation Delay	2.0000
10	Pulse Width	5.7500
11	Spectrometer Frequency	100.52
12	Spectral Width	25000.0
13	Lowest Frequency	-1428.4
14	Nucleus	13C
15	Acquired Size	32768
16	Spectral Size	65536
17	Digital Resolution	0.38

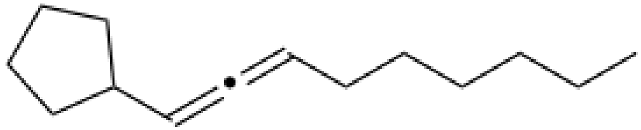

1v

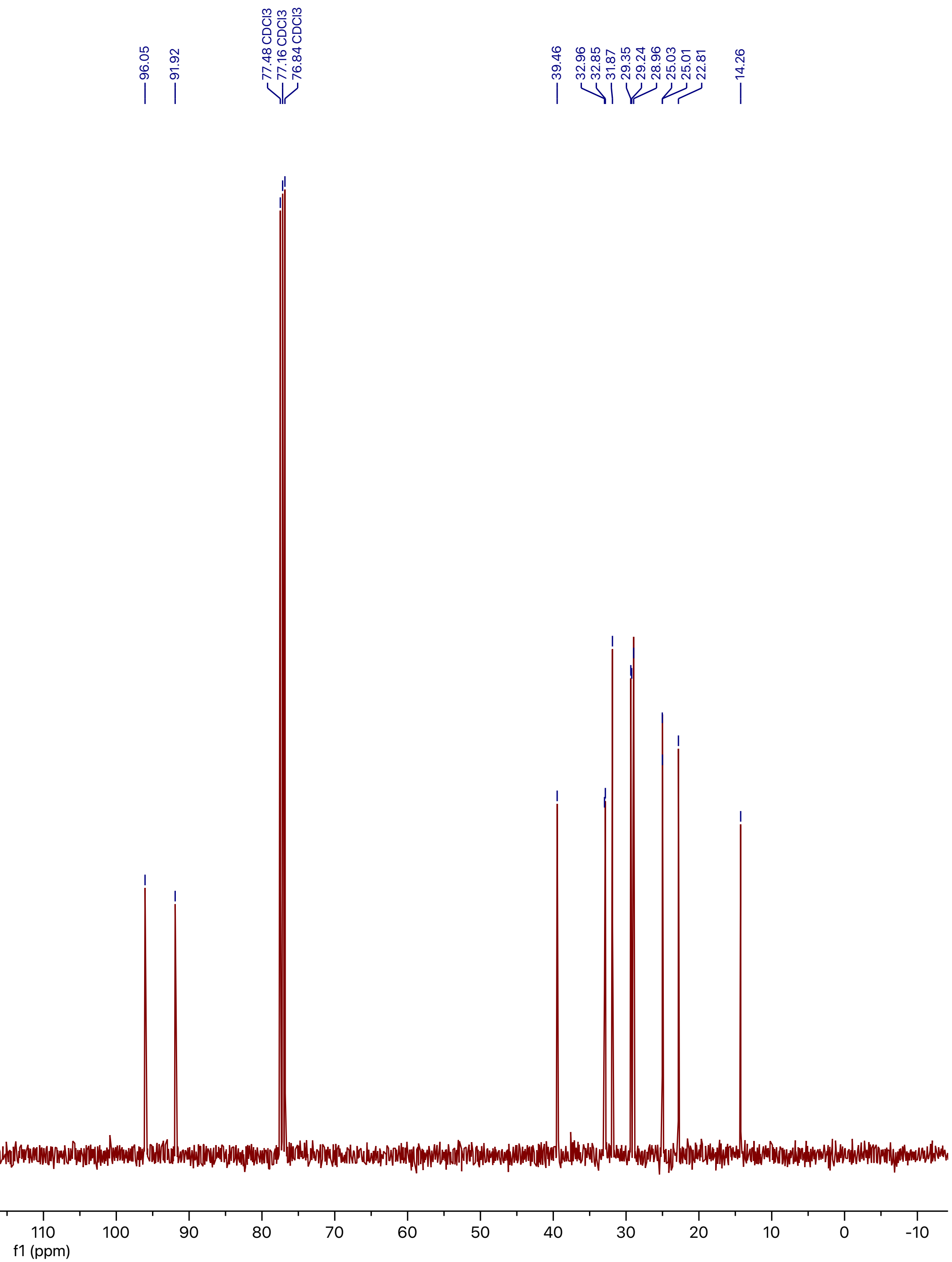

	Parameter	Value
1	Origin	Bruker BioSpin GmbH
2	Instrument	Avance
3	Solvent	CDCl3
4	Temperature	300.0
5	Pulse Sequence	zg30
6	Experiment	1D
7	Probe	Z151574_0073 (PI HR-BBO500S1-BBF/ H/ D-5.0-Z SP)
8	Number of Scans	16
9	Receiver Gain	101.0
10	Relaxation Delay	1.0000
11	Pulse Width	8.0000
12	Spectrometer Frequency	500.21
13	Spectral Width	10000.0
14	Lowest Frequency	-1923.0
15	Nucleus	1H
16	Acquired Size	32768
17	Spectral Size	65536

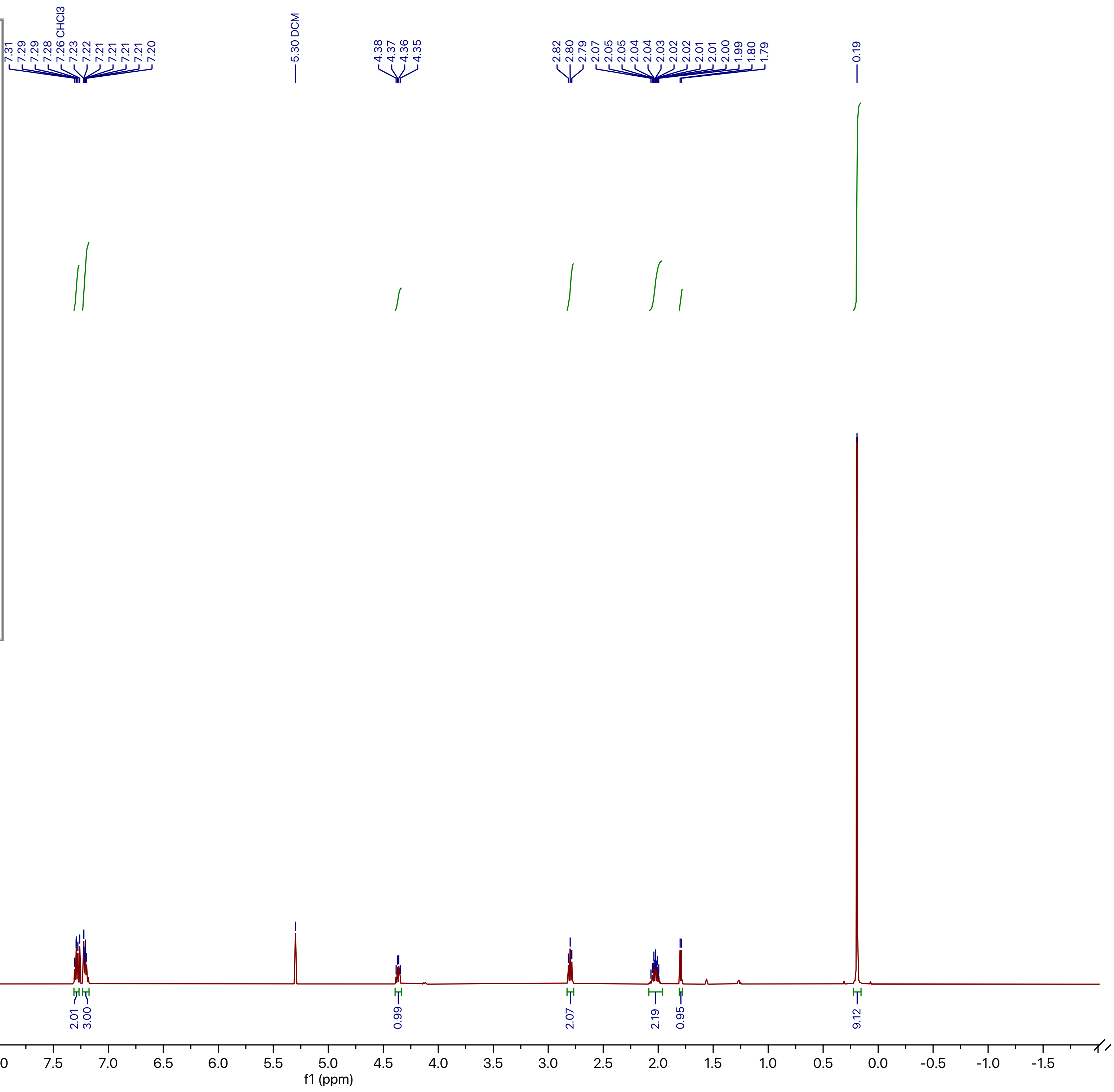

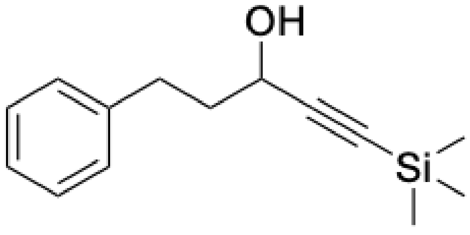

Parameter	Value
1 Origin	Varian
2 Solvent	cdcl3
3 Temperature	25.0
4 Pulse Sequence	s2pul
5 Experiment	1D
6 Probe	ASWPFG8319
7 Number of Scans	16
8 Receiver Gain	50
9 Relaxation Delay	5.0000
10 Pulse Width	7.7500
11 Spectrometer Frequency	399.73
12 Spectral Width	6410.3
13 Lowest Frequency	-806.7
14 Nucleus	1H
15 Acquired Size	16384
16 Spectral Size	65536
17 Digital Resolution	0.10

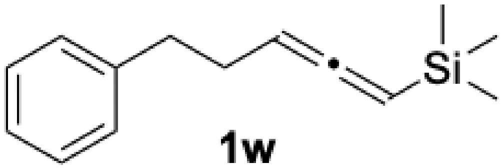

1w

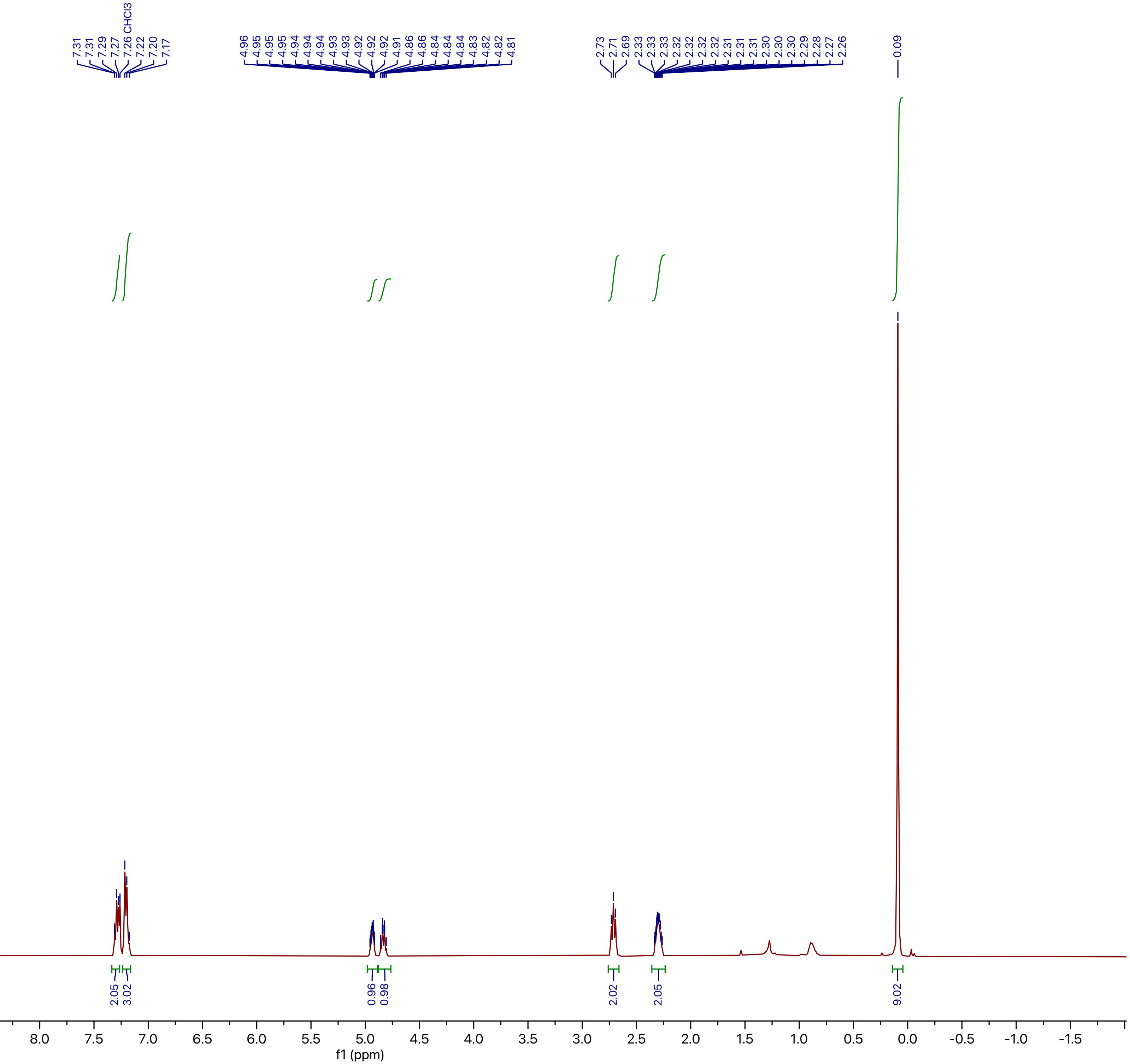

	Parameter	Value
1	Origin	Bruker BioSpin GmbH
2	Instrument	Avance
3	Solvent	CDCl3
4	Temperature	300.0
5	Pulse Sequence	zgpg30
6	Experiment	1D
7	Probe	Z151574_0073 (PI HR-BBO500S1-BBF/ H/ D-5.0-Z SP)
8	Number of Scans	1024
9	Receiver Gain	101.0
10	Relaxation Delay	2.0000
11	Pulse Width	9.0000
12	Spectrometer Frequency	125.79
13	Spectral Width	30120.5
14	Lowest Frequency	-2463.9
15	Nucleus	13C
16	Acquired Size	32768
17	Spectral Size	65536

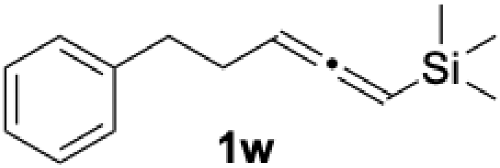

1w

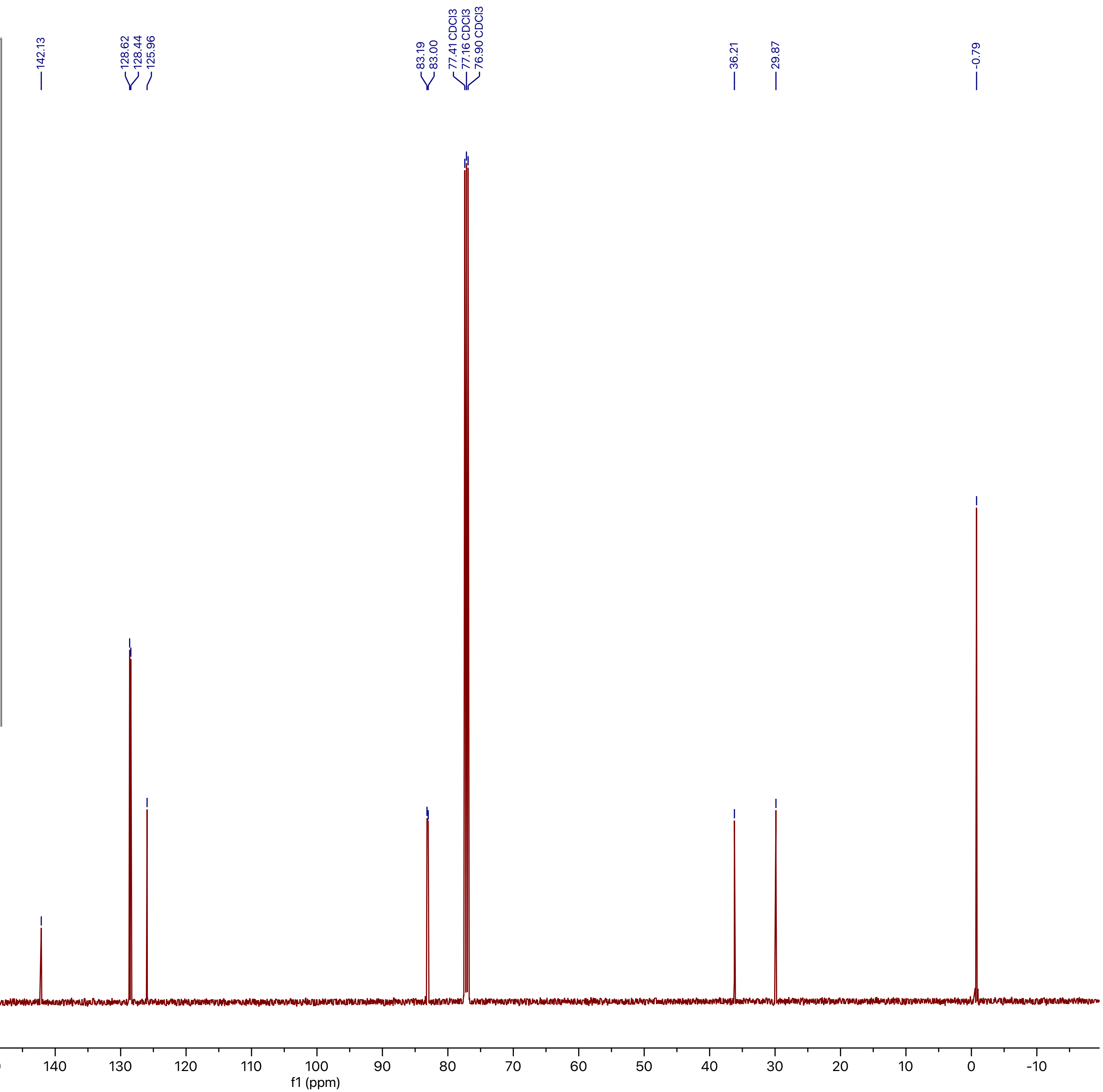

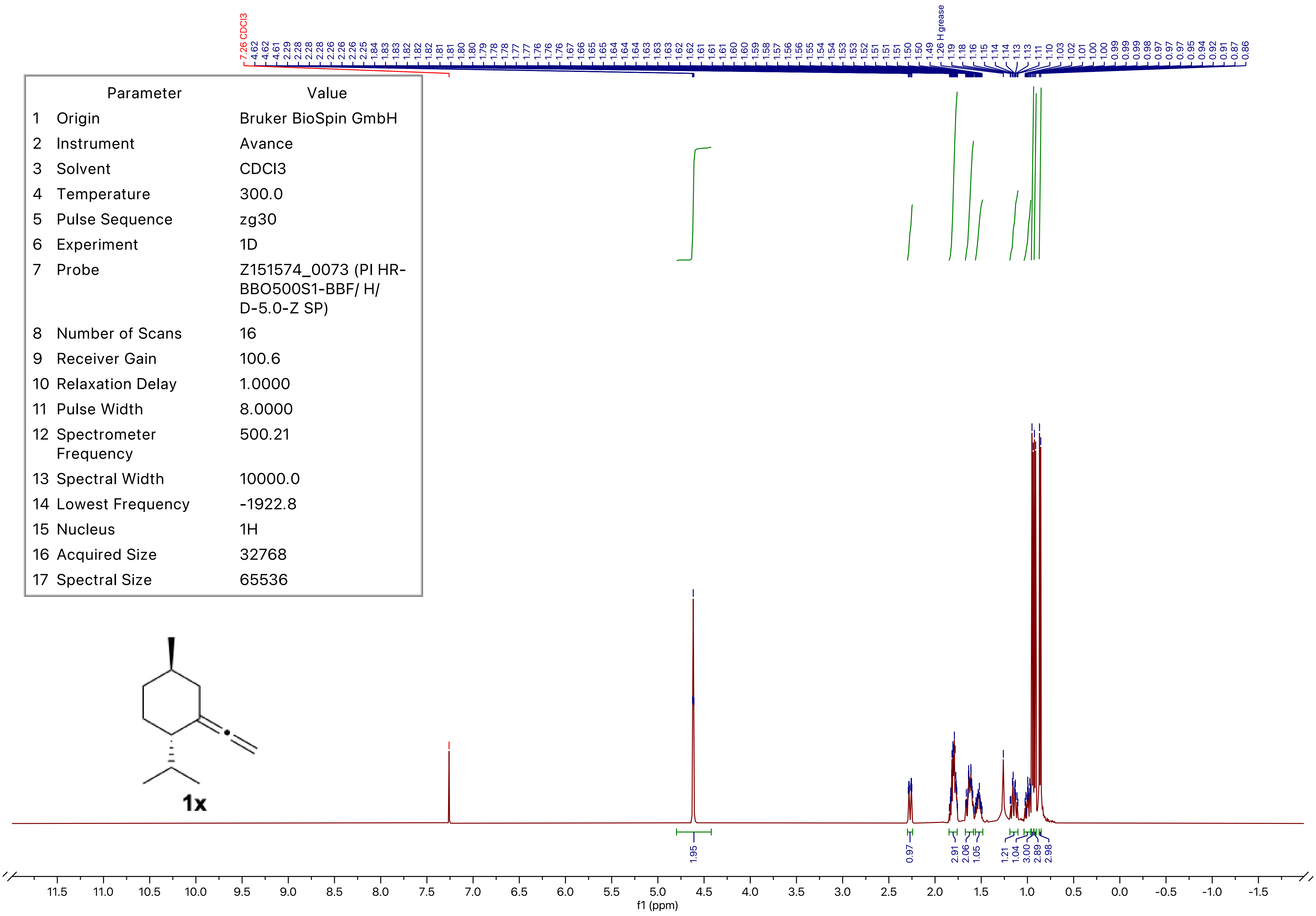

	Parameter	Value
1	Origin	Bruker BioSpin GmbH
2	Instrument	Avance
3	Solvent	CDCl3
4	Temperature	300.0
5	Pulse Sequence	zg30
6	Experiment	1D
7	Probe	Z151574_0073 (PI HR-BBO500S1-BBF/ H/ D-5.0-Z SP)
8	Number of Scans	16
9	Receiver Gain	100.6
10	Relaxation Delay	1.0000
11	Pulse Width	8.0000
12	Spectrometer Frequency	500.21
13	Spectral Width	10000.0
14	Lowest Frequency	-1922.8
15	Nucleus	1H
16	Acquired Size	32768
17	Spectral Size	65536

1x

	Parameter	Value
1	Origin	Bruker BioSpin GmbH
2	Instrument	Avance
3	Solvent	DMSO
4	Temperature	300.0
5	Pulse Sequence	zg30
6	Experiment	1D
7	Probe	Z151574_0073 (PI HR-BBO500S1-BBF/ H/ D-5.0-Z SP)
8	Number of Scans	64
9	Receiver Gain	101.0
10	Relaxation Delay	10.0000
11	Pulse Width	8.0000
12	Spectrometer Frequency	500.21
13	Spectral Width	10000.0
14	Lowest Frequency	-1917.7
15	Nucleus	1H
16	Acquired Size	32768
17	Spectral Size	65536

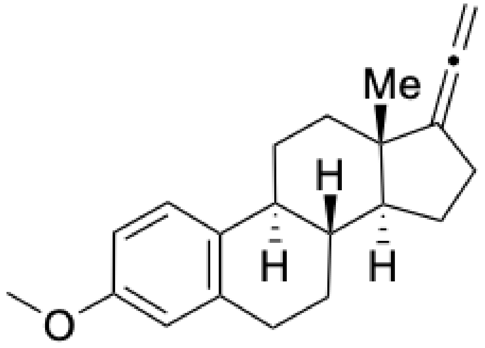

1y

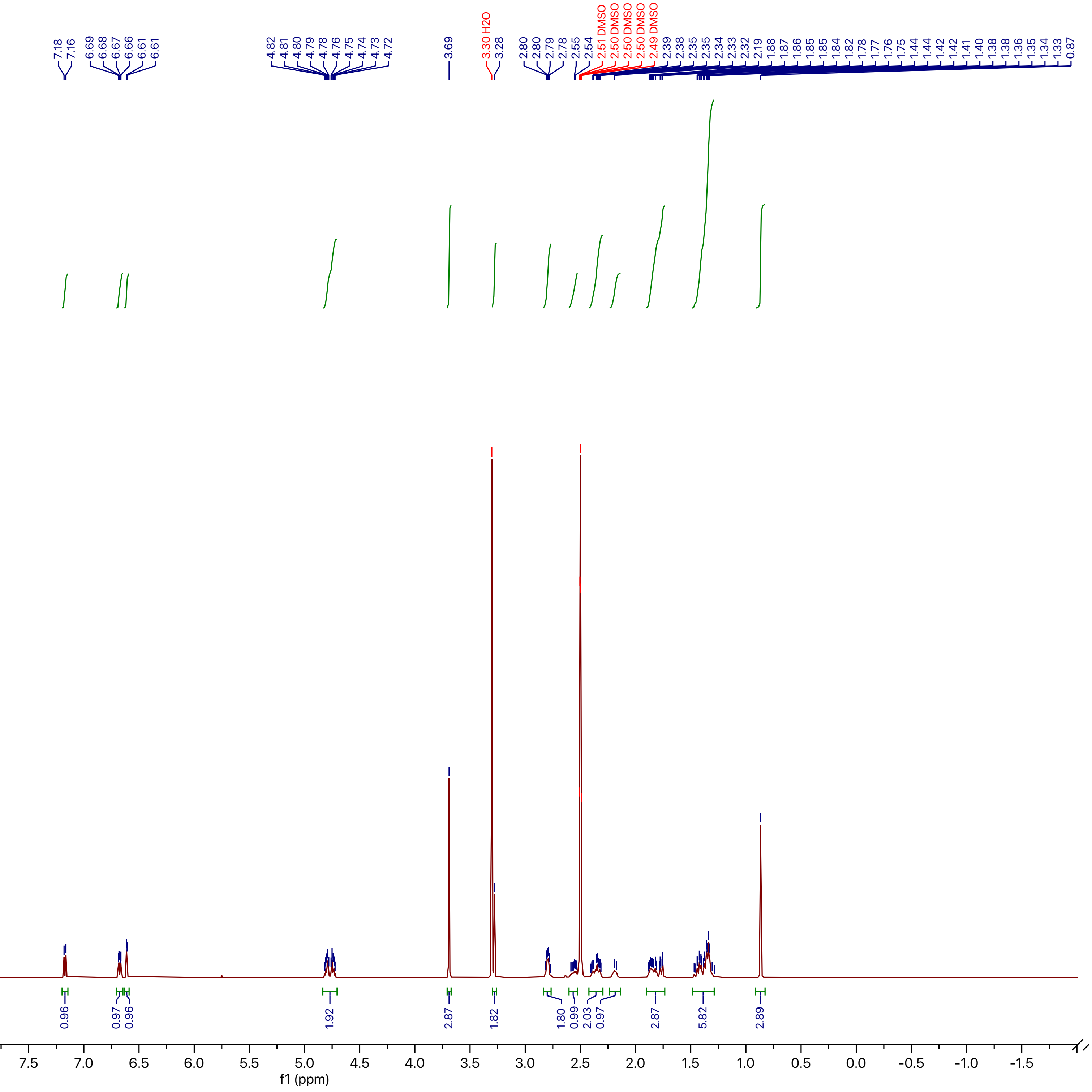

	Parameter	Value
1	Origin	Bruker BioSpin GmbH
2	Instrument	Avance
3	Solvent	CDCl3
4	Temperature	298.0
5	Pulse Sequence	zg30
6	Experiment	1D
7	Probe	Z163739_0400 (PI HR-BBO400S1-BBF/ H/ D-5.0-Z SP)
8	Number of Scans	16
9	Receiver Gain	101.0
10	Relaxation Delay	1.0000
11	Pulse Width	8.0000
12	Spectrometer Frequency	400.13
13	Spectral Width	8196.7
14	Lowest Frequency	-1637.3
15	Nucleus	1H
16	Acquired Size	32768
17	Spectral Size	65536

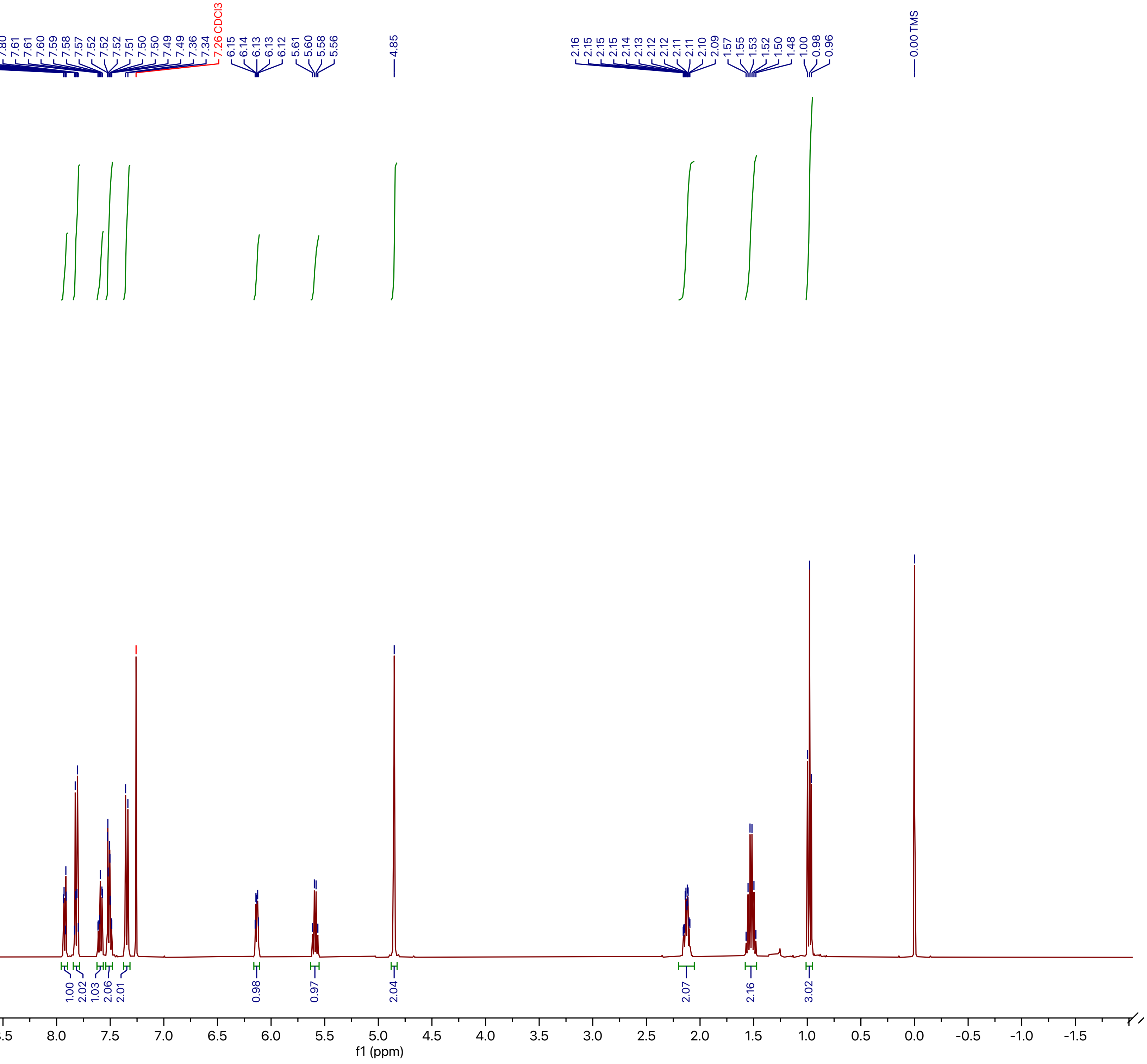

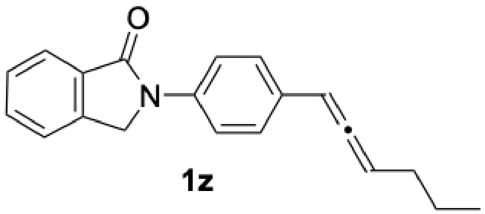

1z

	Parameter	Value
1	Origin	Bruker BioSpin GmbH
2	Instrument	Avance
3	Solvent	CDCl3
4	Temperature	298.0
5	Pulse Sequence	zgpg30
6	Experiment	1D
7	Probe	Z163739_0400 (PI HR-BBO400S1-BBF/ H/ D-5.0-Z SP)
8	Number of Scans	1024
9	Receiver Gain	101.0
10	Relaxation Delay	2.0000
11	Pulse Width	8.0000
12	Spectrometer Frequency	100.62
13	Spectral Width	23809.5
14	Lowest Frequency	-1843.5
15	Nucleus	13C
16	Acquired Size	32768
17	Spectral Size	65536

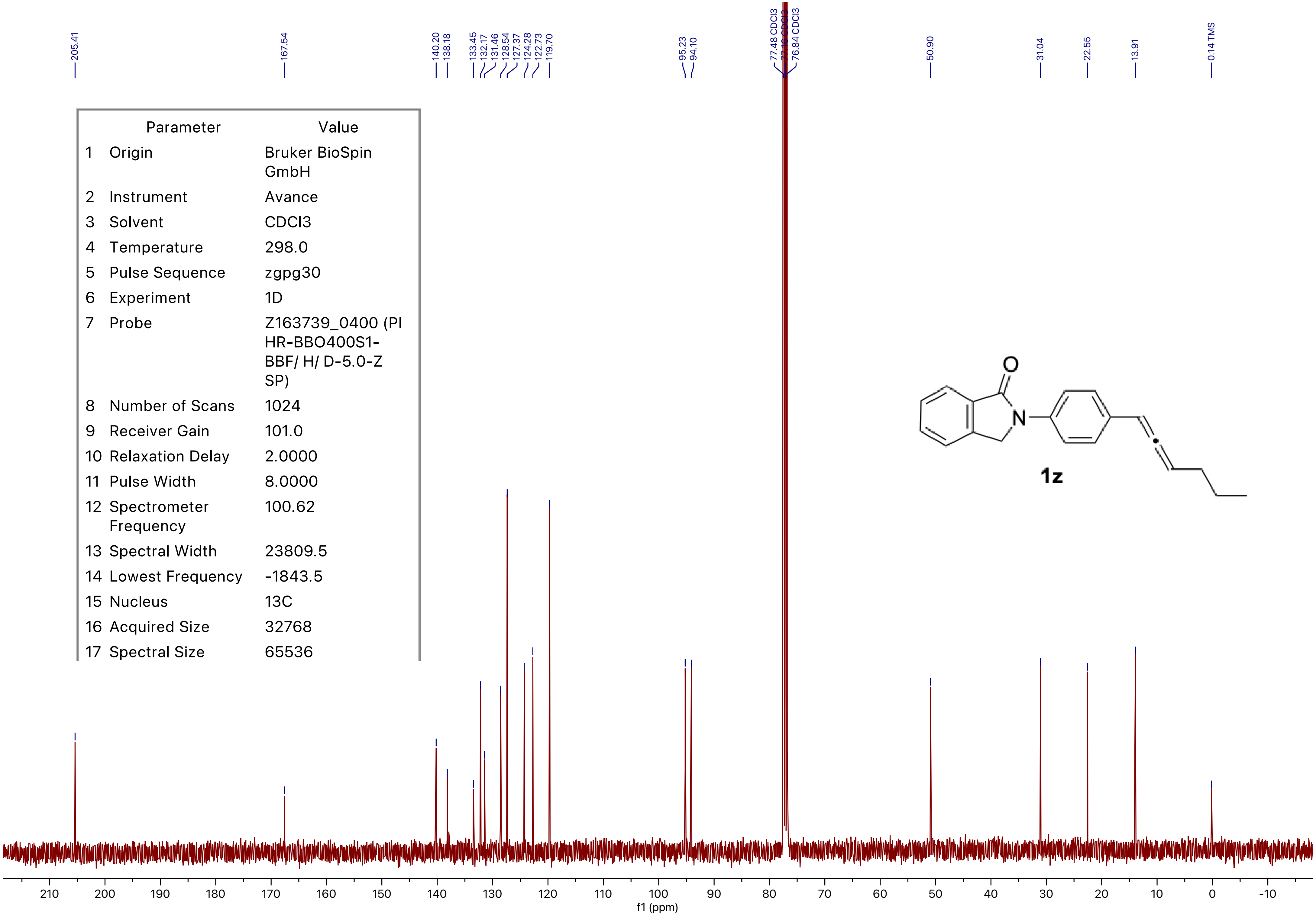

1z

	Parameter	Value
1	Origin	Varian
2	Solvent	cdcl3
3	Temperature	25.0
4	Pulse Sequence	s2pul
5	Experiment	1D
6	Probe	OneNMR_W036
7	Number of Scans	16
8	Receiver Gain	28
9	Relaxation Delay	10.0000
10	Pulse Width	4.8000
11	Spectrometer Frequency	499.73
12	Spectral Width	8012.8
13	Lowest Frequency	-1003.5
14	Nucleus	1H
15	Acquired Size	16384
16	Spectral Size	65536
17	Digital Resolution	0.12

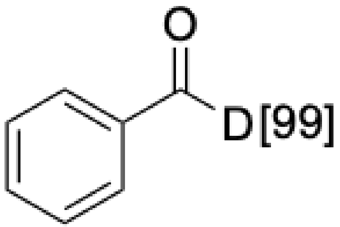

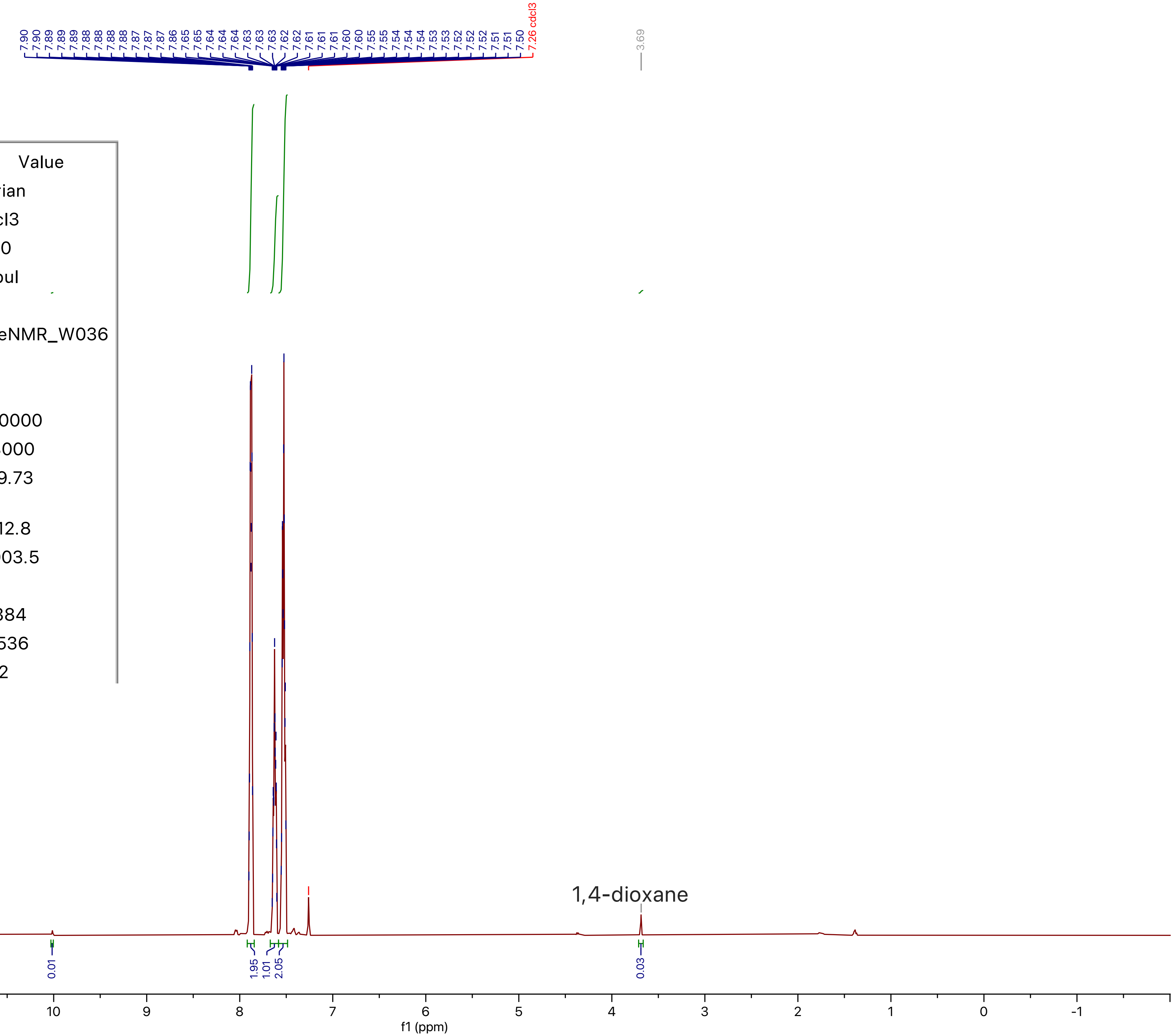

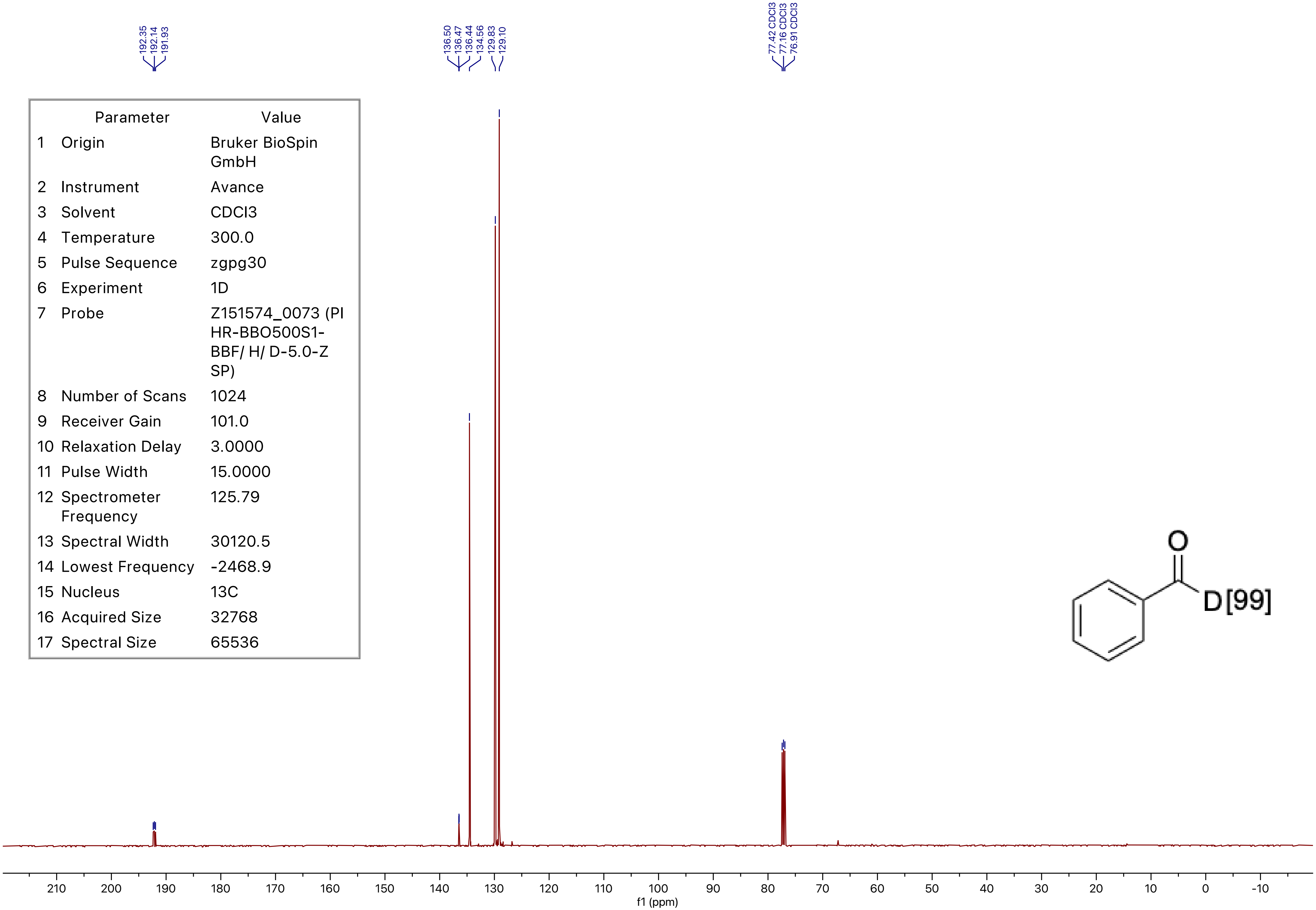

	Parameter	Value
1	Origin	Bruker BioSpin GmbH
2	Instrument	Avance
3	Solvent	CDCl3
4	Temperature	300.0
5	Pulse Sequence	zgpg30
6	Experiment	1D
7	Probe	Z151574_0073 (PI HR-BBO500S1-BBF/ H/ D-5.0-Z SP)
8	Number of Scans	1024
9	Receiver Gain	101.0
10	Relaxation Delay	3.0000
11	Pulse Width	15.0000
12	Spectrometer Frequency	125.79
13	Spectral Width	30120.5
14	Lowest Frequency	-2468.9
15	Nucleus	13C
16	Acquired Size	32768
17	Spectral Size	65536

Parameter		Value
1	Origin	Bruker BioSpin GmbH
2	Instrument	Avance
3	Solvent	CDCl3
4	Temperature	300.0
5	Pulse Sequence	zg30
6	Experiment	1D
7	Probe	Z151574_0073 (PI HR-BBO500S1-BBF/ H/ D-5.0-Z SP)
8	Number of Scans	16
9	Receiver Gain	101.0
10	Relaxation Delay	1.0000
11	Pulse Width	8.0000
12	Spectrometer Frequency	500.21
13	Spectral Width	10000.0
14	Lowest Frequency	-1923.4
15	Nucleus	1H
16	Acquired Size	32768
17	Spectral Size	65536

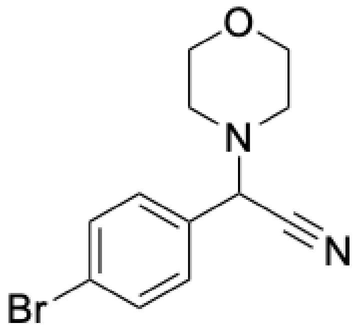

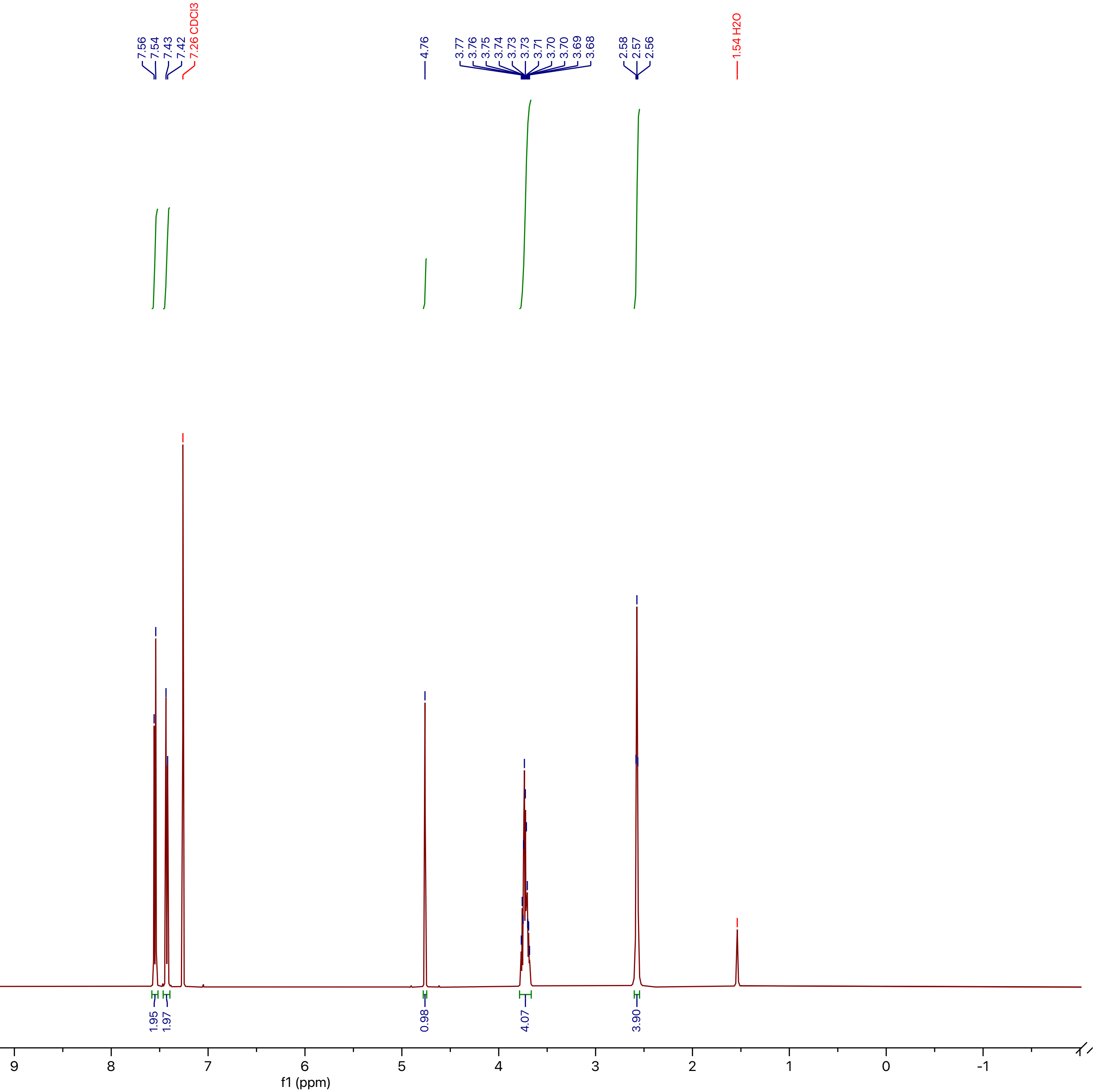

	Parameter	Value
1	Origin	Bruker BioSpin GmbH
2	Instrument	Avance
3	Solvent	CDCl3
4	Temperature	300.0
5	Pulse Sequence	zgpg30
6	Experiment	1D
7	Probe	Z151574_0073 (PI HR-BBO500S1-BBF/ H/ D-5.0-Z SP)
8	Number of Scans	1024
9	Receiver Gain	101.0
10	Relaxation Delay	2.0000
11	Pulse Width	9.0000
12	Spectrometer Frequency	125.79
13	Spectral Width	30120.5
14	Lowest Frequency	-2463.9
15	Nucleus	13C
16	Acquired Size	32768
17	Spectral Size	65536

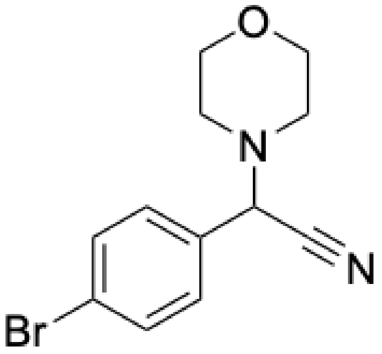

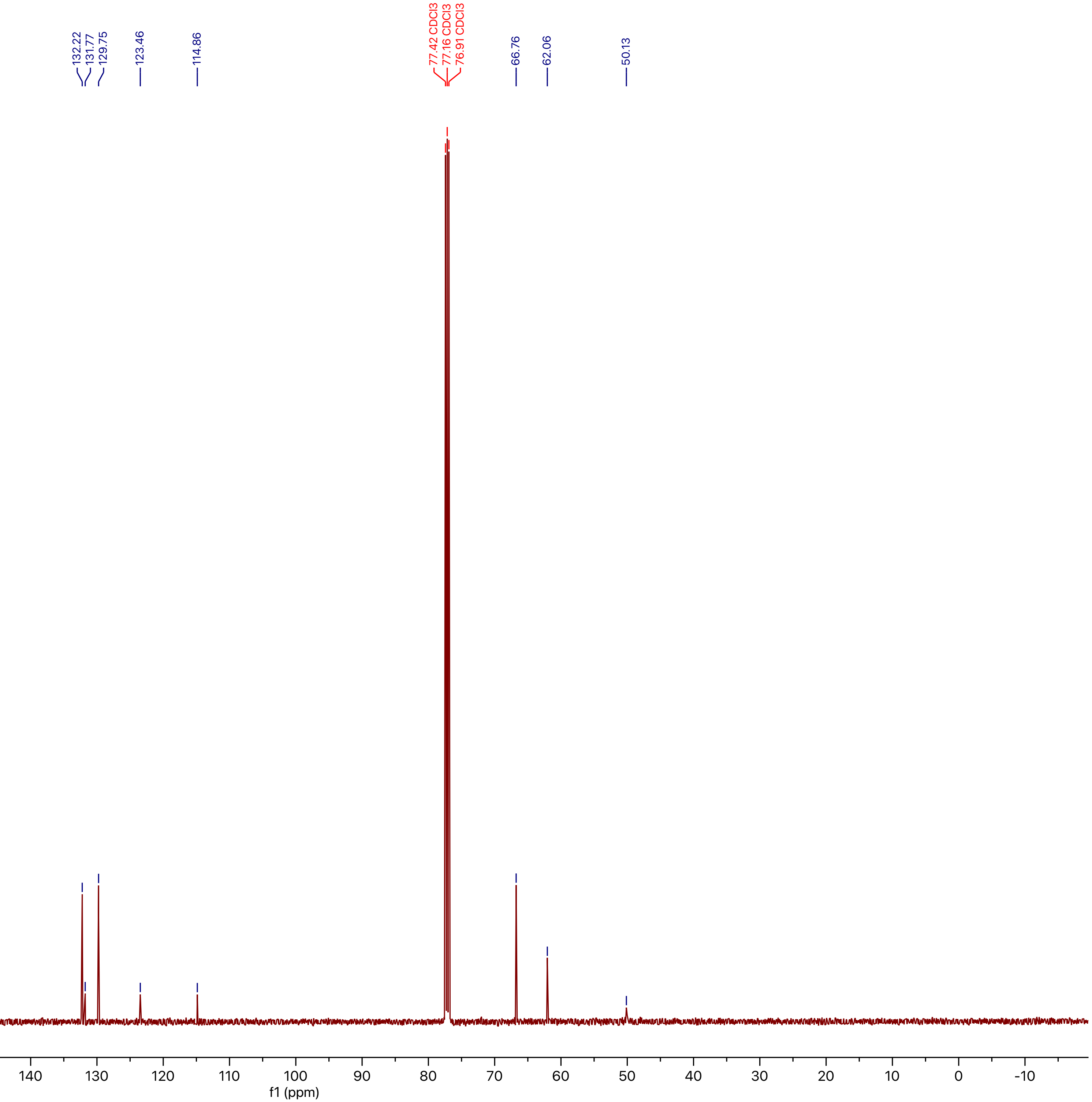

	Parameter	Value
	Origin	Bruker BioSpin GmbH
	Instrument	Avance
	Solvent	CDCl3
	Temperature	300.0
	Pulse Sequence	zg30
	Experiment	1D
	Probe	Z151574_0073 (PI HR-BBO500S1-BBF/ H/ D-5.0-Z SP)
	Number of Scans	16
	Receiver Gain	101.0
0	Relaxation Delay	1.0000
1	Pulse Width	8.0000
2	Spectrometer Frequency	500.21
3	Spectral Width	10000.0
4	Lowest Frequency	-1923.4
5	Nucleus	1H
6	Acquired Size	32768
7	Spectral Size	65536

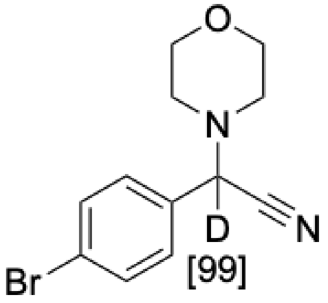

crude ^{1}HNMR

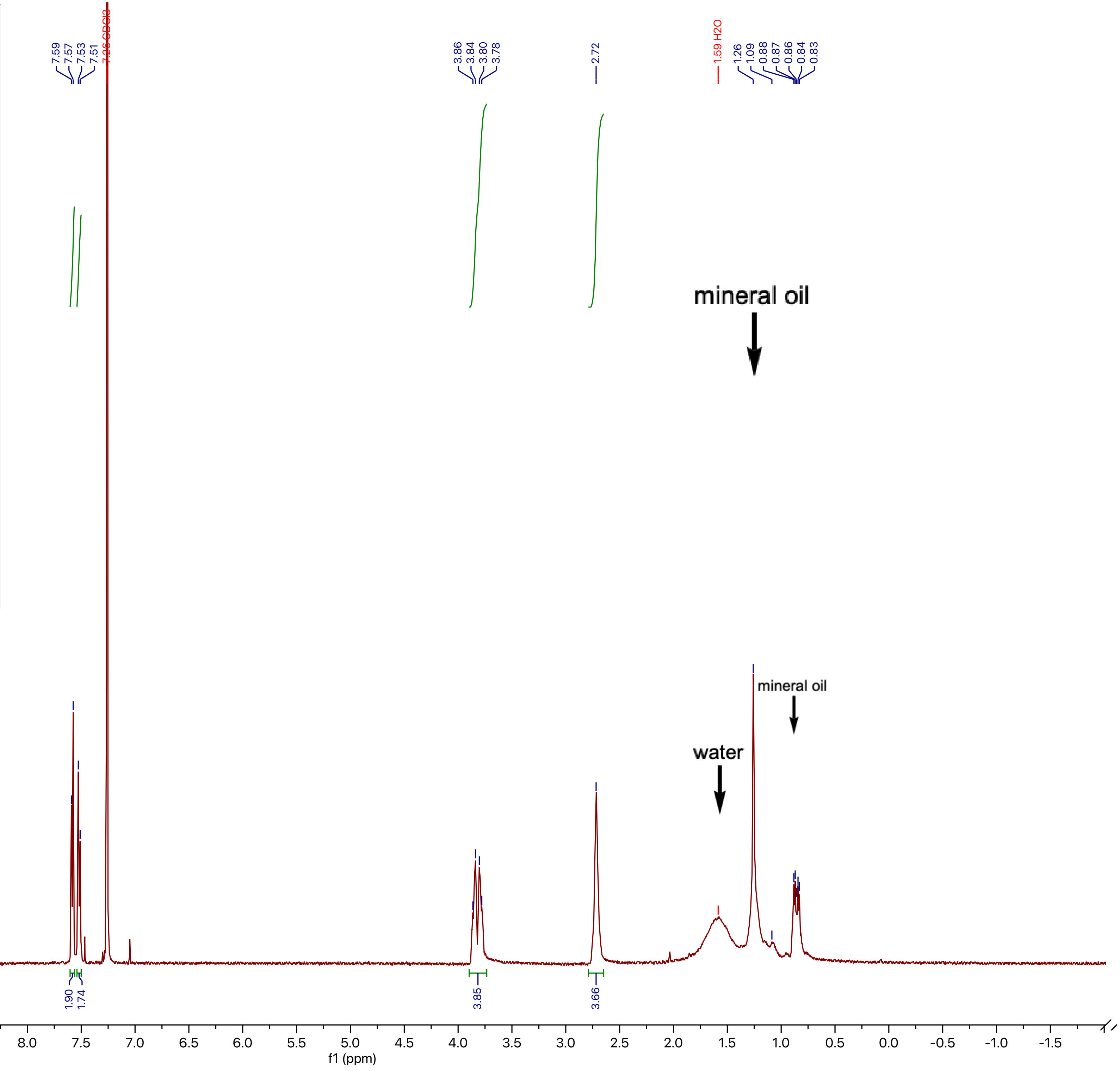

	Parameter	Value
1	Origin	Bruker BioSpin GmbH
2	Instrument	Avance
3	Solvent	CDCl3
4	Temperature	300.0
5	Pulse Sequence	zg30
6	Experiment	1D
7	Probe	Z151574_0073 (PI HR-BBO500S1-BBF/ H/ D-5.0-Z SP)
8	Number of Scans	64
9	Receiver Gain	101.0
10	Relaxation Delay	5.0000
11	Pulse Width	8.0000
12	Spectrometer Frequency	500.21
13	Spectral Width	10000.0
14	Lowest Frequency	-1923.2
15	Nucleus	1H
16	Acquired Size	32768
17	Spectral Size	65536

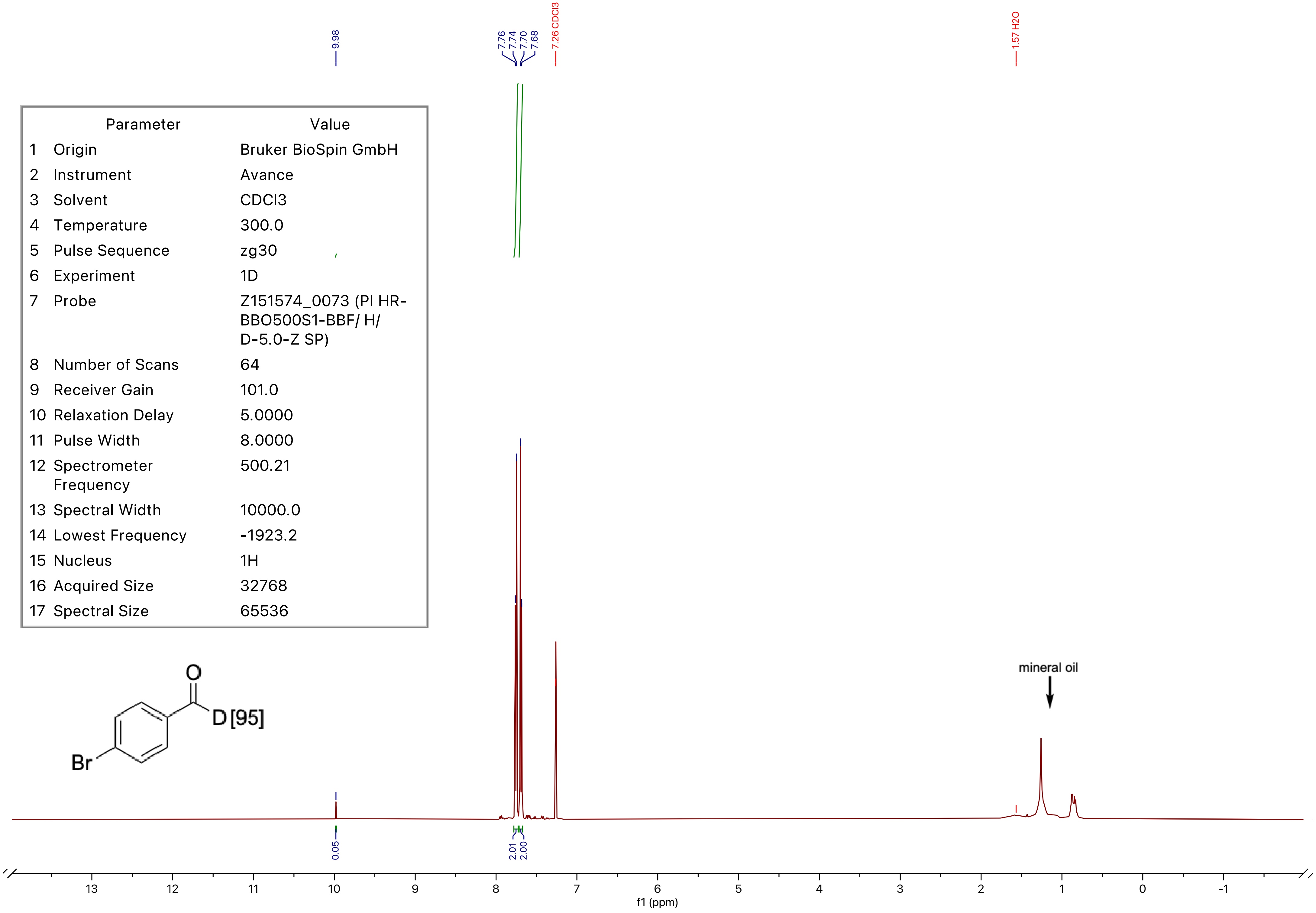

	Parameter	Value
1	Origin	Bruker BioSpin GmbH
2	Instrument	Avance
3	Solvent	CDCl3
4	Temperature	300.0
5	Pulse Sequence	zgpg30
6	Experiment	1D
7	Probe	Z151574_0073 (PI HR-BBO500S1-BBF/ H/ D-5.0-Z SP)
8	Number of Scans	1024
9	Receiver Gain	101.0
10	Relaxation Delay	2.0000
11	Pulse Width	9.0000
12	Spectrometer Frequency	125.79
13	Spectral Width	30120.5
14	Lowest Frequency	-2464.3
15	Nucleus	13C
16	Acquired Size	32768
17	Spectral Size	65536

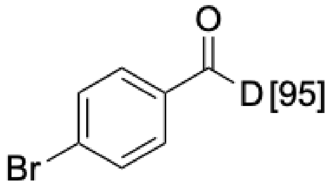

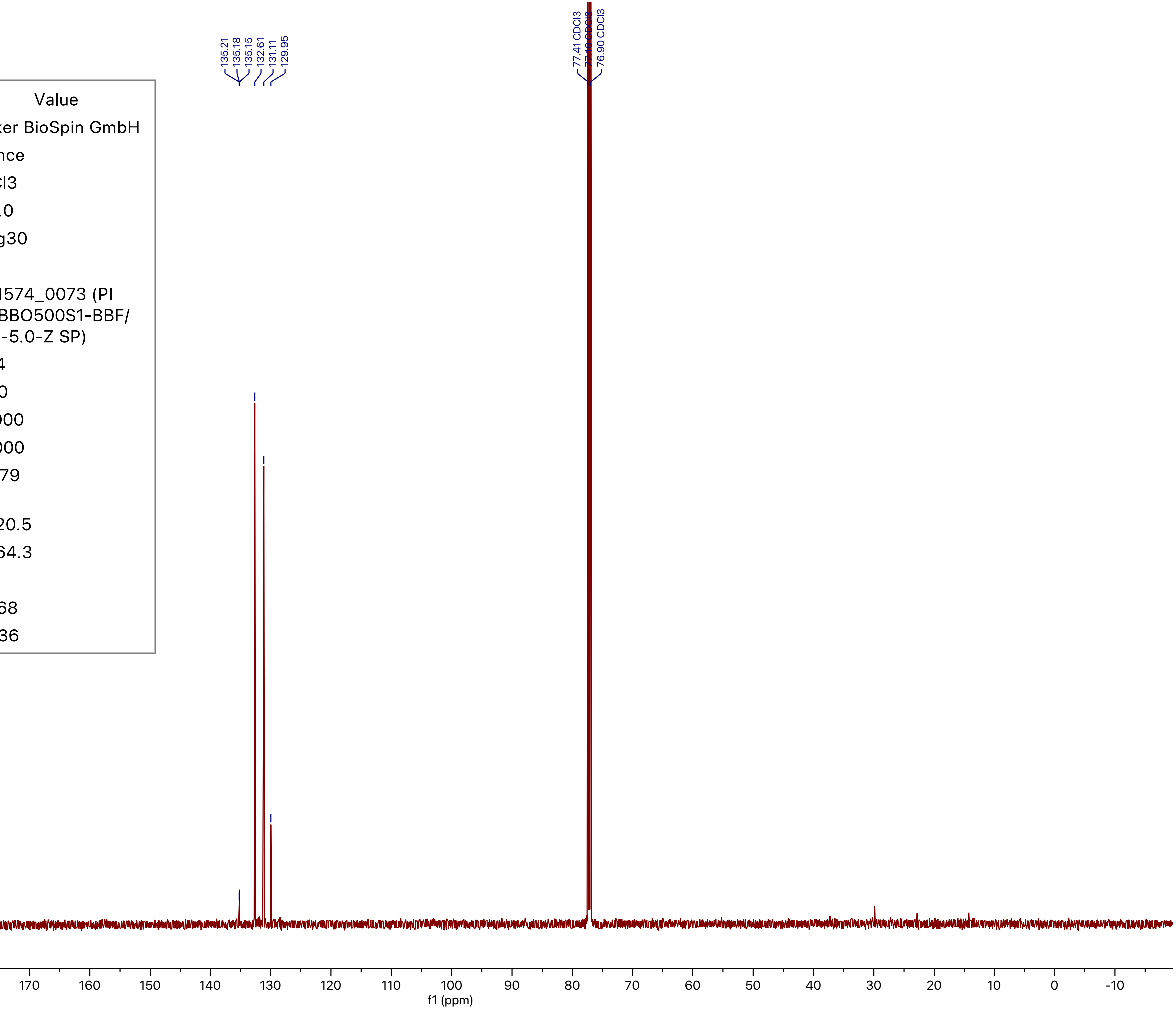

	Parameter	Value
1	Origin	Bruker BioSpin GmbH
2	Instrument	Avance
3	Solvent	CDCl3
4	Temperature	300.0
5	Pulse Sequence	zg30
6	Experiment	1D
7	Probe	Z151574_0073 (PI HR-BBO500S1-BBF/ H/ D-5.0-Z SP)
8	Number of Scans	16
9	Receiver Gain	101.0
10	Relaxation Delay	1.0000
11	Pulse Width	8.0000
12	Spectrometer Frequency	500.21
13	Spectral Width	10000.0
14	Lowest Frequency	-1923.3
15	Nucleus	1H
16	Acquired Size	32768
17	Spectral Size	65536

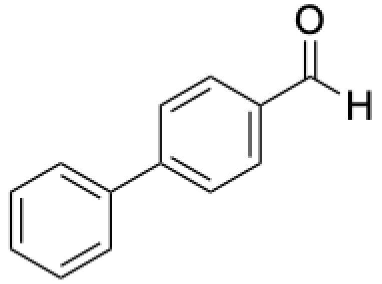

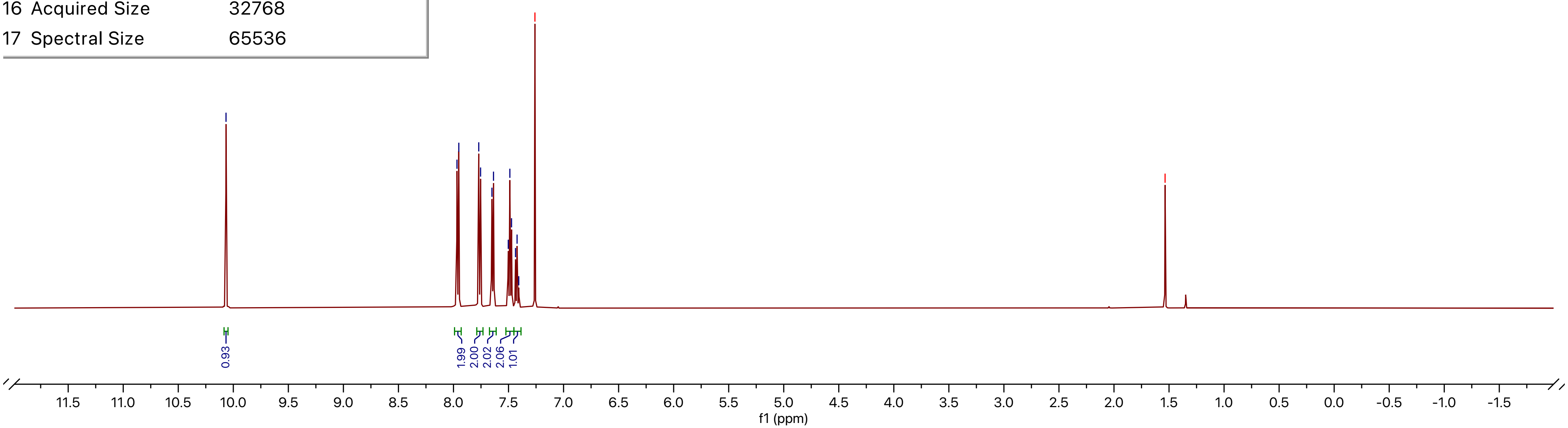

	Parameter	Value
1	Origin	Bruker BioSpin GmbH
2	Instrument	Avance
3	Solvent	CDCl3
4	Temperature	300.0
5	Pulse Sequence	zg30
6	Experiment	1D
7	Probe	Z151574_0073 (PI HR-BBO500S1-BBF/ H/ D-5.0-Z SP)
8	Number of Scans	16
9	Receiver Gain	101.0
10	Relaxation Delay	1.0000
11	Pulse Width	8.0000
12	Spectrometer Frequency	500.21
13	Spectral Width	10000.0
14	Lowest Frequency	-1923.5
15	Nucleus	1H
16	Acquired Size	32768
17	Spectral Size	65536

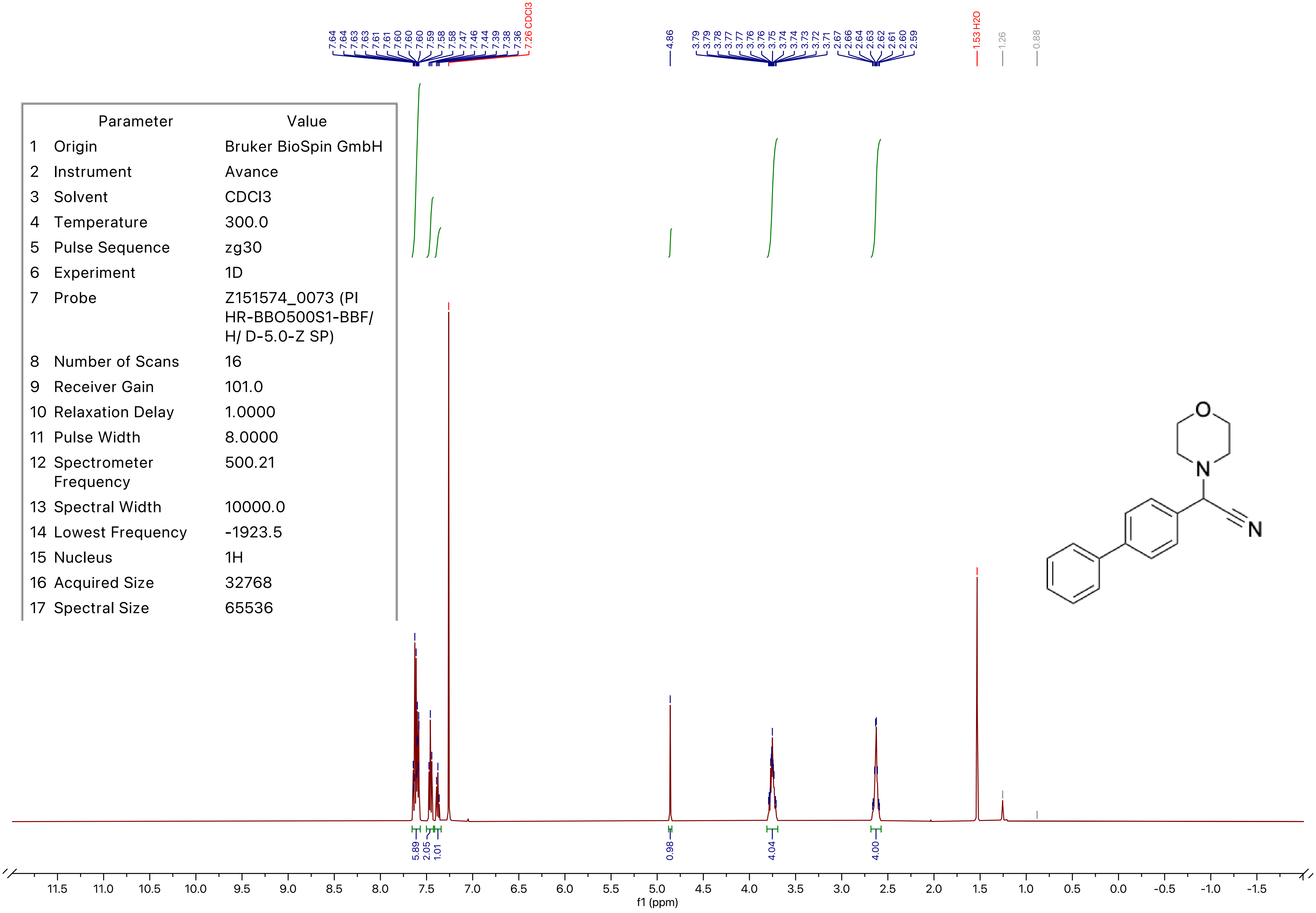

	Parameter	Value
1	Origin	Bruker BioSpin GmbH
2	Instrument	Avance
3	Solvent	CDCl3
4	Temperature	300.0
5	Pulse Sequence	zgpg30
6	Experiment	1D
7	Probe	Z151574_0073 (PI HR-BBO500S1-BBF/ H/ D-5.0-Z SP)
8	Number of Scans	1024
9	Receiver Gain	101.0
10	Relaxation Delay	2.0000
11	Pulse Width	9.0000
12	Spectrometer Frequency	125.79
13	Spectral Width	30120.5
14	Lowest Frequency	-2463.4
15	Nucleus	13C
16	Acquired Size	32768
17	Spectral Size	65536

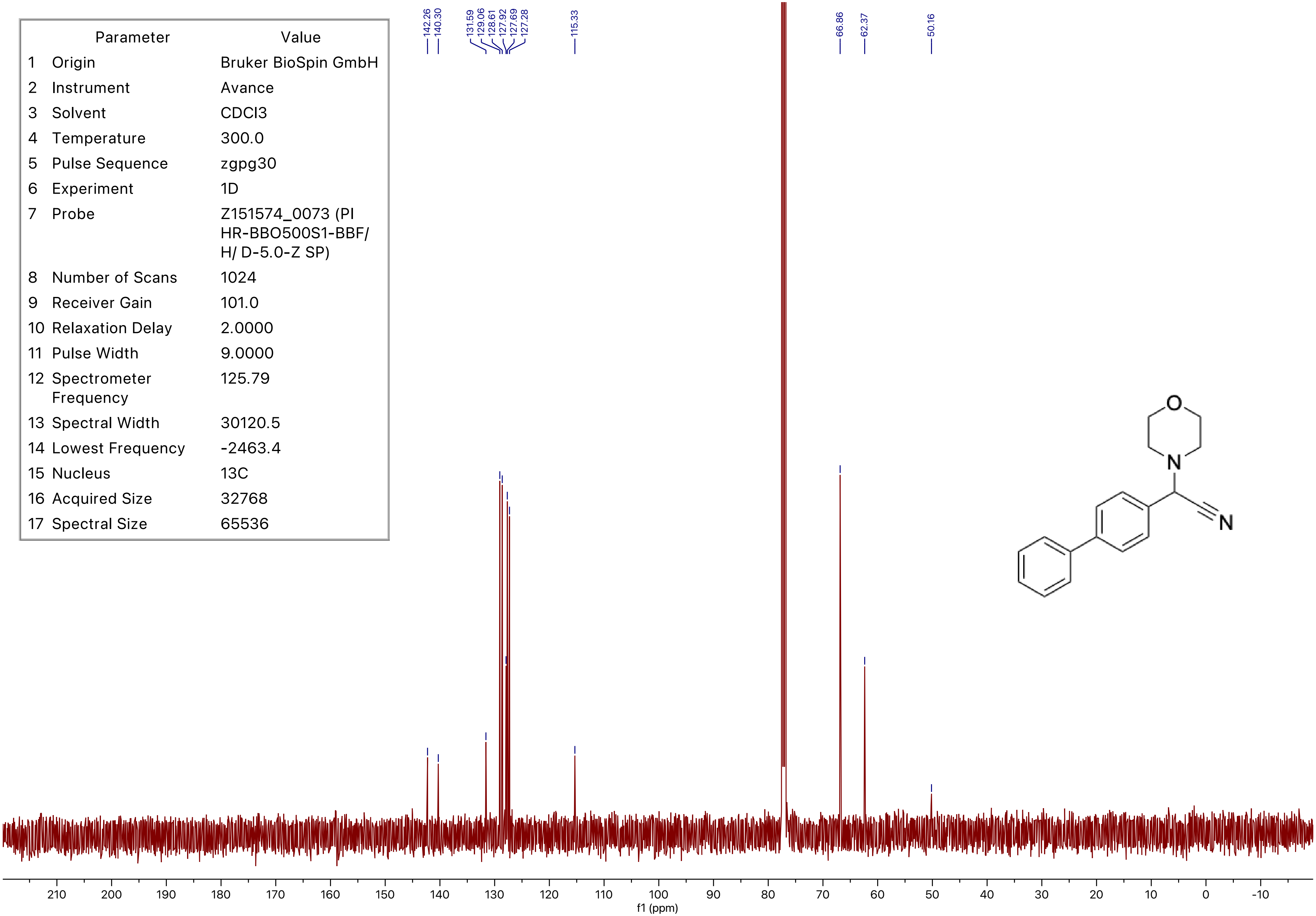

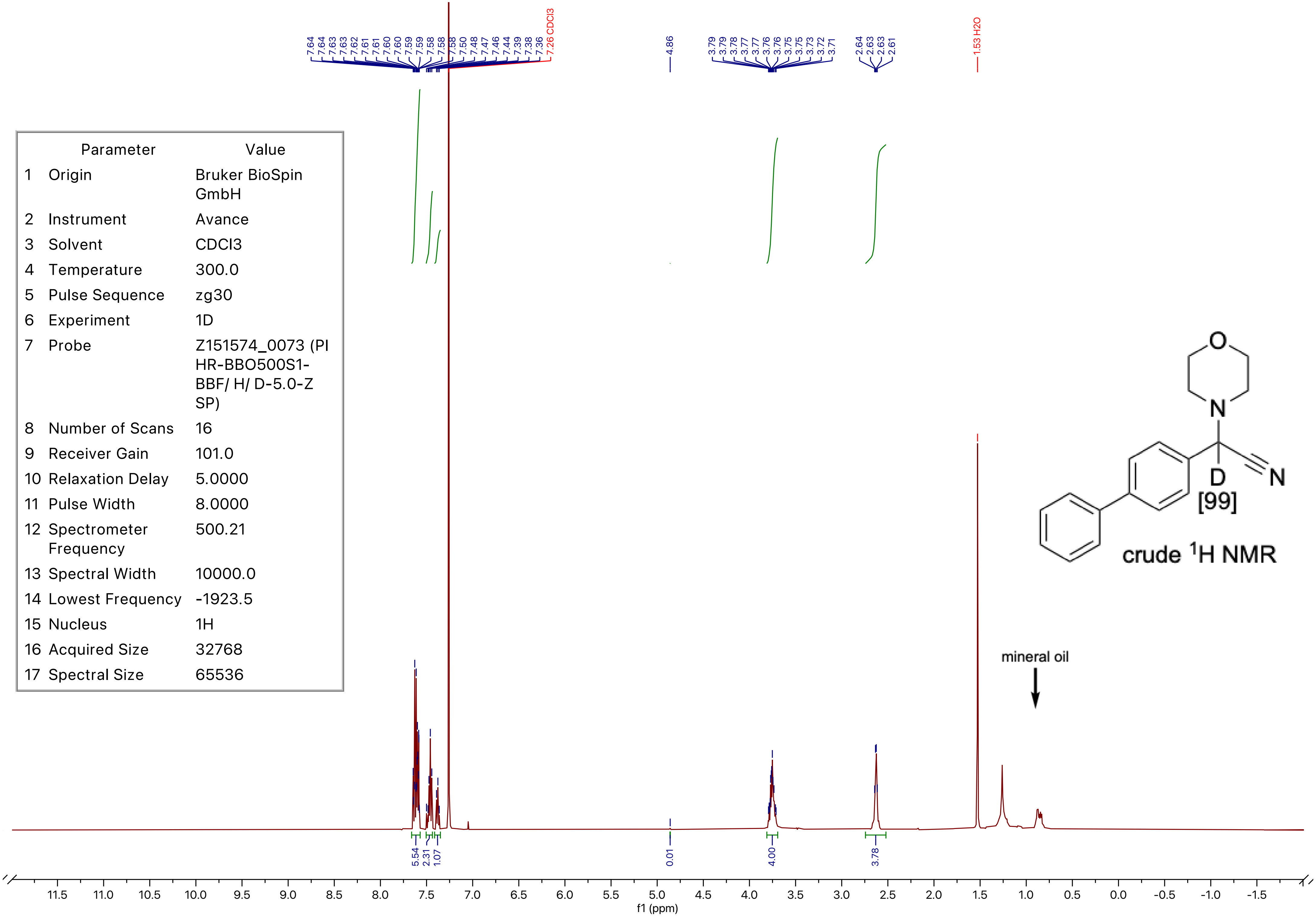

	Parameter	Value
1	Origin	Bruker BioSpin GmbH
2	Instrument	Avance
3	Solvent	CDCl3
4	Temperature	300.0
5	Pulse Sequence	zg30
6	Experiment	1D
7	Probe	Z151574_0073 (PI HR-BBO500S1-BBF/ H/ D-5.0-Z SP)
8	Number of Scans	16
9	Receiver Gain	101.0
10	Relaxation Delay	5.0000
11	Pulse Width	8.0000
12	Spectrometer Frequency	500.21
13	Spectral Width	10000.0
14	Lowest Frequency	-1923.5
15	Nucleus	1H
16	Acquired Size	32768
17	Spectral Size	65536

crude ^{1}H NMR

	Parameter	Value
1	Origin	Bruker BioSpin GmbH
2	Instrument	Avance
3	Solvent	CDCl3
4	Temperature	300.0
5	Pulse Sequence	zg30
6	Experiment	1D
7	Probe	Z151574_0073 (PI HR-BBO500S1-BBF/ H/ D-5.0-Z SP)
8	Number of Scans	16
9	Receiver Gain	101.0
10	Relaxation Delay	10.0000
11	Pulse Width	8.0000
12	Spectrometer Frequency	500.21
13	Spectral Width	10000.0
14	Lowest Frequency	-1923.4
15	Nucleus	1H
16	Acquired Size	32768
17	Spectral Size	65536

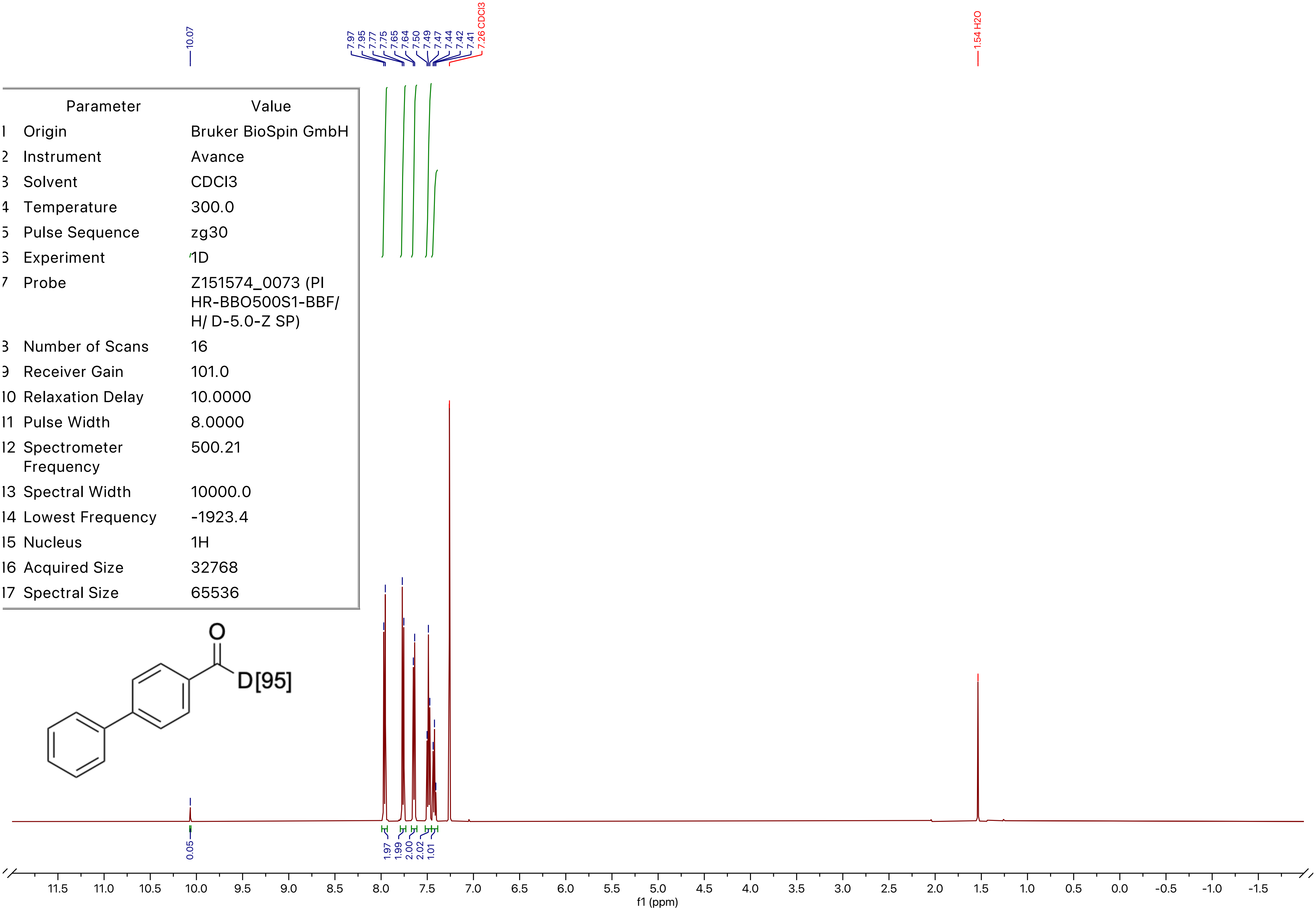

	Parameter	Value
1	Origin	Bruker BioSpin GmbH
2	Instrument	Avance
3	Solvent	CDCl3
4	Temperature	300.0
5	Pulse Sequence	zgpg30
6	Experiment	1D
7	Probe	Z151574_0073 (PI HR-BBO500S1-BBF/ H/ D-5.0-Z SP)
8	Number of Scans	1024
9	Receiver Gain	101.0
10	Relaxation Delay	2.0000
11	Pulse Width	9.0000
12	Spectrometer Frequency	125.79
13	Spectral Width	30120.5
14	Lowest Frequency	-2463.9
15	Nucleus	13C
16	Acquired Size	32768
17	Spectral Size	65536

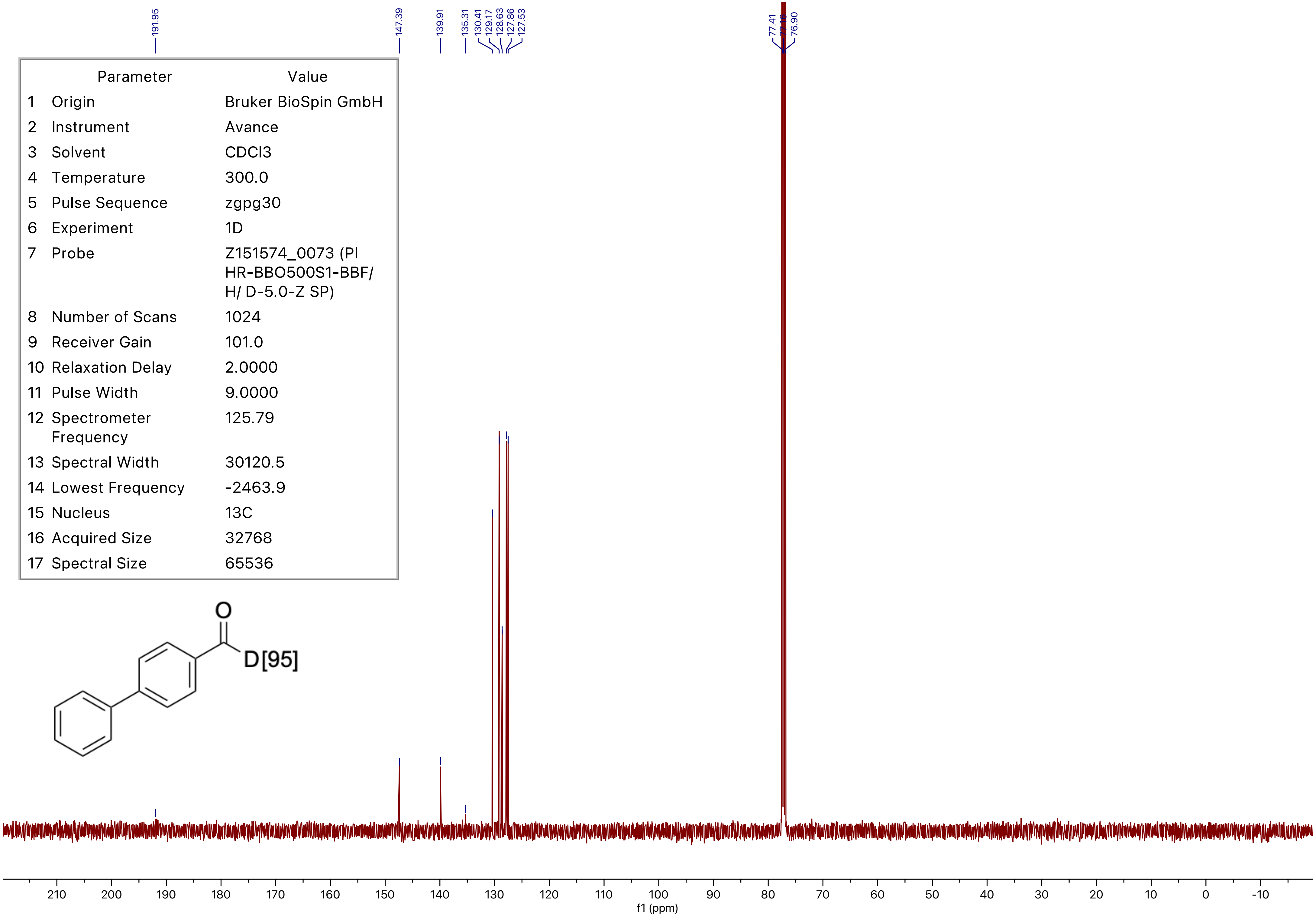

	Parameter	Value
1	Origin	Bruker BioSpin GmbH
2	Instrument	Avance
3	Solvent	CDCl3
4	Temperature	300.0
5	Pulse Sequence	zg30
6	Experiment	1D
7	Probe	Z151574_0073 (PI HR-BBO500S1-BBF/ H/ D-5.0-Z SP)
8	Number of Scans	16
9	Receiver Gain	101.0
10	Relaxation Delay	1.0000
11	Pulse Width	8.0000
12	Spectrometer Frequency	500.21
13	Spectral Width	10000.0
14	Lowest Frequency	-1911.2
15	Nucleus	1H
16	Acquired Size	32768
17	Spectral Size	65536

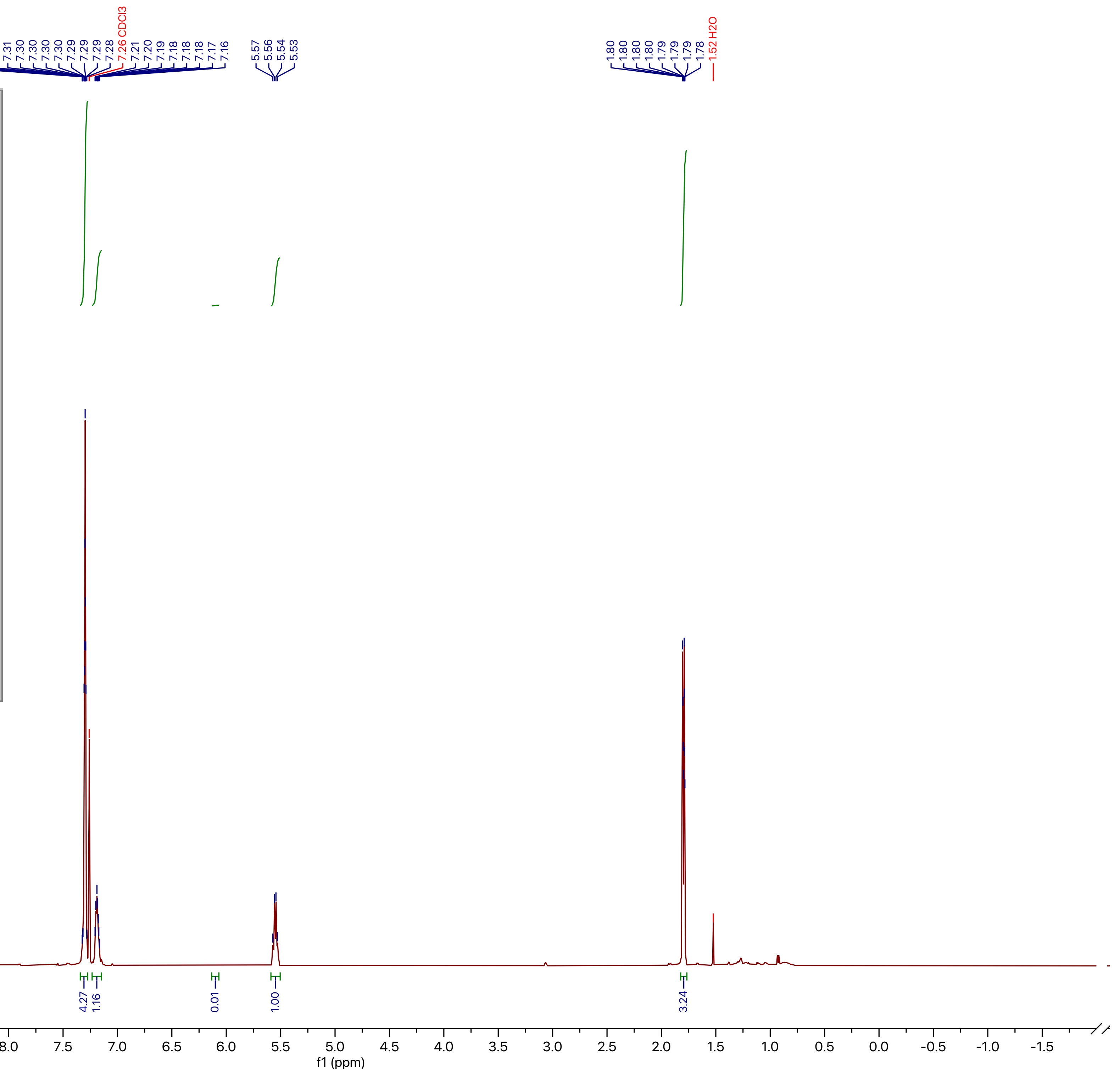

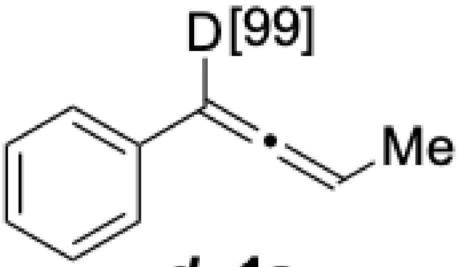

***d*$_1$-1a**

Parameter		Value
1	Origin	Bruker BioSpin GmbH
2	Instrument	Avance
3	Solvent	CDCl3
4	Temperature	300.0
5	Pulse Sequence	zgpg30
6	Experiment	1D
7	Probe	Z151574_0073 (PI HR-BBO500S1-BBF/ H/ D-5.0-Z SP)
8	Number of Scans	1024
9	Receiver Gain	101.0
10	Relaxation Delay	2.0000
11	Pulse Width	9.0000
12	Spectrometer Frequency	125.79
13	Spectral Width	30120.5
14	Lowest Frequency	-2482.4
15	Nucleus	13C
16	Acquired Size	32768
17	Spectral Size	65536

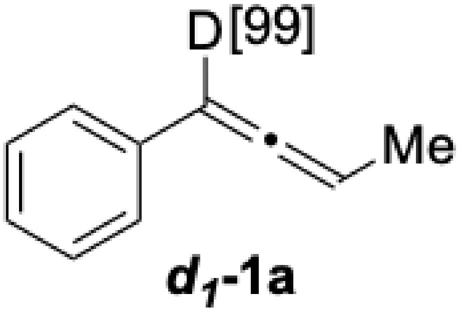

***d*$_1$-1a**

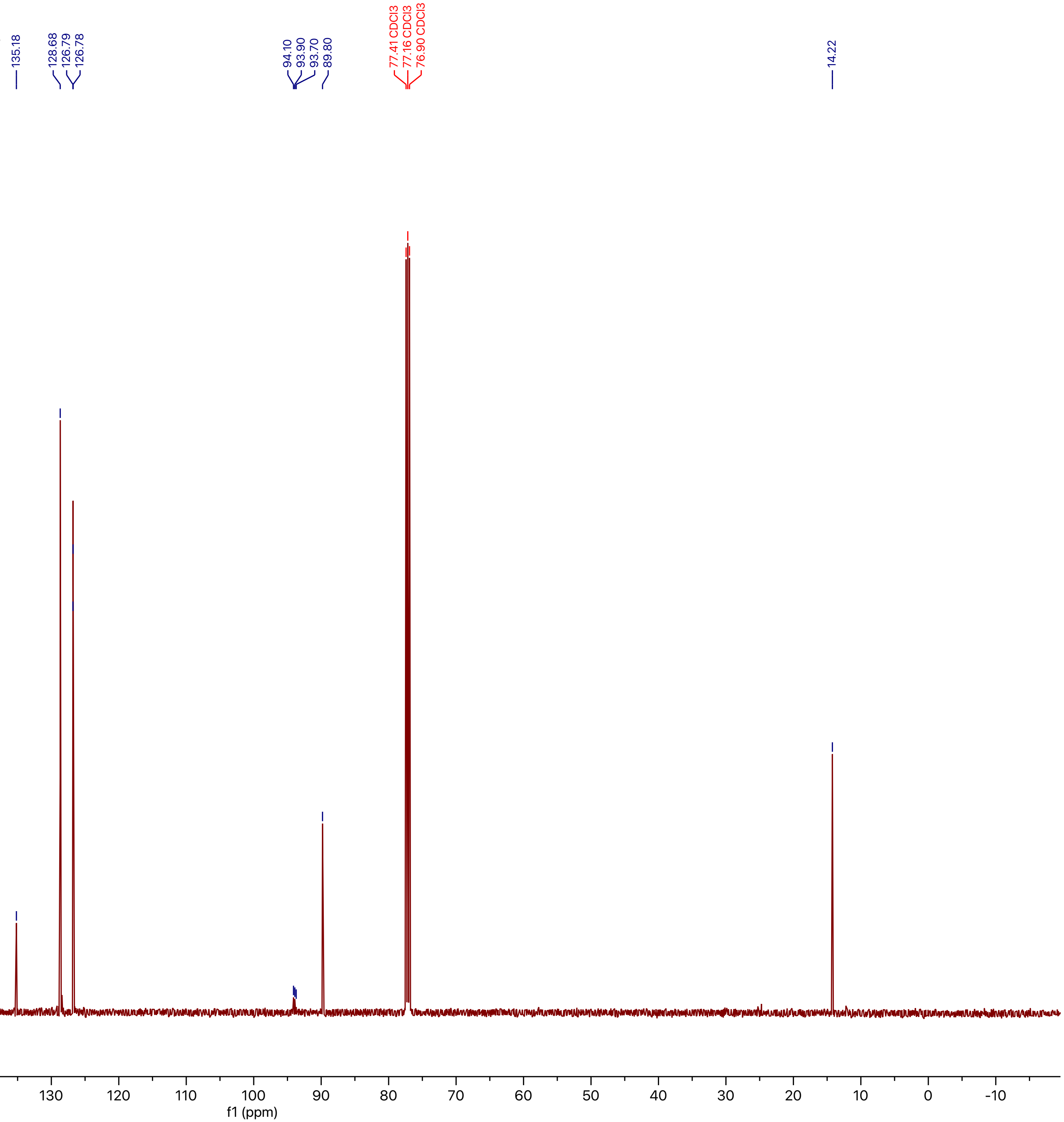

	Parameter	Value
1	Origin	Bruker BioSpin GmbH
2	Instrument	Avance
3	Solvent	DMSO
4	Temperature	300.0
5	Pulse Sequence	zg30
6	Experiment	1D
7	Probe	Z151574_0073 (PI HR-BBO500S1-BBF/ H/ D-5.0-Z SP)
8	Number of Scans	64
9	Receiver Gain	101.0
10	Relaxation Delay	1.0000
11	Pulse Width	8.0000
12	Spectrometer Frequency	500.21
13	Spectral Width	10000.0
14	Lowest Frequency	-2028.1
15	Nucleus	1H
16	Acquired Size	32768
17	Spectral Size	65536

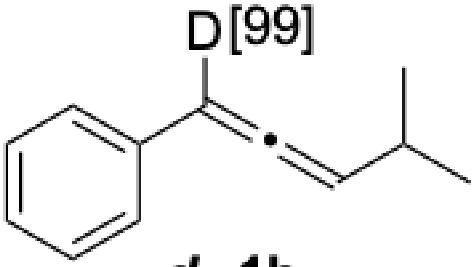

d_1-1b

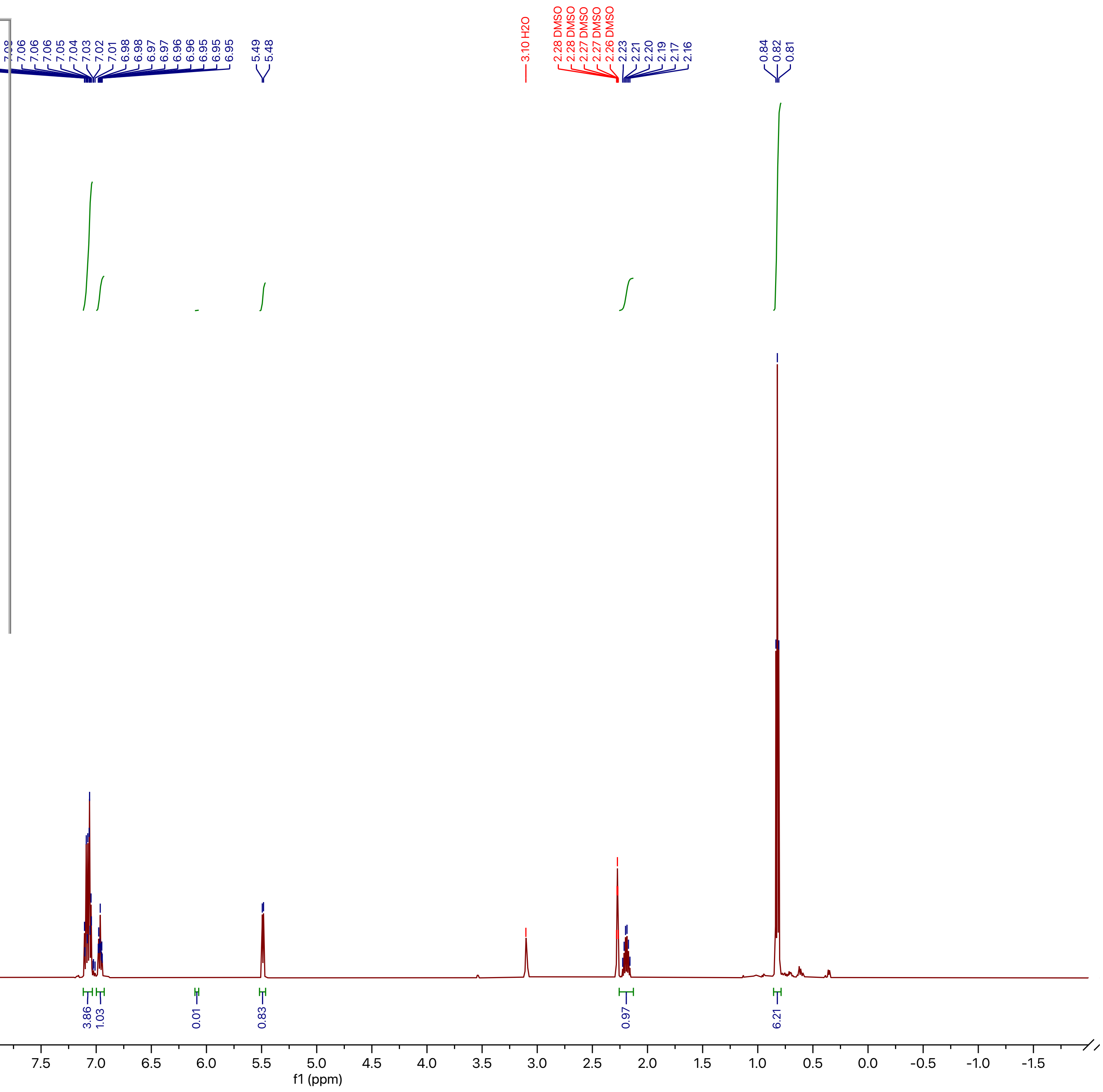

	Parameter	Value
1	Origin	Bruker BioSpin GmbH
2	Instrument	Avance
3	Solvent	DMSO
4	Temperature	300.0
5	Pulse Sequence	zgpg30
6	Experiment	1D
7	Probe	Z151574_0073 (PI HR-BBO500S1-BBF/H/ D-5.0-Z SP)
8	Number of Scans	1024
9	Receiver Gain	101.0
10	Relaxation Delay	2.0000
11	Pulse Width	9.0000
12	Spectrometer Frequency	125.79
13	Spectral Width	30120.5
14	Lowest Frequency	-2482.4
15	Nucleus	13C
16	Acquired Size	32768
17	Spectral Size	65536

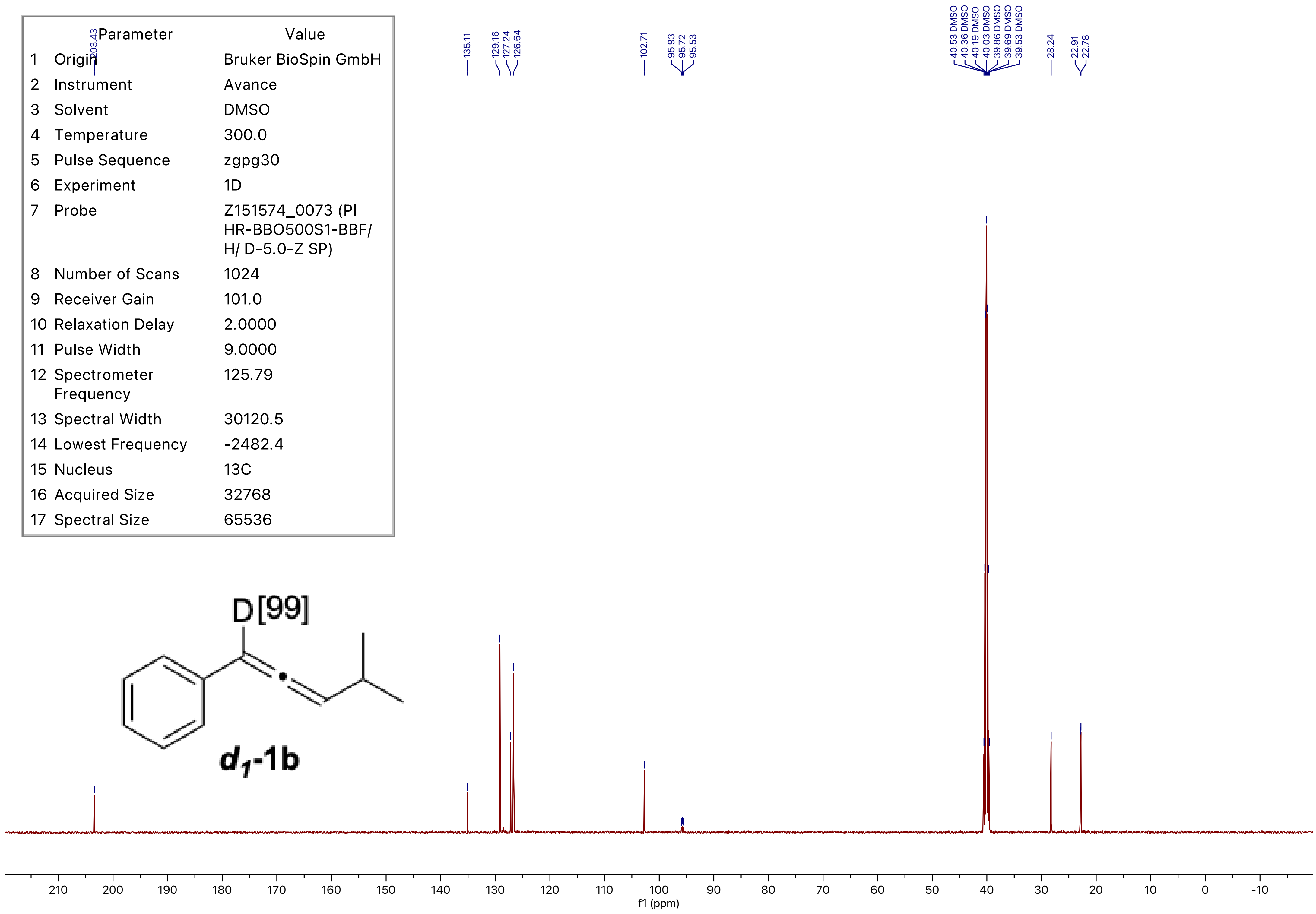

d_1-1b

	Parameter	Value
1	Origin	Bruker BioSpin GmbH
2	Instrument	Avance
3	Solvent	CDCl3
4	Temperature	300.0
5	Pulse Sequence	zg30
6	Experiment	1D
7	Probe	Z151574_0073 (PI HR-BBO500S1-BBF/ H/ D-5.0-Z SP)
8	Number of Scans	16
9	Receiver Gain	101.0
10	Relaxation Delay	1.0000
11	Pulse Width	8.0000
12	Spectrometer Frequency	500.21
13	Spectral Width	10000.0
14	Lowest Frequency	-1911.2
15	Nucleus	1H
16	Acquired Size	32768
17	Spectral Size	65536

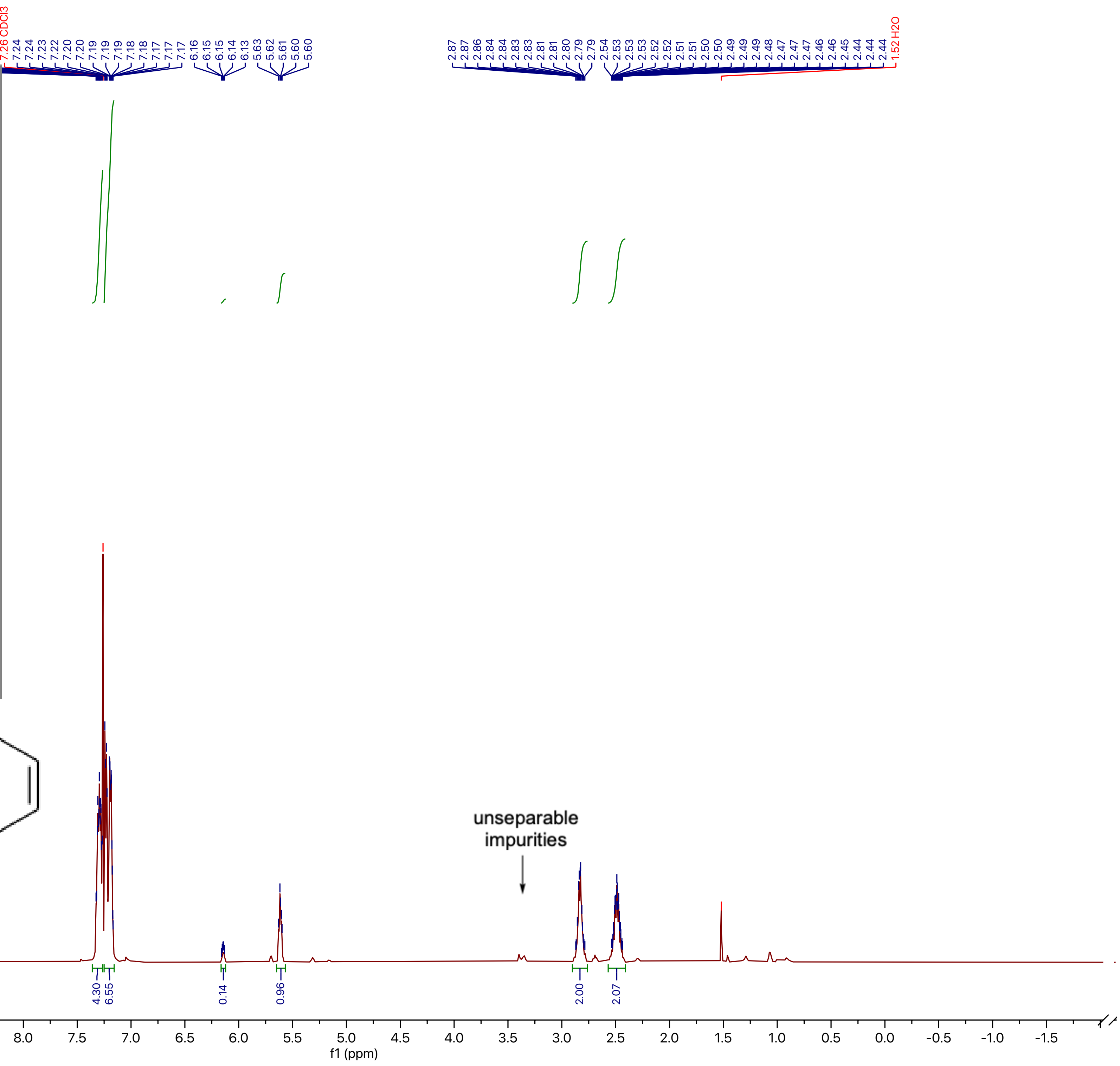

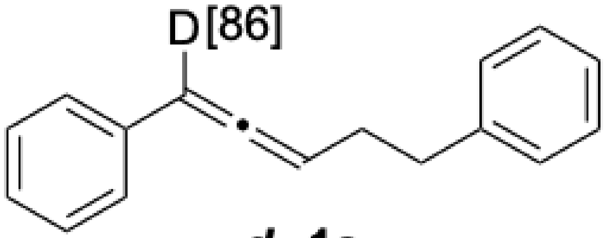

***d_1*-1c**

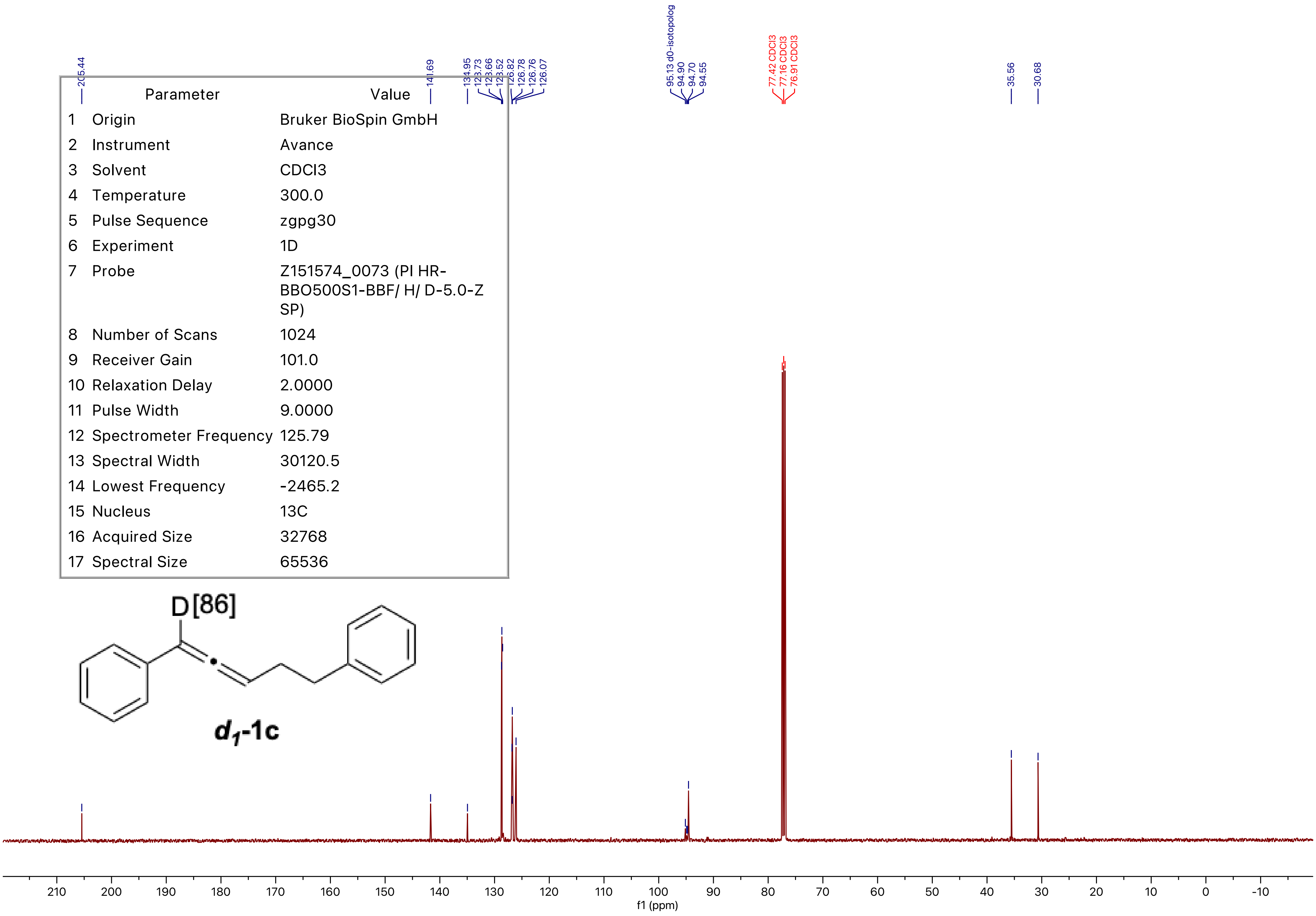

	Parameter	Value
1	Origin	Bruker BioSpin GmbH
2	Instrument	Avance
3	Solvent	CDCl3
4	Temperature	300.0
5	Pulse Sequence	zgpg30
6	Experiment	1D
7	Probe	Z151574_0073 (PI HR-BBO500S1-BBF/ H/ D-5.0-Z SP)
8	Number of Scans	1024
9	Receiver Gain	101.0
10	Relaxation Delay	2.0000
11	Pulse Width	9.0000
12	Spectrometer Frequency	125.79
13	Spectral Width	30120.5
14	Lowest Frequency	-2465.2
15	Nucleus	13C
16	Acquired Size	32768
17	Spectral Size	65536

***d_1*-1c**

	Parameter	Value
1	Origin	Bruker BioSpin GmbH
2	Instrument	Avance
3	Solvent	CDCl3
4	Temperature	300.0
5	Pulse Sequence	zg30
6	Experiment	1D
7	Probe	Z151574_0073 (PI HR-BBO500S1-BBF/ H/ D-5.0-Z SP)
8	Number of Scans	16
9	Receiver Gain	101.0
10	Relaxation Delay	1.0000
11	Pulse Width	8.0000
12	Spectrometer Frequency	500.21
13	Spectral Width	10000.0
14	Lowest Frequency	-1923.4
15	Nucleus	1H
16	Acquired Size	32768
17	Spectral Size	65536

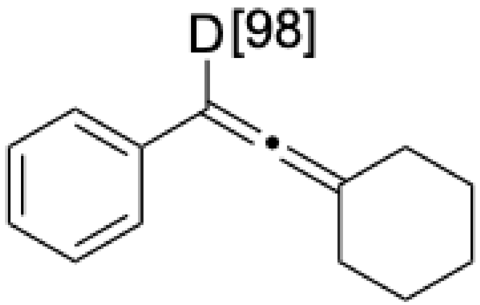

***d*₁-3a**

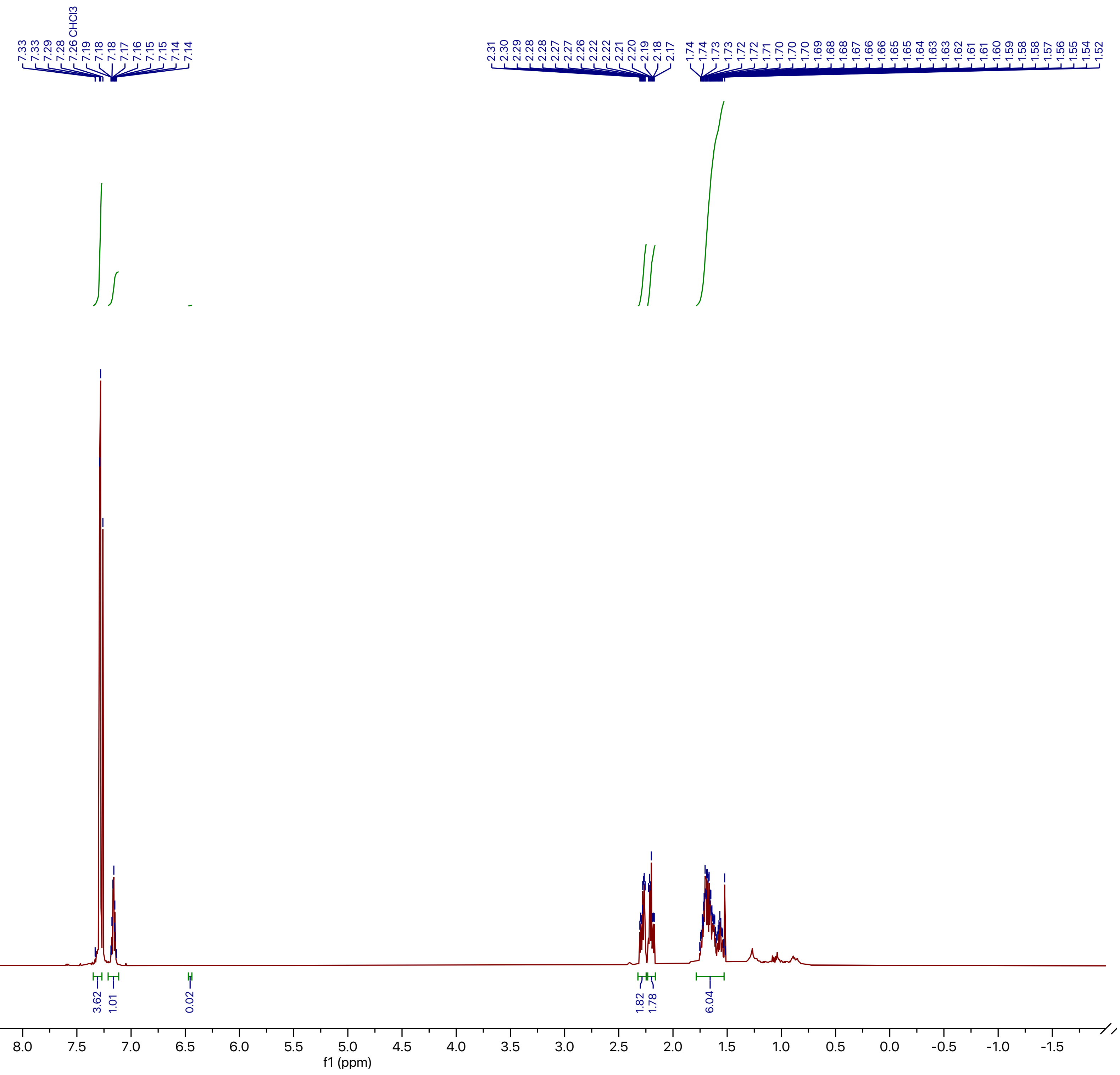

	Parameter	Value
1	Origin	Bruker BioSpin GmbH
2	Instrument	Avance
3	Solvent	CDCl3
4	Temperature	300.0
5	Pulse Sequence	zgpg30
6	Experiment	1D
7	Probe	Z151574_0073 (PI HR-BBO500S1-BBF/ H/ D-5.0-Z SP)
8	Number of Scans	1024
9	Receiver Gain	101.0
10	Relaxation Delay	10.0000
11	Pulse Width	9.0000
12	Spectrometer Frequency	125.79
13	Spectral Width	30120.5
14	Lowest Frequency	-2466.0
15	Nucleus	13C
16	Acquired Size	32768
17	Spectral Size	65536

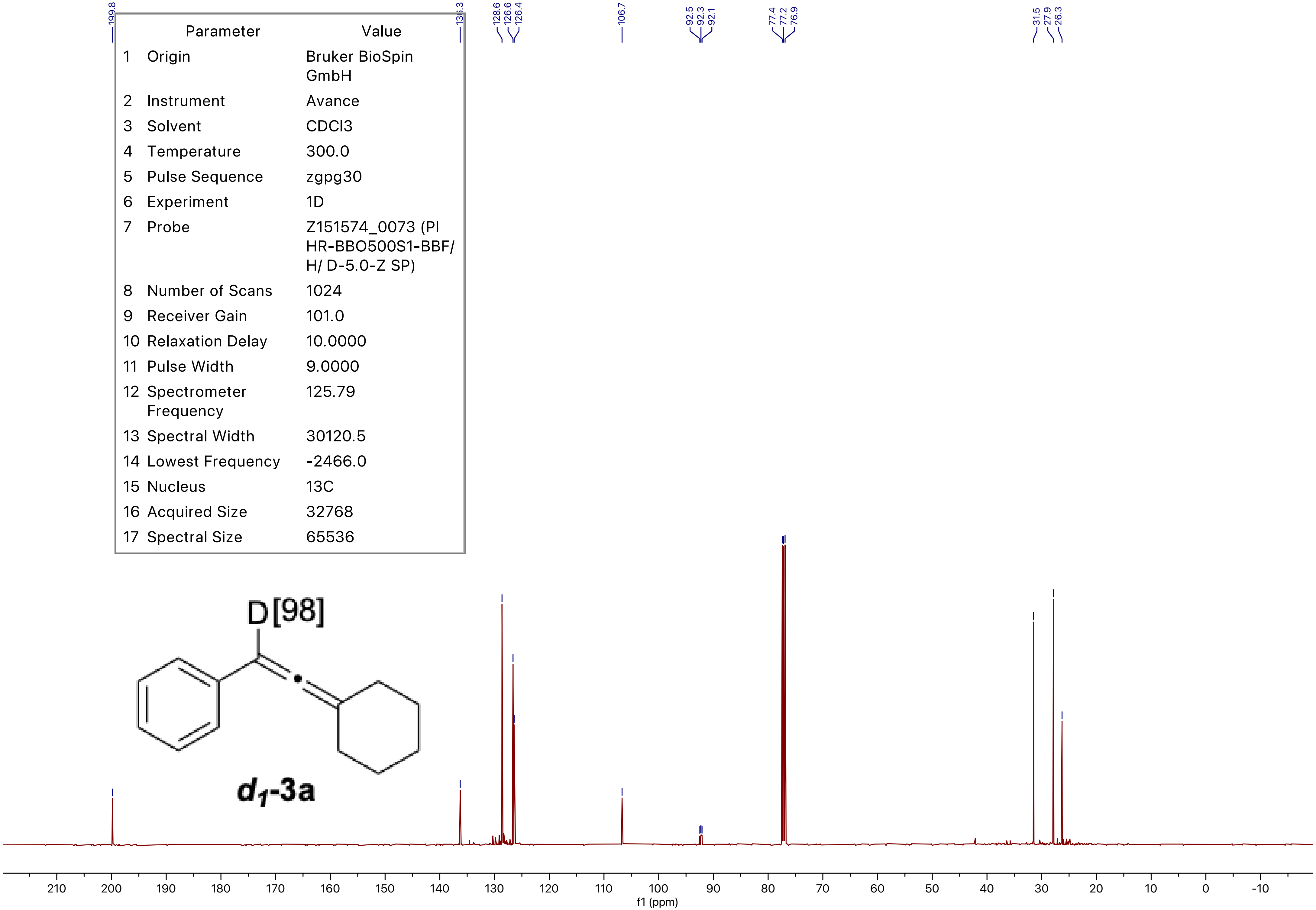

***d*$_1$-3a**

	Parameter	Value
1	Origin	Bruker BioSpin GmbH
2	Instrument	Avance
3	Solvent	CDCl3
4	Temperature	300.0
5	Pulse Sequence	zg30
6	Experiment	1D
7	Probe	Z151574_0073 (PI HR-BBO500S1-BBF/ H/ D-5.0-Z SP)
8	Number of Scans	16
9	Receiver Gain	101.0
10	Relaxation Delay	1.0000
11	Pulse Width	8.0000
12	Spectrometer Frequency	500.21
13	Spectral Width	10000.0
14	Lowest Frequency	-1911.2
15	Nucleus	1H
16	Acquired Size	32768
17	Spectral Size	65536

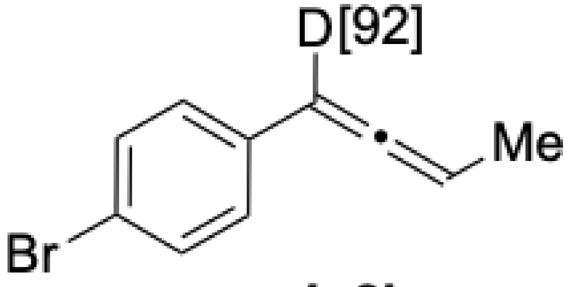

*d*₁-3b

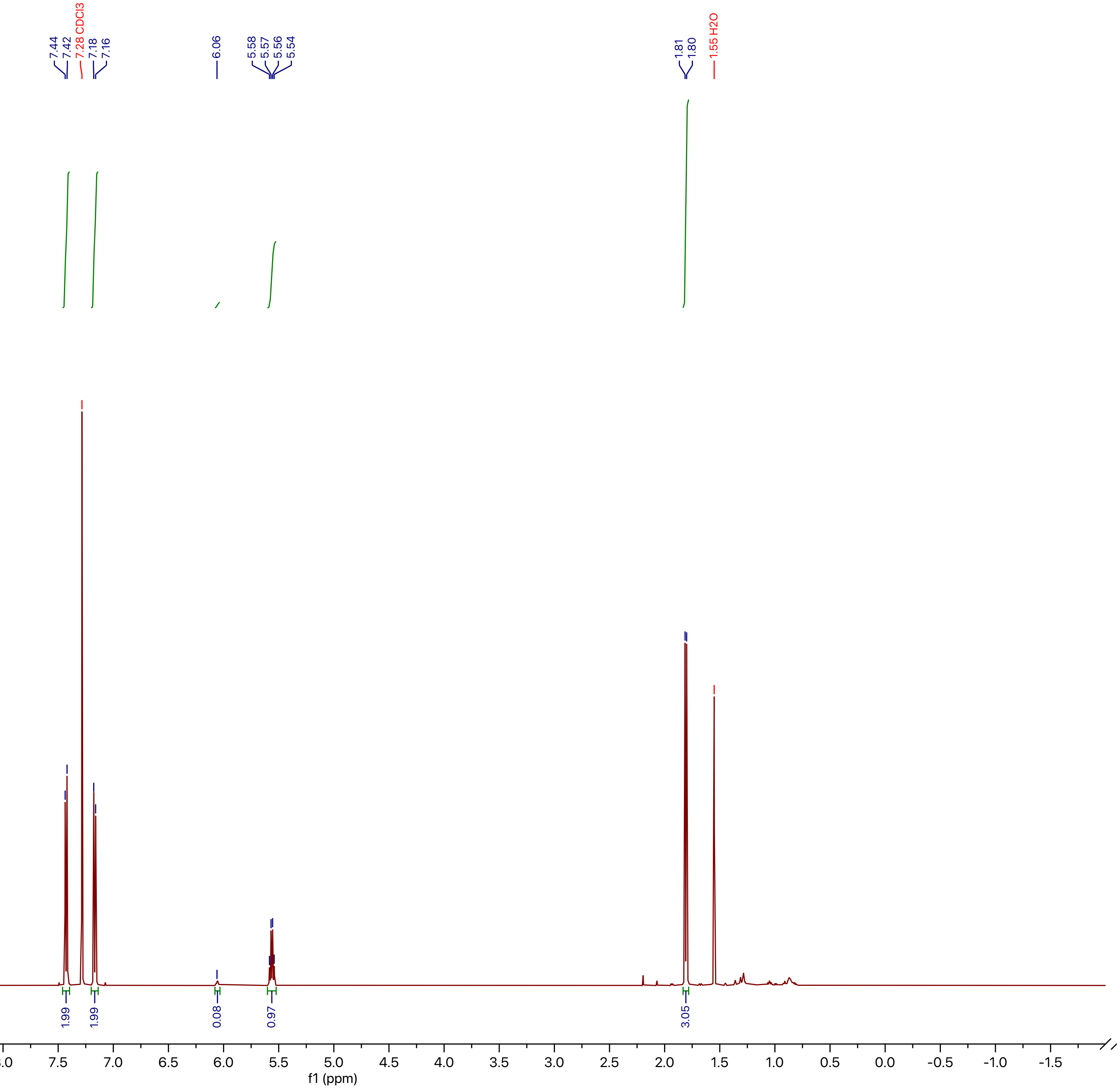

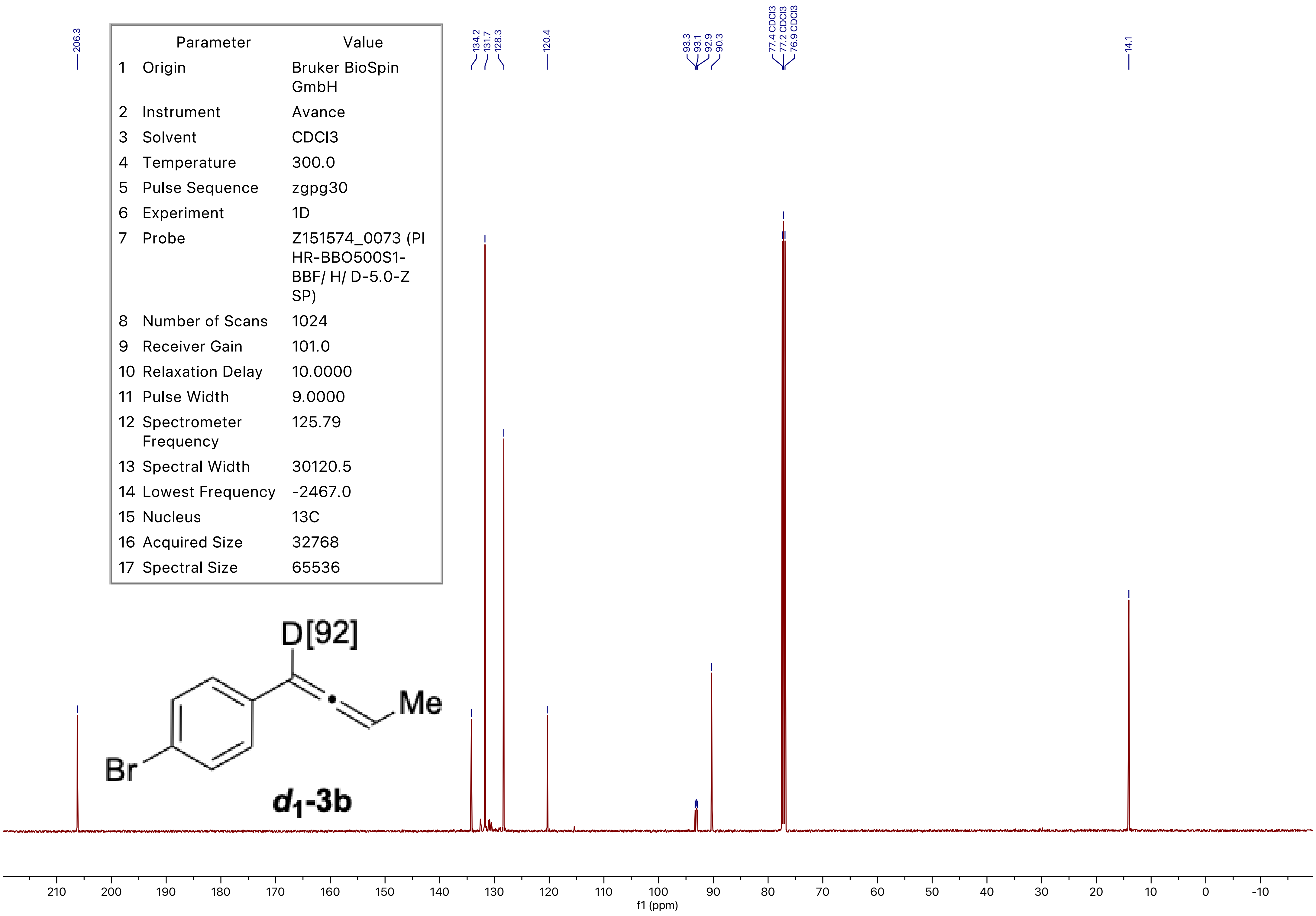

	Parameter	Value
1	Origin	Bruker BioSpin GmbH
2	Instrument	Avance
3	Solvent	CDCl3
4	Temperature	300.0
5	Pulse Sequence	zgpg30
6	Experiment	1D
7	Probe	Z151574_0073 (PI HR-BBO500S1-BBF/ H/ D-5.0-Z SP)
8	Number of Scans	1024
9	Receiver Gain	101.0
10	Relaxation Delay	10.0000
11	Pulse Width	9.0000
12	Spectrometer Frequency	125.79
13	Spectral Width	30120.5
14	Lowest Frequency	-2467.0
15	Nucleus	13C
16	Acquired Size	32768
17	Spectral Size	65536

***d*$_1$-3b**

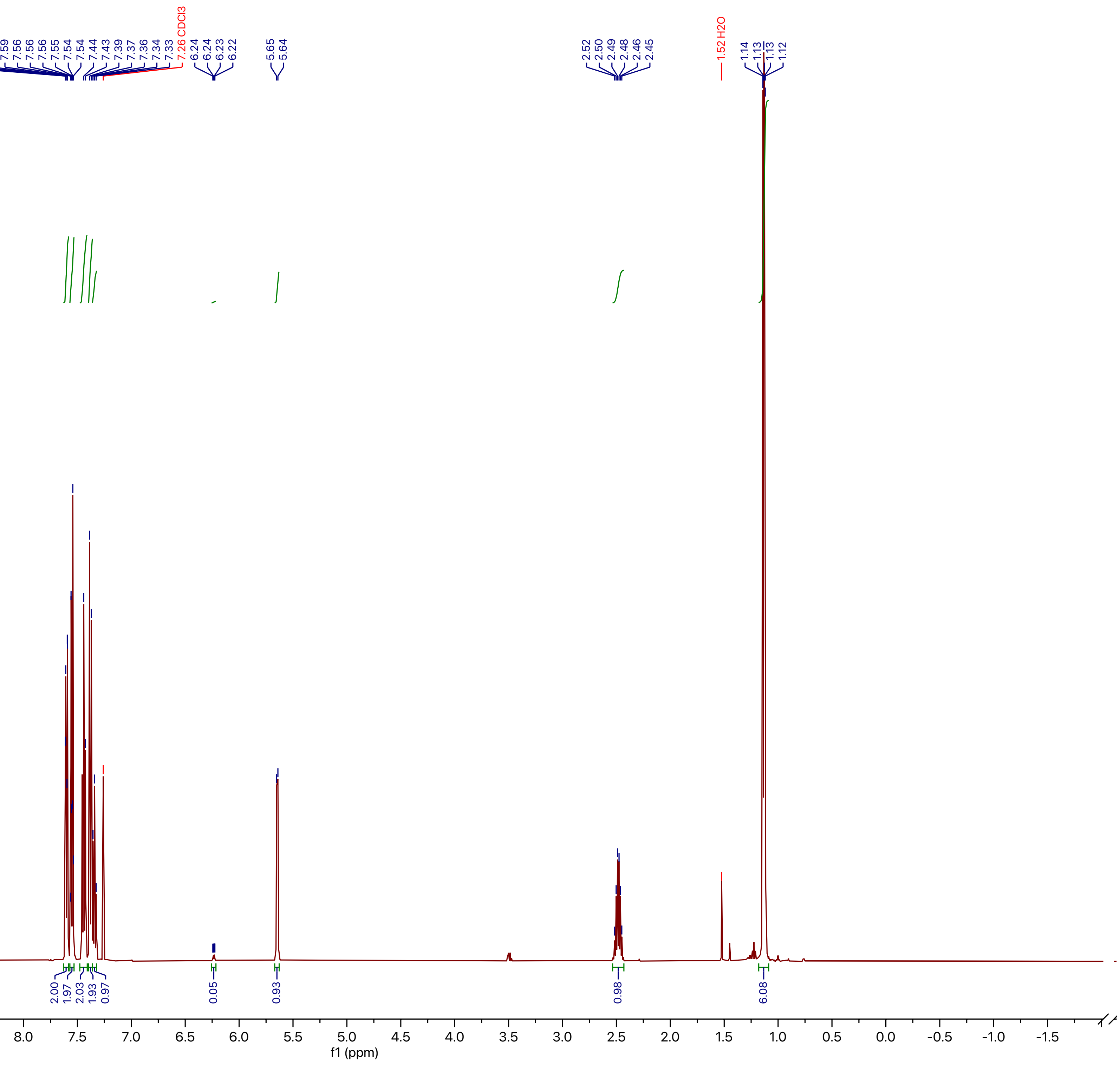

	Parameter	Value
1	Origin	Bruker BioSpin GmbH
2	Instrument	Avance
3	Solvent	CDCl3
4	Temperature	300.0
5	Pulse Sequence	zg30
6	Experiment	1D
7	Probe	Z151574_0073 (PI HR-BBO500S1-BBF/ H/ D-5.0-Z SP)
8	Number of Scans	16
9	Receiver Gain	101.0
10	Relaxation Delay	1.0000
11	Pulse Width	8.0000
12	Spectrometer Frequency	500.21
13	Spectral Width	10000.0
14	Lowest Frequency	-1923.6
15	Nucleus	1H
16	Acquired Size	32768
17	Spectral Size	65536

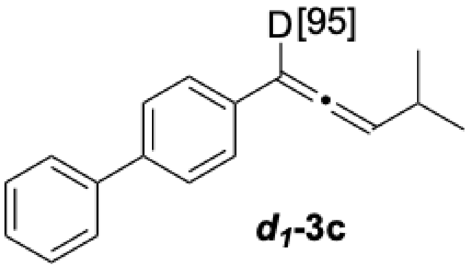

d_1-3c

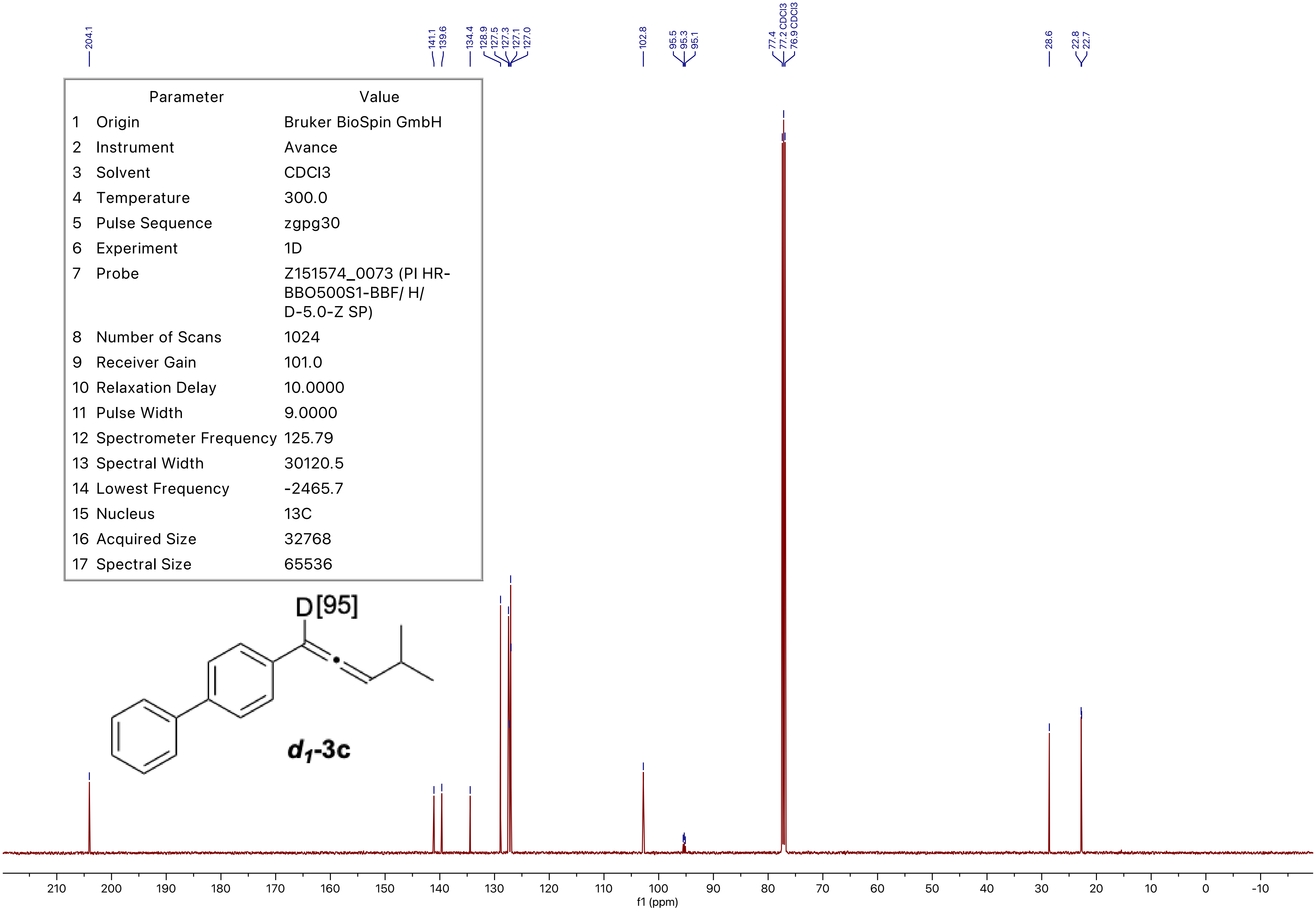

	Parameter	Value
1	Origin	Bruker BioSpin GmbH
2	Instrument	Avance
3	Solvent	CDCl3
4	Temperature	300.0
5	Pulse Sequence	zgpg30
6	Experiment	1D
7	Probe	Z151574_0073 (PI HR-BBO500S1-BBF/ H/ D-5.0-Z SP)
8	Number of Scans	1024
9	Receiver Gain	101.0
10	Relaxation Delay	10.0000
11	Pulse Width	9.0000
12	Spectrometer Frequency	125.79
13	Spectral Width	30120.5
14	Lowest Frequency	-2465.7
15	Nucleus	13C
16	Acquired Size	32768
17	Spectral Size	65536

***d_1*-3c**

	Parameter	Value
1	Origin	Bruker BioSpin GmbH
2	Instrument	Avance
3	Solvent	CDCl3
4	Temperature	298.0
5	Pulse Sequence	zg30
6	Experiment	1D
7	Probe	Z151574_0073 (PI HR-BBO500S1-BBF/ H/ D-5.0-Z SP)
8	Number of Scans	16
9	Receiver Gain	101.0
10	Relaxation Delay	1.0000
11	Pulse Width	8.0000
12	Spectrometer Frequency	500.21
13	Spectral Width	10000.0
14	Lowest Frequency	-1923.2
15	Nucleus	1H
16	Acquired Size	32768
17	Spectral Size	65536

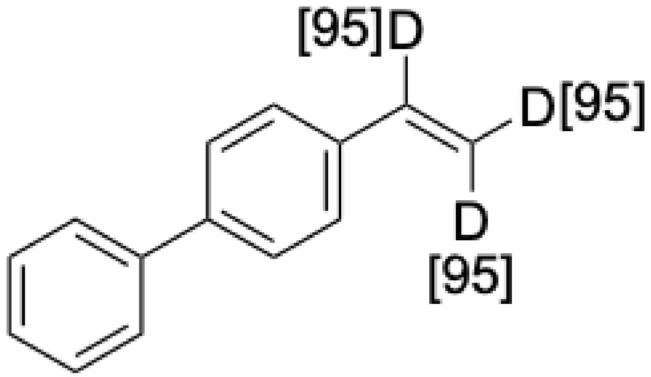

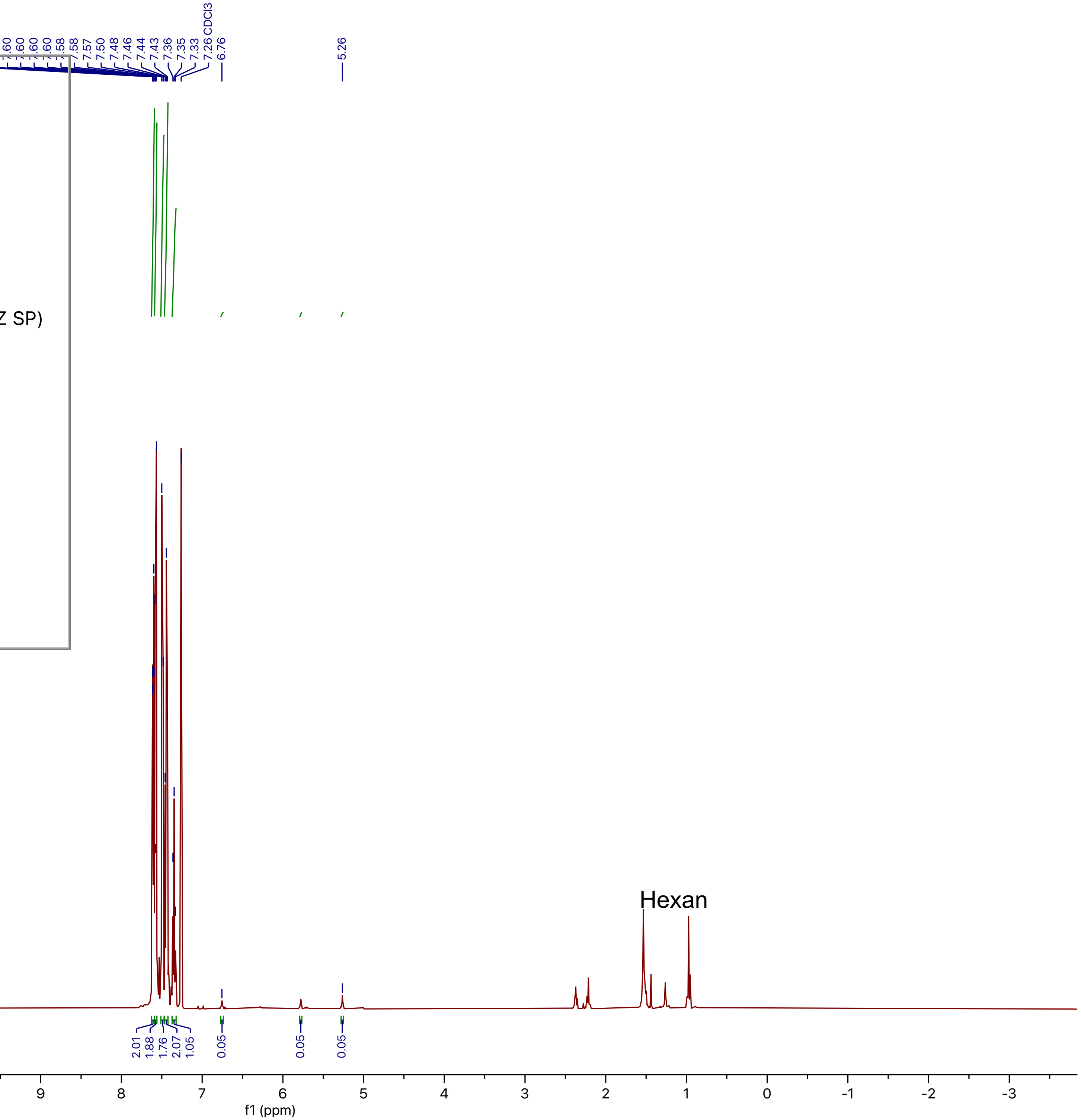

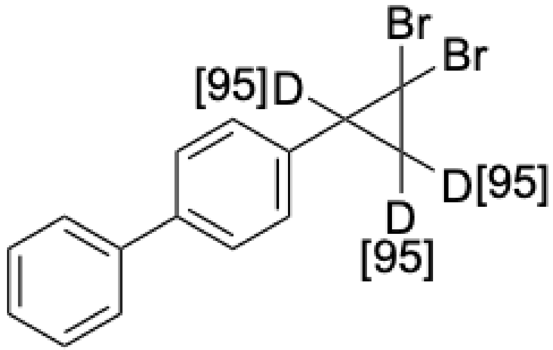

	Parameter	Value
1	Origin	Bruker BioSpin GmbH
2	Instrument	Avance
3	Solvent	CDCl3
4	Temperature	298.0
5	Pulse Sequence	zg30
6	Experiment	1D
7	Probe	Z151574_0073 (PI HR-BBO500S1-BBF/ H/ D-5.0-Z SP)
8	Number of Scans	16
9	Receiver Gain	101.0
10	Relaxation Delay	5.0000
11	Pulse Width	8.0000
12	Spectrometer Frequency	500.21
13	Spectral Width	10000.0
14	Lowest Frequency	-1923.4
15	Nucleus	1H
16	Acquired Size	32768
17	Spectral Size	65536

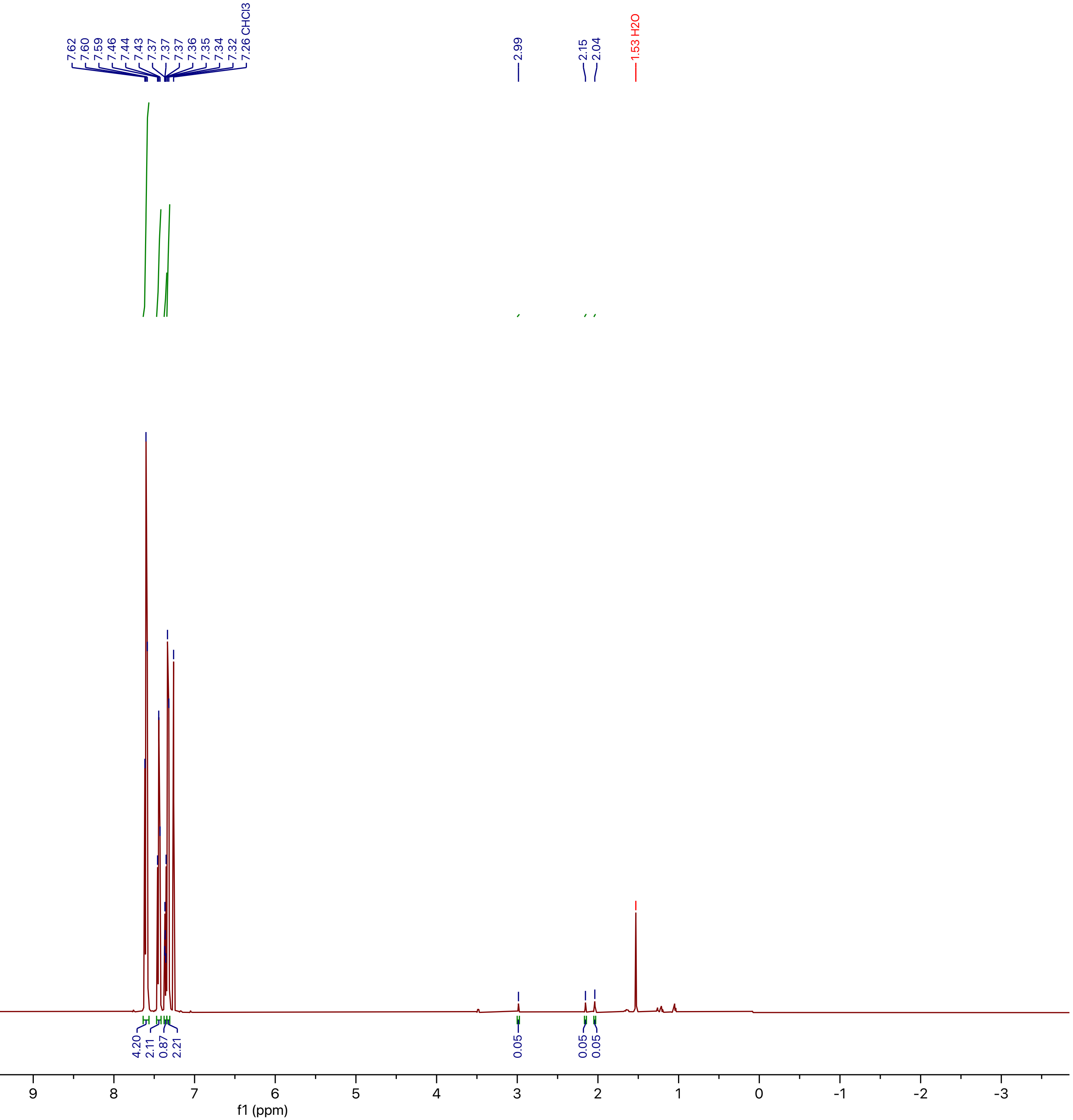

	Parameter	Value
1	Origin	Bruker BioSpin GmbH
2	Instrument	Avance
3	Solvent	CDCl3
4	Temperature	298.0
5	Pulse Sequence	zg30
6	Experiment	1D
7	Probe	Z151574_0073 (PI HR-BBO500S1-BBF/ H/ D-5.0-Z SP)
8	Number of Scans	16
9	Receiver Gain	101.0
10	Relaxation Delay	1.0000
11	Pulse Width	8.0000
12	Spectrometer Frequency	500.21
13	Spectral Width	10000.0
14	Lowest Frequency	-1923.4
15	Nucleus	1H
16	Acquired Size	32768
17	Spectral Size	65536

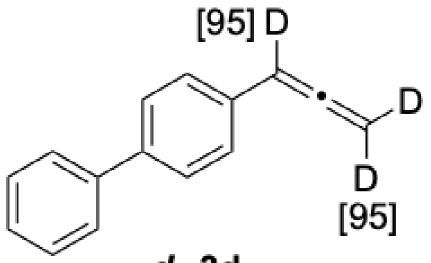

d$_3$-**3d**

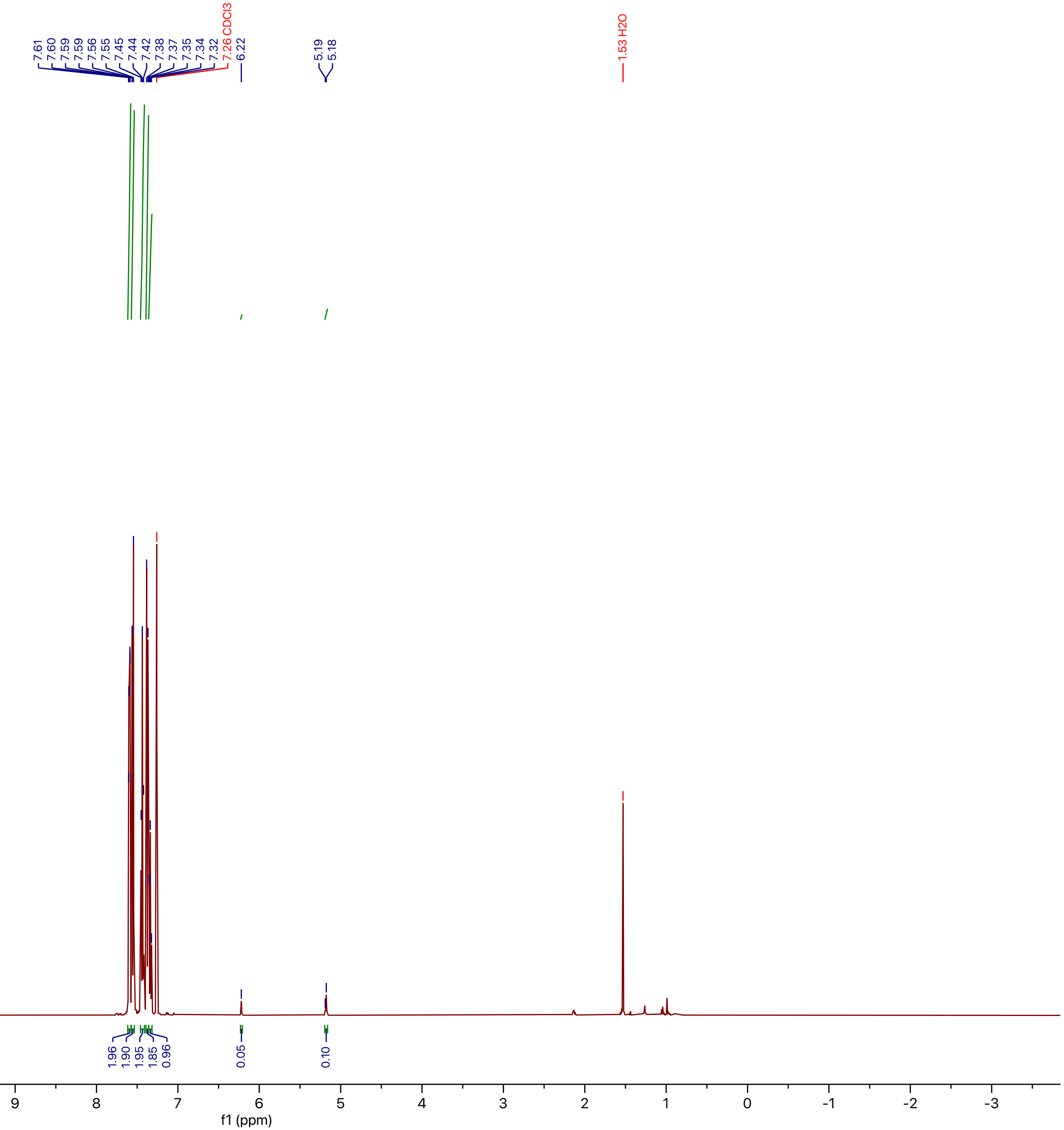

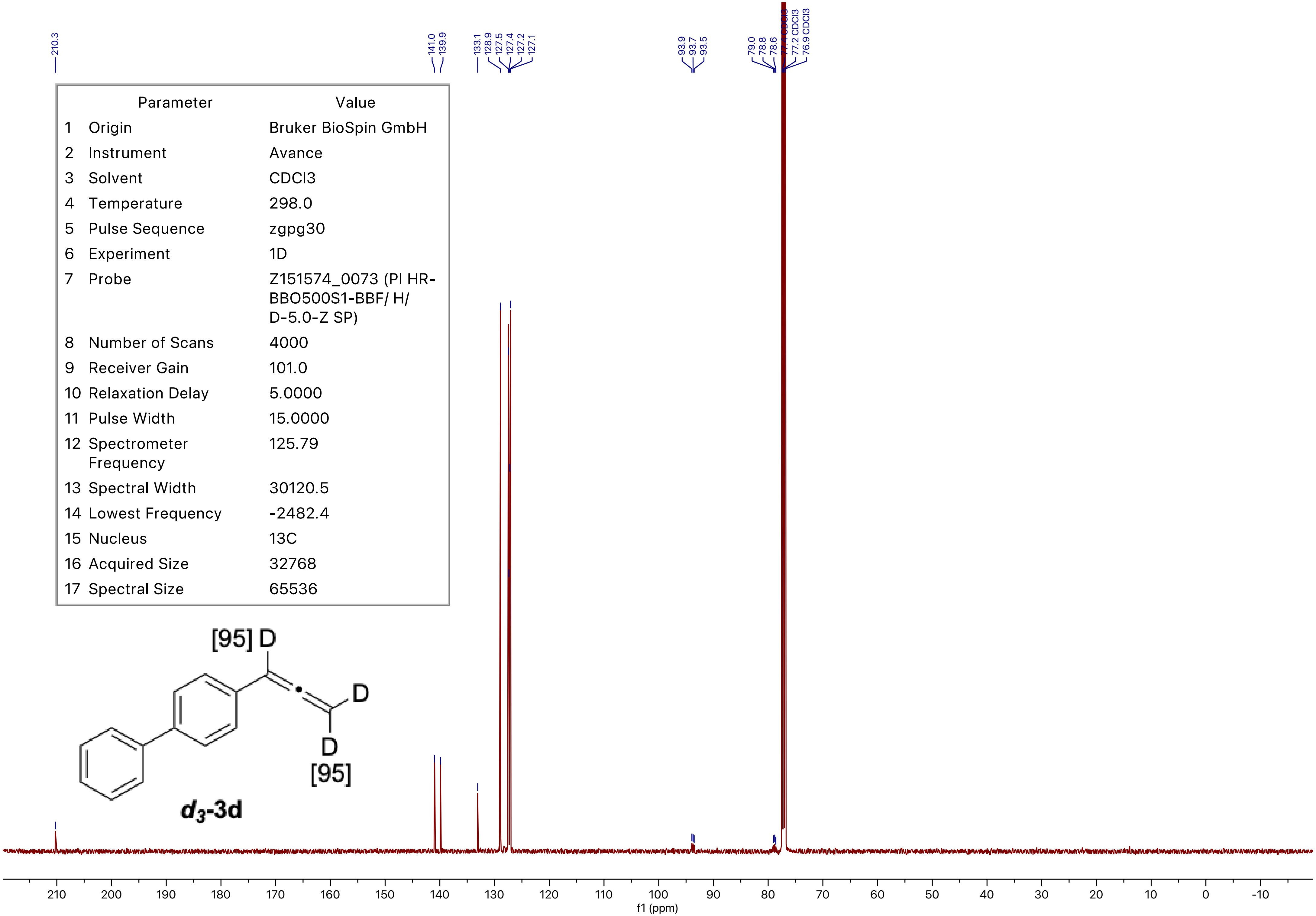

	Parameter	Value
1	Origin	Bruker BioSpin GmbH
2	Instrument	Avance
3	Solvent	CDCl3
4	Temperature	298.0
5	Pulse Sequence	zgpg30
6	Experiment	1D
7	Probe	Z151574_0073 (PI HR-BBO500S1-BBF/ H/ D-5.0-Z SP)
8	Number of Scans	4000
9	Receiver Gain	101.0
10	Relaxation Delay	5.0000
11	Pulse Width	15.0000
12	Spectrometer Frequency	125.79
13	Spectral Width	30120.5
14	Lowest Frequency	-2482.4
15	Nucleus	13C
16	Acquired Size	32768
17	Spectral Size	65536

***d*$_3$-3d**
